# Implementation of genomic surveillance of SARS-CoV-2 in the Caribbean: Lessons learned for sustainability in resource-limited settings

**DOI:** 10.1371/journal.pgph.0001455

**Published:** 2023-02-22

**Authors:** Nikita S. D. Sahadeo, Soren Nicholls, Filipe R. R. Moreira, Áine O’Toole, Vernie Ramkissoon, Charles Whittaker, Verity Hill, John T. McCrone, Nicholas Mohammed, Anushka Ramjag, Arianne Brown Jordan, Sarah C. Hill, Risha Singh, Sue-Min Nathaniel-Girdharrie, Avery Hinds, Nuala Ramkissoon, Kris V. Parag, Naresh Nandram, Roshan Parasram, Zobida Khan-Mohammed, Lisa Edghill, Lisa Indar, Aisha Andrewin, Rhonda Sealey-Thomas, Pearl McMillan, Ayoola Oyinloye, Kenneth George, Irad Potter, John Lee, David Johnson, Shawn Charles, Narine Singh, Jacquiline Bisesor-McKenzie, Hazel Laws, Sharon Belmar-George, Simone Keizer-Beache, Sharra Greenaway-Duberry, Nadia Ashwood, Jerome E. Foster, Karla Georges, Rahul Naidu, Marsha Ivey, Stanley Giddings, Rajini Haraksingh, Adesh Ramsubhag, Jayaraj Jayaraman, Chinnaraja Chinnadurai, Christopher Oura, Oliver G. Pybus, Joy St. John, Gabriel Gonzalez-Escobar, Nuno R. Faria, Christine V. F. Carrington

**Affiliations:** 1 Department of Preclinical Sciences, Faculty of Medical Sciences, The University of the West Indies, St. Augustine, Republic of Trinidad and Tobago; 2 MRC Centre for Global Infectious Disease Analysis, Department for Infectious Disease Epidemiology, Imperial College London, London, United Kingdom; 3 Institute of Evolutionary Biology, University of Edinburgh, Edinburgh, United Kingdom; 4 Department of Zoology, University of Oxford, Oxford, United Kingdom; 5 Department of Pathobiology and Population Sciences, The Royal Veterinary College, London, United Kingdom; 6 Caribbean Public Health Agency (CARPHA), Headquartered in Port of Spain, Republic of Trinidad and Tobago; 7 Ministry of Health, Port of Spain, Republic of Trinidad and Tobago; 8 Ministry of Health, The Valley, Anguilla; 9 Ministry of Health, St. John’s, Antigua and Barbuda; 10 Ministry of Health, Nassau, Bahamas; 11 Ministry of Health, Hamilton, Bermuda; 12 Ministry of Health and Wellness, Bridgetown, Barbados; 13 Ministry of Health and Social Development, Road Town, Tortola, British Virgin Islands; 14 Ministry of Health and Wellness, George Town, Grand Cayman, Cayman Islands; 15 Ministry of Health, Wellness and New Health Investment, Roseau, Dominica; 16 Ministry of Health, St. Georges, Grenada; 17 Ministry of Health, Georgetown, Guyana; 18 Ministry of Health and Wellness, Kingston, Jamaica; 19 Ministry of Health, Basseterre, Saint Kitts and Nevis; 20 Ministry of Health and Wellness, Castries, Saint Lucia; 21 Ministry of Health, Wellness and the Environment, Kingstown, Saint Vincent and the Grenadines; 22 Ministry of Health and Social Services, Brades, Montserrat; 23 Ministry of Health, Agriculture, Sports and Human Services, Grand Turk, Turks and Caicos Islands; 24 School of Veterinary Medicine, Faculty of Medical Sciences, The University of the West Indies, St. Augustine, Republic of Trinidad and Tobago; 25 School of Dentistry, Faculty of Medical Sciences, The University of the West Indies, St. Augustine, Republic of Trinidad and Tobago; 26 Department of Clinical Medical Sciences, Faculty of Medical Sciences, The University of the West Indies, St. Augustine, Republic of Trinidad and Tobago; 27 Department of Life Sciences, Faculty of Sciences of Technology, The University of the West Indies, St. Augustine, Republic of Trinidad and Tobago; 28 Departamento de Moléstias Infecciosas e Parasitárias e Instituto de Medicina Tropical da Faculdade de Medicina, Universidade de São Paulo, São Paulo, Brazil; African Society of Laboratory Medicine, ETHIOPIA

## Abstract

The COVID-19 pandemic highlighted the importance of global genomic surveillance to monitor the emergence and spread of SARS-CoV-2 variants and inform public health decision-making. Until December 2020 there was minimal capacity for viral genomic surveillance in most Caribbean countries. To overcome this constraint, the COVID-19: Infectious disease Molecular epidemiology for PAthogen Control & Tracking (COVID-19 IMPACT) project was implemented to establish rapid SARS-CoV-2 whole genome nanopore sequencing at The University of the West Indies (UWI) in Trinidad and Tobago (T&T) and provide needed SARS-CoV-2 sequencing services for T&T and other Caribbean Public Health Agency Member States (CMS). Using the Oxford Nanopore Technologies MinION sequencing platform and ARTIC network sequencing protocols and bioinformatics pipeline, a total of 3610 SARS-CoV-2 positive RNA samples, received from 17 CMS, were sequenced *in-situ* during the period December 5^th^ 2020 to December 31^st^ 2021. Ninety-one Pango lineages, including those of five variants of concern (VOC), were identified. Genetic analysis revealed at least 260 introductions to the CMS from other global regions. For each of the 17 CMS, the percentage of reported COVID-19 cases sequenced by the COVID-19 IMPACT laboratory ranged from 0·02% to 3·80% (median = 1·12%). Sequences submitted to GISAID by our study represented 73·3% of all SARS-CoV-2 sequences from the 17 CMS available on the database up to December 31^st^ 2021. Increased staffing, process and infrastructural improvement over the course of the project helped reduce turnaround times for reporting to originating institutions and sequence uploads to GISAID. Insights from our genomic surveillance network in the Caribbean region directly influenced non-pharmaceutical countermeasures in the CMS countries. However, limited availability of associated surveillance and clinical data made it challenging to contextualise the observed SARS-CoV-2 diversity and evolution, highlighting the need for development of infrastructure for collecting and integrating genomic sequencing data and sample-associated metadata.

## Introduction

Pathogen genomic sequencing and related analytical approaches have proved to be invaluable tools for healthcare providers and public health decision-makers during the COVID-19 pandemic [1]. From the initial rapid sequencing and sharing of the first SARS-CoV-2 genome sequences [2, 3] to the subsequent global SARS-CoV-2 sequencing effort [4–6], combining genomic surveillance with traditional epidemiological methods has assisted in understanding the virus’ evolution and epidemic behaviour, and in guiding and evaluating clinical and public health interventions, including the development of diagnostic tools, therapeutics and vaccines.

While the pandemic brought the value of genomic surveillance to the forefront, it also highlighted countries and regions lacking the required infrastructure and capacity [7]. In the Caribbean, where SARS-CoV-2 was first detected in March 2020 and slowed by restrictive non-pharmaceutical interventions (NPIs), there was little to no capacity for viral whole genome sequencing (WGS) or viral genomic surveillance. However, The University of the West Indies (UWI) in Trinidad and Tobago (T&T) had significant prior expertise in pathogen genomics [8–10], and sought to contribute this skillset to enhance the public health response. As a result, in late 2020, the COVID-19 Infectious disease Molecular epidemiology for PAthogen Control & Tracking (COVID-19 IMPACT) project was initiated. This project aimed to establish capacity for rapid SARS-CoV-2 WGS in T&T so that viral genomics and related molecular epidemiological approaches could be incorporated into the mitigation and control efforts of the T&T Ministry of Health (T&T MoH) and other Caribbean Public Health Agency (CARPHA) Member States.

Specifically, COVID-19 IMPACT aimed to implement Oxford Nanopore Technology (ONT) MinION sequencing and ARTIC network open-source protocols and bioinformatic pipelines to generate baseline data on SARS-CoV-2 lineages circulating in the Caribbean. In doing this, the project sought to address questions relating to SARS-CoV-2 evolution and transmission within the region; and to respond to questions from local and regional public health bodies relating to COVID-19 control. For example, regarding the relative contribution to COVID-19 incidence of local transmission versus imported cases, whether clusters of cases were linked or the result of distinct, independent chains of transmission, or risks associated with different modes of importation.

COVID-19 IMPACT aimed to process 800 samples over two years and generated its first SARS-CoV-2 whole genome sequences in December 2020, two weeks prior to the first report of the emergence of the first variant of concern (VOC) [11]. The emergence of VOCs, with their attendant effects on transmission rates, disease severity and risk of re-infection/immune evasion, demonstrated the tangible public health impact of viral evolution and prompted a dramatic increase in demand for sequencing from Caribbean countries. Following consultation with the T&T MoH, T&T government diagnostic laboratories were advised to submit for sequencing samples from (i) all individuals entering T&T with a positive test result; (ii) local transmission (1:20 positive samples); (iii) known or suspected superspreading events (3 to 5 samples); (iv) all suspected reinfections and, (v) all persons locally, having entered or belonging to migrant populations, with a positive test result. Similar recommendations for sample selection were given to the other participating CARPHA Member States (CMS). However, due to resource limitations, for CMS submitting samples for sequencing through CARPHA, a maximum of 10 samples per country per month was implemented (although not always enforced).

During the project’s first year, the T&T MoH and regional health ministries in 16 other CMS (Anguilla, Antigua and Barbuda, Bahamas, Barbados, Bermuda, British Virgin Islands, Cayman Islands, Dominica, Grenada, Guyana, Jamaica, Montserrat, Saint Kitts and Nevis, Saint Lucia, Saint Vincent and the Grenadines, and Turks and Caicos Islands) relied on the COVID-19 IMPACT project for the generation, analysis and interpretation of genomic data on SARS-CoV-2 lineages circulating within the region. The effectiveness of this initiative was demonstrated by the rapid detection and reporting of VOCs in several CMS, which informed public health policy and decision-making for economic reopening, international travel restrictions and work policies. For example, in T&T, in order to slow the introduction of the gamma, delta and omicron VOCs to the general population, until community spread was confirmed, sequencing results were used to guide the enforcement of more stringent isolation criteria for individuals entering the country in whom new VOCs were detected (R. Parasram (T&T Chief Medical Officer), *personal communication*). Also, the sequencing information provided by the IMPACT laboratory informed Grenada’s decision to not reinstitute travel restrictions in March of 2021 as newly identified VOCs were identified in multiple countries at that time, demonstrating that further restrictions would be ineffective. (S. Charles (Grenada Chief Medical Officer), *personal communication*).

The COVID-19 IMPACT laboratory at the UWI is now a designated Pan American Health Organization (PAHO) reference sequencing laboratory (PAHO-RSL) and part of the COVID-19 Genomic Surveillance Regional Network. As at July 30, 2022 the project has processed over 4,800 clinical samples and contributed 3,999 sequences from the Caribbean sub-region to GISAID. More recently, it has provided training and technical support to laboratories in other CMS and institutions in building their own WGS capacity. Here, we describe the results of SARS-CoV-2 genomic surveillance efforts by the COVID-19 IMPACT project from December 5^th^ 2020 to December 31^st^ 2021, highlighting challenges and successes that can inform the necessary future development of pathogen genomic surveillance in the Caribbean and other less well-resourced regions.

## Methods

### SARS-CoV-2 sample receipt, data capture and sample processing

De-identified viral RNA extracted from samples positive for SARS-CoV-2 by real-time PCR (RT-PCR) were received by the COVID-19 IMPACT project laboratory at the UWI from (i) CARPHA, a regional public health body which serves 26 member states (https://carpha.org/Who-We-Are/Member-States) and (ii) the Trinidad Public Health Laboratory (TPHL) which, as the reference laboratory for the T&T MoH, receives samples for SARS-CoV-2 confirmatory testing from T&T’s five regional health authorities (RHAs) as well as from private laboratories offering SARS-CoV-2 RT-PCR testing. At CARPHA, RNA extraction was carried out using the Qiagen QiaAmp Viral RNA Mini kit (Qiagen, MD, USA) as per the manufacturer’s guidelines while the RT-PCR was performed on the Applied BioSystems QuantStudio Dx platforms using the Charité- Berlin (Berlin, Germany) protocol for the detection of the E-gene. This protocol utilises the AgPath- ID Ambion One-Step RT-PCR kit (ThermoFisher Scientific, MA, USA) and primer and probes ordered through Integrated DNA Technologies. At the TPHL, RNA extraction was carried out using the Tiangen TIANamp Virus DNA/RNA Kit (TIANGEN BIOTECH, Beijing, China) and RT-PCR carried out using the BGI 2019-nCoV: Real-Time Fluorescent RT-PCR kit (BGI, Cambridge, Massachusetts, USA), as well as the GeneXpert System and Xpert Xpress SARS-CoV-2 RT-PCR kit. In addition to prospectively collected samples, viral RNA extracted from SARS-CoV-2 RT-PCR positive samples collected prior to the implementation of the project during the first wave of COVID-19 in T&T (August to October 2020) were obtained retrospectively from TPHL.

A SARS-CoV-2 genome sequencing requisition form was developed for the project in consultation with the T&T MoH (see S1 Fig) to capture additional relevant sample data. This form was made available to all government laboratories in T&T referring samples to the TPHL. In addition to data gleaned from these requisition forms, for T&T samples, additional data were retrieved as far as possible from the TPHL electronic and paper records. Samples from other CMS submitted for sequencing via CARPHA [Anguilla (AIA), Antigua and Barbuda (ATG), Bahamas (BHS), Barbados (BRB), Bermuda (BMU), British Virgin Islands (VGB), Cayman Islands (CYM), Dominica (DMA), Grenada (GRD), Guyana (GUY), Jamaica (JAM), Montserrat (MSR), Saint Kitts and Nevis (KNA), Saint Lucia (LCA), Saint Vincent and the Grenadines (VCT), and Turks and Caicos Islands (TCA)] were received with information on country of origin, date of sample collection and cycle threshold (Ct) value. Samples were received in insulated coolers on ice packs and stored at -80°C until sequencing.

### SARS-CoV-2 whole genome sequencing

DNA libraries were prepared from viral RNA extracts using the ARTIC Network nCoV-2019 version 3 LoCost sequencing protocol and nCoV-2019 primer panel for cDNA amplification (https://dx.doi.org/10.17504/protocols.io.bbmuik6w), with version 3 used prior to 28^th^ October 2021 and version 4 thereafter. The libraries were sequenced using a MinION sequencing device until October 2021 after which sequencing was carried out using the GridION device (ONT, UK). Consensus sequences were generated using the ARTIC Network CoV-2019 novel coronavirus bioinformatics protocol, version 1.1.0. Reads passing quality control in MinKNOW were filtered using the ARTIC guppyplex tool (version 5.0.16) to retain only reads 400–700bp long and then assembled into consensus sequences using nanopolish (version 1.2.1) [https://artic.network/ncov-2019/ncov2019-bioinformatics-sop.html].

### Pango lineage assignment of SARS-CoV-2 consensus sequences and reporting to sending institutions

Consensus sequences were assigned to Pango lineages using the pangolin COVID-19 lineage web application (https://pangolin.cog-uk.io/) for sequences generated up until October 21^st^ 2021 and using the most recent version of the Pangolin COVID-19 command-line tool [12, 13] (version 3.1.14 to 3.1.17) thereafter. CoV-GLUE (http://cov-glue.cvr.gla.ac.uk/#/home), UShER [14] and Nextclade (https://clades.nextstrain.org/) were used for exploratory analysis prior to reporting results to the originating institutions, for example to inspect mutations, resolve ambiguities, and check for long branches. Samples that failed quality control or for which genome coverage was too low to allow Pango lineage assignment were reported as failed tests. WHO labels and variant categories of Variant of Concern (VOC), Variant of Interest (VOI) or Variant Under Monitoring (VUM) were assigned to sequences based on information on the WHO website (https://www.who.int/en/activities/tracking-SARS-CoV-2-variants/) at the time of reporting.

### Sequence data sharing

Consensus sequences with sufficient genome coverage to be assigned to a Pango lineage, as well as first time and early reports of VOCs (irrespective of the percentage coverage of the SARS-CoV-2 genome obtained), were uploaded to GISAID. In the case of first-time reports of VOCs, upload was delayed until the relevant country had reported publicly the detection of a VOC.

### Sample data analysis

IBM SPSS 24 (IBM Corp. Released 2016. IBM SPSS Statistics for Windows, Version 24.0. Armonk, NY: IBM Corp) was used to process any available demographic and clinical data associated with sequenced samples.

### Estimation of sequencing effort

The number of daily new SARS-CoV-2 cases for each of the 17 countries was accessed from the Johns Hopkins University Center for Systems Science and Engineering (JHU CSSE) COVID-19 Data Repository (https://github.com/CSSEGISandData/COVID-19) using scripts from the subsampler pipeline (https://github.com/andersonbrito/subsampler). The daily case data were subsequently grouped by epidemiological week (EW). The total population for each of the 17 countries was obtained from Worldometer (https://www.worldometers.info/). For each country, the percentage of genomes sequenced by the COVID-19 IMPACT project laboratory for each EW was calculated by dividing the number of genomes from that EW that were sequenced by the total number of cases reported for that week. For EWs during which there were no reported cases but for which samples were sequenced by the COVID-19 IMPACT project laboratory, the percentage of genomes sequenced was given as 100%.

### Phylogenetic analysis of SARS-CoV-2 sequences

A comprehensive reference data set was prepared using publicly available data from the GISAID EpiCoV database [15]. Briefly, metadata from all available sequences on the EpiCoV database, including location and collection date, was downloaded using the GISAID Audacity application table. This table was screened with a custom R script (S1 File), that returned a list of countries with available data for each EW. For each country and EW, a single GISAID epicode was randomly sampled. The complete set of epicodes was used to download one sequence per country per week from December 2019 to 5 January 2022 (n = 10,005; S2 File), which were then added to the novel sequences generated here. After discarding 5’ and 3’ untranslated regions, the combined data set consisted of 12,724 sequences with 29,409 base pairs, which were then mapped to the SARS-CoV-2 reference genome (NCBI accession: NC_045512) with mini-map2 [16].

Next, maximum likelihood phylogenies were estimated with IQ-Tree v2.0.6 [16] under a GTR+F+I+G4 nucleotide substitution model [17, 18] to reconstruct the evolutionary relationships of SARS-CoV-2 sequences. Temporal signal of the dataset was assessed using TempEST v1.5.3 [19] and molecular clock outliers were removed. We then used a newly-implemented approach to approximate the posterior distribution of the time-calibrated phylogenetic trees that are compatible with the final maximum likelihood tree from above. The complete dataset was analysed in BEAST v1.10.5 [20] using BEAST 1.10.5 pre-Thorney (https://github.com/beast-dev/beast-mcmc/releases/tag/v1.10.5pre_thorney), as previously described [21]. A skygrid tree prior [22] was used with monthly grid points and a cutoff of 2.18 years from the most recent sample date. Tracer v1.7.2 [23] was used to verify mixing and convergence of parameters (effective sample size > 200) and LogCombiner [20] was used to combine logs and resample empirical dated trees at lower frequency. A total of 20 independent MCMC chains were run for 100 million generations, sampling parameters and trees every 10,000 steps. After removing 10% of each chain as burn-in, trees were subsequently combined and resampled at lower frequency to generate a representative set of 1,000 empirical time-calibrated trees.

To assess the dynamics of virus lineage introductions into the Caribbean region, we performed a discrete trait analysis with an asymmetric two-state trait model (Caribbean, or from other locations) [24]. This analysis was performed on a sample of 1,000 empirical trees from the posterior distribution, as previously described [25]. A robust counting method was used to map all transitions among states estimated along the branches of phylogenetic trees [26]. For this analysis, two MCMC chains were run for 500,000 steps sampling parameters and trees every 1,000th step. A georeferenced maximum clade credibility tree was generated using TreeAnnotator v.1.10.5 [27].

### Ethical approval

The project protocols were approved by the UWI Campus Research Ethics Committee (CREC-SA.0246/06/2020) and the T&T MoH Ethics Committee (He: 3/13/441 Vol. II).

## Results

### Sample receipt, metadata capture and processing

**Fig 1:**
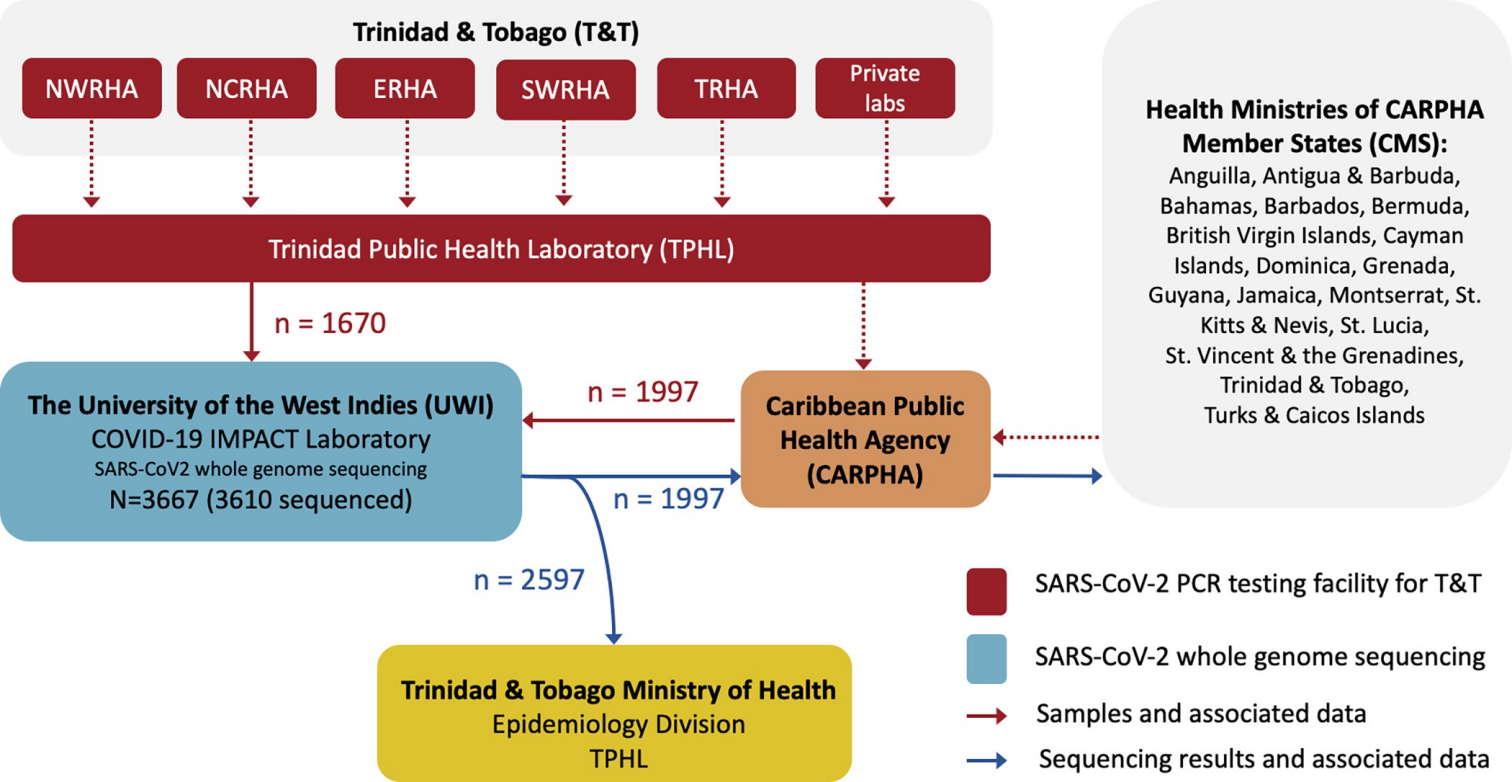
Movement of SARS-CoV-2 positive samples, data and sequencing results (Dec 5^th^ 2020 to Dec 31^st^ 2021). From December 5^th^ 2020 to December 31^st^ 2021, a total of 3667 viral RNA extracts from SARS-CoV-2 positive samples were submitted to the UWI COVID-19 IMPACT laboratory for sequencing. Of those 1670 were received, via TPHL, from T&T’s five regional health authorities (NWRHA, NCRHA, ERHA, SWRHA, and TRHA) as well as from private laboratories within T&T. The remaining 1997 samples were received from CARPHA, from seventeen CARPHA Member States. Sequencing results for samples received from CARPHA were reported back to CARPHA. For the 2597 samples from T&T (regardless of whether received via TPHL or CARPHA), results were reported to the Chief Medical Officer at the T&T Ministry of Health in a weekly cumulative report, except for first time reports of VOCs and VOIs which were reported to the Chief Medical Officer immediately upon detection until local transmission was confirmed. Results for samples received from other CARPHA Member States were reported to CARPHA as soon as they were obtained and relayed to the respective member state by CARPHA. Samples were submitted for sequencing with accompanying sample data. Broken red lines indicate routes where the COVID-19 IMPACT laboratory had no control over the communication of metadata. TPHL = Trinidad Public Health Laboratory. NWRHA = North-west Regional Health Authority. NCRHA = North-central Regional Health Authority. ERHA = East Regional Health Authority. SWRHA = South-west Regional Health Authority. TRHA = Tobago Regional Health Authority. TPHL = Trinidad Public Health Laboratory. Caribbean Public Health Agency = CARPHA. VOC = Variant of Concern. VOI = Variant of Interest.

From December 5^th^ 2020 to December 31^st^ 2021, a total of 3,667 RNA extracts from SARS-CoV-2 RT-PCR positive samples from 17 CMS were received by the COVID-19 IMPACT laboratory for virus WGS in order to screen for VOCs/VOIs. Fig 1 summarises the movement of samples, associated data and sequencing results from the contributing institutions. Samples received via TPHL (n = 1,670) originated from government diagnostic testing facilities in all five of T&T’s regional health authorities (RHAs) and at least three private laboratories. Samples received via CARPHA (n = 1,997) were from Anguilla, Antigua and Barbuda, The Bahamas, Barbados, Bermuda, The British Virgin Islands, The Cayman Islands, Dominica, Grenada, Guyana, Jamaica, Montserrat, Saint Kitts and Nevis, Saint Lucia, Saint Vincent and the Grenadines, and The Turks and Caicos Islands, as well as from T&T. Of all the samples received, 3,610 were sequenced as at December 31^st^ 2021.

Samples submitted via TPHL were usually accompanied by the project sequencing requisition form (S1 Fig), but forms were often incomplete and typically limited to information on date of sample collection, RT-PCR cycle threshold (Ct) or cycle number (CN) value, and originating RHA. Table 1 summarises the data that accompanied the 3667 RNA samples upon receipt by the UWI laboratory or that were recovered subsequently from institutional records (S3 File). Overall, most samples were received with an exact date of sample collection, i.e., year-month-date (96·9%, n = 3554) and either Ct or CN value (94·5%, n = 3,464). Of the latter, 424 (all from TPHL) were CN values, reflecting the use of the Abbott Real-Time SARS-CoV-2 Assay within the T&T RHAs. As shown in Table 1, the availability of the other metadata categories was limited and infrequently recovered.

**Table 1:** Sample metadata retrieved from SARS-CoV-2 sequencing requisition forms and sending institution records for samples received during the period 5^th^ Dec 2020 to 31^st^ Dec 2021.

	Samples from CARPHA (n = 1997)		Samples from TPHL (n = 1670)	
**Data Category**	**n**	**%**	**n**	**%**
Date of sample collection	1970	98·6	1584	94·9
Ct or CN value	1987	99·5	1477	88·4
Country of Origin	1984	99·3	1670	100
Age	29	1·5	83	5·0
Sex	145	7·3	107	6.4
Town/Country	243	12·2	103	6·2
Travel History	9	0·5	1	0·1
Date of onset of symptoms	2	0·1	9	0·5
Sequencing category*	0	0	11	0·7
Vaccination Status	0	0	4	0·2
Symptoms	0	0	116	6·9
T&T Regional Health Authority	139	7·0	509	30·5

* Sequencing category based on one of six indications for sequencing provided on the SARS-CoV-2 genome sequencing requisition form developed for the project in consultation with the T&T MoH, i.e. (A) surveillance of local transmission, (B) individual entering T&T, (C) known/suspected super spreading event, (D) suspected re-infection, (E) cluster investigation and (F) other. The requisition form developed also requested information on other relevant sample data: (i) Ct value (or CN value), (ii) dates of sample collection and extraction, (iii) date of symptom onset, and (iv) any additional comments from the submitting institution (e.g., clinical notes, information on linked samples). In some cases, sample data not included on requisition forms were subsequently recovered from institutional records. T&T = Trinidad and Tobago. MoH = Ministry of Health.

### Phylogenetic analysis and Pango lineage assignment

**Fig 2:**
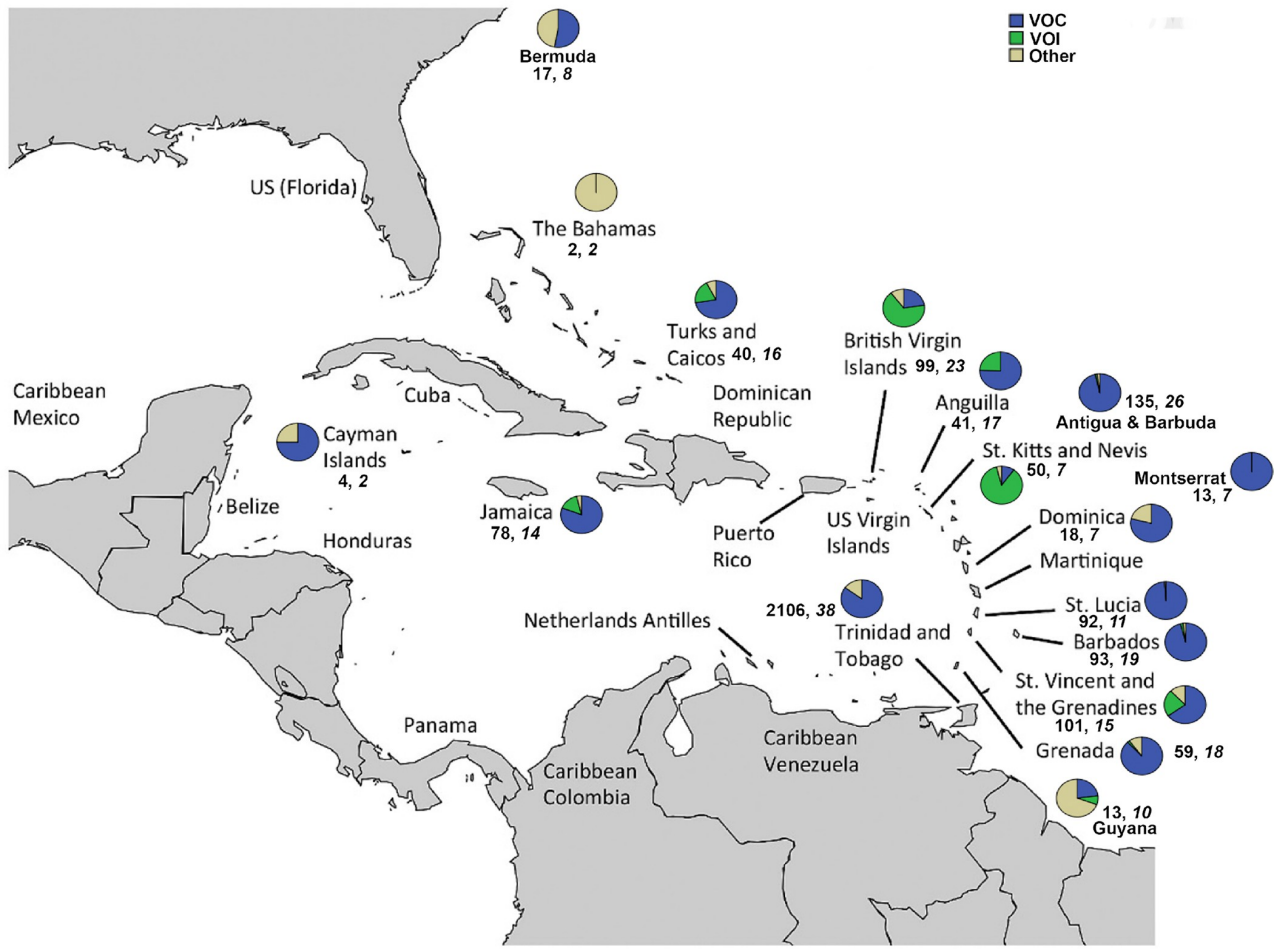
Variants of concern and variants of interest identified per country by the Covid-19 IMPACT sequencing laboratory as at December 31^st^ 2021. From December 5^th^ 2020 to December 31^st^ 2021 a total of 3610 samples were sequenced with sufficient coverage obtained for 2975 (82·4%) to enable assignment to a Pango lineage. For each of the 17 CARPHA CMS participating in the COVID-19 IMPACT project (i.e., Anguilla, Antigua and Barbuda, Bahamas, Barbados, Bermuda, British Virgin Islands, Cayman Islands, Dominica, Grenada, Guyana, Jamaica, Montserrat, Saint Kitts and Nevis, Saint Lucia, Saint Vincent and the Grenadines, Turks and Caicos Islands, and T&T) the percentages of VOCs (Alpha, Beta, Gamma, Delta, and Omicron) and VOIs (Epsilon, Iota, Lambda, and Mu) identified are indicated in a pie chart. All other lineages identified are grouped together and indicated as “Other”. The numbers below the pie charts indicate respectively the number of sequences and lineages identified in each country, as at December 31, 2021. CARPHA = Caribbean Public Health Agency. CMS = CARPHA Member States. VOC = Variants of Concern. VOI = Variants of Interest. Map base layer modified from Serafy et al. 2015 [28] (License CC by 4.0; available at https://doi.org/10.1371/journal.pone.0142022.g001).

**Fig 3:**
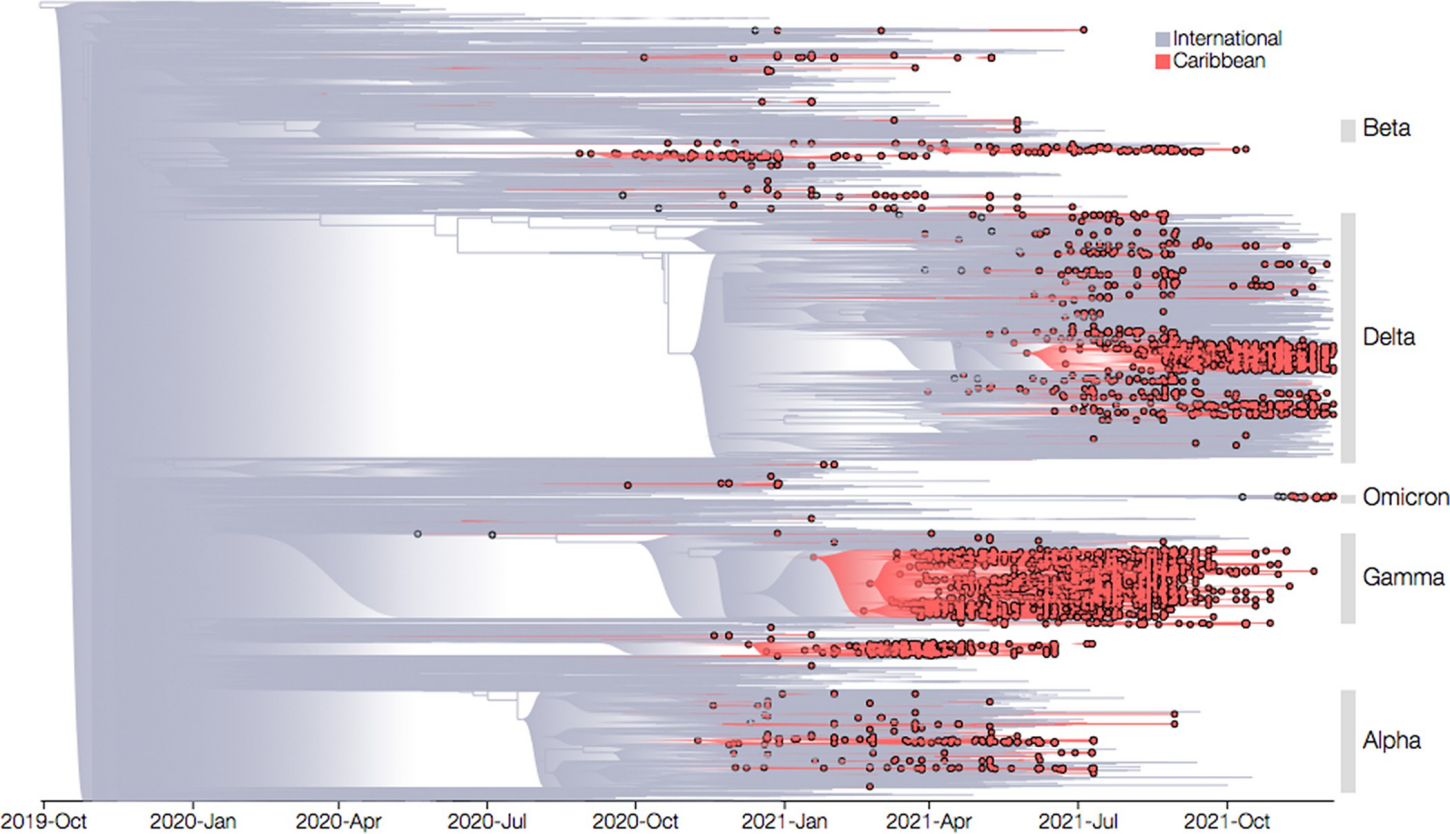
Bayesian timescaled phylogenetic reconstruction. SARS-CoV-2 sequences with over ≥75% genome coverage generated by the COVID-19 IMPACT laboratory between December 5th 2020 to December 31st 2021 are indicated with circles filled in red (n = 2716). Those sequences comprising the background dataset are grouped as either sequences from the Caribbean (specifically CARPHA Member States, indicated by red branches and open circles) or sequences from international countries (i.e. non-CARPHA Member States, indicated by grey branches). VOCs identified are highlighted. CARPHA = Caribbean Public Health Agency. VOC = Variant of Concern.

Sufficient genome coverage to enable assignment to a Pango lineage was obtained for 2,975 [82·4% of all (3,610) samples sequenced]. Overall, 91 lineages were identified, including lineages corresponding to all VOCs (i.e., Alpha, Beta, Gamma, Delta, and Omicron) and VOIs (Epsilon, Iota, Lambda, and Mu). Fig 2 shows the proportion of VOCs and VOIs detected in each CMS. The frequencies of the individual lineages identified in each of the 17 CMS are shown in S1 Table, and the proportion of Pango lineages detected in each country are shown in S2 Fig. At least one VOC was identified in each CMS, except for The Bahamas. During the period under consideration, the Delta VOC was the most frequently detected among samples sequenced in all CMS, except T&T and St. Vincent and the Grenadines, where the Gamma VOC was the most commonly detected. The Alpha VOC was the most commonly detected lineage in The Cayman Islands while VOIs Mu and Lambda were the most commonly detected lineage in The British Virgin Islands, and St. Kitts and Nevis respectively. A phylogenetic tree of all sequences generated by the COVID-19 IMPACT laboratory with ≥75% genome coverage is shown in Fig 3.

Phylogenetic analysis revealed the new genome sequences clustered along diverse lineages of SARS-CoV-2, most prominently with VOCs, confirming genome sequence designations obtained using Pangolin. After removal of 59 molecular clock outliers, a strong temporal signal was identified in the dataset (R^2^ = 0·86; Slope = 1·2 x10^-3^). Hence, a Bayesian timescale phylogenetic reconstruction was performed, which resulted in an estimated median evolutionary rate of 8·39 x 10^−4^ substitutions per site per year [95% (highest posterior density) HPD: 8·28 x 10^−4^–8·51 x 10^−4^], in line with previous estimates [29]. Our analysis indicates the time of the most recent common ancestor (tMRCA) for the whole dataset was late 2019 (95% highest posterior density: 23rd August– 29th October) (Fig 3). The Markov jumps method reconstructed a total of 260 (95% HPD: 249–271) virus lineage introductions into the Caribbean region, while only 22 (95% HPD: 15–29) exports have been identified.

The majority of samples sequenced by the COVID-19 IMPACT sequencing laboratory was from Trinidad and Tobago (n = 2597, 71·9%) where both the project laboratory and CARPHA headquarters are located. The numbers and proportions of samples received from the remaining 16 CMS were as follows: Anguilla (47, 1·3%), Antigua and Barbuda (163, 4·5%), Bahamas (4, 0·1%), Barbados (113, 3·1%), Bermuda (18, 0·5%), British Virgin Islands (120, 3·3%), Cayman Islands (7, 0·2%), Dominica (20, 0·6%), Grenada (69, 1·9%), Guyana (15, 0·4%), Jamaica (95, 2·6%), Montserrat (15, 0·4%), Saint Kitts and Nevis (56, 1·6%), Saint Lucia (104, 2·9%), Saint Vincent and the Grenadines (107, 3·0%), and Turks and Caicos Islands (47, 1·3%).

**Fig 4:**
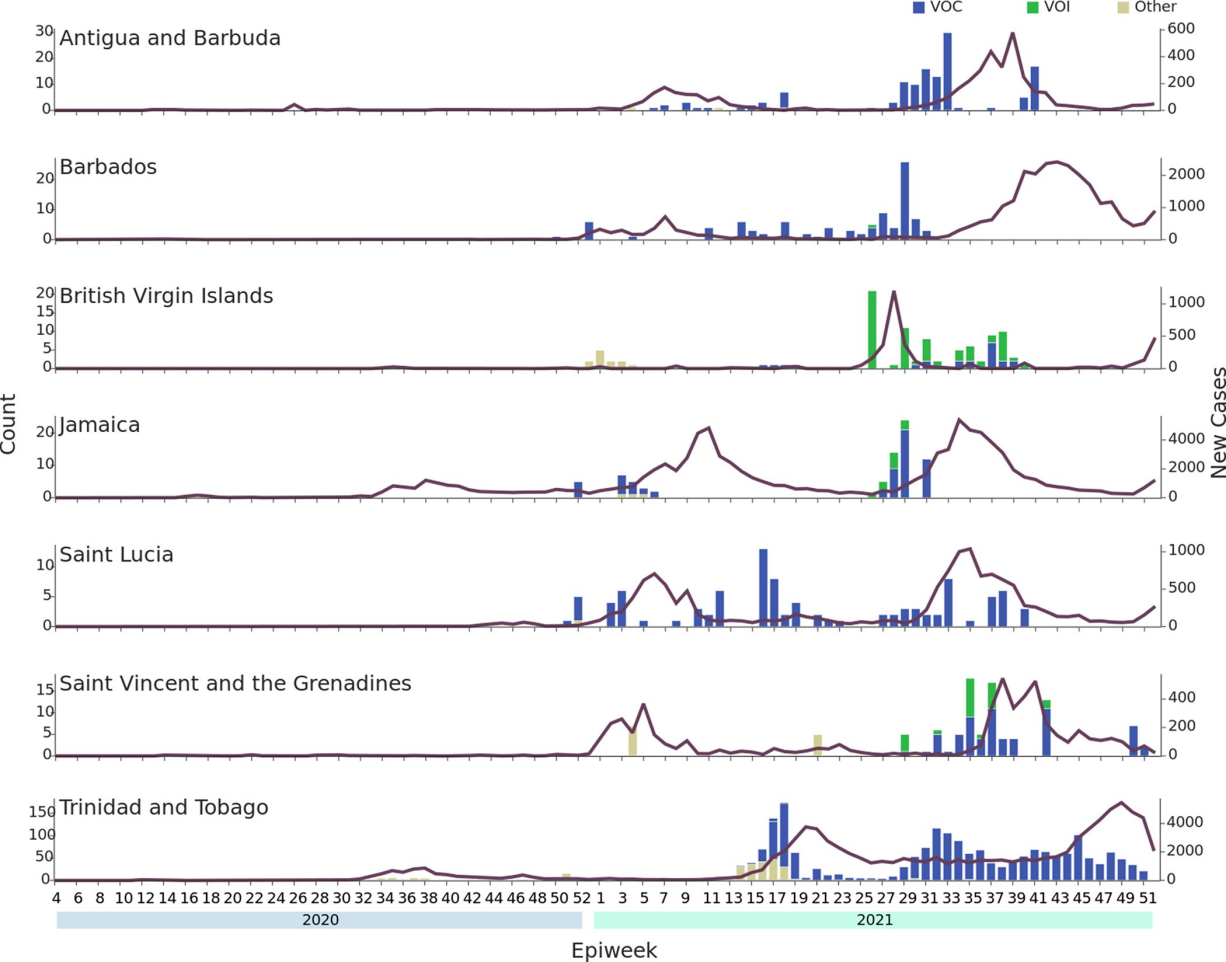
Frequency of variants of concern and interest identified by the COVID-19 IMPACT sequencing laboratory per epidemiological week for those CMS with at least 75 samples sequenced. Each histogram shows the number of VOCs (indicated in blue) and VOIs (indicated in green) per week in each CMS. All other lineages are grouped together as “Other” (yellow). Unsuccessfully sequenced samples are not represented as sufficient genome coverage was not obtained to be able to assign a Pango lineage. The purple line represents the number of confirmed reported COVID-19 cases for the country per week based on data available from The Johns Hopkins University Center for Systems Science and Engineering COVID-19 Data Repository. CMS = CARPHA member states. VOC = Variants of Concern. VOI = Variants of Interest.

For those CMS for which at least 75 samples were sequenced (i.e. Antigua and Barbuda, Barbados, British Virgin Islands, Jamaica, Saint Lucia, Saint Vincent and the Grenadines, and T&T), the distribution of lineages detected over time is shown in Fig 4 along with the epidemiological curve for confirmed reported cases in the respective countries. Results indicate that for the most part VOCs and/or VOIs were present and circulating within the population before a surge in the number of COVID-19 cases was reported in the country. It was not possible to reliably estimate a specific date of introduction for the different VOCs and VOIs in each country using the sequence data available, but in most cases, genomic surveillance first detected a VOC/VOI in samples collected 1 to 2 months before a surge in reported COVID-19 cases was observed in the respective country.

### COVID-19 IMPACT project sequencing effort

**Fig 5:**
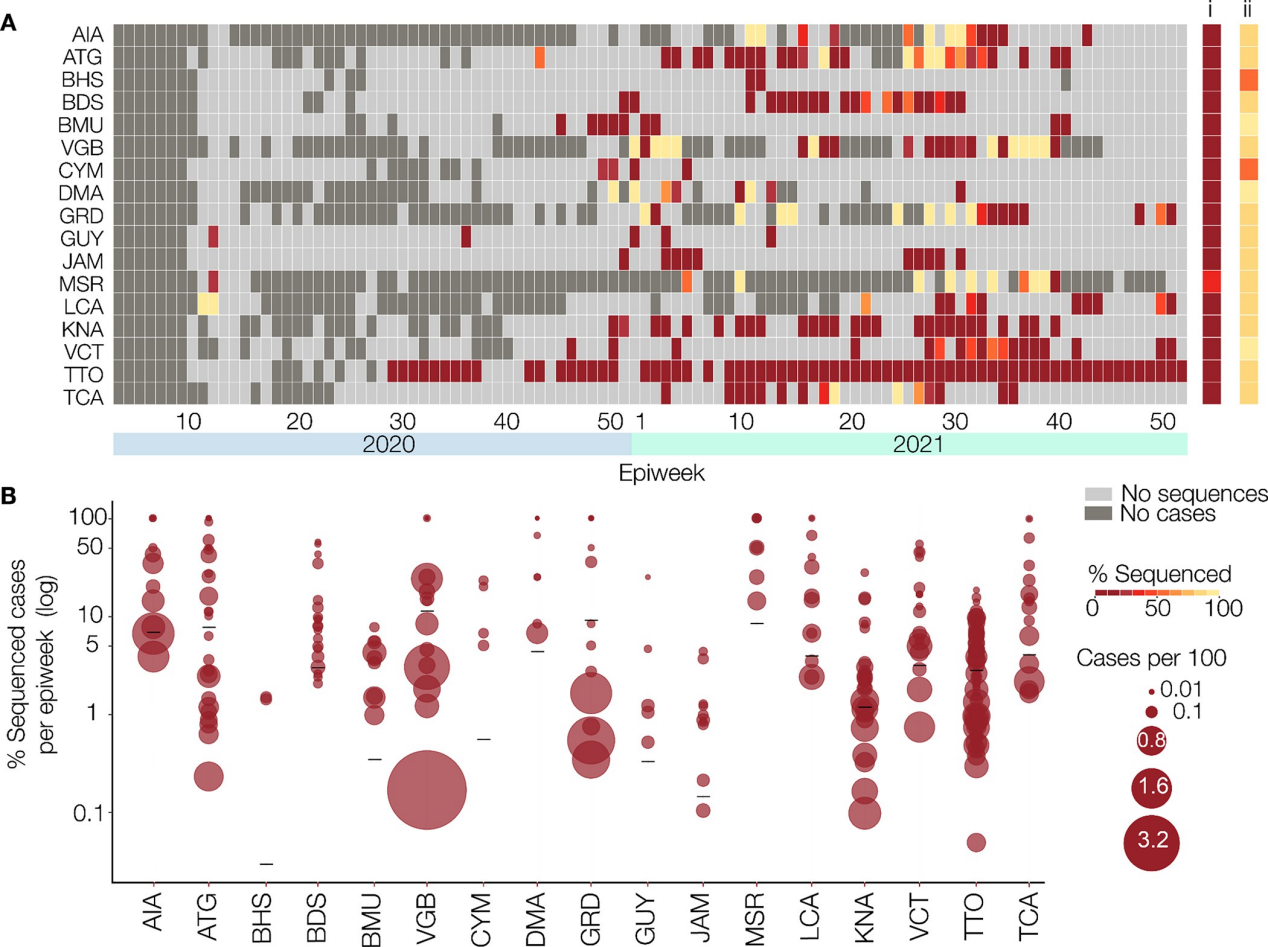
SARS-CoV-2 genomic surveillance by the COVID-19 IMPACT project. (A) Percentage of cases sequenced by the COVID-19 IMPACT laboratory per epidemiological week per CMS, between January 28^th^ 2020 and December 31^st^ 2021. For each CMS, the percentage of sequenced cases per epidemiological week is indicated by a coloured rectangle. Weeks during which there were no reported cases for that CMS are indicated in dark grey, and weeks in which there were cases reported for the CMS, but none were sequenced by the COVID-19 IMPACT laboratory are indicated in light grey. The overall percentage of cases sequenced by the COVID-19 IMPACT laboratory across the entire period is indicated for each CMS in column i. The percentage of samples received by the COVID-19 IMPACT laboratory that were successfully sequenced across the entire period is indicated for each CMS in column ii. The number of reported cases for each CMS is based on data available from The Johns Hopkins University Center for Systems Science and Engineering COVID-19 Data Repository. (B) Proportion of reported cases sequenced per CMS, between January 28^th^ 2020 and December 31^st^ 2021. Each circle indicates an epidemiological week during which at least one reported case for the respective CMS was sequenced and represents the percentage of sequenced cases for that week. Weeks where there were cases reported for the CMS but none were sequenced are not shown. The diameter of each circle represents the incidence (cases per 100/habitants) in that week for the respective CMS. The overall percentage of cases sequenced by the COVID-19 IMPACT laboratory for each CMS across the entire period is indicated by a black line. The number of reported cases for each CMS is based on data available from The Johns Hopkins University Center for Systems Science and Engineering COVID-19 Data Repository. CMS = CARPHA Member States. AIA = Anguilla. ATG = Antigua and Barbuda. BHS = Bahamas. BRB = Barbados. BMU = Bermuda. VGB = British Virgin Islands. CYM = Cayman Islands. DMA = Dominica. GRD = Grenada. GUY = Guyana. JAM = Jamaica. MSR = Montserrat. KNA = Saint Kitts and Nevis. LCA = Saint Lucia. VCT = Saint Vincent and the Grenadines. TTO = Trinidad and Tobago. TCA = Turks and Caicos Islands.

The percentage of reported COVID-19 cases that were sequenced for each EW per CMS is shown in Fig 5. For most CMS, in a majority of EWs there was no sequencing of samples collected (Fig 5A, light grey squares in heatmap). The exception was T&T where 67 of the 89 EWs that had cases were represented among samples sequenced, and Montserrat which was next highest with sequences from 11 out of the 21 weeks with confirmed reported cases. As illustrated in Fig 5B, Montserrat was the only CMS to sequence more than 5% of its confirmed COVID-19 cases with the percentage of cases sequenced per EW ranging from 14·3% to 100%. For all other CMS, the percentage of all cases sequenced ranged from 0·02 to 3·80%, with rates in each EW varying widely from <1% to 100%, the latter being in EWs with the fewest cases.

### Sequencing throughput and turnaround times

The exact dates of sample collection and receipt at the sequencing laboratory were available for 3,459 of the 3,610 samples sequenced. For T&T, the majority of samples (70·8%, n = 1,754) was received within one week of collection, while for samples originating outside of T&T, most took two or more weeks to arrive at the laboratory (S3A Fig). Most samples (82·2%, n = 2,903) were processed, sequenced and passed through the bioinformatics pipeline within two weeks of receipt by the sequencing laboratory (S3B Fig), and in a majority of cases (84·7%; n = 2,887) results were reported to the sending institution (T&T MoH or CARPHA) within one week of sequencing (S3C Fig).

Of all samples assigned to a Pango lineage, 2,864 (96·8%) were uploaded to GISAID. Overall, most (67·2%, n = 1,086) were uploaded more than three weeks after the sequences were generated and only 13·5% (n = 218) within one week (S3D Fig) with little difference between the rate at which T&T and non-T&T sequences were uploaded. However, as shown in S3E Fig, the proportion of samples uploaded within 3 weeks of sequencing increased over time.

**Fig 6:**
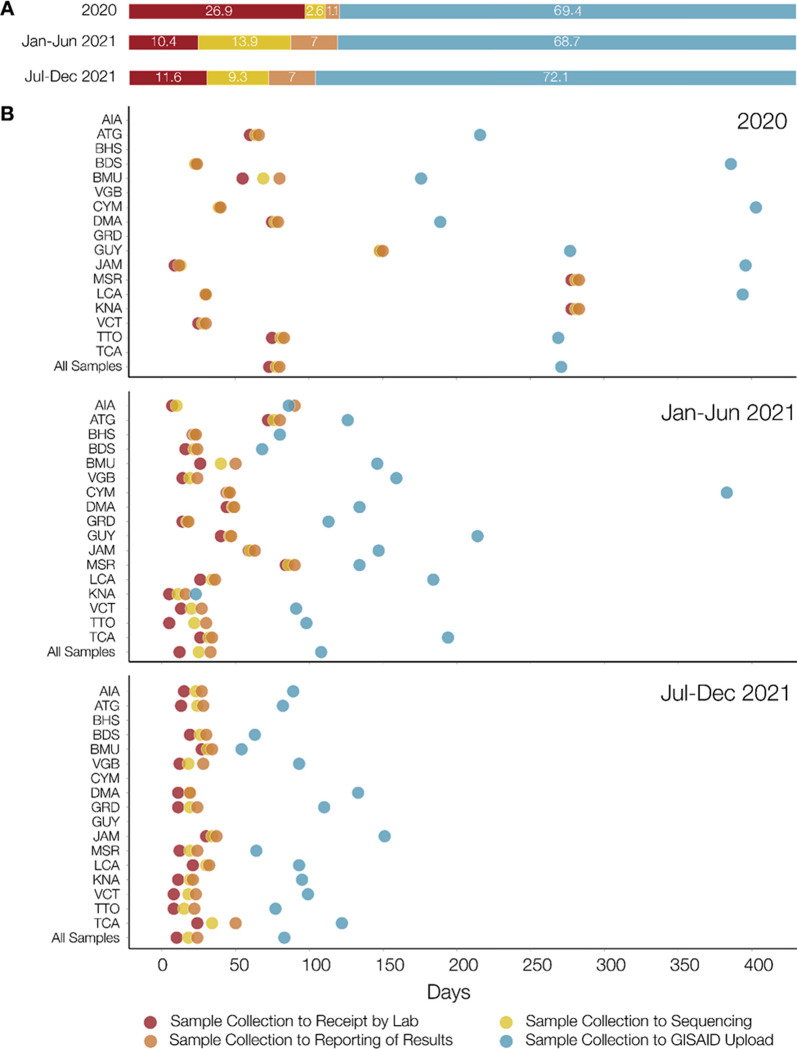
Turnaround times. (A) The average (across all 17 CMS) percentage time taken from (i) sample collection to receipt by the COVID-19 IMPACT laboratory (red), (ii) laboratory receipt to sequencing (yellow), (iii) sequencing to reporting of results to sending institution (i.e. Trinidad Public Health Laboratory (TPHL) or Caribbean Public Health Agency (CARPHA) (peach), and (iv) from reporting to sending institution to GISAID database upload (light blue) during three periods i.e. 2020, January 1st to June 30th 2021, and July 1st to December 31st 2021. (B) For each country, the average number of days from sample collection to: (i) receipt by the COVID-19 IMPACT laboratory (red circles); (ii) sequencing (yellow circles); (iii) reporting to sending institution (orange circles); and (iv) upload to the GISAID database (light blue circles). The times are shown for samples collected during three periods; 2020, January 1st to June 30th 2021 and July 1st to December 31st 2021. AIA = Anguilla. ATG = Antigua and Barbuda. BHS = Bahamas. BRB = Barbados. BMU = Bermuda. VGB = British Virgin Islands. CYM = Cayman Islands. DMA = Dominica. GRD = Grenada. GUY = Guyana. JAM = Jamaica. MSR = Montserrat. KNA = Saint Kitts and Nevis. LCA = Saint Lucia. VCT = Saint Vincent and the Grenadines. TTO = Trinidad and Tobago. TCA = Turks and Caicos Islands.

Fig 6 and S3 Fig show the distribution of the duration between sample collection and sequence upload to GISAID during different stages of the pandemic. Sequencing of samples once received, and reporting of results to CARPHA and/or T&T MoH, accounted for a minority of the time between sample collection from infected individuals and upload of the corresponding sequence to GISAID (Fig 6A), indicating that once samples were received at the laboratory, they were processed, sequenced and the results reported in a timely manner. However, there were significant delays between sample collection and receipt at the COVID-19 IMPACT laboratory, and between reporting of results and upload to GISAID, with the longest being for the latter and for samples collected in 2020. As shown in Fig 6B, by the second half of 2021, the times taken for each stage were much shorter than during 2020, especially for times from sample collection to receipt at the sequencing laboratory, and from sample collection to sequence upload to GISAID. Also, by the second half of 2021, times taken for each stage were comparable across the 17 CMS, except for the time to GISAID upload which, although dramatically reduced, still varied among CMS. Despite the reduction in the time taken for sequences to be uploaded to GISAID by the latter half of 2021, for most of the seventeen CMS, this upload process consistently accounted for the largest proportion of time in the system (Fig 6A).

### Effect of Ct value and sample age on percentage genome coverage

Out of the 3,610 samples sequenced, 3,004 had been received accompanied by the Ct value obtained during confirmatory RT-PCR. Sending institutions were asked to send samples with Ct values <28 and 82·5% (n = 2,979) of those samples sequenced met this criterion including 66·9% (n = 2,416) in the 10–25 range. However, while most of these yielded sequences with genome coverage over 75%, there were many samples with low Ct values that performed poorly and vice versa (S4A Fig). There was no difference in the pattern for T&T versus overseas (non-T&T) samples suggesting that RNA degradation during transit was not responsible for the poor coverage obtained from some samples with low Ct values. To test whether sample age was a factor, we plotted genome coverage against the time between sample collection and sequencing (S4B Fig). Results did not show a trend of genome coverage declining with increasing sample age.

## Discussion

The rapid evolution of SARS-CoV-2 and repeated emergence of lineages with altered epidemiological characteristics of public health significance (e.g., increased transmissibility, immune escape, disease severity), has emphasised the importance of comprehensive and sustained genomic surveillance and the need to enhance capacity in settings where this is currently limited. This is especially important in regions such as the Caribbean, where no such capacity previously existed. The COVID-19 IMPACT project was initiated at the UWI in T&T, in December 2020 in order to address this gap. The project was conceptualised and initially funded as a research project that would implement local capacity for virus whole genome sequencing and would sequence a maximum of 800 retrospective and prospective SARS-CoV-2 samples in order to establish baseline information of viral diversity and evolution in the region, and assist public health bodies with targeted investigations.

Successful implementation of the project in a relatively short time frame was greatly facilitated by (i) pre-existing local expertise in viral genomics and phylogenetics at the UWI, (ii) early commitment to the project by the T&T MoH and CARPHA, (ii) the UWI project laboratory’s prior collaborative research links with local and regional public health bodies (i.e. TPHL and CARPHA), and with international partners, (iv) CARPHA’s rapid engagement and receipt of commitment from other CMS, and (v) open access to global scientific expertise, relevant protocols and scientific publications (see Table 2). However, within two weeks of generating its first sequences, in line with WHO guidance [30], the project was called upon to use its sequencing capacity to support routine surveillance for VOCs in the Caribbean region on behalf of the T&T MoH and CARPHA. This change in focus, from research to *de facto* public health laboratory, meant a dramatic increase in samples submitted to the sequencing laboratory, pressure to minimise turnaround times and the need for rapid implementation of more formal sample handling and reporting procedures, initially with very limited human resources and infrastructure (S2 Table). With additional support from local and regional public health bodies facilitating acquisition of more equipment, reagents, consumables, and engagement of additional personnel, the project was largely able to adapt to the increased demands.

**Table 2:** Considerations in the implementation of the COVID-19 IMPACT genomic surveillance programme, its public health objectives and policy input.

Category	Comment
Available Capacity	Pre-existing expertise in virus genomics, phylogenetics and evolution and in related laboratory and bioinformatic techniques within the COVID-19 IMPACT sequencing laboratory facilitated rapid implementation of whole genome sequencing protocols and bioinformatics tools.
Diagnostic and sequencing capacity	Existence of sufficient laboratory infrastructure, equipment and human resources facilitated rapid integration of wet-laboratory sequencing protocols once a sequencing device was obtained.
Availability of sequencing resources	Low-cost MinION devices (requiring no capital expenditure) facilitated initial implementation of genome sequencing protocols, and ability to respond to requests from public health bodies while funding was sought for more expensive high-throughput devices.
Open-source sequence data analysis tools	Availability of open-source software (e.g., Pangolin, Nextstrain, MicroReact, UShER, CoV-GLUE) and open public access databases (GISAID, outbreak.info) simplified data sharing and analyses.
Key stakeholders	Early engagement of national- and regional-level policymakers and stakeholders (i.e. Chief Medical Officers, Ministry of Health, TPHL, CARPHA, PAHO/WHO) helped to maximise the public health benefit of SARS-CoV-2 sequencing initiative and facilitated rapid communication of timely results.
Funding and human resources	Institutional seed funding (UWI) and support from local (T&T MoH) and regional public health bodies (PAHO/WHO, CARPHA), and funding agencies (AHF Global Public Health Institute) helped to maintain sequencing flow and timely communication of results to stakeholders.
Collaborators / Access to external expertise	Pre-existing collaborative networks facilitated ease of access to expert advice in sequencing protocols and data analysis.
Capacity training	Access to related training prior to and during the pandemic [e.g., Virus Evolution and Molecular Epidemiology Workshops, CADDE Genomic Epidemiology Workshop (Brazil), ARTIC Network and CLIMB-BIG-DATA Joint Workshop on COVID-19 Data Analysis (Online), WHO-GISAID International Training Course in Influenza and SARS-CoV-2 Bioinformatics (Online)] helped in expanding sequencing capacity.

Within the first year, 3,610 samples from 17 CMS were sequenced, analysed, and the results returned to the CMS of origin to inform public health policy as necessary. A large diversity of SARS-CoV-2 lineages were detected, including VOCs and VOIs designated at the time and with at least 260 introductions originating from non-CMS. The estimated tMRCA of late 2019 based on the study data set is consistent with previous estimates for SARS-CoV-2 emergence [31–34]. However, this result should be interpreted with caution since our data set did not include the earliest SARS-CoV-2 genomes and therefore is not ideally suited for estimating the tMRCA for the pandemic as a whole.

It is important for genomic surveillance sampling to be appropriately targeted and sufficiently extensive to detect the early emergence and expansion of new lineages [35]. Vavrek et al. [36] estimated that 5% sampling of all SARS-CoV-2 positive cases would enable the detection of emerging strains with a prevalence of 0·1% to 1·0%, while Brito et al. [7] estimated that detection (with 95% probability) of a lineage circulating in a population at a weekly prevalence of 1% requires the sequencing of at least 300 SARS-CoV-2 genomes per week. For the COVID-19 IMPACT sequencing initiative, following consultation with the T&T MoH, government diagnostic laboratories were advised to submit for sequencing samples from all SARS-CoV-2 positive individuals entering T&T or belonging to migrant populations, 5% of local transmission, 3–5 samples from a superspreading event and all suspected reinfections. Similar recommendations for sample selection were given to the other 16 participating CMS. However, the project’s sequencing capacity was still limited by funding availability, and for CMS submitting through CARPHA, a maximum of 10 samples per country per month was implemented (although not always enforced).

Further, for the vast majority of samples received by the project laboratory, no information was provided to indicate to which, if any, of the targeted sequencing categories the samples belonged, making it difficult to determine the extent to which sampling criteria were being met. This is in part because the study did not anticipate the scale and direction in which it would develop, and (in line with the original objective of establishing a baseline of SARS-CoV-2 diversity in the Caribbean), only date of sample collection, geographic origin, sample type and Ct value were requested and agreed by CARPHA and the T&T MoH. The additional information required to confirm and contextualise the sequencing effort is available within the respective government public health institutions. However, retrieval remains a major challenge because the institutions involved have differing platforms for sample and data management, as well as different policies and arrangements for data sharing. CARPHA does not share data from CMS without their explicit permission, so access would necessarily require agreement from each of them. In the case of T&T, the MoH agreed to share these and other non-identifying metadata, but staffing limitations within the ministry severely hindered retrieval. Also, since the ethical approval granted by the T&T MoH specified that samples must be de-identified before being sent to UWI, UWI staff could not carry out the data retrieval themselves.

With the exception of Montserrat (which sequenced 32.6% of 46 cases reported during the study period), the percentage of reported COVID-19 cases sequenced by the COVID-19 IMPACT laboratory for each CMS between December 5^th^ 2020 to December 31^st^ 2021 varied from 0·02% to 3·80%, with a median of 1·12%. Based on data from the GISAID Submission Tracker, this is considerably lower than rates achieved by countries such as Denmark and the United Kingdom which, as of March 7^th^ 2022, had sequenced and shared approximately 14% and 12% of their cases respectively. For sixteen of the CMS, the rate achieved by the COVID-19 IMPACT laboratory does not represent the total genomic surveillance effort during the specified period, as those CMS intermittently submitted SARS-CoV-2 positive samples to extra-regional laboratories for sequencing. Also, having established in-house sequencing capacity in late 2021, CARPHA began offering sequencing services to its member states in December of 2021. Interestingly, while the overall median percentage of reported COVID-19 cases sequenced by the COVID-19 IMPACT laboratory during the time period in question was 1·12%, the lowest percentages of reported cases were sequenced during the latter half of 2021 (median 0.99 vs. a median of 1.14 for 2020 and a median of 1.63 for January to June 2021). This decrease may in part be explained by sequencing services being offered by CARPHA in late-2021 as well as some Caribbean countries having established in-house sequencing capacity also during late-2021. Nevertheless, overall, the COVID-19 IMPACT laboratory accounted for the majority (73·3%) of the SARS-CoV-2 sequences on GISAID for the 17 CMS up to December 31^st^ 2021, albeit with the percentage of sequences originating from the COVID-19 project varying widely among CMS. While it was not possible to determine a specific date of introduction for the different VOCs and VOIs in each country, in some cases the first detection of a VOC/VOI in a country via sequencing by the Project IMPACT laboratory was in samples collected up to 2 months before a reported surge in COVID-19 cases occurred in the respective country.

The total number of samples and the frequency at which samples were received for sequencing varied widely among the 17 CMS, with the least (n = 4) from the Cayman Islands, and most (n = 2597) from T&T. Only 7 countries had more than 75 sequences uploaded to GISAID and for three of these (Antigua and Barbuda, Jamaica and Barbados), few or no samples from epidemic wave peaks were sent to the COVID-19 IMPACT laboratory for sequencing. Several factors may have influenced this variability in total and weekly sequencing effort via the COVID-19 IMPACT project. For example, resource limitations necessitated the implementation of a limit on the number of samples per month per country. Recognizing that at any given time CMS were not equally affected, this limit was not routinely upheld. Some countries sent many more samples than the prescribed limit while others may have observed the limit even during peak periods. The very low number or absence of samples representing epidemic wave peaks may also reflect the fact that healthcare services were overwhelmed during these peaks and did not have the resources to dedicate effort to genomic surveillance. Alternatively, having confirmed the presence of a VOC (prior to an epidemic peak), countries may have deemed it unnecessary to continue sequencing. Finally, in the case of the Cayman Islands, in-country sequencing capacity was developed in early 2021 and they stopped sending samples to the project at that point.

The critical contributions of global genomic surveillance in elucidating and understanding the epidemiological characteristics of SARS-CoV-2 lineages and in informing public health responses are as much dependent on rapid and open sharing of genomic sequence data, primarily via GISAID [15, 37] as they are on continuous and comprehensive surveillance. From this project, the majority (~80%) of the genome sequences was uploaded to GISAID, although mostly (~60%) >4 weeks after reporting of the results. There was therefore mixed success in the rapid sharing of data. The delays in data upload were largely due to procedural rather than technical reasons since, for each time a VOC or VOI in a CMS was first detected, upload to GISAID was delayed to allow the country’s Ministry of Health to first inform their population, and there was initially no limit on the timeframe allowed. To facilitate more rapid upload, in June 2021 CARPHA advised member states that for the first report of VOC in a given CMS, sequences would be uploaded to GISAID one week after the member state was officially notified by CARPHA, and that all other sequence data would be uploaded as soon as possible after official notification. This change in policy accounts for the increase in the proportion of sequences uploaded to GISAID between 1 to 2 weeks after mid-November 2021 as shown in S3 Fig.

The project was initially funded through a UWI grant initiative that covered the sequencing of about 800 samples by a limited laboratory team (one part-time technician and one full-time postdoctoral researcher) who were responsible for all stages, from sample receipt to GISAID upload. Faster laboratory turnaround times and GISAID upload were facilitated when WHO/PAHO support added five members to the laboratory team in June 2021 and a sixth in September 2021. The sixth had the required expertise in bioinformatics and computer coding to automate the analysis, reporting and upload processes which contributed to an increase in the proportion of sequences uploaded within one week and the clearing of a backlog (represented in S3 Fig by the large number of sequences uploaded 3 weeks and more post reporting of results). As at 31 December 2021, the median lag time between sample collection and upload to GISAID by the COVID-19 IMPACT project was 56 days. This fits approximately in the middle of the distribution of median lag times between sample collection and GISAID submission globally, with the shortest being 16 days (United Kingdom) and the longest being 88 days (Canada) [7]. This emphasizes the importance of having access to bioinformatics and computer-coding expertise and / or training in addition to adequate manpower when establishing genomics surveillance capacity.

For sensitive surveillance, in addition to the intensity of sequencing, the quality of the sequences generated is important. To maximise the probability of obtaining enough genome coverage to enable lineage assignment, maximum Ct and CN values of 28 and 18 respectively were requested for samples sent for sequencing. A minority of samples was received with Ct and CN values over the stipulated threshold but these were nonetheless sequenced since they were flagged as priority samples from cases of particular public health interest. Less than 50% coverage was obtained for 210 samples with a Ct value of ⩽25 suggesting that the Ct value indicated for samples sent for sequencing were not representative of the viral RNA quantity in the samples received. This was observed for samples originating from T&T, as well as those originating from the other 16 CMS, suggesting that unsuccessful sequencing of samples was not the result of RNA degradation during the longer transit times from overseas CMS to the COVID-19 IMPACT laboratory compared to the T&T samples.

Moreover, the observed discrepancy between reported Ct value and sample quality highlights several difficulties in the sample requisition process in T&T. As illustrated in Fig 1, T&T samples from suspected COVID-19 cases were tested at both RHAs and private laboratories, which use different RNA extraction and testing methods, and different standardisation and quality control methods. SARS-CoV-2 positive samples are sent to the TPHL for confirmatory testing. At TPHL, RNA is re-extracted for this purpose and the re-extracted RNA is delivered to the COVID-19 IMPACT laboratory. However, the Ct and CN values provided to the COVID-19 IMPACT laboratory were those obtained during the initial diagnostic testing by the RHAs or private laboratories, and no information was provided on the methods used by those institutions. Furthermore, RNA samples, while usually received by the sequencing laboratory cold, were often unfrozen, further contributing to the discrepancy observed in the relationship between Ct/CN and percentage genome coverage.

Possible issues with the quality of RNA samples received by the COVID-19 IMPACT laboratory prompted the inclusion of a quality control step after cDNA amplification in the library preparation process. However, due to pressure to maintain high throughput and reduce turnaround times, this step was not routinely implemented for all samples. Given that the COVID-19 IMPACT laboratory has little control on RNA sample screening methods used at originating laboratories and on storage conditions during transport, this quality control step should ideally have been included for all samples. This again points to the importance of developing human and infrastructural resources and implementation of standard operating procedures across all institutional stakeholders in genomic surveillance.

Other issues affecting sequencing efficiency and consistency were beyond the control of the laboratory, emphasising the need for holistic capacity building of genomic surveillance systems. For example, delays in the sequencing of samples once received at the laboratory were primarily due to delays in the receipt of funding and in procurement of sequencing reagents and consumables. Even with the introduction of a sequencing specific inventory management system, the latter continues to be an ongoing issue for the COVID-19 IMPACT laboratory due to manufacturer and supplier shortages [38], and slow institutional procurement processes. Explanations for the latter included staffing limitations in the procurement offices, foreign exchange access restrictions and cumbersome institutional requirements for multiple levels of approval for all procurement.

To date, the COVID-19 IMPACT project laboratory, via GISAID, has made only virus genomic data, obtained from SARS-CoV-2 samples collected from the 17 participating CMS between 2020 to present, available. Associated clinical data and surveillance data were not made available to the laboratory for the majority of samples, and to date efforts to retrieve these data have proved difficult. In the absence of these key metadata it was not possible to discern how the virus’ evolution relates to epidemic behaviour and disease profile in the region. This lack of metadata was the major limitation on the COVID-19 IMPACT laboratory’s ability to function as part of a complete genomic surveillance system. For each country and the wider Caribbean region to progress with genomic surveillance, there is a need to develop a digital infrastructure that addresses the challenge of collecting and integrating both genomic sequencing data and sample-associated metadata produced across the individual countries.

When a new disease such as COVID-19 threatens, it is expected that public health bodies turn to academic research institutions such as the UWI for leading-edge advice, training and technical support. However, for an optimal response, it is critical that this support is rapidly converted into enhanced capacity and actions within the public health institutions, freeing up the academic institution to focus on other key activities in readiness to inform and support the next phase. During the course of the COVID-19 pandemic, the UWI laboratory was called upon to not only provide expert advice, training and technical support, but also to use and upgrade its research equipment, infrastructure and personnel to fulfil core public health laboratory services outside of its normal remit for an extended period. The resulting rapid expansion in equipment and laboratory throughput at the COVID-19 IMPACT laboratory was critical for the UWI to effectively partner with public health institutions to address the ongoing COVID-19 pandemic, can be applied to other pathogens and will be essential for future public health emergencies.

For example, the COVID-19 IMPACT laboratory was able to implement a sequencing protocol for yellow fever virus [39] and rabies virus in response to local outbreaks (Seetahal et al., manuscript in preparation), will apply current capacity to other pathogens and is able to offer guidance on database setup and management, training and implementation of sequencing protocols and reference sequencing services. However, a significant proportion of the university laboratory’s time was dedicated to activities that would better be carried out at the level of the national public health laboratories. Activity streams have since begun to be developed at this level, but the overall effects of the logistics involved during the project were limitations on ability to perform more in-depth, targeted and timely investigations that may have further enhanced the sub-region’s response. For optimal genomic surveillance impact, expansion at academic institutions needs to be matched by appropriate and corresponding infrastructure and human capacity development at the national public health laboratories. Where available, material and human resource support from international public health agencies should be sought in order to expedite knowledge transfer and enhancement of local laboratory capacity and thereby bolster the robustness and the sustainability of local public health responses. The COVID-19 IMPACT sequencing initiative propelled CMS out of the pathogen genomic surveillance starting blocks, but further action and additional resources are critically needed to enhance, disseminate, adapt and sustain existing genomic surveillance capacity, in order that Caribbean sub-region is better prepared for future infectious disease health emergencies and pandemics, rather than simply reactive to their arrival.

## Supporting information

S1 Fig Sequencing requisition form. (TIF)

S2 Fig Proportion of SARS-Cov-2 Pango lineages identified per country by the Covid-19 IMPACT sequencing laboratory as at December 31^st^ 2021. For each of the 17 CARPHA territories participating in Project IMPACT (Anguilla, Antigua and Barbuda, Bahamas, Barbados, Bermuda, British Virgin Islands, Cayman Islands, Dominica, Grenada, Guyana, Jamaica, Montserrat, Saint Kitts and Nevis, Saint Lucia, Saint Vincent and the Grenadines, Turks and Caicos Islands and T&T) the percentage of each lineage identified is indicated in a pie chart. The percentage of sequences from each country for which insufficient coverage was obtained to be able to assign a Pango lineage is not shown. From December 5^th^ 2020 to December 31^st^ 2021 a total of 3610 samples were sequenced. Sufficient coverage was obtained for 2975 (82.4% of all samples sequenced) to enable assignment to a Pango lineage. The numbers below the pie charts indicate respectively the number of sequences and lineages identified in each country, as at December 31, 2021. Ninety-one individual lineages were identified, including all 5 VOCs (Alpha, Beta, Gamma, Delta and Omicron) and two VOIs (Epsilon, Iota, Lambda, and Mu). Sub lineages for the VOCs and VOIs identified are grouped together using the WHO classifications. VOC = Variants of Concern. VOI = Variants of Interest. Map base layer modified from Serafy et al. 2015 [28] (License CC by 4.0; available at https://doi.org/10.1371/journal.pone.0142022.g001).
(TIF)

S3 Fig Turnaround times. In panels A-D samples originating from T&T are indicated in blue and samples originating from non-T&T CMS are indicated in pink. (A) Time taken for samples to be received at the COVID-19 IMPACT laboratory after collection. Date of sample collection and date of receipt at the sequencing laboratory were available for 3463 samples sent for sequencing. (B) Time taken for sequencing to be reported once samples have been received by the COVID-19 IMPACT laboratory. This includes time for storage upon receipt, sample processing and library preparation, sequencing and bioinformatics pipeline analysis. Regardless of the sending institution, results for all T&T samples (n = 2596) were reported to the T&T MoH while results for samples coming from CARPHA were reported to CARPHA (n = 1670). Where multiple reports were made (e.g. T&T samples received from CARPHA reported to both CARPHA and T&T MoH) the earliest report date was used. (C) Time taken for results to be reported once having been processed and sequenced. Results for all T&T samples (regardless of the sending institution; n = 2596) were reported to the T&T MoH while results for samples coming from CARPHA (n = 1670) were reported to CARPHA. Where multiple reports were made, the earliest report date was used. (D) Time from date sequenced to upload to the GISAID database. 2828 sequences were uploaded to GISAID as at December 31 2021 (E) Time taken for sequences to be uploaded to the GISAID database after sequencing per week, for samples originating from Trinidad and Tobago and non-Trinidad and Tobago countries. The time taken in days for sequences (n = 2864) to be uploaded to GISAID after generation is indicated for sequences generated from T&T samples (left) and non-T&T samples (right). On the x-axis the time of sample collection (epidemiological week midpoint) is indicated for the samples from which the sequences were derived. T&T = Trinidad and Tobago. T&T MoH = Trinidad and Tobago Ministry of Health. CARPHA = Caribbean Regional Health Agency.
(TIF)

S4 Fig Percentage Genome Coverage of SARS-CoV-2 Sequences retrieved versus (A) Ct Value and (B) Sample Age. Samples originating from Trinidad and Tobago (T&T) are indicated in blue while those from other CARPHA member states (CMS) (overseas) are indicated in green. The median of the frequency distribution for percentage of the SARS-CoV-2 genome retrieved (89%) is indicated by the red line and the maximum Ct value specified in the criteria for sequencing (Ct = 28) is indicated by the black line was requested for samples to be sent for sequencing. Sample age is the length of time from the date a sample was collected from a COVID-19 case to the date the sample was sequenced.
(TIF)

S1 Table Frequencies of the individual Pango lineages identified in each of the 17 Caribbean Public Health Agency member states. (DOCX)

S2 Table Key challenges encountered during the COVID-19 IMPACT project. (DOCX)

S1 File Custom R script. (R)

S2 File Details for sequences generated by this study and background dataset. (XLSX)

S3 File Associated meta data for RNA samples. (PDF)
