# Correction: Implementation of Genomic Surveillance of SARS-CoV-2 in the Caribbean: Lessons learned for sustainability in resource-limited settings

**DOI:** 10.1371/journal.pgph.0002393

**Published:** 2023-09-11

**Authors:** Nikita S. D. Sahadeo, Soren Nicholls, Filipe R. R. Moreira, Áine O’Toole, Vernie Ramkissoon, Charles Whittaker, Verity Hill, John T. McCrone, Nicholas Mohammed, Anushka Ramjag, Arianne Brown Jordan, Sarah C. Hill, Risha Singh, Sue-Min Nathaniel-Girdharrie, Avery Hinds, Nuala Ramkissoon, Kris V. Parag, Naresh Nandram, Roshan Parasram, Zobida Khan-Mohammed, Lisa Edghill, Lisa Indar, Aisha Andrewin, Rhonda Sealey-Thomas, Pearl McMillan, Ayoola Oyinloye, Kenneth George, Irad Potter, John Lee, David Johnson, Shawn Charles, Narine Singh, Jacquiline Bisesor-McKenzie, Hazel Laws, Sharon Belmar-George, Simone Keizer-Beache, Sharra Greenaway-Duberry, Nadia Ashwood, Jerome E. Foster, Karla Georges, Rahul Naidu, Marsha Ivey, Stanley Giddings, Rajini Haraksingh, Adesh Ramsubhag, Jayaraj Jayaraman, Chinnaraja Chinnadurai, Christopher Oura, Oliver G. Pybus, Joy St. John, Gabriel Gonzalez-Escobar, Nuno R. Faria, Christine V. F. Carrington

In the Funding section, funding sources were omitted. The correct Funding Statement is: This work was supported by grants to C. V. F. Carrington from The University of the West Indies—Trinidad &Tobago Research Development Impact Fund (No. 26607–447524), Pan American Health Organization / World Health Organisation (PAHO/WHO; SCON2021-00379), Government of the Republic of Trinidad and Tobago Ministry of Health (He: 10/45/35 Vol 1) and the AHF Global Public Health Institute, and in-kind support from PAHO/WHO and the Caribbean Public Health Agency. Also, a Medical Research Council-Sao Paulo Research Foundation (FAPESP) CADDE partnership award (MR/S0195/1 and FAPESP 18/14389-0) and a Wellcome Trust and Royal Society (Sir Henry Dale Fellowship: 204311/Z/16/Z) to N. Faria. S. C. Hill acknowledges support of a Sir Henry Wellcome Postdoctoral Fellowship (Wellcome Trust grant number 220414/Z/20/Z). The funders had no role in study design, data collection and analysis, decision to publish, or preparation of the manuscript.
