# Supplementary figures and images for: Implementation of genomic surveillance of SARS-CoV-2 in the Caribbean: Lessons learned for sustainability in resource-limited settings
Source: PLOS Glob Public Health. 2023 Feb 22;3(2):e0001455. doi: 10.1371/journal.pgph.0001455 (PMC10022082; doi:10.1371/journal.pgph.0001455)

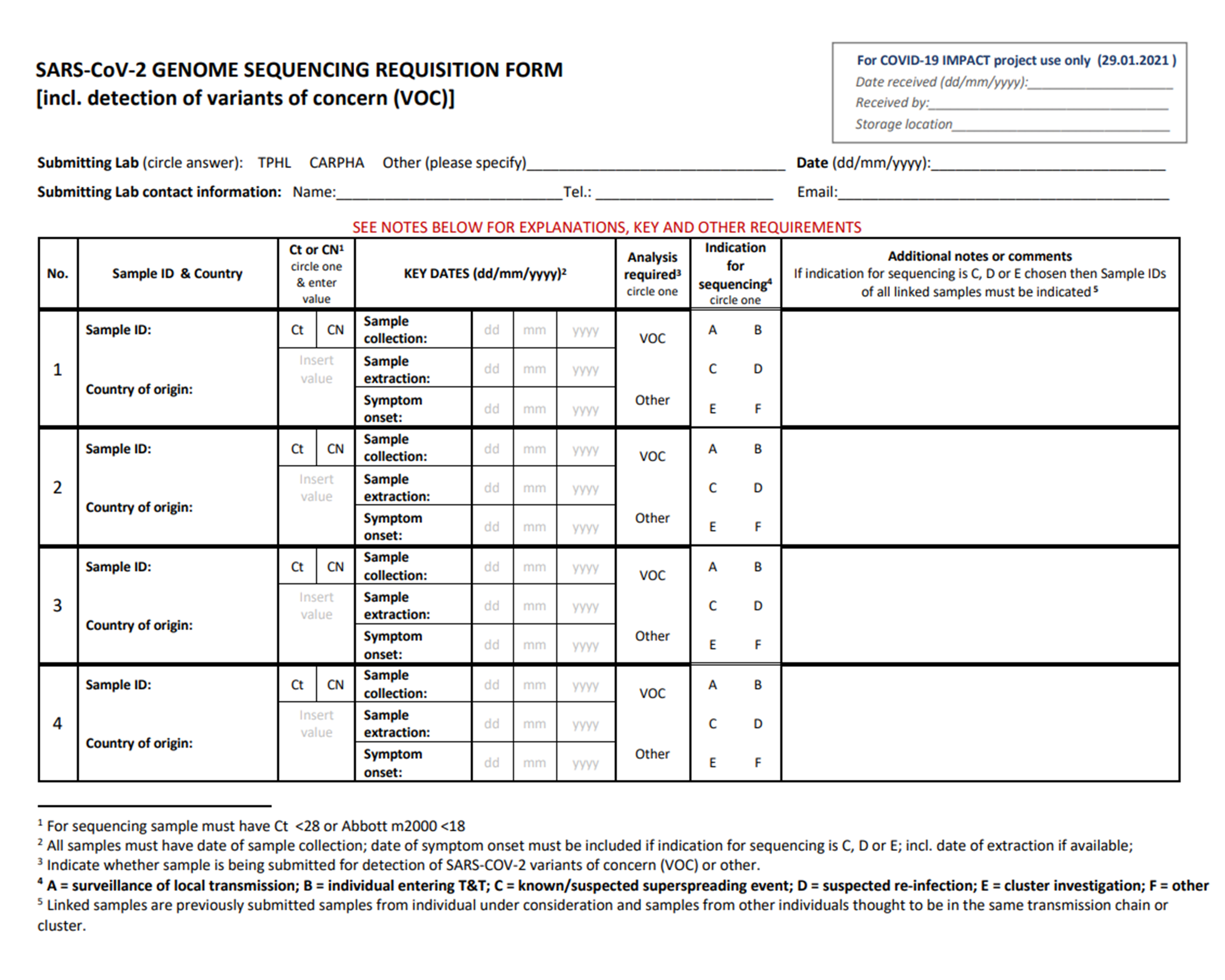

Supplement: S1 Fig — (TIF) [file pgph.0001455.s001.tif]

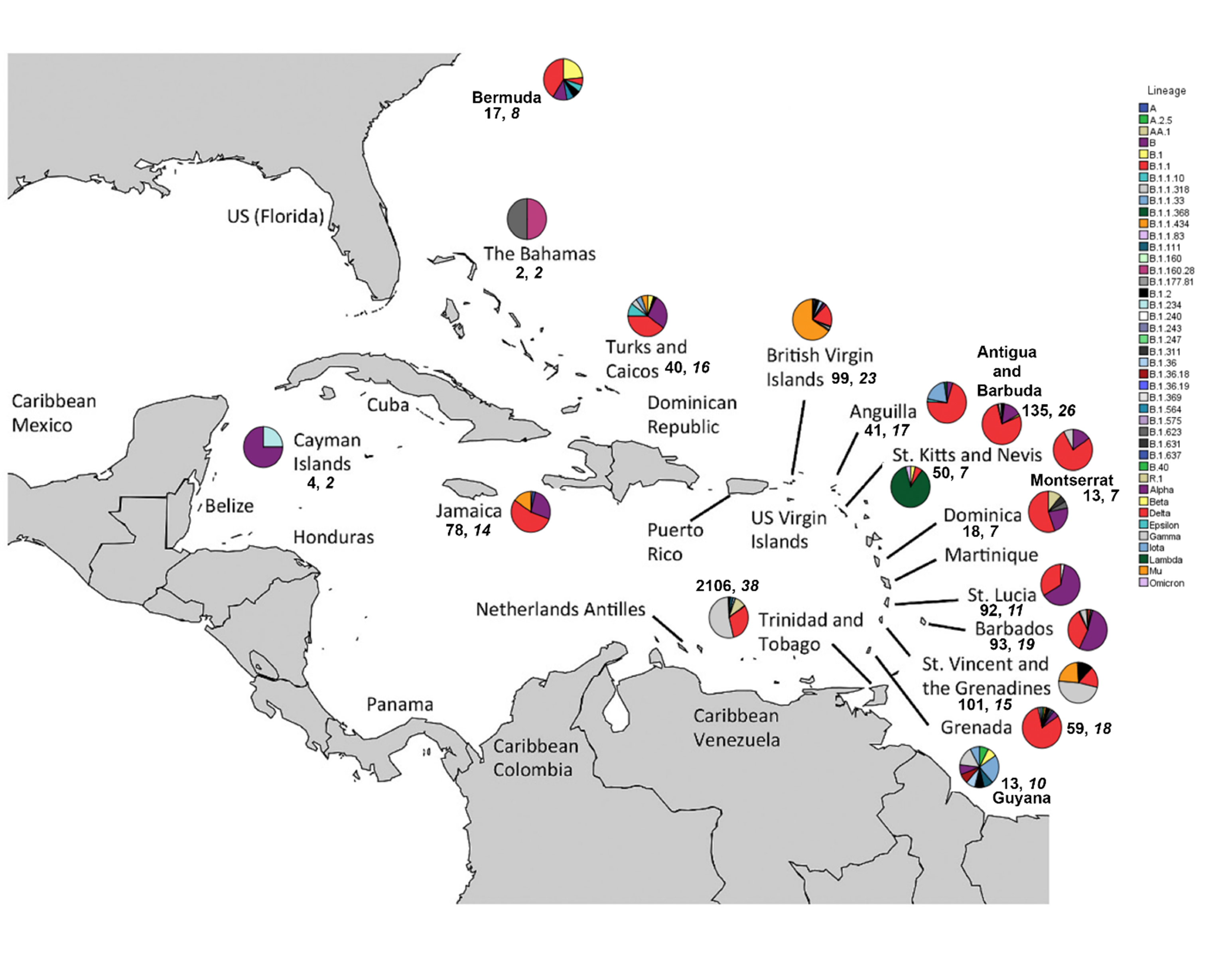

Supplement: S2 Fig — For each of the 17 CARPHA territories participating in Project IMPACT (Anguilla, Antigua and Barbuda, Bahamas, Barbados, Bermuda, British Virgin Islands, Cayman Islands, Dominica, Grenada, Guyana, Jamaica, Montserrat, Saint Kitts and Nevis, Saint Lucia, Saint Vincent and the Grenadines, Turks and Caicos Islands and T&T) the percentage of each lineage identified is indicated in a pie chart. The percentage of sequences from each country for which insufficient coverage was obtained to be able to assign a Pango lineage is not shown. From December 5th 2020 to December 31st 2021 a total of 3610 samples were sequenced. Sufficient coverage was obtained for 2975 (82.4% of all samples sequenced) to enable assignment to a Pango lineage. The numbers below the pie charts indicate respectively the number of sequences and lineages identified in each country, as at December 31, 2021. Ninety-one individual lineages were identified, including all 5 VOCs (Alpha, Beta, Gamma, Delta and Omicron) and two VOIs (Epsilon, Iota, Lambda, and Mu). Sub lineages for the VOCs and VOIs identified are grouped together using the WHO classifications. VOC = Variants of Concern. VOI = Variants of Interest. Map base layer modified from Serafy et al. 2015 [28] (License CC by 4.0; available at https://doi.org/10.1371/journal.pone.0142022.g001). (TIF) [file pgph.0001455.s002.tif]

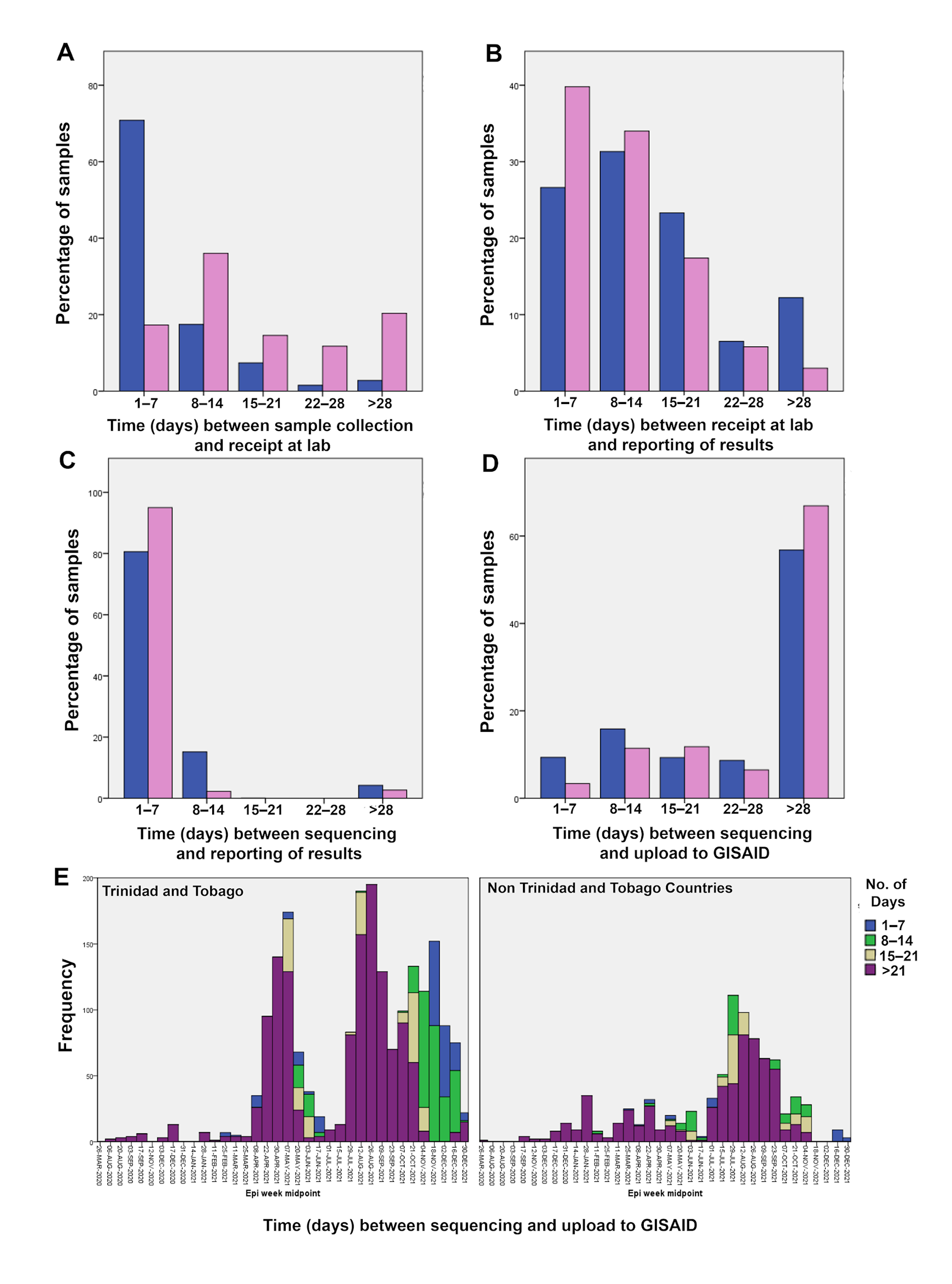

Supplement: S3 Fig — In panels A-D samples originating from T&T are indicated in blue and samples originating from non-T&T CMS are indicated in pink. (A) Time taken for samples to be received at the COVID-19 IMPACT laboratory after collection. Date of sample collection and date of receipt at the sequencing laboratory were available for 3463 samples sent for sequencing. (B) Time taken for sequencing to be reported once samples have been received by the COVID-19 IMPACT laboratory. This includes time for storage upon receipt, sample processing and library preparation, sequencing and bioinformatics pipeline analysis. Regardless of the sending institution, results for all T&T samples (n = 2596) were reported to the T&T MoH while results for samples coming from CARPHA were reported to CARPHA (n = 1670). Where multiple reports were made (e.g. T&T samples received from CARPHA reported to both CARPHA and T&T MoH) the earliest report date was used. (C) Time taken for results to be reported once having been processed and sequenced. Results for all T&T samples (regardless of the sending institution; n = 2596) were reported to the T&T MoH while results for samples coming from CARPHA (n = 1670) were reported to CARPHA. Where multiple reports were made, the earliest report date was used. (D) Time from date sequenced to upload to the GISAID database. 2828 sequences were uploaded to GISAID as at December 31 2021 (E) Time taken for sequences to be uploaded to the GISAID database after sequencing per week, for samples originating from Trinidad and Tobago and non-Trinidad and Tobago countries. The time taken in days for sequences (n = 2864) to be uploaded to GISAID after generation is indicated for sequences generated from T&T samples (left) and non-T&T samples (right). On the x-axis the time of sample collection (epidemiological week midpoint) is indicated for the samples from which the sequences were derived. T&T = Trinidad and Tobago. T&T MoH = Trinidad and Tobago Ministry of Health. CARPHA = Caribbean [file pgph.0001455.s003.tif]

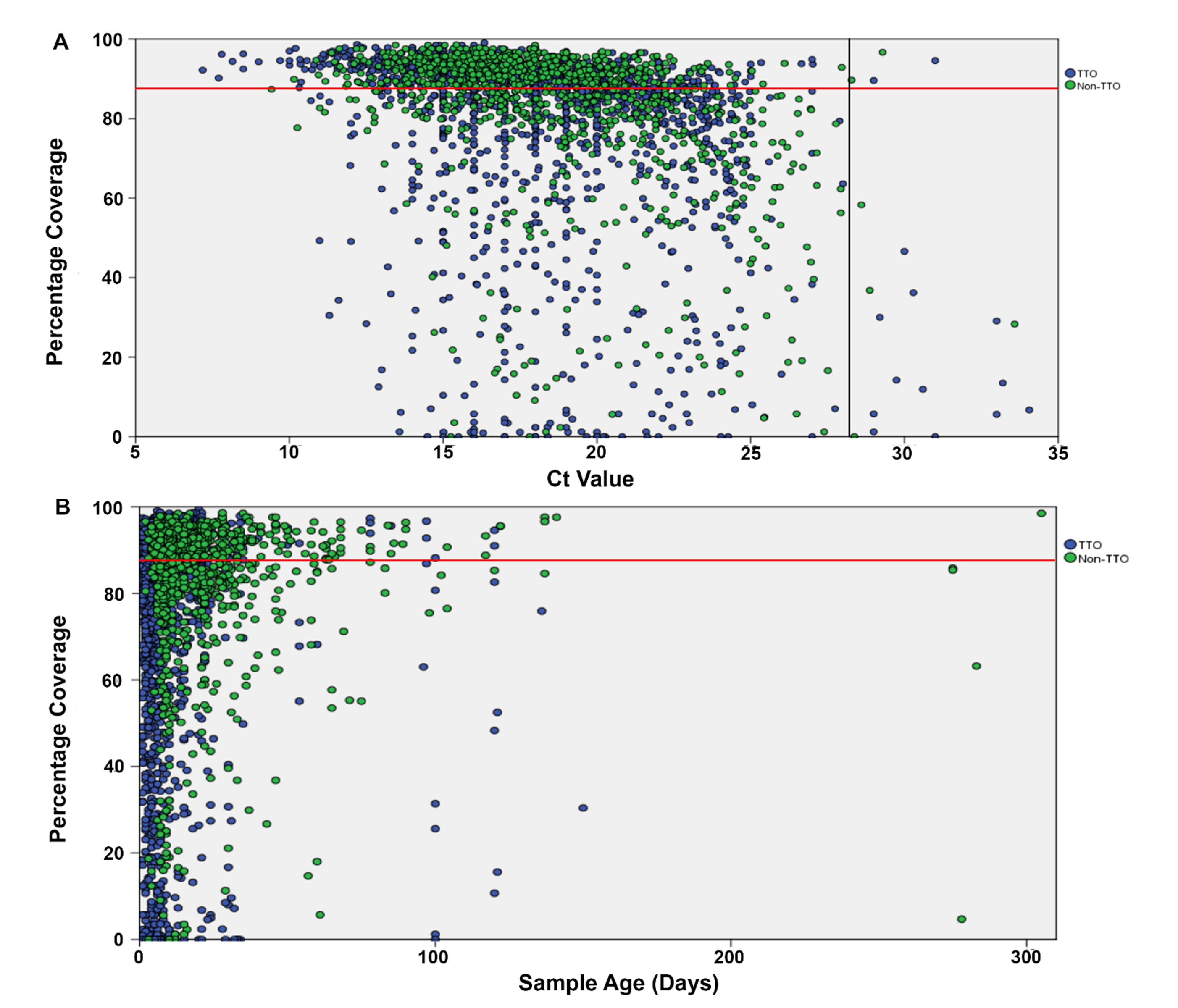

Supplement: S4 Fig — Percentage Genome Coverage of SARS-CoV-2 Sequences retrieved versus (A) Ct Value and (B) Sample Age. Samples originating from Trinidad and Tobago (T&T) are indicated in blue while those from other CARPHA member states (CMS) (overseas) are indicated in green. The median of the frequency distribution for percentage of the SARS-CoV-2 genome retrieved (89%) is indicated by the red line and the maximum Ct value specified in the criteria for sequencing (Ct = 28) is indicated by the black line was requested for samples to be sent for sequencing. Sample age is the length of time from the date a sample was collected from a COVID-19 case to the date the sample was sequenced. (TIF) [file pgph.0001455.s004.tif]
