# Supplementary material for: Implementation of genomic surveillance of SARS-CoV-2 in the Caribbean: Lessons learned for sustainability in resource-limited settings
Source: PLOS Glob Public Health. 2023 Feb 22;3(2):e0001455. doi: 10.1371/journal.pgph.0001455 (PMC10022082; doi:10.1371/journal.pgph.0001455)
Supplement: S1 Table — (DOCX) [file pgph.0001455.s005.docx]

**S1 Table. Frequencies of the individual Pango lineages identified in each of the 17 Caribbean Public Health Agency member states.**

| **Country** | **Lineage** | **Frequency** | **Percent** |  | **Country** | **Lineage** | **Frequency** | **Percent** |
| --- | --- | --- | --- | --- | --- | --- | --- | --- |
| **Anguilla** | AY.100 | 15 | 31.9 |  | **Guyana** | A.2.5 | 1 | 6.7 |
|  | AY.114 | 1 | 2.1 |  |  | B.1 | 1 | 6.7 |
|  | AY.122.1 | 2 | 4.3 |  |  | B.1.1.33 | 3 | 20 |
|  | AY.125 | 2 | 4.3 |  |  | B.1.1.7 | 1 | 6.7 |
|  | AY.14 | 1 | 2.1 |  |  | B.1.111 | 1 | 6.7 |
|  | AY.26 | 1 | 2.1 |  |  | B.1.2 | 1 | 6.7 |
|  | AY.35 | 1 | 2.1 |  |  | B.1.36 | 1 | 6.7 |
|  | AY.39 | 1 | 2.1 |  |  | B.1.36.18 | 1 | 6.7 |
|  | AY.4 | 1 | 2.1 |  |  | B.1.526 | 1 | 6.7 |
|  | AY.42 | 1 | 2.1 |  |  | P.1 | 2 | 13.3 |
|  | AY.47 | 1 | 2.1 |  |  | Sub-Total | 13 | 86.7 |
|  | AY.9.2 | 1 | 2.1 |  |  | Failed | 2 | 13.3 |
|  | B.1.1.7 | 2 | 4.3 |  |  | TOTAL | 15 | 100 |
|  | B.1.427 | 1 | 2.1 |  |  |  |  |  |
|  | B.1.526 | 8 | 17 |  | **Jamaica** | AY.100 | 1 | 1.1 |
|  | B.1.617.2 | 1 | 2.1 |  |  | AY.107 | 9 | 9.5 |
|  | C.37 | 1 | 2.1 |  |  | AY.110 | 3 | 3.2 |
|  | Sub-Total | 41 | 87.2 |  |  | AY.25 | 8 | 8.4 |
|  | Failed | 6 | 12.8 |  |  | AY.26 | 2 | 2.1 |
|  | TOTAL | 47 | 100 |  |  | AY.3 | 5 | 5.3 |
|  |  |  |  |  |  | AY.35 | 4 | 4.2 |
| **Antigua and Barbuda** | AY.100 | 1 | 0.6 |  |  | AY.44 | 2 | 2.1 |
|  | AY.103 | 1 | 0.6 |  |  | AY.9 | 8 | 8.4 |
|  | AY.119 | 3 | 1.8 |  |  | B.1.1.7 | 21 | 22.1 |
|  | AY.122 | 1 | 0.6 |  |  | B.1.240 | 1 | 1.1 |
|  | AY.122.1 | 1 | 0.6 |  |  | B.1.621 | 1 | 1.1 |
|  | AY.25 | 1 | 0.6 |  |  | B.1.621.1 | 11 | 11.6 |
|  | AY.34 | 1 | 0.6 |  |  | B.1.637 | 2 | 2.1 |
|  | AY.35 | 1 | 0.6 |  |  | Sub-Total | 78 | 82.1 |
|  | AY.39 | 32 | 19.6 |  |  | Failed | 17 | 17.9 |
|  | AY.4 | 2 | 1.2 |  |  | TOTAL | 95 | 100 |
|  | AY.44 | 6 | 3.7 |  |  |  |  |  |
|  | AY.49 | 1 | 0.6 |  | **Montserrat** | AY.107 | 5 | 33.3 |
|  | AY.5 | 1 | 0.6 |  |  | AY.39 | 1 | 6.7 |
|  | AY.75 | 3 | 1.8 |  |  | AY.47 | 1 | 6.7 |
|  | AY.88 | 1 | 0.6 |  |  | AY.98 | 1 | 6.7 |
|  | AY.89 | 1 | 0.6 |  |  | B.1.1.7 | 2 | 13.3 |
|  | AY.98 | 26 | 16 |  |  | B.1.617.2 | 2 | 13.3 |
|  | B.1.1.318 | 1 | 0.6 |  |  | P.1 | 1 | 6.7 |
|  | B.1.1.7 | 20 | 12.3 |  |  | Sub-Total | 13 | 86.7 |
|  | B.1.2 | 1 | 0.6 |  |  | Failed | 2 | 13.3 |
|  | B.1.351 | 2 | 1.2 |  |  | TOTAL | 15 | 100 |
|  | B.1.429 | 1 | 0.6 |  |  |  |  |  |
|  | B.1.526 | 1 | 0.6 |  | **Saint Kitts and Nevis** | AY.122 | 1 | 1.8 |
|  | B.1.575 | 1 | 0.6 |  |  | AY.39 | 1 | 1.8 |
|  | B.1.617.2 | 22 | 13.5 |  |  | AY.75 | 1 | 1.8 |
|  | P.1 | 3 | 1.8 |  |  | B.1 | 2 | 3.6 |
|  | Sub-Total | 135 | 82.8 |  |  | BA.1 | 2 | 3.6 |
|  | Failed | 28 | 17.2 |  |  | C.37 | 41 | 73.2 |
|  | TOTAL | 163 | 100 |  |  | C.37.1 | 2 | 3.6 |
|  |  |  |  |  |  | Sub-Total | 50 | 89.3 |
| **Bahamas** | B.1.160.28 | 1 | 25 |  |  | Failed | 6 | 10.7 |
|  | B.1.623 | 1 | 25 |  |  | TOTAL | 56 | 100 |
|  | Sub-Total | 2 | 50 |  |  |  |  |  |
|  | Failed | 2 | 50 |  | **Saint Lucia** | AY.103 | 1 | 1 |
|  | TOTAL | 4 | 100 |  |  | AY.105 | 1 | 1 |
|  |  |  |  |  |  | AY.120 | 1 | 1 |
| **Barbados** | AY.107 | 1 | 0.9 |  |  | AY.25 | 11 | 10.6 |
|  | AY.11 | 2 | 1.8 |  |  | AY.3 | 1 | 1 |
|  | AY.119 | 4 | 3.5 |  |  | AY.34 | 12 | 11.5 |
|  | AY.120 | 2 | 1.8 |  |  | AY.43 | 2 | 1.9 |
|  | AY.122.1 | 1 | 0.9 |  |  | AY.44 | 1 | 1 |
|  | AY.25 | 1 | 0.9 |  |  | AY.5 | 1 | 1 |
|  | AY.39 | 5 | 4.4 |  |  | B.1.1.7 | 58 | 55.8 |
|  | AY.42 | 1 | 0.9 |  |  | B.1.369 | 3 | 2.9 |
|  | AY.44 | 5 | 4.4 |  |  | Sub-Total | 92 | 88.5 |
|  | AY.5 | 1 | 0.9 |  |  | Failed | 12 | 11.5 |
|  | AY.75 | 1 | 0.9 |  |  | TOTAL | 104 | 100 |
|  | B.1.1 | 2 | 1.8 |  |  |  |  |  |
|  | B.1.1.7 | 49 | 43.4 |  | **Saint Vincent and the Grenadines** | AY.113 | 2 | 1.9 |
|  | B.1.160 | 1 | 0.9 |  |  | AY.122 | 1 | 0.9 |
|  | B.1.243 | 1 | 0.9 |  |  | AY.25 | 1 | 0.9 |
|  | B.1.429 | 1 | 0.9 |  |  | AY.34 | 1 | 0.9 |
|  | B.1.617.2 | 9 | 8 |  |  | AY.43 | 1 | 0.9 |
|  | B.1.621.1 | 1 | 0.9 |  |  | AY.5 | 8 | 7.5 |
|  | P.1 | 5 | 4.4 |  |  | AY.6 | 2 | 1.9 |
|  | Sub-Total | 93 | 82.3 |  |  | B.1.2 | 12 | 11.2 |
|  | Failed | 20 | 17.7 |  |  | B.1.617.2 | 1 | 0.9 |
|  | TOTAL | 113 | 100 |  |  | B.1.621 | 1 | 0.9 |
|  |  |  |  |  |  | B.1.621.1 | 22 | 20.6 |
| **Bermuda** | AY.119 | 6 | 33.3 |  |  | BA.1 | 1 | 0.9 |
|  | AY.25 | 1 | 5.6 |  |  | P.1 | 45 | 42.1 |
|  | B.1 | 4 | 22.2 |  |  | P.1.12 | 2 | 1.9 |
|  | B.1.1 | 1 | 5.6 |  |  | P.1.14 | 1 | 0.9 |
|  | B.1.1.10 | 1 | 5.6 |  |  | Sub-Total | 101 | 94.4 |
|  | B.1.1.7 | 2 | 11.1 |  |  | Failed | 6 | 5.6 |
|  | B.1.2 | 1 | 5.6 |  |  | TOTAL | 107 | 100 |
|  | B.1.564 | 1 | 5.6 |  |  |  |  |  |
|  | Sub-Total | 17 | 94.4 |  | **Trinidad and Tobago** | A | 1 | 0 |
|  | Failed | 1 | 5.6 |  |  | A.2.5 | 1 | 0 |
|  | TOTAL | 18 | 100 |  |  | AY.100 | 1 | 0 |
|  |  |  |  |  |  | AY.103 | 57 | 2.2 |
| **British Virgin Islands** | AY.119 | 1 | 0.8 |  |  | AY.113 | 2 | 0.1 |
|  | AY.122 | 1 | 0.8 |  |  | AY.119 | 2 | 0.1 |
|  | AY.122.1 | 4 | 3.3 |  |  | AY.122 | 1 | 0 |
|  | AY.25 | 1 | 0.8 |  |  | AY.25 | 460 | 17.7 |
|  | AY.35 | 1 | 0.8 |  |  | AY.26 | 1 | 0 |
|  | AY.44 | 1 | 0.8 |  |  | AY.3 | 10 | 0.4 |
|  | AY.56 | 1 | 0.8 |  |  | AY.33 | 1 | 0 |
|  | AY.75 | 1 | 0.8 |  |  | AY.36 | 3 | 0.1 |
|  | AY.88 | 4 | 3.3 |  |  | AY.39 | 81 | 3.1 |
|  | AY.98 | 1 | 0.8 |  |  | AY.4 | 1 | 0 |
|  | B.1.1.368 | 1 | 0.8 |  |  | AY.42 | 4 | 0.2 |
|  | B.1.1.7 | 2 | 1.7 |  |  | AY.69 | 4 | 0.2 |
|  | B.1.1.83 | 1 | 0.8 |  |  | AY.84 | 1 | 0 |
|  | B.1.177.81 | 1 | 0.8 |  |  | AY.88 | 2 | 0.1 |
|  | B.1.2 | 2 | 1.7 |  |  | AY.9.2 | 5 | 0.2 |
|  | B.1.243 | 1 | 0.8 |  |  | B | 1 | 0 |
|  | B.1.36 | 3 | 2.5 |  |  | B.1 | 3 | 0.1 |
|  | B.1.429 | 1 | 0.8 |  |  | B.1.1 | 15 | 0.6 |
|  | B.1.526 | 1 | 0.8 |  |  | B.1.1.33 | 3 | 0.1 |
|  | B.1.617.2 | 2 | 1.7 |  |  | B.1.1.7 | 9 | 0.3 |
|  | B.1.621.1 | 65 | 54.2 |  |  | B.1.111 | 52 | 2 |
|  | B.1.623 | 1 | 0.8 |  |  | B.1.247 | 1 | 0 |
|  | P.1 | 2 | 1.7 |  |  | B.1.36 | 38 | 1.5 |
|  | Sub-Total | 99 | 82.5 |  |  | B.1.36.19 | 1 | 0 |
|  | Failed | 21 | 17.5 |  |  | B.1.617.2 | 10 | 0.4 |
|  | TOTAL | 120 | 100 |  |  | B.1.631 | 2 | 0.1 |
|  |  |  |  |  |  | BA.1 | 12 | 0.5 |
| **Cayman Islands** | B.1.1.7 | 3 | 42.9 |  |  | P.1 | 1081 | 41.6 |
|  | B.1.234 | 1 | 14.3 |  |  | P.1.1 | 3 | 0.1 |
|  | Sub-Total | 4 | 57.1 |  |  | P.1.12 | 32 | 1.2 |
|  | Failed | 3 | 42.9 |  |  | P.1.13 | 3 | 0.1 |
|  | TOTAL | 7 | 100 |  |  | P.1.14 | 4 | 0.2 |
|  |  |  |  |  |  | P.1.15 | 1 | 0 |
| **Dominica** | AA.1 | 2 | 10 |  |  | R.1 | 197 | 7.6 |
|  | AY.122.1 | 5 | 25 |  |  | Sub-Total | 2106 | 81.1 |
|  | AY.125 | 1 | 5 |  |  | Failed | 491 | 18.9 |
|  | B.1.1.7 | 4 | 20 |  |  | TOTAL | 2597 | 100 |
|  | B.1.311 | 1 | 5 |  |  |  |  |  |
|  | B.1.617.2 | 4 | 20 |  | **Turks and Caicos Islands** | AY.103 | 2 | 4.3 |
|  | B.1.623 | 1 | 5 |  |  | AY.14 | 1 | 2.1 |
|  | Sub-Total | 18 | 90 |  |  | AY.25 | 3 | 6.4 |
|  | Failed | 2 | 10 |  |  | AY.39 | 3 | 6.4 |
|  | TOTAL | 20 | 100 |  |  | AY.39.1 | 3 | 6.4 |
|  |  |  |  |  |  | AY.44 | 2 | 4.3 |
| **Grenada** | A.2.5 | 1 | 1.4 |  |  | B.1 | 2 | 4.3 |
|  | AY.103 | 1 | 1.4 |  |  | B.1.1.7 | 11 | 23.4 |
|  | AY.122 | 3 | 4.3 |  |  | B.1.2 | 1 | 2.1 |
|  | AY.25 | 1 | 1.4 |  |  | B.1.429 | 4 | 8.5 |
|  | AY.39 | 8 | 11.6 |  |  | B.1.526 | 2 | 4.3 |
|  | AY.39.1 | 1 | 1.4 |  |  | B.1.617.2 | 2 | 4.3 |
|  | AY.44 | 2 | 2.9 |  |  | B.1.621 | 1 | 2.1 |
|  | AY.5 | 7 | 10.1 |  |  | B.1.621.1 | 1 | 2.1 |
|  | AY.75 | 2 | 2.9 |  |  | P.1 | 1 | 2.1 |
|  | AY.88 | 1 | 1.4 |  |  | P.1.17 | 1 | 2.1 |
|  | B.1.1.434 | 1 | 1.4 |  |  | Sub-Total | 40 | 85.1 |
|  | B.1.1.7 | 3 | 4.3 |  |  | Failed | 7 | 14.9 |
|  | B.1.2 | 2 | 2.9 |  |  | TOTAL | 47 | 100 |
|  | B.1.526 | 1 | 1.4 |  |  |  |  |  |
|  | B.1.617.2 | 22 | 31.9 |  |  |  |  |  |
|  | B.1.623 | 1 | 1.4 |  |  |  |  |  |
|  | B.1.637 | 1 | 1.4 |  |  |  |  |  |
|  | P.1.1 | 1 | 1.4 |  |  |  |  |  |
|  | Sub-Total | 59 | 85.5 |  |  |  |  |  |
|  | Failed | 10 | 14.5 |  |  |  |  |  |
|  | TOTAL | 69 | 100 |  |  |  |  |  |
