# Supplementary material for: Implementation of genomic surveillance of SARS-CoV-2 in the Caribbean: Lessons learned for sustainability in resource-limited settings
Source: PLOS Glob Public Health. 2023 Feb 22;3(2):e0001455. doi: 10.1371/journal.pgph.0001455 (PMC10022082; doi:10.1371/journal.pgph.0001455)
Supplement: S2 Table — (DOCX) [file pgph.0001455.s006.docx]

**S2 Table. Key challenges encountered during the COVID-19 IMPACT project.**

| **Category** | **Challenge(s)** | **Reason(s)** | **Action(s) Taken** | **Lessons learned** |
| --- | --- | --- | --- | --- |
| Procurement | Periodic lack of reagents and consumables | Slow and inefficient institutional procurement procedures.  Responses to COVID-19 public health restrictions (e.g., implementation of an appointment system by T&T Customs and Excise Division resulting in back log of items to be cleared).  Supplier shortages. | Implementation of an inventory tracking system specific to SARS-CoV-2 sequencing and hiring of a laboratory manager to oversee this and other operational logistics.  Informing administrative staff in relevant institutional offices about the importance of genomic surveillance and the COVID-19 IMPACT laboratory’s role in the public health response.  Formal requests to Comptroller Customs and Excise Division for expedited clearance of  items required for SARS-CoV-2 genomic sequencing, including information about its importance for COVID-19 mitigation and  control efforts. | Institutions should adapt processes and implement policies that allow for greater agility during health emergencies.  It is important to sensitise stakeholders about the relevance of genomic surveillance to public health responses and the impact of their role on these efforts. |
| Bioinformatic workflow | Inefficient manual workflow for data entry, data analysis, database management and generation of reports. | Lack of personnel with the coding skills required to automate workflow.    Insufficient computational power.    Insufficient data storage capacity. | Hiring of personnel with expertise in bioinformatics and computer coding.    Acquisition of network-attached storage, additional computers and higher throughput sequencing device with onboard compute (i.e., ONT GridION) | IT support, computer-coding skills and appropriate data storage are essential. |
| Metadata availability | Samples received for sequencing with limited metadata. | Only Ct/CN value, type of sample, date and location of sample collection were explicitly specified in the initial study protocol.  Lack of awareness at sample collection sites about the importance of metadata and impact of incompletely filling the sequencing requisition forms.  Different data sharing policies across institutions.  Limited manpower in institutions where the required metadata was held.  Need to preserve confidentiality meant that sequencing laboratory personnel could not independently retrieve metadata. | Development and dissemination of a standardised, more comprehensive sample requisition form with fields for entry of other categories of metadata.  Retrospective retrieval of metadata. | Anticipate the need for metadata beyond essential requirements and design data retrieval system accordingly.  Stakeholders should be sensitised to the utility and potential public health impact of genomic surveillance.  An integrated and secure data sharing system across stakeholder institutions that allows different levels of data access for different user categories is essential for optimal data sharing. |
| Sample quality | Low genome coverage or sequencing failure (despite low Ct/CN values) | *Possible reasons:*  RNA sample degradation subsequent to Ct/CN value determination (e.g. due to inadequate storage and / or transportation conditions).    Lack of standardisation of extraction methods across submitting laboratories.  Re-extracted samples submitted to the COVID-19 IMPACT sequencing laboratory with Ct /CN values from the initial extraction (i.e. not from re-extracted samples). | Implementation of a quality control step during library preparation to identify samples with insufficient viral RNA. | Protocols should be standardised across institutions as far as possible.  Collect detailed information on nucleic extraction protocols and storage conditions used by laboratories submitting samples including comments on deviations from the standard protocol.  Implement routine QC checks to confirm reported Ct/CN values prior to sequencing.  Consider receiving primary samples (e.g. nasopharyngeal swab) at the sequencing laboratory instead of extracted samples.  Ensure institutions handling samples have appropriate sample storage and transportation systems. |
| Sample transport logistics | Long turnaround times (especially for samples originating from outside of T&T i.e., from overseas). | Logistics of overseas transport (including impact of COVID-19 public health restrictions and border closures).  Delaying of submissions to the COVID-19 IMPACT laboratory to accommodate batching of samples.  Submission of samples to the COVID-19 IMPACT laboratory via intermediary institutions (CARPHA, TPHL) added to "sample collection to sequencing laboratory receipt" time. | None. | Build in-country capacity for genomic sequencing and data analysis.  The efficiency of sample transport systems and their flexibility to adapt during health emergencies are important considerations. |
| Sequencing Capacity | Longer turnaround times and worker burnout during periods of high demand | Limited manpower.  Lack of high-throughput sequencing devices.  Inefficient manual workflow for data entry, sequencing, data analysis, database management and generation of reports. | Hired additional laboratory personnel including laboratory technicians, research assistants, laboratory coordinator and an individual with expertise in bioinformatics and computer coding skills.  Purchase of higher throughput (ONT GridION) sequencing devices. | Anticipate the need to rapidly scale operations to meet demand.  Human resource capacity building during inter-epidemic periods will be essential to ensure that there is a pool of appropriately trained individuals in times of need. |
| Data integration and analysis for maximum public health impact | Sub-optimal impact on pandemic response | Lack of metadata to put genomic data in context or perform more in-depth analyses (e.g., cluster investigations, investigations into patterns of viral population growth, transmission and spread, evaluation of drivers of transmission).  Limited time for COVID-19 IMPACT laboratory personnel to dedicate to activities / analyses beyond routine sequencing and lineage identification given the manpower limitations. | Retrospective metadata retrieval and data analysis. | Develop a digital infrastructure for collecting and integrating genomic sequencing data, sequence metadata and case/death surveillance data.  For partnerships between academic and public health institutions to have maximum impact, it is important to ensure that responsibilities and tasks (e.g. research investigations and in-depth analyses versus routine surveillance) are appropriately resourced and assigned. |
