# Supplementary material for: Implementation of genomic surveillance of SARS-CoV-2 in the Caribbean: Lessons learned for sustainability in resource-limited settings
Source: PLOS Glob Public Health. 2023 Feb 22;3(2):e0001455. doi: 10.1371/journal.pgph.0001455 (PMC10022082; doi:10.1371/journal.pgph.0001455)
Supplement: S3 File — (PDF) [file pgph.0001455.s009.pdf]

SAMPLE META DATA

Sample ID	Country of origin	Sample Collection Date	Sending Institution	Clinical / Demographic data provided (Y/N)						Ct Value	CN Value	Date Received by COVID-19 IMPACT lab	Date sequenced	Date reported to MOH	Date reported to CARPHA	GISAID Upload Date
				Age	Date of Onset of Symptoms	Sex	TravelHistory	Town/County	AdditionalData							
C19IMPACT_0001	Trinidad and Tobago	14-Aug-20	MoH	Y	N	Y	N	Y	N	14.87			05-Dec-20	04-Jan-21		
C19IMPACT_0002	Trinidad and Tobago	19-Aug-20	MoH	Y	N	Y	N	Y	N	15.61			05-Dec-20	04-Jan-21		
C19IMPACT_0003	Trinidad and Tobago	19-Aug-20	MoH	N	N	N	N	N	N	34.06			05-Dec-20			
C19IMPACT_0004	Trinidad and Tobago	20-Aug-20	MoH	Y	N	Y	N	N	N	25.34			05-Dec-20			21-Jan-22
C19IMPACT_0005	Trinidad and Tobago	24-Aug-20	MoH	N	N	Y	N	N	N	21.55			05-Dec-20	04-Jan-21		
C19IMPACT_0006	Trinidad and Tobago	26-Aug-20	MoH	N	N	Y	N	Y	N	16.86			05-Dec-20	04-Jan-21		
C19IMPACT_0007	Trinidad and Tobago	27-Aug-20	MoH	N	N	N	N	N	N	30.29			05-Dec-20			
C19IMPACT_0008	Trinidad and Tobago	29-Aug-20	MoH	Y	N	Y	N	Y	N	14.89			05-Dec-20	04-Jan-21		
C19IMPACT_0009	Trinidad and Tobago	02-Sept-20	MoH	N	N	Y	N	Y	N	14.76			05-Dec-20	04-Jan-21		
C19IMPACT_0010	Trinidad and Tobago	09-Sept-20	MoH	Y	N	Y	N	Y	N	17.55			05-Dec-20	04-Jan-21		
C19IMPACT_0011	Trinidad and Tobago	14-Sept-20	MoH	Y	N	Y	N	Y	N	14.95			05-Dec-20	04-Jan-21		
C19IMPACT_0012	Trinidad and Tobago	01-Aug-20	MoH	Y	N	Y	N	Y	N	19.47			16-Dec-20			21-Jan-22
C19IMPACT_0013	Trinidad and Tobago	10-Aug-20	MoH	Y	N	Y	N	N	N	18.80			16-Dec-20			21-Jan-22
C19IMPACT_0014	Trinidad and Tobago	18-Aug-20	MoH	Y	N	Y	N	N	N	18.69			16-Dec-20			
C19IMPACT_0015	Trinidad and Tobago	03-Sept-20	MoH	Y	N	Y	N	Y	N	17.02			16-Dec-20			21-Jan-22
C19IMPACT_0016	Trinidad and Tobago	13-Sept-20	MoH	Y	N	Y	N	N	N	20.49			16-Dec-20			21-Jan-22
C19IMPACT_0017	Trinidad and Tobago	14-Sept-20	MoH	N	N	N	N	N	N	19.92			16-Dec-20			21-Jan-22
C19IMPACT_0018	Trinidad and Tobago	11-Dec-20	MoH	N	N	N	N	N	N			01-Dec-20	24-Dec-20	04-Jan-21	02-Jan-21	21-Jan-22
C19IMPACT_0019	Trinidad and Tobago	17-Dec-20	MoH	N	N	N	N	N	N			01-Dec-20	24-Dec-20	04-Jan-21	02-Jan-21	
C19IMPACT_0020	Trinidad and Tobago		MoH	N	N	N	N	N	N			01-Dec-20	24-Dec-20	04-Jan-21	02-Jan-21	
C19IMPACT_0021	Trinidad and Tobago		MoH	N	N	N	N	N	N			01-Dec-20	24-Dec-20	04-Jan-21		
C19IMPACT_0022	Trinidad and Tobago		MoH	N	N	N	N	N	N			01-Dec-20	24-Dec-20	04-Jan-21	02-Jan-21	
C19IMPACT_0023	Trinidad and Tobago		MoH	N	N	N	N	N	N			01-Dec-20	24-Dec-20	04-Jan-21	02-Jan-21	
C19IMPACT_0024	Trinidad and Tobago		MoH	N	N	N	N	N	N			01-Dec-20	24-Dec-20	04-Jan-21	02-Jan-21	
C19IMPACT_0025	Trinidad and Tobago		MoH	N	N	N	N	N	N			01-Dec-20	24-Dec-20	04-Jan-21	02-Jan-21	
C19IMPACT_0026	Trinidad and Tobago		MoH	N	N	N	N	N	N			01-Dec-20	24-Dec-20	04-Jan-21	02-Jan-21	
C19IMPACT_0027	Trinidad and Tobago		MoH	N	N	N	N	N	N			01-Dec-20	24-Dec-20	04-Jan-21	02-Jan-21	
C19IMPACT_0028	Trinidad and Tobago		MoH	N	N	N	N	N	N			01-Dec-20	24-Dec-20	04-Jan-21	02-Jan-21	
C19IMPACT_0029	Trinidad and Tobago		MoH	N	N	N	N	N	N			01-Dec-20	24-Dec-20	04-Jan-21	02-Jan-21	
C19IMPACT_0030	Trinidad and Tobago		MoH	N	N	N	N	N	N			01-Dec-20	24-Dec-20	04-Jan-21	02-Jan-21	
C19IMPACT_0031	Trinidad and Tobago		MoH	N	N	N	N	N	N			01-Dec-20	24-Dec-20	04-Jan-21	02-Jan-21	
C19IMPACT_0032	Trinidad and Tobago		MoH	N	N	N	N	N	N			01-Dec-20	24-Dec-20	04-Jan-21	02-Jan-21	
C19IMPACT_0033	Trinidad and Tobago	23-Jul-20	CARPHA	N	N	N	N	N	N	23.11		20-Dec-20	28-Dec-20	04-Jan-21	31-Dec-20	
C19IMPACT_0034	Trinidad and Tobago	06-Aug-20	CARPHA	N	N	N	Y	N	N	17.73		20-Dec-20	28-Dec-20	04-Jan-21	31-Dec-20	
C19IMPACT_0035	Trinidad and Tobago	21-Aug-20	CARPHA	N	N	N	N	N	N	23.84		20-Dec-20	28-Dec-20	04-Jan-21	31-Dec-20	
C19IMPACT_0036	Trinidad and Tobago	21-Aug-20	CARPHA	N	N	N	N	N	N	18.90		20-Dec-20	28-Dec-20	04-Jan-21	31-Dec-20	
C19IMPACT_0037	Trinidad and Tobago	22-Aug-20	CARPHA	N	N	N	Y	N	N	15.81		20-Dec-20	28-Dec-20	04-Jan-21	31-Dec-20	06-Apr-21
C19IMPACT_0038	Trinidad and Tobago	22-Aug-20	CARPHA	N	N	N	Y	N	N	21.43		20-Dec-20	28-Dec-20	04-Jan-21	31-Dec-20	
C19IMPACT_0039	Trinidad and Tobago	22-Aug-20	CARPHA	N	N	N	N	N	N	10.57		20-Dec-20	28-Dec-20	04-Jan-21	31-Dec-20	
C19IMPACT_0040	Trinidad and Tobago	22-Aug-20	CARPHA	N	N	N	N	N	N	18.52		20-Dec-20	28-Dec-20	04-Jan-21	31-Dec-20	
C19IMPACT_0041	Trinidad and Tobago	22-Aug-20	CARPHA	N	N	N	N	N	N	22.84		20-Dec-20	28-Dec-20	04-Jan-21	31-Dec-20	
C19IMPACT_0042	Trinidad and Tobago	11-Sept-20	CARPHA	N	N	N	N	N	N	24.41		20-Dec-20	28-Dec-20	04-Jan-21	31-Dec-20	
C19IMPACT_0043	Trinidad and Tobago	11-Sept-20	CARPHA	N	N	N	N	N	N	15.95		20-Dec-20	28-Dec-20	04-Jan-21	31-Dec-20	
C19IMPACT_0044	Trinidad and Tobago	11-Sept-20	CARPHA	N	N	N	N	N	N	21.60		20-Dec-20	28-Dec-20	04-Jan-21	31-Dec-20	
C19IMPACT_0045	Trinidad and Tobago	11-Sept-20	CARPHA	N	N	N	N	N	N	23.87		20-Dec-20	28-Dec-20	04-Jan-21	31-Dec-20	
C19IMPACT_0046	Trinidad and Tobago	11-Sept-20	CARPHA	N	N	N	N	N	N	20.27		20-Dec-20	28-Dec-20	04-Jan-21	31-Dec-20	
C19IMPACT_0047	Trinidad and Tobago	11-Sept-20	CARPHA	N	N	N	N	N	N	21.49		20-Dec-20	28-Dec-20	04-Jan-21	31-Dec-20	
C19IMPACT_0048	Trinidad and Tobago	21-Oct-20	CARPHA	N	N	N	N	N	N	11.98		20-Dec-20	28-Dec-20	04-Jan-21	31-Dec-20	
C19IMPACT_0049	Trinidad and Tobago	27-Oct-20	CARPHA	N	N	N	N	N	N	16.56		20-Dec-20	28-Dec-20	04-Jan-21	31-Dec-20	
C19IMPACT_0050	Trinidad and Tobago	27-Oct-20	CARPHA	N	N	N	N	N	N	22.28		20-Dec-20	28-Dec-20	04-Jan-21	31-Dec-20	
C19IMPACT_0051	Trinidad and Tobago	27-Oct-20	CARPHA	N	N	N	N	N	N	19.62		20-Dec-20	28-Dec-20	04-Jan-21	31-Dec-20	
C19IMPACT_0052	Trinidad and Tobago	25-Nov-20	CARPHA	N	N	N	N	N	N	22.42		20-Dec-20	28-Dec-20	04-Jan-21	31-Dec-20	
C19IMPACT_0053	Trinidad and Tobago	26-Nov-20	CARPHA	N	N	N	N	N	N	22.92		20-Dec-20	28-Dec-20	04-Jan-21	31-Dec-20	
C19IMPACT_0054	Trinidad and Tobago	26-Nov-20	CARPHA	N	N	N	N	N	N	24.36		20-Dec-20	28-Dec-20	04-Jan-21	31-Dec-20	
C19IMPACT_0055	Trinidad and Tobago	26-Nov-20	CARPHA	N	N	N	N	N	N	21.18		20-Dec-20	28-Dec-20	04-Jan-21	31-Dec-20	
C19IMPACT_0056	Saint Kitts and Nevis	20-Mar-20	CARPHA	N	N	N	N	N	N	27.15		28-Dec-20	31-Dec-20		02-Jan-21	
C19IMPACT_0057	Montserrat	25-Mar-20	CARPHA	N	N	N	N	N	N	25.43		28-Dec-20	31-Dec-20		02-Jan-21	
C19IMPACT_0058	Saint Kitts and Nevis	28-Mar-20	CARPHA	N	N	N	N	N	N	26.00		28-Dec-20	31-Dec-20		02-Jan-21	
C19IMPACT_0059	Saint Kitts and Nevis	28-Mar-20	CARPHA	N	N	N	N	N	N	20.90		28-Dec-20	31-Dec-20		02-Jan-21	
C19IMPACT_0060	Trinidad and Tobago	26-Aug-20	MoH	N	N	N	N	N	N	14.88			31-Dec-20	04-Jan-21		

C19IMPACT_0061	Trinidad and Tobago	09-Sept-20	MoH	Y	N	Y	N	Y	N	15.06			31-Dec-20	04-Jan-21		
C19IMPACT_0062	Trinidad and Tobago	09-Sept-20	MoH	Y	N	Y	N	Y	N	15.06			31-Dec-20	04-Jan-21		
C19IMPACT_0063	Trinidad and Tobago	27-Oct-20	CARPHA	N	N	N	N	N	N	13.45		20-Dec-20	31-Dec-20	04-Jan-21	31-Dec-20	
C19IMPACT_0064	Antigua and Barbuda	28-Oct-20	CARPHA	N	N	N	N	N	N	26.49		28-Dec-20	31-Dec-20		02-Jan-21	
C19IMPACT_0065	Antigua and Barbuda	29-Oct-20	CARPHA	N	N	N	N	N	N	17.16		28-Dec-20	31-Dec-20		02-Jan-21	02-Jun-21
C19IMPACT_0066	Saint Vincent and the Grenadines	18-Nov-20	CARPHA	N	N	N	N	N	N	21.63		28-Dec-20	31-Dec-20		02-Jan-21	
C19IMPACT_0067	Trinidad and Tobago	18-Nov-20	CARPHA	N	N	N	N	Y	N	21.48		28-Dec-20	31-Dec-20	04-Jan-21	31-Dec-20	
C19IMPACT_0068	Trinidad and Tobago	26-Nov-20	CARPHA	N	N	N	N	Y	N	13.06		28-Dec-20	31-Dec-20	04-Jan-21	31-Dec-20	
C19IMPACT_0069	Trinidad and Tobago	11-Dec-20	CARPHA	N	N	N	N	N	N	13.72		28-Dec-20	31-Dec-20	04-Jan-21	31-Dec-20	
C19IMPACT_0070	Trinidad and Tobago	12-Dec-20	CARPHA	N	N	N	N	N	N	15.59		28-Dec-20	31-Dec-20	04-Jan-21	31-Dec-20	
C19IMPACT_0071	Trinidad and Tobago	17-Dec-20	CARPHA	N	N	N	N	Y	N	19.46		28-Dec-20	31-Dec-20	04-Jan-21	31-Dec-20	
C19IMPACT_0072	Saint Vincent and the Grenadines	18-Dec-20	CARPHA	N	N	N	N	N	N	25.52		28-Dec-20	31-Dec-20		02-Jan-21	
C19IMPACT_0073	Jamaica	21-Dec-20	CARPHA	N	N	N	N	N	N	25.10		30-Dec-20	31-Dec-20		02-Jan-21	
C19IMPACT_0074	Jamaica	21-Dec-20	CARPHA	N	N	N	N	N	Y	26.97		30-Dec-20	31-Dec-20		02-Jan-21	
C19IMPACT_0075	Jamaica	21-Dec-20	CARPHA	N	N	N	N	N	N	17.28		30-Dec-20	31-Dec-20		02-Jan-21	
C19IMPACT_0076	Jamaica	21-Dec-20	CARPHA	N	N	N	N	N	N	21.91		30-Dec-20	31-Dec-20		02-Jan-21	
C19IMPACT_0077	Jamaica	21-Dec-20	CARPHA	N	N	N	N	N	N	25.20		30-Dec-20	31-Dec-20		02-Jan-21	
C19IMPACT_0078	Trinidad and Tobago		MoH	N	N	N	N	N	Y				31-Dec-20		02-Jan-21	
C19IMPACT_0079	Trinidad and Tobago	13-Nov-20	MoH	N	N	N	N	N	Y			01-Dec-20	08-Jan-21			21-Jan-22
C19IMPACT_0080	Trinidad and Tobago	18-Nov-20	MoH	N	N	N	N	N	Y			01-Dec-20	08-Jan-21			
C19IMPACT_0081	Trinidad and Tobago	18-Nov-20	MoH	N	N	N	N	N	Y			01-Dec-20	08-Jan-21			21-Jan-22
C19IMPACT_0082	Trinidad and Tobago	18-Nov-20	MoH	N	N	N	N	N	Y			01-Dec-20	08-Jan-21			
C19IMPACT_0083	Trinidad and Tobago	18-Nov-20	MoH	N	N	N	N	N	Y			01-Dec-20	08-Jan-21			21-Jan-22
C19IMPACT_0084	Trinidad and Tobago	18-Nov-20	MoH	N	N	N	N	N	Y			01-Dec-20	08-Jan-21			21-Jan-22
C19IMPACT_0085	Trinidad and Tobago	18-Nov-20	MoH	N	N	N	N	N	Y			01-Dec-20	08-Jan-21			21-Jan-22
C19IMPACT_0086	Trinidad and Tobago	18-Nov-20	MoH	N	N	N	N	N	Y			01-Dec-20	08-Jan-21			21-Jan-22
C19IMPACT_0087	Trinidad and Tobago	20-Nov-20	MoH	N	N	N	N	N	Y			01-Dec-20	08-Jan-21			21-Jan-22
C19IMPACT_0088	Trinidad and Tobago	20-Nov-20	MoH	N	N	N	N	N	Y			01-Dec-20	08-Jan-21			21-Jan-22
C19IMPACT_0089	Trinidad and Tobago	20-Nov-20	MoH	N	N	N	N	N	Y			01-Dec-20	08-Jan-21			
C19IMPACT_0090	Trinidad and Tobago	20-Nov-20	MoH	N	N	N	N	N	Y			01-Dec-20	08-Jan-21			21-Jan-22
C19IMPACT_0091	Trinidad and Tobago	20-Nov-20	MoH	N	N	N	N	N	Y			01-Dec-20	08-Jan-21			21-Jan-22
C19IMPACT_0092	Trinidad and Tobago	20-Nov-20	MoH	N	N	N	N	N	Y			01-Dec-20	08-Jan-21			21-Jan-22
C19IMPACT_0093	Trinidad and Tobago	20-Nov-20	MoH	N	N	N	N	N	Y			01-Dec-20	08-Jan-21			21-Jan-22
C19IMPACT_0094	Trinidad and Tobago	20-Nov-20	MoH	N	N	N	N	N	Y			01-Dec-20	08-Jan-21			
C19IMPACT_0095	Trinidad and Tobago	20-Nov-20	MoH	N	N	N	N	N	Y			01-Dec-20	08-Jan-21			
C19IMPACT_0096	Trinidad and Tobago		MoH	N	N	N	N	N	Y			01-Dec-20	08-Jan-21			
C19IMPACT_0097	Trinidad and Tobago		MoH	N	N	N	N	N	Y			01-Dec-20	08-Jan-21			
C19IMPACT_0098	Trinidad and Tobago		MoH	N	N	N	N	N	Y			01-Dec-20	08-Jan-21			
C19IMPACT_0099	Trinidad and Tobago		MoH	N	N	N	N	N	Y			01-Dec-20	08-Jan-21			
C19IMPACT_0100	Trinidad and Tobago		MoH	N	N	N	N	N	Y			01-Dec-20	08-Jan-21			
C19IMPACT_0101	Trinidad and Tobago		MoH	N	N	N	N	N	Y			01-Dec-20	08-Jan-21			
C19IMPACT_0102	tbc	15-Aug-20	CARPHA	N	N	N	N	N	N	22.94		24-Dec-20	19-Jan-21		22-Jan-21	
C19IMPACT_0103	Trinidad and Tobago	29-Aug-20	MoH	N	N	N	N	N	N				19-Jan-21			21-Jan-22
C19IMPACT_0104	Trinidad and Tobago	30-Aug-20	MoH	Y	N	Y	N	Y	N	26.65			19-Jan-21			21-Jan-22
C19IMPACT_0105	Trinidad and Tobago	14-Sept-20	MoH	N	N	N	N	N	N			01-Dec-20	19-Jan-21			21-Jan-22
C19IMPACT_0106	Trinidad and Tobago	14-Sept-20	MoH	N	N	N	N	N	N			01-Dec-20	19-Jan-21			21-Jan-22
C19IMPACT_0107	Trinidad and Tobago	14-Sept-20	MoH	N	N	N	N	N	N			01-Dec-20	19-Jan-21			21-Jan-22
C19IMPACT_0108	tbc	27-Oct-20	CARPHA	N	N	N	N	N	N	30.68		24-Dec-20	19-Jan-21		22-Jan-21	
C19IMPACT_0109	Trinidad and Tobago	04-Dec-20	MoH	N	N	N	N	N	N			01-Dec-20	19-Jan-21			21-Jan-22
C19IMPACT_0110	Trinidad and Tobago	04-Dec-20	MoH	N	N	N	N	N	N			01-Dec-20	19-Jan-21			21-Jan-22
C19IMPACT_0111	Jamaica	21-Dec-20	CARPHA	N	N	N	N	N	Y	20.50		30-Dec-20	19-Jan-21		02-Jan-21	21-Jan-22
C19IMPACT_0112	Trinidad and Tobago		MoH	N	N	N	N	N	N			01-Dec-20	19-Jan-21			
C19IMPACT_0113	Trinidad and Tobago		CARPHA	N	N	N	N	N	N			18-Jan-21	19-Jan-21	21-Jan-21	22-Jan-21	
C19IMPACT_0114	tbc		CARPHA	N	N	N	N	N	N	24.07		24-Dec-20	19-Jan-21		22-Jan-21	
C19IMPACT_0115	tbc		CARPHA	N	N	N	N	N	N	21.78		24-Dec-20	19-Jan-21		22-Jan-21	
C19IMPACT_0116	tbc		CARPHA	N	N	N	N	N	N	17.29		24-Dec-20	19-Jan-21		22-Jan-21	
C19IMPACT_0117	tbc		CARPHA	N	N	N	N	N	N	19.79		24-Dec-20	19-Jan-21		22-Jan-21	
C19IMPACT_0118	tbc		CARPHA	N	N	N	N	N	N	22.60		24-Dec-20	19-Jan-21		22-Jan-21	
C19IMPACT_0119	tbc		CARPHA	N	N	N	N	N	N	17.92		24-Dec-20	19-Jan-21		22-Jan-21	
C19IMPACT_0120	tbc		CARPHA	N	N	N	N	N	N	19.92		24-Dec-20	19-Jan-21		22-Jan-21	
C19IMPACT_0121	tbc		CARPHA	N	N	N	N	N	N	22.89		24-Dec-20	19-Jan-21		22-Jan-21	
C19IMPACT_0122	tbc		CARPHA	N	N	N	N	N	N	29.14		24-Dec-20	19-Jan-21		22-Jan-21	
C19IMPACT_0123	tbc		CARPHA	N	N	N	N	N	N	34.76		24-Dec-20	19-Jan-21		22-Jan-21	
C19IMPACT_0124	tbc		CARPHA	N	N	N	N	N	N	21.35		24-Dec-20	19-Jan-21		22-Jan-21	

C19IMPACT_0125	Trinidad and Tobago		MoH	N	N	N	N	N	N			01-Dec-20	21-Jan-21			
C19IMPACT_0126	Trinidad and Tobago		MoH	N	N	N	N	N	N			01-Dec-20	21-Jan-21			
C19IMPACT_0127	Trinidad and Tobago		MoH	N	N	N	N	N	Y			01-Dec-20	21-Jan-21	29-Jan-21		
C19IMPACT_0128	Trinidad and Tobago	31-Aug-20	MoH	N	N	N	N	N	N	23.02			21-Jan-21			21-Jan-22
C19IMPACT_0129	Cayman Islands	09-Dec-20	CARPHA	N	N	N	N	N	N	22.33		21-Jan-21	21-Jan-21		22-Jan-21	
C19IMPACT_0130	Cayman Islands	11-Dec-20	CARPHA	N	N	N	N	N	N	15.43		21-Jan-21	21-Jan-21		22-Jan-21	21-Jan-22
C19IMPACT_0131	Cayman Islands	15-Dec-20	CARPHA	N	N	N	N	N	N	20.31		21-Jan-21	21-Jan-21		22-Jan-21	21-Jan-22
C19IMPACT_0132	Cayman Islands	15-Dec-20	CARPHA	N	N	N	N	N	N	22.86		21-Jan-21	21-Jan-21		22-Jan-21	
C19IMPACT_0133	Cayman Islands	15-Dec-20	CARPHA	N	N	N	N	N	N	14.82		21-Jan-21	21-Jan-21		22-Jan-21	21-Jan-22
C19IMPACT_0134	Saint Lucia	17-Dec-20	CARPHA	N	N	N	N	N	N	14.51		21-Jan-21	21-Jan-21		22-Jan-21	
C19IMPACT_0135	Saint Lucia	23-Dec-20	CARPHA	N	N	N	N	N	N	16.18		21-Jan-21	21-Jan-21		22-Jan-21	
C19IMPACT_0136	Saint Lucia	23-Dec-20	CARPHA	N	N	N	N	N	N	19.65		21-Jan-21	21-Jan-21		22-Jan-21	21-Jan-22
C19IMPACT_0137	Saint Lucia	23-Dec-20	CARPHA	N	N	N	N	N	N	20.53		21-Jan-21	21-Jan-21		22-Jan-21	21-Jan-22
C19IMPACT_0138	Saint Lucia	23-Dec-20	CARPHA	N	N	N	N	N	N	21.34		21-Jan-21	21-Jan-21		22-Jan-21	
C19IMPACT_0139	Barbados	24-Dec-20	CARPHA	N	N	N	N	N	N	18.98		21-Jan-21	21-Jan-21		22-Jan-21	
C19IMPACT_0140	Saint Lucia	26-Dec-20	CARPHA	N	N	N	N	N	N	13.98		21-Jan-21	21-Jan-21		22-Jan-21	
C19IMPACT_0141	Barbados	30-Dec-20	CARPHA	N	N	N	N	N	N	19.69		21-Jan-21	21-Jan-21		22-Jan-21	
C19IMPACT_0142	Barbados	30-Dec-20	CARPHA	N	N	N	N	N	N	24.50		21-Jan-21	21-Jan-21		22-Jan-21	
C19IMPACT_0143	Barbados	31-Dec-20	CARPHA	N	N	N	N	N	N	20.35		21-Jan-21	21-Jan-21		22-Jan-21	
C19IMPACT_0144	Barbados	31-Dec-20	CARPHA	N	N	N	N	N	N	20.45		21-Jan-21	21-Jan-21		22-Jan-21	21-Jan-22
C19IMPACT_0145	Barbados	02-Jan-21	CARPHA	N	N	N	N	N	N	22.65		21-Jan-21	21-Jan-21		22-Jan-21	21-Jan-22
C19IMPACT_0146	Barbados	03-Jan-21	CARPHA	N	N	N	N	N	N	14.70		21-Jan-21	21-Jan-21		22-Jan-21	
C19IMPACT_0147	Cayman Islands	03-Jan-21	CARPHA	N	N	N	N	N	N	16.01		21-Jan-21	21-Jan-21		22-Jan-21	21-Jan-22
C19IMPACT_0148	British Virgin Islands	03-Jan-21	CARPHA	N	N	N	N	N	N	16.12		22-Jan-21	23-Jan-21		25-Jan-21	
C19IMPACT_0149	British Virgin Islands	03-Jan-21	CARPHA	N	N	N	N	N	N	24.35		22-Jan-21	23-Jan-21		25-Jan-21	21-Jan-22
C19IMPACT_0150	British Virgin Islands	05-Jan-21	CARPHA	N	N	N	N	N	N	24.31		22-Jan-21	23-Jan-21		25-Jan-21	
C19IMPACT_0151	British Virgin Islands	05-Jan-21	CARPHA	N	N	N	N	N	N	15.05		22-Jan-21	23-Jan-21		25-Jan-21	
C19IMPACT_0152	British Virgin Islands	06-Jan-21	CARPHA	N	N	N	N	N	N	21.93		22-Jan-21	23-Jan-21		25-Jan-21	
C19IMPACT_0153	British Virgin Islands	07-Jan-21	CARPHA	N	N	N	N	N	N	16.43		22-Jan-21	23-Jan-21		25-Jan-21	
C19IMPACT_0154	British Virgin Islands	10-Jan-21	CARPHA	N	N	N	N	N	N	25.15		22-Jan-21	23-Jan-21		25-Jan-21	
C19IMPACT_0155	Trinidad and Tobago	18-Jan-21	MoH	N	N	N	N	N	N			22-Jan-21	24-Jan-21	29-Jan-21		
C19IMPACT_0156	Guyana	27-Mar-20	CARPHA	N	N	N	N	N	N	17.99		26-Jan-21	27-Jan-21		28-Jan-21	23-May-21
C19IMPACT_0157	Guyana	07-Sept-20	CARPHA	N	N	N	N	N	N	12.35		26-Jan-21	27-Jan-21		28-Jan-21	11-Jun-21
C19IMPACT_0158	Trinidad and Tobago	07-Sept-20	MoH	N	N	N	N	Y	N			01-Dec-20	27-Jan-21	29-Jan-21		23-May-21
C19IMPACT_0159	Guyana	11-Sept-20	CARPHA	N	N	N	N	N	N	24.97		26-Jan-21	27-Jan-21		28-Jan-21	11-Jun-21
C19IMPACT_0160	Guyana	11-Sept-20	CARPHA	N	N	N	N	N	N	16.06		26-Jan-21	27-Jan-21		28-Jan-21	23-May-21
C19IMPACT_0161	Guyana	11-Sept-20	CARPHA	N	N	N	N	N	N	11.37		26-Jan-21	27-Jan-21		28-Jan-21	11-Jun-21
C19IMPACT_0162	Trinidad and Tobago	20-Nov-20	MoH	N	N	N	N	Y	N			01-Dec-20	27-Jan-21	29-Jan-21		23-May-21
C19IMPACT_0163	Guyana	29-Dec-20	CARPHA	N	N	N	N	N	N	15.75		26-Jan-21	27-Jan-21		28-Jan-21	11-Jun-21
C19IMPACT_0164	Guyana	01-Jan-21	CARPHA	N	N	N	N	N	N	21.83		26-Jan-21	27-Jan-21		28-Jan-21	11-Jun-21
C19IMPACT_0165	Guyana	03-Jan-21	CARPHA	N	N	N	N	N	N	18.29		26-Jan-21	27-Jan-21		28-Jan-21	11-Jun-21
C19IMPACT_0166	Grenada	06-Jan-21	CARPHA	N	N	N	N	N	N	24.29		26-Jan-21	27-Jan-21		28-Jan-21	11-Jun-21
C19IMPACT_0167	Grenada	06-Jan-21	CARPHA	N	N	N	N	N	N	22.36		26-Jan-21	27-Jan-21		28-Jan-21	11-Jun-21
C19IMPACT_0168	Grenada	06-Jan-21	CARPHA	N	N	N	N	N	N	21.22		26-Jan-21	27-Jan-21		28-Jan-21	11-Jun-21
C19IMPACT_0169	Grenada	06-Jan-21	CARPHA	N	N	N	N	N	N	20.90		26-Jan-21	27-Jan-21		28-Jan-21	11-Jun-21
C19IMPACT_0170	Grenada	17-Jan-21	CARPHA	N	N	N	N	N	N	15.97		26-Jan-21	27-Jan-21		28-Jan-21	11-Jun-21
C19IMPACT_0171	Guyana	18-Jan-21	CARPHA	N	N	N	N	N	N	12.18		26-Jan-21	27-Jan-21		28-Jan-21	11-Jun-21
C19IMPACT_0172	Guyana	18-Jan-21	CARPHA	N	N	N	N	N	N	22.49		26-Jan-21	27-Jan-21		28-Jan-21	11-Jun-21
C19IMPACT_0173	Trinidad and Tobago		MoH	N	N	N	N	N	N			01-Dec-20	27-Jan-21	29-Jan-21		
C19IMPACT_0174	Trinidad and Tobago		MoH	N	N	N	N	N	N			01-Dec-20	27-Jan-21	29-Jan-21		
C19IMPACT_0175	Trinidad and Tobago		MoH	N	N	N	N	N	N			01-Dec-20	27-Jan-21	29-Jan-21		
C19IMPACT_0176	Trinidad and Tobago		MoH	N	N	N	N	N	N			01-Dec-20	27-Jan-21	29-Jan-21		
C19IMPACT_0177	Trinidad and Tobago		MoH	N	N	N	N	N	N			01-Dec-20	27-Jan-21	29-Jan-21		
C19IMPACT_0178	Trinidad and Tobago		MoH	N	N	N	N	N	N			01-Dec-20	27-Jan-21	29-Jan-21		
C19IMPACT_0179	Trinidad and Tobago	05-Aug-20	MoH	N	N	N	N	Y	N	27.02			28-Jan-21	29-Jan-21		21-Jan-22
C19IMPACT_0180	Saint Lucia	13-Jan-21	CARPHA	N	N	N	N	N	N	13.13		27-Jan-21	28-Jan-21		29-Jan-21	
C19IMPACT_0181	Saint Lucia	14-Jan-21	CARPHA	N	N	N	N	N	N	13.62		27-Jan-21	28-Jan-21		29-Jan-21	
C19IMPACT_0182	Saint Lucia	14-Jan-21	CARPHA	N	N	N	N	N	N	13.45		27-Jan-21	28-Jan-21		29-Jan-21	
C19IMPACT_0183	Saint Lucia	16-Jan-21	CARPHA	N	N	N	N	N	N	14.94		27-Jan-21	28-Jan-21		29-Jan-21	
C19IMPACT_0184	Saint Lucia	18-Jan-21	CARPHA	N	N	N	N	N	N	17.25		27-Jan-21	28-Jan-21		29-Jan-21	
C19IMPACT_0185	Saint Lucia	18-Jan-21	CARPHA	N	N	N	N	N	N	14.33		27-Jan-21	28-Jan-21		29-Jan-21	
C19IMPACT_0186	Saint Lucia	18-Jan-21	CARPHA	N	N	N	N	N	N	14.50		27-Jan-21	28-Jan-21		29-Jan-21	21-Jan-22
C19IMPACT_0187	Saint Lucia	18-Jan-21	CARPHA	N	N	N	N	N	N	16.59		27-Jan-21	28-Jan-21		29-Jan-21	21-Jan-22
C19IMPACT_0188	Saint Lucia	18-Jan-21	CARPHA	N	N	N	N	N	N	15.39		27-Jan-21	28-Jan-21		29-Jan-21	21-Jan-22

C19IMPACT_0189	Saint Lucia	18-Jan-21	CARPHA	N	N	N	N	N	N	16.00		27-Jan-21	28-Jan-21		29-Jan-21	21-Jan-22
C19IMPACT_0190	Trinidad and Tobago	25-Jan-21	MoH	N	N	N	N	N	N			01-Jan-20	28-Jan-21	29-Jan-21		21-Jan-22
C19IMPACT_0191	Trinidad and Tobago	25-Jan-21	MoH	N	N	N	N	N	N			01-Jan-20	28-Jan-21	29-Jan-21		21-Jan-22
C19IMPACT_0192	Trinidad and Tobago	25-Jan-21	MoH	N	N	N	N	N	N			01-Jan-20	28-Jan-21	29-Jan-21		21-Jan-22
C19IMPACT_0193	Trinidad and Tobago	25-Jan-21	MoH	N	N	N	N	N	N			01-Jan-20	28-Jan-21	29-Jan-21		21-Jan-22
C19IMPACT_0194	Trinidad and Tobago	25-Jan-21	MoH	N	N	N	N	N	N			01-Jan-20	28-Jan-21	29-Jan-21		21-Jan-22
C19IMPACT_0195	Trinidad and Tobago	25-Jan-21	MoH	N	N	N	N	N	N			01-Jan-20	28-Jan-21	13-Oct-21		21-Jan-22
C19IMPACT_0196	Trinidad and Tobago	25-Jan-21	MoH	N	N	N	N	N	N			01-Jan-20	28-Jan-21	29-Jan-21		21-Jan-22
C19IMPACT_0197	Trinidad and Tobago		MoH	N	N	N	N	N	N	19.31			28-Jan-21	29-Jan-21		
C19IMPACT_0198	Bermuda	12-Nov-20	CARPHA	N	N	N	N	N	Y	16.38		03-Feb-21	17-Feb-21		28-Feb-21	04-Jun-21
C19IMPACT_0199	Bermuda	30-Nov-20	CARPHA	N	N	N	N	N	Y	18.94		03-Feb-21	17-Feb-21		28-Feb-21	04-Jun-21
C19IMPACT_0200	Bermuda	06-Dec-20	CARPHA	N	N	N	N	N	Y	14.36		03-Feb-21	17-Feb-21		28-Feb-21	04-Jun-21
C19IMPACT_0201	Bermuda	12-Dec-20	CARPHA	N	N	N	N	N	Y	16.74		03-Feb-21	17-Feb-21		28-Feb-21	04-Jun-21
C19IMPACT_0202	Bermuda	14-Dec-20	CARPHA	N	N	N	N	N	Y	16.07		03-Feb-21	17-Feb-21		28-Feb-21	04-Jun-21
C19IMPACT_0203	Bermuda	19-Dec-20	CARPHA	N	N	N	N	N	Y	13.69		03-Feb-21	17-Feb-21		28-Feb-21	04-Jun-21
C19IMPACT_0204	Bermuda	23-Dec-20	CARPHA	N	N	N	N	N	Y	15.45		03-Feb-21	17-Feb-21		28-Feb-21	04-Jun-21
C19IMPACT_0205	Bermuda	26-Dec-20	CARPHA	N	N	N	N	N	Y	17.35		03-Feb-21	17-Feb-21		28-Feb-21	04-Jun-21
C19IMPACT_0206	Bermuda	05-Jan-21	CARPHA	N	N	N	N	N	Y	16.72		03-Feb-21	17-Feb-21		28-Feb-21	04-Jun-21
C19IMPACT_0207	Bermuda	12-Jan-21	CARPHA	N	N	N	N	N	Y	22.20		03-Feb-21	17-Feb-21		28-Feb-21	04-Jun-21
C19IMPACT_0208	British Virgin Islands	12-Jan-21	CARPHA	N	N	N	N	N	Y	15.49		05-Feb-21	17-Feb-21		28-Feb-21	04-Jun-21
C19IMPACT_0209	British Virgin Islands	13-Jan-21	CARPHA	N	N	N	N	N	Y	16.52		05-Feb-21	17-Feb-21		28-Feb-21	04-Jun-21
C19IMPACT_0210	British Virgin Islands	19-Jan-21	CARPHA	N	N	N	N	N	Y	14.79		05-Feb-21	17-Feb-21		28-Feb-21	04-Jun-21
C19IMPACT_0211	Antigua and Barbuda	21-Jan-21	CARPHA	N	N	N	N	N	Y	22.03		03-Feb-21	17-Feb-21		28-Feb-21	
C19IMPACT_0212	British Virgin Islands	23-Jan-21	CARPHA	N	N	N	N	N	Y	21.18		05-Feb-21	17-Feb-21		28-Feb-21	04-Jun-21
C19IMPACT_0213	British Virgin Islands	25-Jan-21	CARPHA	N	N	N	N	N	Y	16.85		05-Feb-21	17-Feb-21		28-Feb-21	04-Jun-21
C19IMPACT_0214	Antigua and Barbuda	26-Jan-21	CARPHA	N	N	N	N	N	Y	15.85		03-Feb-21	17-Feb-21		28-Feb-21	04-Jun-21
C19IMPACT_0215	Saint Vincent and the Grenadines	27-Jan-21	CARPHA	N	N	N	N	N	Y	21.58		05-Feb-21	17-Feb-21		28-Feb-21	04-Jun-21
C19IMPACT_0216	Saint Vincent and the Grenadines	29-Jan-21	CARPHA	N	N	N	N	N	Y	19.35		05-Feb-21	17-Feb-21		28-Feb-21	04-Jun-21
C19IMPACT_0217	Saint Vincent and the Grenadines	29-Jan-21	CARPHA	N	N	N	N	N	Y	18.24		05-Feb-21	17-Feb-21		28-Feb-21	04-Jun-21
C19IMPACT_0218	Saint Vincent and the Grenadines	29-Jan-21	CARPHA	N	N	N	N	N	Y	17.60		05-Feb-21	17-Feb-21		28-Feb-21	04-Jun-21
C19IMPACT_0219	Trinidad and Tobago	07-Jan-21	MoH	N	N	N	N	N	N		3.46	19-Jan-21	19-Feb-21	26-Feb-21		
C19IMPACT_0220	Trinidad and Tobago	07-Jan-21	MoH	N	N	N	N	N	N		6.42	19-Jan-21	19-Feb-21	26-Feb-21		
C19IMPACT_0221	Trinidad and Tobago	13-Jan-21	MoH	N	N	N	N	N	N		11.38	19-Jan-21	19-Feb-21	26-Feb-21		
C19IMPACT_0222	Trinidad and Tobago	15-Jan-21	MoH	N	N	N	N	N	N		5.00	19-Jan-21	19-Feb-21	26-Feb-21		
C19IMPACT_0223	Trinidad and Tobago	17-Jan-21	MoH	N	N	N	N	N	N		14.00	19-Jan-21	19-Feb-21	26-Feb-21		
C19IMPACT_0224	Trinidad and Tobago	20-Jan-21	MoH	N	N	N	N	N	N		11.38	19-Jan-21	19-Feb-21	26-Feb-21		
C19IMPACT_0225	Turks and Caicos Islands	21-Jan-21	CARPHA	N	N	N	N	N	N	19.09		19-Jan-21	19-Feb-21		25-Feb-21	21-Jan-22
C19IMPACT_0226	Turks and Caicos Islands	21-Jan-21	CARPHA	N	N	N	N	N	N	16.48		19-Jan-21	19-Feb-21		25-Feb-21	21-Jan-22
C19IMPACT_0227	Turks and Caicos Islands	21-Jan-21	CARPHA	N	N	N	N	N	N	21.59		19-Jan-21	19-Feb-21		25-Feb-21	
C19IMPACT_0228	Turks and Caicos Islands	22-Jan-21	CARPHA	N	N	N	N	N	N	19.44		19-Jan-21	19-Feb-21		25-Feb-21	
C19IMPACT_0229	Trinidad and Tobago	29-Jan-21	MoH	N	N	N	N	N	N		4.48	19-Jan-21	19-Feb-21	26-Feb-21		
C19IMPACT_0230	Trinidad and Tobago	05-Feb-21	CARPHA	N	N	N	N	Y	Y			18-Feb-21	19-Feb-21	05-Mar-21	25-Feb-21	21-Jan-22
C19IMPACT_0231	Trinidad and Tobago		CARPHA	N	N	N	N	N	Y			18-Feb-21	19-Feb-21	05-Mar-21	25-Feb-21	
C19IMPACT_0232	Trinidad and Tobago		CARPHA	N	N	N	N	N	Y			18-Feb-21	19-Feb-21	05-Mar-21	25-Feb-21	
C19IMPACT_0233	Trinidad and Tobago		CARPHA	N	N	N	N	N	Y			18-Feb-21	19-Feb-21	05-Mar-21	25-Feb-21	
C19IMPACT_0234	Trinidad and Tobago		CARPHA	N	N	N	N	N	Y			18-Feb-21	19-Feb-21	05-Mar-21	25-Feb-21	
C19IMPACT_0235	Trinidad and Tobago		CARPHA	N	N	N	N	N	Y			18-Feb-21	19-Feb-21	05-Mar-21	25-Feb-21	
C19IMPACT_0236	Trinidad and Tobago	02-Mar-21	MoH	N	N	N	N	N	Y			12-Feb-21	25-Feb-21	05-Mar-21		21-Jan-22
C19IMPACT_0237	Trinidad and Tobago	07-May-21	MoH	N	N	N	N	N	Y			18-Feb-21	25-Feb-21	05-Mar-21		21-Jan-22
C19IMPACT_0238	Trinidad and Tobago	29-Jun-21	MoH	N	N	N	N	N	Y			18-Feb-21	25-Feb-21	05-Mar-21	02-Mar-21	
C19IMPACT_0239	Trinidad and Tobago		MoH	N	N	N	N	N	Y			18-Feb-21	25-Feb-21	05-Mar-21		
C19IMPACT_0240	Trinidad and Tobago		MoH	N	N	N	N	N	Y			18-Feb-21	25-Feb-21	05-Mar-21		
C19IMPACT_0241	Trinidad and Tobago		MoH	N	N	N	N	N	Y			18-Feb-21	25-Feb-21	05-Mar-21		
C19IMPACT_0242	Trinidad and Tobago		MoH	N	N	N	N	N	Y			18-Feb-21	25-Feb-21	05-Mar-21		
C19IMPACT_0243	Trinidad and Tobago		MoH	N	N	N	N	N	Y			18-Feb-21	25-Feb-21	05-Mar-21		
C19IMPACT_0244	Trinidad and Tobago		MoH	N	N	N	N	N	Y			18-Feb-21	25-Feb-21	05-Mar-21		
C19IMPACT_0245	Trinidad and Tobago		MoH	N	N	N	N	N	Y			18-Feb-21	25-Feb-21	05-Mar-21		
C19IMPACT_0246	Trinidad and Tobago		MoH	N	N	N	N	N	Y			18-Feb-21	25-Feb-21	05-Mar-21		
C19IMPACT_0247	Trinidad and Tobago		MoH	N	N	N	N	N	Y			18-Feb-21	25-Feb-21	05-Mar-21		
C19IMPACT_0248	Trinidad and Tobago		MoH	N	N	N	N	N	Y			18-Feb-21	25-Feb-21	05-Mar-21		
C19IMPACT_0249	Trinidad and Tobago		MoH	N	N	N	N	N	Y			18-Feb-21	25-Feb-21	05-Mar-21		
C19IMPACT_0250	Trinidad and Tobago		MoH	N	N	N	N	N	Y			18-Feb-21	25-Feb-21	05-Mar-21		
C19IMPACT_0251	Trinidad and Tobago		MoH	N	N	N	N	N	Y			18-Feb-21	25-Feb-21	05-Mar-21		
C19IMPACT_0252	Trinidad and Tobago		MoH	N	N	N	N	N	Y			18-Feb-21	25-Feb-21	05-Mar-21		

C19IMPACT_0253	Trinidad and Tobago		MoH	N	N	N	N	N	Y			18-Feb-21	25-Feb-21	05-Mar-21		
C19IMPACT_0254	Trinidad and Tobago		MoH	N	N	N	N	N	Y			18-Feb-21	25-Feb-21	05-Mar-21		
C19IMPACT_0255	Trinidad and Tobago		MoH	N	N	N	N	N	Y			18-Feb-21	25-Feb-21	05-Mar-21		
C19IMPACT_0256	Trinidad and Tobago		MoH	N	N	N	N	N	Y			18-Feb-21	25-Feb-21	05-Mar-21		
C19IMPACT_0257	Trinidad and Tobago		MoH	N	N	N	N	N	Y			18-Feb-21	25-Feb-21	05-Mar-21		
C19IMPACT_0258	Dominica	16-Dec-20	CARPHA	N	N	N	N	N	Y	25.52		01-Mar-21	03-Mar-21		05-Mar-21	23-Jun-21
C19IMPACT_0259	Dominica	02-Jan-21	CARPHA	N	N	N	N	N	Y	24.32		01-Mar-21	03-Mar-21		05-Mar-21	23-Jun-21
C19IMPACT_0260	Dominica	02-Jan-21	CARPHA	N	N	N	N	N	Y	22.82		01-Mar-21	03-Mar-21		05-Mar-21	23-Jun-21
C19IMPACT_0261	Jamaica	21-Jan-21	CARPHA	N	N	N	N	N	Y	16.39		01-Mar-21	03-Mar-21		05-Mar-21	23-Jun-21
C19IMPACT_0262	Jamaica	21-Jan-21	CARPHA	N	N	N	N	N	Y	13.95		01-Mar-21	03-Mar-21		05-Mar-21	23-Jun-21
C19IMPACT_0263	Jamaica	21-Jan-21	CARPHA	N	N	N	N	N	Y	23.14		01-Mar-21	03-Mar-21		05-Mar-21	23-Jun-21
C19IMPACT_0264	Dominica	22-Jan-21	CARPHA	N	N	N	N	N	Y	22.84		01-Mar-21	03-Mar-21		05-Mar-21	23-Jun-21
C19IMPACT_0265	Dominica	22-Jan-21	CARPHA	N	N	N	N	N	Y	17.81		01-Mar-21	03-Mar-21		05-Mar-21	23-Jun-21
C19IMPACT_0266	Jamaica	23-Jan-21	CARPHA	N	N	N	N	N	Y	21.47		01-Mar-21	03-Mar-21		05-Mar-21	23-Jun-21
C19IMPACT_0267	Jamaica	23-Jan-21	CARPHA	N	N	N	N	N	Y	14.97		01-Mar-21	03-Mar-21		05-Mar-21	23-Jun-21
C19IMPACT_0268	Jamaica	24-Jan-21	CARPHA	N	N	N	N	N	Y	17.87		01-Mar-21	03-Mar-21		05-Mar-21	23-Jun-21
C19IMPACT_0269	Jamaica	27-Jan-21	CARPHA	N	N	N	N	N	Y	24.23		01-Mar-21	03-Mar-21		05-Mar-21	
C19IMPACT_0270	Jamaica	28-Jan-21	CARPHA	N	N	N	N	N	Y	19.11		01-Mar-21	03-Mar-21		05-Mar-21	23-Jun-21
C19IMPACT_0271	Saint Vincent and the Grenadines	28-Jan-21	CARPHA	N	N	N	N	N	Y	13.45		01-Mar-21	03-Mar-21		05-Mar-21	23-Jun-21
C19IMPACT_0272	Dominica	29-Jan-21	CARPHA	N	N	N	N	N	Y	19.50		01-Mar-21	03-Mar-21		05-Mar-21	23-Jun-21
C19IMPACT_0273	Saint Vincent and the Grenadines	29-Jan-21	CARPHA	N	N	N	N	N	Y	17.82		01-Mar-21	03-Mar-21		05-Mar-21	23-Jun-21
C19IMPACT_0274	Saint Vincent and the Grenadines	30-Jan-21	CARPHA	N	N	N	N	N	Y	18.57		01-Mar-21	03-Mar-21		05-Mar-21	23-Jun-21
C19IMPACT_0275	Trinidad and Tobago	17-Feb-21	MoH	N	N	N	N	N	Y	25.00		24-Feb-21	03-Mar-21	05-Mar-21		23-Jun-21
C19IMPACT_0276	Trinidad and Tobago	19-Feb-21	MoH	N	Y	N	N	Y	Y	18.70		24-Feb-21	03-Mar-21	05-Mar-21		23-Jun-21
C19IMPACT_0277	Trinidad and Tobago	19-Feb-21	MoH	N	N	N	N	N	Y				03-Mar-21	05-Mar-21		21-Jan-22
C19IMPACT_0278	Trinidad and Tobago	19-Feb-21	MoH	N	N	N	N	N	Y				03-Mar-21	05-Mar-21		21-Jan-22
C19IMPACT_0279	Trinidad and Tobago	08-Mar-21	MoH	N	N	N	N	N	Y	7.18		24-Feb-21	03-Mar-21	05-Mar-21		21-Jan-22
C19IMPACT_0280	Trinidad and Tobago	08-Mar-21	MoH	N	N	N	N	Y	N		6.49	16-Mar-21	18-Mar-21	19-Mar-21		21-Jan-22
C19IMPACT_0281	Montserrat	09-Mar-21	CARPHA	N	N	N	N	N	N	20.33		16-Mar-21	18-Mar-21		19-Mar-21	
C19IMPACT_0282	Trinidad and Tobago	17-Mar-21	MoH	N	N	N	N	N	N		9.02	16-Mar-21	18-Mar-21	19-Mar-21		
C19IMPACT_0283	Trinidad and Tobago	17-Mar-21	CARPHA	N	N	N	N	Y	N	17.28		16-Mar-21	18-Mar-21	19-Mar-21	19-Mar-21	
C19IMPACT_0284	Grenada	12-Mar-21	CARPHA	N	N	N	N	N	Y	19.47		18-Mar-21	25-Mar-21		26-Mar-21	14-Jun-21
C19IMPACT_0285	Barbados	16-Mar-21	CARPHA	N	N	N	N	N	Y	17.18		23-Mar-21	25-Mar-21		26-Mar-21	14-Jun-21
C19IMPACT_0286	Barbados	16-Mar-21	CARPHA	N	N	N	N	N	Y	19.86		23-Mar-21	25-Mar-21		26-Mar-21	
C19IMPACT_0287	Barbados	16-Mar-21	CARPHA	N	N	N	N	N	Y	23.60		23-Mar-21	25-Mar-21		26-Mar-21	14-Jun-21
C19IMPACT_0288	Barbados	16-Mar-21	CARPHA	N	N	N	N	N	Y	18.91		23-Mar-21	25-Mar-21		26-Mar-21	14-Jun-21
C19IMPACT_0289	Barbados	16-Mar-21	CARPHA	N	N	N	N	N	Y	17.34		23-Mar-21	25-Mar-21		26-Mar-21	14-Jun-21
C19IMPACT_0290	Trinidad and Tobago	18-Mar-21	CARPHA	N	N	N	N	N	Y	16.68		23-Mar-21	25-Mar-21	26-Mar-21	26-Mar-21	
C19IMPACT_0291	Trinidad and Tobago	18-Mar-21	CARPHA	N	N	N	N	N	Y	22.86		23-Mar-21	25-Mar-21	26-Mar-21	26-Mar-21	
C19IMPACT_0292	Trinidad and Tobago	19-Mar-21	CARPHA	N	N	N	N	Y	Y	18.88		23-Mar-21	25-Mar-21	26-Mar-21	26-Mar-21	
C19IMPACT_0293	Barbados	31-Mar-21	CARPHA	N	N	N	N	N	Y	22.68		23-Mar-21	25-Mar-21		26-Mar-21	14-Jun-21
C19IMPACT_0294	Barbados	26-Apr-21	CARPHA	N	N	N	N	N	Y	17.89		23-Mar-21	25-Mar-21		26-Mar-21	14-Jun-21
C19IMPACT_0295	Saint Lucia	07-Feb-21	CARPHA	N	N	N	N	N	Y	17.84		24-Mar-21	27-Mar-21		29-Mar-21	04-Jun-21
C19IMPACT_0296	Saint Lucia	24-Feb-21	CARPHA	N	N	N	N	N	Y	21.40		24-Mar-21	27-Mar-21		29-Mar-21	04-Jun-21
C19IMPACT_0297	Saint Lucia	12-Mar-21	CARPHA	N	N	N	N	N	Y	23.97		24-Mar-21	27-Mar-21		29-Mar-21	04-Jun-21
C19IMPACT_0298	Saint Lucia	13-Mar-21	CARPHA	N	N	N	N	N	Y	17.96		24-Mar-21	27-Mar-21		29-Mar-21	04-Jun-21
C19IMPACT_0299	Saint Lucia	14-Mar-21	CARPHA	N	N	N	N	N	Y	21.38		24-Mar-21	27-Mar-21		29-Mar-21	04-Jun-21
C19IMPACT_0300	Saint Lucia	15-Mar-21	CARPHA	N	N	N	N	N	Y	20.13		24-Mar-21	27-Mar-21		29-Mar-21	04-Jun-21
C19IMPACT_0301	Saint Lucia	16-Mar-21	CARPHA	N	N	N	N	N	Y	19.00		24-Mar-21	27-Mar-21		29-Mar-21	04-Jun-21
C19IMPACT_0302	Anguilla	18-Mar-21	CARPHA	N	N	N	N	N	Y	19.01		25-Mar-21	27-Mar-21		29-Mar-21	04-Jun-21
C19IMPACT_0303	Trinidad and Tobago	18-Mar-21	MoH	N	N	N	N	Y	Y	18.00		25-Mar-21	27-Mar-21	02-Apr-21		
C19IMPACT_0304	Anguilla	19-Mar-21	CARPHA	N	N	N	N	N	Y	23.17		25-Mar-21	27-Mar-21		29-Mar-21	04-Jun-21
C19IMPACT_0305	Anguilla	23-Mar-21	CARPHA	N	N	N	N	N	Y	15.72		25-Mar-21	27-Mar-21		29-Mar-21	04-Jun-21
C19IMPACT_0306	Trinidad and Tobago	24-Mar-21	MoH	N	N	N	N	N	Y	18.00		25-Mar-21	27-Mar-21	02-Apr-21		
C19IMPACT_0307	Trinidad and Tobago	09-Mar-21	CARPHA	N	N	N	N	N	Y	23.82		01-Apr-21	01-Apr-21	02-Apr-21	02-Apr-21	21-Jan-22
C19IMPACT_0308	Trinidad and Tobago	17-Mar-21	CARPHA	N	N	N	N	N	Y	17.01		01-Apr-21	01-Apr-21	02-Apr-21	02-Apr-21	21-Jan-22
C19IMPACT_0309	Trinidad and Tobago	17-Mar-21	CARPHA	N	N	N	N	N	Y	16.94		01-Apr-21	01-Apr-21	02-Apr-21	02-Apr-21	21-Jan-22
C19IMPACT_0310	Trinidad and Tobago	17-Mar-21	CARPHA	N	N	N	N	N	Y	17.12		01-Apr-21	01-Apr-21	02-Apr-21	02-Apr-21	21-Jan-22
C19IMPACT_0311	Trinidad and Tobago	21-Mar-21	MoH	N	N	N	N	N	Y		7.82	29-Mar-21	01-Apr-21	02-Apr-21		
C19IMPACT_0312	Trinidad and Tobago	22-Mar-21	MoH	N	N	N	N	N	Y		13.42	29-Mar-21	01-Apr-21	02-Apr-21		
C19IMPACT_0313	Trinidad and Tobago	23-Mar-21	CARPHA	N	N	N	Y	N	Y	14.71		01-Apr-21	01-Apr-21	02-Apr-21	02-Apr-21	
C19IMPACT_0314	Trinidad and Tobago	23-Mar-21	CARPHA	N	N	N	Y	N	Y	21.18		01-Apr-21	01-Apr-21	02-Apr-21	02-Apr-21	
C19IMPACT_0315	Trinidad and Tobago	23-Mar-21	CARPHA	N	N	N	Y	N	Y	15.30		01-Apr-21	01-Apr-21	02-Apr-21	02-Apr-21	
C19IMPACT_0316	Trinidad and Tobago	23-Mar-21	CARPHA	N	N	N	Y	N	Y	16.09		01-Apr-21	01-Apr-21	02-Apr-21	02-Apr-21	

C19IMPACT_0317	Trinidad and Tobago	23-Mar-21	CARPHA	N	N	N	Y	N	Y	19.32		01-Apr-21	01-Apr-21	02-Apr-21	02-Apr-21	
C19IMPACT_0318	Trinidad and Tobago	25-Mar-21	CARPHA	N	N	N	N	Y	Y	23.45		01-Apr-21	01-Apr-21	02-Apr-21	02-Apr-21	21-Jan-22
C19IMPACT_0319	Trinidad and Tobago	29-Mar-21	CARPHA	N	N	N	N	N	Y	11.15		01-Apr-21	01-Apr-21	02-Apr-21	02-Apr-21	21-Jan-22
C19IMPACT_0320	Trinidad and Tobago	31-Mar-21	CARPHA	N	N	N	N	Y	Y	20.20		01-Apr-21	01-Apr-21	02-Apr-21	02-Apr-21	
C19IMPACT_0321	Turks and Caicos Islands	01-Mar-21	CARPHA	N	N	N	N	N	N	14.87		06-Apr-21	09-Apr-21		11-Apr-21	22-Jun-21
C19IMPACT_0322	Turks and Caicos Islands	02-Mar-21	CARPHA	N	N	N	N	N	N	23.40		06-Apr-21	09-Apr-21		11-Apr-21	22-Jun-21
C19IMPACT_0323	Turks and Caicos Islands	02-Mar-21	CARPHA	N	N	N	N	N	N	21.14		06-Apr-21	09-Apr-21		11-Apr-21	22-Jun-21
C19IMPACT_0324	Turks and Caicos Islands	03-Mar-21	CARPHA	N	N	N	N	N	N	21.97		06-Apr-21	09-Apr-21		11-Apr-21	22-Jun-21
C19IMPACT_0325	Turks and Caicos Islands	12-Mar-21	CARPHA	N	N	N	N	N	N	16.08		06-Apr-21	09-Apr-21		11-Apr-21	22-Jun-21
C19IMPACT_0326	Turks and Caicos Islands	17-Mar-21	CARPHA	N	N	N	N	N	N	18.03		06-Apr-21	09-Apr-21		11-Apr-21	22-Jun-21
C19IMPACT_0327	Turks and Caicos Islands	22-Mar-21	CARPHA	N	N	N	N	N	N	17.95		06-Apr-21	09-Apr-21		11-Apr-21	22-Jun-21
C19IMPACT_0328	Turks and Caicos Islands	22-Mar-21	CARPHA	N	N	N	N	N	N	18.39		06-Apr-21	09-Apr-21		11-Apr-21	22-Jun-21
C19IMPACT_0329	Turks and Caicos Islands	22-Mar-21	CARPHA	N	N	N	N	N	N	17.91		06-Apr-21	09-Apr-21		11-Apr-21	22-Jun-21
C19IMPACT_0330	Turks and Caicos Islands	22-Mar-21	CARPHA	N	N	N	N	N	N	14.52		06-Apr-21	09-Apr-21		11-Apr-21	22-Jun-21
C19IMPACT_0331	Trinidad and Tobago	18-Feb-21	CARPHA	N	N	N	N	N	N	19.69		09-Apr-21	10-Apr-21	11-Apr-21	11-Apr-21	14-Apr-21
C19IMPACT_0332	Trinidad and Tobago	18-Feb-21	CARPHA	N	N	N	N	N	N	24.56		09-Apr-21	10-Apr-21	11-Apr-21	11-Apr-21	14-Apr-21
C19IMPACT_0333	Trinidad and Tobago	18-Feb-21	CARPHA	N	N	N	N	N	N	20.16		09-Apr-21	10-Apr-21	11-Apr-21	11-Apr-21	14-Apr-21
C19IMPACT_0334	Trinidad and Tobago	05-Mar-21	CARPHA	N	N	N	N	Y	N	24.86		09-Apr-21	10-Apr-21	11-Apr-21	11-Apr-21	14-Apr-21
C19IMPACT_0335	Bahamas	15-Mar-21	CARPHA	N	N	N	N	N	N	18.11		09-Apr-21	10-Apr-21		11-Apr-21	03-Jun-21
C19IMPACT_0336	Bahamas	16-Mar-21	CARPHA	N	N	N	N	N	N	23.94		09-Apr-21	10-Apr-21		11-Apr-21	03-Jun-21
C19IMPACT_0337	Bahamas	22-Mar-21	CARPHA	N	N	N	N	N	N	20.96		09-Apr-21	10-Apr-21		11-Apr-21	
C19IMPACT_0338	Bahamas	22-Mar-21	CARPHA	N	N	N	N	N	N	22.93		09-Apr-21	10-Apr-21		11-Apr-21	
C19IMPACT_0339	Trinidad and Tobago	04-Apr-21	CARPHA	N	N	N	N	N	N	17.35		09-Apr-21	10-Apr-21	11-Apr-21	11-Apr-21	14-Apr-21
C19IMPACT_0340	Trinidad and Tobago	05-Apr-21	CARPHA	N	N	N	N	N	N	21.49		09-Apr-21	10-Apr-21	11-Apr-21	11-Apr-21	14-Apr-21
C19IMPACT_0341	Trinidad and Tobago	05-Apr-21	CARPHA	N	N	N	N	N	N	19.39		09-Apr-21	10-Apr-21	11-Apr-21	11-Apr-21	14-Apr-21
C19IMPACT_0342	Trinidad and Tobago	06-Apr-21	CARPHA	N	N	N	N	Y	N	22.60		09-Apr-21	10-Apr-21	11-Apr-21	11-Apr-21	14-Apr-21
C19IMPACT_0343	Trinidad and Tobago	06-Apr-21	CARPHA	N	N	N	N	Y	N	20.69		09-Apr-21	10-Apr-21	11-Apr-21	11-Apr-21	14-Apr-21
C19IMPACT_0344	Trinidad and Tobago	06-Apr-21	CARPHA	N	N	N	N	Y	N	20.50		09-Apr-21	10-Apr-21	11-Apr-21	11-Apr-21	14-Apr-21
C19IMPACT_0345	Trinidad and Tobago	06-Apr-21	CARPHA	N	N	N	N	N	N	14.07		09-Apr-21	10-Apr-21	11-Apr-21	11-Apr-21	14-Apr-21
C19IMPACT_0346	Trinidad and Tobago	06-Apr-21	CARPHA	N	N	N	N	N	N	17.28		09-Apr-21	10-Apr-21	11-Apr-21	11-Apr-21	14-Apr-21
C19IMPACT_0347	Trinidad and Tobago	06-Apr-21	CARPHA	N	N	N	N	Y	N	20.36		09-Apr-21	10-Apr-21	11-Apr-21	11-Apr-21	14-Apr-21
C19IMPACT_0348	Cayman Islands	01-Feb-21	CARPHA	N	N	N	N	N	N	22.09		13-Apr-21	14-Apr-21		15-Apr-21	
C19IMPACT_0349	Trinidad and Tobago	06-Apr-21	CARPHA	N	N	N	N	Y	N	21.35		13-Apr-21	14-Apr-21	16-Apr-21	15-Apr-21	21-Jun-21
C19IMPACT_0350	Trinidad and Tobago	06-Apr-21	CARPHA	N	N	N	N	Y	N	22.05		13-Apr-21	14-Apr-21	16-Apr-21	15-Apr-21	21-Jun-21
C19IMPACT_0351	Trinidad and Tobago	06-Apr-21	CARPHA	N	N	N	N	N	N	18.85		13-Apr-21	14-Apr-21	16-Apr-21	15-Apr-21	21-Jun-21
C19IMPACT_0352	Trinidad and Tobago	07-Apr-21	CARPHA	N	N	N	N	Y	N	17.91		13-Apr-21	14-Apr-21	16-Apr-21	15-Apr-21	21-Jun-21
C19IMPACT_0353	Trinidad and Tobago	07-Apr-21	CARPHA	N	N	N	N	N	N	23.36		13-Apr-21	14-Apr-21	16-Apr-21	15-Apr-21	21-Jun-21
C19IMPACT_0354	Trinidad and Tobago	07-Apr-21	CARPHA	N	N	N	N	N	N	23.28		13-Apr-21	14-Apr-21	16-Apr-21	15-Apr-21	21-Jun-21
C19IMPACT_0355	Trinidad and Tobago	07-Apr-21	CARPHA	N	N	N	N	N	N	20.64		13-Apr-21	14-Apr-21	16-Apr-21	15-Apr-21	21-Jun-21
C19IMPACT_0356	Trinidad and Tobago	07-Apr-21	CARPHA	N	N	N	N	N	N	24.76		13-Apr-21	14-Apr-21	16-Apr-21	15-Apr-21	
C19IMPACT_0357	Trinidad and Tobago	07-Apr-21	CARPHA	N	N	N	N	N	N	18.99		13-Apr-21	14-Apr-21	16-Apr-21	15-Apr-21	21-Jun-21
C19IMPACT_0358	Trinidad and Tobago	07-Apr-21	CARPHA	N	N	N	N	N	N	17.28		13-Apr-21	14-Apr-21	16-Apr-21	15-Apr-21	21-Jun-21
C19IMPACT_0359	Trinidad and Tobago	08-Apr-21	CARPHA	N	N	N	N	N	N	20.91		13-Apr-21	14-Apr-21	16-Apr-21	15-Apr-21	21-Jun-21
C19IMPACT_0360	Trinidad and Tobago	08-Apr-21	CARPHA	N	N	N	N	Y	N	21.17		13-Apr-21	14-Apr-21	16-Apr-21	15-Apr-21	21-Jun-21
C19IMPACT_0361	Trinidad and Tobago	08-Apr-21	CARPHA	N	N	N	N	N	N	16.54		13-Apr-21	14-Apr-21	16-Apr-21	15-Apr-21	21-Jun-21
C19IMPACT_0362	Trinidad and Tobago	08-Apr-21	CARPHA	N	N	N	N	N	N	22.11		13-Apr-21	14-Apr-21	16-Apr-21	15-Apr-21	21-Jun-21
C19IMPACT_0363	Trinidad and Tobago	08-Apr-21	CARPHA	N	N	N	N	N	N	16.93		13-Apr-21	14-Apr-21	16-Apr-21	15-Apr-21	21-Jun-21
C19IMPACT_0364	Trinidad and Tobago	08-Apr-21	CARPHA	N	N	N	N	Y	N	17.96		13-Apr-21	14-Apr-21	16-Apr-21	15-Apr-21	21-Jun-21
C19IMPACT_0365	Trinidad and Tobago	09-Apr-21	CARPHA	N	N	N	N	N	N	24.04		13-Apr-21	14-Apr-21	16-Apr-21	15-Apr-21	
C19IMPACT_0366	Trinidad and Tobago	10-Apr-21	CARPHA	N	N	N	N	N	N	23.12		14-Apr-21	16-Apr-21	16-Apr-21	16-Apr-21	20-Jun-21
C19IMPACT_0367	Trinidad and Tobago	11-Apr-21	CARPHA	N	N	N	N	Y	N	14.97		14-Apr-21	16-Apr-21	16-Apr-21	16-Apr-21	20-Jun-21
C19IMPACT_0368	Trinidad and Tobago	11-Apr-21	CARPHA	N	N	N	N	Y	N	14.87		14-Apr-21	16-Apr-21	16-Apr-21	16-Apr-21	20-Jun-21
C19IMPACT_0369	Trinidad and Tobago	11-Apr-21	CARPHA	N	N	N	N	Y	N	20.42		14-Apr-21	16-Apr-21	16-Apr-21	16-Apr-21	20-Jun-21
C19IMPACT_0370	Trinidad and Tobago	11-Apr-21	CARPHA	N	N	N	N	Y	N	17.15		14-Apr-21	16-Apr-21	16-Apr-21	16-Apr-21	20-Jun-21
C19IMPACT_0371	Trinidad and Tobago	11-Apr-21	CARPHA	N	N	N	N	Y	N	22.08		14-Apr-21	16-Apr-21	16-Apr-21	16-Apr-21	20-Jun-21
C19IMPACT_0372	Trinidad and Tobago	11-Apr-21	CARPHA	N	N	N	N	Y	N	23.29		14-Apr-21	16-Apr-21	16-Apr-21	16-Apr-21	20-Jun-21
C19IMPACT_0373	Trinidad and Tobago	11-Apr-21	CARPHA	N	N	N	N	Y	N	17.69		14-Apr-21	16-Apr-21	16-Apr-21	16-Apr-21	20-Jun-21
C19IMPACT_0374	Trinidad and Tobago	11-Apr-21	CARPHA	N	N	N	N	Y	N	14.37		14-Apr-21	16-Apr-21	16-Apr-21	16-Apr-21	20-Jun-21
C19IMPACT_0375	Trinidad and Tobago	12-Apr-21	CARPHA	N	N	N	N	N	N	20.49		14-Apr-21	16-Apr-21	16-Apr-21	16-Apr-21	20-Jun-21
C19IMPACT_0376	Trinidad and Tobago	12-Apr-21	CARPHA	N	N	N	N	N	N	16.87		14-Apr-21	16-Apr-21	16-Apr-21	16-Apr-21	20-Jun-21
C19IMPACT_0377	Trinidad and Tobago	12-Apr-21	CARPHA	N	N	N	N	N	N	13.73		14-Apr-21	16-Apr-21	16-Apr-21	16-Apr-21	20-Jun-21
C19IMPACT_0378	Trinidad and Tobago	12-Apr-21	CARPHA	N	N	N	N	N	N	22.47		14-Apr-21	16-Apr-21	16-Apr-21	16-Apr-21	20-Jun-21
C19IMPACT_0379	Trinidad and Tobago	12-Apr-21	CARPHA	N	N	N	N	N	N	16.81		14-Apr-21	16-Apr-21	16-Apr-21	16-Apr-21	20-Jun-21
C19IMPACT_0380	Trinidad and Tobago	12-Apr-21	CARPHA	N	N	N	N	N	N	14.08		14-Apr-21	16-Apr-21	16-Apr-21	16-Apr-21	20-Jun-21

C19IMPACT_0381	Trinidad and Tobago	12-Apr-21	CARPHA	N	N	N	N	N	N	16.14		14-Apr-21	16-Apr-21	16-Apr-21	16-Apr-21	20-Jun-21
C19IMPACT_0382	Trinidad and Tobago	12-Apr-21	CARPHA	N	N	N	N	N	N	16.31		14-Apr-21	16-Apr-21	16-Apr-21	16-Apr-21	20-Jun-21
C19IMPACT_0383	Trinidad and Tobago	12-Apr-21	CARPHA	N	N	N	N	N	N	16.32		15-Apr-21	16-Apr-21	16-Apr-21	16-Apr-21	20-Jun-21
C19IMPACT_0384	Trinidad and Tobago	12-Apr-21	CARPHA	N	N	N	N	N	N	21.82		15-Apr-21	16-Apr-21	16-Apr-21	16-Apr-21	21-Jan-22
C19IMPACT_0385	Trinidad and Tobago	13-Apr-21	CARPHA	N	N	N	N	N	N	22.25		15-Apr-21	16-Apr-21	16-Apr-21	16-Apr-21	20-Jun-21
C19IMPACT_0386	Trinidad and Tobago	13-Apr-21	CARPHA	N	N	N	N	N	N	17.84		15-Apr-21	16-Apr-21	16-Apr-21	16-Apr-21	20-Jun-21
C19IMPACT_0387	Trinidad and Tobago	13-Apr-21	CARPHA	N	N	N	N	N	N	16.83		15-Apr-21	16-Apr-21	16-Apr-21	16-Apr-21	20-Jun-21
C19IMPACT_0388	Trinidad and Tobago	13-Apr-21	MoH	N	N	N	N	N	N	18.41		15-Apr-21	17-Apr-21	23-Apr-21		
C19IMPACT_0389	Trinidad and Tobago	14-Apr-21	CARPHA	N	N	N	N	N	N	21.58		16-Apr-21	17-Apr-21	23-Apr-21	22-Apr-21	20-Jun-21
C19IMPACT_0390	Trinidad and Tobago	14-Apr-21	CARPHA	N	N	N	N	N	N	16.37		16-Apr-21	17-Apr-21	23-Apr-21	22-Apr-21	20-Jun-21
C19IMPACT_0391	Trinidad and Tobago	14-Apr-21	CARPHA	N	N	N	N	N	N	19.67		16-Apr-21	17-Apr-21	23-Apr-21	22-Apr-21	20-Jun-21
C19IMPACT_0392	Trinidad and Tobago	14-Apr-21	CARPHA	N	N	N	N	N	N	16.95		16-Apr-21	17-Apr-21	23-Apr-21	22-Apr-21	
C19IMPACT_0393	Trinidad and Tobago	15-Apr-21	CARPHA	N	N	N	N	N	N	21.29		16-Apr-21	17-Apr-21	23-Apr-21	22-Apr-21	
C19IMPACT_0394	Trinidad and Tobago	15-Apr-21	CARPHA	N	N	N	N	N	N	24.17		16-Apr-21	17-Apr-21	23-Apr-21	22-Apr-21	
C19IMPACT_0395	Trinidad and Tobago	15-Apr-21	CARPHA	N	N	N	N	Y	N	19.03		16-Apr-21	17-Apr-21	23-Apr-21	22-Apr-21	20-Jun-21
C19IMPACT_0396	Trinidad and Tobago	14-Apr-21	CARPHA	N	N	N	N	N	N	21.06		20-Apr-21	21-Apr-21	23-Apr-21	22-Apr-21	17-Jun-21
C19IMPACT_0397	Trinidad and Tobago	15-Apr-21	CARPHA	N	N	N	N	N	N	22.35		19-Apr-21	21-Apr-21	23-Apr-21	22-Apr-21	17-Jun-21
C19IMPACT_0398	Trinidad and Tobago	15-Apr-21	CARPHA	N	N	N	N	N	N	24.33		19-Apr-21	21-Apr-21	23-Apr-21	22-Apr-21	17-Jun-21
C19IMPACT_0399	Trinidad and Tobago	15-Apr-21	CARPHA	N	N	N	N	N	N	17.39		19-Apr-21	21-Apr-21	23-Apr-21	22-Apr-21	17-Jun-21
C19IMPACT_0400	Trinidad and Tobago	15-Apr-21	CARPHA	N	N	N	N	N	N	21.67		19-Apr-21	21-Apr-21	23-Apr-21	22-Apr-21	17-Jun-21
C19IMPACT_0401	Trinidad and Tobago	15-Apr-21	CARPHA	N	N	N	N	N	N	18.89		19-Apr-21	21-Apr-21	23-Apr-21	22-Apr-21	17-Jun-21
C19IMPACT_0402	Trinidad and Tobago	15-Apr-21	CARPHA	N	N	N	N	N	N	18.43		19-Apr-21	21-Apr-21	23-Apr-21	22-Apr-21	17-Jun-21
C19IMPACT_0403	Trinidad and Tobago	15-Apr-21	CARPHA	N	N	N	N	N	N	18.17		19-Apr-21	21-Apr-21	23-Apr-21	22-Apr-21	17-Jun-21
C19IMPACT_0404	Trinidad and Tobago	15-Apr-21	CARPHA	N	N	N	N	N	N	22.46		19-Apr-21	21-Apr-21	23-Apr-21	22-Apr-21	
C19IMPACT_0405	Trinidad and Tobago	16-Apr-21	CARPHA	N	N	N	N	Y	N	19.70		20-Apr-21	21-Apr-21	23-Apr-21	22-Apr-21	17-Jun-21
C19IMPACT_0406	Trinidad and Tobago	16-Apr-21	CARPHA	N	N	N	N	N	N	20.32		20-Apr-21	21-Apr-21	23-Apr-21	22-Apr-21	17-Jun-21
C19IMPACT_0407	Trinidad and Tobago	16-Apr-21	CARPHA	N	N	N	N	N	N	21.99		20-Apr-21	21-Apr-21	23-Apr-21	22-Apr-21	17-Jun-21
C19IMPACT_0408	Trinidad and Tobago	16-Apr-21	CARPHA	N	N	N	N	N	N	22.85		20-Apr-21	21-Apr-21	23-Apr-21	22-Apr-21	17-Jun-21
C19IMPACT_0409	Trinidad and Tobago	16-Apr-21	CARPHA	N	N	N	N	N	N	17.77		20-Apr-21	21-Apr-21	23-Apr-21	22-Apr-21	17-Jun-21
C19IMPACT_0410	Trinidad and Tobago	16-Apr-21	CARPHA	N	N	N	N	N	N	19.42		20-Apr-21	21-Apr-21	23-Apr-21	22-Apr-21	17-Jun-21
C19IMPACT_0411	Trinidad and Tobago	16-Apr-21	CARPHA	N	N	N	N	N	N	20.12		20-Apr-21	21-Apr-21	23-Apr-21	22-Apr-21	17-Jun-21
C19IMPACT_0412	Trinidad and Tobago	16-Apr-21	CARPHA	N	N	N	N	N	N	18.72		20-Apr-21	21-Apr-21	23-Apr-21	22-Apr-21	17-Jun-21
C19IMPACT_0413	Trinidad and Tobago	16-Apr-21	CARPHA	N	N	N	N	N	N	21.22		20-Apr-21	21-Apr-21	23-Apr-21	22-Apr-21	17-Jun-21
C19IMPACT_0414	Trinidad and Tobago	16-Apr-21	CARPHA	N	N	N	N	N	N	17.81		20-Apr-21	21-Apr-21	23-Apr-21	22-Apr-21	17-Jun-21
C19IMPACT_0415	Trinidad and Tobago	17-Apr-21	CARPHA	N	N	N	N	N	N	17.77		20-Apr-21	21-Apr-21	23-Apr-21	22-Apr-21	17-Jun-21
C19IMPACT_0416	Trinidad and Tobago	17-Apr-21	CARPHA	N	N	N	N	Y	N	19.76		20-Apr-21	21-Apr-21	23-Apr-21	22-Apr-21	17-Jun-21
C19IMPACT_0417	Trinidad and Tobago	18-Apr-21	CARPHA	N	N	N	N	Y	N	20.42		20-Apr-21	21-Apr-21	23-Apr-21	22-Apr-21	17-Jun-21
C19IMPACT_0418	Trinidad and Tobago	19-Apr-21	CARPHA	N	N	N	N	Y	N	22.07		20-Apr-21	21-Apr-21	23-Apr-21	22-Apr-21	17-Jun-21
C19IMPACT_0419	Jamaica	21-Jan-21	CARPHA	N	N	N	N	N	N	16.56		21-Apr-21	22-Apr-21		25-Apr-21	16-Jun-21
C19IMPACT_0420	Jamaica	25-Jan-21	CARPHA	N	N	N	N	N	N	18.55		21-Apr-21	22-Apr-21		25-Apr-21	16-Jun-21
C19IMPACT_0421	Jamaica	26-Jan-21	CARPHA	N	N	N	N	N	N	19.54		21-Apr-21	22-Apr-21		25-Apr-21	16-Jun-21
C19IMPACT_0422	Jamaica	27-Jan-21	CARPHA	N	N	N	N	N	N	14.86		21-Apr-21	22-Apr-21		25-Apr-21	16-Jun-21
C19IMPACT_0423	Jamaica	28-Jan-21	CARPHA	N	N	N	N	N	N	21.98		21-Apr-21	22-Apr-21		25-Apr-21	16-Jun-21
C19IMPACT_0424	Jamaica	02-Feb-21	CARPHA	N	N	N	N	N	N	20.52		21-Apr-21	22-Apr-21		25-Apr-21	16-Jun-21
C19IMPACT_0425	Jamaica	02-Feb-21	CARPHA	N	N	N	N	N	N	17.12		21-Apr-21	22-Apr-21		25-Apr-21	16-Jun-21
C19IMPACT_0426	Jamaica	02-Feb-21	CARPHA	N	N	N	N	N	N	20.46		21-Apr-21	22-Apr-21		25-Apr-21	16-Jun-21
C19IMPACT_0427	Jamaica	11-Feb-21	CARPHA	N	N	N	N	N	N	23.53		21-Apr-21	22-Apr-21		25-Apr-21	
C19IMPACT_0428	Jamaica	12-Feb-21	CARPHA	N	N	N	N	N	N	19.03		21-Apr-21	22-Apr-21		25-Apr-21	16-Jun-21
C19IMPACT_0429	Trinidad and Tobago	16-Apr-21	CARPHA	N	N	N	N	N	N	15.52		21-Apr-21	22-Apr-21	30-Apr-21	25-Apr-21	16-Jun-21
C19IMPACT_0430	Trinidad and Tobago	17-Apr-21	CARPHA	N	N	N	N	N	N	22.46		21-Apr-21	22-Apr-21	30-Apr-21	25-Apr-21	16-Jun-21
C19IMPACT_0431	Trinidad and Tobago	17-Apr-21	CARPHA	N	N	N	N	N	N	19.98		21-Apr-21	22-Apr-21	30-Apr-21	25-Apr-21	16-Jun-21
C19IMPACT_0432	Trinidad and Tobago	19-Apr-21	CARPHA	N	N	N	N	N	N	17.79		21-Apr-21	22-Apr-21	30-Apr-21	25-Apr-21	16-Jun-21
C19IMPACT_0433	Trinidad and Tobago	19-Apr-21	CARPHA	N	N	N	N	N	N	18.37		21-Apr-21	22-Apr-21	30-Apr-21	25-Apr-21	16-Jun-21
C19IMPACT_0434	Trinidad and Tobago	19-Apr-21	CARPHA	N	N	N	N	N	N	23.29		21-Apr-21	22-Apr-21	30-Apr-21	25-Apr-21	16-Jun-21
C19IMPACT_0435	Trinidad and Tobago	19-Apr-21	CARPHA	N	N	N	N	Y	N	21.01		21-Apr-21	22-Apr-21	30-Apr-21	25-Apr-21	16-Jun-21
C19IMPACT_0436	Trinidad and Tobago	19-Apr-21	CARPHA	N	N	N	N	Y	N	17.97		23-Apr-21	24-Apr-21	30-Apr-21	27-Apr-21	24-Apr-21
C19IMPACT_0437	Trinidad and Tobago	19-Apr-21	CARPHA	N	N	N	N	Y	N	23.09		23-Apr-21	24-Apr-21	30-Apr-21	27-Apr-21	24-Apr-21
C19IMPACT_0438	Trinidad and Tobago	19-Apr-21	CARPHA	N	N	N	N	Y	N	19.53		23-Apr-21	24-Apr-21	30-Apr-21	27-Apr-21	24-Apr-21
C19IMPACT_0439	Trinidad and Tobago	19-Apr-21	CARPHA	N	N	N	N	Y	N	21.92		23-Apr-21	24-Apr-21	30-Apr-21	27-Apr-21	24-Apr-21
C19IMPACT_0440	Trinidad and Tobago	19-Apr-21	CARPHA	N	N	N	N	Y	N	20.71		23-Apr-21	24-Apr-21	30-Apr-21	27-Apr-21	
C19IMPACT_0441	Trinidad and Tobago	19-Apr-21	CARPHA	N	N	N	N	Y	N	18.74		23-Apr-21	24-Apr-21	30-Apr-21	27-Apr-21	24-Apr-21
C19IMPACT_0442	Trinidad and Tobago	19-Apr-21	CARPHA	N	N	N	N	Y	N	24.29		23-Apr-21	24-Apr-21	30-Apr-21	27-Apr-21	
C19IMPACT_0443	Trinidad and Tobago	19-Apr-21	CARPHA	N	N	Y	N	Y	N	17.85		23-Apr-21	24-Apr-21	30-Apr-21	27-Apr-21	
C19IMPACT_0444	Trinidad and Tobago	19-Apr-21	CARPHA	N	N	N	N	N	N	17.26		23-Apr-21	24-Apr-21	30-Apr-21	27-Apr-21	24-Apr-21

C19IMPACT_0445	Trinidad and Tobago	19-Apr-21	CARPHA	N	N	N	N	Y	N	20.13		23-Apr-21	24-Apr-21	30-Apr-21	27-Apr-21	24-Apr-21
C19IMPACT_0446	Trinidad and Tobago	19-Apr-21	CARPHA	N	N	N	N	N	N	19.34		23-Apr-21	24-Apr-21	30-Apr-21	27-Apr-21	24-Apr-21
C19IMPACT_0447	Trinidad and Tobago	20-Apr-21	CARPHA	N	N	Y	N	N	N	22.25		23-Apr-21	24-Apr-21	30-Apr-21	27-Apr-21	
C19IMPACT_0448	Trinidad and Tobago	20-Apr-21	CARPHA	N	N	N	N	Y	N	19.73		23-Apr-21	24-Apr-21	30-Apr-21	27-Apr-21	24-Apr-21
C19IMPACT_0449	Trinidad and Tobago	20-Apr-21	CARPHA	N	N	N	N	N	N	20.97		23-Apr-21	24-Apr-21	30-Apr-21	27-Apr-21	24-Apr-21
C19IMPACT_0450	Trinidad and Tobago	20-Apr-21	CARPHA	N	N	N	N	N	N	21.47		23-Apr-21	24-Apr-21	30-Apr-21	27-Apr-21	24-Apr-21
C19IMPACT_0451	Trinidad and Tobago	20-Apr-21	CARPHA	N	N	Y	N	N	N	22.77		23-Apr-21	24-Apr-21	30-Apr-21	27-Apr-21	24-Apr-21
C19IMPACT_0452	Trinidad and Tobago	20-Apr-21	CARPHA	N	N	N	N	N	N	24.16		23-Apr-21	24-Apr-21	30-Apr-21	27-Apr-21	24-Apr-21
C19IMPACT_0453	Trinidad and Tobago	20-Apr-21	CARPHA	N	N	N	N	N	N	19.21		23-Apr-21	24-Apr-21	30-Apr-21	27-Apr-21	24-Apr-21
C19IMPACT_0454	Trinidad and Tobago	20-Apr-21	CARPHA	N	N	N	N	N	N	18.12		23-Apr-21	24-Apr-21	30-Apr-21	27-Apr-21	24-Apr-21
C19IMPACT_0455	Trinidad and Tobago	20-Apr-21	CARPHA	N	N	N	N	N	N	16.47		23-Apr-21	24-Apr-21	30-Apr-21	27-Apr-21	24-Apr-21
C19IMPACT_0456	Barbados	08-Apr-21	CARPHA	N	N	N	N	N	N	19.75		23-Apr-21	26-Apr-21		28-Apr-21	15-Jun-21
C19IMPACT_0457	Grenada	08-Apr-21	CARPHA	N	N	N	N	N	N	17.15		23-Apr-21	26-Apr-21		28-Apr-21	15-Jun-21
C19IMPACT_0458	Barbados	10-Apr-21	CARPHA	N	N	N	N	N	N	19.51		23-Apr-21	26-Apr-21		28-Apr-21	15-Jun-21
C19IMPACT_0459	Barbados	10-Apr-21	CARPHA	N	N	N	N	N	N	22.43		23-Apr-21	26-Apr-21		28-Apr-21	15-Jun-21
C19IMPACT_0460	Barbados	10-Apr-21	CARPHA	N	N	N	N	N	N	24.10		23-Apr-21	26-Apr-21		28-Apr-21	
C19IMPACT_0461	Barbados	10-Apr-21	CARPHA	N	N	N	N	N	N	15.45		23-Apr-21	26-Apr-21		28-Apr-21	15-Jun-21
C19IMPACT_0462	Barbados	10-Apr-21	CARPHA	N	N	N	N	N	N	19.95		23-Apr-21	26-Apr-21		28-Apr-21	15-Jun-21
C19IMPACT_0463	Barbados	12-Apr-21	CARPHA	N	N	N	N	N	N	21.68		23-Apr-21	26-Apr-21		28-Apr-21	15-Jun-21
C19IMPACT_0464	Barbados	12-Apr-21	CARPHA	N	N	N	N	N	N	21.47		23-Apr-21	26-Apr-21		28-Apr-21	15-Jun-21
C19IMPACT_0465	Grenada	12-Apr-21	CARPHA	N	N	N	N	N	N	22.59		23-Apr-21	26-Apr-21		28-Apr-21	15-Jun-21
C19IMPACT_0466	Barbados	14-Apr-21	CARPHA	N	N	N	N	N	N	19.29		23-Apr-21	26-Apr-21		28-Apr-21	
C19IMPACT_0467	Trinidad and Tobago	19-Apr-21	CARPHA	N	N	N	N	N	N	22.22		23-Apr-21	26-Apr-21	30-Apr-21	28-Apr-21	15-Jun-21
C19IMPACT_0468	Trinidad and Tobago	19-Apr-21	CARPHA	N	N	N	N	N	N	19.12		23-Apr-21	26-Apr-21	30-Apr-21	28-Apr-21	15-Jun-21
C19IMPACT_0469	Trinidad and Tobago	19-Apr-21	CARPHA	N	N	N	N	N	N	23.48		23-Apr-21	26-Apr-21	30-Apr-21	28-Apr-21	15-Jun-21
C19IMPACT_0470	Trinidad and Tobago	20-Apr-21	CARPHA	N	N	N	N	N	N	17.27		23-Apr-21	26-Apr-21	30-Apr-21	28-Apr-21	15-Jun-21
C19IMPACT_0471	Trinidad and Tobago	20-Apr-21	CARPHA	N	N	N	N	N	N	16.45		23-Apr-21	26-Apr-21	30-Apr-21	28-Apr-21	
C19IMPACT_0472	Trinidad and Tobago	20-Apr-21	CARPHA	N	N	N	N	N	N	17.10		23-Apr-21	26-Apr-21	30-Apr-21	28-Apr-21	15-Jun-21
C19IMPACT_0473	Trinidad and Tobago	20-Apr-21	CARPHA	Y	N	Y	N	N	N	18.05		23-Apr-21	26-Apr-21	30-Apr-21	28-Apr-21	15-Jun-21
C19IMPACT_0474	Trinidad and Tobago	26-Apr-21	CARPHA	N	N	N	N	N	N	16.45		29-Apr-21	27-Apr-21			
C19IMPACT_0475	Anguilla	20-Apr-21	CARPHA	N	N	N	N	N	N	20.76		27-Apr-21	28-Apr-21		30-Apr-21	03-Jun-21
C19IMPACT_0476	Trinidad and Tobago	20-Apr-21	CARPHA	N	N	N	N	Y	N	20.04		27-Apr-21	28-Apr-21	30-Apr-21	30-Apr-21	03-Jun-21
C19IMPACT_0477	Anguilla	21-Apr-21	CARPHA	N	N	N	N	N	N	22.21		27-Apr-21	28-Apr-21		30-Apr-21	03-Jun-21
C19IMPACT_0478	Anguilla	21-Apr-21	CARPHA	N	N	N	N	N	N	24.81		27-Apr-21	28-Apr-21		30-Apr-21	
C19IMPACT_0479	Anguilla	21-Apr-21	CARPHA	N	N	N	N	N	N	20.57		27-Apr-21	28-Apr-21		30-Apr-21	03-Jun-21
C19IMPACT_0480	Anguilla	21-Apr-21	CARPHA	N	N	N	N	N	Y	21.09		27-Apr-21	28-Apr-21		06-Dec-22	
C19IMPACT_0481	Trinidad and Tobago	21-Apr-21	CARPHA	N	N	N	N	N	N	23.08		27-Apr-21	28-Apr-21	30-Apr-21	30-Apr-21	
C19IMPACT_0482	Trinidad and Tobago	21-Apr-21	CARPHA	N	N	N	N	N	N	21.97		27-Apr-21	28-Apr-21	30-Apr-21	30-Apr-21	03-Jun-21
C19IMPACT_0483	Anguilla	22-Apr-21	CARPHA	N	N	N	N	N	N	20.49		27-Apr-21	28-Apr-21		30-Apr-21	03-Jun-21
C19IMPACT_0484	Anguilla	22-Apr-21	CARPHA	N	N	N	N	N	N	19.53		27-Apr-21	28-Apr-21		30-Apr-21	03-Jun-21
C19IMPACT_0485	Anguilla	22-Apr-21	CARPHA	N	N	N	N	N	N	20.29		27-Apr-21	28-Apr-21		30-Apr-21	03-Jun-21
C19IMPACT_0486	Anguilla	22-Apr-21	CARPHA	N	N	N	N	N	N	20.91		27-Apr-21	28-Apr-21		30-Apr-21	03-Jun-21
C19IMPACT_0487	Anguilla	22-Apr-21	CARPHA	N	N	N	N	N	Y	23.41		27-Apr-21	28-Apr-21		06-Dec-22	
C19IMPACT_0488	Trinidad and Tobago	22-Apr-21	CARPHA	N	N	N	N	Y	N	16.01		27-Apr-21	28-Apr-21	30-Apr-21	30-Apr-21	03-Jun-21
C19IMPACT_0489	Trinidad and Tobago	22-Apr-21	CARPHA	N	N	N	N	N	N	17.00		27-Apr-21	28-Apr-21	30-Apr-21	30-Apr-21	03-Jun-21
C19IMPACT_0490	Trinidad and Tobago	22-Apr-21	CARPHA	N	N	N	N	N	N	22.71		27-Apr-21	28-Apr-21	30-Apr-21	30-Apr-21	03-Jun-21
C19IMPACT_0491	Trinidad and Tobago	22-Apr-21	CARPHA	N	N	N	N	N	N	17.93		27-Apr-21	28-Apr-21	30-Apr-21	30-Apr-21	03-Jun-21
C19IMPACT_0492	Trinidad and Tobago	22-Apr-21	CARPHA	N	N	N	N	Y	N	22.94		27-Apr-21	28-Apr-21	30-Apr-21	30-Apr-21	03-Jun-21
C19IMPACT_0493	Trinidad and Tobago	22-Apr-21	CARPHA	N	N	N	N	Y	N	21.28		27-Apr-21	28-Apr-21	30-Apr-21	30-Apr-21	03-Jun-21
C19IMPACT_0494	Trinidad and Tobago	23-Apr-21	CARPHA	N	N	N	N	N	N	23.96		27-Apr-21	28-Apr-21	30-Apr-21	30-Apr-21	
C19IMPACT_0495	Trinidad and Tobago	23-Apr-21	CARPHA	N	N	N	N	N	N	23.25		27-Apr-21	28-Apr-21	30-Apr-21	30-Apr-21	
C19IMPACT_0496	Trinidad and Tobago	26-Apr-21	MoH	N	N	N	N	N	N			27-Apr-21	28-Apr-21	30-Apr-21		21-Jan-22
C19IMPACT_0497	Trinidad and Tobago	08-Apr-21	MoH	Y	N	Y	N	N	Y		8.07	26-Apr-21	29-Apr-21	11-Jun-21		02-Jun-21
C19IMPACT_0498	Trinidad and Tobago	14-Apr-21	MoH	N	N	N	N	N	Y		8.15	22-Apr-21	29-Apr-21	13-Oct-21	01-May-21	02-Jun-21
C19IMPACT_0499	Trinidad and Tobago	19-Apr-21	CARPHA	N	N	N	N	Y	N	19.96		26-Apr-21	29-Apr-21	11-Jun-21	01-May-21	02-Jun-21
C19IMPACT_0500	Trinidad and Tobago	19-Apr-21	CARPHA	N	N	N	N	N	N	23.63		26-Apr-21	29-Apr-21	13-Oct-21	01-May-21	02-Jun-21
C19IMPACT_0501	Trinidad and Tobago	20-Apr-21	CARPHA	N	N	N	N	N	N	23.25		26-Apr-21	29-Apr-21	27-Aug-21	01-May-21	02-Jun-21
C19IMPACT_0502	Trinidad and Tobago	20-Apr-21	CARPHA	N	N	N	N	N	N	16.80		27-Apr-21	29-Apr-21	11-Jun-21	01-May-21	02-Jun-21
C19IMPACT_0503	Trinidad and Tobago	20-Apr-21	CARPHA	N	N	N	N	N	N	15.92		26-Apr-21	29-Apr-21	13-Oct-21	01-May-21	02-Jun-21
C19IMPACT_0504	Trinidad and Tobago	21-Apr-21	CARPHA	N	N	N	N	Y	N	16.33		27-Apr-21	29-Apr-21	11-Jun-21	01-May-21	02-Jun-21
C19IMPACT_0505	Trinidad and Tobago	21-Apr-21	CARPHA	N	N	N	N	N	N	23.84		26-Apr-21	29-Apr-21	13-Oct-21	01-May-21	
C19IMPACT_0506	Trinidad and Tobago	22-Apr-21	CARPHA	N	N	N	N	N	N	20.63		27-Apr-21	29-Apr-21	13-Oct-21	01-May-21	02-Jun-21
C19IMPACT_0507	Trinidad and Tobago	22-Apr-21	CARPHA	N	N	N	N	N	N	22.18		27-Apr-21	29-Apr-21	13-Oct-21	10-May-21	02-Jun-21
C19IMPACT_0508	Trinidad and Tobago	22-Apr-21	CARPHA	N	N	N	N	Y	N	16.02		27-Apr-21	29-Apr-21	11-Jun-21	01-May-21	02-Jun-21

C19IMPACT_0509	Trinidad and Tobago	22-Apr-21	MoH	N	N	N	N	Y	N			26-Apr-21	29-Apr-21	11-Jun-21		02-Jun-21
C19IMPACT_0510	Trinidad and Tobago	23-Apr-21	MoH	N	N	N	N	N	N			26-Apr-21	29-Apr-21	11-Jun-21		02-Jun-21
C19IMPACT_0511	Trinidad and Tobago	23-Apr-21	MoH	N	N	N	N	N	N			26-Apr-21	29-Apr-21	11-Jun-21		
C19IMPACT_0512	Trinidad and Tobago	24-Apr-21	MoH	N	N	N	N	Y	N			26-Apr-21	29-Apr-21	11-Jun-21		02-Jun-21
C19IMPACT_0513	Trinidad and Tobago	24-Apr-21	MoH	N	N	N	N	Y	N			26-Apr-21	29-Apr-21	13-Oct-21	01-May-21	02-Jun-21
C19IMPACT_0514	Trinidad and Tobago	25-Apr-21	MoH	N	N	N	N	N	N			26-Apr-21	29-Apr-21	11-Jun-21		
C19IMPACT_0515	Trinidad and Tobago	25-Apr-21	MoH	N	N	N	N	N	N			26-Apr-21	29-Apr-21	13-Oct-21	01-May-21	02-Jun-21
C19IMPACT_0516	Trinidad and Tobago	25-Apr-21	MoH	N	N	N	N	N	N			26-Apr-21	29-Apr-21	13-Oct-21	01-May-21	
C19IMPACT_0517	Trinidad and Tobago	25-Apr-21	MoH	N	N	N	N	Y	N			26-Apr-21	29-Apr-21	13-Oct-21	01-May-21	02-Jun-21
C19IMPACT_0518	Trinidad and Tobago	25-Apr-21	MoH	Y	N	Y	N	N	N			26-Apr-21	29-Apr-21	13-Oct-21	01-May-21	02-Jun-21
C19IMPACT_0519	Trinidad and Tobago	23-Apr-21	MoH	N	N	N	N	N	N		11.98	30-Apr-21	30-Apr-21	14-May-21	10-May-21	
C19IMPACT_0520	Trinidad and Tobago	23-Apr-21	CARPHA	N	N	N	N	N	N	23.61		27-Apr-21	01-May-21	08-May-21	04-May-21	23-Jun-21
C19IMPACT_0521	Trinidad and Tobago	23-Apr-21	CARPHA	N	N	Y	N	Y	N	17.28		27-Apr-21	01-May-21	08-May-21	04-May-21	23-Jun-21
C19IMPACT_0522	Trinidad and Tobago	23-Apr-21	CARPHA	N	N	N	N	Y	N	20.33		27-Apr-21	01-May-21	08-May-21	04-May-21	23-Jun-21
C19IMPACT_0523	Trinidad and Tobago	23-Apr-21	CARPHA	N	N	N	N	Y	N	21.59		27-Apr-21	01-May-21	08-May-21	04-May-21	23-Jun-21
C19IMPACT_0524	Trinidad and Tobago	23-Apr-21	CARPHA	N	N	N	N	N	N	18.11		27-Apr-21	01-May-21	08-May-21	04-May-21	23-Jun-21
C19IMPACT_0525	Trinidad and Tobago	23-Apr-21	CARPHA	N	N	N	N	Y	N	22.95		27-Apr-21	01-May-21	08-May-21	04-May-21	23-Jun-21
C19IMPACT_0526	Trinidad and Tobago	23-Apr-21	CARPHA	N	N	N	N	Y	N	13.80		27-Apr-21	01-May-21	08-May-21	04-May-21	23-Jun-21
C19IMPACT_0527	Trinidad and Tobago	23-Apr-21	CARPHA	N	N	N	N	Y	N	20.54		27-Apr-21	01-May-21	08-May-21	04-May-21	23-Jun-21
C19IMPACT_0528	Trinidad and Tobago	24-Apr-21	CARPHA	N	N	N	N	N	Y	16.08		27-Apr-21	01-May-21	08-May-21	04-May-21	23-Jun-21
C19IMPACT_0529	Trinidad and Tobago	24-Apr-21	CARPHA	N	N	N	N	N	N	20.70		27-Apr-21	01-May-21	08-May-21	04-May-21	23-Jun-21
C19IMPACT_0530	Trinidad and Tobago	24-Apr-21	CARPHA	N	N	N	N	Y	N	18.63		27-Apr-21	01-May-21	08-May-21	04-May-21	21-Jan-22
C19IMPACT_0531	Trinidad and Tobago	24-Apr-21	CARPHA	N	N	N	N	Y	N	23.57		27-Apr-21	01-May-21	08-May-21	04-May-21	23-Jun-21
C19IMPACT_0532	Trinidad and Tobago	24-Apr-21	CARPHA	N	N	N	N	Y	N	17.88		27-Apr-21	01-May-21	08-May-21	04-May-21	23-Jun-21
C19IMPACT_0533	Trinidad and Tobago	24-Apr-21	CARPHA	N	N	N	N	Y	N	19.58		27-Apr-21	01-May-21	08-May-21	04-May-21	23-Jun-21
C19IMPACT_0534	Trinidad and Tobago	24-Apr-21	CARPHA	N	N	N	N	N	N	21.53		27-Apr-21	01-May-21	08-May-21	04-May-21	23-Jun-21
C19IMPACT_0535	Trinidad and Tobago	24-Apr-21	CARPHA	N	N	N	N	N	N	16.91		27-Apr-21	01-May-21	08-May-21	04-May-21	23-Jun-21
C19IMPACT_0536	Trinidad and Tobago	25-Apr-21	CARPHA	N	N	N	N	N	N	16.08		27-Apr-21	01-May-21	08-May-21	04-May-21	23-Jun-21
C19IMPACT_0537	Trinidad and Tobago	25-Apr-21	CARPHA	N	N	N	N	N	N	15.86		27-Apr-21	01-May-21	08-May-21	04-May-21	23-Jun-21
C19IMPACT_0538	Trinidad and Tobago	27-Apr-21	MoH	N	N	N	N	N	N			29-Apr-21	01-May-21	08-May-21		23-Jun-21
C19IMPACT_0539	Trinidad and Tobago	27-Apr-21	MoH	N	N	N	N	Y	N			29-Apr-21	01-May-21	08-May-21		23-Jun-21
C19IMPACT_0540	Trinidad and Tobago	27-Apr-21	CARPHA	N	N	N	N	N	N	18.20		29-Apr-21	01-May-21	08-May-21	04-May-21	23-Jun-21
C19IMPACT_0541	Trinidad and Tobago	28-Apr-21	MoH	N	N	N	N	N	N			29-Apr-21	01-May-21	08-May-21		23-Jun-21
C19IMPACT_0542	British Virgin Islands	19-Apr-21	CARPHA	N	N	N	N	N	N	15.35		29-Apr-21	02-May-21		04-May-21	23-Jun-21
C19IMPACT_0543	Trinidad and Tobago	26-Apr-21	CARPHA	N	N	N	N	N	N	23.18		29-Apr-21	02-May-21	08-May-21	04-May-21	23-Jun-21
C19IMPACT_0544	Trinidad and Tobago	26-Apr-21	CARPHA	N	N	N	N	N	N	15.11		29-Apr-21	02-May-21	08-May-21	04-May-21	23-Jun-21
C19IMPACT_0545	Trinidad and Tobago	26-Apr-21	CARPHA	N	N	N	N	Y	N	13.90		29-Apr-21	02-May-21	08-May-21	04-May-21	23-Jun-21
C19IMPACT_0546	Trinidad and Tobago	26-Apr-21	CARPHA	N	N	N	N	N	N	18.11		29-Apr-21	02-May-21	08-May-21	04-May-21	23-Jun-21
C19IMPACT_0547	Trinidad and Tobago	26-Apr-21	CARPHA	N	N	N	N	N	N	20.14		29-Apr-21	02-May-21	08-May-21	08-May-21	21-Jan-22
C19IMPACT_0548	Trinidad and Tobago	27-Apr-21	CARPHA	N	N	N	N	Y	N	15.76		29-Apr-21	02-May-21	08-May-21	04-May-21	23-Jun-21
C19IMPACT_0549	Trinidad and Tobago	27-Apr-21	CARPHA	N	N	N	N	N	N	22.78		29-Apr-21	02-May-21	08-May-21	04-May-21	23-Jun-21
C19IMPACT_0550	Trinidad and Tobago	27-Apr-21	CARPHA	N	N	N	N	N	N	17.95		29-Apr-21	02-May-21	08-May-21	04-May-21	23-Jun-21
C19IMPACT_0551	Trinidad and Tobago	27-Apr-21	CARPHA	N	N	N	N	N	N	20.26		29-Apr-21	02-May-21	08-May-21	04-May-21	23-Jun-21
C19IMPACT_0552	Trinidad and Tobago	27-Apr-21	CARPHA	N	N	N	N	N	N	21.36		29-Apr-21	02-May-21	08-May-21	04-May-21	23-Jun-21
C19IMPACT_0553	Trinidad and Tobago	27-Apr-21	CARPHA	N	N	N	N	N	N	18.84		29-Apr-21	02-May-21	08-May-21	04-May-21	23-Jun-21
C19IMPACT_0554	Trinidad and Tobago	27-Apr-21	CARPHA	N	N	N	N	N	N	14.78		29-Apr-21	02-May-21	08-May-21	04-May-21	23-Jun-21
C19IMPACT_0555	Trinidad and Tobago	27-Apr-21	CARPHA	N	N	N	N	Y	N	20.09		29-Apr-21	02-May-21	08-May-21	04-May-21	23-Jun-21
C19IMPACT_0556	Trinidad and Tobago	27-Apr-21	CARPHA	N	N	N	N	Y	N	21.66		29-Apr-21	02-May-21	08-May-21	04-May-21	23-Jun-21
C19IMPACT_0557	Trinidad and Tobago	27-Apr-21	CARPHA	N	N	N	N	Y	N	19.28		29-Apr-21	02-May-21	08-May-21	04-May-21	23-Jun-21
C19IMPACT_0558	Trinidad and Tobago	27-Apr-21	CARPHA	N	N	N	N	N	N	17.72		29-Apr-21	02-May-21	08-May-21	04-May-21	23-Jun-21
C19IMPACT_0559	Trinidad and Tobago	27-Apr-21	CARPHA	N	N	N	N	Y	N	17.26		29-Apr-21	02-May-21	08-May-21	04-May-21	23-Jun-21
C19IMPACT_0560	Trinidad and Tobago	27-Apr-21	CARPHA	N	N	N	N	Y	N	17.02		29-Apr-21	02-May-21	08-May-21	04-May-21	23-Jun-21
C19IMPACT_0561	Trinidad and Tobago	27-Apr-21	CARPHA	N	N	N	N	Y	N	15.86		29-Apr-21	02-May-21	08-May-21	04-May-21	23-Jun-21
C19IMPACT_0562	Trinidad and Tobago	27-Apr-21	CARPHA	N	N	N	N	Y	N	14.85		29-Apr-21	02-May-21	08-May-21	04-May-21	23-Jun-21
C19IMPACT_0563	Trinidad and Tobago	27-Apr-21	CARPHA	N	N	N	N	Y	N	23.61		29-Apr-21	02-May-21	08-May-21	04-May-21	23-Jun-21
C19IMPACT_0564	Trinidad and Tobago	26-Apr-21	CARPHA	N	N	N	N	Y	N	18.23		29-Apr-21	03-May-21	08-May-21	08-May-21	22-Jun-21
C19IMPACT_0565	Trinidad and Tobago	26-Apr-21	CARPHA	N	N	N	N	N	N	19.06		29-Apr-21	03-May-21	08-May-21	08-May-21	22-Jun-21
C19IMPACT_0566	Trinidad and Tobago	26-Apr-21	CARPHA	N	N	N	N	N	N	23.61		29-Apr-21	03-May-21	08-May-21	08-May-21	22-Jun-21
C19IMPACT_0567	Trinidad and Tobago	26-Apr-21	CARPHA	N	N	N	N	N	N	18.72		29-Apr-21	03-May-21	08-May-21	08-May-21	22-Jun-21
C19IMPACT_0568	Trinidad and Tobago	26-Apr-21	CARPHA	N	N	N	N	N	N	19.70		29-Apr-21	03-May-21	08-May-21	08-May-21	22-Jun-21
C19IMPACT_0569	Trinidad and Tobago	26-Apr-21	CARPHA	N	N	N	N	N	N	23.41		29-Apr-21	03-May-21	08-May-21	08-May-21	22-Jun-21
C19IMPACT_0570	Trinidad and Tobago	26-Apr-21	CARPHA	N	N	N	N	N	N	15.44		29-Apr-21	03-May-21	08-May-21	08-May-21	22-Jun-21
C19IMPACT_0571	Trinidad and Tobago	26-Apr-21	CARPHA	N	N	N	N	N	N	16.45		29-Apr-21	03-May-21	13-Oct-21	06-Nov-21	21-Jan-22
C19IMPACT_0572	Trinidad and Tobago	27-Apr-21	CARPHA	N	N	N	N	N	N	21.77		29-Apr-21	03-May-21	08-May-21	08-May-21	22-Jun-21

C19IMPACT_0573	Trinidad and Tobago	27-Apr-21	CARPHA	N	N	N	N	N	N	12.47		29-Apr-21	03-May-21	08-May-21	08-May-21	22-Jun-21
C19IMPACT_0574	Trinidad and Tobago	27-Apr-21	CARPHA	N	N	N	N	Y	N	14.49		29-Apr-21	03-May-21	08-May-21	08-May-21	
C19IMPACT_0575	Trinidad and Tobago	27-Apr-21	CARPHA	N	N	N	N	N	N	22.97		29-Apr-21	03-May-21	08-May-21	08-May-21	
C19IMPACT_0576	Trinidad and Tobago	27-Apr-21	CARPHA	N	N	N	N	N	N	14.18		29-Apr-21	03-May-21	08-May-21	08-May-21	22-Jun-21
C19IMPACT_0577	Trinidad and Tobago	27-Apr-21	CARPHA	N	N	N	N	N	N	19.38		29-Apr-21	03-May-21	08-May-21	08-May-21	22-Jun-21
C19IMPACT_0578	Trinidad and Tobago	27-Apr-21	CARPHA	N	N	N	N	N	N	20.89		29-Apr-21	03-May-21	08-May-21	08-May-21	22-Jun-21
C19IMPACT_0579	Trinidad and Tobago	27-Apr-21	CARPHA	N	N	N	N	N	N	19.73		29-Apr-21	03-May-21	08-May-21	08-May-21	22-Jun-21
C19IMPACT_0580	Trinidad and Tobago	27-Apr-21	CARPHA	N	N	N	N	N	N	21.56		29-Apr-21	03-May-21	08-May-21	08-May-21	22-Jun-21
C19IMPACT_0581	Trinidad and Tobago	27-Apr-21	CARPHA	N	N	N	N	N	N	19.49		29-Apr-21	03-May-21	08-May-21	08-May-21	22-Jun-21
C19IMPACT_0582	Trinidad and Tobago	27-Apr-21	CARPHA	N	N	N	N	Y	N	16.18		29-Apr-21	03-May-21	08-May-21	08-May-21	22-Jun-21
C19IMPACT_0583	Trinidad and Tobago	27-Apr-21	CARPHA	N	N	N	N	Y	N	19.60		29-Apr-21	03-May-21	08-May-21	08-May-21	22-Jun-21
C19IMPACT_0584	Trinidad and Tobago	28-Apr-21	CARPHA	N	N	N	N	N	N	13.76		29-Apr-21	03-May-21	08-May-21	08-May-21	22-Jun-21
C19IMPACT_0585	Trinidad and Tobago	28-Apr-21	CARPHA	N	N	N	N	N	N	22.90		29-Apr-21	03-May-21	08-May-21	08-May-21	22-Jun-21
C19IMPACT_0586	Trinidad and Tobago	26-Apr-21	CARPHA	N	N	N	N	N	Y	20.28		29-Apr-21	05-May-21	08-May-21	08-May-21	22-Jun-21
C19IMPACT_0587	Trinidad and Tobago	26-Apr-21	CARPHA	N	N	N	N	Y	N	19.98		29-Apr-21	05-May-21	08-May-21	08-May-21	22-Jun-21
C19IMPACT_0588	Trinidad and Tobago	26-Apr-21	CARPHA	N	N	N	N	Y	Y	22.74		29-Apr-21	05-May-21	08-May-21	08-May-21	22-Jun-21
C19IMPACT_0589	Trinidad and Tobago	26-Apr-21	CARPHA	N	N	N	N	Y	Y	15.78		29-Apr-21	05-May-21	08-May-21	08-May-21	22-Jun-21
C19IMPACT_0590	Trinidad and Tobago	26-Apr-21	CARPHA	N	N	N	N	N	Y	22.28		29-Apr-21	05-May-21	08-May-21	08-May-21	22-Jun-21
C19IMPACT_0591	Trinidad and Tobago	26-Apr-21	CARPHA	N	N	N	N	Y	Y	13.98		29-Apr-21	05-May-21	08-May-21	08-May-21	22-Jun-21
C19IMPACT_0592	Trinidad and Tobago	26-Apr-21	CARPHA	N	N	N	N	N	Y	17.87		29-Apr-21	05-May-21	08-May-21	08-May-21	22-Jun-21
C19IMPACT_0593	Trinidad and Tobago	26-Apr-21	CARPHA	N	N	N	N	Y	Y	18.06		29-Apr-21	05-May-21	08-May-21	08-May-21	22-Jun-21
C19IMPACT_0594	Trinidad and Tobago	26-Apr-21	CARPHA	N	N	N	N	Y	Y	16.48		29-Apr-21	05-May-21	08-May-21	08-May-21	22-Jun-21
C19IMPACT_0595	Trinidad and Tobago	26-Apr-21	CARPHA	N	N	N	N	Y	Y	24.40		29-Apr-21	05-May-21	08-May-21	08-May-21	
C19IMPACT_0596	Trinidad and Tobago	26-Apr-21	CARPHA	N	N	N	N	Y	Y	21.95		29-Apr-21	05-May-21	08-May-21	08-May-21	22-Jun-21
C19IMPACT_0597	Trinidad and Tobago	26-Apr-21	CARPHA	N	N	N	N	Y	Y	23.94		29-Apr-21	05-May-21	08-May-21	08-May-21	22-Jun-21
C19IMPACT_0598	Trinidad and Tobago	26-Apr-21	CARPHA	N	N	N	N	Y	Y	16.61		29-Apr-21	05-May-21	08-May-21	08-May-21	22-Jun-21
C19IMPACT_0599	Trinidad and Tobago	26-Apr-21	CARPHA	N	N	N	N	Y	Y	21.87		29-Apr-21	05-May-21	08-May-21	08-May-21	22-Jun-21
C19IMPACT_0600	Trinidad and Tobago	26-Apr-21	CARPHA	N	N	N	N	Y	Y	24.57		29-Apr-21	05-May-21	08-May-21	08-May-21	
C19IMPACT_0601	Trinidad and Tobago	26-Apr-21	CARPHA	N	N	N	N	Y	Y	17.09		29-Apr-21	05-May-21	08-May-21	08-May-21	22-Jun-21
C19IMPACT_0602	Trinidad and Tobago	26-Apr-21	CARPHA	N	N	N	N	N	Y	21.96		29-Apr-21	05-May-21	08-May-21	08-May-21	22-Jun-21
C19IMPACT_0603	Trinidad and Tobago	26-Apr-21	CARPHA	N	N	N	N	N	Y	22.22		29-Apr-21	05-May-21	08-May-21	08-May-21	22-Jun-21
C19IMPACT_0604	Trinidad and Tobago	28-Apr-21	MoH	N	N	N	N	N	Y	21.38		29-Apr-21	05-May-21	08-May-21		22-Jun-21
C19IMPACT_0605	Trinidad and Tobago	28-Apr-21	MoH	N	N	N	N	Y	N	21.07		29-Apr-21	05-May-21	08-May-21		22-Jun-21
C19IMPACT_0606	Trinidad and Tobago	28-Apr-21	MoH	N	N	N	N	Y	Y	23.42		29-Apr-21	05-May-21	08-May-21		22-Jun-21
C19IMPACT_0607	Trinidad and Tobago	28-Apr-21	MoH	N	N	N	N	Y	Y	23.95		29-Apr-21	05-May-21	08-May-21		22-Jun-21
C19IMPACT_0608	Trinidad and Tobago	26-Apr-21	CARPHA	N	N	N	N	Y	N	24.87		30-Apr-21	06-May-21	08-May-21	08-May-21	
C19IMPACT_0609	Trinidad and Tobago	26-Apr-21	CARPHA	N	N	N	N	N	N	16.39		30-Apr-21	06-May-21	08-May-21	08-May-21	22-Jun-21
C19IMPACT_0610	Trinidad and Tobago	26-Apr-21	CARPHA	N	N	N	N	N	N	23.12		30-Apr-21	06-May-21	08-May-21	08-May-21	
C19IMPACT_0611	Trinidad and Tobago	26-Apr-21	CARPHA	N	N	N	N	N	N	20.96		30-Apr-21	06-May-21	08-May-21	08-May-21	22-Jun-21
C19IMPACT_0612	Trinidad and Tobago	27-Apr-21	CARPHA	N	N	N	N	N	N	19.56		30-Apr-21	06-May-21	08-May-21	08-May-21	22-Jun-21
C19IMPACT_0613	Trinidad and Tobago	27-Apr-21	CARPHA	N	N	N	N	Y	N	21.72		30-Apr-21	06-May-21	08-May-21	08-May-21	22-Jun-21
C19IMPACT_0614	Trinidad and Tobago	27-Apr-21	CARPHA	N	N	N	N	Y	N	15.97		30-Apr-21	06-May-21	08-May-21	08-May-21	22-Jun-21
C19IMPACT_0615	Trinidad and Tobago	27-Apr-21	CARPHA	N	N	N	N	Y	N	23.28		30-Apr-21	06-May-21	08-May-21	08-May-21	22-Jun-21
C19IMPACT_0616	Trinidad and Tobago	27-Apr-21	CARPHA	N	N	N	N	N	N	19.69		30-Apr-21	06-May-21	08-May-21	08-May-21	22-Jun-21
C19IMPACT_0617	Trinidad and Tobago	27-Apr-21	CARPHA	N	N	N	N	N	N	17.96		30-Apr-21	06-May-21	08-May-21	08-May-21	22-Jun-21
C19IMPACT_0618	Trinidad and Tobago	27-Apr-21	CARPHA	N	N	N	N	N	N	21.24		30-Apr-21	06-May-21	08-May-21	08-May-21	22-Jun-21
C19IMPACT_0619	Trinidad and Tobago	27-Apr-21	CARPHA	N	N	N	N	N	N	24.22		30-Apr-21	06-May-21	08-May-21	08-May-21	
C19IMPACT_0620	Trinidad and Tobago	27-Apr-21	CARPHA	N	N	N	N	N	N	17.56		30-Apr-21	06-May-21	08-May-21	08-May-21	22-Jun-21
C19IMPACT_0621	Trinidad and Tobago	27-Apr-21	CARPHA	N	N	N	N	N	N	19.73		30-Apr-21	06-May-21	08-May-21	08-May-21	22-Jun-21
C19IMPACT_0622	Trinidad and Tobago	27-Apr-21	CARPHA	N	N	N	N	N	N	16.83		30-Apr-21	06-May-21	08-May-21	08-May-21	22-Jun-21
C19IMPACT_0623	Trinidad and Tobago	28-Apr-21	CARPHA	N	N	N	N	N	N	23.12		30-Apr-21	06-May-21	08-May-21	08-May-21	22-Jun-21
C19IMPACT_0624	Trinidad and Tobago	28-Apr-21	CARPHA	N	N	N	N	N	N	22.45		30-Apr-21	06-May-21	08-May-21	08-May-21	
C19IMPACT_0625	Trinidad and Tobago	28-Apr-21	CARPHA	N	N	N	N	N	N	18.31		30-Apr-21	06-May-21	08-May-21	08-May-21	22-Jun-21
C19IMPACT_0626	Trinidad and Tobago	28-Apr-21	CARPHA	N	N	N	N	N	N	23.80		30-Apr-21	06-May-21	08-May-21	08-May-21	
C19IMPACT_0627	Trinidad and Tobago	28-Apr-21	CARPHA	N	N	N	N	N	N	19.37		30-Apr-21	06-May-21	08-May-21	08-May-21	22-Jun-21
C19IMPACT_0628	Trinidad and Tobago	28-Apr-21	CARPHA	N	N	N	N	N	N	16.65		30-Apr-21	06-May-21	08-May-21	08-May-21	22-Jun-21
C19IMPACT_0629	Trinidad and Tobago	28-Apr-21	CARPHA	N	N	N	N	N	N	18.56		30-Apr-21	06-May-21	08-May-21	08-May-21	22-Jun-21
C19IMPACT_0630	Dominica	09-Mar-21	CARPHA	N	N	N	N	N	N	19.96		04-May-21	10-May-21		10-May-21	21-Jun-21
C19IMPACT_0631	Dominica	21-Mar-21	CARPHA	N	N	N	N	N	N	18.55		04-May-21	10-May-21		10-May-21	21-Jun-21
C19IMPACT_0632	Dominica	01-Apr-21	CARPHA	N	N	N	N	N	N	19.28		04-May-21	10-May-21		10-May-21	21-Jun-21
C19IMPACT_0633	Trinidad and Tobago	27-Apr-21	CARPHA	N	N	N	N	N	N	21.59		30-Apr-21	10-May-21	13-Oct-21	10-May-21	21-Jun-21
C19IMPACT_0634	Trinidad and Tobago	27-Apr-21	CARPHA	N	N	N	N	N	N	17.32		30-Apr-21	10-May-21	13-Oct-21	10-May-21	21-Jun-21
C19IMPACT_0635	Trinidad and Tobago	27-Apr-21	CARPHA	N	N	N	N	N	N	24.30		30-Apr-21	10-May-21	13-Oct-21	10-May-21	21-Jun-21
C19IMPACT_0636	Trinidad and Tobago	28-Apr-21	CARPHA	N	N	N	N	N	N	19.63		30-Apr-21	10-May-21	13-Oct-21	10-May-21	21-Jun-21

C19IMPACT_0637	Trinidad and Tobago	28-Apr-21	CARPHA	N	N	N	N	N	N	20.39		30-Apr-21	10-May-21	13-Oct-21	10-May-21	21-Jun-21
C19IMPACT_0638	Trinidad and Tobago	28-Apr-21	CARPHA	N	N	N	N	N	N	15.33		30-Apr-21	10-May-21	13-Oct-21	10-May-21	21-Jun-21
C19IMPACT_0639	Trinidad and Tobago	29-Apr-21	CARPHA	N	N	N	N	N	N	25.01		30-Apr-21	10-May-21	14-May-21	10-May-21	
C19IMPACT_0640	Trinidad and Tobago	29-Apr-21	CARPHA	N	N	N	N	N	N	18.37		04-May-21	10-May-21	14-May-21	10-May-21	21-Jun-21
C19IMPACT_0641	Trinidad and Tobago	29-Apr-21	CARPHA	N	N	N	N	N	N	18.64		04-May-21	10-May-21	14-May-21	10-May-21	21-Jun-21
C19IMPACT_0642	Trinidad and Tobago	30-Apr-21	MoH	N	N	N	N	N	N	15.68		03-May-21	10-May-21	14-May-21		21-Jun-21
C19IMPACT_0643	Trinidad and Tobago	30-Apr-21	MoH	N	N	N	N	Y	Y			03-May-21	10-May-21	14-May-21		21-Jun-21
C19IMPACT_0644	Trinidad and Tobago	30-Apr-21	CARPHA	N	N	N	N	N	N	22.93		04-May-21	10-May-21	14-May-21	10-May-21	21-Jun-21
C19IMPACT_0645	Trinidad and Tobago	30-Apr-21	CARPHA	N	N	N	N	Y	N	17.33		04-May-21	10-May-21	14-May-21	10-May-21	21-Jun-21
C19IMPACT_0646	Trinidad and Tobago	30-Apr-21	CARPHA	N	N	N	N	Y	N	21.55		04-May-21	10-May-21	14-May-21	10-May-21	21-Jun-21
C19IMPACT_0647	Trinidad and Tobago	30-Apr-21	CARPHA	N	N	N	N	Y	N	18.57		04-May-21	10-May-21	14-May-21	10-May-21	21-Jun-21
C19IMPACT_0648	Trinidad and Tobago	30-Apr-21	CARPHA	N	N	N	N	N	N	22.43		04-May-21	10-May-21	14-May-21	10-May-21	21-Jun-21
C19IMPACT_0649	Trinidad and Tobago	30-Apr-21	CARPHA	N	N	N	N	N	N	24.82		04-May-21	10-May-21	14-May-21	10-May-21	21-Jun-21
C19IMPACT_0650	Dominica		CARPHA	N	N	N	N	N	N	23.89		04-May-21	10-May-21		10-May-21	
C19IMPACT_0651	Trinidad and Tobago		CARPHA	N	N	N	N	N	Y				10-May-21	14-May-21	10-May-21	
C19IMPACT_0652	Trinidad and Tobago	27-Apr-21	CARPHA	N	N	N	N	N	N	19.63		04-May-21	11-May-21	03-Dec-21	03-Dec-21	01-Dec-21
C19IMPACT_0653	Trinidad and Tobago	28-Apr-21	CARPHA	N	N	N	N	N	N	16.08		04-May-21	11-May-21	24-May-21	18-May-21	21-Jun-21
C19IMPACT_0654	Trinidad and Tobago	28-Apr-21	CARPHA	N	N	N	N	N	N	23.30		04-May-21	11-May-21	24-May-21	18-May-21	21-Jun-21
C19IMPACT_0655	Trinidad and Tobago	28-Apr-21	CARPHA	N	N	N	N	N	N	13.99		04-May-21	11-May-21	24-May-21	18-May-21	21-Jun-21
C19IMPACT_0656	Trinidad and Tobago	28-Apr-21	CARPHA	N	N	N	N	N	N	16.19		04-May-21	11-May-21	24-May-21	18-May-21	21-Jun-21
C19IMPACT_0657	Trinidad and Tobago	28-Apr-21	CARPHA	N	N	N	N	N	N	16.34		04-May-21	11-May-21	24-May-21	18-May-21	21-Jun-21
C19IMPACT_0658	Trinidad and Tobago	28-Apr-21	CARPHA	N	N	N	N	N	N	22.66		04-May-21	11-May-21	24-May-21	18-May-21	21-Jun-21
C19IMPACT_0659	Trinidad and Tobago	28-Apr-21	CARPHA	N	N	N	N	N	N	15.79		04-May-21	11-May-21	24-May-21	18-May-21	21-Jun-21
C19IMPACT_0660	Trinidad and Tobago	28-Apr-21	CARPHA	N	N	N	N	N	N	22.45		04-May-21	11-May-21	24-May-21	18-May-21	21-Jun-21
C19IMPACT_0661	Trinidad and Tobago	28-Apr-21	CARPHA	N	N	N	N	N	N	16.29		04-May-21	11-May-21	24-May-21	18-May-21	21-Jun-21
C19IMPACT_0662	Trinidad and Tobago	28-Apr-21	CARPHA	N	N	N	N	N	N	22.88		04-May-21	11-May-21	24-May-21	18-May-21	21-Jun-21
C19IMPACT_0663	Trinidad and Tobago	28-Apr-21	CARPHA	N	N	N	N	N	N	20.35		04-May-21	11-May-21	24-May-21	18-May-21	21-Jun-21
C19IMPACT_0664	Trinidad and Tobago	28-Apr-21	CARPHA	N	N	N	N	N	N	21.86		04-May-21	11-May-21	24-May-21	18-May-21	21-Jun-21
C19IMPACT_0665	Trinidad and Tobago	28-Apr-21	CARPHA	N	N	N	N	N	N	16.93		04-May-21	11-May-21	03-Dec-21	03-Dec-21	01-Dec-21
C19IMPACT_0666	Trinidad and Tobago	28-Apr-21	CARPHA	N	N	N	N	N	Y	21.42		04-May-21	11-May-21	03-Dec-21	03-Dec-21	01-Dec-21
C19IMPACT_0667	Trinidad and Tobago	28-Apr-21	CARPHA	N	N	N	N	N	N	19.04		04-May-21	11-May-21	03-Dec-21	03-Dec-21	01-Dec-21
C19IMPACT_0668	Trinidad and Tobago	28-Apr-21	CARPHA	N	N	N	N	N	N	22.66		04-May-21	11-May-21	03-Dec-21	03-Dec-21	01-Dec-21
C19IMPACT_0669	Trinidad and Tobago	28-Apr-21	CARPHA	N	N	N	N	N	N	16.43		04-May-21	11-May-21	03-Dec-21	03-Dec-21	01-Dec-21
C19IMPACT_0670	Trinidad and Tobago	01-May-21	CARPHA	N	N	N	N	N	N	15.30		06-May-21	11-May-21	24-May-21	18-May-21	21-Jun-21
C19IMPACT_0671	Trinidad and Tobago	01-May-21	CARPHA	N	N	N	N	N	N	17.65		06-May-21	11-May-21	24-May-21	18-May-21	21-Jun-21
C19IMPACT_0672	Trinidad and Tobago	01-May-21	CARPHA	N	N	N	N	N	N	24.27		06-May-21	11-May-21	24-May-21	18-May-21	
C19IMPACT_0673	Trinidad and Tobago	01-May-21	CARPHA	N	N	N	N	N	N	18.05		06-May-21	11-May-21	24-May-21	18-May-21	21-Jun-21
C19IMPACT_0674	Saint Lucia	27-Apr-21	CARPHA	N	N	N	N	N	N	19.14		07-May-21	14-May-21		18-May-21	21-Jun-21
C19IMPACT_0675	Saint Lucia	27-Apr-21	CARPHA	N	N	N	N	N	N	16.99		07-May-21	14-May-21		18-May-21	21-Jun-21
C19IMPACT_0676	Saint Lucia	27-Apr-21	CARPHA	N	N	N	N	N	N	17.05		07-May-21	14-May-21		18-May-21	21-Jun-21
C19IMPACT_0677	Saint Lucia	27-Apr-21	CARPHA	N	N	N	N	N	N	21.29		07-May-21	14-May-21		18-May-21	
C19IMPACT_0678	Saint Lucia	27-Apr-21	CARPHA	N	N	N	N	N	N	17.40		07-May-21	14-May-21		18-May-21	
C19IMPACT_0679	Saint Lucia	28-Apr-21	CARPHA	N	N	N	N	N	N	12.92		07-May-21	14-May-21		18-May-21	21-Jun-21
C19IMPACT_0680	Trinidad and Tobago	28-Apr-21	CARPHA	N	N	N	N	N	N	21.60		06-May-21	14-May-21	24-May-21	18-May-21	21-Jun-21
C19IMPACT_0681	Saint Lucia	29-Apr-21	CARPHA	N	N	N	N	Y	N	33.58		07-May-21	14-May-21		18-May-21	
C19IMPACT_0682	Saint Lucia	29-Apr-21	CARPHA	N	N	N	N	N	N	14.57		07-May-21	14-May-21		18-May-21	21-Jun-21
C19IMPACT_0683	Saint Lucia	29-Apr-21	CARPHA	N	N	N	N	N	N	13.81		07-May-21	14-May-21		18-May-21	21-Jun-21
C19IMPACT_0684	Saint Lucia	29-Apr-21	CARPHA	N	N	N	N	N	N	15.27		07-May-21	14-May-21		18-May-21	21-Jun-21
C19IMPACT_0685	Saint Lucia	29-Apr-21	CARPHA	N	N	N	N	N	N	12.18		07-May-21	14-May-21		18-May-21	21-Jun-21
C19IMPACT_0686	Trinidad and Tobago	30-Apr-21	CARPHA	N	N	N	N	N	N	18.98		06-May-21	14-May-21	24-May-21	18-May-21	21-Jun-21
C19IMPACT_0687	Trinidad and Tobago	30-Apr-21	CARPHA	N	N	N	N	N	N	22.22		06-May-21	14-May-21	24-May-21	18-May-21	
C19IMPACT_0688	Trinidad and Tobago	30-Apr-21	CARPHA	N	N	N	N	Y	N	15.99		06-May-21	14-May-21	24-May-21	18-May-21	21-Jun-21
C19IMPACT_0689	Trinidad and Tobago	01-May-21	CARPHA	N	N	N	N	Y	N	20.77		06-May-21	14-May-21	24-May-21	18-May-21	21-Jun-21
C19IMPACT_0690	Trinidad and Tobago	01-May-21	CARPHA	N	N	N	N	N	N	17.84		06-May-21	14-May-21	24-May-21	18-May-21	21-Jun-21
C19IMPACT_0691	Trinidad and Tobago	01-May-21	CARPHA	N	N	N	N	N	N	20.83		06-May-21	14-May-21	24-May-21	18-May-21	21-Jun-21
C19IMPACT_0692	Trinidad and Tobago	01-May-21	CARPHA	N	N	N	N	N	N	18.15		06-May-21	14-May-21	24-May-21	18-May-21	21-Jun-21
C19IMPACT_0693	Trinidad and Tobago	02-May-21	CARPHA	N	N	N	N	N	N	21.27		06-May-21	14-May-21	24-May-21	18-May-21	21-Jun-21
C19IMPACT_0694	Trinidad and Tobago	02-May-21	CARPHA	N	N	N	N	N	N	19.33		06-May-21	14-May-21	24-May-21	18-May-21	21-Jun-21
C19IMPACT_0695	Trinidad and Tobago	02-May-21	CARPHA	N	N	N	N	N	N	20.11		06-May-21	14-May-21	13-Oct-21	06-Nov-21	21-Jan-22
C19IMPACT_0696	Trinidad and Tobago	30-Apr-21	CARPHA	N	N	N	N	N	N	23.83		06-May-21	19-May-21	24-May-21	21-May-21	20-Jun-21
C19IMPACT_0697	Trinidad and Tobago	01-May-21	CARPHA	N	N	N	N	Y	N	24.55		06-May-21	19-May-21	24-May-21	21-May-21	20-Jun-21
C19IMPACT_0698	Trinidad and Tobago	01-May-21	CARPHA	N	N	N	N	N	N	18.95		06-May-21	19-May-21	24-May-21	21-May-21	20-Jun-21
C19IMPACT_0699	Trinidad and Tobago	02-May-21	CARPHA	N	N	N	N	Y	N	22.14		06-May-21	19-May-21	24-May-21	21-May-21	20-Jun-21
C19IMPACT_0700	Trinidad and Tobago	02-May-21	CARPHA	N	N	N	N	N	N	20.02		06-May-21	19-May-21	24-May-21	21-May-21	20-Jun-21

C19IMPACT_0701	Trinidad and Tobago	03-May-21	CARPHA	N	N	N	N	Y	N	18.62		06-May-21	19-May-21	24-May-21	21-May-21	20-Jun-21
C19IMPACT_0702	Trinidad and Tobago	03-May-21	CARPHA	N	N	N	N	N	N	13.23		06-May-21	19-May-21	24-May-21	21-May-21	20-Jun-21
C19IMPACT_0703	Trinidad and Tobago	03-May-21	CARPHA	N	N	N	N	N	N	13.93		06-May-21	19-May-21	24-May-21	21-May-21	20-Jun-21
C19IMPACT_0704	Trinidad and Tobago	03-May-21	CARPHA	N	N	N	N	N	N	20.72		06-May-21	19-May-21	24-May-21	21-May-21	20-Jun-21
C19IMPACT_0705	Trinidad and Tobago	03-May-21	CARPHA	N	N	N	N	N	N	19.54		06-May-21	19-May-21	24-May-21	21-May-21	20-Jun-21
C19IMPACT_0706	Trinidad and Tobago	03-May-21	CARPHA	N	N	N	N	N	N	17.88		06-May-21	19-May-21	24-May-21	21-May-21	20-Jun-21
C19IMPACT_0707	Trinidad and Tobago	03-May-21	CARPHA	N	N	N	N	Y	N	23.59		06-May-21	19-May-21	24-May-21	21-May-21	20-Jun-21
C19IMPACT_0708	Trinidad and Tobago	03-May-21	CARPHA	N	N	N	N	Y	N	17.64		06-May-21	19-May-21	24-May-21	21-May-21	20-Jun-21
C19IMPACT_0709	Trinidad and Tobago	03-May-21	CARPHA	N	N	N	N	Y	N	22.12		06-May-21	19-May-21	24-May-21	21-May-21	20-Jun-21
C19IMPACT_0710	Trinidad and Tobago	03-May-21	CARPHA	N	N	N	N	Y	N	22.83		06-May-21	19-May-21	24-May-21	21-May-21	20-Jun-21
C19IMPACT_0711	Trinidad and Tobago	03-May-21	CARPHA	N	N	N	N	Y	N	12.36		06-May-21	19-May-21	24-May-21	21-May-21	20-Jun-21
C19IMPACT_0712	Trinidad and Tobago	03-May-21	CARPHA	N	N	N	N	Y	N	19.96		06-May-21	19-May-21	24-May-21	21-May-21	20-Jun-21
C19IMPACT_0713	Barbados	04-May-21	CARPHA	N	N	N	N	Y	N	17.27		10-May-21	19-May-21		21-May-21	20-Jun-21
C19IMPACT_0714	Barbados	04-May-21	CARPHA	N	N	N	N	N	N	16.34		10-May-21	19-May-21		21-May-21	20-Jun-21
C19IMPACT_0715	Trinidad and Tobago	04-May-21	CARPHA	N	N	N	N	N	N	21.70		06-May-21	19-May-21	24-May-21	05-Jun-21	20-Jun-21
C19IMPACT_0716	Trinidad and Tobago	04-May-21	CARPHA	N	N	N	N	N	N	23.73		06-May-21	19-May-21	24-May-21	05-Jun-21	20-Jun-21
C19IMPACT_0717	Trinidad and Tobago	04-May-21	CARPHA	N	N	N	N	N	N	18.61		06-May-21	19-May-21	24-May-21	21-May-21	20-Jun-21
C19IMPACT_0718	Trinidad and Tobago		MoH	N	N	N	N	N	Y	18.00		07-May-21	21-May-21	28-May-21		21-Jan-22
C19IMPACT_0719	Trinidad and Tobago	04-May-21	MoH	Y	N	Y	N	N	N			07-May-21	21-May-21	28-May-21		21-Jan-22
C19IMPACT_0720	Trinidad and Tobago	05-May-21	MoH	Y	N	Y	N	N	N	19.00		07-May-21	21-May-21	28-May-21		21-Jan-22
C19IMPACT_0721	Trinidad and Tobago	05-May-21	MoH	Y	N	Y	N	N	N	17.00		07-May-21	21-May-21	28-May-21		21-Jan-22
C19IMPACT_0722	Trinidad and Tobago	05-May-21	MoH	Y	N	N	N	N	N	17.00		07-May-21	21-May-21	28-May-21		21-Jan-22
C19IMPACT_0723	Trinidad and Tobago	05-May-21	MoH	Y	N	Y	N	N	N	22.00		07-May-21	21-May-21	28-May-21		21-Jan-22
C19IMPACT_0724	Trinidad and Tobago	05-May-21	MoH	Y	N	Y	N	N	N	17.00		07-May-21	21-May-21	28-May-21		21-Jan-22
C19IMPACT_0725	Trinidad and Tobago	05-May-21	MoH	Y	N	Y	N	N	N	21.00		07-May-21	21-May-21	28-May-21		21-Jan-22
C19IMPACT_0726	Trinidad and Tobago	05-May-21	MoH	Y	N	Y	N	N	N	16.00		07-May-21	21-May-21	28-May-21		21-Jan-22
C19IMPACT_0727	Trinidad and Tobago	05-May-21	MoH	Y	N	Y	N	N	N	20.00		07-May-21	21-May-21	28-May-21		21-Jan-22
C19IMPACT_0728	Trinidad and Tobago	05-May-21	MoH	Y	N	Y	N	N	N	22.00		07-May-21	21-May-21	28-May-21		21-Jan-22
C19IMPACT_0729	Trinidad and Tobago	05-May-21	MoH	Y	N	Y	N	N	N	20.00		07-May-21	21-May-21	28-May-21		21-Jan-22
C19IMPACT_0730	Trinidad and Tobago	05-May-21	MoH	Y	N	Y	N	N	N	19.00		07-May-21	21-May-21	28-May-21		21-Jan-22
C19IMPACT_0731	Trinidad and Tobago	05-May-21	MoH	Y	N	Y	N	N	N	18.00		07-May-21	21-May-21	28-May-21		21-Jan-22
C19IMPACT_0732	Trinidad and Tobago	05-May-21	MoH	Y	N	Y	N	N	N			07-May-21	21-May-21	28-May-21		21-Jan-22
C19IMPACT_0733	Trinidad and Tobago	05-May-21	MoH	N	N	Y	N	N	N	16.00		07-May-21	21-May-21	28-May-21		21-Jan-22
C19IMPACT_0734	Trinidad and Tobago	05-May-21	MoH	Y	N	Y	N	N	N	19.00		07-May-21	21-May-21	28-May-21		21-Jan-22
C19IMPACT_0735	Trinidad and Tobago		MoH	N	N	N	N	N	N	19.00		07-May-21	21-May-21	28-May-21		
C19IMPACT_0736	Trinidad and Tobago		MoH	N	N	N	N	N	N	21.00		07-May-21	21-May-21	28-May-21		
C19IMPACT_0737	Trinidad and Tobago		MoH	N	N	N	N	N	N	17.00		07-May-21	21-May-21	28-May-21		
C19IMPACT_0738	Trinidad and Tobago		MoH	N	N	N	N	N	Y			07-May-21	21-May-21	28-May-21		
C19IMPACT_0739	Trinidad and Tobago	30-Apr-21	MoH	Y	N	Y	N	N	Y		8.36	07-May-21	26-May-21	28-May-21		21-Jan-22
C19IMPACT_0740	Trinidad and Tobago	02-May-21	CARPHA	Y	N	Y	N	N	N	15.78		06-May-21	26-May-21	28-May-21	05-Jun-21	21-Jan-22
C19IMPACT_0741	Trinidad and Tobago	02-May-21	CARPHA	Y	N	Y	N	Y	N	21.15		06-May-21	26-May-21	28-May-21	05-Jun-21	21-Jan-22
C19IMPACT_0742	Trinidad and Tobago	02-May-21	CARPHA	N	N	N	N	Y	N	15.33		06-May-21	26-May-21	28-May-21	05-Jun-21	21-Jan-22
C19IMPACT_0743	Trinidad and Tobago	03-May-21	CARPHA	N	N	N	N	Y	N	24.76		06-May-21	26-May-21	28-May-21	05-Jun-21	21-Jan-22
C19IMPACT_0744	Trinidad and Tobago	04-May-21	CARPHA	N	N	N	N	N	N	17.45		10-May-21	26-May-21	28-May-21	05-Jun-21	21-Jan-22
C19IMPACT_0745	Trinidad and Tobago	04-May-21	MoH	N	N	N	N	Y	N	19.00		07-May-21	26-May-21	28-May-21		21-Jan-22
C19IMPACT_0746	Trinidad and Tobago	04-May-21	MoH	Y	N	Y	N	N	N			07-May-21	26-May-21	28-May-21		21-Jan-22
C19IMPACT_0747	Trinidad and Tobago	05-May-21	CARPHA	N	N	N	N	N	N	22.70		10-May-21	26-May-21	28-May-21	05-Jun-21	21-Jan-22
C19IMPACT_0748	Trinidad and Tobago	05-May-21	CARPHA	N	N	N	N	Y	N	15.80		10-May-21	26-May-21	28-May-21	05-Jun-21	21-Jan-22
C19IMPACT_0749	Trinidad and Tobago	06-May-21	CARPHA	N	N	N	N	Y	N	21.97		10-May-21	26-May-21	28-May-21	05-Jun-21	21-Jan-22
C19IMPACT_0750	Trinidad and Tobago	06-May-21	CARPHA	N	N	N	N	N	N	13.13		10-May-21	26-May-21	28-May-21	05-Jun-21	21-Jan-22
C19IMPACT_0751	Trinidad and Tobago	06-May-21	CARPHA	N	N	N	N	N	N	19.63		10-May-21	26-May-21	28-May-21	05-Jun-21	21-Jan-22
C19IMPACT_0752	Trinidad and Tobago	06-May-21	CARPHA	N	N	N	N	N	N	18.98		10-May-21	26-May-21	28-May-21	05-Jun-21	21-Jan-22
C19IMPACT_0753	Trinidad and Tobago	07-May-21	CARPHA	N	N	N	N	Y	Y	21.34		10-May-21	26-May-21	28-May-21	05-Jun-21	21-Jan-22
C19IMPACT_0754	Trinidad and Tobago		MoH	Y	N	Y	N	N	N	15.00		07-May-21	26-May-21	28-May-21		
C19IMPACT_0755	Trinidad and Tobago		MoH	Y	N	N	N	N	N	21.00		07-May-21	26-May-21	28-May-21		
C19IMPACT_0756	Trinidad and Tobago		MoH	Y	N	Y	N	N	N	21.00		07-May-21	26-May-21	28-May-21		
C19IMPACT_0757	Trinidad and Tobago		MoH	Y	N	Y	N	N	N	16.00		07-May-21	26-May-21	28-May-21		
C19IMPACT_0758	Trinidad and Tobago		MoH	N	N	N	N	N	N	17.00		07-May-21	26-May-21	28-May-21		
C19IMPACT_0759	Trinidad and Tobago		MoH	N	N	N	N	N	N	18.00		07-May-21	26-May-21	28-May-21		
C19IMPACT_0760	Trinidad and Tobago		MoH	N	N	N	N	N	N	21.00		07-May-21	26-May-21	28-May-21		
C19IMPACT_0761	Trinidad and Tobago	04-May-21	CARPHA	N	N	N	N	N	N	17.64		17-May-21	27-May-21	05-Jun-21	05-Jun-21	21-Jan-22
C19IMPACT_0762	Trinidad and Tobago	10-May-21	CARPHA	N	N	N	N	N	N	19.70		17-May-21	27-May-21	05-Jun-21	05-Jun-21	21-Jan-22
C19IMPACT_0763	Trinidad and Tobago	10-May-21	CARPHA	N	N	N	N	Y	N	15.52		17-May-21	27-May-21	05-Jun-21	05-Jun-21	21-Jan-22
C19IMPACT_0764	Trinidad and Tobago	10-May-21	CARPHA	N	N	N	N	Y	N	23.95		17-May-21	27-May-21	05-Jun-21	05-Jun-21	21-Jan-22

C19IMPACT_0765	Trinidad and Tobago	10-May-21	CARPHA	N	N	N	N	N	N	19.98		17-May-21	27-May-21	05-Jun-21	05-Jun-21	21-Jan-22
C19IMPACT_0766	Trinidad and Tobago	10-May-21	CARPHA	N	N	N	N	N	N	16.13		17-May-21	27-May-21	05-Jun-21	05-Jun-21	21-Jan-22
C19IMPACT_0767	Trinidad and Tobago	10-May-21	CARPHA	N	N	N	N	Y	N	20.32		17-May-21	27-May-21	05-Jun-21	05-Jun-21	21-Jan-22
C19IMPACT_0768	Trinidad and Tobago	10-May-21	CARPHA	N	N	N	N	Y	N	16.30		17-May-21	27-May-21	05-Jun-21	05-Jun-21	21-Jan-22
C19IMPACT_0769	Trinidad and Tobago	10-May-21	CARPHA	N	N	N	N	Y	N	20.68		17-May-21	27-May-21	05-Jun-21	05-Jun-21	21-Jan-22
C19IMPACT_0770	Trinidad and Tobago	10-May-21	CARPHA	N	N	N	N	Y	N	18.82		17-May-21	27-May-21	05-Jun-21	05-Jun-21	21-Jan-22
C19IMPACT_0771	Trinidad and Tobago	10-May-21	CARPHA	N	N	N	N	Y	N	17.46		17-May-21	27-May-21	05-Jun-21	05-Jun-21	21-Jan-22
C19IMPACT_0772	Trinidad and Tobago	10-May-21	CARPHA	N	N	N	N	Y	N	22.55		17-May-21	27-May-21	13-Oct-21	06-Nov-21	
C19IMPACT_0773	Trinidad and Tobago	10-May-21	CARPHA	N	N	N	N	N	N	21.10		17-May-21	27-May-21	13-Oct-21	06-Nov-21	
C19IMPACT_0774	Trinidad and Tobago	11-May-21	CARPHA	N	N	N	N	N	N	21.13		17-May-21	27-May-21	05-Jun-21	05-Jun-21	21-Jan-22
C19IMPACT_0775	Trinidad and Tobago	11-May-21	CARPHA	N	N	N	N	Y	N	19.69		17-May-21	27-May-21	05-Jun-21	05-Jun-21	21-Jan-22
C19IMPACT_0776	Trinidad and Tobago	11-May-21	CARPHA	N	N	N	N	N	N	23.35		17-May-21	27-May-21	05-Jun-21	05-Jun-21	21-Jan-22
C19IMPACT_0777	Trinidad and Tobago	11-May-21	CARPHA	N	N	N	N	N	N	23.68		17-May-21	27-May-21	05-Jun-21	05-Jun-21	21-Jan-22
C19IMPACT_0778	Trinidad and Tobago	11-May-21	CARPHA	N	N	N	N	Y	N	14.81		17-May-21	27-May-21	05-Jun-21	05-Jun-21	21-Jan-22
C19IMPACT_0779	Trinidad and Tobago	11-May-21	CARPHA	N	N	N	N	Y	N	18.90		17-May-21	27-May-21	05-Jun-21	05-Jun-21	21-Jan-22
C19IMPACT_0780	Trinidad and Tobago	11-May-21	CARPHA	N	N	N	N	Y	N	19.74		17-May-21	27-May-21	05-Jun-21	05-Jun-21	21-Jan-22
C19IMPACT_0781	Trinidad and Tobago	11-May-21	CARPHA	N	N	N	N	Y	N	15.77		17-May-21	27-May-21	05-Jun-21	05-Jun-21	21-Jan-22
C19IMPACT_0782	Trinidad and Tobago	11-May-21	CARPHA	N	N	N	N	Y	N	22.13		17-May-21	27-May-21	05-Jun-21	05-Jun-21	21-Jan-22
C19IMPACT_0783	Trinidad and Tobago	03-May-21	CARPHA	N	N	N	N	N	N	18.57		07-May-21	02-Jun-21	05-Jun-21	05-Jun-21	
C19IMPACT_0784	Trinidad and Tobago	03-May-21	CARPHA	N	N	N	N	Y	N	16.03		10-May-21	02-Jun-21	05-Jun-21	05-Jun-21	24-Jun-21
C19IMPACT_0785	Trinidad and Tobago	03-May-21	CARPHA	N	N	N	N	N	N	17.99		10-May-21	02-Jun-21	05-Jun-21	05-Jun-21	24-Jun-21
C19IMPACT_0786	Trinidad and Tobago	03-May-21	CARPHA	N	N	N	N	Y	N	15.19		10-May-21	02-Jun-21	05-Jun-21	05-Jun-21	24-Jun-21
C19IMPACT_0787	Trinidad and Tobago	03-May-21	CARPHA	N	N	N	N	Y	N	18.36		10-May-21	02-Jun-21	05-Jun-21	05-Jun-21	24-Jun-21
C19IMPACT_0788	Trinidad and Tobago	03-May-21	CARPHA	N	N	N	N	Y	N	20.20		10-May-21	02-Jun-21	05-Jun-21	05-Jun-21	24-Jun-21
C19IMPACT_0789	Trinidad and Tobago	03-May-21	CARPHA	N	N	N	N	Y	N	15.46		10-May-21	02-Jun-21	05-Jun-21	05-Jun-21	24-Jun-21
C19IMPACT_0790	Trinidad and Tobago	03-May-21	CARPHA	N	N	N	N	N	N	21.97		10-May-21	02-Jun-21	05-Jun-21	05-Jun-21	24-Jun-21
C19IMPACT_0791	Trinidad and Tobago	03-May-21	CARPHA	N	N	N	N	Y	N	17.90		10-May-21	02-Jun-21	05-Jun-21	05-Jun-21	24-Jun-21
C19IMPACT_0792	Trinidad and Tobago	03-May-21	CARPHA	N	N	N	N	Y	N	18.41		10-May-21	02-Jun-21	05-Jun-21	05-Jun-21	
C19IMPACT_0793	Trinidad and Tobago	03-May-21	CARPHA	N	N	N	N	Y	N	18.76		07-May-21	02-Jun-21	05-Jun-21	05-Jun-21	24-Jun-21
C19IMPACT_0794	Trinidad and Tobago	03-May-21	CARPHA	N	N	N	N	Y	N	23.37		07-May-21	02-Jun-21	05-Jun-21	05-Jun-21	24-Jun-21
C19IMPACT_0795	Trinidad and Tobago	03-May-21	CARPHA	N	N	N	N	Y	N	15.63		07-May-21	02-Jun-21	05-Jun-21	05-Jun-21	24-Jun-21
C19IMPACT_0796	Trinidad and Tobago	03-May-21	CARPHA	N	N	N	N	Y	N	17.36		07-May-21	02-Jun-21	05-Jun-21	05-Jun-21	
C19IMPACT_0797	Trinidad and Tobago	03-May-21	CARPHA	N	N	N	N	Y	N	22.02		10-May-21	02-Jun-21	13-Oct-21	06-Nov-21	21-Jan-22
C19IMPACT_0798	Trinidad and Tobago	04-May-21	CARPHA	N	N	N	N	N	N	23.23		10-May-21	02-Jun-21	05-Jun-21	05-Jun-21	
C19IMPACT_0799	Trinidad and Tobago	04-May-21	CARPHA	N	N	N	N	Y	N	19.20		07-May-21	02-Jun-21	05-Jun-21	05-Jun-21	24-Jun-21
C19IMPACT_0800	Trinidad and Tobago	05-May-21	CARPHA	N	N	N	N	Y	N	19.16		10-May-21	02-Jun-21	05-Jun-21	05-Jun-21	24-Jun-21
C19IMPACT_0801	Trinidad and Tobago	05-May-21	CARPHA	N	N	N	N	Y	N	20.80		10-May-21	02-Jun-21	05-Jun-21	05-Jun-21	24-Jun-21
C19IMPACT_0802	Trinidad and Tobago	06-May-21	CARPHA	N	N	N	N	N	N	18.57		10-May-21	02-Jun-21	05-Jun-21	06-Nov-21	24-Jun-21
C19IMPACT_0803	Trinidad and Tobago	06-May-21	CARPHA	N	N	N	N	N	N	13.72		10-May-21	02-Jun-21	05-Jun-21	05-Jun-21	24-Jun-21
C19IMPACT_0804	Trinidad and Tobago	06-May-21	CARPHA	N	N	N	N	Y	N	20.27		10-May-21	02-Jun-21	05-Jun-21	05-Jun-21	24-Jun-21
C19IMPACT_0805	Trinidad and Tobago	03-May-21	CARPHA	N	N	N	N	Y	Y	13.92		07-May-21	04-Jun-21	11-Jun-21	09-Jun-21	24-Jun-21
C19IMPACT_0806	Trinidad and Tobago	03-May-21	CARPHA	N	N	N	N	N	Y	20.52		07-May-21	04-Jun-21	11-Jun-21	09-Jun-21	24-Jun-21
C19IMPACT_0807	Trinidad and Tobago	03-May-21	CARPHA	N	N	N	N	Y	Y	23.54		07-May-21	04-Jun-21	11-Jun-21	09-Jun-21	
C19IMPACT_0808	Trinidad and Tobago	03-May-21	CARPHA	N	N	N	N	N	Y	18.62		07-May-21	04-Jun-21	11-Jun-21	09-Jun-21	
C19IMPACT_0809	Trinidad and Tobago	04-May-21	CARPHA	N	N	N	N	N	Y	13.41		07-May-21	04-Jun-21	11-Jun-21	09-Jun-21	24-Jun-21
C19IMPACT_0810	Trinidad and Tobago	04-May-21	CARPHA	N	N	N	N	N	Y	18.74		07-May-21	04-Jun-21	11-Jun-21	09-Jun-21	24-Jun-21
C19IMPACT_0811	Trinidad and Tobago	04-May-21	CARPHA	N	N	N	N	N	Y	15.23		07-May-21	04-Jun-21	11-Jun-21	09-Jun-21	24-Jun-21
C19IMPACT_0812	Trinidad and Tobago	10-May-21	CARPHA	N	N	N	N	N	Y	24.62		17-May-21	04-Jun-21	11-Jun-21	09-Jun-21	24-Jun-21
C19IMPACT_0813	Trinidad and Tobago	10-May-21	CARPHA	N	N	N	N	N	Y	23.84		17-May-21	04-Jun-21	11-Jun-21	09-Jun-21	24-Jun-21
C19IMPACT_0814	Trinidad and Tobago	10-May-21	CARPHA	N	N	N	N	Y	Y	19.34		17-May-21	04-Jun-21	11-Jun-21	09-Jun-21	24-Jun-21
C19IMPACT_0815	Trinidad and Tobago	11-May-21	CARPHA	N	N	N	N	Y	Y	14.62		17-May-21	04-Jun-21	11-Jun-21	09-Jun-21	24-Jun-21
C19IMPACT_0816	Trinidad and Tobago	11-May-21	CARPHA	N	N	N	N	Y	Y	22.42		17-May-21	04-Jun-21	11-Jun-21	09-Jun-21	24-Jun-21
C19IMPACT_0817	Trinidad and Tobago	11-May-21	CARPHA	N	N	N	N	Y	Y	19.64		17-May-21	04-Jun-21	11-Jun-21	09-Jun-21	24-Jun-21
C19IMPACT_0818	Trinidad and Tobago	11-May-21	CARPHA	N	N	N	N	Y	Y	24.09		17-May-21	04-Jun-21	11-Jun-21	09-Jun-21	24-Jun-21
C19IMPACT_0819	Trinidad and Tobago	11-May-21	CARPHA	N	N	N	N	Y	Y	24.39		17-May-21	04-Jun-21	11-Jun-21	09-Jun-21	24-Jun-21
C19IMPACT_0820	Trinidad and Tobago	11-May-21	CARPHA	N	N	N	N	Y	Y	14.99		17-May-21	04-Jun-21	11-Jun-21	09-Jun-21	24-Jun-21
C19IMPACT_0821	Trinidad and Tobago	11-May-21	CARPHA	N	N	N	N	Y	Y	16.35		17-May-21	04-Jun-21	11-Jun-21	09-Jun-21	24-Jun-21
C19IMPACT_0822	Trinidad and Tobago	11-May-21	CARPHA	N	N	N	N	Y	Y	15.81		17-May-21	04-Jun-21	11-Jun-21	09-Jun-21	24-Jun-21
C19IMPACT_0823	Trinidad and Tobago	11-May-21	CARPHA	N	N	N	N	Y	Y	24.02		17-May-21	04-Jun-21	11-Jun-21	09-Jun-21	24-Jun-21
C19IMPACT_0824	Trinidad and Tobago	11-May-21	CARPHA	N	N	N	N	Y	Y	19.32		17-May-21	04-Jun-21	11-Jun-21	09-Jun-21	24-Jun-21
C19IMPACT_0825	Trinidad and Tobago	11-May-21	CARPHA	N	N	N	N	Y	Y	24.32		17-May-21	04-Jun-21	11-Jun-21	09-Jun-21	24-Jun-21
C19IMPACT_0826	Trinidad and Tobago	11-May-21	CARPHA	N	N	N	N	Y	Y	23.41		17-May-21	04-Jun-21	11-Jun-21	09-Jun-21	24-Jun-21
C19IMPACT_0827	Saint Lucia	26-Mar-21	CARPHA	N	N	N	N	Y	N	16.07		28-May-21	07-Jun-21		09-Jun-21	21-Jan-22
C19IMPACT_0828	Saint Lucia	26-Mar-21	CARPHA	N	N	N	N	N	N	15.69		28-May-21	07-Jun-21		09-Jun-21	21-Jan-22

C19IMPACT_0829	Saint Lucia	26-Mar-21	CARPHA	N	N	N	N	N	N	14.31		28-May-21	07-Jun-21		09-Jun-21	21-Jan-22
C19IMPACT_0830	Saint Lucia	26-Mar-21	CARPHA	N	N	N	N	N	N	19.51		28-May-21	07-Jun-21		09-Jun-21	21-Jan-22
C19IMPACT_0831	Saint Lucia	26-Mar-21	CARPHA	N	N	N	N	N	N	17.94		28-May-21	07-Jun-21		09-Jun-21	21-Jan-22
C19IMPACT_0832	Saint Lucia	27-Mar-21	CARPHA	N	N	N	N	N	N	16.81		28-May-21	07-Jun-21		09-Jun-21	21-Jan-22
C19IMPACT_0833	Guyana	29-Mar-21	CARPHA	N	N	N	N	N	N	21.10		28-May-21	07-Jun-21		09-Jun-21	21-Jan-22
C19IMPACT_0834	Guyana	29-Mar-21	CARPHA	N	N	N	N	N	N	23.49		28-May-21	07-Jun-21		09-Jun-21	
C19IMPACT_0835	Guyana	30-Mar-21	CARPHA	N	N	N	N	N	N	16.37		28-May-21	07-Jun-21		09-Jun-21	21-Jan-22
C19IMPACT_0836	Guyana	31-Mar-21	CARPHA	N	N	N	N	N	N	14.37		28-May-21	07-Jun-21		09-Jun-21	21-Jan-22
C19IMPACT_0837	Guyana	01-Apr-21	CARPHA	N	N	N	N	N	N	18.68		28-May-21	07-Jun-21		09-Jun-21	
C19IMPACT_0838	Turks and Caicos Islands	08-Apr-21	CARPHA	N	N	N	N	N	N	18.54		28-May-21	07-Jun-21		09-Jun-21	21-Jan-22
C19IMPACT_0839	Turks and Caicos Islands	08-Apr-21	CARPHA	N	N	N	N	N	N	18.62		28-May-21	07-Jun-21		09-Jun-21	21-Jan-22
C19IMPACT_0840	Turks and Caicos Islands	20-Apr-21	CARPHA	N	N	N	N	N	N	20.82		28-May-21	07-Jun-21		09-Jun-21	21-Jan-22
C19IMPACT_0841	Saint Lucia	23-Apr-21	CARPHA	N	N	N	N	N	N	17.91		28-May-21	07-Jun-21		09-Jun-21	21-Jan-22
C19IMPACT_0842	Saint Lucia	23-Apr-21	CARPHA	N	N	N	N	N	N	15.25		28-May-21	07-Jun-21		09-Jun-21	21-Jan-22
C19IMPACT_0843	Saint Lucia	24-Apr-21	CARPHA	N	N	N	N	N	N	18.72		28-May-21	07-Jun-21		09-Jun-21	21-Jan-22
C19IMPACT_0844	Saint Lucia	24-Apr-21	CARPHA	N	N	N	N	N	N	17.72		28-May-21	07-Jun-21		09-Jun-21	21-Jan-22
C19IMPACT_0845	Saint Lucia	24-Apr-21	CARPHA	N	N	N	N	N	N	21.90		28-May-21	07-Jun-21		09-Jun-21	21-Jan-22
C19IMPACT_0846	Saint Lucia	24-Apr-21	CARPHA	N	N	N	N	N	N	15.20		28-May-21	07-Jun-21		09-Jun-21	21-Jan-22
C19IMPACT_0847	Saint Lucia	24-Apr-21	CARPHA	N	N	N	N	N	N	16.18		28-May-21	07-Jun-21		09-Jun-21	21-Jan-22
C19IMPACT_0848	Saint Lucia	24-Apr-21	CARPHA	N	N	N	N	N	N	17.89		28-May-21	07-Jun-21		09-Jun-21	21-Jan-22
C19IMPACT_0849	Saint Lucia	24-Apr-21	CARPHA	N	N	N	N	N	N	18.68		28-May-21	07-Jun-21		09-Jun-21	21-Jan-22
C19IMPACT_0850	Saint Lucia	24-Apr-21	CARPHA	N	N	N	N	N	N	15.24		28-May-21	07-Jun-21		09-Jun-21	21-Jan-22
C19IMPACT_0851	Saint Lucia	24-Apr-21	CARPHA	N	N	N	N	N	N	15.41		28-May-21	07-Jun-21		09-Jun-21	21-Jan-22
C19IMPACT_0852	Saint Lucia	24-Apr-21	CARPHA	N	N	N	N	N	N	16.73		28-May-21	07-Jun-21		09-Jun-21	21-Jan-22
C19IMPACT_0853	Saint Lucia	24-Apr-21	CARPHA	N	N	N	N	N	N	17.02		28-May-21	07-Jun-21		09-Jun-21	21-Jan-22
C19IMPACT_0854	Barbados	25-Apr-21	CARPHA	N	N	N	N	N	N	17.04		28-May-21	07-Jun-21		09-Jun-21	20-Jun-21
C19IMPACT_0855	Barbados	25-Apr-21	CARPHA	N	N	N	N	N	N	23.48		28-May-21	07-Jun-21		09-Jun-21	20-Jun-21
C19IMPACT_0856	British Virgin Islands	02-May-21	CARPHA	N	N	N	N	N	N	14.61		28-May-21	07-Jun-21		09-Jun-21	21-Jan-22
C19IMPACT_0857	Turks and Caicos Islands	03-May-21	CARPHA	N	N	N	N	N	N	17.48		28-May-21	07-Jun-21		09-Jun-21	21-Jan-22
C19IMPACT_0858	Turks and Caicos Islands	04-May-21	CARPHA	N	N	N	N	N	N	17.06		28-May-21	07-Jun-21		09-Jun-21	21-Jan-22
C19IMPACT_0859	Turks and Caicos Islands	07-May-21	CARPHA	N	N	N	N	N	N	13.77		28-May-21	07-Jun-21		09-Jun-21	21-Jan-22
C19IMPACT_0860	British Virgin Islands	09-May-21	CARPHA	N	N	N	N	N	N	18.84		28-May-21	07-Jun-21		09-Jun-21	21-Jan-22
C19IMPACT_0861	Turks and Caicos Islands	09-May-21	CARPHA	N	N	N	N	N	N	25.04		28-May-21	07-Jun-21		09-Jun-21	21-Jan-22
C19IMPACT_0862	Anguilla	10-May-21	CARPHA	N	N	N	N	N	N	18.55		28-May-21	07-Jun-21		09-Jun-21	21-Jan-22
C19IMPACT_0863	Anguilla	10-May-21	CARPHA	N	N	N	N	N	N	22.13		28-May-21	07-Jun-21		09-Jun-21	21-Jan-22
C19IMPACT_0864	British Virgin Islands	11-May-21	CARPHA	N	N	N	N	N	N	17.72		28-May-21	07-Jun-21		09-Jun-21	21-Jan-22
C19IMPACT_0865	Turks and Caicos Islands	12-May-21	CARPHA	N	N	N	N	N	N	20.82		28-May-21	07-Jun-21		09-Jun-21	21-Jan-22
C19IMPACT_0866	Turks and Caicos Islands	13-May-21	CARPHA	N	N	N	N	N	N	18.67		28-May-21	07-Jun-21		09-Jun-21	21-Jan-22
C19IMPACT_0867	Turks and Caicos Islands	14-May-21	CARPHA	N	N	N	N	N	N	22.01		28-May-21	07-Jun-21		09-Jun-21	21-Jan-22
C19IMPACT_0868	Trinidad and Tobago	25-May-21	CARPHA	N	N	N	N	N	N	19.83		28-May-21	07-Jun-21	11-Jun-21	09-Jun-21	21-Jan-22
C19IMPACT_0869	Trinidad and Tobago	25-May-21	CARPHA	N	N	N	N	Y	N	17.55		28-May-21	07-Jun-21	11-Jun-21	09-Jun-21	21-Jan-22
C19IMPACT_0870	Saint Lucia		CARPHA	N	N	N	N	Y	N	20.02		28-May-21	07-Jun-21		09-Jun-21	
C19IMPACT_0871	Barbados	04-May-21	CARPHA	N	N	N	N	N	N	15.09		02-Jun-21	08-Jun-21		15-Jun-21	24-Jun-21
C19IMPACT_0872	Barbados	04-May-21	CARPHA	N	N	N	N	N	N	21.38		02-Jun-21	08-Jun-21		15-Jun-21	24-Jun-21
C19IMPACT_0873	Barbados	05-May-21	CARPHA	N	N	N	N	N	N	20.07		02-Jun-21	08-Jun-21		15-Jun-21	24-Jun-21
C19IMPACT_0874	Barbados	08-May-21	CARPHA	N	N	N	N	N	N	21.59		02-Jun-21	08-Jun-21		15-Jun-21	24-Jun-21
C19IMPACT_0875	Trinidad and Tobago	20-May-21	MoH	N	N	N	N	N	N	19.16		02-Jun-21	08-Jun-21	18-Jun-21		
C19IMPACT_0876	Barbados	21-May-21	CARPHA	N	N	N	N	N	N	23.48		02-Jun-21	08-Jun-21		15-Jun-21	24-Jun-21
C19IMPACT_0877	Barbados	22-May-21	CARPHA	N	N	N	N	N	N	22.92		02-Jun-21	08-Jun-21		15-Jun-21	20-Jun-21
C19IMPACT_0878	Barbados	24-May-21	CARPHA	N	N	N	N	N	N	20.64		02-Jun-21	08-Jun-21		15-Jun-21	24-Jun-21
C19IMPACT_0879	Saint Vincent and the Grenadines	24-May-21	CARPHA	N	N	N	N	N	N	20.62		02-Jun-21	08-Jun-21		15-Jun-21	
C19IMPACT_0880	Saint Vincent and the Grenadines	25-May-21	CARPHA	N	N	N	N	N	N	14.23		02-Jun-21	08-Jun-21		15-Jun-21	24-Jun-21
C19IMPACT_0881	Saint Vincent and the Grenadines	25-May-21	CARPHA	N	N	N	N	N	N	15.39		02-Jun-21	08-Jun-21		15-Jun-21	24-Jun-21
C19IMPACT_0882	Trinidad and Tobago	25-May-21	MoH	Y	N	Y	N	N	Y	33.20		02-Jun-21	08-Jun-21	18-Jun-21		
C19IMPACT_0883	Saint Vincent and the Grenadines	26-May-21	CARPHA	N	N	N	Y	N	N	16.88		02-Jun-21	08-Jun-21		15-Jun-21	24-Jun-21
C19IMPACT_0884	Saint Vincent and the Grenadines	26-May-21	CARPHA	N	N	N	N	N	N	18.30		02-Jun-21	08-Jun-21		15-Jun-21	24-Jun-21
C19IMPACT_0885	Trinidad and Tobago	26-May-21	MoH	N	N	N	N	N	N	30.60		02-Jun-21	08-Jun-21	18-Jun-21		
C19IMPACT_0886	Saint Vincent and the Grenadines	27-May-21	CARPHA	N	N	N	N	N	N	15.18		02-Jun-21	08-Jun-21		15-Jun-21	24-Jun-21
C19IMPACT_0887	Trinidad and Tobago	04-May-21	CARPHA	N	N	N	N	N	Y	18.29		07-May-21	09-Jun-21	18-Jun-21	15-Jun-21	24-Jun-21
C19IMPACT_0888	Trinidad and Tobago	04-May-21	CARPHA	N	N	N	N	N	N	23.37		07-May-21	09-Jun-21	18-Jun-21	15-Jun-21	24-Jun-21
C19IMPACT_0889	Trinidad and Tobago	04-May-21	CARPHA	N	N	N	N	N	N	14.84		07-May-21	09-Jun-21	18-Jun-21	15-Jun-21	24-Jun-21
C19IMPACT_0890	Trinidad and Tobago	04-May-21	CARPHA	N	N	N	N	Y	N	18.25		07-May-21	09-Jun-21	18-Jun-21	15-Jun-21	24-Jun-21
C19IMPACT_0891	Trinidad and Tobago	04-May-21	CARPHA	N	N	N	N	Y	N	12.20		07-May-21	09-Jun-21	18-Jun-21	15-Jun-21	24-Jun-21
C19IMPACT_0892	Trinidad and Tobago	05-May-21	CARPHA	N	N	N	N	Y	N	21.19		07-May-21	09-Jun-21	18-Jun-21	15-Jun-21	

C19IMPACT_0893	Trinidad and Tobago	05-May-21	CARPHA	N	N	N	N	N	N	22.00		07-May-21	09-Jun-21	18-Jun-21	15-Jun-21	
C19IMPACT_0894	Trinidad and Tobago	05-May-21	CARPHA	N	N	N	N	Y	N	20.07		07-May-21	09-Jun-21	18-Jun-21	15-Jun-21	
C19IMPACT_0895	Trinidad and Tobago	05-May-21	CARPHA	N	N	N	N	N	N	17.11		07-May-21	09-Jun-21	18-Jun-21	15-Jun-21	24-Jun-21
C19IMPACT_0896	Trinidad and Tobago	05-May-21	CARPHA	N	N	N	N	Y	N	23.13		07-May-21	09-Jun-21	18-Jun-21	15-Jun-21	24-Jun-21
C19IMPACT_0897	Trinidad and Tobago	05-May-21	CARPHA	N	N	N	N	Y	N	14.49		07-May-21	09-Jun-21	18-Jun-21	15-Jun-21	24-Jun-21
C19IMPACT_0898	Trinidad and Tobago	05-May-21	CARPHA	N	N	N	N	N	N	15.91		07-May-21	09-Jun-21	18-Jun-21	15-Jun-21	24-Jun-21
C19IMPACT_0899	Trinidad and Tobago	05-May-21	CARPHA	N	N	N	N	Y	N	11.09		07-May-21	09-Jun-21	18-Jun-21	15-Jun-21	24-Jun-21
C19IMPACT_0900	Trinidad and Tobago	05-May-21	CARPHA	N	N	N	N	N	N	13.35		07-May-21	09-Jun-21	18-Jun-21	15-Jun-21	24-Jun-21
C19IMPACT_0901	Trinidad and Tobago	05-May-21	CARPHA	N	N	N	N	N	N	13.98		07-May-21	09-Jun-21	18-Jun-21	15-Jun-21	24-Jun-21
C19IMPACT_0902	Trinidad and Tobago	05-May-21	CARPHA	N	N	N	N	N	N	14.81		07-May-21	09-Jun-21	18-Jun-21	15-Jun-21	24-Jun-21
C19IMPACT_0903	Trinidad and Tobago	05-May-21	CARPHA	N	N	N	N	N	N	27.89		07-May-21	09-Jun-21	18-Jun-21	15-Jun-21	24-Jun-21
C19IMPACT_0904	Trinidad and Tobago	05-May-21	CARPHA	N	N	N	N	N	N	22.79		07-May-21	09-Jun-21	18-Jun-21	15-Jun-21	24-Jun-21
C19IMPACT_0905	Trinidad and Tobago	05-May-21	CARPHA	N	N	N	N	N	N	24.56		07-May-21	09-Jun-21	18-Jun-21	15-Jun-21	24-Jun-21
C19IMPACT_0906	Trinidad and Tobago	05-May-21	CARPHA	N	N	N	N	N	N	22.47		07-May-21	09-Jun-21	18-Jun-21	15-Jun-21	24-Jun-21
C19IMPACT_0907	Trinidad and Tobago	05-May-21	CARPHA	N	N	N	N	N	N	14.50		07-May-21	09-Jun-21	18-Jun-21	15-Jun-21	24-Jun-21
C19IMPACT_0908	Trinidad and Tobago	05-May-21	CARPHA	N	N	N	N	N	N	20.95		07-May-21	09-Jun-21	18-Jun-21	15-Jun-21	24-Jun-21
C19IMPACT_0909	Montserrat	05-Feb-21	CARPHA	N	N	N	N	Y	N	16.97		07-Jun-21	10-Jun-21		15-Jun-21	19-Jun-21
C19IMPACT_0910	Montserrat	05-Feb-21	CARPHA	N	N	N	N	N	N	16.44		07-Jun-21	10-Jun-21		15-Jun-21	19-Jun-21
C19IMPACT_0911	Trinidad and Tobago	07-May-21	CARPHA	N	N	N	N	N	N	13.63		11-May-21	10-Jun-21	18-Jun-21	15-Jun-21	30-Jun-21
C19IMPACT_0912	Trinidad and Tobago	07-May-21	CARPHA	N	N	N	N	Y	N	15.09		11-May-21	10-Jun-21	18-Jun-21	15-Jun-21	30-Jun-21
C19IMPACT_0913	Trinidad and Tobago	07-May-21	CARPHA	N	N	N	N	N	N	21.70		11-May-21	10-Jun-21	18-Jun-21	15-Jun-21	
C19IMPACT_0914	Trinidad and Tobago	07-May-21	CARPHA	N	N	N	N	N	N	16.51		11-May-21	10-Jun-21	18-Jun-21	15-Jun-21	30-Jun-21
C19IMPACT_0915	Trinidad and Tobago	07-May-21	CARPHA	N	N	N	N	Y	N	15.32		11-May-21	10-Jun-21	18-Jun-21	15-Jun-21	30-Jun-21
C19IMPACT_0916	Trinidad and Tobago	07-May-21	CARPHA	N	N	N	N	Y	N	21.67		11-May-21	10-Jun-21	18-Jun-21	15-Jun-21	30-Jun-21
C19IMPACT_0917	Trinidad and Tobago	07-May-21	CARPHA	N	N	N	N	Y	N	14.27		11-May-21	10-Jun-21	18-Jun-21	15-Jun-21	30-Jun-21
C19IMPACT_0918	Trinidad and Tobago	07-May-21	CARPHA	N	N	N	N	Y	N	15.65		11-May-21	10-Jun-21	18-Jun-21	15-Jun-21	30-Jun-21
C19IMPACT_0919	Trinidad and Tobago	07-May-21	CARPHA	N	N	N	N	Y	N	19.76		11-May-21	10-Jun-21	18-Jun-21	15-Jun-21	30-Jun-21
C19IMPACT_0920	Trinidad and Tobago	08-May-21	CARPHA	N	N	N	N	Y	N	24.30		11-May-21	10-Jun-21	18-Jun-21	15-Jun-21	
C19IMPACT_0921	Trinidad and Tobago	08-May-21	CARPHA	N	N	N	N	N	N	22.15		11-May-21	10-Jun-21	18-Jun-21	15-Jun-21	30-Jun-21
C19IMPACT_0922	Trinidad and Tobago	08-May-21	CARPHA	N	N	N	N	N	N	22.49		11-May-21	10-Jun-21	18-Jun-21	15-Jun-21	30-Jun-21
C19IMPACT_0923	Trinidad and Tobago	08-May-21	CARPHA	N	N	N	N	N	N	21.94		11-May-21	10-Jun-21	18-Jun-21	15-Jun-21	30-Jun-21
C19IMPACT_0924	Trinidad and Tobago	08-May-21	CARPHA	N	N	N	N	N	N	16.72		11-May-21	10-Jun-21	18-Jun-21	15-Jun-21	30-Jun-21
C19IMPACT_0925	Trinidad and Tobago	08-May-21	CARPHA	N	N	N	N	N	N	21.74		11-May-21	10-Jun-21	18-Jun-21	15-Jun-21	30-Jun-21
C19IMPACT_0926	Trinidad and Tobago	08-May-21	CARPHA	N	N	N	N	N	N	18.99		11-May-21	10-Jun-21	18-Jun-21	15-Jun-21	30-Jun-21
C19IMPACT_0927	Trinidad and Tobago	08-May-21	CARPHA	N	N	N	N	N	N	29.00		11-May-21	10-Jun-21	18-Jun-21	15-Jun-21	30-Jun-21
C19IMPACT_0928	Trinidad and Tobago	09-May-21	CARPHA	N	N	N	N	N	N	17.09		11-May-21	10-Jun-21	18-Jun-21	15-Jun-21	30-Jun-21
C19IMPACT_0929	Trinidad and Tobago	10-May-21	CARPHA	N	N	N	N	N	N	15.56		11-May-21	10-Jun-21	18-Jun-21	15-Jun-21	30-Jun-21
C19IMPACT_0930	Trinidad and Tobago	10-May-21	CARPHA	N	N	N	N	N	N	18.33		11-May-21	10-Jun-21	18-Jun-21	15-Jun-21	30-Jun-21
C19IMPACT_0931	Trinidad and Tobago	26-May-21	CARPHA	N	N	N	N	N	N	19.79		02-Jun-21	10-Jun-21	18-Jun-21	15-Jun-21	30-Jun-21
C19IMPACT_0932	Trinidad and Tobago	28-May-21	CARPHA	N	N	N	N	N	N	20.66		02-Jun-21	10-Jun-21	18-Jun-21	15-Jun-21	30-Jun-21
C19IMPACT_0933	Trinidad and Tobago	28-May-21	CARPHA	N	N	N	N	Y	N	14.07		02-Jun-21	10-Jun-21	18-Jun-21	15-Jun-21	30-Jun-21
C19IMPACT_0934	Trinidad and Tobago	28-May-21	CARPHA	N	N	N	N	N	N	14.49		02-Jun-21	10-Jun-21	18-Jun-21	15-Jun-21	30-Jun-21
C19IMPACT_0935	Trinidad and Tobago	28-May-21	CARPHA	N	N	N	N	N	N	16.64		02-Jun-21	10-Jun-21	18-Jun-21	15-Jun-21	30-Jun-21
C19IMPACT_0936	Trinidad and Tobago	28-May-21	CARPHA	N	N	N	N	Y	N	17.57		02-Jun-21	10-Jun-21	18-Jun-21	15-Jun-21	30-Jun-21
C19IMPACT_0937	Trinidad and Tobago	28-May-21	CARPHA	N	N	N	N	Y	N	19.96		02-Jun-21	10-Jun-21	18-Jun-21	15-Jun-21	30-Jun-21
C19IMPACT_0938	Trinidad and Tobago	29-May-21	CARPHA	N	N	N	N	N	N	22.33		04-Jun-21	10-Jun-21	18-Jun-21	15-Jun-21	30-Jun-21
C19IMPACT_0939	Saint Kitts and Nevis	31-May-21	CARPHA	N	N	N	N	N	N	15.39		04-Jun-21	10-Jun-21		15-Jun-21	30-Jun-21
C19IMPACT_0940	Trinidad and Tobago	31-May-21	CARPHA	N	N	N	N	N	N	17.10		04-Jun-21	10-Jun-21	18-Jun-21	15-Jun-21	
C19IMPACT_0941	Trinidad and Tobago	31-May-21	CARPHA	N	N	N	N	N	N	17.72		07-Jun-21	10-Jun-21	18-Jun-21	15-Jun-21	30-Jun-21
C19IMPACT_0942	Trinidad and Tobago	31-May-21	CARPHA	N	N	N	N	Y	N	11.56		02-Jun-21	10-Jun-21	18-Jun-21	15-Jun-21	21-Jan-22
C19IMPACT_0943	Trinidad and Tobago	01-Jun-21	CARPHA	N	N	N	N	Y	N	21.75		07-Jun-21	10-Jun-21	18-Jun-21	15-Jun-21	30-Jun-21
C19IMPACT_0944	Trinidad and Tobago	01-Jun-21	CARPHA	N	N	N	N	Y	N	19.33		07-Jun-21	10-Jun-21	18-Jun-21	15-Jun-21	30-Jun-21
C19IMPACT_0945	Trinidad and Tobago	01-Jun-21	CARPHA	N	N	N	N	Y	N	18.40		04-Jun-21	10-Jun-21	18-Jun-21	15-Jun-21	30-Jun-21
C19IMPACT_0946	Trinidad and Tobago	02-Jun-21	CARPHA	N	N	N	N	N	N	19.98		07-Jun-21	10-Jun-21	18-Jun-21	15-Jun-21	30-Jun-21
C19IMPACT_0947	Trinidad and Tobago	02-Jun-21	CARPHA	N	N	N	N	Y	N	19.88		07-Jun-21	10-Jun-21	18-Jun-21	15-Jun-21	
C19IMPACT_0948	Trinidad and Tobago	03-Jun-21	CARPHA	N	N	N	N	N	N	14.46		07-Jun-21	10-Jun-21	18-Jun-21	15-Jun-21	30-Jun-21
C19IMPACT_0949	Trinidad and Tobago	03-Jun-21	CARPHA	N	N	N	N	Y	N	16.66		07-Jun-21	10-Jun-21	18-Jun-21	15-Jun-21	30-Jun-21
C19IMPACT_0950	Trinidad and Tobago	04-Jun-21	CARPHA	N	N	N	N	Y	N	18.05		07-Jun-21	10-Jun-21	18-Jun-21	15-Jun-21	30-Jun-21
C19IMPACT_0951	Trinidad and Tobago		CARPHA	N	N	N	N	Y	N				10-Jun-21	18-Jun-21	15-Jun-21	
C19IMPACT_0952	Trinidad and Tobago	21-May-21	CARPHA	N	N	N	N	N	N	19.32		28-May-21	13-Jun-21	18-Jun-21	15-Jun-21	24-Jun-21
C19IMPACT_0953	Trinidad and Tobago	22-May-21	CARPHA	N	N	N	N	N	N	16.08		28-May-21	13-Jun-21	18-Jun-21	15-Jun-21	
C19IMPACT_0954	Trinidad and Tobago	22-May-21	CARPHA	N	N	N	N	N	N	13.30		28-May-21	13-Jun-21	18-Jun-21	15-Jun-21	
C19IMPACT_0955	Trinidad and Tobago	22-May-21	CARPHA	N	N	N	N	Y	N	23.09		28-May-21	13-Jun-21	18-Jun-21	15-Jun-21	
C19IMPACT_0956	Trinidad and Tobago	23-May-21	CARPHA	N	N	N	N	N	N	20.29		28-May-21	13-Jun-21	18-Jun-21	15-Jun-21	24-Jun-21

C19IMPACT_0957	Trinidad and Tobago	24-May-21	CARPHA	N	N	N	N	N	N	20.75	28-May-21	13-Jun-21	18-Jun-21	15-Jun-21	24-Jun-21
C19IMPACT_0958	Trinidad and Tobago	24-May-21	CARPHA	N	N	N	N	Y	N	22.35	28-May-21	13-Jun-21	18-Jun-21	15-Jun-21	24-Jun-21
C19IMPACT_0959	Trinidad and Tobago	25-May-21	CARPHA	N	N	N	N	Y	N	22.97	28-May-21	13-Jun-21	18-Jun-21	15-Jun-21	24-Jun-21
C19IMPACT_0960	Trinidad and Tobago	25-May-21	CARPHA	N	N	N	N	Y	N	19.99	28-May-21	13-Jun-21	18-Jun-21	15-Jun-21	24-Jun-21
C19IMPACT_0961	Trinidad and Tobago	25-May-21	CARPHA	N	N	N	N	Y	N	23.00	28-May-21	13-Jun-21	18-Jun-21	15-Jun-21	
C19IMPACT_0962	Trinidad and Tobago	25-May-21	CARPHA	N	N	N	N	Y	N	21.70	28-May-21	13-Jun-21	18-Jun-21	15-Jun-21	
C19IMPACT_0963	Trinidad and Tobago	25-May-21	CARPHA	N	N	N	N	Y	N	22.94	28-May-21	13-Jun-21	18-Jun-21	15-Jun-21	
C19IMPACT_0964	Trinidad and Tobago	25-May-21	CARPHA	N	N	N	N	Y	N	14.01	28-May-21	13-Jun-21	18-Jun-21	15-Jun-21	24-Jun-21
C19IMPACT_0965	Trinidad and Tobago	25-May-21	CARPHA	N	N	N	N	Y	N	19.99	28-May-21	13-Jun-21	18-Jun-21	15-Jun-21	24-Jun-21
C19IMPACT_0966	Trinidad and Tobago	25-May-21	CARPHA	N	N	N	N	Y	N	22.46	28-May-21	13-Jun-21	18-Jun-21	15-Jun-21	24-Jun-21
C19IMPACT_0967	Trinidad and Tobago	25-May-21	CARPHA	N	N	N	N	Y	N	23.67	28-May-21	13-Jun-21	18-Jun-21	15-Jun-21	24-Jun-21
C19IMPACT_0968	Trinidad and Tobago	25-May-21	CARPHA	N	N	N	N	Y	N	14.74	28-May-21	13-Jun-21	18-Jun-21	15-Jun-21	24-Jun-21
C19IMPACT_0969	Trinidad and Tobago	25-May-21	CARPHA	N	N	N	N	Y	N	12.22	28-May-21	13-Jun-21	18-Jun-21	15-Jun-21	24-Jun-21
C19IMPACT_0970	Trinidad and Tobago	25-May-21	CARPHA	N	N	N	N	Y	N	21.97	28-May-21	13-Jun-21	18-Jun-21	15-Jun-21	24-Jun-21
C19IMPACT_0971	Trinidad and Tobago	25-May-21	CARPHA	N	N	N	N	Y	N	15.03	28-May-21	13-Jun-21	18-Jun-21	15-Jun-21	24-Jun-21
C19IMPACT_0972	Trinidad and Tobago	25-May-21	CARPHA	N	N	N	N	Y	N	19.00	28-May-21	13-Jun-21	18-Jun-21	15-Jun-21	24-Jun-21
C19IMPACT_0973	Trinidad and Tobago	25-May-21	CARPHA	N	N	N	N	Y	N	16.55	28-May-21	13-Jun-21	18-Jun-21	15-Jun-21	24-Jun-21
C19IMPACT_0974	Antigua and Barbuda	14-Feb-21	CARPHA	N	N	N	N	Y	N	22.59	14-Jun-21	16-Jun-21		21-Jun-21	14-Jul-21
C19IMPACT_0975	Antigua and Barbuda	17-Feb-21	CARPHA	N	N	N	N	N	N	14.62	14-Jun-21	16-Jun-21		21-Jun-21	14-Jul-21
C19IMPACT_0976	Antigua and Barbuda	17-Feb-21	CARPHA	N	N	N	N	N	N	14.21	14-Jun-21	16-Jun-21		21-Jun-21	14-Jul-21
C19IMPACT_0977	Antigua and Barbuda	02-Mar-21	CARPHA	N	N	N	N	N	N	22.52	14-Jun-21	16-Jun-21		21-Jun-21	14-Jul-21
C19IMPACT_0978	Antigua and Barbuda	02-Mar-21	CARPHA	N	N	N	N	N	N	16.59	14-Jun-21	16-Jun-21		21-Jun-21	14-Jul-21
C19IMPACT_0979	Antigua and Barbuda	04-Mar-21	CARPHA	N	N	N	N	N	N	22.00	14-Jun-21	16-Jun-21		21-Jun-21	21-Jan-22
C19IMPACT_0980	Antigua and Barbuda	08-Mar-21	CARPHA	N	N	N	N	N	N	23.14	14-Jun-21	16-Jun-21		21-Jun-21	14-Jul-21
C19IMPACT_0981	Trinidad and Tobago	10-Mar-21	CARPHA	Y	N	Y	N	N	N	22.21	14-Jun-21	16-Jun-21	26-Jun-21	21-Jun-21	
C19IMPACT_0982	Antigua and Barbuda	17-Mar-21	CARPHA	N	N	N	N	N	N	13.95	14-Jun-21	16-Jun-21		21-Jun-21	14-Jul-21
C19IMPACT_0983	Trinidad and Tobago	10-May-21	CARPHA	Y	N	Y	N	N	N	18.08	12-May-21	16-Jun-21	26-Jun-21	21-Jun-21	30-Jun-21
C19IMPACT_0984	Trinidad and Tobago	10-May-21	CARPHA	Y	N	Y	N	N	N	21.79	12-May-21	16-Jun-21	26-Jun-21	21-Jun-21	
C19IMPACT_0985	Trinidad and Tobago	10-May-21	CARPHA	Y	N	Y	N	N	N	22.65	12-May-21	16-Jun-21	26-Jun-21	21-Jun-21	
C19IMPACT_0986	Trinidad and Tobago	10-May-21	CARPHA	Y	N	Y	N	N	N	23.75	12-May-21	16-Jun-21	26-Jun-21	21-Jun-21	
C19IMPACT_0987	Trinidad and Tobago	10-May-21	CARPHA	Y	N	Y	N	N	N	19.59	12-May-21	16-Jun-21	26-Jun-21	21-Jun-21	30-Jun-21
C19IMPACT_0988	Trinidad and Tobago	10-May-21	CARPHA	Y	N	Y	N	N	N	10.32	12-May-21	16-Jun-21	26-Jun-21	21-Jun-21	30-Jun-21
C19IMPACT_0989	Trinidad and Tobago	10-May-21	CARPHA	Y	N	Y	N	N	N	18.20	12-May-21	16-Jun-21	26-Jun-21	21-Jun-21	30-Jun-21
C19IMPACT_0990	Trinidad and Tobago	10-May-21	CARPHA	Y	N	Y	N	N	N	23.65	12-May-21	16-Jun-21	26-Jun-21	21-Jun-21	30-Jun-21
C19IMPACT_0991	Trinidad and Tobago	10-May-21	CARPHA	Y	N	Y	N	N	N	16.70	12-May-21	16-Jun-21	26-Jun-21	21-Jun-21	30-Jun-21
C19IMPACT_0992	Trinidad and Tobago	10-May-21	CARPHA	Y	N	Y	N	N	N	20.23	12-May-21	16-Jun-21	26-Jun-21	21-Jun-21	30-Jun-21
C19IMPACT_0993	Trinidad and Tobago	10-May-21	CARPHA	Y	N	Y	N	N	N	24.03	12-May-21	16-Jun-21	26-Jun-21	21-Jun-21	
C19IMPACT_0994	Trinidad and Tobago	11-May-21	CARPHA	Y	N	Y	N	N	N	18.75	12-May-21	16-Jun-21	26-Jun-21	21-Jun-21	30-Jun-21
C19IMPACT_0995	Trinidad and Tobago	11-May-21	CARPHA	Y	N	Y	N	N	N	15.98	12-May-21	16-Jun-21	26-Jun-21	21-Jun-21	30-Jun-21
C19IMPACT_0996	Trinidad and Tobago	11-May-21	CARPHA	Y	N	Y	N	N	N	18.98	12-May-21	16-Jun-21	26-Jun-21	21-Jun-21	28-Jun-21
C19IMPACT_0997	Trinidad and Tobago	11-May-21	CARPHA	N	N	N	N	N	N	21.79	12-May-21	16-Jun-21	26-Jun-21	21-Jun-21	28-Jun-21
C19IMPACT_0998	Trinidad and Tobago	11-May-21	CARPHA	N	N	N	N	N	N	24.65	12-May-21	16-Jun-21	26-Jun-21	21-Jun-21	28-Jun-21
C19IMPACT_0999	Trinidad and Tobago	11-May-21	CARPHA	N	N	N	N	N	N	13.69	12-May-21	16-Jun-21	26-Jun-21	21-Jun-21	28-Jun-21
C19IMPACT_1000	Trinidad and Tobago	11-May-21	CARPHA	N	N	N	N	N	N	21.85	12-May-21	16-Jun-21	26-Jun-21	21-Jun-21	28-Jun-21
C19IMPACT_1001	Trinidad and Tobago	23-May-21	CARPHA	Y	N	Y	N	N	N	18.07	28-May-21	16-Jun-21	26-Jun-21	21-Jun-21	30-Jun-21
C19IMPACT_1002	Trinidad and Tobago	02-Jun-21	CARPHA	Y	N	Y	N	N	N	21.23	10-Jun-21	16-Jun-21	26-Jun-21	21-Jun-21	28-Jun-21
C19IMPACT_1003	Trinidad and Tobago	04-Jun-21	CARPHA	Y	N	Y	N	N	N	14.30	10-Jun-21	16-Jun-21	26-Jun-21	21-Jun-21	28-Jun-21
C19IMPACT_1004	Saint Kitts and Nevis	05-Jun-21	CARPHA	N	N	N	N	N	N	16.63	10-Jun-21	16-Jun-21		21-Jun-21	28-Jun-21
C19IMPACT_1005	Saint Kitts and Nevis	05-Jun-21	CARPHA	N	N	N	N	N	N	18.34	10-Jun-21	16-Jun-21		21-Jun-21	28-Jun-21
C19IMPACT_1006	Saint Kitts and Nevis	05-Jun-21	CARPHA	N	N	N	N	N	N	18.14	10-Jun-21	16-Jun-21		21-Jun-21	28-Jun-21
C19IMPACT_1007	Saint Kitts and Nevis	05-Jun-21	CARPHA	N	N	N	N	N	N	18.97	10-Jun-21	16-Jun-21		21-Jun-21	28-Jun-21
C19IMPACT_1008	Saint Kitts and Nevis	05-Jun-21	CARPHA	N	N	N	N	N	N	17.73	10-Jun-21	16-Jun-21		21-Jun-21	28-Jun-21
C19IMPACT_1009	Saint Kitts and Nevis	05-Jun-21	CARPHA	N	N	N	N	N	N	20.70	10-Jun-21	16-Jun-21		21-Jun-21	28-Jun-21
C19IMPACT_1010	Saint Kitts and Nevis	06-Jun-21	CARPHA	N	N	N	N	N	N	16.48	10-Jun-21	16-Jun-21		21-Jun-21	28-Jun-21
C19IMPACT_1011	Saint Kitts and Nevis	06-Jun-21	CARPHA	N	N	N	N	N	N	17.99	10-Jun-21	16-Jun-21		21-Jun-21	28-Jun-21
C19IMPACT_1012	Saint Kitts and Nevis	06-Jun-21	CARPHA	N	N	N	N	N	N	17.55	10-Jun-21	16-Jun-21		21-Jun-21	28-Jun-21
C19IMPACT_1013	Trinidad and Tobago	06-Jun-21	CARPHA	N	N	N	N	N	N	19.85	11-Jun-21	16-Jun-21	26-Jun-21	21-Jun-21	28-Jun-21
C19IMPACT_1014	Trinidad and Tobago	07-Jun-21	CARPHA	Y	N	Y	N	N	N	19.03	10-Jun-21	16-Jun-21	26-Jun-21	21-Jun-21	28-Jun-21
C19IMPACT_1015	Trinidad and Tobago	07-Jun-21	CARPHA	N	N	N	N	N	N	16.78	10-Jun-21	16-Jun-21	26-Jun-21	21-Jun-21	28-Jun-21
C19IMPACT_1016	Trinidad and Tobago	07-Jun-21	CARPHA	N	N	N	N	N	N	22.15	11-Jun-21	16-Jun-21	26-Jun-21	21-Jun-21	28-Jun-21
C19IMPACT_1017	Trinidad and Tobago	09-Jun-21	CARPHA	Y	N	Y	N	N	N	24.46	11-Jun-21	16-Jun-21	26-Jun-21	21-Jun-21	
C19IMPACT_1018	Trinidad and Tobago	05-May-21	CARPHA	N	N	N	N	N	N	15.41	28-May-21	22-Jun-21	26-Jun-21	26-Jun-21	28-Jun-21
C19IMPACT_1019	Trinidad and Tobago	06-May-21	CARPHA	N	N	N	N	N	N	13.45	28-May-21	22-Jun-21	26-Jun-21	26-Jun-21	28-Jun-21
C19IMPACT_1020	Trinidad and Tobago	06-May-21	CARPHA	N	N	N	N	N	N	17.10	28-May-21	22-Jun-21	26-Jun-21	26-Jun-21	28-Jun-21

C19IMPACT_1021	Trinidad and Tobago	06-May-21	CARPHA	N	N	N	N	N	N	17.69		28-May-21	22-Jun-21	26-Jun-21	26-Jun-21	28-Jun-21
C19IMPACT_1022	Trinidad and Tobago	06-May-21	CARPHA	N	N	N	N	N	N	17.83		28-May-21	22-Jun-21	26-Jun-21	26-Jun-21	28-Jun-21
C19IMPACT_1023	Trinidad and Tobago	11-May-21	CARPHA	N	N	N	N	N	N	16.40		28-May-21	22-Jun-21	26-Jun-21	26-Jun-21	
C19IMPACT_1024	Trinidad and Tobago	12-May-21	CARPHA	N	N	N	N	N	N	20.49		28-May-21	22-Jun-21	26-Jun-21	26-Jun-21	28-Jun-21
C19IMPACT_1025	Trinidad and Tobago	14-May-21	CARPHA	N	N	N	N	N	N	16.40		28-May-21	22-Jun-21	26-Jun-21	26-Jun-21	
C19IMPACT_1026	Trinidad and Tobago	14-May-21	CARPHA	N	N	N	N	N	N	21.21		28-May-21	22-Jun-21	26-Jun-21	26-Jun-21	
C19IMPACT_1027	Trinidad and Tobago	14-May-21	CARPHA	N	N	N	N	N	N	21.50		28-May-21	22-Jun-21	26-Jun-21	26-Jun-21	
C19IMPACT_1028	Trinidad and Tobago	14-May-21	CARPHA	N	N	N	N	N	N	14.51		28-May-21	22-Jun-21	26-Jun-21	26-Jun-21	28-Jun-21
C19IMPACT_1029	Trinidad and Tobago	14-May-21	CARPHA	N	N	N	N	N	N	16.13		28-May-21	22-Jun-21	26-Jun-21	26-Jun-21	28-Jun-21
C19IMPACT_1030	Trinidad and Tobago	14-May-21	CARPHA	N	N	N	N	N	N	17.52		28-May-21	22-Jun-21	26-Jun-21	26-Jun-21	28-Jun-21
C19IMPACT_1031	Trinidad and Tobago	14-May-21	CARPHA	N	N	N	N	N	N	14.97		28-May-21	22-Jun-21	26-Jun-21	26-Jun-21	28-Jun-21
C19IMPACT_1032	Trinidad and Tobago	15-May-21	CARPHA	N	N	N	N	N	N	16.01		28-May-21	22-Jun-21	26-Jun-21	26-Jun-21	28-Jun-21
C19IMPACT_1033	Trinidad and Tobago	15-May-21	CARPHA	N	N	N	N	N	N	15.99		28-May-21	22-Jun-21	26-Jun-21	26-Jun-21	28-Jun-21
C19IMPACT_1034	Trinidad and Tobago	17-May-21	CARPHA	N	N	N	N	N	N	24.80		28-May-21	22-Jun-21	26-Jun-21	26-Jun-21	28-Jun-21
C19IMPACT_1035	Trinidad and Tobago	17-May-21	CARPHA	N	N	N	N	N	N	15.98		28-May-21	22-Jun-21	26-Jun-21	26-Jun-21	28-Jun-21
C19IMPACT_1036	Trinidad and Tobago	22-May-21	CARPHA	N	N	N	N	N	N	22.46		28-May-21	22-Jun-21	26-Jun-21	26-Jun-21	
C19IMPACT_1037	Trinidad and Tobago	23-May-21	CARPHA	N	N	N	N	N	N	18.57		28-May-21	22-Jun-21	26-Jun-21	26-Jun-21	28-Jun-21
C19IMPACT_1038	Trinidad and Tobago	25-May-21	CARPHA	N	N	N	N	N	N	14.82		28-May-21	22-Jun-21	26-Jun-21	26-Jun-21	28-Jun-21
C19IMPACT_1039	Trinidad and Tobago	25-May-21	CARPHA	N	N	N	N	N	N	21.54		28-May-21	22-Jun-21	26-Jun-21	26-Jun-21	28-Jun-21
C19IMPACT_1040	Antigua and Barbuda	23-Mar-21	CARPHA	N	N	N	N	N	N	15.10		21-Jun-21	24-Jun-21		26-Jun-21	28-Jun-21
C19IMPACT_1041	Antigua and Barbuda	07-Apr-21	CARPHA	N	N	N	N	N	N	15.70		21-Jun-21	24-Jun-21		26-Jun-21	28-Jun-21
C19IMPACT_1042	Antigua and Barbuda	14-Apr-21	CARPHA	N	N	N	N	N	N	19.07		21-Jun-21	24-Jun-21		26-Jun-21	28-Jun-21
C19IMPACT_1043	Antigua and Barbuda	16-Apr-21	CARPHA	N	N	N	N	N	N	20.99		21-Jun-21	24-Jun-21		26-Jun-21	28-Jun-21
C19IMPACT_1044	Antigua and Barbuda	20-Apr-21	CARPHA	N	N	N	N	N	N	14.15		21-Jun-21	24-Jun-21		26-Jun-21	21-Jan-22
C19IMPACT_1045	Antigua and Barbuda	24-Apr-21	CARPHA	N	N	N	N	N	N	22.40		21-Jun-21	24-Jun-21		26-Jun-21	28-Jun-21
C19IMPACT_1046	Antigua and Barbuda	24-Apr-21	CARPHA	N	N	N	N	N	N	20.94		21-Jun-21	24-Jun-21		26-Jun-21	21-Jan-22
C19IMPACT_1047	Antigua and Barbuda	03-May-21	CARPHA	N	N	N	N	N	N	16.88		21-Jun-21	24-Jun-21		26-Jun-21	28-Jun-21
C19IMPACT_1048	Antigua and Barbuda	06-May-21	CARPHA	N	N	N	N	N	N	18.74		21-Jun-21	24-Jun-21		26-Jun-21	28-Jun-21
C19IMPACT_1049	Antigua and Barbuda	06-May-21	CARPHA	N	N	N	N	N	N	16.78		21-Jun-21	24-Jun-21		26-Jun-21	28-Jun-21
C19IMPACT_1050	Trinidad and Tobago	10-Jun-21	CARPHA	N	N	N	N	N	N	14.72		21-Jun-21	24-Jun-21	26-Jun-21	26-Jun-21	28-Jun-21
C19IMPACT_1051	Trinidad and Tobago	11-Jun-21	CARPHA	N	N	N	N	N	N	18.11		21-Jun-21	24-Jun-21	26-Jun-21	26-Jun-21	28-Jun-21
C19IMPACT_1052	Trinidad and Tobago	12-Jun-21	CARPHA	N	N	N	N	N	N	22.19		21-Jun-21	24-Jun-21	26-Jun-21	26-Jun-21	28-Jun-21
C19IMPACT_1053	Trinidad and Tobago	12-Jun-21	CARPHA	N	N	N	N	N	N	18.09		21-Jun-21	24-Jun-21	26-Jun-21	26-Jun-21	28-Jun-21
C19IMPACT_1054	Trinidad and Tobago	12-Jun-21	CARPHA	N	N	N	N	N	N	20.11		21-Jun-21	24-Jun-21	26-Jun-21	26-Jun-21	28-Jun-21
C19IMPACT_1055	Trinidad and Tobago	13-Jun-21	CARPHA	N	N	N	N	N	N	20.70		21-Jun-21	24-Jun-21	26-Jun-21	26-Jun-21	28-Jun-21
C19IMPACT_1056	Trinidad and Tobago	13-Jun-21	CARPHA	N	N	N	N	N	N	20.90		21-Jun-21	24-Jun-21	26-Jun-21	26-Jun-21	28-Jun-21
C19IMPACT_1057	Trinidad and Tobago	13-Jun-21	CARPHA	N	N	N	N	N	N	15.41		21-Jun-21	24-Jun-21	26-Jun-21	26-Jun-21	28-Jun-21
C19IMPACT_1058	Trinidad and Tobago	13-Jun-21	CARPHA	N	N	Y	N	N	N	21.90		21-Jun-21	24-Jun-21	26-Jun-21	26-Jun-21	28-Jun-21
C19IMPACT_1059	Trinidad and Tobago	14-Jun-21	CARPHA	N	N	N	N	N	N	17.48		21-Jun-21	24-Jun-21	26-Jun-21	26-Jun-21	28-Jun-21
C19IMPACT_1060	Trinidad and Tobago	15-Jun-21	CARPHA	N	N	N	N	N	N	25.05		21-Jun-21	24-Jun-21	26-Jun-21	26-Jun-21	28-Jun-21
C19IMPACT_1061	Trinidad and Tobago	15-Jun-21	CARPHA	N	N	Y	N	N	N	22.62		21-Jun-21	24-Jun-21	26-Jun-21	26-Jun-21	28-Jun-21
C19IMPACT_1062	Trinidad and Tobago	02-Jul-21	MoH	N	N	Y	N	N	Y	31.00		03-Jul-21	04-Jul-21	09-Jul-21		22-Aug-21
C19IMPACT_1063	Saint Lucia	03-May-21	CARPHA	N	N	N	N	Y	N	19.59		23-Jun-21	07-Jul-21		09-Jul-21	15-Jul-21
C19IMPACT_1064	Saint Lucia	03-May-21	CARPHA	N	N	N	N	N	N	21.67		23-Jun-21	07-Jul-21		09-Jul-21	21-Jan-22
C19IMPACT_1065	Saint Lucia	13-May-21	CARPHA	N	N	N	N	N	N	16.31		23-Jun-21	07-Jul-21		09-Jul-21	15-Jul-21
C19IMPACT_1066	Saint Lucia	13-May-21	CARPHA	N	N	N	N	N	N	13.13		23-Jun-21	07-Jul-21		09-Jul-21	15-Jul-21
C19IMPACT_1067	Saint Lucia	16-May-21	CARPHA	N	N	N	N	N	N	13.48		23-Jun-21	07-Jul-21		09-Jul-21	15-Jul-21
C19IMPACT_1068	Saint Lucia	16-May-21	CARPHA	N	N	N	N	N	N	17.46		23-Jun-21	07-Jul-21		09-Jul-21	15-Jul-21
C19IMPACT_1069	Saint Lucia	25-May-21	CARPHA	N	N	N	N	N	N	13.83		23-Jun-21	07-Jul-21		09-Jul-21	15-Jul-21
C19IMPACT_1070	Saint Lucia	25-May-21	CARPHA	N	N	N	N	N	N	15.47		23-Jun-21	07-Jul-21		09-Jul-21	21-Jan-22
C19IMPACT_1071	Barbados	29-May-21	CARPHA	N	N	N	N	N	N	24.52		28-Jun-21	07-Jul-21		09-Jul-21	
C19IMPACT_1072	Saint Lucia	31-May-21	CARPHA	N	N	N	N	N	N	14.97		23-Jun-21	07-Jul-21		09-Jul-21	15-Jul-21
C19IMPACT_1073	Barbados	01-Jun-21	CARPHA	N	N	N	N	N	N	21.37		28-Jun-21	07-Jul-21		09-Jul-21	15-Jul-21
C19IMPACT_1074	Barbados	02-Jun-21	CARPHA	N	N	N	N	N	N	18.20		28-Jun-21	07-Jul-21		09-Jul-21	15-Jul-21
C19IMPACT_1075	Barbados	03-Jun-21	CARPHA	N	N	N	N	N	N	24.32		28-Jun-21	07-Jul-21		09-Jul-21	
C19IMPACT_1076	Barbados	03-Jun-21	CARPHA	N	N	N	N	N	N	13.61		28-Jun-21	07-Jul-21		09-Jul-21	15-Jul-21
C19IMPACT_1077	Barbados	03-Jun-21	CARPHA	N	N	N	N	N	N	18.96		28-Jun-21	07-Jul-21		09-Jul-21	15-Jul-21
C19IMPACT_1078	Barbados	04-Jun-21	CARPHA	N	N	N	N	N	N	26.23		28-Jun-21	07-Jul-21		09-Jul-21	
C19IMPACT_1079	Saint Lucia	12-Jun-21	CARPHA	N	N	N	N	N	N	12.90		23-Jun-21	07-Jul-21		09-Jul-21	15-Jul-21
C19IMPACT_1080	Barbados	18-Jun-21	CARPHA	N	N	N	N	N	N	20.72		28-Jun-21	07-Jul-21		09-Jul-21	15-Jul-21
C19IMPACT_1081	Barbados	18-Jun-21	CARPHA	N	N	N	N	N	N	14.81		28-Jun-21	07-Jul-21		09-Jul-21	21-Jan-22
C19IMPACT_1082	Trinidad and Tobago	19-Jun-21	CARPHA	N	N	Y	N	N	N	18.97		23-Jun-21	07-Jul-21	09-Jul-21	09-Jul-21	22-Aug-21
C19IMPACT_1083	Barbados	20-Jun-21	CARPHA	N	N	N	N	N	N	24.52		28-Jun-21	07-Jul-21		09-Jul-21	
C19IMPACT_1084	Trinidad and Tobago	20-Jun-21	CARPHA	N	N	Y	N	N	N	18.51		23-Jun-21	07-Jul-21	09-Jul-21	09-Jul-21	22-Aug-21

C19IMPACT_1085	Trinidad and Tobago	08-Jun-21	MoH	N	N	N	N	N	N			28-Jun-21	08-Jul-21	09-Jul-21		
C19IMPACT_1086	Trinidad and Tobago	15-Jun-21	MoH	N	N	N	N	N	Y	26.00		28-Jun-21	08-Jul-21	09-Jul-21		
C19IMPACT_1087	Barbados	16-Jun-21	CARPHA	N	N	N	N	N	N	21.90		06-Jul-21	08-Jul-21		09-Jul-21	15-Jul-21
C19IMPACT_1088	Trinidad and Tobago	18-Jun-21	MoH	N	N	N	N	N	Y			23-Jun-21	08-Jul-21	09-Jul-21		22-Aug-21
C19IMPACT_1089	Barbados	22-Jun-21	CARPHA	N	N	N	N	N	N	25.79		07-Jul-21	08-Jul-21		09-Jul-21	
C19IMPACT_1090	Barbados	22-Jun-21	CARPHA	N	N	N	N	N	N	19.99		06-Jul-21	08-Jul-21		09-Jul-21	15-Jul-21
C19IMPACT_1091	Grenada	24-Jun-21	CARPHA	N	N	N	N	N	N	18.31		01-Jul-21	08-Jul-21		09-Jul-21	15-Jul-21
C19IMPACT_1092	Trinidad and Tobago	26-Jun-21	CARPHA	N	N	N	N	N	N	24.65		01-Jul-21	08-Jul-21	09-Jul-21	09-Jul-21	
C19IMPACT_1093	Trinidad and Tobago	26-Jun-21	CARPHA	N	N	N	N	N	N	25.05		01-Jul-21	08-Jul-21	09-Jul-21	09-Jul-21	
C19IMPACT_1094	Barbados	27-Jun-21	CARPHA	N	N	N	N	N	N	21.97		06-Jul-21	08-Jul-21		09-Jul-21	15-Jul-21
C19IMPACT_1095	Trinidad and Tobago	29-Jun-21	MoH	N	N	Y	N	N	Y			06-Jul-21	08-Jul-21	09-Jul-21		22-Aug-21
C19IMPACT_1096	Trinidad and Tobago	29-Jun-21	CARPHA	N	N	Y	N	Y	N	16.78		06-Jul-21	08-Jul-21	09-Jul-21	09-Jul-21	22-Aug-21
C19IMPACT_1097	Trinidad and Tobago	30-Jun-21	CARPHA	N	N	N	N	Y	N	19.15		06-Jul-21	08-Jul-21	09-Jul-21	09-Jul-21	
C19IMPACT_1098	Trinidad and Tobago	30-Jun-21	CARPHA	N	N	Y	N	N	N	22.58		06-Jul-21	08-Jul-21	09-Jul-21	09-Jul-21	22-Aug-21
C19IMPACT_1099	Barbados	03-Jul-21	CARPHA	N	N	N	N	N	N	18.69		06-Jul-21	08-Jul-21		09-Jul-21	15-Jul-21
C19IMPACT_1100	Barbados	03-Jul-21	CARPHA	N	N	N	N	N	N	20.91		06-Jul-21	08-Jul-21		09-Jul-21	15-Jul-21
C19IMPACT_1101	Barbados	03-Jul-21	CARPHA	N	N	N	N	N	N	18.62		06-Jul-21	08-Jul-21		09-Jul-21	15-Jul-21
C19IMPACT_1102	Barbados	03-Jul-21	CARPHA	N	N	N	N	N	N	14.84		06-Jul-21	08-Jul-21		09-Jul-21	15-Jul-21
C19IMPACT_1103	Trinidad and Tobago	03-Jul-21	CARPHA	N	N	N	N	N	N	24.22		06-Jul-21	08-Jul-21	09-Jul-21	09-Jul-21	
C19IMPACT_1104	Trinidad and Tobago	05-Jul-21	MoH	N	N	Y	N	N	N	16.00		06-Jul-21	08-Jul-21	09-Jul-21		22-Aug-21
C19IMPACT_1105	Trinidad and Tobago	03-May-21	CARPHA	N	N	N	N	Y	N	20.82		07-May-21	12-Jul-21	23-Jul-21	22-Jul-21	
C19IMPACT_1106	Trinidad and Tobago	03-May-21	CARPHA	N	N	N	N	N	N	16.47		07-May-21	12-Jul-21	29-Oct-21	29-Oct-21	21-Jan-22
C19IMPACT_1107	Trinidad and Tobago	03-May-21	CARPHA	N	N	N	N	N	N	24.03		07-May-21	12-Jul-21	23-Jul-21	22-Jul-21	
C19IMPACT_1108	Trinidad and Tobago	03-May-21	CARPHA	N	N	N	N	N	N	24.35		07-May-21	12-Jul-21	23-Jul-21	22-Jul-21	
C19IMPACT_1109	Trinidad and Tobago	03-May-21	CARPHA	N	N	N	N	N	N	15.57		07-May-21	12-Jul-21	23-Jul-21	22-Jul-21	23-Aug-21
C19IMPACT_1110	Trinidad and Tobago	03-May-21	CARPHA	N	N	N	N	N	N	15.77		07-May-21	12-Jul-21	23-Jul-21	22-Jul-21	23-Aug-21
C19IMPACT_1111	Trinidad and Tobago	03-May-21	CARPHA	N	N	N	N	N	N	22.86		07-May-21	12-Jul-21	23-Jul-21	22-Jul-21	
C19IMPACT_1112	Trinidad and Tobago	03-May-21	CARPHA	N	N	N	N	N	N	15.82		07-May-21	12-Jul-21	23-Jul-21	22-Jul-21	23-Aug-21
C19IMPACT_1113	Trinidad and Tobago	03-May-21	CARPHA	N	N	N	N	N	N	18.93		07-May-21	12-Jul-21	23-Jul-21	22-Jul-21	23-Aug-21
C19IMPACT_1114	Trinidad and Tobago	03-May-21	CARPHA	N	N	N	N	N	N	15.17		07-May-21	12-Jul-21	23-Jul-21	22-Jul-21	23-Aug-21
C19IMPACT_1115	Trinidad and Tobago	03-May-21	CARPHA	N	N	N	N	N	N	14.36		07-May-21	12-Jul-21	23-Jul-21	22-Jul-21	23-Aug-21
C19IMPACT_1116	Trinidad and Tobago	04-May-21	CARPHA	N	N	N	N	N	N	15.28		07-May-21	12-Jul-21	23-Jul-21	22-Jul-21	23-Aug-21
C19IMPACT_1117	Trinidad and Tobago	04-May-21	CARPHA	N	N	N	N	N	N	20.07		07-May-21	12-Jul-21	16-Jul-21	22-Jul-21	
C19IMPACT_1118	Trinidad and Tobago	04-May-21	CARPHA	N	N	N	N	N	N	17.85		07-May-21	12-Jul-21	23-Jul-21	22-Jul-21	
C19IMPACT_1119	Trinidad and Tobago	04-May-21	CARPHA	N	N	N	N	N	N	14.89		07-May-21	12-Jul-21	23-Jul-21	22-Jul-21	
C19IMPACT_1120	Trinidad and Tobago	04-May-21	CARPHA	N	N	N	N	N	N	24.37		07-May-21	12-Jul-21	23-Jul-21	22-Jul-21	23-Aug-21
C19IMPACT_1121	Trinidad and Tobago	04-May-21	CARPHA	N	N	N	N	N	N	16.30		07-May-21	12-Jul-21	23-Jul-21	22-Jul-21	23-Aug-21
C19IMPACT_1122	Trinidad and Tobago	04-May-21	CARPHA	N	N	N	N	N	N	16.56		07-May-21	12-Jul-21	23-Jul-21	22-Jul-21	23-Aug-21
C19IMPACT_1123	Trinidad and Tobago	04-May-21	CARPHA	N	N	N	N	N	N	23.89		07-May-21	12-Jul-21	23-Jul-21	22-Jul-21	23-Aug-21
C19IMPACT_1124	Trinidad and Tobago	04-May-21	CARPHA	N	N	N	N	N	N	21.13		07-May-21	12-Jul-21	23-Jul-21	22-Jul-21	23-Aug-21
C19IMPACT_1125	Trinidad and Tobago	04-May-21	CARPHA	N	N	N	N	N	N	18.37		07-May-21	12-Jul-21	23-Jul-21	22-Jul-21	23-Aug-21
C19IMPACT_1126	Trinidad and Tobago	04-May-21	CARPHA	N	N	N	N	N	N	19.46		07-May-21	12-Jul-21	23-Jul-21	22-Jul-21	23-Aug-21
C19IMPACT_1127	Trinidad and Tobago	04-May-21	CARPHA	N	N	N	N	N	N	20.43		07-May-21	12-Jul-21	23-Jul-21	22-Jul-21	23-Aug-21
C19IMPACT_1128	Trinidad and Tobago	04-May-21	CARPHA	N	N	N	N	N	N	15.82		07-May-21	12-Jul-21	23-Jul-21	22-Jul-21	23-Aug-21
C19IMPACT_1129	Trinidad and Tobago	04-May-21	CARPHA	N	N	N	N	N	N	16.86		07-May-21	12-Jul-21	23-Jul-21	22-Jul-21	23-Aug-21
C19IMPACT_1130	Trinidad and Tobago	04-May-21	CARPHA	N	N	N	N	N	N	21.11		07-May-21	12-Jul-21	23-Jul-21	22-Jul-21	23-Aug-21
C19IMPACT_1131	Trinidad and Tobago	04-May-21	CARPHA	N	N	N	N	N	N	15.72		07-May-21	12-Jul-21	23-Jul-21	22-Jul-21	23-Aug-21
C19IMPACT_1132	Trinidad and Tobago	04-May-21	CARPHA	N	N	N	N	N	N	20.05		07-May-21	12-Jul-21	23-Jul-21	22-Jul-21	23-Aug-21
C19IMPACT_1133	Trinidad and Tobago	04-May-21	CARPHA	N	N	N	N	N	N	14.45		07-May-21	12-Jul-21	23-Jul-21	22-Jul-21	23-Aug-21
C19IMPACT_1134	Trinidad and Tobago	04-May-21	CARPHA	N	N	N	N	N	N	22.21		07-May-21	12-Jul-21	23-Jul-21	22-Jul-21	23-Aug-21
C19IMPACT_1135	Trinidad and Tobago	04-May-21	CARPHA	N	N	N	N	N	N	14.89		07-May-21	12-Jul-21	23-Jul-21	22-Jul-21	23-Aug-21
C19IMPACT_1136	Trinidad and Tobago	04-May-21	CARPHA	N	N	N	N	N	N	16.33		07-May-21	12-Jul-21	23-Jul-21	22-Jul-21	23-Aug-21
C19IMPACT_1137	Trinidad and Tobago	04-May-21	CARPHA	N	N	N	N	N	N	23.53		07-May-21	12-Jul-21	23-Jul-21	22-Jul-21	23-Aug-21
C19IMPACT_1138	Trinidad and Tobago	04-May-21	CARPHA	N	N	N	N	N	N	18.89		07-May-21	12-Jul-21	29-Oct-21	29-Oct-21	21-Jan-22
C19IMPACT_1139	Trinidad and Tobago	04-May-21	CARPHA	N	N	N	N	N	N	12.66		07-May-21	12-Jul-21	29-Oct-21	29-Oct-21	21-Jan-22
C19IMPACT_1140	Trinidad and Tobago	05-May-21	CARPHA	N	N	N	N	N	N	23.56		07-May-21	12-Jul-21	23-Jul-21	22-Jul-21	23-Aug-21
C19IMPACT_1141	Trinidad and Tobago	05-May-21	CARPHA	N	N	N	N	N	N	23.75		07-May-21	12-Jul-21	23-Jul-21	22-Jul-21	23-Aug-21
C19IMPACT_1142	Trinidad and Tobago	11-Jun-21	CARPHA	N	N	N	N	N	N	15.56		21-Jun-21	12-Jul-21	13-Oct-21	06-Nov-21	21-Jan-22
C19IMPACT_1143	Trinidad and Tobago	12-Jun-21	CARPHA	N	N	N	N	N	N	25.60		21-Jun-21	12-Jul-21	13-Oct-21	06-Nov-21	
C19IMPACT_1144	Trinidad and Tobago	24-Jun-21	MoH	Y	N	Y	N	N	N	16.00		23-Jun-21	12-Jul-21	23-Jul-21		23-Aug-21
C19IMPACT_1145	Trinidad and Tobago	24-Jun-21	MoH	N	N	Y	N	Y	N	15.00		23-Jun-21	12-Jul-21	23-Jul-21		23-Aug-21
C19IMPACT_1146	Trinidad and Tobago	26-Jun-21	MoH	N	N	N	N	Y	N			01-Jul-21	12-Jul-21	23-Jul-21		23-Aug-21
C19IMPACT_1147	Trinidad and Tobago	27-Jun-21	MoH	N	N	N	N	N	N			01-Jul-21	12-Jul-21	23-Jul-21		23-Aug-21
C19IMPACT_1148	Trinidad and Tobago	27-Jun-21	MoH	N	N	N	N	N	N			01-Jul-21	12-Jul-21	23-Jul-21		23-Aug-21

C19IMPACT_1149	British Virgin Islands	28-Jun-21	CARPHA	N	N	N	N	N	N	16.21		09-Jul-21	12-Jul-21		16-Jul-21	22-Aug-21
C19IMPACT_1150	Trinidad and Tobago	28-Jun-21	MoH	N	N	N	N	N	N	29.00		01-Jul-21	12-Jul-21	23-Jul-21		
C19IMPACT_1151	British Virgin Islands	29-Jun-21	CARPHA	N	N	N	N	N	N	14.65		09-Jul-21	12-Jul-21		16-Jul-21	
C19IMPACT_1152	British Virgin Islands	29-Jun-21	CARPHA	N	N	N	N	N	N	17.87		09-Jul-21	12-Jul-21		16-Jul-21	
C19IMPACT_1153	British Virgin Islands	30-Jun-21	CARPHA	N	N	N	N	N	N	15.97		09-Jul-21	12-Jul-21		16-Jul-21	22-Aug-21
C19IMPACT_1154	British Virgin Islands	30-Jun-21	CARPHA	N	N	N	N	N	N	17.29		09-Jul-21	12-Jul-21		16-Jul-21	
C19IMPACT_1155	British Virgin Islands	30-Jun-21	CARPHA	N	N	N	N	N	N	16.30		09-Jul-21	12-Jul-21		16-Jul-21	
C19IMPACT_1156	British Virgin Islands	30-Jun-21	CARPHA	N	N	N	N	N	N	15.31		09-Jul-21	12-Jul-21		16-Jul-21	
C19IMPACT_1157	British Virgin Islands	30-Jun-21	CARPHA	N	N	N	N	N	N	16.85		09-Jul-21	12-Jul-21		16-Jul-21	
C19IMPACT_1158	British Virgin Islands	30-Jun-21	CARPHA	N	N	N	N	N	N	18.32		09-Jul-21	12-Jul-21		16-Jul-21	22-Aug-21
C19IMPACT_1159	British Virgin Islands	30-Jun-21	CARPHA	N	N	N	N	N	N	16.76		09-Jul-21	12-Jul-21		16-Jul-21	
C19IMPACT_1160	British Virgin Islands	01-Jul-21	CARPHA	N	N	N	N	N	N	16.68		09-Jul-21	12-Jul-21		16-Jul-21	
C19IMPACT_1161	Anguilla	01-Jul-21	CARPHA	N	N	N	N	N	N	19.18		09-Jul-21	12-Jul-21		16-Jul-21	22-Aug-21
C19IMPACT_1162	British Virgin Islands	01-Jul-21	CARPHA	N	N	N	N	N	N	19.29		09-Jul-21	12-Jul-21		16-Jul-21	
C19IMPACT_1163	British Virgin Islands	01-Jul-21	CARPHA	N	N	N	N	N	N	18.08		09-Jul-21	12-Jul-21		16-Jul-21	22-Aug-21
C19IMPACT_1164	British Virgin Islands	01-Jul-21	CARPHA	N	N	N	N	N	N	17.16		09-Jul-21	12-Jul-21		16-Jul-21	22-Aug-21
C19IMPACT_1165	British Virgin Islands	01-Jul-21	CARPHA	N	N	N	N	N	N	17.81		09-Jul-21	12-Jul-21		16-Jul-21	
C19IMPACT_1166	British Virgin Islands	01-Jul-21	CARPHA	N	N	N	N	N	N	16.35		09-Jul-21	12-Jul-21		16-Jul-21	22-Aug-21
C19IMPACT_1167	British Virgin Islands	01-Jul-21	CARPHA	N	N	N	N	N	N	17.45		09-Jul-21	12-Jul-21		16-Jul-21	22-Aug-21
C19IMPACT_1168	British Virgin Islands	01-Jul-21	CARPHA	N	N	N	N	N	N	15.02		09-Jul-21	12-Jul-21		16-Jul-21	22-Aug-21
C19IMPACT_1169	British Virgin Islands	01-Jul-21	CARPHA	N	N	N	N	N	N	13.23		09-Jul-21	12-Jul-21		16-Jul-21	22-Aug-21
C19IMPACT_1170	British Virgin Islands	01-Jul-21	CARPHA	N	N	N	N	N	N	12.54		09-Jul-21	12-Jul-21		16-Jul-21	22-Aug-21
C19IMPACT_1171	British Virgin Islands	02-Jul-21	CARPHA	N	N	N	N	N	N	18.87		09-Jul-21	12-Jul-21		16-Jul-21	
C19IMPACT_1172	British Virgin Islands	02-Jul-21	CARPHA	N	N	N	N	N	N	21.73		09-Jul-21	12-Jul-21		16-Jul-21	
C19IMPACT_1173	British Virgin Islands	02-Jul-21	CARPHA	N	N	N	N	N	N	15.86		09-Jul-21	12-Jul-21		16-Jul-21	22-Aug-21
C19IMPACT_1174	British Virgin Islands	02-Jul-21	CARPHA	N	N	N	N	N	N	15.04		09-Jul-21	12-Jul-21		16-Jul-21	
C19IMPACT_1175	British Virgin Islands	02-Jul-21	CARPHA	N	N	N	N	N	N	18.94		09-Jul-21	12-Jul-21		16-Jul-21	22-Aug-21
C19IMPACT_1176	British Virgin Islands	02-Jul-21	CARPHA	N	N	N	N	N	N	17.64		09-Jul-21	12-Jul-21		16-Jul-21	
C19IMPACT_1177	British Virgin Islands	02-Jul-21	CARPHA	N	N	N	N	N	N	17.98		09-Jul-21	12-Jul-21		16-Jul-21	
C19IMPACT_1178	British Virgin Islands	02-Jul-21	CARPHA	N	N	N	N	N	N	14.71		09-Jul-21	12-Jul-21		16-Jul-21	
C19IMPACT_1179	British Virgin Islands	02-Jul-21	CARPHA	N	N	N	N	N	N	14.47		09-Jul-21	12-Jul-21		16-Jul-21	22-Aug-21
C19IMPACT_1180	British Virgin Islands	02-Jul-21	CARPHA	N	N	N	N	N	N	14.19		09-Jul-21	12-Jul-21		16-Jul-21	22-Aug-21
C19IMPACT_1181	British Virgin Islands	02-Jul-21	CARPHA	N	N	N	N	N	N	16.00		09-Jul-21	12-Jul-21		16-Jul-21	22-Aug-21
C19IMPACT_1182	British Virgin Islands	02-Jul-21	CARPHA	N	N	N	N	N	N	19.03		09-Jul-21	12-Jul-21		16-Jul-21	
C19IMPACT_1183	British Virgin Islands	02-Jul-21	CARPHA	N	N	N	N	N	N	13.37		09-Jul-21	12-Jul-21		16-Jul-21	22-Aug-21
C19IMPACT_1184	British Virgin Islands	02-Jul-21	CARPHA	N	N	N	N	N	N	14.95		09-Jul-21	12-Jul-21		16-Jul-21	22-Aug-21
C19IMPACT_1185	British Virgin Islands	02-Jul-21	CARPHA	N	N	N	N	N	N	16.25		09-Jul-21	12-Jul-21		16-Jul-21	22-Aug-21
C19IMPACT_1186	British Virgin Islands	02-Jul-21	CARPHA	N	N	N	N	N	N	17.49		09-Jul-21	12-Jul-21		16-Jul-21	22-Aug-21
C19IMPACT_1187	British Virgin Islands	02-Jul-21	CARPHA	N	N	N	N	N	N	17.74		09-Jul-21	12-Jul-21		16-Jul-21	22-Aug-21
C19IMPACT_1188	British Virgin Islands	02-Jul-21	CARPHA	N	N	N	N	N	N	13.32		09-Jul-21	12-Jul-21		16-Jul-21	22-Aug-21
C19IMPACT_1189	Trinidad and Tobago	02-Jul-21	MoH	N	N	N	N	N	N	20.00		09-Jul-21	12-Jul-21	16-Jul-21		
C19IMPACT_1190	Trinidad and Tobago	05-Jul-21	CARPHA	N	N	N	N	N	N	27.74		09-Jul-21	12-Jul-21	16-Jul-21	16-Jul-21	
C19IMPACT_1191	Trinidad and Tobago	06-Jul-21	MoH	N	N	N	N	N	N	21.64		09-Jul-21	12-Jul-21	16-Jul-21		
C19IMPACT_1192	Trinidad and Tobago	09-Jul-21	MoH	N	N	N	N	N	N			09-Jul-21	12-Jul-21	16-Jul-21		22-Aug-21
C19IMPACT_1193	Trinidad and Tobago	03-May-21	CARPHA	N	N	N	N	N	N	19.37		07-May-21	17-Jul-21	23-Jul-21	22-Jul-21	21-Jan-22
C19IMPACT_1194	Trinidad and Tobago	03-May-21	CARPHA	N	N	N	N	N	N	15.04		07-May-21	17-Jul-21	23-Jul-21	22-Jul-21	21-Jan-22
C19IMPACT_1195	Trinidad and Tobago	03-May-21	CARPHA	N	N	N	N	N	N	16.78		07-May-21	17-Jul-21	23-Jul-21	22-Jul-21	21-Jan-22
C19IMPACT_1196	Trinidad and Tobago	03-May-21	CARPHA	N	N	Y	N	N	N	14.78		07-May-21	17-Jul-21	23-Jul-21	22-Jul-21	21-Jan-22
C19IMPACT_1197	Trinidad and Tobago	03-May-21	CARPHA	N	N	Y	N	N	N	19.52		07-May-21	17-Jul-21	23-Jul-21	22-Jul-21	21-Jan-22
C19IMPACT_1198	Trinidad and Tobago	05-May-21	CARPHA	N	N	N	N	N	N	17.36		07-May-21	17-Jul-21	18-Jun-21	21-Jul-21	
C19IMPACT_1199	Trinidad and Tobago	04-May-21	CARPHA	N	N	N	N	N	N	15.11		07-May-21	17-Jul-21	23-Jul-21	21-Jul-21	21-Jan-22
C19IMPACT_1200	Antigua and Barbuda	05-May-21	CARPHA	N	N	N	N	N	N	19.72		12-Jul-21	17-Jul-21		21-Jul-21	23-Aug-21
C19IMPACT_1201	Antigua and Barbuda	05-May-21	CARPHA	N	N	N	N	N	N	15.50		12-Jul-21	17-Jul-21		21-Jul-21	23-Aug-21
C19IMPACT_1202	Antigua and Barbuda	05-May-21	CARPHA	N	N	N	N	N	N	18.12		12-Jul-21	17-Jul-21		21-Jul-21	23-Aug-21
C19IMPACT_1203	Trinidad and Tobago	05-May-21	CARPHA	N	N	N	N	N	N	18.23		07-May-21	17-Jul-21	23-Jul-21	21-Jul-21	21-Jan-22
C19IMPACT_1204	Trinidad and Tobago	05-May-21	CARPHA	N	N	N	N	N	N	18.21		07-May-21	17-Jul-21	23-Jul-21	21-Jul-21	21-Jan-22
C19IMPACT_1205	Trinidad and Tobago	05-May-21	CARPHA	N	N	Y	N	N	N	22.14		07-May-21	17-Jul-21	23-Jul-21	21-Jul-21	21-Jan-22
C19IMPACT_1206	Trinidad and Tobago	05-May-21	CARPHA	N	N	N	N	N	N	24.00		07-May-21	17-Jul-21	23-Jul-21	21-Jul-21	21-Jan-22
C19IMPACT_1207	Trinidad and Tobago	05-May-21	CARPHA	N	N	Y	N	N	N	17.83		07-May-21	17-Jul-21	23-Jul-21	21-Jul-21	21-Jan-22
C19IMPACT_1208	Trinidad and Tobago	05-May-21	CARPHA	N	N	N	N	N	N	13.95		07-May-21	17-Jul-21	23-Jul-21	21-Jul-21	21-Jan-22
C19IMPACT_1209	Trinidad and Tobago	05-May-21	CARPHA	N	N	N	N	N	N	19.12		07-May-21	17-Jul-21	23-Jul-21	21-Jul-21	21-Jan-22
C19IMPACT_1210	Trinidad and Tobago	05-May-21	CARPHA	N	N	N	N	N	N	19.03		07-May-21	17-Jul-21	23-Jul-21	21-Jul-21	21-Jan-22
C19IMPACT_1211	Trinidad and Tobago	05-May-21	CARPHA	N	N	N	N	N	N	17.73		07-May-21	17-Jul-21	23-Jul-21	21-Jul-21	21-Jan-22
C19IMPACT_1212	Trinidad and Tobago	05-May-21	CARPHA	N	N	N	N	N	N	20.84		07-May-21	17-Jul-21	23-Jul-21	21-Jul-21	21-Jan-22

C19IMPACT_1213	Trinidad and Tobago	05-May-21	CARPHA	N	N	N	N	N	N	18.90		07-May-21	17-Jul-21	23-Jul-21	21-Jul-21	21-Jan-22
C19IMPACT_1214	Trinidad and Tobago	05-May-21	CARPHA	N	N	N	N	N	N	18.16		07-May-21	17-Jul-21	23-Jul-21	21-Jul-21	21-Jan-22
C19IMPACT_1215	Trinidad and Tobago	07-May-21	CARPHA	N	N	N	N	N	N	23.56		11-May-21	17-Jul-21	23-Jul-21	22-Jul-21	21-Jan-22
C19IMPACT_1216	Trinidad and Tobago	07-May-21	CARPHA	N	N	N	N	N	N	22.48		11-May-21	17-Jul-21	23-Jul-21	21-Jul-21	
C19IMPACT_1217	Trinidad and Tobago	07-May-21	CARPHA	N	N	N	N	N	N	20.00		11-May-21	17-Jul-21	23-Jul-21	21-Jul-21	
C19IMPACT_1218	Trinidad and Tobago	07-May-21	CARPHA	N	N	N	N	N	N	14.79		11-May-21	17-Jul-21	23-Jul-21	21-Jul-21	21-Jan-22
C19IMPACT_1219	Trinidad and Tobago	07-May-21	CARPHA	N	N	N	N	N	N	20.77		11-May-21	17-Jul-21	23-Jul-21	22-Jul-21	21-Jan-22
C19IMPACT_1220	Trinidad and Tobago	07-May-21	CARPHA	N	N	N	N	N	N	15.32		11-May-21	17-Jul-21	23-Jul-21	22-Jul-21	21-Jan-22
C19IMPACT_1221	Trinidad and Tobago	07-May-21	CARPHA	N	N	N	N	N	N	21.05		11-May-21	17-Jul-21	23-Jul-21	22-Jul-21	21-Jan-22
C19IMPACT_1222	Trinidad and Tobago	07-May-21	CARPHA	N	N	N	N	N	N	13.20		11-May-21	17-Jul-21	23-Jul-21	22-Jul-21	21-Jan-22
C19IMPACT_1223	Trinidad and Tobago	07-May-21	CARPHA	N	N	N	N	N	N	17.12		11-May-21	17-Jul-21	23-Jul-21	22-Jul-21	21-Jan-22
C19IMPACT_1224	Trinidad and Tobago	07-May-21	CARPHA	N	N	N	N	N	N	18.75		11-May-21	17-Jul-21	23-Jul-21	22-Jul-21	21-Jan-22
C19IMPACT_1225	Trinidad and Tobago	07-May-21	CARPHA	N	N	N	N	N	N	19.84		11-May-21	17-Jul-21	23-Jul-21	22-Jul-21	21-Jan-22
C19IMPACT_1226	Trinidad and Tobago	07-May-21	CARPHA	N	N	N	N	N	N	18.64		11-May-21	17-Jul-21	23-Jul-21	22-Jul-21	21-Jan-22
C19IMPACT_1227	Trinidad and Tobago	07-May-21	CARPHA	N	N	N	N	N	N	17.72		11-May-21	17-Jul-21	23-Jul-21	22-Jul-21	21-Jan-22
C19IMPACT_1228	Trinidad and Tobago	07-May-21	CARPHA	N	N	N	N	N	N	16.31		11-May-21	17-Jul-21	23-Jul-21	22-Jul-21	21-Jan-22
C19IMPACT_1229	Trinidad and Tobago	07-May-21	CARPHA	N	N	N	N	N	N	15.55		11-May-21	17-Jul-21	23-Jul-21	22-Jul-21	21-Jan-22
C19IMPACT_1230	Trinidad and Tobago	07-May-21	CARPHA	N	N	N	N	N	N	12.27		11-May-21	17-Jul-21	23-Jul-21	22-Jul-21	21-Jan-22
C19IMPACT_1231	Trinidad and Tobago	07-May-21	CARPHA	N	N	N	N	N	N	14.83		11-May-21	17-Jul-21	23-Jul-21	22-Jul-21	21-Jan-22
C19IMPACT_1232	Trinidad and Tobago	08-May-21	CARPHA	N	N	Y	N	N	N	19.80		11-May-21	17-Jul-21	23-Jul-21	22-Jul-21	21-Jan-22
C19IMPACT_1233	Trinidad and Tobago	08-May-21	CARPHA	N	N	N	N	N	N	14.57		11-May-21	17-Jul-21	23-Jul-21	22-Jul-21	21-Jan-22
C19IMPACT_1234	Antigua and Barbuda	09-May-21	CARPHA	N	N	N	N	N	N	23.17		12-Jul-21	17-Jul-21		21-Jul-21	23-Aug-21
C19IMPACT_1235	Trinidad and Tobago	09-May-21	CARPHA	N	N	Y	N	N	N	17.15		11-May-21	17-Jul-21	23-Jul-21	22-Jul-21	21-Jan-22
C19IMPACT_1236	Trinidad and Tobago	12-May-21	CARPHA	N	N	N	N	N	N	15.60		28-May-21	17-Jul-21	23-Jul-21	21-Jul-21	
C19IMPACT_1237	Trinidad and Tobago	12-May-21	CARPHA	N	N	N	N	N	N	24.83		28-May-21	17-Jul-21	23-Jul-21	21-Jul-21	
C19IMPACT_1238	Trinidad and Tobago	12-May-21	CARPHA	N	N	N	N	N	N	13.84		28-May-21	17-Jul-21	23-Jul-21	21-Jul-21	23-Aug-21
C19IMPACT_1239	Trinidad and Tobago	12-May-21	CARPHA	N	N	N	N	N	N	23.55		28-May-21	17-Jul-21	23-Jul-21	21-Jul-21	
C19IMPACT_1240	Trinidad and Tobago	12-May-21	CARPHA	N	N	N	N	N	N	16.11		28-May-21	17-Jul-21	23-Jul-21	21-Jul-21	21-Jan-22
C19IMPACT_1241	Trinidad and Tobago	12-May-21	CARPHA	N	N	N	N	N	N	12.88		28-May-21	17-Jul-21	23-Jul-21	21-Jul-21	21-Jan-22
C19IMPACT_1242	Trinidad and Tobago	12-May-21	CARPHA	N	N	N	N	N	N	18.98		28-May-21	17-Jul-21	23-Jul-21	21-Jul-21	21-Jan-22
C19IMPACT_1243	Antigua and Barbuda	14-May-21	CARPHA	N	N	N	N	N	N	14.41		12-Jul-21	17-Jul-21		21-Jul-21	23-Aug-21
C19IMPACT_1244	Trinidad and Tobago	14-May-21	CARPHA	N	N	N	N	N	N	14.46		28-May-21	17-Jul-21	23-Jul-21	21-Jul-21	21-Jan-22
C19IMPACT_1245	Antigua and Barbuda	20-May-21	CARPHA	N	N	N	N	N	N	14.09		12-Jul-21	17-Jul-21		21-Jul-21	23-Aug-21
C19IMPACT_1246	Antigua and Barbuda	02-Jul-21	CARPHA	N	N	N	N	N	N	17.89		12-Jul-21	17-Jul-21		21-Jul-21	23-Aug-21
C19IMPACT_1247	Trinidad and Tobago	06-Jul-21	CARPHA	N	N	N	N	N	Y	19.51		14-Jul-21	17-Jul-21	23-Jul-21	21-Jul-21	
C19IMPACT_1248	Montserrat	08-Jul-21	CARPHA	N	N	N	N	N	Y	21.13		16-Jul-21	17-Jul-21		21-Jul-21	23-Aug-21
C19IMPACT_1249	Trinidad and Tobago	09-Jul-21	MoH	N	N	N	N	N	Y	23.00		13-Jul-21	17-Jul-21	23-Jul-21		
C19IMPACT_1250	Trinidad and Tobago	09-Jul-21	CARPHA	N	N	N	N	N	Y	16.95		14-Jul-21	17-Jul-21	23-Jul-21	21-Jul-21	
C19IMPACT_1251	Trinidad and Tobago	09-Jul-21	MoH	N	N	Y	N	N	Y	15.00		13-Jul-21	17-Jul-21	23-Jul-21		23-Aug-21
C19IMPACT_1252	Trinidad and Tobago	09-Jul-21	MoH	N	N	Y	N	Y	Y	19.00		13-Jul-21	17-Jul-21	23-Jul-21		23-Aug-21
C19IMPACT_1253	Trinidad and Tobago	09-Jul-21	MoH	N	N	N	N	Y	Y	18.00		13-Jul-21	17-Jul-21	23-Jul-21		
C19IMPACT_1254	Trinidad and Tobago	13-Jul-21	CARPHA	N	N	N	N	N	Y	10.98		16-Jul-21	17-Jul-21	23-Jul-21	21-Jul-21	
C19IMPACT_1255	Saint Vincent and the Grenadines	13-Jul-21	CARPHA	N	N	N	N	N	Y	17.81		16-Jul-21	17-Jul-21		21-Jul-21	
C19IMPACT_1256	Trinidad and Tobago	14-Jul-21	MoH	N	N	N	N	N	N	18.00		16-Jul-21	17-Jul-21	23-Jul-21		
C19IMPACT_1257	Trinidad and Tobago	14-Jul-21	MoH	N	N	Y	N	N	Y	17.00		16-Jul-21	17-Jul-21	23-Jul-21		23-Aug-21
C19IMPACT_1258	Trinidad and Tobago	15-Jul-21	MoH	N	N	N	N	Y	N			16-Jul-21	17-Jul-21	23-Jul-21		
C19IMPACT_1259	Barbados	04-Jul-21	CARPHA	N	N	N	N	N	Y	22.22		20-Jul-21	22-Jul-21		26-Jul-21	
C19IMPACT_1260	Barbados	04-Jul-21	CARPHA	N	N	N	N	N	Y	19.04		20-Jul-21	22-Jul-21		26-Jul-21	23-Aug-21
C19IMPACT_1261	Barbados	06-Jul-21	CARPHA	N	N	N	N	N	Y	17.40		20-Jul-21	22-Jul-21		26-Jul-21	23-Aug-21
C19IMPACT_1262	Barbados	06-Jul-21	CARPHA	N	N	N	N	N	Y	17.29		20-Jul-21	22-Jul-21		26-Jul-21	23-Aug-21
C19IMPACT_1263	Barbados	07-Jul-21	CARPHA	N	N	N	N	N	Y	15.09		20-Jul-21	22-Jul-21		26-Jul-21	23-Aug-21
C19IMPACT_1264	Barbados	10-Jul-21	CARPHA	N	N	N	N	N	Y	25.06		20-Jul-21	22-Jul-21		26-Jul-21	
C19IMPACT_1265	Barbados	10-Jul-21	CARPHA	N	N	N	N	N	Y	25.24		20-Jul-21	22-Jul-21		26-Jul-21	
C19IMPACT_1266	Barbados	10-Jul-21	CARPHA	N	N	N	N	N	Y	15.00		20-Jul-21	22-Jul-21		26-Jul-21	23-Aug-21
C19IMPACT_1267	Barbados	10-Jul-21	CARPHA	N	N	N	N	N	Y	19.29		20-Jul-21	22-Jul-21		26-Jul-21	23-Aug-21
C19IMPACT_1268	Barbados	10-Jul-21	CARPHA	N	N	N	N	N	Y	18.27		20-Jul-21	22-Jul-21		26-Jul-21	23-Aug-21
C19IMPACT_1269	Barbados	10-Jul-21	CARPHA	N	N	N	N	N	Y	15.62		20-Jul-21	22-Jul-21		26-Jul-21	23-Aug-21
C19IMPACT_1270	Barbados	10-Jul-21	CARPHA	N	N	N	N	N	N	21.78		20-Jul-21	22-Jul-21		29-Oct-21	21-Jan-22
C19IMPACT_1271	Barbados	11-Jul-21	CARPHA	N	N	N	N	N	Y	14.87		20-Jul-21	22-Jul-21		26-Jul-21	23-Aug-21
C19IMPACT_1272	Trinidad and Tobago	14-Jul-21	CARPHA	N	N	Y	N	N	Y	14.87		20-Jul-21	22-Jul-21	13-Oct-21	26-Jul-21	23-Aug-21
C19IMPACT_1273	Trinidad and Tobago	14-Jul-21	CARPHA	N	N	Y	N	N	Y	21.84		20-Jul-21	22-Jul-21	13-Oct-21	26-Jul-21	23-Aug-21
C19IMPACT_1274	Trinidad and Tobago	14-Jul-21	CARPHA	N	N	Y	N	N	Y	17.95		20-Jul-21	22-Jul-21	13-Oct-21	26-Jul-21	23-Aug-21
C19IMPACT_1275	Trinidad and Tobago	17-Jul-21	MoH	Y	N	Y	N	N	Y	19.00		20-Jul-21	22-Jul-21	13-Oct-21		23-Aug-21
C19IMPACT_1276	Trinidad and Tobago	17-Jul-21	MoH	Y	N	Y	N	Y	Y	15.00		20-Jul-21	22-Jul-21	13-Oct-21		23-Aug-21

C19IMPACT_1277	Trinidad and Tobago	17-Jul-21	MoH	Y	N	Y	N	Y	Y	25.00		20-Jul-21	22-Jul-21	13-Oct-21		23-Aug-21
C19IMPACT_1278	Trinidad and Tobago	18-Jul-21	MoH	N	N	N	N	Y	Y	23.00		20-Jul-21	22-Jul-21	13-Oct-21		
C19IMPACT_1279	Trinidad and Tobago	18-Jul-21	MoH	N	N	Y	N	N	Y	16.00		20-Jul-21	22-Jul-21	13-Oct-21		23-Aug-21
C19IMPACT_1280	Trinidad and Tobago	19-Jul-21	MoH	N	N	N	N	Y	Y			20-Jul-21	22-Jul-21	13-Oct-21		
C19IMPACT_1281	Turks and Caicos Islands	25-Jun-21	CARPHA	N	N	N	N	N	Y	19.45		22-Jul-21	28-Jul-21		30-Jul-21	23-Aug-21
C19IMPACT_1282	Turks and Caicos Islands	05-Jul-21	CARPHA	N	N	N	N	N	Y	18.29		22-Jul-21	28-Jul-21		30-Jul-21	23-Aug-21
C19IMPACT_1283	Turks and Caicos Islands	08-Jul-21	CARPHA	N	N	N	N	N	Y	17.83		22-Jul-21	28-Jul-21		30-Jul-21	
C19IMPACT_1284	Antigua and Barbuda	10-Jul-21	CARPHA	N	N	N	N	N	Y	17.71		22-Jul-21	28-Jul-21		30-Jul-21	
C19IMPACT_1285	Turks and Caicos Islands	11-Jul-21	CARPHA	N	N	N	N	N	Y	18.30		22-Jul-21	28-Jul-21		30-Jul-21	
C19IMPACT_1286	Turks and Caicos Islands	12-Jul-21	CARPHA	N	N	N	N	N	Y	16.51		22-Jul-21	28-Jul-21		30-Jul-21	23-Aug-21
C19IMPACT_1287	Turks and Caicos Islands	12-Jul-21	CARPHA	N	N	N	N	N	Y	16.64		22-Jul-21	28-Jul-21		30-Jul-21	23-Aug-21
C19IMPACT_1288	Turks and Caicos Islands	12-Jul-21	CARPHA	N	N	N	N	N	Y	19.22		22-Jul-21	28-Jul-21		30-Jul-21	23-Aug-21
C19IMPACT_1289	Turks and Caicos Islands	12-Jul-21	CARPHA	N	N	N	N	N	Y	22.26		22-Jul-21	28-Jul-21		30-Jul-21	23-Aug-21
C19IMPACT_1290	Antigua and Barbuda	13-Jul-21	CARPHA	N	N	N	N	N	Y	26.67		22-Jul-21	28-Jul-21		30-Jul-21	
C19IMPACT_1291	Antigua and Barbuda	14-Jul-21	CARPHA	N	N	N	N	N	Y	15.34		22-Jul-21	28-Jul-21		30-Jul-21	23-Aug-21
C19IMPACT_1292	Antigua and Barbuda	14-Jul-21	CARPHA	N	N	N	N	N	Y	19.24		22-Jul-21	28-Jul-21		30-Jul-21	23-Aug-21
C19IMPACT_1293	Trinidad and Tobago	19-Jul-21	CARPHA	N	N	N	N	N	Y	19.70		22-Jul-21	28-Jul-21	30-Jul-21	30-Jul-21	
C19IMPACT_1294	Trinidad and Tobago	19-Jul-21	CARPHA	N	N	N	N	N	Y	23.57		22-Jul-21	28-Jul-21	30-Jul-21	30-Jul-21	23-Aug-21
C19IMPACT_1295	Trinidad and Tobago	19-Jul-21	CARPHA	Y	N	Y	N	N	Y	20.41		22-Jul-21	28-Jul-21	30-Jul-21	30-Jul-21	23-Aug-21
C19IMPACT_1296	Trinidad and Tobago	19-Jul-21	CARPHA	N	Y	Y	N	Y	Y	17.89		22-Jul-21	28-Jul-21	30-Jul-21	30-Jul-21	23-Aug-21
C19IMPACT_1297	Trinidad and Tobago	21-Jul-21	MoH	N	N	N	N	Y	Y	12.00		22-Jul-21	28-Jul-21	30-Jul-21		
C19IMPACT_1298	Trinidad and Tobago	21-Jul-21	MoH	N	N	N	N	N	Y	15.00		22-Jul-21	28-Jul-21	30-Jul-21		
C19IMPACT_1299	Trinidad and Tobago	21-Jul-21	MoH	N	N	N	N	N	Y	14.00		23-Jul-21	28-Jul-21	30-Jul-21		
C19IMPACT_1300	Trinidad and Tobago	22-Jul-21	MoH	N	N	N	N	N	Y			23-Jul-21	28-Jul-21	30-Jul-21		
C19IMPACT_1301	Trinidad and Tobago	22-Jul-21	MoH	N	N	N	N	N	Y			23-Jul-21	28-Jul-21	30-Jul-21		
C19IMPACT_1302	Trinidad and Tobago	22-Jul-21	MoH	N	N	N	N	N	Y			23-Jul-21	28-Jul-21	13-Oct-21		
C19IMPACT_1303	British Virgin Islands	18-Jul-21	CARPHA	N	N	N	N	N	N	24.54		27-Jul-21	29-Jul-21		30-Jul-21	
C19IMPACT_1304	British Virgin Islands	18-Jul-21	CARPHA	N	N	N	N	N	N	16.86		27-Jul-21	29-Jul-21		30-Jul-21	21-Jan-22
C19IMPACT_1305	Anguilla	18-Jul-21	CARPHA	N	N	N	N	N	N	14.53		27-Jul-21	29-Jul-21		30-Jul-21	23-Aug-21
C19IMPACT_1306	Anguilla	18-Jul-21	CARPHA	N	N	N	N	N	N	18.35		27-Jul-21	29-Jul-21		30-Jul-21	23-Aug-21
C19IMPACT_1307	Trinidad and Tobago	19-Jul-21	MoH	N	N	N	N	N	Y		11.15	27-Jul-21	29-Jul-21	30-Jul-21		
C19IMPACT_1308	Trinidad and Tobago	19-Jul-21	MoH	N	N	N	N	N	Y		4.27	27-Jul-21	29-Jul-21	30-Jul-21		21-Jan-22
C19IMPACT_1309	Trinidad and Tobago	19-Jul-21	MoH	N	N	N	N	N	Y		5.86	27-Jul-21	29-Jul-21	30-Jul-21		21-Jan-22
C19IMPACT_1310	Trinidad and Tobago	19-Jul-21	MoH	N	N	N	N	N	N	15.00		27-Jul-21	29-Jul-21	30-Jul-21		21-Jan-22
C19IMPACT_1311	British Virgin Islands	19-Jul-21	CARPHA	N	N	N	N	N	N	23.44		27-Jul-21	29-Jul-21		30-Jul-21	21-Jan-22
C19IMPACT_1312	British Virgin Islands	19-Jul-21	CARPHA	N	N	N	N	N	N	11.17		27-Jul-21	29-Jul-21		30-Jul-21	21-Jan-22
C19IMPACT_1313	British Virgin Islands	19-Jul-21	CARPHA	N	N	N	N	N	N	19.16		27-Jul-21	29-Jul-21		30-Jul-21	21-Jan-22
C19IMPACT_1314	British Virgin Islands	19-Jul-21	CARPHA	N	N	N	N	N	N	16.65		27-Jul-21	29-Jul-21		30-Jul-21	21-Jan-22
C19IMPACT_1315	British Virgin Islands	19-Jul-21	CARPHA	N	N	N	N	N	N	16.25		27-Jul-21	29-Jul-21		30-Jul-21	21-Jan-22
C19IMPACT_1316	Saint Vincent and the Grenadines	19-Jul-21	CARPHA	N	N	N	N	N	N	15.68		27-Jul-21	29-Jul-21		30-Jul-21	21-Jan-22
C19IMPACT_1317	Saint Vincent and the Grenadines	19-Jul-21	CARPHA	N	N	N	N	N	N	20.62		27-Jul-21	29-Jul-21		30-Jul-21	21-Jan-22
C19IMPACT_1318	Saint Vincent and the Grenadines	19-Jul-21	CARPHA	N	N	N	N	N	N	14.98		27-Jul-21	29-Jul-21		30-Jul-21	21-Jan-22
C19IMPACT_1319	Trinidad and Tobago	19-Jul-21	MoH	N	N	N	N	N	N		19.84	27-Jul-21	29-Jul-21	13-Oct-21		
C19IMPACT_1320	Trinidad and Tobago	20-Jul-21	CARPHA	N	N	N	N	N	Y	21.43		23-Jul-21	29-Jul-21	30-Jul-21	30-Jul-21	
C19IMPACT_1321	Trinidad and Tobago	20-Jul-21	CARPHA	N	N	N	N	N	Y	24.19		23-Jul-21	29-Jul-21	30-Jul-21	30-Jul-21	
C19IMPACT_1322	Trinidad and Tobago	20-Jul-21	CARPHA	N	N	N	N	N	Y	24.47		23-Jul-21	29-Jul-21	30-Jul-21	30-Jul-21	
C19IMPACT_1323	Trinidad and Tobago	20-Jul-21	MoH	N	N	N	N	N	Y		8.00	27-Jul-21	29-Jul-21	30-Jul-21		21-Jan-22
C19IMPACT_1324	Trinidad and Tobago	20-Jul-21	MoH	N	N	N	N	N	Y		3.00	27-Jul-21	29-Jul-21	30-Jul-21		21-Jan-22
C19IMPACT_1325	Trinidad and Tobago	20-Jul-21	MoH	N	N	N	N	N	Y		5.00	27-Jul-21	29-Jul-21	30-Jul-21		21-Jan-22
C19IMPACT_1326	Saint Vincent and the Grenadines	20-Jul-21	CARPHA	N	N	N	N	N	N	17.31		27-Jul-21	29-Jul-21		30-Jul-21	21-Jan-22
C19IMPACT_1327	Trinidad and Tobago	20-Jul-21	MoH	N	N	N	N	N	Y		8.86	27-Jul-21	29-Jul-21	30-Jul-21		21-Jan-22
C19IMPACT_1328	Trinidad and Tobago	20-Jul-21	MoH	N	N	N	N	N	Y		6.37	27-Jul-21	29-Jul-21	30-Jul-21		21-Jan-22
C19IMPACT_1329	Trinidad and Tobago	21-Jul-21	MoH	N	N	N	N	N	Y		4.00	27-Jul-21	29-Jul-21	30-Jul-21		21-Jan-22
C19IMPACT_1330	Trinidad and Tobago	21-Jul-21	MoH	N	N	N	N	N	Y		13.00	27-Jul-21	29-Jul-21	30-Jul-21		21-Jan-22
C19IMPACT_1331	Trinidad and Tobago	21-Jul-21	CARPHA	N	N	Y	N	N	N	22.21		27-Jul-21	29-Jul-21	30-Jul-21	30-Jul-21	21-Jan-22
C19IMPACT_1332	Trinidad and Tobago	21-Jul-21	MoH	N	N	N	N	Y	Y		21.00	27-Jul-21	29-Jul-21	30-Jul-21		
C19IMPACT_1333	Trinidad and Tobago	22-Jul-21	MoH	N	N	N	N	N	Y	15.24		23-Jul-21	29-Jul-21	30-Jul-21		21-Jan-22
C19IMPACT_1334	Trinidad and Tobago	22-Jul-21	MoH	N	N	N	N	N	Y	15.32		23-Jul-21	29-Jul-21	30-Jul-21		21-Jan-22
C19IMPACT_1335	Trinidad and Tobago	22-Jul-21	MoH	N	N	Y	N	N	N	15.00		27-Jul-21	29-Jul-21	30-Jul-21		21-Jan-22
C19IMPACT_1336	Trinidad and Tobago	22-Jul-21	MoH	Y	N	Y	N	Y	N	15.00		27-Jul-21	29-Jul-21	30-Jul-21		21-Jan-22
C19IMPACT_1337	Trinidad and Tobago	22-Jul-21	MoH	N	N	Y	N	Y	N	18.00		27-Jul-21	29-Jul-21	30-Jul-21		21-Jan-22
C19IMPACT_1338	Trinidad and Tobago	23-Jul-21	MoH	N	N	N	N	Y	Y	17.00		27-Jul-21	29-Jul-21	30-Jul-21		
C19IMPACT_1339	Trinidad and Tobago	23-Jul-21	MoH	N	N	N	N	N	Y	17.00		27-Jul-21	29-Jul-21	30-Jul-21		21-Jan-22
C19IMPACT_1340	Trinidad and Tobago	23-Jul-21	MoH	Y	N	Y	N	N	N	14.00		27-Jul-21	29-Jul-21	30-Jul-21		21-Jan-22

C19IMPACT_1341	Trinidad and Tobago	23-Jul-21	MoH	N	N	Y	N	Y	N	17.00		27-Jul-21	29-Jul-21	30-Jul-21		21-Jan-22
C19IMPACT_1342	Trinidad and Tobago	23-Jul-21	MoH	N	N	N	N	Y	Y	18.00		27-Jul-21	29-Jul-21	30-Jul-21		
C19IMPACT_1343	Trinidad and Tobago	24-Jul-21	MoH	N	N	N	N	N	Y	18.90		27-Jul-21	29-Jul-21	30-Jul-21		
C19IMPACT_1344	Trinidad and Tobago	24-Jul-21	MoH	N	N	N	N	N	Y	16.60		27-Jul-21	29-Jul-21	30-Jul-21		21-Jan-22
C19IMPACT_1345	Trinidad and Tobago	24-Jul-21	MoH	N	N	N	N	N	Y	19.20		27-Jul-21	29-Jul-21	30-Jul-21		21-Jan-22
C19IMPACT_1346	Trinidad and Tobago	24-Jul-21	MoH	N	N	N	N	N	Y	13.20		27-Jul-21	29-Jul-21	30-Jul-21		21-Jan-22
C19IMPACT_1347	Trinidad and Tobago	24-Jul-21	MoH	N	N	N	N	N	N	33.00		27-Jul-21	29-Jul-21	13-Oct-21		
C19IMPACT_1348	Trinidad and Tobago	21-Jul-21	MoH	N	N	N	N	N	Y			29-Jul-21	31-Jul-21	06-Aug-21		
C19IMPACT_1349	Trinidad and Tobago	21-Jul-21	MoH	N	N	N	N	N	N			27-Jul-21	31-Jul-21	06-Aug-21		21-Jan-22
C19IMPACT_1350	Trinidad and Tobago	22-Jul-21	MoH	N	N	N	N	N	Y			29-Jul-21	31-Jul-21	06-Aug-21		21-Jan-22
C19IMPACT_1351	Trinidad and Tobago	23-Jul-21	MoH	N	N	N	N	N	Y			29-Jul-21	31-Jul-21	06-Aug-21		21-Jan-22
C19IMPACT_1352	Trinidad and Tobago	24-Jul-21	MoH	N	N	N	N	N	N			27-Jul-21	31-Jul-21	06-Aug-21		21-Jan-22
C19IMPACT_1353	Trinidad and Tobago	24-Jul-21	MoH	N	N	N	N	N	N	12.60		29-Jul-21	31-Jul-21	06-Aug-21		21-Jan-22
C19IMPACT_1354	Trinidad and Tobago	26-Jul-21	MoH	N	N	N	N	N	Y	17.00		29-Jul-21	31-Jul-21	06-Aug-21		
C19IMPACT_1355	Trinidad and Tobago	26-Jul-21	MoH	N	N	N	N	N	Y	24.00		29-Jul-21	31-Jul-21	06-Aug-21		
C19IMPACT_1356	Trinidad and Tobago	26-Jul-21	MoH	N	N	N	N	N	N	11.50		29-Jul-21	31-Jul-21	06-Aug-21		21-Jan-22
C19IMPACT_1357	Trinidad and Tobago	26-Jul-21	MoH	N	N	N	N	N	Y		12.00	29-Jul-21	31-Jul-21	06-Aug-21		21-Jan-22
C19IMPACT_1358	Trinidad and Tobago	26-Jul-21	MoH	N	N	N	N	N	Y		11.00	29-Jul-21	31-Jul-21	06-Aug-21		21-Jan-22
C19IMPACT_1359	Trinidad and Tobago	26-Jul-21	MoH	N	N	N	N	N	Y		5.00	29-Jul-21	31-Jul-21	06-Aug-21		21-Jan-22
C19IMPACT_1360	Trinidad and Tobago	26-Jul-21	MoH	N	N	N	N	N	Y		7.00	29-Jul-21	31-Jul-21	06-Aug-21		21-Jan-22
C19IMPACT_1361	Trinidad and Tobago	26-Jul-21	MoH	N	N	N	N	N	Y		6.00	29-Jul-21	31-Jul-21	06-Aug-21		21-Jan-22
C19IMPACT_1362	Trinidad and Tobago	26-Jul-21	MoH	N	N	N	N	N	Y		11.00	29-Jul-21	31-Jul-21	06-Aug-21		21-Jan-22
C19IMPACT_1363	Trinidad and Tobago	26-Jul-21	MoH	N	N	N	N	N	Y			29-Jul-21	31-Jul-21	06-Aug-21		21-Jan-22
C19IMPACT_1364	Trinidad and Tobago	26-Jul-21	MoH	N	N	N	N	N	Y			29-Jul-21	31-Jul-21	06-Aug-21		21-Jan-22
C19IMPACT_1365	Trinidad and Tobago	26-Jul-21	MoH	N	N	N	N	N	Y	27.00		29-Jul-21	31-Jul-21	06-Aug-21		21-Jan-22
C19IMPACT_1366	Trinidad and Tobago	26-Jul-21	MoH	N	N	N	N	N	Y		19.00	29-Jul-21	31-Jul-21	06-Aug-21		
C19IMPACT_1367	Trinidad and Tobago	27-Jul-21	MoH	N	N	N	N	N	N	15.20		29-Jul-21	31-Jul-21	06-Aug-21		21-Jan-22
C19IMPACT_1368	Trinidad and Tobago	27-Jul-21	MoH	N	N	N	N	N	N	13.10		29-Jul-21	31-Jul-21	06-Aug-21		21-Jan-22
C19IMPACT_1369	Trinidad and Tobago	27-Jul-21	MoH	N	N	N	N	N	N	11.10		29-Jul-21	31-Jul-21	06-Aug-21		21-Jan-22
C19IMPACT_1370	Grenada	14-Jul-21	CARPHA	N	N	N	N	N	N	16.20		29-Jul-21	04-Aug-21		05-Aug-21	29-Aug-21
C19IMPACT_1371	Antigua and Barbuda	18-Jul-21	CARPHA	N	N	N	N	N	N	16.16		29-Jul-21	04-Aug-21		05-Aug-21	23-Aug-21
C19IMPACT_1372	Antigua and Barbuda	21-Jul-21	CARPHA	N	N	N	N	N	N	20.07		29-Jul-21	04-Aug-21		05-Aug-21	23-Aug-21
C19IMPACT_1373	Antigua and Barbuda	21-Jul-21	CARPHA	N	N	N	N	N	N	10.87		29-Jul-21	04-Aug-21		05-Aug-21	23-Aug-21
C19IMPACT_1374	Saint Vincent and the Grenadines	21-Jul-21	CARPHA	N	N	N	N	N	N	20.42		29-Jul-21	04-Aug-21		05-Aug-21	23-Aug-21
C19IMPACT_1375	Antigua and Barbuda	22-Jul-21	CARPHA	N	N	N	N	N	N	15.72		29-Jul-21	04-Aug-21		05-Aug-21	29-Aug-21
C19IMPACT_1376	Antigua and Barbuda	22-Jul-21	CARPHA	N	N	N	N	N	N	17.18		29-Jul-21	04-Aug-21		05-Aug-21	23-Aug-21
C19IMPACT_1377	Antigua and Barbuda	22-Jul-21	CARPHA	N	N	N	N	N	N	21.59		29-Jul-21	04-Aug-21		05-Aug-21	29-Aug-21
C19IMPACT_1378	Antigua and Barbuda	22-Jul-21	CARPHA	N	N	N	N	N	N	26.95		29-Jul-21	04-Aug-21		05-Aug-21	29-Aug-21
C19IMPACT_1379	Antigua and Barbuda	22-Jul-21	CARPHA	N	N	N	N	N	N	21.61		29-Jul-21	04-Aug-21		05-Aug-21	23-Aug-21
C19IMPACT_1380	Trinidad and Tobago	26-Jul-21	MoH	N	N	N	N	N	Y		17.00	29-Jul-21	04-Aug-21	06-Aug-21		
C19IMPACT_1381	Trinidad and Tobago	26-Jul-21	MoH	N	N	N	N	N	Y		9.00	29-Jul-21	04-Aug-21	06-Aug-21		29-Aug-21
C19IMPACT_1382	Trinidad and Tobago	27-Jul-21	CARPHA	N	N	Y	N	N	N	15.90		29-Jul-21	04-Aug-21	06-Aug-21	29-Oct-21	29-Aug-21
C19IMPACT_1383	Trinidad and Tobago	28-Jul-21	MoH	N	N	N	N	Y	N	22.00		30-Jul-21	04-Aug-21	06-Aug-21		29-Aug-21
C19IMPACT_1384	Trinidad and Tobago	28-Jul-21	MoH	N	N	Y	N	N	N	27.00		30-Jul-21	04-Aug-21	06-Aug-21		29-Aug-21
C19IMPACT_1385	Trinidad and Tobago	28-Jul-21	MoH	N	N	N	N	Y	N	14.60		30-Jul-21	04-Aug-21	06-Aug-21		29-Aug-21
C19IMPACT_1386	Trinidad and Tobago	28-Jul-21	MoH	N	N	Y	N	N	N	18.00		30-Jul-21	04-Aug-21	06-Aug-21		29-Aug-21
C19IMPACT_1387	Trinidad and Tobago	29-Jul-21	MoH	N	N	N	N	Y	N	17.00		30-Jul-21	04-Aug-21	06-Aug-21		
C19IMPACT_1388	Trinidad and Tobago	29-Jul-21	MoH	N	N	N	N	N	N	25.00		30-Jul-21	04-Aug-21	06-Aug-21		
C19IMPACT_1389	Trinidad and Tobago	29-Jul-21	MoH	N	N	N	N	N	N	23.00		30-Jul-21	04-Aug-21	06-Aug-21		29-Aug-21
C19IMPACT_1390	Trinidad and Tobago	29-Jul-21	MoH	N	Y	Y	N	N	N	22.00		30-Jul-21	04-Aug-21	06-Aug-21		29-Aug-21
C19IMPACT_1391	Trinidad and Tobago	26-Jul-21	MoH	N	N	N	N	Y	Y		29.00	03-Aug-21	06-Aug-21	06-Aug-21		
C19IMPACT_1392	Trinidad and Tobago	26-Jul-21	MoH	N	N	N	N	N	Y		13.00	03-Aug-21	06-Aug-21	06-Aug-21		31-Aug-21
C19IMPACT_1393	Trinidad and Tobago	27-Jul-21	MoH	N	N	N	N	N	N			03-Aug-21	06-Aug-21	06-Aug-21		
C19IMPACT_1394	Trinidad and Tobago	27-Jul-21	MoH	N	N	N	N	N	N			03-Aug-21	06-Aug-21	06-Aug-21		31-Aug-21
C19IMPACT_1395	Trinidad and Tobago	27-Jul-21	MoH	N	N	N	N	N	Y		8.00	03-Aug-21	06-Aug-21	06-Aug-21		31-Aug-21
C19IMPACT_1396	Trinidad and Tobago	27-Jul-21	MoH	N	N	N	N	N	Y		11.00	03-Aug-21	06-Aug-21	06-Aug-21		31-Aug-21
C19IMPACT_1397	Trinidad and Tobago	28-Jul-21	MoH	N	N	N	N	N	Y	15.70		03-Aug-21	06-Aug-21	06-Aug-21		
C19IMPACT_1398	Trinidad and Tobago	28-Jul-21	MoH	N	N	N	N	N	Y	19.50		03-Aug-21	06-Aug-21	06-Aug-21		
C19IMPACT_1399	Trinidad and Tobago	28-Jul-21	MoH	N	N	N	N	N	Y	17.10		03-Aug-21	06-Aug-21	06-Aug-21		
C19IMPACT_1400	Trinidad and Tobago	28-Jul-21	MoH	N	N	N	N	N	Y	17.50		03-Aug-21	06-Aug-21	06-Aug-21		
C19IMPACT_1401	Trinidad and Tobago	28-Jul-21	MoH	N	N	N	N	N	Y	18.40		03-Aug-21	06-Aug-21	06-Aug-21		
C19IMPACT_1402	Trinidad and Tobago	28-Jul-21	MoH	N	N	N	N	N	Y		21.00	03-Aug-21	06-Aug-21	06-Aug-21		
C19IMPACT_1403	Trinidad and Tobago	28-Jul-21	MoH	N	N	N	N	N	Y	14.70		03-Aug-21	06-Aug-21	06-Aug-21		31-Aug-21
C19IMPACT_1404	Trinidad and Tobago	28-Jul-21	MoH	N	N	N	N	N	Y		6.00	03-Aug-21	06-Aug-21	06-Aug-21		31-Aug-21

C19IMPACT_1405	Trinidad and Tobago	28-Jul-21	MoH	N	N	N	N	N	Y		7.00	03-Aug-21	06-Aug-21	06-Aug-21		31-Aug-21
C19IMPACT_1406	Trinidad and Tobago	28-Jul-21	MoH	N	N	N	N	N	Y		10.00	03-Aug-21	06-Aug-21	06-Aug-21		31-Aug-21
C19IMPACT_1407	Trinidad and Tobago	29-Jul-21	MoH	N	N	N	N	N	N			03-Aug-21	06-Aug-21	06-Aug-21		31-Aug-21
C19IMPACT_1408	Trinidad and Tobago	29-Jul-21	MoH	N	N	N	N	N	Y		4.00	03-Aug-21	06-Aug-21	06-Aug-21		31-Aug-21
C19IMPACT_1409	Trinidad and Tobago	31-Jul-21	MoH	N	N	N	N	N	N			03-Aug-21	06-Aug-21	06-Aug-21		31-Aug-21
C19IMPACT_1410	Trinidad and Tobago	31-Jul-21	MoH	N	N	N	N	N	N			03-Aug-21	06-Aug-21	06-Aug-21		31-Aug-21
C19IMPACT_1411	Trinidad and Tobago	01-Aug-21	MoH	N	N	N	N	N	N			03-Aug-21	06-Aug-21	06-Aug-21		31-Aug-21
C19IMPACT_1412	Trinidad and Tobago		MoH	N	N	N	N	N	N			03-Aug-21	06-Aug-21	06-Aug-21		
C19IMPACT_1413	Barbados	19-Jul-21	CARPHA	N	N	N	N	N	N	26.70		04-Aug-21	09-Aug-21		12-Aug-21	
C19IMPACT_1414	Barbados	19-Jul-21	CARPHA	N	N	N	N	N	N	23.92		04-Aug-21	09-Aug-21		12-Aug-21	01-Sept-21
C19IMPACT_1415	Barbados	19-Jul-21	CARPHA	N	N	N	N	N	N	23.43		04-Aug-21	09-Aug-21		12-Aug-21	01-Sept-21
C19IMPACT_1416	Barbados	19-Jul-21	CARPHA	N	N	N	N	N	N	25.39		04-Aug-21	09-Aug-21		12-Aug-21	23-Aug-21
C19IMPACT_1417	Barbados	19-Jul-21	CARPHA	N	N	N	N	N	N	19.20		04-Aug-21	09-Aug-21		12-Aug-21	23-Aug-21
C19IMPACT_1418	Barbados	20-Jul-21	CARPHA	N	N	N	N	N	N	17.39		04-Aug-21	09-Aug-21		12-Aug-21	23-Aug-21
C19IMPACT_1419	Barbados	20-Jul-21	CARPHA	N	N	N	N	N	N	17.26		04-Aug-21	09-Aug-21		12-Aug-21	23-Aug-21
C19IMPACT_1420	Barbados	20-Jul-21	CARPHA	N	N	N	N	N	N	25.88		04-Aug-21	09-Aug-21		12-Aug-21	23-Aug-21
C19IMPACT_1421	Barbados	21-Jul-21	CARPHA	N	N	N	N	N	N	17.78		04-Aug-21	09-Aug-21		12-Aug-21	23-Aug-21
C19IMPACT_1422	Barbados	21-Jul-21	CARPHA	N	N	N	N	N	N	18.14		04-Aug-21	09-Aug-21		12-Aug-21	23-Aug-21
C19IMPACT_1423	Barbados	21-Jul-21	CARPHA	N	N	N	N	N	N	19.24		04-Aug-21	09-Aug-21		12-Aug-21	01-Sept-21
C19IMPACT_1424	Barbados	22-Jul-21	CARPHA	N	N	N	N	N	N	19.83		04-Aug-21	09-Aug-21		12-Aug-21	01-Sept-21
C19IMPACT_1425	Barbados	22-Jul-21	CARPHA	N	N	N	N	N	N	21.25		04-Aug-21	09-Aug-21		12-Aug-21	
C19IMPACT_1426	Barbados	22-Jul-21	CARPHA	N	N	N	N	N	N	20.42		04-Aug-21	09-Aug-21		12-Aug-21	01-Sept-21
C19IMPACT_1427	Barbados	22-Jul-21	CARPHA	N	N	N	N	N	N	17.27		04-Aug-21	09-Aug-21		12-Aug-21	23-Aug-21
C19IMPACT_1428	Barbados	22-Jul-21	CARPHA	N	N	N	N	N	N	19.48		04-Aug-21	09-Aug-21		12-Aug-21	01-Sept-21
C19IMPACT_1429	Barbados	23-Jul-21	CARPHA	N	N	N	N	N	N	25.87		04-Aug-21	09-Aug-21		12-Aug-21	23-Aug-21
C19IMPACT_1430	Barbados	23-Jul-21	CARPHA	N	N	N	N	N	N	19.70		04-Aug-21	09-Aug-21		12-Aug-21	01-Sept-21
C19IMPACT_1431	Barbados	23-Jul-21	CARPHA	N	N	N	N	N	N	20.65		04-Aug-21	09-Aug-21		12-Aug-21	01-Sept-21
C19IMPACT_1432	Barbados	25-Jul-21	CARPHA	N	N	N	N	N	N	22.67		04-Aug-21	09-Aug-21		12-Aug-21	01-Sept-21
C19IMPACT_1433	Barbados	25-Jul-21	CARPHA	N	N	N	N	N	N	23.08		04-Aug-21	09-Aug-21		12-Aug-21	
C19IMPACT_1434	Barbados	25-Jul-21	CARPHA	N	N	N	N	N	N	15.73		04-Aug-21	09-Aug-21		12-Aug-21	23-Aug-21
C19IMPACT_1435	Barbados	25-Jul-21	CARPHA	N	N	N	N	N	N	21.12		04-Aug-21	09-Aug-21		12-Aug-21	23-Aug-21
C19IMPACT_1436	Barbados	25-Jul-21	CARPHA	N	N	N	N	N	N	18.58		04-Aug-21	09-Aug-21		12-Aug-21	23-Aug-21
C19IMPACT_1437	Trinidad and Tobago	26-Jul-21	MoH	N	N	N	N	N	Y	18.00		03-Aug-21	09-Aug-21	06-Aug-21		
C19IMPACT_1438	Trinidad and Tobago	27-Jul-21	CARPHA	N	N	N	N	N	N	20.97		04-Aug-21	09-Aug-21	13-Aug-21	12-Aug-21	01-Sept-21
C19IMPACT_1439	Trinidad and Tobago	27-Jul-21	MoH	N	N	N	N	N	N			03-Aug-21	09-Aug-21	13-Aug-21		01-Sept-21
C19IMPACT_1440	Trinidad and Tobago	29-Jul-21	MoH	N	N	N	N	N	Y	11.00		03-Aug-21	09-Aug-21	13-Aug-21		01-Sept-21
C19IMPACT_1441	Trinidad and Tobago	29-Jul-21	MoH	N	N	N	N	N	Y	16.00		03-Aug-21	09-Aug-21	13-Aug-21		01-Sept-21
C19IMPACT_1442	Trinidad and Tobago	29-Jul-21	MoH	N	N	N	N	N	Y	20.00		03-Aug-21	09-Aug-21	13-Aug-21		01-Sept-21
C19IMPACT_1443	Trinidad and Tobago	30-Jul-21	MoH	N	N	N	N	N	Y	15.00		03-Aug-21	09-Aug-21	13-Aug-21		
C19IMPACT_1444	Trinidad and Tobago	30-Jul-21	MoH	N	N	N	N	N	Y	17.00		03-Aug-21	09-Aug-21	13-Aug-21		
C19IMPACT_1445	Trinidad and Tobago	30-Jul-21	MoH	N	N	N	N	N	Y	15.00		03-Aug-21	09-Aug-21	13-Aug-21		01-Sept-21
C19IMPACT_1446	Trinidad and Tobago	30-Jul-21	MoH	N	N	N	N	N	Y	18.00		03-Aug-21	09-Aug-21	13-Aug-21		01-Sept-21
C19IMPACT_1447	Trinidad and Tobago	30-Jul-21	MoH	N	N	N	N	N	Y	13.00		03-Aug-21	09-Aug-21	13-Aug-21		01-Sept-21
C19IMPACT_1448	Trinidad and Tobago	30-Jul-21	MoH	N	N	N	N	N	Y	12.00		03-Aug-21	09-Aug-21	13-Aug-21		01-Sept-21
C19IMPACT_1449	Trinidad and Tobago	30-Jul-21	MoH	N	N	N	N	N	Y	17.00		03-Aug-21	09-Aug-21	13-Aug-21		01-Sept-21
C19IMPACT_1450	Trinidad and Tobago	30-Jul-21	MoH	N	N	N	N	N	Y	17.00		03-Aug-21	09-Aug-21	13-Aug-21		01-Sept-21
C19IMPACT_1451	Trinidad and Tobago	30-Jul-21	MoH	N	N	N	N	N	N			04-Aug-21	09-Aug-21	13-Aug-21		
C19IMPACT_1452	Trinidad and Tobago	01-Aug-21	CARPHA	N	N	N	N	N	N	29.20		04-Aug-21	09-Aug-21	13-Aug-21	12-Aug-21	
C19IMPACT_1453	Trinidad and Tobago	02-Aug-21	MoH	N	N	N	N	N	N			04-Aug-21	09-Aug-21	13-Aug-21		01-Sept-21
C19IMPACT_1454	Trinidad and Tobago	02-Aug-21	MoH	N	N	N	N	N	N			04-Aug-21	09-Aug-21	13-Aug-21		01-Sept-21
C19IMPACT_1455	Trinidad and Tobago	02-Aug-21	MoH	N	N	N	N	N	N			04-Aug-21	09-Aug-21	13-Aug-21		01-Sept-21
C19IMPACT_1456	Trinidad and Tobago	02-Aug-21	MoH	N	N	N	N	N	Y			04-Aug-21	09-Aug-21	13-Aug-21		
C19IMPACT_1457	Saint Lucia	07-Jul-21	CARPHA	N	N	N	N	N	N	15.43		04-Aug-21	10-Aug-21		12-Aug-21	27-Aug-21
C19IMPACT_1458	Saint Lucia	09-Jul-21	CARPHA	N	N	N	N	N	N	15.23		04-Aug-21	10-Aug-21		12-Aug-21	27-Aug-21
C19IMPACT_1459	Barbados	12-Jul-21	CARPHA	N	N	N	N	N	N	24.55		04-Aug-21	10-Aug-21		12-Aug-21	27-Aug-21
C19IMPACT_1460	Barbados	13-Jul-21	CARPHA	N	N	N	N	N	N	21.09		04-Aug-21	10-Aug-21		12-Aug-21	27-Aug-21
C19IMPACT_1461	Barbados	13-Jul-21	CARPHA	N	N	N	N	N	N	26.03		04-Aug-21	10-Aug-21		12-Aug-21	27-Aug-21
C19IMPACT_1462	Barbados	14-Jul-21	CARPHA	N	N	N	N	N	N	18.55		04-Aug-21	10-Aug-21		12-Aug-21	23-Aug-21
C19IMPACT_1463	Saint Lucia	14-Jul-21	CARPHA	N	N	N	N	N	N	16.96		04-Aug-21	10-Aug-21		12-Aug-21	27-Aug-21
C19IMPACT_1464	Saint Lucia	14-Jul-21	CARPHA	N	N	N	N	N	N	15.07		04-Aug-21	10-Aug-21		12-Aug-21	23-Aug-21
C19IMPACT_1465	Barbados	19-Jul-21	CARPHA	N	N	N	N	N	N	22.83		04-Aug-21	10-Aug-21		12-Aug-21	
C19IMPACT_1466	British Virgin Islands	19-Jul-21	CARPHA	N	N	N	N	N	N	15.63		04-Aug-21	10-Aug-21		12-Aug-21	27-Aug-21
C19IMPACT_1467	Barbados	19-Jul-21	CARPHA	N	N	N	N	N	N	18.13		04-Aug-21	10-Aug-21		12-Aug-21	27-Aug-21
C19IMPACT_1468	Barbados	19-Jul-21	CARPHA	N	N	N	N	N	N	24.44		04-Aug-21	10-Aug-21		12-Aug-21	27-Aug-21

C19IMPACT_1469	Barbados	19-Jul-21	CARPHA	N	N	N	N	N	N	22.04		04-Aug-21	10-Aug-21		12-Aug-21	23-Aug-21
C19IMPACT_1470	Saint Lucia	19-Jul-21	CARPHA	N	N	N	N	N	N	14.62		04-Aug-21	10-Aug-21		12-Aug-21	27-Aug-21
C19IMPACT_1471	British Virgin Islands	19-Jul-21	CARPHA	N	N	N	N	N	N	19.95		04-Aug-21	10-Aug-21		12-Aug-21	27-Aug-21
C19IMPACT_1472	Barbados	20-Jul-21	CARPHA	N	N	N	N	N	N	19.27		04-Aug-21	10-Aug-21		12-Aug-21	23-Aug-21
C19IMPACT_1473	Antigua and Barbuda	24-Jul-21	CARPHA	N	N	N	N	N	N	21.82		04-Aug-21	10-Aug-21		12-Aug-21	23-Aug-21
C19IMPACT_1474	Antigua and Barbuda	24-Jul-21	CARPHA	N	N	N	N	N	N	17.20		04-Aug-21	10-Aug-21		12-Aug-21	23-Aug-21
C19IMPACT_1475	British Virgin Islands	25-Jul-21	CARPHA	N	N	N	N	N	N	21.03		04-Aug-21	10-Aug-21		12-Aug-21	27-Aug-21
C19IMPACT_1476	British Virgin Islands	25-Jul-21	CARPHA	N	N	N	N	N	N	17.05		04-Aug-21	10-Aug-21		12-Aug-21	27-Aug-21
C19IMPACT_1477	British Virgin Islands	25-Jul-21	CARPHA	N	N	N	N	N	N	24.51		04-Aug-21	10-Aug-21		12-Aug-21	27-Aug-21
C19IMPACT_1478	British Virgin Islands	25-Jul-21	CARPHA	N	N	N	N	N	N	18.49		04-Aug-21	10-Aug-21		12-Aug-21	27-Aug-21
C19IMPACT_1479	Saint Lucia	25-Jul-21	CARPHA	N	N	N	N	N	N	18.85		04-Aug-21	10-Aug-21		12-Aug-21	23-Aug-21
C19IMPACT_1480	Saint Lucia	25-Jul-21	CARPHA	N	N	N	N	N	N	17.22		04-Aug-21	10-Aug-21		12-Aug-21	23-Aug-21
C19IMPACT_1481	Antigua and Barbuda	25-Jul-21	CARPHA	N	N	N	N	N	N	18.54		04-Aug-21	10-Aug-21		12-Aug-21	27-Aug-21
C19IMPACT_1482	Antigua and Barbuda	25-Jul-21	CARPHA	N	N	N	N	N	Y	20.94		04-Aug-21	10-Aug-21		12-Aug-21	23-Aug-21
C19IMPACT_1483	Grenada	26-Jul-21	CARPHA	N	N	N	N	N	N	17.47		04-Aug-21	10-Aug-21		12-Aug-21	23-Aug-21
C19IMPACT_1484	Grenada	26-Jul-21	CARPHA	N	N	N	N	N	N	16.91		04-Aug-21	10-Aug-21		12-Aug-21	23-Aug-21
C19IMPACT_1485	Saint Lucia	26-Jul-21	CARPHA	N	N	N	N	N	N	14.76		04-Aug-21	10-Aug-21		12-Aug-21	23-Aug-21
C19IMPACT_1486	Saint Lucia	26-Jul-21	CARPHA	N	N	N	N	N	N	15.43		04-Aug-21	10-Aug-21		12-Aug-21	27-Aug-21
C19IMPACT_1487	Saint Lucia	26-Jul-21	CARPHA	N	N	N	N	N	N	14.87		04-Aug-21	10-Aug-21		12-Aug-21	27-Aug-21
C19IMPACT_1488	Grenada	26-Jul-21	CARPHA	N	N	N	N	N	N	20.11		04-Aug-21	10-Aug-21		12-Aug-21	23-Aug-21
C19IMPACT_1489	Antigua and Barbuda	26-Jul-21	CARPHA	N	N	N	N	N	N	20.78		04-Aug-21	10-Aug-21		12-Aug-21	23-Aug-21
C19IMPACT_1490	Antigua and Barbuda	27-Jul-21	CARPHA	N	N	N	N	N	N	20.20		04-Aug-21	10-Aug-21		12-Aug-21	23-Aug-21
C19IMPACT_1491	Antigua and Barbuda	27-Jul-21	CARPHA	N	N	N	N	N	N	17.30		04-Aug-21	10-Aug-21		12-Aug-21	23-Aug-21
C19IMPACT_1492	British Virgin Islands	27-Jul-21	CARPHA	N	N	N	N	N	N	29.29		04-Aug-21	10-Aug-21		12-Aug-21	23-Aug-21
C19IMPACT_1493	Antigua and Barbuda	28-Jul-21	CARPHA	N	N	N	N	N	N	18.58		04-Aug-21	10-Aug-21		12-Aug-21	
C19IMPACT_1494	Trinidad and Tobago	28-Jul-21	MoH	N	N	N	N	N	N	15.00		05-Aug-21	10-Aug-21	13-Aug-21		27-Aug-21
C19IMPACT_1495	Antigua and Barbuda	28-Jul-21	CARPHA	N	N	N	N	N	N	19.99		04-Aug-21	10-Aug-21		12-Aug-21	23-Aug-21
C19IMPACT_1496	Antigua and Barbuda	28-Jul-21	CARPHA	N	N	N	N	N	N	17.37		04-Aug-21	10-Aug-21		12-Aug-21	23-Aug-21
C19IMPACT_1497	British Virgin Islands	28-Jul-21	CARPHA	N	N	N	N	N	N	20.95		04-Aug-21	10-Aug-21		12-Aug-21	27-Aug-21
C19IMPACT_1498	Antigua and Barbuda	29-Jul-21	CARPHA	N	N	N	N	N	N	23.48		04-Aug-21	10-Aug-21		12-Aug-21	23-Aug-21
C19IMPACT_1499	Trinidad and Tobago	30-Jul-21	MoH	N	N	N	N	N	N	18.00		05-Aug-21	10-Aug-21	13-Aug-21		27-Aug-21
C19IMPACT_1500	Trinidad and Tobago	03-Aug-21	MoH	N	N	N	N	N	Y	20.00		05-Aug-21	10-Aug-21	13-Aug-21		23-Aug-21
C19IMPACT_1501	Saint Kitts and Nevis	25-Jul-21	CARPHA	N	N	N	N	N	Y	14.96		06-Aug-21	12-Aug-21		13-Aug-21	21-Jan-22
C19IMPACT_1502	Saint Kitts and Nevis	26-Jul-21	CARPHA	N	N	N	N	N	Y	11.47		06-Aug-21	12-Aug-21		13-Aug-21	21-Jan-22
C19IMPACT_1503	Trinidad and Tobago	30-Jul-21	MoH	Y	N	Y	N	N	Y	18.00		05-Aug-21	12-Aug-21	13-Aug-21		21-Jan-22
C19IMPACT_1504	Trinidad and Tobago	30-Jul-21	MoH	Y	N	Y	N	Y	Y	19.00		05-Aug-21	12-Aug-21	13-Aug-21		21-Jan-22
C19IMPACT_1505	Trinidad and Tobago	30-Jul-21	MoH	N	N	N	N	Y	N	16.00		05-Aug-21	12-Aug-21	13-Aug-21		21-Jan-22
C19IMPACT_1506	Trinidad and Tobago	30-Jul-21	MoH	N	N	N	N	N	N	16.00		05-Aug-21	12-Aug-21	13-Aug-21		21-Jan-22
C19IMPACT_1507	Trinidad and Tobago	30-Jul-21	MoH	N	N	N	N	N	N	17.00		05-Aug-21	12-Aug-21	13-Aug-21		21-Jan-22
C19IMPACT_1508	Trinidad and Tobago	31-Jul-21	MoH	Y	N	Y	N	N	Y	19.00		05-Aug-21	12-Aug-21	13-Aug-21		21-Jan-22
C19IMPACT_1509	Trinidad and Tobago	03-Aug-21	MoH	N	N	N	N	Y	Y	14.00		06-Aug-21	12-Aug-21	13-Aug-21		
C19IMPACT_1510	Trinidad and Tobago	03-Aug-21	MoH	N	N	N	N	N	Y	14.00		06-Aug-21	12-Aug-21	13-Aug-21		
C19IMPACT_1511	Trinidad and Tobago	03-Aug-21	MoH	N	N	N	N	N	Y	15.00		06-Aug-21	12-Aug-21	13-Aug-21		
C19IMPACT_1512	Trinidad and Tobago	03-Aug-21	MoH	N	N	N	N	N	Y	14.00		06-Aug-21	12-Aug-21	13-Aug-21		21-Jan-22
C19IMPACT_1513	Trinidad and Tobago	03-Aug-21	CARPHA	N	N	N	N	N	Y	26.42		06-Aug-21	12-Aug-21	13-Aug-21	13-Aug-21	
C19IMPACT_1514	Trinidad and Tobago	03-Aug-21	CARPHA	N	N	N	N	N	Y	17.55		06-Aug-21	12-Aug-21	13-Aug-21	13-Aug-21	21-Jan-22
C19IMPACT_1515	Trinidad and Tobago	03-Aug-21	CARPHA	N	N	Y	N	N	Y	22.31		06-Aug-21	12-Aug-21	13-Aug-21	13-Aug-21	21-Jan-22
C19IMPACT_1516	Trinidad and Tobago	03-Aug-21	CARPHA	Y	N	Y	N	N	Y	18.99		06-Aug-21	12-Aug-21	13-Aug-21	13-Aug-21	21-Jan-22
C19IMPACT_1517	Trinidad and Tobago	03-Aug-21	CARPHA	N	N	Y	N	Y	Y	19.64		06-Aug-21	12-Aug-21	13-Aug-21	13-Aug-21	21-Jan-22
C19IMPACT_1518	Trinidad and Tobago	03-Aug-21	CARPHA	N	N	N	N	N	Y	21.62		06-Aug-21	12-Aug-21	13-Aug-21	13-Aug-21	21-Jan-22
C19IMPACT_1519	Trinidad and Tobago	04-Aug-21	MoH	N	N	N	N	N	Y	16.00		06-Aug-21	12-Aug-21	13-Aug-21		
C19IMPACT_1520	Trinidad and Tobago	04-Aug-21	MoH	N	N	N	N	N	Y	19.00		06-Aug-21	12-Aug-21	13-Aug-21		21-Jan-22
C19IMPACT_1521	Trinidad and Tobago	04-Aug-21	MoH	N	N	N	N	N	Y	18.00		06-Aug-21	12-Aug-21	13-Aug-21		21-Jan-22
C19IMPACT_1522	Trinidad and Tobago	04-Aug-21	MoH	Y	N	Y	N	N	Y	19.00		06-Aug-21	12-Aug-21	13-Aug-21		21-Jan-22
C19IMPACT_1523	Trinidad and Tobago	04-Aug-21	MoH	N	N	N	N	Y	Y	17.00		06-Aug-21	12-Aug-21	13-Aug-21		21-Jan-22
C19IMPACT_1524	Trinidad and Tobago	04-Aug-21	MoH	Y	N	Y	N	N	Y	19.00		06-Aug-21	12-Aug-21	13-Aug-21		21-Jan-22
C19IMPACT_1525	Trinidad and Tobago	04-Aug-21	MoH	Y	N	Y	N	Y	Y	17.00		06-Aug-21	12-Aug-21	13-Aug-21		21-Jan-22
C19IMPACT_1526	Trinidad and Tobago	04-Aug-21	MoH	Y	N	Y	N	Y	Y	19.00		06-Aug-21	12-Aug-21	13-Aug-21		21-Jan-22
C19IMPACT_1527	Trinidad and Tobago	04-Aug-21	MoH	N	N	N	N	Y	Y	16.00		06-Aug-21	12-Aug-21	13-Aug-21		21-Jan-22
C19IMPACT_1528	Trinidad and Tobago	04-Aug-21	MoH	N	N	N	N	N	Y	15.00		06-Aug-21	12-Aug-21	13-Aug-21		
C19IMPACT_1529	Trinidad and Tobago	04-Aug-21	MoH	N	N	N	N	N	Y	18.00		06-Aug-21	12-Aug-21	13-Aug-21		21-Jan-22
C19IMPACT_1530	Trinidad and Tobago	04-Aug-21	MoH	N	N	N	N	N	Y	15.00		06-Aug-21	12-Aug-21	13-Aug-21		21-Jan-22
C19IMPACT_1531	Trinidad and Tobago	05-Aug-21	MoH	N	N	N	N	N	Y	19.00		06-Aug-21	12-Aug-21	13-Aug-21		21-Jan-22
C19IMPACT_1532	Trinidad and Tobago	05-Aug-21	MoH	N	N	N	N	N	Y	12.90		10-Aug-21	12-Aug-21	13-Aug-21		

C19IMPACT_1533	Trinidad and Tobago	05-Aug-21	MoH	N	N	N	N	N	Y	13.20		10-Aug-21	12-Aug-21	13-Aug-21		21-Jan-22
C19IMPACT_1534	Trinidad and Tobago	05-Aug-21	MoH	N	N	N	N	N	Y	13.30		10-Aug-21	12-Aug-21	13-Aug-21		21-Jan-22
C19IMPACT_1535	Trinidad and Tobago	05-Aug-21	MoH	N	N	N	N	N	Y	12.60		10-Aug-21	12-Aug-21	13-Aug-21		21-Jan-22
C19IMPACT_1536	Trinidad and Tobago	05-Aug-21	MoH	N	N	N	N	N	Y	15.80		10-Aug-21	12-Aug-21	13-Aug-21		21-Jan-22
C19IMPACT_1537	Trinidad and Tobago	05-Aug-21	MoH	N	N	N	N	N	Y	12.20		10-Aug-21	12-Aug-21	13-Aug-21		21-Jan-22
C19IMPACT_1538	Trinidad and Tobago	10-Aug-21	MoH	Y	N	Y	N	N	Y	18.00		11-Aug-21	12-Aug-21	13-Aug-21		21-Jan-22
C19IMPACT_1539	Trinidad and Tobago	10-Aug-21	MoH	Y	N	Y	N	Y	Y	17.00		11-Aug-21	12-Aug-21	13-Aug-21		21-Jan-22
C19IMPACT_1540	Trinidad and Tobago	10-Aug-21	MoH	N	N	N	N	Y	Y	21.00		11-Aug-21	12-Aug-21	13-Aug-21		21-Jan-22
C19IMPACT_1541	Trinidad and Tobago	10-Aug-21	MoH	N	N	N	N	N	Y	17.00		11-Aug-21	12-Aug-21	13-Aug-21		21-Jan-22
C19IMPACT_1542	Trinidad and Tobago	10-Aug-21	MoH	N	N	N	N	N	Y	20.00		11-Aug-21	12-Aug-21	13-Aug-21		21-Jan-22
C19IMPACT_1543	Saint Kitts and Nevis		CARPHA	N	N	N	N	N	Y	19.12		06-Aug-21	12-Aug-21		13-Aug-21	
C19IMPACT_1544	Saint Kitts and Nevis		CARPHA	N	N	N	N	N	Y	15.80		06-Aug-21	12-Aug-21		13-Aug-21	
C19IMPACT_1545	Trinidad and Tobago	05-May-21	MoH	N	N	N	N	N	Y			10-Aug-21	14-Aug-21	20-Aug-21		21-Jan-22
C19IMPACT_1546	Trinidad and Tobago	05-May-21	MoH	N	N	N	N	N	Y			10-Aug-21	14-Aug-21	20-Aug-21		21-Jan-22
C19IMPACT_1547	Jamaica	28-Jun-21	CARPHA	N	N	N	N	N	N	24.76		13-Aug-21	14-Aug-21		16-Aug-21	
C19IMPACT_1548	Jamaica	29-Jun-21	CARPHA	N	N	N	N	N	N	15.63		13-Aug-21	14-Aug-21		16-Aug-21	21-Jan-22
C19IMPACT_1549	Jamaica	09-Jul-21	CARPHA	N	N	N	N	N	N	18.77		13-Aug-21	14-Aug-21		16-Aug-21	21-Jan-22
C19IMPACT_1550	Jamaica	09-Jul-21	CARPHA	N	N	N	N	N	N	19.14		13-Aug-21	14-Aug-21		16-Aug-21	21-Jan-22
C19IMPACT_1551	Jamaica	10-Jul-21	CARPHA	N	N	N	N	N	N	15.54		13-Aug-21	14-Aug-21		16-Aug-21	21-Jan-22
C19IMPACT_1552	Jamaica	11-Jul-21	CARPHA	N	N	N	N	N	N	21.59		13-Aug-21	14-Aug-21		16-Aug-21	
C19IMPACT_1553	Jamaica	11-Jul-21	CARPHA	N	N	N	N	N	N	20.64		13-Aug-21	14-Aug-21		16-Aug-21	21-Jan-22
C19IMPACT_1554	Jamaica	11-Jul-21	CARPHA	N	N	N	N	N	N	20.14		13-Aug-21	14-Aug-21		16-Aug-21	21-Jan-22
C19IMPACT_1555	Jamaica	12-Jul-21	CARPHA	N	N	N	N	N	N	15.60		13-Aug-21	14-Aug-21		16-Aug-21	21-Jan-22
C19IMPACT_1556	Jamaica	12-Jul-21	CARPHA	N	N	N	N	N	N	16.54		13-Aug-21	14-Aug-21		16-Aug-21	21-Jan-22
C19IMPACT_1557	Jamaica	12-Jul-21	CARPHA	N	N	N	N	N	N	17.37		13-Aug-21	14-Aug-21		16-Aug-21	21-Jan-22
C19IMPACT_1558	Jamaica	13-Jul-21	CARPHA	N	N	N	N	N	N	24.06		13-Aug-21	14-Aug-21		16-Aug-21	
C19IMPACT_1559	Jamaica	13-Jul-21	CARPHA	N	N	N	N	N	N	15.19		13-Aug-21	14-Aug-21		16-Aug-21	21-Jan-22
C19IMPACT_1560	Jamaica	13-Jul-21	CARPHA	N	N	N	N	N	N	16.88		13-Aug-21	14-Aug-21		16-Aug-21	21-Jan-22
C19IMPACT_1561	Jamaica	14-Jul-21	CARPHA	N	N	N	N	N	N	24.47		13-Aug-21	14-Aug-21		16-Aug-21	
C19IMPACT_1562	Jamaica	14-Jul-21	CARPHA	N	N	N	N	N	N	27.05		13-Aug-21	14-Aug-21		16-Aug-21	
C19IMPACT_1563	Jamaica	14-Jul-21	CARPHA	N	N	N	N	N	N	23.52		13-Aug-21	14-Aug-21		16-Aug-21	21-Jan-22
C19IMPACT_1564	Jamaica	16-Jul-21	CARPHA	N	N	N	N	N	N	17.42		13-Aug-21	14-Aug-21		16-Aug-21	21-Jan-22
C19IMPACT_1565	Jamaica	16-Jul-21	CARPHA	N	N	N	N	N	N	26.59		13-Aug-21	14-Aug-21		16-Aug-21	21-Jan-22
C19IMPACT_1566	Jamaica	16-Jul-21	CARPHA	N	N	N	N	N	N	21.42		13-Aug-21	14-Aug-21		16-Aug-21	21-Jan-22
C19IMPACT_1567	Jamaica	16-Jul-21	CARPHA	N	N	N	N	N	N	16.94		13-Aug-21	14-Aug-21		16-Aug-21	21-Jan-22
C19IMPACT_1568	Jamaica	16-Jul-21	CARPHA	N	N	N	N	N	N	22.27		13-Aug-21	14-Aug-21		16-Aug-21	21-Jan-22
C19IMPACT_1569	Jamaica	16-Jul-21	CARPHA	N	N	N	N	N	N	22.94		13-Aug-21	14-Aug-21		16-Aug-21	21-Jan-22
C19IMPACT_1570	Jamaica	16-Jul-21	CARPHA	N	N	N	N	N	N	21.37		13-Aug-21	14-Aug-21		16-Aug-21	21-Jan-22
C19IMPACT_1571	Jamaica	17-Jul-21	CARPHA	N	N	N	N	N	N	21.96		13-Aug-21	14-Aug-21		16-Aug-21	21-Jan-22
C19IMPACT_1572	Jamaica	18-Jul-21	CARPHA	N	N	N	N	N	N	25.71		13-Aug-21	14-Aug-21		16-Aug-21	
C19IMPACT_1573	Jamaica	19-Jul-21	CARPHA	N	N	N	N	N	N	17.99		13-Aug-21	14-Aug-21		16-Aug-21	21-Jan-22
C19IMPACT_1574	Jamaica	19-Jul-21	CARPHA	N	N	N	N	N	N	16.72		13-Aug-21	14-Aug-21		16-Aug-21	21-Jan-22
C19IMPACT_1575	Jamaica	20-Jul-21	CARPHA	N	N	N	N	N	N	24.99		13-Aug-21	14-Aug-21		16-Aug-21	
C19IMPACT_1576	Jamaica	20-Jul-21	CARPHA	N	N	N	N	N	N	19.75		13-Aug-21	14-Aug-21		16-Aug-21	21-Jan-22
C19IMPACT_1577	Jamaica	20-Jul-21	CARPHA	N	N	N	N	N	N	19.78		13-Aug-21	14-Aug-21		16-Aug-21	21-Jan-22
C19IMPACT_1578	Jamaica	21-Jul-21	CARPHA	N	N	N	N	N	N	18.94		13-Aug-21	14-Aug-21		16-Aug-21	21-Jan-22
C19IMPACT_1579	Jamaica	22-Jul-21	CARPHA	N	N	N	N	N	N	17.61		13-Aug-21	14-Aug-21		16-Aug-21	21-Jan-22
C19IMPACT_1580	Jamaica	22-Jul-21	CARPHA	N	N	N	N	N	N	22.04		13-Aug-21	14-Aug-21		16-Aug-21	
C19IMPACT_1581	Jamaica	22-Jul-21	CARPHA	N	N	N	N	N	N	15.15		13-Aug-21	14-Aug-21		16-Aug-21	21-Jan-22
C19IMPACT_1582	Jamaica	23-Jul-21	CARPHA	N	N	N	N	N	N	26.39		13-Aug-21	14-Aug-21		16-Aug-21	
C19IMPACT_1583	Jamaica	23-Jul-21	CARPHA	N	N	N	N	N	N	24.49		13-Aug-21	14-Aug-21		16-Aug-21	21-Jan-22
C19IMPACT_1584	Jamaica	23-Jul-21	CARPHA	N	N	N	N	N	N	23.94		13-Aug-21	14-Aug-21		16-Aug-21	21-Jan-22
C19IMPACT_1585	Jamaica	23-Jul-21	CARPHA	N	N	N	N	N	N	21.33		13-Aug-21	14-Aug-21		16-Aug-21	21-Jan-22
C19IMPACT_1586	Jamaica	24-Jul-21	CARPHA	N	N	N	N	N	N	27.15		13-Aug-21	14-Aug-21		16-Aug-21	21-Jan-22
C19IMPACT_1587	Trinidad and Tobago	03-Aug-21	CARPHA	Y	Y	Y	N	N	Y	20.37		10-Aug-21	14-Aug-21	20-Aug-21	16-Aug-21	21-Jan-22
C19IMPACT_1588	Trinidad and Tobago	03-Aug-21	MoH	N	N	N	N	Y	Y			10-Aug-21	14-Aug-21	20-Aug-21		21-Jan-22
C19IMPACT_1589	Trinidad and Tobago	04-Aug-21	CARPHA	N	N	N	N	N	Y	24.37		10-Aug-21	14-Aug-21	20-Aug-21	16-Aug-21	21-Jan-22
C19IMPACT_1590	Trinidad and Tobago	04-Aug-21	MoH	N	N	N	N	N	Y			10-Aug-21	14-Aug-21	20-Aug-21		
C19IMPACT_1591	Trinidad and Tobago	05-Aug-21	MoH	N	N	N	N	N	Y	13.20		10-Aug-21	14-Aug-21	20-Aug-21		
C19IMPACT_1592	Trinidad and Tobago	05-Aug-21	MoH	N	N	N	N	N	Y	19.20		10-Aug-21	14-Aug-21	20-Aug-21		
C19IMPACT_1593	Trinidad and Tobago	05-Aug-21	MoH	N	N	N	N	N	Y	17.10		10-Aug-21	14-Aug-21	20-Aug-21		
C19IMPACT_1594	Trinidad and Tobago	05-Aug-21	MoH	N	N	N	N	N	Y	15.00		10-Aug-21	14-Aug-21	20-Aug-21		21-Jan-22
C19IMPACT_1595	Trinidad and Tobago	05-Aug-21	MoH	N	N	N	N	N	Y	19.00		10-Aug-21	14-Aug-21	20-Aug-21		21-Jan-22
C19IMPACT_1596	Trinidad and Tobago	06-Aug-21	MoH	N	Y	N	N	N	Y		4.12	11-Aug-21	14-Aug-21	20-Aug-21		21-Jan-22

C19IMPACT_1597	Trinidad and Tobago	06-Aug-21	MoH	N	N	N	N	N	Y		19.00	10-Aug-21	14-Aug-21	20-Aug-21		
C19IMPACT_1598	Trinidad and Tobago	06-Aug-21	MoH	N	N	N	N	N	Y		6.00	10-Aug-21	14-Aug-21	20-Aug-21		
C19IMPACT_1599	Trinidad and Tobago	06-Aug-21	MoH	N	N	N	N	N	Y		12.00	10-Aug-21	14-Aug-21	20-Aug-21		
C19IMPACT_1600	Trinidad and Tobago	06-Aug-21	MoH	N	N	N	N	N	Y		4.00	10-Aug-21	14-Aug-21	20-Aug-21		21-Jan-22
C19IMPACT_1601	Trinidad and Tobago	06-Aug-21	MoH	N	N	N	N	N	Y		8.00	10-Aug-21	14-Aug-21	20-Aug-21		21-Jan-22
C19IMPACT_1602	Trinidad and Tobago	06-Aug-21	MoH	N	N	N	N	N	Y		6.00	10-Aug-21	14-Aug-21	20-Aug-21		21-Jan-22
C19IMPACT_1603	Trinidad and Tobago	06-Aug-21	MoH	N	N	N	N	N	Y		4.00	10-Aug-21	14-Aug-21	20-Aug-21		21-Jan-22
C19IMPACT_1604	Trinidad and Tobago	06-Aug-21	MoH	N	N	N	N	N	Y		9.00	10-Aug-21	14-Aug-21	20-Aug-21		21-Jan-22
C19IMPACT_1605	Trinidad and Tobago	06-Aug-21	MoH	N	N	N	N	N	Y		3.00	10-Aug-21	14-Aug-21	20-Aug-21		21-Jan-22
C19IMPACT_1606	Trinidad and Tobago	07-Aug-21	MoH	N	Y	N	N	N	Y		5.50	11-Aug-21	14-Aug-21	20-Aug-21		21-Jan-22
C19IMPACT_1607	Trinidad and Tobago	07-Aug-21	MoH	N	N	N	N	N	Y		15.30	11-Aug-21	14-Aug-21	20-Aug-21		
C19IMPACT_1608	Trinidad and Tobago	08-Aug-21	MoH	N	N	N	N	N	N	19.00		11-Aug-21	14-Aug-21	20-Aug-21		21-Jan-22
C19IMPACT_1609	Trinidad and Tobago	08-Aug-21	MoH	N	N	N	N	N	N	14.00		11-Aug-21	14-Aug-21	20-Aug-21		21-Jan-22
C19IMPACT_1610	Trinidad and Tobago	08-Aug-21	MoH	N	N	N	N	N	N	18.00		11-Aug-21	14-Aug-21	20-Aug-21		21-Jan-22
C19IMPACT_1611	Trinidad and Tobago	08-Aug-21	MoH	N	N	N	N	N	N	18.00		11-Aug-21	14-Aug-21	20-Aug-21		
C19IMPACT_1612	Trinidad and Tobago	08-Aug-21	MoH	Y	N	Y	N	N	N	19.00		11-Aug-21	14-Aug-21	20-Aug-21		21-Jan-22
C19IMPACT_1613	Trinidad and Tobago	08-Aug-21	MoH	Y	N	Y	N	Y	N	19.00		11-Aug-21	14-Aug-21	20-Aug-21		21-Jan-22
C19IMPACT_1614	Trinidad and Tobago	08-Aug-21	MoH	N	N	Y	N	Y	N	18.00		11-Aug-21	14-Aug-21	20-Aug-21		21-Jan-22
C19IMPACT_1615	Trinidad and Tobago	08-Aug-21	MoH	N	N	N	N	Y	N	19.00		11-Aug-21	14-Aug-21	20-Aug-21		21-Jan-22
C19IMPACT_1616	Trinidad and Tobago	08-Aug-21	MoH	N	N	N	N	N	N	18.00		11-Aug-21	14-Aug-21	20-Aug-21		21-Jan-22
C19IMPACT_1617	Trinidad and Tobago	08-Aug-21	MoH	N	N	N	N	N	N	19.00		11-Aug-21	14-Aug-21	20-Aug-21		21-Jan-22
C19IMPACT_1618	Trinidad and Tobago	08-Aug-21	MoH	N	N	N	N	N	N	19.00		11-Aug-21	14-Aug-21	20-Aug-21		21-Jan-22
C19IMPACT_1619	Trinidad and Tobago	08-Aug-21	MoH	N	N	N	N	N	N	17.00		11-Aug-21	14-Aug-21	20-Aug-21		21-Jan-22
C19IMPACT_1620	Trinidad and Tobago	08-Aug-21	MoH	N	N	N	N	N	N	18.00		11-Aug-21	14-Aug-21	20-Aug-21		
C19IMPACT_1621	Trinidad and Tobago	09-Aug-21	CARPHA	N	N	N	N	N	N	15.63		10-Aug-21	14-Aug-21	26-Nov-21	26-Nov-21	21-Jan-22
C19IMPACT_1622	Trinidad and Tobago	10-Aug-21	MoH	N	N	N	N	N	N	18.00		11-Aug-21	14-Aug-21	20-Aug-21		21-Jan-22
C19IMPACT_1623	Trinidad and Tobago	10-Aug-21	MoH	Y	N	Y	N	N	N	18.00		11-Aug-21	14-Aug-21	26-Nov-21		21-Jan-22
C19IMPACT_1624	Trinidad and Tobago	10-Aug-21	MoH	Y	N	Y	N	Y	N	16.00		11-Aug-21	14-Aug-21	26-Nov-21		21-Jan-22
C19IMPACT_1625	Trinidad and Tobago	10-Aug-21	MoH	N	N	N	N	Y	N	19.00		11-Aug-21	14-Aug-21	20-Aug-21		21-Jan-22
C19IMPACT_1626	Trinidad and Tobago	10-Aug-21	MoH	N	N	N	N	N	N	19.00		11-Aug-21	14-Aug-21	20-Aug-21		21-Jan-22
C19IMPACT_1627	Trinidad and Tobago	10-Aug-21	MoH	N	N	N	N	N	N	20.00		11-Aug-21	14-Aug-21	20-Aug-21		21-Jan-22
C19IMPACT_1628	Trinidad and Tobago	11-Aug-21	MoH	N	N	N	N	N	Y	31.00		13-Aug-21	14-Aug-21	20-Aug-21		
C19IMPACT_1629	Trinidad and Tobago	11-Aug-21	MoH	N	N	N	N	N	Y	33.00		13-Aug-21	14-Aug-21	20-Aug-21		
C19IMPACT_1630	Trinidad and Tobago	11-Aug-21	MoH	N	N	N	N	N	Y	18.00		13-Aug-21	14-Aug-21	20-Aug-21		21-Jan-22
C19IMPACT_1631	Jamaica	19-Jul-21	CARPHA	N	N	N	N	N	N	24.77		13-Aug-21	19-Aug-21		23-Aug-21	08-Sept-21
C19IMPACT_1632	Jamaica	21-Jul-21	CARPHA	N	N	N	N	N	N	14.22		13-Aug-21	19-Aug-21		23-Aug-21	08-Sept-21
C19IMPACT_1633	Jamaica	21-Jul-21	CARPHA	N	N	N	N	N	N	25.97		13-Aug-21	19-Aug-21		23-Aug-21	
C19IMPACT_1634	Jamaica	21-Jul-21	CARPHA	N	N	N	N	N	N	18.22		13-Aug-21	19-Aug-21		23-Aug-21	08-Sept-21
C19IMPACT_1635	Jamaica	21-Jul-21	CARPHA	N	N	N	N	N	N	18.30		13-Aug-21	19-Aug-21		23-Aug-21	08-Sept-21
C19IMPACT_1636	Jamaica	21-Jul-21	CARPHA	N	N	N	N	N	N	20.28		13-Aug-21	19-Aug-21		23-Aug-21	08-Sept-21
C19IMPACT_1637	Jamaica	22-Jul-21	CARPHA	N	N	N	N	N	N	24.76		13-Aug-21	19-Aug-21		23-Aug-21	
C19IMPACT_1638	Jamaica	22-Jul-21	CARPHA	N	N	N	N	N	N	17.86		13-Aug-21	19-Aug-21		23-Aug-21	08-Sept-21
C19IMPACT_1639	Jamaica	22-Jul-21	CARPHA	N	N	N	N	N	N	17.96		13-Aug-21	19-Aug-21		23-Aug-21	08-Sept-21
C19IMPACT_1640	Jamaica	22-Jul-21	CARPHA	N	N	N	N	N	N	21.89		13-Aug-21	19-Aug-21		23-Aug-21	08-Sept-21
C19IMPACT_1641	Jamaica	22-Jul-21	CARPHA	N	N	N	N	N	N	16.12		13-Aug-21	19-Aug-21		23-Aug-21	08-Sept-21
C19IMPACT_1642	Jamaica	22-Jul-21	CARPHA	N	N	N	N	N	N	16.71		13-Aug-21	19-Aug-21		23-Aug-21	08-Sept-21
C19IMPACT_1643	Jamaica	22-Jul-21	CARPHA	N	N	N	N	N	N	20.12		13-Aug-21	19-Aug-21		23-Aug-21	08-Sept-21
C19IMPACT_1644	Jamaica	23-Jul-21	CARPHA	N	N	N	N	N	N	24.78		13-Aug-21	19-Aug-21		23-Aug-21	
C19IMPACT_1645	Jamaica	23-Jul-21	CARPHA	N	N	N	N	N	N	25.95		13-Aug-21	19-Aug-21		23-Aug-21	
C19IMPACT_1646	Jamaica	23-Jul-21	CARPHA	N	N	N	N	N	N	23.72		13-Aug-21	19-Aug-21		23-Aug-21	08-Sept-21
C19IMPACT_1647	Jamaica	23-Jul-21	CARPHA	N	N	N	N	N	N	22.44		13-Aug-21	19-Aug-21		23-Aug-21	08-Sept-21
C19IMPACT_1648	Antigua and Barbuda	30-Jul-21	CARPHA	N	N	N	N	N	N	20.10		13-Aug-21	19-Aug-21		23-Aug-21	08-Sept-21
C19IMPACT_1649	Antigua and Barbuda	30-Jul-21	CARPHA	N	N	N	N	N	N	19.74		13-Aug-21	19-Aug-21		23-Aug-21	08-Sept-21
C19IMPACT_1650	Antigua and Barbuda	31-Jul-21	CARPHA	N	N	N	N	N	N	15.48		13-Aug-21	19-Aug-21		23-Aug-21	08-Sept-21
C19IMPACT_1651	Montserrat	01-Aug-21	CARPHA	N	N	N	N	N	N	15.63		13-Aug-21	19-Aug-21		23-Aug-21	08-Sept-21
C19IMPACT_1652	Anguilla	01-Aug-21	CARPHA	N	N	N	N	N	N	16.49		13-Aug-21	19-Aug-21		23-Aug-21	08-Sept-21
C19IMPACT_1653	Antigua and Barbuda	01-Aug-21	CARPHA	N	N	N	N	N	N	14.50		13-Aug-21	19-Aug-21		23-Aug-21	08-Sept-21
C19IMPACT_1654	Antigua and Barbuda	02-Aug-21	CARPHA	N	N	N	N	N	N	15.52		13-Aug-21	19-Aug-21		23-Aug-21	09-Sept-21
C19IMPACT_1655	Antigua and Barbuda	04-Aug-21	CARPHA	N	N	N	N	N	N	26.83		13-Aug-21	19-Aug-21		23-Aug-21	
C19IMPACT_1656	Antigua and Barbuda	04-Aug-21	CARPHA	N	N	N	N	N	N	16.85		13-Aug-21	19-Aug-21		23-Aug-21	
C19IMPACT_1657	Antigua and Barbuda	04-Aug-21	CARPHA	N	N	N	N	N	N	23.34		13-Aug-21	19-Aug-21		23-Aug-21	
C19IMPACT_1658	Antigua and Barbuda	04-Aug-21	CARPHA	N	N	N	N	N	N	19.74		13-Aug-21	19-Aug-21		23-Aug-21	08-Sept-21
C19IMPACT_1659	Antigua and Barbuda	04-Aug-21	CARPHA	N	N	N	N	N	N	19.12		13-Aug-21	19-Aug-21		23-Aug-21	08-Sept-21
C19IMPACT_1660	Antigua and Barbuda	04-Aug-21	CARPHA	N	N	N	N	N	N	19.03		13-Aug-21	19-Aug-21		23-Aug-21	08-Sept-21

C19IMPACT_1661	Anguilla	04-Aug-21	CARPHA	N	N	N	N	N	N	16.67		13-Aug-21	19-Aug-21		23-Aug-21	08-Sept-21
C19IMPACT_1662	Trinidad and Tobago	04-Aug-21	MoH	N	N	N	N	N	N	19.00		13-Aug-21	19-Aug-21	27-Aug-21		09-Sept-21
C19IMPACT_1663	Antigua and Barbuda	05-Aug-21	CARPHA	N	N	N	N	N	N	19.37		13-Aug-21	19-Aug-21		23-Aug-21	08-Sept-21
C19IMPACT_1664	Antigua and Barbuda	05-Aug-21	CARPHA	N	N	N	N	N	N	20.77		13-Aug-21	19-Aug-21		23-Aug-21	08-Sept-21
C19IMPACT_1665	Antigua and Barbuda	05-Aug-21	CARPHA	N	N	N	N	N	N	19.49		13-Aug-21	19-Aug-21		23-Aug-21	08-Sept-21
C19IMPACT_1666	Antigua and Barbuda	05-Aug-21	CARPHA	N	N	N	N	N	N	19.36		13-Aug-21	19-Aug-21		23-Aug-21	08-Sept-21
C19IMPACT_1667	Antigua and Barbuda	05-Aug-21	CARPHA	N	N	N	N	N	N	20.50		13-Aug-21	19-Aug-21		23-Aug-21	
C19IMPACT_1668	Trinidad and Tobago	05-Aug-21	MoH	N	N	N	N	N	N			13-Aug-21	19-Aug-21	27-Aug-21		09-Sept-21
C19IMPACT_1669	Antigua and Barbuda	06-Aug-21	CARPHA	N	N	N	N	N	N	18.66		13-Aug-21	19-Aug-21		23-Aug-21	08-Sept-21
C19IMPACT_1670	Antigua and Barbuda	06-Aug-21	CARPHA	N	N	N	N	N	N	17.69		13-Aug-21	19-Aug-21		23-Aug-21	08-Sept-21
C19IMPACT_1671	Antigua and Barbuda	06-Aug-21	CARPHA	N	N	N	N	N	N	18.90		13-Aug-21	19-Aug-21		23-Aug-21	08-Sept-21
C19IMPACT_1672	Trinidad and Tobago	06-Aug-21	MoH	N	N	N	N	N	Y		11.00	16-Aug-21	19-Aug-21	27-Aug-21		09-Sept-21
C19IMPACT_1673	Antigua and Barbuda	06-Aug-21	CARPHA	N	N	N	N	N	N	17.25		13-Aug-21	19-Aug-21		23-Aug-21	09-Sept-21
C19IMPACT_1674	Antigua and Barbuda	06-Aug-21	CARPHA	N	N	N	N	N	N	24.05		13-Aug-21	19-Aug-21		23-Aug-21	09-Sept-21
C19IMPACT_1675	Trinidad and Tobago	06-Aug-21	MoH	N	N	N	N	N	Y			13-Aug-21	19-Aug-21	29-Oct-21		21-Jan-22
C19IMPACT_1676	Trinidad and Tobago	06-Aug-21	MoH	N	N	N	N	N	N			13-Aug-21	19-Aug-21	29-Oct-21		21-Jan-22
C19IMPACT_1677	Trinidad and Tobago	07-Aug-21	CARPHA	N	N	Y	N	N	N	14.71		13-Aug-21	19-Aug-21	27-Aug-21	23-Aug-21	08-Sept-21
C19IMPACT_1678	Trinidad and Tobago	07-Aug-21	MoH	N	N	N	N	N	Y		4.00	16-Aug-21	19-Aug-21	27-Aug-21		09-Sept-21
C19IMPACT_1679	Trinidad and Tobago	07-Aug-21	CARPHA	N	N	N	N	N	N	14.84		13-Aug-21	19-Aug-21	27-Aug-21	23-Aug-21	09-Sept-21
C19IMPACT_1680	Saint Vincent and the Grenadines	08-Aug-21	CARPHA	N	N	N	N	N	N	15.90		13-Aug-21	19-Aug-21		23-Aug-21	08-Sept-21
C19IMPACT_1681	Trinidad and Tobago	08-Aug-21	MoH	N	N	N	N	N	Y	20.70		13-Aug-21	19-Aug-21	27-Aug-21		
C19IMPACT_1682	Trinidad and Tobago	08-Aug-21	MoH	N	N	N	N	N	Y	15.60		13-Aug-21	19-Aug-21	27-Aug-21		09-Sept-21
C19IMPACT_1683	Trinidad and Tobago	08-Aug-21	MoH	N	N	N	N	N	N			13-Aug-21	19-Aug-21	27-Aug-21		
C19IMPACT_1684	Trinidad and Tobago	08-Aug-21	MoH	N	N	N	N	N	N			13-Aug-21	19-Aug-21	27-Aug-21		09-Sept-21
C19IMPACT_1685	Trinidad and Tobago	08-Aug-21	MoH	N	N	N	N	N	Y		5.70	13-Aug-21	19-Aug-21	27-Aug-21		09-Sept-21
C19IMPACT_1686	Trinidad and Tobago	08-Aug-21	MoH	N	N	N	N	N	N			13-Aug-21	19-Aug-21	27-Aug-21		09-Sept-21
C19IMPACT_1687	Trinidad and Tobago	08-Aug-21	MoH	N	N	N	N	N	Y		3.29	13-Aug-21	19-Aug-21	27-Aug-21		09-Sept-21
C19IMPACT_1688	Trinidad and Tobago	08-Aug-21	MoH	N	N	N	N	N	Y	20.00		13-Aug-21	19-Aug-21	27-Aug-21		
C19IMPACT_1689	Saint Vincent and the Grenadines	09-Aug-21	CARPHA	N	N	N	N	N	N	15.00		13-Aug-21	19-Aug-21		23-Aug-21	08-Sept-21
C19IMPACT_1690	Anguilla	09-Aug-21	CARPHA	N	N	N	N	N	N	22.42		13-Aug-21	19-Aug-21		23-Aug-21	
C19IMPACT_1691	Anguilla	09-Aug-21	CARPHA	N	N	N	N	N	N	17.30		13-Aug-21	19-Aug-21		23-Aug-21	
C19IMPACT_1692	Anguilla	09-Aug-21	CARPHA	N	N	N	N	N	N	15.86		13-Aug-21	19-Aug-21		23-Aug-21	08-Sept-21
C19IMPACT_1693	Trinidad and Tobago	09-Aug-21	CARPHA	N	N	N	N	N	N	19.92		13-Aug-21	19-Aug-21	27-Aug-21	23-Aug-21	09-Sept-21
C19IMPACT_1694	Trinidad and Tobago	09-Aug-21	CARPHA	N	N	N	N	N	N	14.22		13-Aug-21	19-Aug-21	27-Aug-21	23-Aug-21	09-Sept-21
C19IMPACT_1695	Trinidad and Tobago	09-Aug-21	CARPHA	N	N	N	N	N	N	16.39		13-Aug-21	19-Aug-21	27-Aug-21	23-Aug-21	09-Sept-21
C19IMPACT_1696	Trinidad and Tobago	09-Aug-21	CARPHA	N	N	Y	N	N	N	16.12		13-Aug-21	19-Aug-21	27-Aug-21	23-Aug-21	09-Sept-21
C19IMPACT_1697	Trinidad and Tobago	09-Aug-21	MoH	N	N	N	N	Y	Y		13.10	13-Aug-21	19-Aug-21	27-Aug-21		09-Sept-21
C19IMPACT_1698	Trinidad and Tobago	09-Aug-21	MoH	N	Y	Y	N	N	N	18.00		13-Aug-21	19-Aug-21	27-Aug-21		09-Sept-21
C19IMPACT_1699	Trinidad and Tobago	09-Aug-21	MoH	N	N	N	N	Y	N			13-Aug-21	19-Aug-21	27-Aug-21		09-Sept-21
C19IMPACT_1700	Trinidad and Tobago	09-Aug-21	MoH	N	N	N	N	N	Y		4.73	13-Aug-21	19-Aug-21	27-Aug-21		09-Sept-21
C19IMPACT_1701	Trinidad and Tobago	10-Aug-21	MoH	N	N	N	N	N	Y	17.00		13-Aug-21	19-Aug-21	27-Aug-21		09-Sept-21
C19IMPACT_1702	Trinidad and Tobago	10-Aug-21	MoH	Y	N	Y	N	N	N	17.00		11-Aug-21	19-Aug-21	27-Aug-21		08-Sept-21
C19IMPACT_1703	Trinidad and Tobago	10-Aug-21	MoH	N	N	N	N	Y	Y	15.00		13-Aug-21	19-Aug-21	27-Aug-21		09-Sept-21
C19IMPACT_1704	Trinidad and Tobago	10-Aug-21	MoH	N	N	N	N	N	Y		5.41	13-Aug-21	19-Aug-21	27-Aug-21		09-Sept-21
C19IMPACT_1705	Trinidad and Tobago	10-Aug-21	MoH	N	N	N	N	N	N	19.00		13-Aug-21	19-Aug-21	27-Aug-21		09-Sept-21
C19IMPACT_1706	Trinidad and Tobago	10-Aug-21	MoH	N	N	N	N	N	Y	15.00		13-Aug-21	19-Aug-21	27-Aug-21		09-Sept-21
C19IMPACT_1707	Trinidad and Tobago	11-Aug-21	MoH	N	N	N	N	N	Y	19.60		16-Aug-21	19-Aug-21	27-Aug-21		
C19IMPACT_1708	Trinidad and Tobago	11-Aug-21	MoH	N	N	N	N	N	Y	17.70		16-Aug-21	19-Aug-21	27-Aug-21		
C19IMPACT_1709	Trinidad and Tobago	11-Aug-21	MoH	N	N	N	N	N	Y	15.40		16-Aug-21	19-Aug-21	27-Aug-21		
C19IMPACT_1710	Trinidad and Tobago	11-Aug-21	MoH	N	N	N	N	N	Y	18.90		16-Aug-21	19-Aug-21	27-Aug-21		09-Sept-21
C19IMPACT_1711	Trinidad and Tobago	11-Aug-21	MoH	N	N	Y	N	N	N	17.03		13-Aug-21	19-Aug-21	27-Aug-21		09-Sept-21
C19IMPACT_1712	Trinidad and Tobago	11-Aug-21	MoH	Y	Y	Y	N	Y	N	19.67		13-Aug-21	19-Aug-21	27-Aug-21		09-Sept-21
C19IMPACT_1713	Trinidad and Tobago	11-Aug-21	MoH	Y	N	Y	N	Y	N	17.00		13-Aug-21	19-Aug-21	27-Aug-21		09-Sept-21
C19IMPACT_1714	Trinidad and Tobago	11-Aug-21	MoH	Y	Y	Y	N	Y	N	18.88		13-Aug-21	19-Aug-21	27-Aug-21		09-Sept-21
C19IMPACT_1715	Trinidad and Tobago	11-Aug-21	MoH	N	N	N	N	Y	N	19.47		13-Aug-21	19-Aug-21	29-Oct-21		21-Jan-22
C19IMPACT_1716	Trinidad and Tobago	12-Aug-21	MoH	N	N	N	N	N	N	18.00		16-Aug-21	19-Aug-21	27-Aug-21		09-Sept-21
C19IMPACT_1717	Trinidad and Tobago	12-Aug-21	MoH	N	N	N	N	N	Y	18.00		16-Aug-21	19-Aug-21	27-Aug-21		09-Sept-21
C19IMPACT_1718	Trinidad and Tobago	09-Aug-21	MoH	N	N	N	N	N	Y		8.00	16-Aug-21	23-Aug-21	27-Aug-21		21-Jan-22
C19IMPACT_1719	Trinidad and Tobago	09-Aug-21	CARPHA	N	N	N	N	N	Y	25.57		13-Aug-21	23-Aug-21	27-Aug-21	26-Aug-21	
C19IMPACT_1720	Trinidad and Tobago	09-Aug-21	CARPHA	N	N	N	N	N	Y	14.99		13-Aug-21	23-Aug-21	27-Aug-21	26-Aug-21	21-Jan-22
C19IMPACT_1721	Trinidad and Tobago	09-Aug-21	MoH	N	N	N	N	N	Y	18.50		16-Aug-21	23-Aug-21	27-Aug-21		21-Jan-22
C19IMPACT_1722	Trinidad and Tobago	10-Aug-21	MoH	N	N	N	N	N	Y	17.90		16-Aug-21	23-Aug-21	27-Aug-21		21-Jan-22
C19IMPACT_1723	Grenada	10-Aug-21	CARPHA	N	N	N	N	N	Y	17.16		17-Aug-21	23-Aug-21		26-Aug-21	21-Jan-22
C19IMPACT_1724	Grenada	10-Aug-21	CARPHA	N	N	N	N	N	Y	21.80		17-Aug-21	23-Aug-21		26-Aug-21	21-Jan-22

C19IMPACT_1725	Grenada	10-Aug-21	CARPHA	N	N	N	N	N	Y	20.47		17-Aug-21	23-Aug-21		26-Aug-21	21-Jan-22
C19IMPACT_1726	Trinidad and Tobago	10-Aug-21	MoH	N	N	N	N	N	Y	18.40		16-Aug-21	23-Aug-21	27-Aug-21		
C19IMPACT_1727	Trinidad and Tobago	11-Aug-21	MoH	N	N	N	N	N	Y	18.00		16-Aug-21	23-Aug-21	27-Aug-21		21-Jan-22
C19IMPACT_1728	Trinidad and Tobago	11-Aug-21	MoH	N	N	N	N	N	Y	28.00		16-Aug-21	23-Aug-21	27-Aug-21		
C19IMPACT_1729	Trinidad and Tobago	11-Aug-21	MoH	N	N	N	N	N	Y	18.40		16-Aug-21	23-Aug-21	27-Aug-21		21-Jan-22
C19IMPACT_1730	Trinidad and Tobago	11-Aug-21	MoH	N	N	N	N	N	Y	16.60		16-Aug-21	23-Aug-21	27-Aug-21		21-Jan-22
C19IMPACT_1731	Trinidad and Tobago	11-Aug-21	MoH	N	N	N	N	N	Y	18.00		16-Aug-21	23-Aug-21	27-Aug-21		21-Jan-22
C19IMPACT_1732	Trinidad and Tobago	12-Aug-21	MoH	N	N	N	N	N	Y	18.20		16-Aug-21	23-Aug-21	27-Aug-21		21-Jan-22
C19IMPACT_1733	Trinidad and Tobago	12-Aug-21	MoH	Y	N	Y	N	N	Y	19.00		17-Aug-21	23-Aug-21	27-Aug-21		21-Jan-22
C19IMPACT_1734	Trinidad and Tobago	12-Aug-21	MoH	N	N	N	N	Y	Y	19.50		16-Aug-21	23-Aug-21	27-Aug-21		
C19IMPACT_1735	Trinidad and Tobago	12-Aug-21	MoH	N	N	N	N	N	Y	14.70		16-Aug-21	23-Aug-21	27-Aug-21		21-Jan-22
C19IMPACT_1736	Trinidad and Tobago	12-Aug-21	MoH	N	N	Y	N	N	Y	15.60		16-Aug-21	23-Aug-21	27-Aug-21		21-Jan-22
C19IMPACT_1737	Trinidad and Tobago	12-Aug-21	MoH	N	N	N	N	N	Y	19.40		16-Aug-21	23-Aug-21	27-Aug-21		21-Jan-22
C19IMPACT_1738	Trinidad and Tobago	12-Aug-21	MoH	N	N	N	N	N	Y	18.10		16-Aug-21	23-Aug-21	27-Aug-21		21-Jan-22
C19IMPACT_1739	Trinidad and Tobago	12-Aug-21	MoH	N	N	N	N	N	Y	19.60		16-Aug-21	23-Aug-21	27-Aug-21		21-Jan-22
C19IMPACT_1740	Trinidad and Tobago	13-Aug-21	MoH	N	N	N	N	N	Y	17.00		17-Aug-21	23-Aug-21	27-Aug-21		
C19IMPACT_1741	Trinidad and Tobago	13-Aug-21	MoH	Y	N	Y	N	N	Y	16.00		17-Aug-21	23-Aug-21	27-Aug-21		21-Jan-22
C19IMPACT_1742	Trinidad and Tobago	13-Aug-21	MoH	N	N	N	N	Y	Y	18.00		17-Aug-21	23-Aug-21	27-Aug-21		21-Jan-22
C19IMPACT_1743	Trinidad and Tobago	13-Aug-21	MoH	N	N	N	N	N	Y	18.00		17-Aug-21	23-Aug-21	27-Aug-21		
C19IMPACT_1744	Trinidad and Tobago	13-Aug-21	MoH	N	N	N	N	N	Y	17.00		17-Aug-21	23-Aug-21	27-Aug-21		
C19IMPACT_1745	Trinidad and Tobago	13-Aug-21	MoH	N	N	N	N	N	Y	19.00		17-Aug-21	23-Aug-21	27-Aug-21		
C19IMPACT_1746	Trinidad and Tobago	13-Aug-21	MoH	N	N	N	N	N	Y	19.00		17-Aug-21	23-Aug-21	27-Aug-21		
C19IMPACT_1747	Trinidad and Tobago	13-Aug-21	MoH	N	N	N	N	N	Y	19.00		17-Aug-21	23-Aug-21	27-Aug-21		
C19IMPACT_1748	Trinidad and Tobago	13-Aug-21	MoH	N	N	N	N	N	Y	18.00		17-Aug-21	23-Aug-21	27-Aug-21		21-Jan-22
C19IMPACT_1749	Trinidad and Tobago	13-Aug-21	MoH	N	N	N	N	N	Y	13.00		17-Aug-21	23-Aug-21	27-Aug-21		21-Jan-22
C19IMPACT_1750	Trinidad and Tobago	13-Aug-21	MoH	Y	N	Y	N	N	Y	19.00		17-Aug-21	23-Aug-21	27-Aug-21		21-Jan-22
C19IMPACT_1751	Trinidad and Tobago	13-Aug-21	MoH	Y	N	Y	N	Y	Y	19.00		17-Aug-21	23-Aug-21	27-Aug-21		21-Jan-22
C19IMPACT_1752	Trinidad and Tobago	13-Aug-21	MoH	N	N	N	N	Y	Y	19.00		17-Aug-21	23-Aug-21	27-Aug-21		21-Jan-22
C19IMPACT_1753	Trinidad and Tobago	13-Aug-21	MoH	N	N	N	N	N	Y	16.00		17-Aug-21	23-Aug-21	27-Aug-21		21-Jan-22
C19IMPACT_1754	Trinidad and Tobago	13-Aug-21	MoH	N	N	Y	N	N	Y	15.00		17-Aug-21	23-Aug-21	27-Aug-21		21-Jan-22
C19IMPACT_1755	Trinidad and Tobago	13-Aug-21	MoH	N	N	N	N	N	Y	17.00		17-Aug-21	23-Aug-21	27-Aug-21		21-Jan-22
C19IMPACT_1756	Trinidad and Tobago	13-Aug-21	CARPHA	N	N	N	N	N	Y	18.45		17-Aug-21	23-Aug-21	27-Aug-21	26-Aug-21	21-Jan-22
C19IMPACT_1757	Trinidad and Tobago	13-Aug-21	CARPHA	N	N	Y	N	N	Y	21.09		17-Aug-21	23-Aug-21	27-Aug-21	26-Aug-21	21-Jan-22
C19IMPACT_1758	Trinidad and Tobago	13-Aug-21	MoH	N	N	N	N	Y	Y	17.40		16-Aug-21	23-Aug-21	27-Aug-21		21-Jan-22
C19IMPACT_1759	Trinidad and Tobago	13-Aug-21	MoH	N	N	N	N	N	Y	23.00		16-Aug-21	23-Aug-21	27-Aug-21		21-Jan-22
C19IMPACT_1760	Trinidad and Tobago	13-Aug-21	MoH	Y	N	Y	N	N	Y	16.00		18-Aug-21	23-Aug-21	27-Aug-21		21-Jan-22
C19IMPACT_1761	Trinidad and Tobago	13-Aug-21	MoH	N	N	N	N	Y	Y	17.00		17-Aug-21	23-Aug-21	27-Aug-21		21-Jan-22
C19IMPACT_1762	Trinidad and Tobago	13-Aug-21	MoH	N	N	N	N	N	Y	17.00		17-Aug-21	23-Aug-21	27-Aug-21		21-Jan-22
C19IMPACT_1763	Trinidad and Tobago	14-Aug-21	MoH	Y	N	Y	N	N	Y	17.00		17-Aug-21	23-Aug-21	27-Aug-21		21-Jan-22
C19IMPACT_1764	Trinidad and Tobago	14-Aug-21	MoH	N	N	N	N	Y	Y	21.00		17-Aug-21	23-Aug-21	27-Aug-21		21-Jan-22
C19IMPACT_1765	Trinidad and Tobago	14-Aug-21	MoH	N	N	Y	N	N	Y	18.30		16-Aug-21	23-Aug-21	27-Aug-21		21-Jan-22
C19IMPACT_1766	Trinidad and Tobago	14-Aug-21	CARPHA	N	N	Y	N	N	Y	15.19		17-Aug-21	23-Aug-21	27-Aug-21	26-Aug-21	21-Jan-22
C19IMPACT_1767	Trinidad and Tobago	14-Aug-21	MoH	Y	N	Y	N	Y	Y	18.00		17-Aug-21	23-Aug-21	27-Aug-21		21-Jan-22
C19IMPACT_1768	Trinidad and Tobago	14-Aug-21	MoH	N	N	N	N	Y	Y	19.00		17-Aug-21	23-Aug-21	27-Aug-21		21-Jan-22
C19IMPACT_1769	Trinidad and Tobago	14-Aug-21	MoH	N	N	N	N	N	Y	17.00		17-Aug-21	23-Aug-21	27-Aug-21		21-Jan-22
C19IMPACT_1770	Trinidad and Tobago	14-Aug-21	MoH	N	N	N	N	N	Y	19.00		17-Aug-21	23-Aug-21	27-Aug-21		21-Jan-22
C19IMPACT_1771	Trinidad and Tobago	14-Aug-21	MoH	N	N	N	N	N	Y	17.00		17-Aug-21	23-Aug-21	27-Aug-21		21-Jan-22
C19IMPACT_1772	Trinidad and Tobago	14-Aug-21	MoH	N	N	N	N	N	Y	17.00		17-Aug-21	23-Aug-21	27-Aug-21		21-Jan-22
C19IMPACT_1773	Trinidad and Tobago	15-Aug-21	CARPHA	N	N	Y	N	N	Y	16.87		17-Aug-21	23-Aug-21	27-Aug-21	26-Aug-21	21-Jan-22
C19IMPACT_1774	Trinidad and Tobago	15-Aug-21	CARPHA	Y	N	Y	N	N	Y	18.67		17-Aug-21	23-Aug-21	27-Aug-21	26-Aug-21	21-Jan-22
C19IMPACT_1775	Trinidad and Tobago	15-Aug-21	CARPHA	N	N	Y	N	Y	Y	15.61		17-Aug-21	23-Aug-21	27-Aug-21	26-Aug-21	21-Jan-22
C19IMPACT_1776	Trinidad and Tobago	15-Aug-21	CARPHA	N	N	Y	N	N	Y	15.80		17-Aug-21	23-Aug-21	27-Aug-21	26-Aug-21	21-Jan-22
C19IMPACT_1777	Trinidad and Tobago	15-Aug-21	MoH	N	N	N	N	N	Y	17.00		17-Aug-21	23-Aug-21	27-Aug-21		
C19IMPACT_1778	Trinidad and Tobago	15-Aug-21	MoH	N	N	N	N	N	Y	19.00		17-Aug-21	23-Aug-21	27-Aug-21		21-Jan-22
C19IMPACT_1779	Trinidad and Tobago	15-Aug-21	MoH	N	N	N	N	N	Y	18.00		17-Aug-21	23-Aug-21	27-Aug-21		21-Jan-22
C19IMPACT_1780	Trinidad and Tobago	15-Aug-21	MoH	N	N	N	N	N	Y	19.00		17-Aug-21	23-Aug-21	27-Aug-21		21-Jan-22
C19IMPACT_1781	Trinidad and Tobago	15-Aug-21	MoH	N	N	N	N	N	Y	15.00		18-Aug-21	23-Aug-21	27-Aug-21		21-Jan-22
C19IMPACT_1782	Trinidad and Tobago	16-Aug-21	MoH	N	N	N	N	N	Y	17.00		17-Aug-21	23-Aug-21	27-Aug-21		21-Jan-22
C19IMPACT_1783	Trinidad and Tobago	16-Aug-21	MoH	N	N	Y	N	N	Y	19.00		17-Aug-21	23-Aug-21	27-Aug-21		21-Jan-22
C19IMPACT_1784	Trinidad and Tobago	16-Aug-21	MoH	Y	N	Y	N	Y	Y	19.00		17-Aug-21	23-Aug-21	27-Aug-21		21-Jan-22
C19IMPACT_1785	Trinidad and Tobago	16-Aug-21	MoH	Y	N	Y	N	Y	Y	16.00		17-Aug-21	23-Aug-21	27-Aug-21		21-Jan-22
C19IMPACT_1786	Trinidad and Tobago	16-Aug-21	MoH	Y	N	Y	N	Y	Y	17.00		17-Aug-21	23-Aug-21	27-Aug-21		21-Jan-22
C19IMPACT_1787	Trinidad and Tobago	16-Aug-21	MoH	Y	N	Y	N	Y	Y	18.00		17-Aug-21	23-Aug-21	27-Aug-21		21-Jan-22
C19IMPACT_1788	Trinidad and Tobago	16-Aug-21	MoH	N	N	N	N	Y	Y	17.00		17-Aug-21	23-Aug-21	27-Aug-21		21-Jan-22

C19IMPACT_1789	Trinidad and Tobago	16-Aug-21	MoH	N	N	N	N	N	Y	19.00		17-Aug-21	23-Aug-21	27-Aug-21		21-Jan-22
C19IMPACT_1790	Trinidad and Tobago	16-Aug-21	MoH	N	N	N	N	N	Y	16.00		17-Aug-21	23-Aug-21	27-Aug-21		21-Jan-22
C19IMPACT_1791	Trinidad and Tobago	16-Aug-21	MoH	N	N	N	N	N	Y	19.00		17-Aug-21	23-Aug-21	27-Aug-21		21-Jan-22
C19IMPACT_1792	Trinidad and Tobago	16-Aug-21	MoH	N	N	N	N	N	Y	17.00		18-Aug-21	23-Aug-21	27-Aug-21		
C19IMPACT_1793	Trinidad and Tobago	16-Aug-21	MoH	N	N	N	N	N	Y	16.00		18-Aug-21	23-Aug-21	27-Aug-21		
C19IMPACT_1794	Trinidad and Tobago	16-Aug-21	MoH	N	N	N	N	N	Y	19.00		18-Aug-21	23-Aug-21	27-Aug-21		21-Jan-22
C19IMPACT_1795	Trinidad and Tobago	16-Aug-21	MoH	N	N	N	N	N	Y	18.00		18-Aug-21	23-Aug-21	27-Aug-21		21-Jan-22
C19IMPACT_1796	Trinidad and Tobago	16-Aug-21	MoH	N	N	N	N	N	Y	17.00		18-Aug-21	23-Aug-21	27-Aug-21		21-Jan-22
C19IMPACT_1797	Trinidad and Tobago	16-Aug-21	MoH	N	N	N	N	N	Y	17.00		18-Aug-21	23-Aug-21	27-Aug-21		21-Jan-22
C19IMPACT_1798	Trinidad and Tobago	16-Aug-21	MoH	N	N	N	N	N	Y	18.00		18-Aug-21	23-Aug-21	27-Aug-21		21-Jan-22
C19IMPACT_1799	Trinidad and Tobago	16-Aug-21	MoH	N	N	N	N	N	N	20.00		18-Aug-21	23-Aug-21	27-Aug-21		21-Jan-22
C19IMPACT_1800	Trinidad and Tobago	16-Aug-21	MoH	N	N	N	N	N	Y			18-Aug-21	23-Aug-21	27-Aug-21		21-Jan-22
C19IMPACT_1801	Trinidad and Tobago	16-Aug-21	MoH	N	N	N	N	N	Y	20.00		18-Aug-21	23-Aug-21	27-Aug-21		21-Jan-22
C19IMPACT_1802	Trinidad and Tobago	16-Aug-21	MoH	N	N	N	N	N	N	20.00		18-Aug-21	23-Aug-21	27-Aug-21		21-Jan-22
C19IMPACT_1803	Trinidad and Tobago	16-Aug-21	MoH	N	N	N	N	N	Y	18.00		18-Aug-21	23-Aug-21	27-Aug-21		21-Jan-22
C19IMPACT_1804	Trinidad and Tobago	16-Aug-21	MoH	N	N	N	N	N	Y	19.00		18-Aug-21	23-Aug-21	27-Aug-21		21-Jan-22
C19IMPACT_1805	Trinidad and Tobago	16-Aug-21	MoH	N	N	N	N	N	Y	18.00		17-Aug-21	23-Aug-21	27-Aug-21		
C19IMPACT_1806	Trinidad and Tobago	18-Jul-21	MoH	Y	N	Y	N	N	Y	19.00		20-Aug-21	26-Aug-21	27-Aug-21		18-Dec-21
C19IMPACT_1807	Dominica	06-Aug-21	CARPHA	N	N	N	N	Y	Y	16.36		18-Aug-21	26-Aug-21		26-Aug-21	18-Dec-21
C19IMPACT_1808	Trinidad and Tobago	07-Aug-21	MoH	N	N	N	N	N	Y		4.35	18-Aug-21	26-Aug-21	20-Aug-21		18-Dec-21
C19IMPACT_1809	Dominica	07-Aug-21	CARPHA	N	N	N	N	N	Y	15.59		18-Aug-21	26-Aug-21		26-Aug-21	18-Dec-21
C19IMPACT_1810	Dominica	07-Aug-21	CARPHA	N	N	N	N	N	Y	15.84		18-Aug-21	26-Aug-21		26-Aug-21	18-Dec-21
C19IMPACT_1811	Dominica	07-Aug-21	CARPHA	N	N	N	N	N	Y	20.39		18-Aug-21	26-Aug-21		26-Aug-21	18-Dec-21
C19IMPACT_1812	Dominica	07-Aug-21	CARPHA	N	N	N	N	N	Y	17.80		18-Aug-21	26-Aug-21		26-Aug-21	18-Dec-21
C19IMPACT_1813	Dominica	07-Aug-21	CARPHA	N	N	N	N	N	Y	16.22		18-Aug-21	26-Aug-21		26-Aug-21	18-Dec-21
C19IMPACT_1814	Dominica	07-Aug-21	CARPHA	N	N	N	N	N	Y	16.93		18-Aug-21	26-Aug-21		26-Aug-21	18-Dec-21
C19IMPACT_1815	Dominica	07-Aug-21	CARPHA	N	N	N	N	N	Y	16.53		18-Aug-21	26-Aug-21		26-Aug-21	18-Dec-21
C19IMPACT_1816	Dominica	07-Aug-21	CARPHA	N	N	N	N	N	Y	14.93		18-Aug-21	26-Aug-21		26-Aug-21	18-Dec-21
C19IMPACT_1817	Dominica	07-Aug-21	CARPHA	N	N	N	N	N	Y	15.16		18-Aug-21	26-Aug-21		26-Aug-21	18-Dec-21
C19IMPACT_1818	Trinidad and Tobago	10-Aug-21	MoH	N	N	N	N	N	Y		3.12	18-Aug-21	26-Aug-21	27-Aug-21		18-Dec-21
C19IMPACT_1819	Trinidad and Tobago	10-Aug-21	MoH	N	N	N	N	N	Y		4.38	18-Aug-21	26-Aug-21	27-Aug-21		18-Dec-21
C19IMPACT_1820	Trinidad and Tobago	11-Aug-21	MoH	N	N	N	N	N	Y		3.26	18-Aug-21	26-Aug-21	27-Aug-21		18-Dec-21
C19IMPACT_1821	Trinidad and Tobago	12-Aug-21	CARPHA	N	N	N	N	N	Y	17.66		18-Aug-21	26-Aug-21	27-Aug-21	26-Aug-21	18-Dec-21
C19IMPACT_1822	Saint Vincent and the Grenadines	12-Aug-21	CARPHA	N	N	N	N	N	Y	19.64		18-Aug-21	26-Aug-21		26-Aug-21	18-Dec-21
C19IMPACT_1823	Saint Vincent and the Grenadines	12-Aug-21	CARPHA	N	N	N	N	N	Y	16.60		18-Aug-21	26-Aug-21		26-Aug-21	18-Dec-21
C19IMPACT_1824	Trinidad and Tobago	13-Aug-21	MoH	N	N	N	N	N	Y	16.00		20-Aug-21	26-Aug-21	27-Aug-21		18-Dec-21
C19IMPACT_1825	Trinidad and Tobago	16-Aug-21	MoH	Y	Y	Y	N	N	Y	19.00		20-Aug-21	26-Aug-21	27-Aug-21		18-Dec-21
C19IMPACT_1826	Trinidad and Tobago	16-Aug-21	MoH	Y	N	Y	N	Y	Y	16.00		20-Aug-21	26-Aug-21	27-Aug-21		18-Dec-21
C19IMPACT_1827	Trinidad and Tobago	16-Aug-21	MoH	N	N	N	N	Y	Y	17.00		20-Aug-21	26-Aug-21	27-Aug-21		18-Dec-21
C19IMPACT_1828	Trinidad and Tobago	16-Aug-21	MoH	Y	Y	Y	N	N	N	20.00		18-Aug-21	26-Aug-21	27-Aug-21		18-Dec-21
C19IMPACT_1829	Trinidad and Tobago	16-Aug-21	CARPHA	Y	N	Y	N	Y	Y	18.17		18-Aug-21	26-Aug-21	27-Aug-21	26-Aug-21	18-Dec-21
C19IMPACT_1830	Trinidad and Tobago	16-Aug-21	MoH	Y	N	Y	N	Y	Y	16.00		20-Aug-21	26-Aug-21	27-Aug-21		18-Dec-21
C19IMPACT_1831	Trinidad and Tobago	16-Aug-21	MoH	N	N	Y	N	Y	Y			20-Aug-21	26-Aug-21	27-Aug-21		18-Dec-21
C19IMPACT_1832	Trinidad and Tobago	16-Aug-21	MoH	N	N	N	N	N	Y	18.00		20-Aug-21	26-Aug-21	27-Aug-21		18-Dec-21
C19IMPACT_1833	Trinidad and Tobago	17-Aug-21	MoH	Y	N	Y	N	N	Y	16.00		20-Aug-21	26-Aug-21	27-Aug-21		18-Dec-21
C19IMPACT_1834	Trinidad and Tobago	17-Aug-21	MoH	N	N	N	N	Y	Y	17.00		20-Aug-21	26-Aug-21	27-Aug-21		18-Dec-21
C19IMPACT_1835	Trinidad and Tobago	17-Aug-21	MoH	N	N	N	N	N	Y	19.00		20-Aug-21	26-Aug-21	27-Aug-21		18-Dec-21
C19IMPACT_1836	Trinidad and Tobago	17-Aug-21	MoH	N	N	N	N	N	Y	17.00		20-Aug-21	26-Aug-21	27-Aug-21		18-Dec-21
C19IMPACT_1837	Trinidad and Tobago	17-Aug-21	MoH	N	N	Y	N	N	Y	18.00		20-Aug-21	26-Aug-21	27-Aug-21		18-Dec-21
C19IMPACT_1838	Trinidad and Tobago	17-Aug-21	CARPHA	Y	N	Y	N	Y	Y	16.56		18-Aug-21	26-Aug-21	27-Aug-21	26-Aug-21	18-Dec-21
C19IMPACT_1839	Trinidad and Tobago	17-Aug-21	MoH	Y	N	Y	N	Y	Y	19.00		20-Aug-21	26-Aug-21	27-Aug-21		18-Dec-21
C19IMPACT_1840	Trinidad and Tobago	17-Aug-21	MoH	N	N	Y	N	Y	Y	18.00		20-Aug-21	26-Aug-21	27-Aug-21		18-Dec-21
C19IMPACT_1841	Trinidad and Tobago	17-Aug-21	MoH	N	N	Y	N	Y	Y	15.00		20-Aug-21	26-Aug-21	27-Aug-21		18-Dec-21
C19IMPACT_1842	Trinidad and Tobago	17-Aug-21	MoH	Y	N	Y	N	Y	Y	19.00		20-Aug-21	26-Aug-21	27-Aug-21		18-Dec-21
C19IMPACT_1843	Trinidad and Tobago	17-Aug-21	MoH	Y	N	Y	N	Y	Y	16.00		20-Aug-21	26-Aug-21	27-Aug-21		18-Dec-21
C19IMPACT_1844	Trinidad and Tobago	17-Aug-21	MoH	Y	N	Y	N	Y	Y	19.00		20-Aug-21	26-Aug-21	27-Aug-21		18-Dec-21
C19IMPACT_1845	Trinidad and Tobago	17-Aug-21	MoH	N	N	N	N	Y	Y	18.00		20-Aug-21	26-Aug-21	27-Aug-21		18-Dec-21
C19IMPACT_1846	Trinidad and Tobago	17-Aug-21	MoH	N	N	N	N	N	Y	17.00		20-Aug-21	26-Aug-21	27-Aug-21		18-Dec-21
C19IMPACT_1847	Trinidad and Tobago	18-Aug-21	MoH	Y	N	Y	N	N	Y	18.00		20-Aug-21	26-Aug-21	27-Aug-21		18-Dec-21
C19IMPACT_1848	Trinidad and Tobago	18-Aug-21	MoH	N	N	N	N	Y	Y	18.00		20-Aug-21	26-Aug-21	27-Aug-21		18-Dec-21
C19IMPACT_1849	British Virgin Islands	12-Aug-21	CARPHA	N	N	N	N	N	Y	18.63		20-Aug-21	01-Sept-21		05-Sept-21	
C19IMPACT_1850	British Virgin Islands	12-Aug-21	CARPHA	N	N	N	N	N	Y	18.27		20-Aug-21	01-Sept-21		05-Sept-21	10-Oct-21
C19IMPACT_1851	British Virgin Islands	12-Aug-21	CARPHA	N	N	N	N	N	Y	16.40		20-Aug-21	01-Sept-21		05-Sept-21	10-Oct-21
C19IMPACT_1852	Anguilla	13-Aug-21	CARPHA	N	N	N	N	N	Y	14.58		20-Aug-21	01-Sept-21		05-Sept-21	10-Oct-21

C19IMPACT_1853	Trinidad and Tobago	13-Aug-21	CARPHA	N	N	N	N	N	Y	15.75		20-Aug-21	01-Sept-21	05-Sept-21	05-Sept-21	10-Oct-21
C19IMPACT_1854	Antigua and Barbuda	13-Aug-21	CARPHA	N	N	N	N	N	Y	19.98		20-Aug-21	01-Sept-21		05-Sept-21	10-Oct-21
C19IMPACT_1855	Antigua and Barbuda	13-Aug-21	CARPHA	N	N	N	N	N	Y	20.41		20-Aug-21	01-Sept-21		05-Sept-21	10-Oct-21
C19IMPACT_1856	Trinidad and Tobago	14-Aug-21	CARPHA	N	N	N	N	N	Y	16.68		20-Aug-21	01-Sept-21	05-Sept-21	05-Sept-21	10-Oct-21
C19IMPACT_1857	Trinidad and Tobago	14-Aug-21	CARPHA	N	N	N	N	N	Y	15.44		20-Aug-21	01-Sept-21	05-Sept-21	05-Sept-21	10-Oct-21
C19IMPACT_1858	Antigua and Barbuda	14-Aug-21	CARPHA	N	N	N	N	N	Y	20.12		20-Aug-21	01-Sept-21		05-Sept-21	10-Oct-21
C19IMPACT_1859	Montserrat	14-Aug-21	CARPHA	N	N	N	N	N	Y	21.95		20-Aug-21	01-Sept-21		05-Sept-21	10-Oct-21
C19IMPACT_1860	Antigua and Barbuda	14-Aug-21	CARPHA	N	N	N	N	N	Y	19.07		20-Aug-21	01-Sept-21		05-Sept-21	10-Oct-21
C19IMPACT_1861	Trinidad and Tobago	15-Aug-21	CARPHA	N	N	N	N	N	Y	17.91		20-Aug-21	01-Sept-21	05-Sept-21	05-Sept-21	10-Oct-21
C19IMPACT_1862	Antigua and Barbuda	16-Aug-21	CARPHA	N	N	N	N	N	Y	18.36		20-Aug-21	01-Sept-21		05-Sept-21	
C19IMPACT_1863	Trinidad and Tobago	16-Aug-21	CARPHA	N	N	N	N	N	Y	19.40		20-Aug-21	01-Sept-21	05-Sept-21	05-Sept-21	10-Oct-21
C19IMPACT_1864	Trinidad and Tobago	16-Aug-21	CARPHA	N	N	N	N	N	Y	20.13		20-Aug-21	01-Sept-21	05-Sept-21	05-Sept-21	10-Oct-21
C19IMPACT_1865	Trinidad and Tobago	17-Aug-21	CARPHA	N	N	N	N	N	Y	16.83		20-Aug-21	01-Sept-21	05-Sept-21	05-Sept-21	10-Oct-21
C19IMPACT_1866	Antigua and Barbuda	18-Aug-21	CARPHA	N	N	N	N	N	Y	21.64		20-Aug-21	01-Sept-21		05-Sept-21	10-Oct-21
C19IMPACT_1867	Antigua and Barbuda		CARPHA	N	N	N	N	N	Y	17.38		20-Aug-21	01-Sept-21		05-Sept-21	
C19IMPACT_1868	Antigua and Barbuda		CARPHA	N	N	N	N	N	Y	16.36		20-Aug-21	01-Sept-21		05-Sept-21	
C19IMPACT_1869	Antigua and Barbuda	06-Aug-21	CARPHA	N	N	N	N	N	Y	14.44		20-Aug-21	06-Sept-21		10-Sept-21	21-Jan-22
C19IMPACT_1870	Antigua and Barbuda	07-Aug-21	CARPHA	N	N	N	N	N	Y	22.06		20-Aug-21	06-Sept-21		10-Sept-21	
C19IMPACT_1871	Antigua and Barbuda	07-Aug-21	CARPHA	N	N	N	N	N	Y	18.86		20-Aug-21	06-Sept-21		10-Sept-21	21-Jan-22
C19IMPACT_1872	Antigua and Barbuda	09-Aug-21	CARPHA	N	N	N	N	N	Y	23.16		20-Aug-21	06-Sept-21		10-Sept-21	21-Jan-22
C19IMPACT_1873	Antigua and Barbuda	09-Aug-21	CARPHA	N	N	N	N	N	Y	19.67		20-Aug-21	06-Sept-21		10-Sept-21	21-Jan-22
C19IMPACT_1874	Antigua and Barbuda	09-Aug-21	CARPHA	N	N	N	N	N	Y	19.62		20-Aug-21	06-Sept-21		10-Sept-21	21-Jan-22
C19IMPACT_1875	Antigua and Barbuda	09-Aug-21	CARPHA	N	N	N	N	N	Y	15.02		20-Aug-21	06-Sept-21		10-Sept-21	21-Jan-22
C19IMPACT_1876	Antigua and Barbuda	09-Aug-21	CARPHA	N	N	N	N	N	Y	19.05		20-Aug-21	06-Sept-21		10-Sept-21	21-Jan-22
C19IMPACT_1877	Antigua and Barbuda	09-Aug-21	CARPHA	N	N	N	N	N	Y	19.90		20-Aug-21	06-Sept-21		10-Sept-21	21-Jan-22
C19IMPACT_1878	Antigua and Barbuda	12-Aug-21	CARPHA	N	N	N	N	N	Y	15.69		20-Aug-21	06-Sept-21		10-Sept-21	
C19IMPACT_1879	Antigua and Barbuda	12-Aug-21	CARPHA	N	N	N	N	N	Y	15.26		20-Aug-21	06-Sept-21		10-Sept-21	21-Jan-22
C19IMPACT_1880	Saint Vincent and the Grenadines	14-Aug-21	CARPHA	N	N	N	N	N	Y	21.19		20-Aug-21	06-Sept-21		10-Sept-21	21-Jan-22
C19IMPACT_1881	Anguilla	14-Aug-21	CARPHA	N	N	N	N	N	Y	16.79		20-Aug-21	06-Sept-21		10-Sept-21	21-Jan-22
C19IMPACT_1882	Saint Vincent and the Grenadines	14-Aug-21	CARPHA	N	N	N	N	N	Y	18.24		20-Aug-21	06-Sept-21		10-Sept-21	21-Jan-22
C19IMPACT_1883	Trinidad and Tobago	14-Aug-21	CARPHA	N	N	N	N	N	Y	19.27		20-Aug-21	06-Sept-21	10-Sept-21	10-Sept-21	21-Jan-22
C19IMPACT_1884	Trinidad and Tobago	14-Aug-21	CARPHA	N	N	N	N	N	Y	21.52		20-Aug-21	06-Sept-21	10-Sept-21	10-Sept-21	21-Jan-22
C19IMPACT_1885	Trinidad and Tobago	14-Aug-21	CARPHA	N	N	N	N	N	Y	21.94		20-Aug-21	06-Sept-21	10-Sept-21	10-Sept-21	21-Jan-22
C19IMPACT_1886	Antigua and Barbuda	15-Aug-21	CARPHA	N	N	N	N	N	Y	15.91		20-Aug-21	06-Sept-21		10-Sept-21	21-Jan-22
C19IMPACT_1887	Anguilla	15-Aug-21	CARPHA	N	N	N	N	N	Y	17.03		20-Aug-21	06-Sept-21		10-Sept-21	21-Jan-22
C19IMPACT_1888	Anguilla	16-Aug-21	CARPHA	N	N	N	N	N	Y	18.02		20-Aug-21	06-Sept-21		10-Sept-21	21-Jan-22
C19IMPACT_1889	Antigua and Barbuda	07-Aug-21	CARPHA	N	N	N	N	N	Y	15.11		20-Aug-21	08-Sept-21		10-Sept-21	21-Jan-22
C19IMPACT_1890	Antigua and Barbuda	09-Aug-21	CARPHA	N	N	N	N	N	Y	15.64		20-Aug-21	08-Sept-21		10-Sept-21	21-Jan-22
C19IMPACT_1891	Antigua and Barbuda	13-Aug-21	CARPHA	N	N	N	N	N	Y	18.93		20-Aug-21	08-Sept-21		10-Sept-21	21-Jan-22
C19IMPACT_1892	Saint Kitts and Nevis	14-Aug-21	CARPHA	N	N	N	N	N	N	14.66		25-Aug-21	08-Sept-21		10-Sept-21	21-Jan-22
C19IMPACT_1893	Saint Kitts and Nevis	14-Aug-21	CARPHA	N	N	N	N	N	N	14.73		25-Aug-21	08-Sept-21		10-Sept-21	21-Jan-22
C19IMPACT_1894	Saint Kitts and Nevis	14-Aug-21	CARPHA	N	N	N	N	N	N	17.38		25-Aug-21	08-Sept-21		10-Sept-21	21-Jan-22
C19IMPACT_1895	Saint Kitts and Nevis	15-Aug-21	CARPHA	N	N	N	N	N	N	15.11		25-Aug-21	08-Sept-21		10-Sept-21	
C19IMPACT_1896	Saint Kitts and Nevis	15-Aug-21	CARPHA	N	N	N	N	N	N	17.99		25-Aug-21	08-Sept-21		10-Sept-21	21-Jan-22
C19IMPACT_1897	Saint Kitts and Nevis	15-Aug-21	CARPHA	N	N	N	N	N	N	12.97		25-Aug-21	08-Sept-21		10-Sept-21	21-Jan-22
C19IMPACT_1898	Saint Kitts and Nevis	15-Aug-21	CARPHA	N	N	N	N	N	N	17.27		25-Aug-21	08-Sept-21		10-Sept-21	21-Jan-22
C19IMPACT_1899	Saint Kitts and Nevis	15-Aug-21	CARPHA	N	N	N	N	N	N	17.44		25-Aug-21	08-Sept-21		10-Sept-21	21-Jan-22
C19IMPACT_1900	Saint Kitts and Nevis	15-Aug-21	CARPHA	N	N	N	N	N	N	14.46		25-Aug-21	08-Sept-21		10-Sept-21	21-Jan-22
C19IMPACT_1901	Saint Kitts and Nevis	15-Aug-21	CARPHA	N	N	N	N	N	N	16.84		25-Aug-21	08-Sept-21		10-Sept-21	21-Jan-22
C19IMPACT_1902	Saint Kitts and Nevis	15-Aug-21	CARPHA	N	N	N	N	N	N	16.28		25-Aug-21	08-Sept-21		10-Sept-21	21-Jan-22
C19IMPACT_1903	Saint Kitts and Nevis	15-Aug-21	CARPHA	N	N	N	N	N	N	15.97		25-Aug-21	08-Sept-21		10-Sept-21	21-Jan-22
C19IMPACT_1904	Trinidad and Tobago	16-Aug-21	MoH	N	N	N	N	N	Y			20-Aug-21	08-Sept-21	10-Sept-21		21-Jan-22
C19IMPACT_1905	Trinidad and Tobago	17-Aug-21	CARPHA	N	N	N	N	N	N	25.49		25-Aug-21	08-Sept-21	10-Sept-21	10-Sept-21	
C19IMPACT_1906	Trinidad and Tobago	17-Aug-21	MoH	N	N	N	N	N	Y	17.00		20-Aug-21	08-Sept-21	10-Sept-21		21-Jan-22
C19IMPACT_1907	Trinidad and Tobago	17-Aug-21	MoH	N	N	N	N	N	Y			20-Aug-21	08-Sept-21	10-Sept-21		21-Jan-22
C19IMPACT_1908	Trinidad and Tobago	17-Aug-21	MoH	N	N	N	N	N	Y			20-Aug-21	08-Sept-21	10-Sept-21		21-Jan-22
C19IMPACT_1909	Trinidad and Tobago	18-Aug-21	MoH	N	N	N	N	N	Y	18.00		23-Aug-21	08-Sept-21	10-Sept-21		21-Jan-22
C19IMPACT_1910	Trinidad and Tobago	18-Aug-21	MoH	N	N	N	N	N	Y	16.00		23-Aug-21	08-Sept-21	10-Sept-21		21-Jan-22
C19IMPACT_1911	Trinidad and Tobago	19-Aug-21	MoH	N	N	N	N	N	Y	19.00		23-Aug-21	08-Sept-21	10-Sept-21		21-Jan-22
C19IMPACT_1912	Trinidad and Tobago	19-Aug-21	MoH	N	N	N	N	N	Y	16.00		23-Aug-21	08-Sept-21	10-Sept-21		21-Jan-22
C19IMPACT_1913	Trinidad and Tobago	19-Aug-21	MoH	N	N	N	N	N	Y	16.00		23-Aug-21	08-Sept-21	10-Sept-21		21-Jan-22
C19IMPACT_1914	Trinidad and Tobago	19-Aug-21	MoH	N	N	N	N	N	Y	19.00		23-Aug-21	08-Sept-21	10-Sept-21		21-Jan-22
C19IMPACT_1915	Trinidad and Tobago	20-Aug-21	MoH	N	N	N	N	N	Y	19.00		23-Aug-21	08-Sept-21	10-Sept-21		21-Jan-22
C19IMPACT_1916	Trinidad and Tobago	20-Aug-21	MoH	N	N	N	N	N	Y	20.00		23-Aug-21	08-Sept-21	10-Sept-21		21-Jan-22

C19IMPACT_1917	Trinidad and Tobago	20-Aug-21	MoH	N	N	N	N	N	Y	20.00		23-Aug-21	08-Sept-21	10-Sept-21		21-Jan-22
C19IMPACT_1918	Trinidad and Tobago	20-Aug-21	MoH	N	N	N	N	N	Y	19.00		23-Aug-21	08-Sept-21	10-Sept-21		21-Jan-22
C19IMPACT_1919	Trinidad and Tobago	20-Aug-21	MoH	N	N	N	N	N	Y	18.00		23-Aug-21	08-Sept-21	10-Sept-21		21-Jan-22
C19IMPACT_1920	Trinidad and Tobago	20-Aug-21	MoH	N	N	N	N	N	Y	18.00		23-Aug-21	08-Sept-21	10-Sept-21		21-Jan-22
C19IMPACT_1921	Trinidad and Tobago	20-Aug-21	MoH	N	N	N	N	N	Y	16.00		23-Aug-21	08-Sept-21	10-Sept-21		21-Jan-22
C19IMPACT_1922	Trinidad and Tobago	20-Aug-21	MoH	N	N	N	N	N	Y	19.00		23-Aug-21	08-Sept-21	10-Sept-21		21-Jan-22
C19IMPACT_1923	Trinidad and Tobago	20-Aug-21	MoH	N	N	N	N	N	Y	19.00		23-Aug-21	08-Sept-21	10-Sept-21		21-Jan-22
C19IMPACT_1924	Trinidad and Tobago	20-Aug-21	MoH	N	N	N	N	N	Y	19.00		23-Aug-21	08-Sept-21	10-Sept-21		21-Jan-22
C19IMPACT_1925	Trinidad and Tobago	20-Aug-21	MoH	N	N	N	N	N	Y	18.00		23-Aug-21	08-Sept-21	10-Sept-21		21-Jan-22
C19IMPACT_1926	Trinidad and Tobago	20-Aug-21	MoH	N	N	N	N	N	Y	18.00		23-Aug-21	08-Sept-21	10-Sept-21		21-Jan-22
C19IMPACT_1927	Trinidad and Tobago	20-Aug-21	MoH	N	N	N	N	N	Y	17.00		23-Aug-21	08-Sept-21	10-Sept-21		21-Jan-22
C19IMPACT_1928	Trinidad and Tobago	20-Aug-21	MoH	N	N	N	N	N	Y	16.00		23-Aug-21	08-Sept-21	10-Sept-21		
C19IMPACT_1929	Grenada	10-Aug-21	CARPHA	N	N	N	N	N	N	20.09		25-Aug-21	10-Sept-21		11-Sept-21	21-Jan-22
C19IMPACT_1930	Grenada	12-Aug-21	CARPHA	N	N	N	N	N	N	27.51		25-Aug-21	10-Sept-21		11-Sept-21	
C19IMPACT_1931	Grenada	12-Aug-21	CARPHA	N	N	N	N	N	N	20.01		25-Aug-21	10-Sept-21		11-Sept-21	21-Jan-22
C19IMPACT_1932	Trinidad and Tobago	13-Aug-21	MoH	N	N	N	N	N	Y	18.00		23-Aug-21	10-Sept-21	17-Sept-21		21-Jan-22
C19IMPACT_1933	Saint Kitts and Nevis	13-Aug-21	CARPHA	N	N	N	N	N	N	16.93		25-Aug-21	10-Sept-21		11-Sept-21	21-Jan-22
C19IMPACT_1934	Saint Vincent and the Grenadines	14-Aug-21	CARPHA	N	N	N	N	N	N	20.59		25-Aug-21	10-Sept-21		11-Sept-21	21-Jan-22
C19IMPACT_1935	Grenada	15-Aug-21	CARPHA	N	N	N	N	N	N	23.85		25-Aug-21	10-Sept-21		11-Sept-21	
C19IMPACT_1936	Trinidad and Tobago	16-Aug-21	MoH	N	N	N	N	N	Y			23-Aug-21	10-Sept-21	17-Sept-21		
C19IMPACT_1937	Grenada	16-Aug-21	CARPHA	N	N	N	N	N	N	20.48		25-Aug-21	10-Sept-21		11-Sept-21	21-Jan-22
C19IMPACT_1938	Grenada	16-Aug-21	CARPHA	N	N	N	N	N	N	22.14		25-Aug-21	10-Sept-21		11-Sept-21	21-Jan-22
C19IMPACT_1939	Saint Kitts and Nevis	16-Aug-21	CARPHA	N	N	N	N	N	N	15.96		25-Aug-21	10-Sept-21		11-Sept-21	21-Jan-22
C19IMPACT_1940	Grenada	16-Aug-21	CARPHA	N	N	N	N	N	N	23.62		25-Aug-21	10-Sept-21		11-Sept-21	21-Jan-22
C19IMPACT_1941	Trinidad and Tobago	17-Aug-21	MoH	N	N	N	N	N	Y	17.00		23-Aug-21	10-Sept-21	17-Sept-21		
C19IMPACT_1942	Trinidad and Tobago	17-Aug-21	MoH	N	N	N	N	N	Y	19.00		23-Aug-21	10-Sept-21	17-Sept-21		21-Jan-22
C19IMPACT_1943	Trinidad and Tobago	17-Aug-21	MoH	N	N	N	N	N	Y	15.00		23-Aug-21	10-Sept-21	17-Sept-21		21-Jan-22
C19IMPACT_1944	Trinidad and Tobago	17-Aug-21	CARPHA	N	N	N	N	N	N	24.20		25-Aug-21	10-Sept-21	17-Sept-21	11-Sept-21	
C19IMPACT_1945	Saint Kitts and Nevis	17-Aug-21	CARPHA	N	N	N	N	N	N	13.44		25-Aug-21	10-Sept-21		11-Sept-21	21-Jan-22
C19IMPACT_1946	Trinidad and Tobago	17-Aug-21	MoH	N	N	N	N	N	Y		27.00	23-Aug-21	10-Sept-21	17-Sept-21		
C19IMPACT_1947	Trinidad and Tobago	18-Aug-21	MoH	N	N	N	N	N	Y		4.00	23-Aug-21	10-Sept-21	17-Sept-21		21-Jan-22
C19IMPACT_1948	Trinidad and Tobago	18-Aug-21	MoH	N	N	N	N	N	Y	17.00		23-Aug-21	10-Sept-21	17-Sept-21		21-Jan-22
C19IMPACT_1949	Grenada	18-Aug-21	CARPHA	N	N	N	N	N	N	21.53		25-Aug-21	10-Sept-21		11-Sept-21	21-Jan-22
C19IMPACT_1950	Grenada	18-Aug-21	CARPHA	N	N	N	N	N	N	15.31		25-Aug-21	10-Sept-21		11-Sept-21	21-Jan-22
C19IMPACT_1951	Grenada	18-Aug-21	CARPHA	N	N	N	N	N	N	23.38		25-Aug-21	10-Sept-21		11-Sept-21	21-Jan-22
C19IMPACT_1952	Saint Kitts and Nevis	18-Aug-21	CARPHA	N	N	N	N	N	N	13.79		25-Aug-21	10-Sept-21		11-Sept-21	21-Jan-22
C19IMPACT_1953	Saint Kitts and Nevis	18-Aug-21	CARPHA	N	N	N	N	N	N	13.59		25-Aug-21	10-Sept-21		11-Sept-21	21-Jan-22
C19IMPACT_1954	Trinidad and Tobago	19-Aug-21	MoH	N	N	N	N	N	Y	19.00		23-Aug-21	10-Sept-21	17-Sept-21		21-Jan-22
C19IMPACT_1955	Trinidad and Tobago	19-Aug-21	MoH	N	N	N	N	N	Y	19.00		23-Aug-21	10-Sept-21	17-Sept-21		21-Jan-22
C19IMPACT_1956	Trinidad and Tobago	19-Aug-21	MoH	N	N	N	N	N	Y			23-Aug-21	10-Sept-21	17-Sept-21		21-Jan-22
C19IMPACT_1957	Trinidad and Tobago	19-Aug-21	MoH	N	N	N	N	N	N	15.00		24-Aug-21	10-Sept-21	17-Sept-21		21-Jan-22
C19IMPACT_1958	Trinidad and Tobago	19-Aug-21	MoH	N	N	N	N	N	N	15.00		24-Aug-21	10-Sept-21	17-Sept-21		21-Jan-22
C19IMPACT_1959	Trinidad and Tobago	19-Aug-21	MoH	N	N	N	N	N	Y	18.00		23-Aug-21	10-Sept-21	17-Sept-21		21-Jan-22
C19IMPACT_1960	Trinidad and Tobago	19-Aug-21	MoH	N	N	N	N	N	Y	18.00		23-Aug-21	10-Sept-21	17-Sept-21		21-Jan-22
C19IMPACT_1961	Trinidad and Tobago	19-Aug-21	MoH	N	N	N	N	N	Y	18.00		23-Aug-21	10-Sept-21	17-Sept-21		21-Jan-22
C19IMPACT_1962	Trinidad and Tobago	19-Aug-21	MoH	N	N	N	N	N	Y	20.00		23-Aug-21	10-Sept-21	17-Sept-21		21-Jan-22
C19IMPACT_1963	Trinidad and Tobago	19-Aug-21	MoH	N	N	N	N	N	Y	17.00		23-Aug-21	10-Sept-21	17-Sept-21		21-Jan-22
C19IMPACT_1964	Trinidad and Tobago	20-Aug-21	MoH	N	N	N	N	N	N	19.00		24-Aug-21	10-Sept-21	17-Sept-21		21-Jan-22
C19IMPACT_1965	Trinidad and Tobago	20-Aug-21	MoH	N	N	N	N	N	N	19.00		24-Aug-21	10-Sept-21	17-Sept-21		21-Jan-22
C19IMPACT_1966	Trinidad and Tobago	20-Aug-21	MoH	N	N	N	N	N	N	19.00		24-Aug-21	10-Sept-21	17-Sept-21		21-Jan-22
C19IMPACT_1967	Trinidad and Tobago	20-Aug-21	MoH	N	N	N	N	N	N	19.00		24-Aug-21	10-Sept-21	17-Sept-21		21-Jan-22
C19IMPACT_1968	Trinidad and Tobago	20-Aug-21	MoH	N	N	N	N	N	Y	15.00		23-Aug-21	10-Sept-21	17-Sept-21		21-Jan-22
C19IMPACT_1969	Trinidad and Tobago	20-Aug-21	MoH	N	N	N	N	N	N	18.00		24-Aug-21	10-Sept-21	17-Sept-21		21-Jan-22
C19IMPACT_1970	Trinidad and Tobago	20-Aug-21	MoH	N	N	N	N	N	N	19.00		24-Aug-21	10-Sept-21	17-Sept-21		21-Jan-22
C19IMPACT_1971	Trinidad and Tobago	20-Aug-21	MoH	N	N	N	N	N	N	19.00		24-Aug-21	10-Sept-21	17-Sept-21		21-Jan-22
C19IMPACT_1972	Trinidad and Tobago	20-Aug-21	CARPHA	N	N	N	N	N	N	19.51		25-Aug-21	10-Sept-21	17-Sept-21	11-Sept-21	21-Jan-22
C19IMPACT_1973	Trinidad and Tobago	04-Aug-21	CARPHA	N	N	N	N	N	N	24.49		25-Aug-21	13-Sept-21	17-Sept-21	15-Sept-21	
C19IMPACT_1974	Trinidad and Tobago	04-Aug-21	CARPHA	N	N	N	N	N	N	16.86		25-Aug-21	13-Sept-21	17-Sept-21	15-Sept-21	21-Jan-22
C19IMPACT_1975	Trinidad and Tobago	04-Aug-21	CARPHA	N	N	N	N	N	N	16.74		25-Aug-21	13-Sept-21	17-Sept-21	15-Sept-21	21-Jan-22
C19IMPACT_1976	Trinidad and Tobago	05-Aug-21	CARPHA	N	N	N	N	N	N	19.22		25-Aug-21	13-Sept-21	17-Sept-21	15-Sept-21	21-Jan-22
C19IMPACT_1977	Trinidad and Tobago	05-Aug-21	CARPHA	N	N	N	N	N	N	17.36		25-Aug-21	13-Sept-21	17-Sept-21	15-Sept-21	21-Jan-22
C19IMPACT_1978	Trinidad and Tobago	05-Aug-21	CARPHA	N	N	N	N	N	N	19.77		25-Aug-21	13-Sept-21	17-Sept-21	15-Sept-21	21-Jan-22
C19IMPACT_1979	Trinidad and Tobago	10-Aug-21	CARPHA	N	N	N	N	N	N	24.05		25-Aug-21	13-Sept-21	17-Sept-21	15-Sept-21	21-Jan-22
C19IMPACT_1980	Trinidad and Tobago	10-Aug-21	CARPHA	N	N	N	N	N	N	21.27		25-Aug-21	13-Sept-21	17-Sept-21	15-Sept-21	21-Jan-22

C19IMPACT_1981	Trinidad and Tobago	11-Aug-21	CARPHA	N	N	N	N	N	N	24.20		25-Aug-21	13-Sept-21	17-Sept-21	15-Sept-21	
C19IMPACT_1982	Trinidad and Tobago	11-Aug-21	CARPHA	N	N	N	N	N	N	17.34		25-Aug-21	13-Sept-21	17-Sept-21	15-Sept-21	21-Jan-22
C19IMPACT_1983	Trinidad and Tobago	11-Aug-21	CARPHA	N	N	N	N	N	N	21.37		25-Aug-21	13-Sept-21	17-Sept-21	15-Sept-21	21-Jan-22
C19IMPACT_1984	Trinidad and Tobago	12-Aug-21	CARPHA	N	N	N	N	N	N	25.67		25-Aug-21	13-Sept-21	17-Sept-21	15-Sept-21	
C19IMPACT_1985	Trinidad and Tobago	12-Aug-21	CARPHA	N	N	N	N	N	N	22.05		25-Aug-21	13-Sept-21	17-Sept-21	15-Sept-21	21-Jan-22
C19IMPACT_1986	Trinidad and Tobago	12-Aug-21	CARPHA	N	N	N	N	N	N	17.96		25-Aug-21	13-Sept-21	17-Sept-21	15-Sept-21	21-Jan-22
C19IMPACT_1987	Trinidad and Tobago	12-Aug-21	CARPHA	N	N	N	N	N	N	21.19		25-Aug-21	13-Sept-21	17-Sept-21	15-Sept-21	21-Jan-22
C19IMPACT_1988	Trinidad and Tobago	13-Aug-21	CARPHA	N	N	N	N	N	N	20.95		25-Aug-21	13-Sept-21	17-Sept-21	15-Sept-21	21-Jan-22
C19IMPACT_1989	Grenada	18-Aug-21	CARPHA	N	N	N	N	N	N	26.34		25-Aug-21	13-Sept-21		15-Sept-21	
C19IMPACT_1990	Grenada	18-Aug-21	CARPHA	N	N	N	N	N	N	26.96		25-Aug-21	13-Sept-21		15-Sept-21	
C19IMPACT_1991	Grenada	18-Aug-21	CARPHA	N	N	N	N	N	N	18.36		25-Aug-21	13-Sept-21		15-Sept-21	21-Jan-22
C19IMPACT_1992	Trinidad and Tobago	23-Aug-21	MoH	N	N	N	N	N	N	18.00		27-Aug-21	13-Sept-21	17-Sept-21		21-Jan-22
C19IMPACT_1993	Trinidad and Tobago	23-Aug-21	MoH	N	N	N	N	N	N	20.00		27-Aug-21	13-Sept-21	17-Sept-21		21-Jan-22
C19IMPACT_1994	Trinidad and Tobago	23-Aug-21	MoH	N	N	N	N	N	N	19.00		27-Aug-21	13-Sept-21	17-Sept-21		21-Jan-22
C19IMPACT_1995	Trinidad and Tobago	23-Aug-21	MoH	N	N	N	N	N	N	18.00		27-Aug-21	13-Sept-21	17-Sept-21		21-Jan-22
C19IMPACT_1996	Trinidad and Tobago	23-Aug-21	MoH	N	N	N	N	N	N	18.00		27-Aug-21	13-Sept-21	17-Sept-21		21-Jan-22
C19IMPACT_1997	Trinidad and Tobago	23-Aug-21	MoH	N	N	N	N	N	N	19.00		27-Aug-21	13-Sept-21	17-Sept-21		21-Jan-22
C19IMPACT_1998	Trinidad and Tobago	23-Aug-21	MoH	N	N	N	N	N	N	19.00		27-Aug-21	13-Sept-21	17-Sept-21		21-Jan-22
C19IMPACT_1999	Trinidad and Tobago	23-Aug-21	MoH	N	N	N	N	N	N	19.00		27-Aug-21	13-Sept-21	17-Sept-21		21-Jan-22
C19IMPACT_2000	Trinidad and Tobago	23-Aug-21	MoH	N	N	N	N	N	N	16.00		27-Aug-21	13-Sept-21	17-Sept-21		21-Jan-22
C19IMPACT_2001	Trinidad and Tobago	24-Aug-21	MoH	N	N	N	N	N	N	20.00		27-Aug-21	13-Sept-21	17-Sept-21		21-Jan-22
C19IMPACT_2002	Trinidad and Tobago	24-Aug-21	MoH	N	N	N	N	N	N	20.00		27-Aug-21	13-Sept-21	17-Sept-21		21-Jan-22
C19IMPACT_2003	Trinidad and Tobago	24-Aug-21	MoH	N	N	N	N	N	N	13.00		27-Aug-21	13-Sept-21	17-Sept-21		21-Jan-22
C19IMPACT_2004	Trinidad and Tobago	24-Aug-21	MoH	N	N	N	N	N	N	20.00		27-Aug-21	13-Sept-21	17-Sept-21		21-Jan-22
C19IMPACT_2005	Trinidad and Tobago	24-Aug-21	MoH	N	N	N	N	N	N	15.00		27-Aug-21	13-Sept-21	17-Sept-21		21-Jan-22
C19IMPACT_2006	Trinidad and Tobago	24-Aug-21	MoH	N	N	N	N	N	N	20.00		27-Aug-21	13-Sept-21	17-Sept-21		21-Jan-22
C19IMPACT_2007	Trinidad and Tobago	24-Aug-21	MoH	N	N	N	N	N	N	20.00		27-Aug-21	13-Sept-21	17-Sept-21		21-Jan-22
C19IMPACT_2008	Trinidad and Tobago	24-Aug-21	MoH	N	N	N	N	N	N	18.00		27-Aug-21	13-Sept-21	17-Sept-21		
C19IMPACT_2009	Trinidad and Tobago	24-Aug-21	MoH	N	N	N	N	N	N	15.00		27-Aug-21	13-Sept-21	17-Sept-21		21-Jan-22
C19IMPACT_2010	Trinidad and Tobago	24-Aug-21	MoH	N	N	N	N	N	N	17.00		27-Aug-21	13-Sept-21	17-Sept-21		21-Jan-22
C19IMPACT_2011	Trinidad and Tobago	24-Aug-21	MoH	N	N	N	N	N	N	19.00		27-Aug-21	13-Sept-21	17-Sept-21		21-Jan-22
C19IMPACT_2012	Trinidad and Tobago	24-Aug-21	MoH	N	N	N	N	N	N	19.00		27-Aug-21	13-Sept-21	17-Sept-21		21-Jan-22
C19IMPACT_2013	Trinidad and Tobago	24-Aug-21	MoH	N	N	N	N	N	N	24.00		27-Aug-21	13-Sept-21	17-Sept-21		21-Jan-22
C19IMPACT_2014	Trinidad and Tobago	24-Aug-21	MoH	N	N	N	N	N	N	20.00		27-Aug-21	13-Sept-21	17-Sept-21		21-Jan-22
C19IMPACT_2015	Trinidad and Tobago	24-Aug-21	MoH	N	N	N	N	N	N	17.00		27-Aug-21	13-Sept-21	17-Sept-21		21-Jan-22
C19IMPACT_2016	Trinidad and Tobago	24-Aug-21	MoH	N	N	N	N	N	N	15.00		30-Aug-21	13-Sept-21	17-Sept-21		
C19IMPACT_2017	Trinidad and Tobago	24-Aug-21	MoH	N	N	N	N	N	N	18.00		30-Aug-21	13-Sept-21	17-Sept-21		21-Jan-22
C19IMPACT_2018	Trinidad and Tobago	24-Aug-21	MoH	N	N	N	N	N	N	18.00		30-Aug-21	13-Sept-21	17-Sept-21		21-Jan-22
C19IMPACT_2019	Trinidad and Tobago	25-Aug-21	MoH	N	N	N	N	N	N	17.00		27-Aug-21	13-Sept-21	17-Sept-21		21-Jan-22
C19IMPACT_2020	Trinidad and Tobago	25-Aug-21	MoH	N	N	N	N	N	N	15.00		27-Aug-21	13-Sept-21	17-Sept-21		21-Jan-22
C19IMPACT_2021	Trinidad and Tobago	25-Aug-21	MoH	N	N	N	N	N	N	17.00		27-Aug-21	13-Sept-21	17-Sept-21		
C19IMPACT_2022	Trinidad and Tobago	25-Aug-21	MoH	N	N	N	N	N	N	19.00		27-Aug-21	13-Sept-21	17-Sept-21		21-Jan-22
C19IMPACT_2023	Trinidad and Tobago	25-Aug-21	MoH	N	N	N	N	N	N	20.00		27-Aug-21	13-Sept-21	17-Sept-21		21-Jan-22
C19IMPACT_2024	Trinidad and Tobago	25-Aug-21	MoH	N	N	N	N	N	N	20.00		27-Aug-21	13-Sept-21	17-Sept-21		21-Jan-22
C19IMPACT_2025	Trinidad and Tobago	25-Aug-21	MoH	N	N	N	N	N	N	18.00		27-Aug-21	13-Sept-21	17-Sept-21		21-Jan-22
C19IMPACT_2026	Trinidad and Tobago	25-Aug-21	MoH	N	N	N	N	N	N	16.00		27-Aug-21	13-Sept-21	17-Sept-21		21-Jan-22
C19IMPACT_2027	Trinidad and Tobago	25-Aug-21	MoH	N	N	N	N	N	N	16.00		27-Aug-21	13-Sept-21	17-Sept-21		21-Jan-22
C19IMPACT_2028	Trinidad and Tobago	25-Aug-21	MoH	N	N	N	N	N	N	20.00		27-Aug-21	13-Sept-21	17-Sept-21		21-Jan-22
C19IMPACT_2029	Trinidad and Tobago	25-Aug-21	MoH	N	N	N	N	N	N	17.00		27-Aug-21	13-Sept-21	17-Sept-21		21-Jan-22
C19IMPACT_2030	Trinidad and Tobago	25-Aug-21	MoH	N	N	N	N	N	N	17.00		27-Aug-21	13-Sept-21	17-Sept-21		21-Jan-22
C19IMPACT_2031	Trinidad and Tobago	25-Aug-21	MoH	N	N	N	N	N	N	16.00		27-Aug-21	13-Sept-21	17-Sept-21		21-Jan-22
C19IMPACT_2032	Trinidad and Tobago	25-Aug-21	MoH	N	N	N	N	N	N	17.00		27-Aug-21	13-Sept-21	17-Sept-21		21-Jan-22
C19IMPACT_2033	Trinidad and Tobago	25-Aug-21	MoH	N	N	N	N	N	N	18.00		27-Aug-21	13-Sept-21	17-Sept-21		21-Jan-22
C19IMPACT_2034	Trinidad and Tobago	25-Aug-21	MoH	N	N	N	N	N	N	18.00		27-Aug-21	13-Sept-21	17-Sept-21		21-Jan-22
C19IMPACT_2035	Trinidad and Tobago	25-Aug-21	MoH	N	N	N	N	N	N	19.00		27-Aug-21	13-Sept-21	17-Sept-21		21-Jan-22
C19IMPACT_2036	Trinidad and Tobago	25-Aug-21	MoH	N	N	N	N	N	N	20.00		27-Aug-21	13-Sept-21	17-Sept-21		21-Jan-22
C19IMPACT_2037	Trinidad and Tobago	25-Aug-21	MoH	N	N	N	N	N	N	13.00		27-Aug-21	13-Sept-21	17-Sept-21		21-Jan-22
C19IMPACT_2038	Trinidad and Tobago	25-Aug-21	MoH	N	N	N	N	N	N	19.00		27-Aug-21	13-Sept-21	17-Sept-21		21-Jan-22
C19IMPACT_2039	Trinidad and Tobago	25-Aug-21	MoH	N	N	N	N	N	N	19.00		27-Aug-21	13-Sept-21	17-Sept-21		21-Jan-22
C19IMPACT_2040	Trinidad and Tobago	25-Aug-21	MoH	N	N	N	N	N	N	19.00		27-Aug-21	13-Sept-21	17-Sept-21		21-Jan-22
C19IMPACT_2041	Trinidad and Tobago	25-Aug-21	MoH	N	N	N	N	N	N	13.00		30-Aug-21	13-Sept-21	17-Sept-21		21-Jan-22
C19IMPACT_2042	Trinidad and Tobago	25-Aug-21	MoH	N	N	N	N	N	N	20.00		27-Aug-21	13-Sept-21	17-Sept-21		21-Jan-22
C19IMPACT_2043	Trinidad and Tobago	26-Aug-21	MoH	N	N	N	N	N	N	19.00		30-Aug-21	13-Sept-21	17-Sept-21		21-Jan-22
C19IMPACT_2044	Trinidad and Tobago	26-Aug-21	MoH	N	N	N	N	N	N	18.00		30-Aug-21	13-Sept-21	17-Sept-21		21-Jan-22

C19IMPACT_2045	Trinidad and Tobago	26-Aug-21	MoH	N	N	N	N	N	N	17.00		30-Aug-21	13-Sept-21	17-Sept-21		21-Jan-22
C19IMPACT_2046	Trinidad and Tobago	26-Aug-21	MoH	N	N	N	N	N	N	18.00		30-Aug-21	13-Sept-21	17-Sept-21		21-Jan-22
C19IMPACT_2047	Trinidad and Tobago	26-Aug-21	MoH	N	N	N	N	N	N	19.00		30-Aug-21	13-Sept-21	17-Sept-21		21-Jan-22
C19IMPACT_2048	Trinidad and Tobago	26-Aug-21	MoH	N	N	N	N	N	N	19.00		30-Aug-21	13-Sept-21	17-Sept-21		21-Jan-22
C19IMPACT_2049	Trinidad and Tobago	26-Aug-21	MoH	N	N	N	N	N	N	19.00		30-Aug-21	13-Sept-21	17-Sept-21		21-Jan-22
C19IMPACT_2050	Trinidad and Tobago	26-Aug-21	MoH	N	N	N	N	N	N	17.00		30-Aug-21	13-Sept-21	17-Sept-21		21-Jan-22
C19IMPACT_2051	Trinidad and Tobago	26-Aug-21	MoH	N	N	N	N	N	N	18.00		30-Aug-21	13-Sept-21	17-Sept-21		21-Jan-22
C19IMPACT_2052	Trinidad and Tobago	26-Aug-21	MoH	N	N	N	N	N	N	17.00		30-Aug-21	13-Sept-21	17-Sept-21		21-Jan-22
C19IMPACT_2053	Trinidad and Tobago	26-Aug-21	MoH	N	N	N	N	N	N	16.00		30-Aug-21	13-Sept-21	17-Sept-21		21-Jan-22
C19IMPACT_2054	Trinidad and Tobago	26-Aug-21	MoH	N	N	N	N	N	N	19.00		30-Aug-21	13-Sept-21	17-Sept-21		21-Jan-22
C19IMPACT_2055	Trinidad and Tobago	26-Aug-21	MoH	N	N	N	N	N	N	18.00		30-Aug-21	13-Sept-21	17-Sept-21		21-Jan-22
C19IMPACT_2056	Trinidad and Tobago	26-Aug-21	MoH	N	N	N	N	N	N	18.00		30-Aug-21	13-Sept-21	17-Sept-21		21-Jan-22
C19IMPACT_2057	Trinidad and Tobago	27-Aug-21	MoH	N	N	N	N	N	N	16.00		30-Aug-21	13-Sept-21	17-Sept-21		21-Jan-22
C19IMPACT_2058	Trinidad and Tobago	27-Aug-21	MoH	N	N	N	N	N	N	18.00		30-Aug-21	13-Sept-21	17-Sept-21		21-Jan-22
C19IMPACT_2059	Trinidad and Tobago	27-Aug-21	MoH	N	N	N	N	N	N	19.00		30-Aug-21	13-Sept-21	17-Sept-21		21-Jan-22
C19IMPACT_2060	Trinidad and Tobago		MoH	N	N	N	N	N	N			27-Aug-21	13-Sept-21	13-Oct-21		
C19IMPACT_2061	British Virgin Islands	02-Aug-21	CARPHA	N	N	N	N	N	N	16.09		02-Sept-21	16-Sept-21		16-Nov-21	21-Jan-22
C19IMPACT_2062	British Virgin Islands	03-Aug-21	CARPHA	N	N	N	N	N	N	19.96		02-Sept-21	16-Sept-21		16-Nov-21	21-Jan-22
C19IMPACT_2063	British Virgin Islands	03-Aug-21	CARPHA	N	N	N	N	N	N	19.90		02-Sept-21	16-Sept-21		16-Nov-21	21-Jan-22
C19IMPACT_2064	British Virgin Islands	03-Aug-21	CARPHA	N	N	N	N	N	N	22.84		02-Sept-21	16-Sept-21		16-Nov-21	21-Jan-22
C19IMPACT_2065	British Virgin Islands	04-Aug-21	CARPHA	N	N	N	N	N	N	13.98		02-Sept-21	16-Sept-21		16-Nov-21	21-Jan-22
C19IMPACT_2066	British Virgin Islands	05-Aug-21	CARPHA	N	N	N	N	N	N	13.65		02-Sept-21	16-Sept-21		16-Nov-21	21-Jan-22
C19IMPACT_2067	British Virgin Islands	06-Aug-21	CARPHA	N	N	N	N	N	N	18.35		02-Sept-21	16-Sept-21		16-Nov-21	21-Jan-22
C19IMPACT_2068	British Virgin Islands	08-Aug-21	CARPHA	N	N	N	N	N	N	20.36		02-Sept-21	16-Sept-21		16-Nov-21	21-Jan-22
C19IMPACT_2069	Trinidad and Tobago	21-Aug-21	MoH	N	N	N	N	N	N			02-Sept-21	16-Sept-21	19-Nov-21		21-Jan-22
C19IMPACT_2070	Trinidad and Tobago	22-Aug-21	MoH	N	N	N	N	N	N			02-Sept-21	16-Sept-21	19-Nov-21		21-Jan-22
C19IMPACT_2071	Trinidad and Tobago	24-Aug-21	CARPHA	N	N	N	N	N	N	23.12		02-Sept-21	16-Sept-21	19-Nov-21	16-Nov-21	21-Jan-22
C19IMPACT_2072	Saint Vincent and the Grenadines	24-Aug-21	CARPHA	N	N	N	N	N	N	17.55		02-Sept-21	16-Sept-21		16-Nov-21	21-Jan-22
C19IMPACT_2073	Saint Vincent and the Grenadines	24-Aug-21	CARPHA	N	N	N	N	N	N	20.76		02-Sept-21	16-Sept-21		16-Nov-21	21-Jan-22
C19IMPACT_2074	Saint Vincent and the Grenadines	24-Aug-21	CARPHA	N	N	N	N	N	N	26.30		02-Sept-21	16-Sept-21		16-Nov-21	
C19IMPACT_2075	Trinidad and Tobago	25-Aug-21	CARPHA	N	N	N	N	N	N	15.48		02-Sept-21	16-Sept-21	19-Nov-21	16-Nov-21	21-Jan-22
C19IMPACT_2076	Trinidad and Tobago	25-Aug-21	CARPHA	N	N	N	N	N	N	13.14		02-Sept-21	16-Sept-21	19-Nov-21	16-Nov-21	21-Jan-22
C19IMPACT_2077	British Virgin Islands	25-Aug-21	CARPHA	N	N	N	N	N	N	16.88		02-Sept-21	16-Sept-21		16-Nov-21	21-Jan-22
C19IMPACT_2078	British Virgin Islands	25-Aug-21	CARPHA	N	N	N	N	N	N	16.58		02-Sept-21	16-Sept-21		16-Nov-21	21-Jan-22
C19IMPACT_2079	Saint Vincent and the Grenadines	25-Aug-21	CARPHA	N	N	N	N	N	N	12.70		02-Sept-21	16-Sept-21		16-Nov-21	21-Jan-22
C19IMPACT_2080	Trinidad and Tobago	27-Aug-21	MoH	N	N	N	N	N	N	15.00		30-Aug-21	16-Sept-21	19-Nov-21		21-Jan-22
C19IMPACT_2081	Trinidad and Tobago	27-Aug-21	MoH	N	N	N	N	N	N	19.00		30-Aug-21	16-Sept-21	19-Nov-21		21-Jan-22
C19IMPACT_2082	Trinidad and Tobago	27-Aug-21	MoH	N	N	N	N	N	N	16.00		30-Aug-21	16-Sept-21	19-Nov-21		
C19IMPACT_2083	Trinidad and Tobago	27-Aug-21	MoH	N	N	N	N	N	N	20.00		30-Aug-21	16-Sept-21	19-Nov-21		21-Jan-22
C19IMPACT_2084	Trinidad and Tobago	28-Aug-21	MoH	N	N	N	N	N	N	19.00		30-Aug-21	16-Sept-21	19-Nov-21		21-Jan-22
C19IMPACT_2085	Trinidad and Tobago	28-Aug-21	MoH	N	N	N	N	N	N	19.00		30-Aug-21	16-Sept-21	19-Nov-21		21-Jan-22
C19IMPACT_2086	Trinidad and Tobago	28-Aug-21	MoH	N	N	N	N	N	N	17.00		30-Aug-21	16-Sept-21	19-Nov-21		21-Jan-22
C19IMPACT_2087	Trinidad and Tobago	29-Aug-21	MoH	N	N	N	N	N	N	19.00		30-Aug-21	16-Sept-21	19-Nov-21		21-Jan-22
C19IMPACT_2088	Trinidad and Tobago	29-Aug-21	MoH	N	N	N	N	N	N	18.00		30-Aug-21	16-Sept-21	19-Nov-21		21-Jan-22
C19IMPACT_2089	Trinidad and Tobago	29-Aug-21	MoH	N	N	N	N	N	N	18.00		30-Aug-21	16-Sept-21	19-Nov-21		21-Jan-22
C19IMPACT_2090	Trinidad and Tobago	30-Aug-21	MoH	N	N	N	N	N	N	17.00		01-Sept-21	16-Sept-21	19-Nov-21		
C19IMPACT_2091	Trinidad and Tobago	30-Aug-21	MoH	N	N	N	N	N	N	16.00		01-Sept-21	16-Sept-21	19-Nov-21		
C19IMPACT_2092	Trinidad and Tobago	30-Aug-21	MoH	N	N	N	N	N	N	19.00		01-Sept-21	16-Sept-21	19-Nov-21		21-Jan-22
C19IMPACT_2093	Trinidad and Tobago	30-Aug-21	MoH	N	N	N	N	N	N	20.00		01-Sept-21	16-Sept-21	19-Nov-21		21-Jan-22
C19IMPACT_2094	Trinidad and Tobago	30-Aug-21	MoH	N	N	N	N	N	N	20.00		01-Sept-21	16-Sept-21	19-Nov-21		21-Jan-22
C19IMPACT_2095	Trinidad and Tobago	30-Aug-21	MoH	N	N	N	N	N	N	18.00		01-Sept-21	16-Sept-21	19-Nov-21		21-Jan-22
C19IMPACT_2096	Trinidad and Tobago	30-Aug-21	MoH	N	N	N	N	N	N	17.00		01-Sept-21	16-Sept-21	19-Nov-21		21-Jan-22
C19IMPACT_2097	Trinidad and Tobago	30-Aug-21	MoH	N	N	N	N	N	N	19.00		01-Sept-21	16-Sept-21	19-Nov-21		21-Jan-22
C19IMPACT_2098	Trinidad and Tobago	30-Aug-21	MoH	N	N	N	N	N	N	20.00		01-Sept-21	16-Sept-21	19-Nov-21		21-Jan-22
C19IMPACT_2099	Trinidad and Tobago	01-Sept-21	MoH	N	N	N	N	N	N	17.60		02-Sept-21	16-Sept-21	19-Nov-21		
C19IMPACT_2100	Trinidad and Tobago	01-Sept-21	MoH	N	N	N	N	N	N	16.30		02-Sept-21	16-Sept-21	19-Nov-21		
C19IMPACT_2101	Trinidad and Tobago	01-Sept-21	MoH	N	N	N	N	N	N	16.40		02-Sept-21	16-Sept-21	19-Nov-21		
C19IMPACT_2102	Trinidad and Tobago	01-Sept-21	MoH	N	N	N	N	N	N	14.10		02-Sept-21	16-Sept-21	19-Nov-21		
C19IMPACT_2103	Trinidad and Tobago	01-Sept-21	MoH	N	N	N	N	N	N	16.00		03-Sept-21	16-Sept-21	19-Nov-21		21-Jan-22
C19IMPACT_2104	Trinidad and Tobago	01-Sept-21	MoH	N	N	N	N	N	N	19.00		03-Sept-21	16-Sept-21	19-Nov-21		21-Jan-22
C19IMPACT_2105	Trinidad and Tobago	26-Aug-21	MoH	N	N	N	N	N	N			03-Sept-21	17-Sept-21	25-Sept-21		21-Jan-22
C19IMPACT_2106	Trinidad and Tobago	26-Aug-21	MoH	N	N	N	N	N	Y		9.33	03-Sept-21	17-Sept-21	25-Sept-21		21-Jan-22
C19IMPACT_2107	Trinidad and Tobago	26-Aug-21	MoH	N	N	N	N	N	Y		5.44	03-Sept-21	17-Sept-21	25-Sept-21		21-Jan-22
C19IMPACT_2108	Trinidad and Tobago	26-Aug-21	MoH	N	N	N	N	N	Y		14.81	03-Sept-21	17-Sept-21	25-Sept-21		

C19IMPACT_2109	Trinidad and Tobago	27-Aug-21	MoH	N	N	N	N	N	Y		14.16	03-Sept-21	17-Sept-21	25-Sept-21		
C19IMPACT_2110	Trinidad and Tobago	28-Aug-21	MoH	N	N	N	N	N	Y		13.90	03-Sept-21	17-Sept-21	25-Sept-21		21-Jan-22
C19IMPACT_2111	Trinidad and Tobago	28-Aug-21	MoH	N	N	N	N	N	Y		15.37	03-Sept-21	17-Sept-21	25-Sept-21		
C19IMPACT_2112	Trinidad and Tobago	29-Aug-21	MoH	N	N	N	N	N	Y		4.39	03-Sept-21	17-Sept-21	25-Sept-21		
C19IMPACT_2113	Trinidad and Tobago	29-Aug-21	MoH	N	N	N	N	N	Y		5.60	03-Sept-21	17-Sept-21	25-Sept-21		21-Jan-22
C19IMPACT_2114	Trinidad and Tobago	29-Aug-21	MoH	N	N	N	N	N	Y		10.73	03-Sept-21	17-Sept-21	25-Sept-21		21-Jan-22
C19IMPACT_2115	Trinidad and Tobago	30-Aug-21	MoH	N	N	N	N	N	N	12.00		07-Sept-21	17-Sept-21	25-Sept-21		21-Jan-22
C19IMPACT_2116	Trinidad and Tobago	30-Aug-21	MoH	N	N	N	N	N	Y		5.42	07-Sept-21	17-Sept-21	25-Sept-21		21-Jan-22
C19IMPACT_2117	Trinidad and Tobago	30-Aug-21	MoH	N	N	N	N	N	Y		4.63	07-Sept-21	17-Sept-21	25-Sept-21		21-Jan-22
C19IMPACT_2118	Trinidad and Tobago	30-Aug-21	MoH	N	N	N	N	N	Y		10.27	03-Sept-21	17-Sept-21	25-Sept-21		21-Jan-22
C19IMPACT_2119	Trinidad and Tobago	30-Aug-21	MoH	N	N	N	N	N	N	19.00		07-Sept-21	17-Sept-21	25-Sept-21		
C19IMPACT_2120	Trinidad and Tobago	30-Aug-21	MoH	N	N	N	N	N	N	27.00		07-Sept-21	17-Sept-21	25-Sept-21		
C19IMPACT_2121	Trinidad and Tobago	01-Sept-21	MoH	N	N	N	N	N	Y		7.80	07-Sept-21	17-Sept-21	25-Sept-21		21-Jan-22
C19IMPACT_2122	Trinidad and Tobago	01-Sept-21	MoH	N	N	N	N	N	Y		8.91	07-Sept-21	17-Sept-21	25-Sept-21		21-Jan-22
C19IMPACT_2123	Trinidad and Tobago	01-Sept-21	MoH	N	N	N	N	N	N	14.00		07-Sept-21	17-Sept-21	25-Sept-21		
C19IMPACT_2124	Trinidad and Tobago	01-Sept-21	MoH	N	N	N	N	N	N	14.60		07-Sept-21	17-Sept-21	25-Sept-21		
C19IMPACT_2125	Trinidad and Tobago	01-Sept-21	MoH	N	N	N	N	N	N	16.00		03-Sept-21	17-Sept-21	25-Sept-21		21-Jan-22
C19IMPACT_2126	Trinidad and Tobago	01-Sept-21	MoH	N	N	N	N	N	N	19.00		03-Sept-21	17-Sept-21	25-Sept-21		21-Jan-22
C19IMPACT_2127	Trinidad and Tobago	01-Sept-21	MoH	N	N	N	N	N	N	17.00		03-Sept-21	17-Sept-21	25-Sept-21		21-Jan-22
C19IMPACT_2128	Trinidad and Tobago	01-Sept-21	MoH	N	N	N	N	N	N	20.00		03-Sept-21	17-Sept-21	25-Sept-21		21-Jan-22
C19IMPACT_2129	Trinidad and Tobago	01-Sept-21	MoH	N	N	N	N	N	N	20.00		03-Sept-21	17-Sept-21	25-Sept-21		21-Jan-22
C19IMPACT_2130	Trinidad and Tobago	01-Sept-21	MoH	N	N	N	N	N	N	19.00		03-Sept-21	17-Sept-21	25-Sept-21		21-Jan-22
C19IMPACT_2131	Trinidad and Tobago	01-Sept-21	MoH	N	N	N	N	N	N	17.00		03-Sept-21	17-Sept-21	25-Sept-21		21-Jan-22
C19IMPACT_2132	Trinidad and Tobago	01-Sept-21	MoH	N	N	N	N	N	N	17.00		03-Sept-21	17-Sept-21	25-Sept-21		21-Jan-22
C19IMPACT_2133	Trinidad and Tobago	01-Sept-21	MoH	N	N	N	N	N	N	19.00		03-Sept-21	17-Sept-21	25-Sept-21		21-Jan-22
C19IMPACT_2134	Trinidad and Tobago	01-Sept-21	MoH	N	N	N	N	N	Y	22.00		03-Sept-21	17-Sept-21	25-Sept-21		
C19IMPACT_2135	Trinidad and Tobago	02-Sept-21	MoH	N	N	N	N	N	N	14.20		07-Sept-21	17-Sept-21	25-Sept-21		
C19IMPACT_2136	Trinidad and Tobago	02-Sept-21	MoH	N	N	N	N	N	N	14.20		07-Sept-21	17-Sept-21	25-Sept-21		
C19IMPACT_2137	Trinidad and Tobago	02-Sept-21	MoH	N	N	N	N	N	Y		8.07	07-Sept-21	17-Sept-21	25-Sept-21		21-Jan-22
C19IMPACT_2138	Trinidad and Tobago	04-Sept-21	MoH	N	N	N	N	N	N	19.00		07-Sept-21	17-Sept-21	25-Sept-21		21-Jan-22
C19IMPACT_2139	Trinidad and Tobago	04-Sept-21	MoH	N	N	N	N	N	N	17.00		07-Sept-21	17-Sept-21	25-Sept-21		21-Jan-22
C19IMPACT_2140	Trinidad and Tobago	04-Sept-21	MoH	N	N	N	N	N	N	20.00		07-Sept-21	17-Sept-21	25-Sept-21		21-Jan-22
C19IMPACT_2141	Trinidad and Tobago	04-Sept-21	MoH	N	N	N	N	N	N	18.00		07-Sept-21	17-Sept-21	25-Sept-21		21-Jan-22
C19IMPACT_2142	Trinidad and Tobago	04-Sept-21	MoH	N	N	N	N	N	N	19.00		07-Sept-21	17-Sept-21	25-Sept-21		21-Jan-22
C19IMPACT_2143	Trinidad and Tobago	04-Sept-21	MoH	N	N	N	N	N	N	19.00		07-Sept-21	17-Sept-21	25-Sept-21		21-Jan-22
C19IMPACT_2144	Trinidad and Tobago	04-Sept-21	MoH	N	N	N	N	N	N	20.00		07-Sept-21	17-Sept-21	25-Sept-21		21-Jan-22
C19IMPACT_2145	Trinidad and Tobago	04-Sept-21	MoH	N	N	N	N	N	N	16.00		07-Sept-21	17-Sept-21	25-Sept-21		21-Jan-22
C19IMPACT_2146	Trinidad and Tobago	05-Sept-21	MoH	N	N	N	N	N	N	18.00		07-Sept-21	17-Sept-21	25-Sept-21		21-Jan-22
C19IMPACT_2147	Trinidad and Tobago	12-Sept-21	MoH	N	N	N	N	N	N	15.00		15-Sept-21	17-Sept-21	25-Sept-21		
C19IMPACT_2148	Trinidad and Tobago	12-Sept-21	MoH	N	N	N	N	N	N	17.00		15-Sept-21	17-Sept-21	25-Sept-21		
C19IMPACT_2149	Turks and Caicos Islands	05-Jul-21	CARPHA	N	N	N	N	N	N	20.87		08-Sept-21	22-Sept-21		25-Sept-21	
C19IMPACT_2150	Turks and Caicos Islands	05-Jul-21	CARPHA	N	N	N	N	N	N	21.86		08-Sept-21	22-Sept-21		25-Sept-21	21-Jan-22
C19IMPACT_2151	Turks and Caicos Islands	05-Jul-21	CARPHA	N	N	N	N	N	N	20.24		08-Sept-21	22-Sept-21		25-Sept-21	
C19IMPACT_2152	Turks and Caicos Islands	05-Jul-21	CARPHA	N	N	N	N	N	N	20.66		08-Sept-21	22-Sept-21		25-Sept-21	21-Jan-22
C19IMPACT_2153	Turks and Caicos Islands	20-Jul-21	CARPHA	N	N	N	N	N	N	19.62		08-Sept-21	22-Sept-21		25-Sept-21	21-Jan-22
C19IMPACT_2154	Barbados	31-Jul-21	CARPHA	N	N	N	N	N	N	25.03		08-Sept-21	22-Sept-21		25-Sept-21	
C19IMPACT_2155	Barbados	03-Aug-21	CARPHA	N	N	N	N	N	N	22.49		08-Sept-21	22-Sept-21		25-Sept-21	
C19IMPACT_2156	Barbados	03-Aug-21	CARPHA	N	N	N	N	N	N	14.55		08-Sept-21	22-Sept-21		25-Sept-21	21-Jan-22
C19IMPACT_2157	Barbados	03-Aug-21	CARPHA	N	N	N	N	N	N	24.53		08-Sept-21	22-Sept-21		25-Sept-21	
C19IMPACT_2158	Trinidad and Tobago	04-Aug-21	CARPHA	N	N	N	N	N	N	20.20		08-Sept-21	22-Sept-21	25-Sept-21	25-Sept-21	21-Jan-22
C19IMPACT_2159	Trinidad and Tobago	04-Aug-21	CARPHA	N	N	N	N	N	N	23.62		08-Sept-21	22-Sept-21	25-Sept-21	25-Sept-21	21-Jan-22
C19IMPACT_2160	Barbados	04-Aug-21	CARPHA	N	N	N	N	N	N	19.15		08-Sept-21	22-Sept-21		25-Sept-21	21-Jan-22
C19IMPACT_2161	Trinidad and Tobago	05-Aug-21	CARPHA	N	N	N	N	N	N	20.93		08-Sept-21	22-Sept-21	25-Sept-21	25-Sept-21	21-Jan-22
C19IMPACT_2162	Saint Lucia	06-Aug-21	CARPHA	N	N	N	N	N	N	9.42		08-Sept-21	22-Sept-21		25-Sept-21	21-Jan-22
C19IMPACT_2163	Trinidad and Tobago	07-Aug-21	CARPHA	N	N	N	N	N	N	17.10		08-Sept-21	22-Sept-21	25-Sept-21	25-Sept-21	21-Jan-22
C19IMPACT_2164	Saint Lucia	07-Aug-21	CARPHA	N	N	N	N	N	N	18.52		08-Sept-21	22-Sept-21		25-Sept-21	21-Jan-22
C19IMPACT_2165	Trinidad and Tobago	09-Aug-21	CARPHA	N	N	N	N	N	N	18.70		08-Sept-21	22-Sept-21	25-Sept-21	25-Sept-21	21-Jan-22
C19IMPACT_2166	Trinidad and Tobago	09-Aug-21	CARPHA	N	N	N	N	N	N	15.60		08-Sept-21	22-Sept-21	25-Sept-21	25-Sept-21	
C19IMPACT_2167	Trinidad and Tobago	09-Aug-21	CARPHA	N	N	N	N	N	N	22.35		08-Sept-21	22-Sept-21	25-Sept-21	25-Sept-21	
C19IMPACT_2168	Trinidad and Tobago	09-Aug-21	CARPHA	N	N	N	N	N	N	22.40		08-Sept-21	22-Sept-21	25-Sept-21	25-Sept-21	
C19IMPACT_2169	Trinidad and Tobago	09-Aug-21	CARPHA	N	N	N	N	N	N	22.98		08-Sept-21	22-Sept-21	25-Sept-21	25-Sept-21	21-Jan-22
C19IMPACT_2170	Trinidad and Tobago	09-Aug-21	CARPHA	N	N	N	N	N	N	23.09		08-Sept-21	22-Sept-21	25-Sept-21	25-Sept-21	21-Jan-22
C19IMPACT_2171	Trinidad and Tobago	09-Aug-21	CARPHA	N	N	N	N	N	N	21.36		08-Sept-21	22-Sept-21	25-Sept-21	25-Sept-21	
C19IMPACT_2172	Trinidad and Tobago	09-Aug-21	CARPHA	N	N	N	N	N	N	20.78		08-Sept-21	22-Sept-21	25-Sept-21	25-Sept-21	21-Jan-22

C19IMPACT_2173	Trinidad and Tobago	12-Aug-21	MoH	N	N	N	N	N	Y		21.00	08-Sept-21	22-Sept-21	25-Sept-21		21-Jan-22
C19IMPACT_2174	Saint Lucia	12-Aug-21	CARPHA	N	N	N	N	N	N	20.22		08-Sept-21	22-Sept-21		25-Sept-21	21-Jan-22
C19IMPACT_2175	Trinidad and Tobago	13-Aug-21	CARPHA	N	N	N	N	N	N	17.50		08-Sept-21	22-Sept-21	25-Sept-21	25-Sept-21	21-Jan-22
C19IMPACT_2176	Saint Lucia	14-Aug-21	CARPHA	N	N	N	N	N	N	10.15		08-Sept-21	22-Sept-21		25-Sept-21	21-Jan-22
C19IMPACT_2177	Trinidad and Tobago	15-Aug-21	CARPHA	N	N	N	N	N	N	15.38		08-Sept-21	22-Sept-21	25-Sept-21	25-Sept-21	21-Jan-22
C19IMPACT_2178	Trinidad and Tobago	15-Aug-21	CARPHA	N	N	N	N	N	N	25.45		08-Sept-21	22-Sept-21	25-Sept-21	25-Sept-21	
C19IMPACT_2179	Trinidad and Tobago	15-Aug-21	CARPHA	N	N	N	N	N	N	18.56		08-Sept-21	22-Sept-21	25-Sept-21	25-Sept-21	21-Jan-22
C19IMPACT_2180	Trinidad and Tobago	16-Aug-21	CARPHA	N	N	N	N	N	N	19.33		08-Sept-21	22-Sept-21	25-Sept-21	25-Sept-21	21-Jan-22
C19IMPACT_2181	Trinidad and Tobago	16-Aug-21	CARPHA	N	N	N	N	N	N	22.14		08-Sept-21	22-Sept-21	25-Sept-21	25-Sept-21	
C19IMPACT_2182	Trinidad and Tobago	16-Aug-21	CARPHA	N	N	N	N	N	N	18.63		08-Sept-21	22-Sept-21	25-Sept-21	25-Sept-21	
C19IMPACT_2183	Trinidad and Tobago	16-Aug-21	CARPHA	N	N	N	N	N	N	24.80		08-Sept-21	22-Sept-21	25-Sept-21	25-Sept-21	21-Jan-22
C19IMPACT_2184	Trinidad and Tobago	16-Aug-21	CARPHA	N	N	N	N	N	N	18.95		08-Sept-21	22-Sept-21	25-Sept-21	25-Sept-21	21-Jan-22
C19IMPACT_2185	Trinidad and Tobago	17-Aug-21	CARPHA	N	N	N	N	N	N	18.68		08-Sept-21	22-Sept-21	25-Sept-21	25-Sept-21	
C19IMPACT_2186	Saint Lucia	17-Aug-21	CARPHA	N	N	N	N	N	N	23.80		08-Sept-21	22-Sept-21		25-Sept-21	
C19IMPACT_2187	Trinidad and Tobago	18-Aug-21	CARPHA	N	N	N	N	N	N	15.11		08-Sept-21	22-Sept-21	25-Sept-21	25-Sept-21	21-Jan-22
C19IMPACT_2188	Trinidad and Tobago	18-Aug-21	CARPHA	N	N	N	N	N	N	23.54		08-Sept-21	22-Sept-21	25-Sept-21	25-Sept-21	21-Jan-22
C19IMPACT_2189	Trinidad and Tobago	18-Aug-21	CARPHA	N	N	N	N	N	N	18.34		08-Sept-21	22-Sept-21	25-Sept-21	25-Sept-21	21-Jan-22
C19IMPACT_2190	Trinidad and Tobago	18-Aug-21	CARPHA	N	N	N	N	N	N	17.27		08-Sept-21	22-Sept-21	25-Sept-21	25-Sept-21	21-Jan-22
C19IMPACT_2191	Trinidad and Tobago	18-Aug-21	CARPHA	N	N	N	N	N	N	22.80		08-Sept-21	22-Sept-21	25-Sept-21	25-Sept-21	21-Jan-22
C19IMPACT_2192	Trinidad and Tobago	18-Aug-21	CARPHA	N	N	N	N	N	N	20.15		08-Sept-21	22-Sept-21	25-Sept-21	25-Sept-21	21-Jan-22
C19IMPACT_2193	Trinidad and Tobago	18-Aug-21	CARPHA	N	N	N	N	N	N	23.23		08-Sept-21	22-Sept-21	25-Sept-21	25-Sept-21	21-Jan-22
C19IMPACT_2194	Saint Lucia	18-Aug-21	CARPHA	N	N	N	N	N	N	11.64		08-Sept-21	22-Sept-21		25-Sept-21	21-Jan-22
C19IMPACT_2195	Saint Lucia	18-Aug-21	CARPHA	N	N	N	N	N	N	15.58		08-Sept-21	22-Sept-21		25-Sept-21	21-Jan-22
C19IMPACT_2196	Saint Lucia	18-Aug-21	CARPHA	N	N	N	N	N	N	17.05		08-Sept-21	22-Sept-21		25-Sept-21	21-Jan-22
C19IMPACT_2197	Anguilla	18-Aug-21	CARPHA	N	N	N	N	N	N	11.75		08-Sept-21	22-Sept-21		25-Sept-21	21-Jan-22
C19IMPACT_2198	Saint Lucia	19-Aug-21	CARPHA	N	N	N	N	N	N	22.63		08-Sept-21	22-Sept-21		25-Sept-21	21-Jan-22
C19IMPACT_2199	Saint Lucia	19-Aug-21	CARPHA	N	N	N	N	N	N	22.23		08-Sept-21	22-Sept-21		25-Sept-21	21-Jan-22
C19IMPACT_2200	Anguilla	19-Aug-21	CARPHA	N	N	N	N	N	N	11.53		08-Sept-21	22-Sept-21		25-Sept-21	21-Jan-22
C19IMPACT_2201	Anguilla	23-Aug-21	CARPHA	N	N	N	N	N	N	13.77		08-Sept-21	22-Sept-21		25-Sept-21	21-Jan-22
C19IMPACT_2202	Anguilla	24-Aug-21	CARPHA	N	N	N	N	N	N	15.29		08-Sept-21	22-Sept-21		25-Sept-21	21-Jan-22
C19IMPACT_2203	Anguilla	24-Aug-21	CARPHA	N	N	N	N	N	N	19.72		08-Sept-21	22-Sept-21		25-Sept-21	21-Jan-22
C19IMPACT_2204	Anguilla	24-Aug-21	CARPHA	N	N	N	N	N	N	12.47		08-Sept-21	22-Sept-21		25-Sept-21	21-Jan-22
C19IMPACT_2205	Trinidad and Tobago	25-Aug-21	MoH	N	N	N	N	N	N		20.00	08-Sept-21	22-Sept-21	25-Sept-21		21-Jan-22
C19IMPACT_2206	Trinidad and Tobago	27-Aug-21	MoH	N	N	N	N	N	N		4.00	08-Sept-21	22-Sept-21	25-Sept-21		21-Jan-22
C19IMPACT_2207	Trinidad and Tobago	27-Aug-21	MoH	N	N	N	N	N	N		8.00	08-Sept-21	22-Sept-21	25-Sept-21		21-Jan-22
C19IMPACT_2208	British Virgin Islands	27-Aug-21	CARPHA	N	N	N	N	N	N	11.87		08-Sept-21	22-Sept-21		25-Sept-21	21-Jan-22
C19IMPACT_2209	Anguilla	27-Aug-21	CARPHA	N	N	N	N	N	N	16.19		08-Sept-21	22-Sept-21		25-Sept-21	21-Jan-22
C19IMPACT_2210	British Virgin Islands	27-Aug-21	CARPHA	N	N	N	N	N	N	14.29		08-Sept-21	22-Sept-21		25-Sept-21	21-Jan-22
C19IMPACT_2211	Trinidad and Tobago	28-Aug-21	MoH	N	N	N	N	N	N		6.00	08-Sept-21	22-Sept-21	25-Sept-21		21-Jan-22
C19IMPACT_2212	Trinidad and Tobago	29-Aug-21	MoH	N	N	N	N	N	N		4.00	08-Sept-21	22-Sept-21	25-Sept-21		21-Jan-22
C19IMPACT_2213	Saint Vincent and the Grenadines	30-Aug-21	CARPHA	N	N	N	N	N	N	15.11		08-Sept-21	22-Sept-21		25-Sept-21	21-Jan-22
C19IMPACT_2214	Saint Vincent and the Grenadines	30-Aug-21	CARPHA	N	N	N	N	N	N	15.76		08-Sept-21	22-Sept-21		25-Sept-21	21-Jan-22
C19IMPACT_2215	Anguilla	30-Aug-21	CARPHA	N	N	N	N	N	N	14.64		08-Sept-21	22-Sept-21		25-Sept-21	21-Jan-22
C19IMPACT_2216	Anguilla	30-Aug-21	CARPHA	N	N	N	N	N	N	19.51		08-Sept-21	22-Sept-21		25-Sept-21	21-Jan-22
C19IMPACT_2217	British Virgin Islands	30-Aug-21	CARPHA	N	N	N	N	N	N	12.58		08-Sept-21	22-Sept-21		25-Sept-21	21-Jan-22
C19IMPACT_2218	Anguilla	30-Aug-21	CARPHA	N	N	N	N	N	N	23.34		08-Sept-21	22-Sept-21		25-Sept-21	21-Jan-22
C19IMPACT_2219	British Virgin Islands	30-Aug-21	CARPHA	N	N	N	N	N	N	17.60		08-Sept-21	22-Sept-21		25-Sept-21	21-Jan-22
C19IMPACT_2220	British Virgin Islands	31-Aug-21	CARPHA	N	N	N	N	N	N	23.09		08-Sept-21	22-Sept-21		25-Sept-21	21-Jan-22
C19IMPACT_2221	Trinidad and Tobago	01-Sept-21	MoH	N	N	N	N	N	N	19.40		08-Sept-21	22-Sept-21	25-Sept-21		
C19IMPACT_2222	Trinidad and Tobago	01-Sept-21	MoH	N	N	N	N	N	N	14.60		08-Sept-21	22-Sept-21	25-Sept-21		
C19IMPACT_2223	Trinidad and Tobago	01-Sept-21	MoH	N	N	N	N	N	N	14.20		08-Sept-21	22-Sept-21	25-Sept-21		
C19IMPACT_2224	Trinidad and Tobago	01-Sept-21	MoH	N	N	N	N	N	N	19.00		08-Sept-21	22-Sept-21	25-Sept-21		
C19IMPACT_2225	Trinidad and Tobago	02-Sept-21	MoH	N	N	N	N	N	Y	16.00		08-Sept-21	22-Sept-21	25-Sept-21		21-Jan-22
C19IMPACT_2226	Trinidad and Tobago	02-Sept-21	MoH	N	N	N	N	N	Y	18.00		08-Sept-21	22-Sept-21	25-Sept-21		21-Jan-22
C19IMPACT_2227	Trinidad and Tobago	02-Sept-21	MoH	N	N	N	N	N	N		14.00	08-Sept-21	22-Sept-21	14-Jan-22		21-Jan-22
C19IMPACT_2228	Trinidad and Tobago	03-Sept-21	MoH	N	N	N	N	N	N	18.00		08-Sept-21	22-Sept-21	25-Sept-21		
C19IMPACT_2229	Trinidad and Tobago	05-Sept-21	MoH	N	N	N	N	N	Y	17.00		08-Sept-21	22-Sept-21	25-Sept-21		21-Jan-22
C19IMPACT_2230	Trinidad and Tobago	05-Sept-21	MoH	N	N	N	N	N	Y	17.00		08-Sept-21	22-Sept-21	25-Sept-21		21-Jan-22
C19IMPACT_2231	Trinidad and Tobago	05-Sept-21	MoH	N	N	N	N	N	N		11.00	08-Sept-21	22-Sept-21	25-Sept-21		21-Jan-22
C19IMPACT_2232	Trinidad and Tobago	05-Sept-21	MoH	N	N	N	N	N	N		5.00	08-Sept-21	22-Sept-21	25-Sept-21		21-Jan-22
C19IMPACT_2233	Trinidad and Tobago	05-Sept-21	MoH	N	N	N	N	N	N		7.00	08-Sept-21	22-Sept-21	25-Sept-21		21-Jan-22
C19IMPACT_2234	Trinidad and Tobago	06-Sept-21	MoH	N	N	N	N	N	N	18.00		08-Sept-21	22-Sept-21	25-Sept-21		21-Jan-22
C19IMPACT_2235	Trinidad and Tobago	06-Sept-21	MoH	N	N	N	N	N	N	15.00		08-Sept-21	22-Sept-21	25-Sept-21		21-Jan-22
C19IMPACT_2236	Trinidad and Tobago	16-Sept-21	MoH	N	N	N	N	N	Y			17-Sept-21	22-Sept-21	25-Sept-21		21-Jan-22

C19IMPACT_2237	Trinidad and Tobago		MoH	N	N	N	N	N	Y			08-Sept-21	22-Sept-21	25-Sept-21		
C19IMPACT_2238	Barbados	22-Jul-21	CARPHA	N	N	N	N	N	N	20.40		08-Sept-21	23-Sept-21		28-Sept-21	17-Dec-21
C19IMPACT_2239	Barbados	26-Jul-21	CARPHA	N	N	N	N	N	N	23.88		08-Sept-21	23-Sept-21		28-Sept-21	17-Dec-21
C19IMPACT_2240	Barbados	28-Jul-21	CARPHA	N	N	N	N	N	N	21.29		08-Sept-21	23-Sept-21		28-Sept-21	17-Dec-21
C19IMPACT_2241	Barbados	29-Jul-21	CARPHA	N	N	N	N	N	N	16.49		08-Sept-21	23-Sept-21		28-Sept-21	17-Dec-21
C19IMPACT_2242	Barbados	31-Jul-21	CARPHA	N	N	N	N	N	N	25.71		08-Sept-21	23-Sept-21		28-Sept-21	17-Dec-21
C19IMPACT_2243	Barbados	01-Aug-21	CARPHA	N	N	N	N	N	N	20.03		08-Sept-21	23-Sept-21		28-Sept-21	17-Dec-21
C19IMPACT_2244	Barbados	01-Aug-21	CARPHA	N	N	N	N	N	N	15.91		08-Sept-21	23-Sept-21		28-Sept-21	17-Dec-21
C19IMPACT_2245	Barbados	01-Aug-21	CARPHA	N	N	N	N	N	N	24.98		08-Sept-21	23-Sept-21		28-Sept-21	17-Dec-21
C19IMPACT_2246	Barbados	01-Aug-21	CARPHA	N	N	N	N	N	N	19.95		08-Sept-21	23-Sept-21		28-Sept-21	17-Dec-21
C19IMPACT_2247	Barbados	02-Aug-21	CARPHA	N	N	N	N	N	N	13.05		08-Sept-21	23-Sept-21		28-Sept-21	17-Dec-21
C19IMPACT_2248	Trinidad and Tobago	13-Aug-21	CARPHA	N	N	N	N	N	N	16.79		08-Sept-21	23-Sept-21	25-Sept-21		17-Dec-21
C19IMPACT_2249	Trinidad and Tobago	15-Aug-21	CARPHA	N	N	N	N	N	N	23.35		08-Sept-21	23-Sept-21	25-Sept-21		17-Dec-21
C19IMPACT_2250	Trinidad and Tobago	15-Aug-21	CARPHA	N	N	N	N	N	N	20.15		08-Sept-21	23-Sept-21	25-Sept-21		17-Dec-21
C19IMPACT_2251	Grenada	18-Aug-21	CARPHA	N	N	N	N	N	N	21.28		10-Sept-21	23-Sept-21		25-Sept-21	17-Dec-21
C19IMPACT_2252	Grenada	18-Aug-21	CARPHA	N	N	N	N	N	N	14.22		10-Sept-21	23-Sept-21		30-Sept-21	17-Dec-21
C19IMPACT_2253	Grenada	19-Aug-21	CARPHA	N	N	N	N	N	N	27.77		10-Sept-21	23-Sept-21		25-Sept-21	17-Dec-21
C19IMPACT_2254	Grenada	19-Aug-21	CARPHA	N	N	N	N	N	N	15.99		10-Sept-21	23-Sept-21		25-Sept-21	17-Dec-21
C19IMPACT_2255	Antigua and Barbuda	21-Aug-21	CARPHA	N	N	N	N	N	N	12.90		10-Sept-21	23-Sept-21		30-Sept-21	17-Dec-21
C19IMPACT_2256	Antigua and Barbuda	22-Aug-21	CARPHA	N	N	N	N	N	N	20.69		10-Sept-21	23-Sept-21		30-Sept-21	17-Dec-21
C19IMPACT_2257	Antigua and Barbuda	23-Aug-21	CARPHA	N	N	N	N	N	N	15.77		10-Sept-21	23-Sept-21		30-Sept-21	17-Dec-21
C19IMPACT_2258	Grenada	25-Aug-21	CARPHA	N	N	N	N	N	N	15.82		10-Sept-21	23-Sept-21		25-Sept-21	17-Dec-21
C19IMPACT_2259	Montserrat	27-Aug-21	CARPHA	N	N	N	N	N	N	13.65		08-Sept-21	23-Sept-21		28-Sept-21	17-Dec-21
C19IMPACT_2260	Saint Vincent and the Grenadines	28-Aug-21	CARPHA	N	N	N	N	N	N	17.42		08-Sept-21	23-Sept-21		28-Sept-21	17-Dec-21
C19IMPACT_2261	Montserrat	28-Aug-21	CARPHA	N	N	N	N	N	N	16.01		08-Sept-21	23-Sept-21		28-Sept-21	17-Dec-21
C19IMPACT_2262	Trinidad and Tobago	29-Aug-21	MoH	N	N	N	N	N	N		9.00	08-Sept-21	23-Sept-21	29-Oct-21		17-Dec-21
C19IMPACT_2263	Trinidad and Tobago	30-Aug-21	MoH	N	N	N	N	N	N		14.00	08-Sept-21	23-Sept-21	29-Oct-21		17-Dec-21
C19IMPACT_2264	Trinidad and Tobago	31-Aug-21	MoH	N	N	N	N	N	N		3.00	08-Sept-21	23-Sept-21	29-Oct-21		17-Dec-21
C19IMPACT_2265	Saint Vincent and the Grenadines	31-Aug-21	CARPHA	N	N	N	N	N	N	15.47		10-Sept-21	23-Sept-21		25-Sept-21	17-Dec-21
C19IMPACT_2266	Saint Vincent and the Grenadines	31-Aug-21	CARPHA	N	N	N	N	N	N	14.45		10-Sept-21	23-Sept-21		25-Sept-21	17-Dec-21
C19IMPACT_2267	Grenada	31-Aug-21	CARPHA	N	N	N	N	N	N	14.43		20-Sept-21	23-Sept-21		30-Sept-21	17-Dec-21
C19IMPACT_2268	Trinidad and Tobago	02-Sept-21	MoH	N	N	N	N	N	Y		5.24	10-Sept-21	23-Sept-21	25-Sept-21		17-Dec-21
C19IMPACT_2269	Trinidad and Tobago	02-Sept-21	MoH	N	N	N	N	N	Y		6.32	10-Sept-21	23-Sept-21	25-Sept-21		17-Dec-21
C19IMPACT_2270	Trinidad and Tobago	02-Sept-21	MoH	N	N	N	N	N	Y		5.27	10-Sept-21	23-Sept-21	29-Oct-21		17-Dec-21
C19IMPACT_2271	Trinidad and Tobago	03-Sept-21	MoH	N	N	N	N	N	N	15.00		08-Sept-21	23-Sept-21	29-Oct-21		17-Dec-21
C19IMPACT_2272	Trinidad and Tobago	03-Sept-21	MoH	N	N	N	N	N	N		5.00	08-Sept-21	23-Sept-21	29-Oct-21		17-Dec-21
C19IMPACT_2273	Trinidad and Tobago	03-Sept-21	MoH	N	N	N	N	N	Y		8.96	10-Sept-21	23-Sept-21	25-Sept-21		17-Dec-21
C19IMPACT_2274	Trinidad and Tobago	03-Sept-21	MoH	N	N	N	N	N	Y		6.92	10-Sept-21	23-Sept-21	25-Sept-21		17-Dec-21
C19IMPACT_2275	Grenada	03-Sept-21	CARPHA	N	N	N	N	N	N	19.46		20-Sept-21	23-Sept-21		30-Sept-21	17-Dec-21
C19IMPACT_2276	Grenada	03-Sept-21	CARPHA	N	N	N	N	N	N	19.52		20-Sept-21	23-Sept-21		30-Sept-21	17-Dec-21
C19IMPACT_2277	Trinidad and Tobago	03-Sept-21	MoH	N	N	N	N	N	Y		15.36	10-Sept-21	23-Sept-21	29-Oct-21		
C19IMPACT_2278	Trinidad and Tobago	04-Sept-21	MoH	N	N	N	N	N	N	9.00		08-Sept-21	23-Sept-21	29-Oct-21		17-Dec-21
C19IMPACT_2279	Trinidad and Tobago	04-Sept-21	MoH	N	N	N	N	N	N		6.00	08-Sept-21	23-Sept-21	29-Oct-21		17-Dec-21
C19IMPACT_2280	Trinidad and Tobago	04-Sept-21	MoH	N	N	N	N	N	N		8.00	08-Sept-21	23-Sept-21	29-Oct-21		17-Dec-21
C19IMPACT_2281	Trinidad and Tobago	04-Sept-21	MoH	N	N	N	N	N	Y		10.40	10-Sept-21	23-Sept-21	25-Sept-21		17-Dec-21
C19IMPACT_2282	Trinidad and Tobago	04-Sept-21	MoH	N	N	N	N	N	Y		6.44	10-Sept-21	23-Sept-21	25-Sept-21		17-Dec-21
C19IMPACT_2283	Trinidad and Tobago	06-Sept-21	MoH	N	N	N	N	N	N	15.00		08-Sept-21	23-Sept-21	29-Oct-21		17-Dec-21
C19IMPACT_2284	Trinidad and Tobago	06-Sept-21	CARPHA	N	N	N	N	N	N	15.90		10-Sept-21	23-Sept-21	25-Sept-21	25-Sept-21	
C19IMPACT_2285	Trinidad and Tobago	06-Sept-21	CARPHA	N	N	N	N	N	N	19.06		10-Sept-21	23-Sept-21	25-Sept-21	25-Sept-21	
C19IMPACT_2286	Trinidad and Tobago	06-Sept-21	CARPHA	N	N	N	N	N	N	15.01		10-Sept-21	23-Sept-21	25-Sept-21	25-Sept-21	
C19IMPACT_2287	Trinidad and Tobago	06-Sept-21	MoH	N	N	N	N	N	N	17.50		10-Sept-21	23-Sept-21	25-Sept-21		
C19IMPACT_2288	Trinidad and Tobago	06-Sept-21	MoH	N	N	N	N	N	N	13.40		10-Sept-21	23-Sept-21	25-Sept-21		
C19IMPACT_2289	Trinidad and Tobago	06-Sept-21	MoH	N	N	N	N	N	N		4.00	10-Sept-21	23-Sept-21	25-Sept-21		17-Dec-21
C19IMPACT_2290	Trinidad and Tobago	06-Sept-21	CARPHA	N	N	N	N	N	N	17.23		10-Sept-21	23-Sept-21	25-Sept-21	26-Sept-21	17-Dec-21
C19IMPACT_2291	Trinidad and Tobago	06-Sept-21	CARPHA	N	N	N	N	N	N	14.72		10-Sept-21	23-Sept-21	25-Sept-21	26-Sept-21	17-Dec-21
C19IMPACT_2292	Trinidad and Tobago	06-Sept-21	CARPHA	N	N	N	N	N	N	15.65		10-Sept-21	23-Sept-21	25-Sept-21	25-Sept-21	17-Dec-21
C19IMPACT_2293	Trinidad and Tobago	06-Sept-21	CARPHA	N	N	N	N	N	N	21.27		10-Sept-21	23-Sept-21	25-Sept-21	26-Sept-21	17-Dec-21
C19IMPACT_2294	Trinidad and Tobago	06-Sept-21	MoH	N	N	N	N	N	N	14.00		10-Sept-21	23-Sept-21	25-Sept-21		
C19IMPACT_2295	Trinidad and Tobago	06-Sept-21	MoH	N	N	N	N	N	N	15.00		10-Sept-21	23-Sept-21	25-Sept-21		17-Dec-21
C19IMPACT_2296	Trinidad and Tobago	06-Sept-21	MoH	N	N	N	N	N	N	16.00		10-Sept-21	23-Sept-21	25-Sept-21		17-Dec-21
C19IMPACT_2297	Grenada	06-Sept-21	CARPHA	N	N	N	N	N	N	15.97		20-Sept-21	23-Sept-21		30-Sept-21	17-Dec-21
C19IMPACT_2298	Trinidad and Tobago	06-Sept-21	MoH	N	N	N	N	N	Y	30.00		15-Sept-21	23-Sept-21	01-Oct-21		
C19IMPACT_2299	Trinidad and Tobago	06-Sept-21	MoH	N	N	N	N	N	Y	18.00		15-Sept-21	23-Sept-21	01-Oct-21		17-Dec-21
C19IMPACT_2300	Trinidad and Tobago	06-Sept-21	MoH	N	N	N	N	N	Y	22.00		15-Sept-21	23-Sept-21	01-Oct-21		17-Dec-21

C19IMPACT_2301	Trinidad and Tobago	06-Sept-21	MoH	N	N	N	N	N	Y	17.00		15-Sept-21	23-Sept-21	01-Oct-21		17-Dec-21
C19IMPACT_2302	Trinidad and Tobago	06-Sept-21	CARPHA	N	N	N	N	N	N	19.05		10-Sept-21	23-Sept-21	29-Oct-21	29-Oct-21	
C19IMPACT_2303	Trinidad and Tobago	06-Sept-21	MoH	N	N	N	N	N	N		12.00	10-Sept-21	23-Sept-21	29-Oct-21		
C19IMPACT_2304	Trinidad and Tobago	06-Sept-21	MoH	N	N	N	N	N	N		15.00	10-Sept-21	23-Sept-21	29-Oct-21		
C19IMPACT_2305	Trinidad and Tobago	07-Sept-21	MoH	N	N	N	N	N	N	18.00		10-Sept-21	23-Sept-21	25-Sept-21		17-Dec-21
C19IMPACT_2306	Trinidad and Tobago	07-Sept-21	MoH	N	N	N	N	N	N	18.00		10-Sept-21	23-Sept-21	25-Sept-21		17-Dec-21
C19IMPACT_2307	Trinidad and Tobago	07-Sept-21	MoH	N	N	N	N	N	N	19.00		10-Sept-21	23-Sept-21	25-Sept-21		17-Dec-21
C19IMPACT_2308	Trinidad and Tobago	07-Sept-21	MoH	N	N	N	N	N	N	18.00		10-Sept-21	23-Sept-21	25-Sept-21		17-Dec-21
C19IMPACT_2309	Trinidad and Tobago	07-Sept-21	MoH	N	N	N	N	N	N	17.00		10-Sept-21	23-Sept-21	25-Sept-21		17-Dec-21
C19IMPACT_2310	Trinidad and Tobago	07-Sept-21	MoH	N	N	N	N	N	N	19.00		10-Sept-21	23-Sept-21	25-Sept-21		17-Dec-21
C19IMPACT_2311	Trinidad and Tobago	07-Sept-21	MoH	N	N	N	N	N	N	19.00		10-Sept-21	23-Sept-21	25-Sept-21		17-Dec-21
C19IMPACT_2312	Trinidad and Tobago	07-Sept-21	MoH	N	N	N	N	N	N	17.00		10-Sept-21	23-Sept-21	25-Sept-21		17-Dec-21
C19IMPACT_2313	Trinidad and Tobago	07-Sept-21	MoH	N	N	N	N	N	N	18.00		10-Sept-21	23-Sept-21	25-Sept-21		17-Dec-21
C19IMPACT_2314	Trinidad and Tobago	07-Sept-21	MoH	N	N	N	N	N	N	19.00		10-Sept-21	23-Sept-21	25-Sept-21		17-Dec-21
C19IMPACT_2315	Trinidad and Tobago	07-Sept-21	MoH	N	N	N	N	N	N	18.00		10-Sept-21	23-Sept-21	25-Sept-21		17-Dec-21
C19IMPACT_2316	Trinidad and Tobago	07-Sept-21	CARPHA	N	N	N	N	N	N	14.62		10-Sept-21	23-Sept-21	01-Oct-21	30-Sept-21	17-Dec-21
C19IMPACT_2317	Grenada	08-Sept-21	CARPHA	N	N	N	N	N	N	27.39		20-Sept-21	23-Sept-21		30-Sept-21	
C19IMPACT_2318	Grenada	08-Sept-21	CARPHA	N	N	N	N	N	N	15.70		20-Sept-21	23-Sept-21		30-Sept-21	17-Dec-21
C19IMPACT_2319	Grenada	08-Sept-21	CARPHA	N	N	N	N	N	N	28.37		20-Sept-21	23-Sept-21		30-Sept-21	
C19IMPACT_2320	Grenada	09-Sept-21	CARPHA	N	N	N	N	N	Y	17.42		20-Sept-21	23-Sept-21		30-Sept-21	17-Dec-21
C19IMPACT_2321	Grenada	09-Sept-21	CARPHA	N	N	N	N	N	N	16.13		20-Sept-21	23-Sept-21		30-Sept-21	17-Dec-21
C19IMPACT_2322	Grenada	09-Sept-21	CARPHA	N	N	N	N	N	N	10.95		20-Sept-21	23-Sept-21		30-Sept-21	17-Dec-21
C19IMPACT_2323	Grenada	09-Sept-21	CARPHA	N	N	N	N	N	N	15.91		20-Sept-21	23-Sept-21		30-Sept-21	17-Dec-21
C19IMPACT_2324	Trinidad and Tobago	09-Sept-21	MoH	N	N	N	N	N	Y	25.00		15-Sept-21	23-Sept-21	01-Oct-21		17-Dec-21
C19IMPACT_2325	Grenada	09-Sept-21	CARPHA	N	N	N	N	N	N	18.50		20-Sept-21	23-Sept-21		06-Nov-21	17-Dec-21
C19IMPACT_2326	Grenada	10-Sept-21	CARPHA	N	N	N	N	N	N	14.22		20-Sept-21	23-Sept-21		30-Sept-21	17-Dec-21
C19IMPACT_2327	Grenada	10-Sept-21	CARPHA	N	N	N	N	N	N	12.72		20-Sept-21	23-Sept-21		30-Sept-21	17-Dec-21
C19IMPACT_2328	Grenada	10-Sept-21	CARPHA	N	N	N	N	N	N	13.33		20-Sept-21	23-Sept-21		30-Sept-21	17-Dec-21
C19IMPACT_2329	Grenada	10-Sept-21	CARPHA	N	N	N	N	N	N	15.25		20-Sept-21	23-Sept-21		30-Sept-21	
C19IMPACT_2330	Grenada	11-Sept-21	CARPHA	N	N	N	N	N	N	14.70		20-Sept-21	23-Sept-21		30-Sept-21	17-Dec-21
C19IMPACT_2331	Grenada	11-Sept-21	CARPHA	N	N	N	N	N	N	13.20		20-Sept-21	23-Sept-21		30-Sept-21	17-Dec-21
C19IMPACT_2332	Grenada	11-Sept-21	CARPHA	N	N	N	N	N	N	14.35		20-Sept-21	23-Sept-21		30-Sept-21	17-Dec-21
C19IMPACT_2333	Trinidad and Tobago	12-Sept-21	MoH	N	N	N	N	N	Y	17.00		15-Sept-21	23-Sept-21	01-Oct-21		17-Dec-21
C19IMPACT_2334	Grenada	12-Sept-21	CARPHA	N	N	N	N	N	N	13.61		20-Sept-21	23-Sept-21		30-Sept-21	17-Dec-21
C19IMPACT_2335	Trinidad and Tobago	12-Sept-21	MoH	N	N	N	N	N	Y	18.00		15-Sept-21	23-Sept-21	01-Oct-21		
C19IMPACT_2336	Trinidad and Tobago	12-Sept-21	MoH	N	N	N	N	N	Y	17.00		15-Sept-21	23-Sept-21	01-Oct-21		
C19IMPACT_2337	Grenada	12-Sept-21	CARPHA	N	N	N	N	N	N	12.42		20-Sept-21	23-Sept-21		30-Sept-21	17-Dec-21
C19IMPACT_2338	Trinidad and Tobago	12-Sept-21	MoH	N	N	N	N	N	Y		4.00	15-Sept-21	23-Sept-21	01-Oct-21		17-Dec-21
C19IMPACT_2339	Trinidad and Tobago	12-Sept-21	MoH	N	N	N	N	N	Y	12.00		15-Sept-21	23-Sept-21	01-Oct-21		17-Dec-21
C19IMPACT_2340	Grenada	13-Sept-21	CARPHA	N	N	N	N	N	N	11.15		20-Sept-21	23-Sept-21		30-Sept-21	17-Dec-21
C19IMPACT_2341	Grenada	13-Sept-21	CARPHA	N	N	N	N	N	N	15.64		20-Sept-21	23-Sept-21		30-Sept-21	17-Dec-21
C19IMPACT_2342	Grenada	13-Sept-21	CARPHA	N	N	N	N	N	N	26.10		20-Sept-21	23-Sept-21		30-Sept-21	17-Dec-21
C19IMPACT_2343	Grenada	13-Sept-21	CARPHA	N	N	N	N	N	N	27.96		20-Sept-21	23-Sept-21		30-Sept-21	17-Dec-21
C19IMPACT_2344	Grenada	14-Sept-21	CARPHA	N	N	N	N	N	N	19.58		20-Sept-21	23-Sept-21		30-Sept-21	17-Dec-21
C19IMPACT_2345	Grenada	14-Sept-21	CARPHA	N	N	N	N	N	N	12.23		20-Sept-21	23-Sept-21		30-Sept-21	17-Dec-21
C19IMPACT_2346	Grenada	14-Sept-21	CARPHA	N	N	N	N	N	N	17.89		20-Sept-21	23-Sept-21		30-Sept-21	17-Dec-21
C19IMPACT_2347	Grenada	14-Sept-21	CARPHA	N	N	N	N	N	N	19.85		20-Sept-21	23-Sept-21		30-Sept-21	17-Dec-21
C19IMPACT_2348	Trinidad and Tobago	10-Jun-21	CARPHA	N	N	N	N	N	N	11.31		15-Sept-21	27-Sept-21	08-Oct-21	04-Oct-21	
C19IMPACT_2349	Grenada	18-Aug-21	CARPHA	N	N	N	N	N	N	18.96		10-Sept-21	27-Sept-21		04-Oct-21	21-Jan-22
C19IMPACT_2350	Grenada	18-Aug-21	CARPHA	N	N	N	N	N	N	20.60		10-Sept-21	27-Sept-21		04-Oct-21	21-Jan-22
C19IMPACT_2351	Antigua and Barbuda	19-Aug-21	CARPHA	N	N	N	N	N	N	21.46		10-Sept-21	27-Sept-21		04-Oct-21	21-Jan-22
C19IMPACT_2352	Antigua and Barbuda	19-Aug-21	CARPHA	N	N	N	N	N	N	22.63		10-Sept-21	27-Sept-21		04-Oct-21	
C19IMPACT_2353	Antigua and Barbuda	19-Aug-21	CARPHA	N	N	N	N	N	N	26.57		10-Sept-21	27-Sept-21		04-Oct-21	
C19IMPACT_2354	Antigua and Barbuda	19-Aug-21	CARPHA	N	N	N	N	N	N	16.88		10-Sept-21	27-Sept-21		04-Oct-21	21-Jan-22
C19IMPACT_2355	Antigua and Barbuda	19-Aug-21	CARPHA	N	N	N	N	N	N	19.53		10-Sept-21	27-Sept-21		04-Oct-21	21-Jan-22
C19IMPACT_2356	Antigua and Barbuda	20-Aug-21	CARPHA	N	N	N	N	N	N	16.98		10-Sept-21	27-Sept-21		04-Oct-21	21-Jan-22
C19IMPACT_2357	Antigua and Barbuda	20-Aug-21	CARPHA	N	N	N	N	N	N	28.28		10-Sept-21	27-Sept-21		04-Oct-21	
C19IMPACT_2358	Antigua and Barbuda	20-Aug-21	CARPHA	N	N	N	N	N	N	15.38		10-Sept-21	27-Sept-21		04-Oct-21	21-Jan-22
C19IMPACT_2359	Antigua and Barbuda	20-Aug-21	CARPHA	N	N	N	N	N	N	20.82		10-Sept-21	27-Sept-21		04-Oct-21	
C19IMPACT_2360	Antigua and Barbuda	20-Aug-21	CARPHA	N	N	N	N	N	N	18.11		10-Sept-21	27-Sept-21		04-Oct-21	21-Jan-22
C19IMPACT_2361	Antigua and Barbuda	21-Aug-21	CARPHA	N	N	N	N	N	N	16.79		10-Sept-21	27-Sept-21		04-Oct-21	
C19IMPACT_2362	Antigua and Barbuda	21-Aug-21	CARPHA	N	N	N	N	N	N	17.87		10-Sept-21	27-Sept-21		04-Oct-21	21-Jan-22
C19IMPACT_2363	Antigua and Barbuda	21-Aug-21	CARPHA	N	N	N	N	N	N	11.84		10-Sept-21	27-Sept-21		04-Oct-21	21-Jan-22
C19IMPACT_2364	Antigua and Barbuda	21-Aug-21	CARPHA	N	N	N	N	N	N	18.74		10-Sept-21	27-Sept-21		04-Oct-21	21-Jan-22

C19IMPACT_2365	Antigua and Barbuda	21-Aug-21	CARPHA	N	N	N	N	N	N	14.40		10-Sept-21	27-Sept-21		04-Oct-21	21-Jan-22
C19IMPACT_2366	Antigua and Barbuda	22-Aug-21	CARPHA	N	N	N	N	N	N	18.02		10-Sept-21	27-Sept-21		04-Oct-21	21-Jan-22
C19IMPACT_2367	Antigua and Barbuda	22-Aug-21	CARPHA	N	N	N	N	N	N	14.21		10-Sept-21	27-Sept-21		04-Oct-21	21-Jan-22
C19IMPACT_2368	Antigua and Barbuda	22-Aug-21	CARPHA	N	N	N	N	N	N	20.24		10-Sept-21	27-Sept-21		04-Oct-21	21-Jan-22
C19IMPACT_2369	Antigua and Barbuda	22-Aug-21	CARPHA	N	N	N	N	N	N	12.16		10-Sept-21	27-Sept-21		04-Oct-21	21-Jan-22
C19IMPACT_2370	Antigua and Barbuda	22-Aug-21	CARPHA	N	N	N	N	N	N	17.20		10-Sept-21	27-Sept-21		04-Oct-21	21-Jan-22
C19IMPACT_2371	British Virgin Islands	01-Sept-21	CARPHA	N	N	N	N	N	N	20.86		13-Sept-21	27-Sept-21		04-Oct-21	21-Jan-22
C19IMPACT_2372	British Virgin Islands	01-Sept-21	CARPHA	N	N	N	N	N	N	17.82		13-Sept-21	27-Sept-21		04-Oct-21	21-Jan-22
C19IMPACT_2373	British Virgin Islands	01-Sept-21	CARPHA	N	N	N	N	N	N	17.13		13-Sept-21	27-Sept-21		04-Oct-21	21-Jan-22
C19IMPACT_2374	Saint Vincent and the Grenadines	02-Sept-21	CARPHA	N	N	N	N	N	N	22.71		13-Sept-21	27-Sept-21		04-Oct-21	21-Jan-22
C19IMPACT_2375	Saint Vincent and the Grenadines	02-Sept-21	CARPHA	N	N	N	N	N	N	20.65		13-Sept-21	27-Sept-21		04-Oct-21	21-Jan-22
C19IMPACT_2376	Turks and Caicos Islands	02-Sept-21	CARPHA	N	N	N	N	N	N	17.96		17-Sept-21	27-Sept-21		06-Nov-21	21-Jan-22
C19IMPACT_2377	Turks and Caicos Islands	02-Sept-21	CARPHA	N	N	N	N	N	N	16.57		17-Sept-21	27-Sept-21		06-Nov-21	21-Jan-22
C19IMPACT_2378	Turks and Caicos Islands	02-Sept-21	CARPHA	N	N	N	N	N	N	17.35		17-Sept-21	27-Sept-21		06-Nov-21	21-Jan-22
C19IMPACT_2379	Trinidad and Tobago	02-Sept-21	MoH	N	N	N	N	N	N	15.00		15-Sept-21	27-Sept-21	13-Nov-21		21-Jan-22
C19IMPACT_2380	Trinidad and Tobago	02-Sept-21	MoH	N	N	N	N	N	N	14.00		15-Sept-21	27-Sept-21	13-Nov-21		21-Jan-22
C19IMPACT_2381	Trinidad and Tobago	02-Sept-21	MoH	N	N	N	N	N	N	18.00		15-Sept-21	27-Sept-21	13-Nov-21		21-Jan-22
C19IMPACT_2382	Saint Vincent and the Grenadines	03-Sept-21	CARPHA	N	N	N	N	N	N	20.04		13-Sept-21	27-Sept-21		04-Oct-21	21-Jan-22
C19IMPACT_2383	Saint Vincent and the Grenadines	03-Sept-21	CARPHA	N	N	N	N	N	N	19.55		13-Sept-21	27-Sept-21		04-Oct-21	21-Jan-22
C19IMPACT_2384	Saint Vincent and the Grenadines	03-Sept-21	CARPHA	N	N	N	N	N	N	16.37		13-Sept-21	27-Sept-21		04-Oct-21	21-Jan-22
C19IMPACT_2385	Saint Vincent and the Grenadines	03-Sept-21	CARPHA	N	N	N	N	N	N	20.88		13-Sept-21	27-Sept-21		04-Oct-21	21-Jan-22
C19IMPACT_2386	Saint Vincent and the Grenadines	03-Sept-21	CARPHA	N	N	N	N	N	N	21.43		13-Sept-21	27-Sept-21		04-Oct-21	21-Jan-22
C19IMPACT_2387	Turks and Caicos Islands	03-Sept-21	CARPHA	N	N	N	N	N	N	17.98		15-Sept-21	27-Sept-21		04-Oct-21	21-Jan-22
C19IMPACT_2388	Turks and Caicos Islands	03-Sept-21	CARPHA	N	N	N	N	N	N	15.06		15-Sept-21	27-Sept-21		04-Oct-21	21-Jan-22
C19IMPACT_2389	Turks and Caicos Islands	03-Sept-21	CARPHA	N	N	N	N	N	N	17.17		17-Sept-21	27-Sept-21		06-Nov-21	21-Jan-22
C19IMPACT_2390	Turks and Caicos Islands	03-Sept-21	CARPHA	N	N	N	N	N	N	20.28		17-Sept-21	27-Sept-21		06-Nov-21	21-Jan-22
C19IMPACT_2391	Turks and Caicos Islands	03-Sept-21	CARPHA	N	N	N	N	N	N	20.10		17-Sept-21	27-Sept-21		06-Nov-21	21-Jan-22
C19IMPACT_2392	Saint Vincent and the Grenadines	04-Sept-21	CARPHA	N	N	N	N	N	N	18.57		13-Sept-21	27-Sept-21		04-Oct-21	21-Jan-22
C19IMPACT_2393	Saint Vincent and the Grenadines	04-Sept-21	CARPHA	N	N	N	N	N	N	18.46		13-Sept-21	27-Sept-21		04-Oct-21	21-Jan-22
C19IMPACT_2394	Saint Vincent and the Grenadines	04-Sept-21	CARPHA	N	N	N	N	N	N	13.78		13-Sept-21	27-Sept-21		04-Oct-21	21-Jan-22
C19IMPACT_2395	Saint Vincent and the Grenadines	04-Sept-21	CARPHA	N	N	N	N	N	N	16.62		13-Sept-21	27-Sept-21		04-Oct-21	21-Jan-22
C19IMPACT_2396	Trinidad and Tobago	05-Sept-21	MoH	N	N	N	N	N	Y		4.00	13-Sept-21	27-Sept-21	08-Oct-21		21-Jan-22
C19IMPACT_2397	Saint Vincent and the Grenadines	05-Sept-21	CARPHA	N	N	N	N	N	N	16.95		15-Sept-21	27-Sept-21		04-Oct-21	21-Jan-22
C19IMPACT_2398	Saint Vincent and the Grenadines	05-Sept-21	CARPHA	N	N	N	N	N	N	19.76		15-Sept-21	27-Sept-21		04-Oct-21	21-Jan-22
C19IMPACT_2399	Saint Vincent and the Grenadines	05-Sept-21	CARPHA	N	N	N	N	N	N	17.00		15-Sept-21	27-Sept-21		04-Oct-21	21-Jan-22
C19IMPACT_2400	British Virgin Islands	06-Sept-21	CARPHA	N	N	N	N	N	N	22.23		13-Sept-21	27-Sept-21		04-Oct-21	21-Jan-22
C19IMPACT_2401	British Virgin Islands	06-Sept-21	CARPHA	N	N	N	N	N	N	15.71		13-Sept-21	27-Sept-21		04-Oct-21	21-Jan-22
C19IMPACT_2402	Trinidad and Tobago	06-Sept-21	MoH	N	N	N	N	N	Y		3.00	13-Sept-21	27-Sept-21	08-Oct-21		21-Jan-22
C19IMPACT_2403	Trinidad and Tobago	06-Sept-21	MoH	N	N	N	N	N	Y		2.00	13-Sept-21	27-Sept-21	08-Oct-21		21-Jan-22
C19IMPACT_2404	Saint Vincent and the Grenadines	06-Sept-21	CARPHA	N	N	N	N	N	N	17.52		15-Sept-21	27-Sept-21		04-Oct-21	21-Jan-22
C19IMPACT_2405	Saint Vincent and the Grenadines	06-Sept-21	CARPHA	N	N	N	N	N	N	17.04		15-Sept-21	27-Sept-21		04-Oct-21	21-Jan-22
C19IMPACT_2406	Turks and Caicos Islands	06-Sept-21	CARPHA	N	N	N	N	N	N	16.27		17-Sept-21	27-Sept-21		06-Nov-21	21-Jan-22
C19IMPACT_2407	Turks and Caicos Islands	06-Sept-21	CARPHA	N	N	N	N	N	N	17.49		17-Sept-21	27-Sept-21		06-Nov-21	21-Jan-22
C19IMPACT_2408	Trinidad and Tobago	07-Sept-21	CARPHA	N	N	N	N	N	N	16.84		13-Sept-21	27-Sept-21	08-Oct-21	04-Oct-21	21-Jan-22
C19IMPACT_2409	Trinidad and Tobago	07-Sept-21	MoH	N	N	N	N	N	Y	18.00		13-Sept-21	27-Sept-21	08-Oct-21		21-Jan-22
C19IMPACT_2410	Trinidad and Tobago	07-Sept-21	MoH	N	N	N	N	N	Y	20.00		13-Sept-21	27-Sept-21	08-Oct-21		21-Jan-22
C19IMPACT_2411	Trinidad and Tobago	07-Sept-21	MoH	N	N	N	N	N	Y		6.00	13-Sept-21	27-Sept-21	08-Oct-21		21-Jan-22
C19IMPACT_2412	Trinidad and Tobago	07-Sept-21	MoH	N	N	N	N	N	N	19.00		13-Sept-21	27-Sept-21	08-Oct-21		21-Jan-22
C19IMPACT_2413	Trinidad and Tobago	07-Sept-21	MoH	N	N	N	N	N	N	17.00		15-Sept-21	27-Sept-21	13-Nov-21		21-Jan-22
C19IMPACT_2414	Trinidad and Tobago	07-Sept-21	MoH	N	N	N	N	N	N		7.00	15-Sept-21	27-Sept-21	13-Nov-21		21-Jan-22
C19IMPACT_2415	Trinidad and Tobago	08-Sept-21	MoH	N	N	N	N	N	N	17.00		13-Sept-21	27-Sept-21	08-Oct-21		21-Jan-22
C19IMPACT_2416	Trinidad and Tobago	08-Sept-21	MoH	N	N	N	N	N	Y	16.00		13-Sept-21	27-Sept-21	08-Oct-21		21-Jan-22
C19IMPACT_2417	Saint Vincent and the Grenadines	08-Sept-21	CARPHA	N	N	N	N	N	N	16.62		15-Sept-21	27-Sept-21		04-Oct-21	21-Jan-22
C19IMPACT_2418	Saint Vincent and the Grenadines	08-Sept-21	CARPHA	N	N	N	N	N	N	12.10		15-Sept-21	27-Sept-21		04-Oct-21	21-Jan-22
C19IMPACT_2419	Trinidad and Tobago	08-Sept-21	MoH	N	N	N	N	N	N		6.00	15-Sept-21	27-Sept-21	13-Nov-21		21-Jan-22
C19IMPACT_2420	Trinidad and Tobago	08-Sept-21	MoH	N	N	N	N	N	N		9.00	15-Sept-21	27-Sept-21	13-Nov-21		21-Jan-22
C19IMPACT_2421	Trinidad and Tobago	08-Sept-21	MoH	N	N	N	N	N	N	15.00		15-Sept-21	27-Sept-21	13-Nov-21		
C19IMPACT_2422	Trinidad and Tobago	09-Sept-21	MoH	N	N	N	N	N	Y	17.00		15-Sept-21	27-Sept-21	01-Oct-21		21-Jan-22
C19IMPACT_2423	Trinidad and Tobago	09-Sept-21	MoH	N	N	N	N	N	Y	20.00		13-Sept-21	27-Sept-21	08-Oct-21		21-Jan-22
C19IMPACT_2424	Trinidad and Tobago	09-Sept-21	MoH	N	N	N	N	N	N	18.00		15-Sept-21	27-Sept-21	13-Nov-21		21-Jan-22
C19IMPACT_2425	Trinidad and Tobago	09-Sept-21	MoH	N	N	N	N	N	N	17.00		15-Sept-21	27-Sept-21	13-Nov-21		21-Jan-22
C19IMPACT_2426	Trinidad and Tobago	09-Sept-21	MoH	N	N	N	N	N	N	16.00		15-Sept-21	27-Sept-21	13-Nov-21		
C19IMPACT_2427	Trinidad and Tobago	10-Sept-21	CARPHA	N	N	N	N	N	N	23.34		15-Sept-21	27-Sept-21	08-Oct-21	04-Oct-21	21-Jan-22
C19IMPACT_2428	Trinidad and Tobago	10-Sept-21	CARPHA	N	N	N	N	N	N	13.98		15-Sept-21	27-Sept-21	08-Oct-21	04-Oct-21	21-Jan-22

C19IMPACT_2429	Trinidad and Tobago	10-Sept-21	CARPHA	N	N	N	N	N	N	24.97		15-Sept-21	27-Sept-21	08-Oct-21	04-Oct-21	21-Jan-22
C19IMPACT_2430	Saint Vincent and the Grenadines	11-Sept-21	CARPHA	N	N	N	N	N	N	20.19		15-Sept-21	27-Sept-21		04-Oct-21	21-Jan-22
C19IMPACT_2431	Trinidad and Tobago	11-Sept-21	CARPHA	N	N	N	N	N	N	24.47		15-Sept-21	27-Sept-21	08-Oct-21	04-Oct-21	21-Jan-22
C19IMPACT_2432	Trinidad and Tobago	13-Sept-21	CARPHA	N	N	N	N	N	N	24.40		15-Sept-21	27-Sept-21	08-Oct-21	04-Oct-21	21-Jan-22
C19IMPACT_2433	Trinidad and Tobago	22-Sept-21	MoH	N	N	N	N	N	Y			22-Sept-21	27-Sept-21	08-Oct-21		21-Jan-22
C19IMPACT_2434	Jamaica	03-Aug-21	CARPHA	N	N	N	N	N	N	22.65		20-Sept-21	29-Sept-21		04-Oct-21	21-Jan-22
C19IMPACT_2435	Jamaica	04-Aug-21	CARPHA	N	N	N	N	N	N	22.75		20-Sept-21	29-Sept-21		04-Oct-21	21-Jan-22
C19IMPACT_2436	Jamaica	04-Aug-21	CARPHA	N	N	N	N	N	N	23.29		20-Sept-21	29-Sept-21		04-Oct-21	21-Jan-22
C19IMPACT_2437	Jamaica	04-Aug-21	CARPHA	N	N	N	N	N	N	27.93		20-Sept-21	29-Sept-21		04-Oct-21	
C19IMPACT_2438	Jamaica	04-Aug-21	CARPHA	N	N	N	N	N	N	20.89		20-Sept-21	29-Sept-21		04-Oct-21	21-Jan-22
C19IMPACT_2439	Jamaica	05-Aug-21	CARPHA	N	N	N	N	N	N	28.87		20-Sept-21	29-Sept-21		04-Oct-21	
C19IMPACT_2440	Jamaica	05-Aug-21	CARPHA	N	N	N	N	N	N	16.77		20-Sept-21	29-Sept-21		04-Oct-21	21-Jan-22
C19IMPACT_2441	Jamaica	05-Aug-21	CARPHA	N	N	N	N	N	N	21.62		20-Sept-21	29-Sept-21		04-Oct-21	21-Jan-22
C19IMPACT_2442	Jamaica	05-Aug-21	CARPHA	N	N	N	N	N	N	25.38		20-Sept-21	29-Sept-21		04-Oct-21	21-Jan-22
C19IMPACT_2443	Jamaica	05-Aug-21	CARPHA	N	N	N	N	N	N	24.77		20-Sept-21	29-Sept-21		04-Oct-21	21-Jan-22
C19IMPACT_2444	Jamaica	05-Aug-21	CARPHA	N	N	N	N	N	N	22.24		20-Sept-21	29-Sept-21		04-Oct-21	21-Jan-22
C19IMPACT_2445	Jamaica	05-Aug-21	CARPHA	N	N	N	N	N	N	14.11		20-Sept-21	29-Sept-21		04-Oct-21	21-Jan-22
C19IMPACT_2446	Jamaica	05-Aug-21	CARPHA	N	N	N	N	N	N	15.54		20-Sept-21	29-Sept-21		04-Oct-21	21-Jan-22
C19IMPACT_2447	Jamaica	05-Aug-21	CARPHA	N	N	N	N	N	N	17.15		20-Sept-21	29-Sept-21		04-Oct-21	21-Jan-22
C19IMPACT_2448	Antigua and Barbuda	18-Aug-21	CARPHA	N	N	N	N	N	N	22.27		10-Sept-21	29-Sept-21		04-Oct-21	
C19IMPACT_2449	Antigua and Barbuda	19-Aug-21	CARPHA	N	N	N	N	N	N	15.74		10-Sept-21	29-Sept-21		04-Oct-21	21-Jan-22
C19IMPACT_2450	Antigua and Barbuda	19-Aug-21	CARPHA	N	N	N	N	N	N	14.09		10-Sept-21	29-Sept-21		04-Oct-21	21-Jan-22
C19IMPACT_2451	Antigua and Barbuda	19-Aug-21	CARPHA	N	N	N	N	N	N	12.32		10-Sept-21	29-Sept-21		04-Oct-21	21-Jan-22
C19IMPACT_2452	Antigua and Barbuda	19-Aug-21	CARPHA	N	N	N	N	N	N	25.40		10-Sept-21	29-Sept-21		04-Oct-21	21-Jan-22
C19IMPACT_2453	Antigua and Barbuda	19-Aug-21	CARPHA	N	N	N	N	N	N	20.59		10-Sept-21	29-Sept-21		04-Oct-21	21-Jan-22
C19IMPACT_2454	Antigua and Barbuda	19-Aug-21	CARPHA	N	N	N	N	N	N	25.07		10-Sept-21	29-Sept-21		04-Oct-21	
C19IMPACT_2455	Antigua and Barbuda	19-Aug-21	CARPHA	N	N	N	N	N	N	15.37		10-Sept-21	29-Sept-21		04-Oct-21	21-Jan-22
C19IMPACT_2456	Antigua and Barbuda	19-Aug-21	CARPHA	N	N	N	N	N	N	10.26		10-Sept-21	29-Sept-21		04-Oct-21	21-Jan-22
C19IMPACT_2457	Antigua and Barbuda	19-Aug-21	CARPHA	N	N	N	N	N	N	20.40		10-Sept-21	29-Sept-21		04-Oct-21	21-Jan-22
C19IMPACT_2458	Antigua and Barbuda	19-Aug-21	CARPHA	N	N	N	N	N	N	23.16		10-Sept-21	29-Sept-21		04-Oct-21	
C19IMPACT_2459	Antigua and Barbuda	19-Aug-21	CARPHA	N	N	N	N	N	N	22.26		10-Sept-21	29-Sept-21		04-Oct-21	
C19IMPACT_2460	Antigua and Barbuda	19-Aug-21	CARPHA	N	N	N	N	N	N	21.49		10-Sept-21	29-Sept-21		04-Oct-21	21-Jan-22
C19IMPACT_2461	Antigua and Barbuda	20-Aug-21	CARPHA	N	N	N	N	N	N	22.79		10-Sept-21	29-Sept-21		04-Oct-21	21-Jan-22
C19IMPACT_2462	Antigua and Barbuda	20-Aug-21	CARPHA	N	N	N	N	N	N	19.23		10-Sept-21	29-Sept-21		04-Oct-21	21-Jan-22
C19IMPACT_2463	Saint Vincent and the Grenadines	22-Aug-21	CARPHA	N	N	N	N	N	N	17.32		20-Sept-21	29-Sept-21		04-Oct-21	21-Jan-22
C19IMPACT_2464	Saint Vincent and the Grenadines	24-Aug-21	CARPHA	N	N	N	N	N	N	18.33		20-Sept-21	29-Sept-21		04-Oct-21	21-Jan-22
C19IMPACT_2465	Trinidad and Tobago	07-Sept-21	CARPHA	N	N	N	N	N	N	19.11		13-Sept-21	29-Sept-21	08-Oct-21	04-Oct-21	21-Jan-22
C19IMPACT_2466	Trinidad and Tobago	07-Sept-21	CARPHA	N	N	N	N	N	N	16.57		13-Sept-21	29-Sept-21	08-Oct-21	04-Oct-21	21-Jan-22
C19IMPACT_2467	Trinidad and Tobago	07-Sept-21	CARPHA	N	N	N	N	N	N	15.81		13-Sept-21	29-Sept-21	08-Oct-21	04-Oct-21	21-Jan-22
C19IMPACT_2468	Trinidad and Tobago	07-Sept-21	CARPHA	N	N	N	N	N	N	18.31		13-Sept-21	29-Sept-21	08-Oct-21	04-Oct-21	21-Jan-22
C19IMPACT_2469	Trinidad and Tobago	07-Sept-21	CARPHA	N	N	N	N	N	N	10.38		13-Sept-21	29-Sept-21	08-Oct-21	04-Oct-21	21-Jan-22
C19IMPACT_2470	Trinidad and Tobago	07-Sept-21	CARPHA	N	N	N	N	N	N	23.13		13-Sept-21	29-Sept-21	08-Oct-21	04-Oct-21	
C19IMPACT_2471	Trinidad and Tobago	07-Sept-21	CARPHA	N	N	Y	N	N	N	18.75		20-Sept-21	29-Sept-21	08-Oct-21	04-Oct-21	21-Jan-22
C19IMPACT_2472	Trinidad and Tobago	08-Sept-21	CARPHA	N	N	Y	N	Y	N	13.16		13-Sept-21	29-Sept-21	08-Oct-21	04-Oct-21	21-Jan-22
C19IMPACT_2473	Trinidad and Tobago	08-Sept-21	CARPHA	N	N	Y	N	N	N	14.76		20-Sept-21	29-Sept-21	08-Oct-21	04-Oct-21	21-Jan-22
C19IMPACT_2474	Trinidad and Tobago	09-Sept-21	CARPHA	N	N	Y	N	Y	N	24.98		20-Sept-21	29-Sept-21	08-Oct-21	04-Oct-21	21-Jan-22
C19IMPACT_2475	Trinidad and Tobago	09-Sept-21	MoH	N	N	N	N	Y	Y	15.00		15-Sept-21	29-Sept-21	08-Oct-21		21-Jan-22
C19IMPACT_2476	Trinidad and Tobago	09-Sept-21	MoH	N	N	N	N	N	Y	15.00		15-Sept-21	29-Sept-21	08-Oct-21		21-Jan-22
C19IMPACT_2477	Trinidad and Tobago	09-Sept-21	MoH	N	N	N	N	N	Y	14.00		15-Sept-21	29-Sept-21	08-Oct-21		21-Jan-22
C19IMPACT_2478	Trinidad and Tobago	09-Sept-21	MoH	N	N	N	N	N	Y	17.00		15-Sept-21	29-Sept-21	08-Oct-21		21-Jan-22
C19IMPACT_2479	Trinidad and Tobago	09-Sept-21	CARPHA	N	N	Y	N	N	N	14.98		20-Sept-21	29-Sept-21	08-Oct-21	04-Oct-21	21-Jan-22
C19IMPACT_2480	Trinidad and Tobago	10-Sept-21	MoH	N	N	N	N	N	Y	19.00		15-Sept-21	29-Sept-21	08-Oct-21		
C19IMPACT_2481	Trinidad and Tobago	11-Sept-21	MoH	N	N	N	N	N	Y		6.00	17-Sept-21	29-Sept-21	08-Oct-21		21-Jan-22
C19IMPACT_2482	Trinidad and Tobago	12-Sept-21	MoH	N	N	N	N	N	Y		6.00	17-Sept-21	29-Sept-21	08-Oct-21		21-Jan-22
C19IMPACT_2483	Trinidad and Tobago	12-Sept-21	MoH	N	N	N	N	N	Y		4.00	17-Sept-21	29-Sept-21	08-Oct-21		21-Jan-22
C19IMPACT_2484	Trinidad and Tobago	12-Sept-21	MoH	N	N	N	N	N	Y		4.00	17-Sept-21	29-Sept-21	08-Oct-21		21-Jan-22
C19IMPACT_2485	Trinidad and Tobago	13-Sept-21	MoH	N	N	N	N	N	Y		7.00	17-Sept-21	29-Sept-21	08-Oct-21		
C19IMPACT_2486	Trinidad and Tobago	13-Sept-21	MoH	N	N	N	N	N	Y		3.00	17-Sept-21	29-Sept-21	08-Oct-21		21-Jan-22
C19IMPACT_2487	Trinidad and Tobago	13-Sept-21	CARPHA	N	N	Y	N	N	N	12.88		20-Sept-21	29-Sept-21	08-Oct-21	04-Oct-21	21-Jan-22
C19IMPACT_2488	Trinidad and Tobago	13-Sept-21	CARPHA	N	N	Y	N	N	N	18.69		20-Sept-21	29-Sept-21	08-Oct-21	04-Oct-21	21-Jan-22
C19IMPACT_2489	Trinidad and Tobago	14-Sept-21	MoH	N	N	N	N	Y	Y		7.00	17-Sept-21	29-Sept-21	08-Oct-21		21-Jan-22
C19IMPACT_2490	Trinidad and Tobago	14-Sept-21	MoH	N	N	N	N	N	Y	16.00		17-Sept-21	29-Sept-21	08-Oct-21		
C19IMPACT_2491	Trinidad and Tobago	14-Sept-21	MoH	N	N	N	N	N	Y	19.00		17-Sept-21	29-Sept-21	08-Oct-21		
C19IMPACT_2492	Trinidad and Tobago	14-Sept-21	MoH	N	N	N	N	N	Y	19.00		17-Sept-21	29-Sept-21	08-Oct-21		

C19IMPACT_2493	Trinidad and Tobago	14-Sept-21	MoH	N	N	N	N	N	Y	15.00		17-Sept-21	29-Sept-21	08-Oct-21		
C19IMPACT_2494	Trinidad and Tobago	14-Sept-21	CARPHA	N	N	Y	N	N	N	17.02		20-Sept-21	29-Sept-21	08-Oct-21	04-Oct-21	21-Jan-22
C19IMPACT_2495	Trinidad and Tobago	14-Sept-21	CARPHA	N	N	N	N	N	N	23.60		20-Sept-21	29-Sept-21	08-Oct-21	04-Oct-21	
C19IMPACT_2496	Trinidad and Tobago	15-Sept-21	MoH	N	N	N	N	N	Y		10.00	17-Sept-21	29-Sept-21	08-Oct-21		21-Jan-22
C19IMPACT_2497	Trinidad and Tobago	16-Sept-21	CARPHA	N	N	Y	N	N	Y	7.70		20-Sept-21	29-Sept-21	01-Oct-21	04-Oct-21	21-Jan-22
C19IMPACT_2498	Trinidad and Tobago	16-Sept-21	CARPHA	N	N	Y	N	Y	N	20.16		20-Sept-21	29-Sept-21	08-Oct-21	04-Oct-21	21-Jan-22
C19IMPACT_2499	Trinidad and Tobago	16-Sept-21	CARPHA	N	N	Y	N	N	N	8.15		20-Sept-21	29-Sept-21	08-Oct-21	04-Oct-21	21-Jan-22
C19IMPACT_2500	Saint Lucia	21-Aug-21	CARPHA	N	N	N	N	Y	N	15.00		22-Sept-21	30-Sept-21		06-Oct-21	21-Jan-22
C19IMPACT_2501	Saint Lucia	21-Aug-21	CARPHA	N	N	N	N	N	N	20.34		22-Sept-21	30-Sept-21		06-Oct-21	21-Jan-22
C19IMPACT_2502	Saint Lucia	21-Aug-21	CARPHA	N	N	N	N	N	N	27.93		22-Sept-21	30-Sept-21		06-Oct-21	
C19IMPACT_2503	Saint Lucia	22-Aug-21	CARPHA	N	N	N	N	N	N	15.24		22-Sept-21	30-Sept-21		06-Oct-21	21-Jan-22
C19IMPACT_2504	British Virgin Islands	26-Aug-21	CARPHA	N	N	N	N	N	N	13.25		22-Sept-21	30-Sept-21		06-Oct-21	21-Jan-22
C19IMPACT_2505	Saint Lucia	03-Sept-21	CARPHA	N	N	N	N	N	N	20.26		22-Sept-21	30-Sept-21		06-Oct-21	21-Jan-22
C19IMPACT_2506	British Virgin Islands	11-Sept-21	CARPHA	N	N	N	N	N	N	20.79		22-Sept-21	30-Sept-21		06-Oct-21	21-Jan-22
C19IMPACT_2507	British Virgin Islands	11-Sept-21	CARPHA	N	N	N	N	N	N	22.06		22-Sept-21	30-Sept-21		06-Oct-21	21-Jan-22
C19IMPACT_2508	British Virgin Islands	12-Sept-21	CARPHA	N	N	N	N	N	N	21.14		22-Sept-21	30-Sept-21		06-Oct-21	21-Jan-22
C19IMPACT_2509	Saint Vincent and the Grenadines	13-Sept-21	CARPHA	N	N	N	N	N	N	15.36		20-Sept-21	30-Sept-21		06-Oct-21	21-Jan-22
C19IMPACT_2510	Saint Vincent and the Grenadines	13-Sept-21	CARPHA	N	N	N	N	N	N	14.27		20-Sept-21	30-Sept-21		06-Oct-21	21-Jan-22
C19IMPACT_2511	Saint Vincent and the Grenadines	13-Sept-21	CARPHA	N	N	N	N	N	N	13.73		20-Sept-21	30-Sept-21		06-Oct-21	21-Jan-22
C19IMPACT_2512	Saint Vincent and the Grenadines	13-Sept-21	CARPHA	N	N	N	N	N	N	18.63		20-Sept-21	30-Sept-21		06-Oct-21	21-Jan-22
C19IMPACT_2513	Montserrat	13-Sept-21	CARPHA	N	N	N	N	N	N	11.62		20-Sept-21	30-Sept-21		06-Oct-21	21-Jan-22
C19IMPACT_2514	British Virgin Islands	13-Sept-21	CARPHA	N	N	N	N	N	N	19.85		22-Sept-21	30-Sept-21		06-Oct-21	21-Jan-22
C19IMPACT_2515	British Virgin Islands	13-Sept-21	CARPHA	N	N	N	N	N	N	17.57		22-Sept-21	30-Sept-21		06-Oct-21	
C19IMPACT_2516	British Virgin Islands	13-Sept-21	CARPHA	N	N	N	N	N	N	14.20		22-Sept-21	30-Sept-21		06-Oct-21	
C19IMPACT_2517	British Virgin Islands	13-Sept-21	CARPHA	N	N	N	N	N	N	19.02		22-Sept-21	30-Sept-21		06-Oct-21	
C19IMPACT_2518	British Virgin Islands	13-Sept-21	CARPHA	N	N	N	N	N	N	21.49		22-Sept-21	30-Sept-21		06-Oct-21	21-Jan-22
C19IMPACT_2519	Saint Lucia	14-Sept-21	CARPHA	N	N	N	N	N	N	14.65		22-Sept-21	30-Sept-21		06-Oct-21	21-Jan-22
C19IMPACT_2520	Saint Lucia	14-Sept-21	CARPHA	N	N	N	N	N	N	20.35		22-Sept-21	30-Sept-21		06-Oct-21	21-Jan-22
C19IMPACT_2521	Saint Lucia	14-Sept-21	CARPHA	N	N	N	N	N	N	14.81		22-Sept-21	30-Sept-21		06-Oct-21	21-Jan-22
C19IMPACT_2522	Saint Lucia	14-Sept-21	CARPHA	N	N	N	N	N	N	15.04		22-Sept-21	30-Sept-21		06-Oct-21	21-Jan-22
C19IMPACT_2523	Saint Vincent and the Grenadines	14-Sept-21	CARPHA	N	N	N	N	N	N	17.86		22-Sept-21	30-Sept-21		06-Oct-21	21-Jan-22
C19IMPACT_2524	Trinidad and Tobago	15-Sept-21	CARPHA	N	N	N	N	N	N	24.60		20-Sept-21	30-Sept-21	08-Oct-21	06-Oct-21	
C19IMPACT_2525	Saint Lucia	15-Sept-21	CARPHA	N	N	N	N	N	N	14.58		22-Sept-21	30-Sept-21		06-Oct-21	21-Jan-22
C19IMPACT_2526	Saint Vincent and the Grenadines	15-Sept-21	CARPHA	N	N	N	N	N	N	15.62		22-Sept-21	30-Sept-21		06-Oct-21	21-Jan-22
C19IMPACT_2527	Saint Vincent and the Grenadines	15-Sept-21	CARPHA	N	N	N	N	N	N	17.01		22-Sept-21	30-Sept-21		06-Oct-21	21-Jan-22
C19IMPACT_2528	Saint Vincent and the Grenadines	15-Sept-21	CARPHA	N	N	N	N	N	N	15.55		22-Sept-21	30-Sept-21		06-Oct-21	21-Jan-22
C19IMPACT_2529	Saint Vincent and the Grenadines	15-Sept-21	CARPHA	N	N	N	N	N	N	17.55		22-Sept-21	30-Sept-21		06-Oct-21	21-Jan-22
C19IMPACT_2530	Saint Vincent and the Grenadines	15-Sept-21	CARPHA	N	N	N	N	N	N	17.38		22-Sept-21	30-Sept-21		06-Oct-21	21-Jan-22
C19IMPACT_2531	Saint Vincent and the Grenadines	15-Sept-21	CARPHA	N	N	N	N	N	N	18.45		22-Sept-21	30-Sept-21		06-Oct-21	21-Jan-22
C19IMPACT_2532	British Virgin Islands	15-Sept-21	CARPHA	N	N	N	N	N	N	22.10		22-Sept-21	30-Sept-21		06-Oct-21	21-Jan-22
C19IMPACT_2533	Trinidad and Tobago	15-Sept-21	MoH	N	N	N	N	N	Y	11.60		22-Sept-21	30-Sept-21	08-Oct-21		
C19IMPACT_2534	Trinidad and Tobago	15-Sept-21	MoH	N	N	N	N	N	Y	14.00		22-Sept-21	30-Sept-21	08-Oct-21		
C19IMPACT_2535	Trinidad and Tobago	15-Sept-21	MoH	N	N	N	N	N	N		25.00	22-Sept-21	30-Sept-21	08-Oct-21		
C19IMPACT_2536	Trinidad and Tobago	15-Sept-21	MoH	N	N	N	N	N	Y	15.50		22-Sept-21	30-Sept-21	08-Oct-21		
C19IMPACT_2537	Saint Vincent and the Grenadines	16-Sept-21	CARPHA	N	N	N	N	N	N	18.83		22-Sept-21	30-Sept-21		06-Oct-21	21-Jan-22
C19IMPACT_2538	Trinidad and Tobago	16-Sept-21	MoH	N	N	N	N	N	N	15.00		22-Sept-21	30-Sept-21	08-Oct-21		21-Jan-22
C19IMPACT_2539	Trinidad and Tobago	16-Sept-21	MoH	N	N	N	N	N	N		7.00	22-Sept-21	30-Sept-21	08-Oct-21		
C19IMPACT_2540	Trinidad and Tobago	16-Sept-21	MoH	N	N	N	N	N	N		22.00	22-Sept-21	30-Sept-21	08-Oct-21		
C19IMPACT_2541	Trinidad and Tobago	17-Sept-21	CARPHA	N	N	Y	N	N	N	14.03		22-Sept-21	30-Sept-21	08-Oct-21	06-Oct-21	21-Jan-22
C19IMPACT_2542	Trinidad and Tobago	17-Sept-21	CARPHA	N	N	Y	N	N	N	13.15		22-Sept-21	30-Sept-21	08-Oct-21	06-Oct-21	21-Jan-22
C19IMPACT_2543	Trinidad and Tobago	17-Sept-21	MoH	N	N	N	N	Y	N		3.00	22-Sept-21	30-Sept-21	08-Oct-21		21-Jan-22
C19IMPACT_2544	Trinidad and Tobago	18-Sept-21	CARPHA	N	N	Y	N	N	N	13.72		22-Sept-21	30-Sept-21	08-Oct-21	06-Oct-21	21-Jan-22
C19IMPACT_2545	Trinidad and Tobago	18-Sept-21	MoH	N	N	N	N	N	N		9.00	22-Sept-21	30-Sept-21	08-Oct-21		21-Jan-22
C19IMPACT_2546	Saint Vincent and the Grenadines	18-Sept-21	CARPHA	N	N	N	N	N	N	16.33		27-Sept-21	30-Sept-21		06-Oct-21	21-Jan-22
C19IMPACT_2547	Saint Vincent and the Grenadines	18-Sept-21	CARPHA	N	N	N	N	N	N	15.97		27-Sept-21	30-Sept-21		06-Oct-21	21-Jan-22
C19IMPACT_2548	Saint Vincent and the Grenadines	18-Sept-21	CARPHA	N	N	N	N	N	N	16.84		27-Sept-21	30-Sept-21		06-Oct-21	21-Jan-22
C19IMPACT_2549	Saint Vincent and the Grenadines	18-Sept-21	CARPHA	N	N	N	N	N	N	13.86		27-Sept-21	30-Sept-21		06-Oct-21	21-Jan-22
C19IMPACT_2550	Saint Vincent and the Grenadines	19-Sept-21	CARPHA	N	N	N	N	N	N	18.82		27-Sept-21	30-Sept-21		06-Oct-21	21-Jan-22
C19IMPACT_2551	Trinidad and Tobago	20-Sept-21	CARPHA	N	N	Y	N	N	N	14.56		22-Sept-21	30-Sept-21	08-Oct-21	06-Oct-21	21-Jan-22
C19IMPACT_2552	Trinidad and Tobago	20-Sept-21	CARPHA	N	N	N	N	N	N	24.02		27-Sept-21	30-Sept-21	08-Oct-21	06-Oct-21	
C19IMPACT_2553	Trinidad and Tobago	20-Sept-21	CARPHA	N	N	N	N	N	N	16.69		27-Sept-21	30-Sept-21	08-Oct-21	06-Oct-21	
C19IMPACT_2554	Trinidad and Tobago	20-Sept-21	CARPHA	N	N	N	N	N	N	22.00		27-Sept-21	30-Sept-21	08-Oct-21	06-Oct-21	
C19IMPACT_2555	Trinidad and Tobago	20-Sept-21	CARPHA	N	N	Y	N	N	N	15.86		27-Sept-21	30-Sept-21	08-Oct-21	06-Oct-21	21-Jan-22
C19IMPACT_2556	Trinidad and Tobago	21-Sept-21	CARPHA	N	N	N	N	N	N	15.46		27-Sept-21	30-Sept-21	08-Oct-21	06-Oct-21	

C19IMPACT_2557	Trinidad and Tobago	21-Sept-21	CARPHA	N	N	Y	N	N	N	16.58		27-Sept-21	30-Sept-21	08-Oct-21	06-Oct-21	21-Jan-22
C19IMPACT_2558	Trinidad and Tobago	21-Sept-21	CARPHA	N	N	Y	N	Y	N	17.32		27-Sept-21	30-Sept-21	08-Oct-21	06-Oct-21	21-Jan-22
C19IMPACT_2559	Trinidad and Tobago	21-Sept-21	CARPHA	N	N	Y	N	Y	N	14.00		27-Sept-21	30-Sept-21	08-Oct-21	06-Oct-21	21-Jan-22
C19IMPACT_2560	Trinidad and Tobago	21-Sept-21	CARPHA	N	N	N	N	N	N	22.12		27-Sept-21	30-Sept-21	08-Oct-21	06-Oct-21	
C19IMPACT_2561	Trinidad and Tobago	21-Sept-21	CARPHA	N	N	Y	N	N	N	19.55		27-Sept-21	30-Sept-21	08-Oct-21	06-Oct-21	21-Jan-22
C19IMPACT_2562	Trinidad and Tobago	22-Sept-21	CARPHA	N	N	Y	N	N	N	12.92		27-Sept-21	30-Sept-21	08-Oct-21	06-Oct-21	21-Jan-22
C19IMPACT_2563	Trinidad and Tobago	22-Sept-21	CARPHA	N	N	Y	N	Y	N	16.78		27-Sept-21	30-Sept-21	08-Oct-21	06-Oct-21	21-Jan-22
C19IMPACT_2564	Trinidad and Tobago	22-Sept-21	CARPHA	N	N	Y	N	Y	N	14.85		27-Sept-21	30-Sept-21	08-Oct-21	06-Oct-21	21-Jan-22
C19IMPACT_2565	Trinidad and Tobago	22-Sept-21	CARPHA	N	N	N	N	N	N	23.21		27-Sept-21	30-Sept-21	08-Oct-21	06-Oct-21	
C19IMPACT_2566	Trinidad and Tobago	12-Sept-21	MoH	N	N	N	N	N	Y		15.08	22-Sept-21	04-Oct-21	16-Oct-21		
C19IMPACT_2567	Trinidad and Tobago	12-Sept-21	MoH	N	N	N	N	N	Y		6.12	22-Sept-21	04-Oct-21	16-Oct-21		11-Nov-21
C19IMPACT_2568	Trinidad and Tobago	13-Sept-21	MoH	N	N	N	N	N	Y		8.85	22-Sept-21	04-Oct-21	16-Oct-21		
C19IMPACT_2569	Trinidad and Tobago	13-Sept-21	MoH	N	N	N	N	N	Y		11.81	22-Sept-21	04-Oct-21	16-Oct-21		
C19IMPACT_2570	Trinidad and Tobago	13-Sept-21	MoH	N	N	N	N	N	Y		4.38	22-Sept-21	04-Oct-21	16-Oct-21		11-Nov-21
C19IMPACT_2571	Trinidad and Tobago	14-Sept-21	MoH	N	N	N	N	N	Y		14.92	22-Sept-21	04-Oct-21	16-Oct-21		11-Nov-21
C19IMPACT_2572	Trinidad and Tobago	14-Sept-21	MoH	N	N	N	N	N	Y		11.55	22-Sept-21	04-Oct-21	16-Oct-21		
C19IMPACT_2573	Trinidad and Tobago	14-Sept-21	MoH	N	N	N	N	N	Y		14.02	22-Sept-21	04-Oct-21	16-Oct-21		
C19IMPACT_2574	Trinidad and Tobago	15-Sept-21	MoH	N	N	N	N	N	Y			22-Sept-21	04-Oct-21	16-Oct-21		
C19IMPACT_2575	Trinidad and Tobago	15-Sept-21	MoH	N	N	N	N	N	N	17.00		22-Sept-21	04-Oct-21	16-Oct-21		11-Nov-21
C19IMPACT_2576	Trinidad and Tobago	15-Sept-21	MoH	N	N	N	N	N	Y		9.18	22-Sept-21	04-Oct-21	16-Oct-21		11-Nov-21
C19IMPACT_2577	Trinidad and Tobago	15-Sept-21	MoH	N	N	N	N	N	Y		2.94	22-Sept-21	04-Oct-21	16-Oct-21		11-Nov-21
C19IMPACT_2578	Trinidad and Tobago	15-Sept-21	MoH	N	N	N	N	N	Y		8.42	22-Sept-21	04-Oct-21	16-Oct-21		11-Nov-21
C19IMPACT_2579	Trinidad and Tobago	16-Sept-21	MoH	N	N	N	N	N	N	16.00		22-Sept-21	04-Oct-21	16-Oct-21		11-Nov-21
C19IMPACT_2580	Trinidad and Tobago	16-Sept-21	MoH	N	N	N	N	N	N	18.00		22-Sept-21	04-Oct-21	16-Oct-21		11-Nov-21
C19IMPACT_2581	Trinidad and Tobago	16-Sept-21	MoH	N	N	N	N	N	N	19.00		22-Sept-21	04-Oct-21	16-Oct-21		11-Nov-21
C19IMPACT_2582	Trinidad and Tobago	16-Sept-21	MoH	N	N	N	N	N	N	13.00		22-Sept-21	04-Oct-21	16-Oct-21		11-Nov-21
C19IMPACT_2583	Trinidad and Tobago	16-Sept-21	MoH	N	N	N	N	N	N	18.00		22-Sept-21	04-Oct-21	16-Oct-21		11-Nov-21
C19IMPACT_2584	Trinidad and Tobago	16-Sept-21	MoH	N	N	N	N	N	N	15.00		22-Sept-21	04-Oct-21	16-Oct-21		11-Nov-21
C19IMPACT_2585	Trinidad and Tobago	16-Sept-21	MoH	N	N	N	N	N	Y		7.69	22-Sept-21	04-Oct-21	16-Oct-21		11-Nov-21
C19IMPACT_2586	Trinidad and Tobago	16-Sept-21	MoH	N	N	N	N	N	Y		3.39	22-Sept-21	04-Oct-21	16-Oct-21		11-Nov-21
C19IMPACT_2587	Trinidad and Tobago	17-Sept-21	MoH	N	N	N	N	N	N	17.00		22-Sept-21	04-Oct-21	16-Oct-21		11-Nov-21
C19IMPACT_2588	Trinidad and Tobago	17-Sept-21	MoH	N	N	N	N	N	N	17.00		22-Sept-21	04-Oct-21	16-Oct-21		11-Nov-21
C19IMPACT_2589	Trinidad and Tobago	17-Sept-21	MoH	N	N	N	N	N	N	17.00		22-Sept-21	04-Oct-21	16-Oct-21		
C19IMPACT_2590	Trinidad and Tobago	17-Sept-21	MoH	N	N	N	N	N	N	15.00		22-Sept-21	04-Oct-21	16-Oct-21		11-Nov-21
C19IMPACT_2591	Trinidad and Tobago	17-Sept-21	MoH	N	N	N	N	N	N	22.00		22-Sept-21	04-Oct-21	16-Oct-21		11-Nov-21
C19IMPACT_2592	Trinidad and Tobago	17-Sept-21	MoH	N	N	N	N	N	Y		7.17	22-Sept-21	04-Oct-21	16-Oct-21		11-Nov-21
C19IMPACT_2593	Trinidad and Tobago	17-Sept-21	MoH	N	N	N	N	N	Y		10.39	22-Sept-21	04-Oct-21	16-Oct-21		11-Nov-21
C19IMPACT_2594	Trinidad and Tobago	17-Sept-21	MoH	N	N	N	N	N	Y		13.47	22-Sept-21	04-Oct-21	16-Oct-21		
C19IMPACT_2595	Trinidad and Tobago	18-Sept-21	MoH	N	N	N	N	N	Y		13.19	22-Sept-21	04-Oct-21	16-Oct-21		11-Nov-21
C19IMPACT_2596	Trinidad and Tobago	19-Sept-21	MoH	N	N	N	N	N	Y		8.00	22-Sept-21	04-Oct-21	16-Oct-21		11-Nov-21
C19IMPACT_2597	Trinidad and Tobago	20-Sept-21	MoH	N	N	N	N	N	N	19.00		30-Sept-21	04-Oct-21	16-Oct-21		
C19IMPACT_2598	Saint Vincent and the Grenadines	20-Sept-21	CARPHA	N	N	N	N	N	N	13.19		01-Oct-21	04-Oct-21		10-Oct-21	11-Nov-21
C19IMPACT_2599	Trinidad and Tobago	20-Sept-21	MoH	N	N	N	N	N	Y		9.64	30-Sept-21	04-Oct-21	16-Oct-21		11-Nov-21
C19IMPACT_2600	Trinidad and Tobago	20-Sept-21	MoH	N	N	N	N	N	Y		3.60	30-Sept-21	04-Oct-21	16-Oct-21		11-Nov-21
C19IMPACT_2601	Trinidad and Tobago	20-Sept-21	MoH	N	N	N	N	N	N		9.00	30-Sept-21	04-Oct-21	16-Oct-21		11-Nov-21
C19IMPACT_2602	Trinidad and Tobago	20-Sept-21	MoH	N	N	N	N	N	N	18.00		30-Sept-21	04-Oct-21	16-Oct-21		
C19IMPACT_2603	Trinidad and Tobago	20-Sept-21	MoH	N	N	N	N	N	N	17.00		30-Sept-21	04-Oct-21	16-Oct-21		
C19IMPACT_2604	Trinidad and Tobago	20-Sept-21	MoH	N	N	N	N	N	N	16.00		30-Sept-21	04-Oct-21	16-Oct-21		
C19IMPACT_2605	Saint Vincent and the Grenadines	20-Sept-21	CARPHA	N	N	N	N	N	N	14.86		01-Oct-21	04-Oct-21		10-Oct-21	11-Nov-21
C19IMPACT_2606	Saint Vincent and the Grenadines	20-Sept-21	CARPHA	N	N	N	N	N	N	14.58		01-Oct-21	04-Oct-21		10-Oct-21	11-Nov-21
C19IMPACT_2607	Trinidad and Tobago	20-Sept-21	MoH	N	N	N	N	N	N	29.00		22-Sept-21	04-Oct-21	16-Oct-21		
C19IMPACT_2608	Trinidad and Tobago	20-Sept-21	MoH	N	N	N	N	N	N	18.00		22-Sept-21	04-Oct-21	16-Oct-21		11-Nov-21
C19IMPACT_2609	Trinidad and Tobago	20-Sept-21	MoH	N	N	N	N	N	N	16.00		22-Sept-21	04-Oct-21	16-Oct-21		11-Nov-21
C19IMPACT_2610	Trinidad and Tobago	20-Sept-21	MoH	N	N	N	N	N	N	15.00		22-Sept-21	04-Oct-21	16-Oct-21		11-Nov-21
C19IMPACT_2611	Trinidad and Tobago	20-Sept-21	MoH	N	N	N	N	N	N	24.00		22-Sept-21	04-Oct-21	16-Oct-21		11-Nov-21
C19IMPACT_2612	Trinidad and Tobago	20-Sept-21	MoH	N	N	N	N	N	N	17.00		30-Sept-21	04-Oct-21	16-Oct-21		
C19IMPACT_2613	Trinidad and Tobago	20-Sept-21	MoH	N	N	N	N	N	N	20.00		30-Sept-21	04-Oct-21	16-Oct-21		
C19IMPACT_2614	Trinidad and Tobago	21-Sept-21	MoH	N	N	N	N	N	Y		11.55	30-Sept-21	04-Oct-21	16-Oct-21		
C19IMPACT_2615	Trinidad and Tobago	21-Sept-21	MoH	N	N	N	N	N	Y		15.68	30-Sept-21	04-Oct-21	16-Oct-21		
C19IMPACT_2616	Trinidad and Tobago	21-Sept-21	MoH	N	N	N	N	N	N		8.00	30-Sept-21	04-Oct-21	16-Oct-21		11-Nov-21
C19IMPACT_2617	Trinidad and Tobago	21-Sept-21	MoH	N	N	N	N	N	N			22-Sept-21	04-Oct-21	16-Oct-21		
C19IMPACT_2618	Trinidad and Tobago	23-Sept-21	MoH	N	N	N	N	N	N	20.00		27-Sept-21	04-Oct-21	16-Oct-21		
C19IMPACT_2619	Trinidad and Tobago	24-Sept-21	MoH	N	N	N	N	N	N	20.00		30-Sept-21	04-Oct-21	16-Oct-21		11-Nov-21
C19IMPACT_2620	Trinidad and Tobago	24-Sept-21	MoH	N	N	N	N	N	N	15.00		30-Sept-21	04-Oct-21	16-Oct-21		11-Nov-21

C19IMPACT_2621	Trinidad and Tobago	24-Sept-21	MoH	N	N	N	N	N	N	16.00		30-Sept-21	04-Oct-21	16-Oct-21		
C19IMPACT_2622	Trinidad and Tobago	24-Sept-21	MoH	N	N	N	N	N	N	19.00		30-Sept-21	04-Oct-21	16-Oct-21		
C19IMPACT_2623	Montserrat	24-Sept-21	CARPHA	N	N	N	N	N	N	19.06		01-Oct-21	04-Oct-21		10-Oct-21	11-Nov-21
C19IMPACT_2624	Trinidad and Tobago	27-Sept-21	MoH	N	N	N	N	N	Y		4.00	30-Sept-21	04-Oct-21	16-Oct-21		11-Nov-21
C19IMPACT_2625	Trinidad and Tobago	27-Sept-21	MoH	N	N	N	N	N	N		4.00	30-Sept-21	04-Oct-21	16-Oct-21		11-Nov-21
C19IMPACT_2626	Trinidad and Tobago	27-Sept-21	MoH	N	N	N	N	N	N		6.00	30-Sept-21	04-Oct-21	16-Oct-21		11-Nov-21
C19IMPACT_2627	Trinidad and Tobago	28-Sept-21	CARPHA	N	N	Y	N	N	N	15.33		01-Oct-21	04-Oct-21	16-Oct-21	16-Oct-21	11-Nov-21
C19IMPACT_2628	Trinidad and Tobago	28-Sept-21	CARPHA	N	N	Y	N	Y	N	17.38		01-Oct-21	04-Oct-21	16-Oct-21	10-Oct-21	11-Nov-21
C19IMPACT_2629	Trinidad and Tobago	28-Sept-21	CARPHA	N	N	Y	N	N	N	15.03		01-Oct-21	04-Oct-21	16-Oct-21	10-Oct-21	11-Nov-21
C19IMPACT_2630	Trinidad and Tobago	28-Sept-21	CARPHA	N	N	Y	N	N	N	15.93		01-Oct-21	04-Oct-21	16-Oct-21	10-Oct-21	11-Nov-21
C19IMPACT_2631	Trinidad and Tobago	29-Sept-21	CARPHA	N	N	Y	N	Y	N	16.56		01-Oct-21	04-Oct-21	16-Oct-21	16-Oct-21	11-Nov-21
C19IMPACT_2632	Trinidad and Tobago	21-Sept-21	MoH	N	N	N	N	N	N	27.00		30-Sept-21	05-Oct-21	08-Oct-21		
C19IMPACT_2633	Trinidad and Tobago	21-Sept-21	MoH	N	N	N	N	N	Y		3.47	30-Sept-21	05-Oct-21	08-Oct-21		11-Nov-21
C19IMPACT_2634	Trinidad and Tobago	21-Sept-21	MoH	N	N	N	N	N	Y		6.24	30-Sept-21	05-Oct-21	08-Oct-21		11-Nov-21
C19IMPACT_2635	Trinidad and Tobago	21-Sept-21	MoH	N	N	N	N	N	N	15.00		30-Sept-21	05-Oct-21	08-Oct-21		11-Nov-21
C19IMPACT_2636	Trinidad and Tobago	22-Sept-21	MoH	N	N	N	N	N	N	17.00		30-Sept-21	05-Oct-21	08-Oct-21		11-Nov-21
C19IMPACT_2637	Trinidad and Tobago	22-Sept-21	MoH	N	N	N	N	N	N	16.00		30-Sept-21	05-Oct-21	08-Oct-21		11-Nov-21
C19IMPACT_2638	Trinidad and Tobago	22-Sept-21	MoH	N	N	N	N	N	Y		3.86	30-Sept-21	05-Oct-21	08-Oct-21		11-Nov-21
C19IMPACT_2639	Trinidad and Tobago	22-Sept-21	MoH	N	N	N	N	N	Y		5.66	30-Sept-21	05-Oct-21	08-Oct-21		11-Nov-21
C19IMPACT_2640	Trinidad and Tobago	23-Sept-21	MoH	N	N	N	N	N	N	16.00		30-Sept-21	05-Oct-21	08-Oct-21		11-Nov-21
C19IMPACT_2641	Trinidad and Tobago	24-Sept-21	MoH	N	N	N	N	N	N	18.00		30-Sept-21	05-Oct-21	08-Oct-21		11-Nov-21
C19IMPACT_2642	Trinidad and Tobago	24-Sept-21	MoH	N	N	N	N	N	Y		28.23	30-Sept-21	05-Oct-21	08-Oct-21		
C19IMPACT_2643	Trinidad and Tobago	25-Sept-21	MoH	N	N	N	N	N	N	19.00		30-Sept-21	05-Oct-21	08-Oct-21		11-Nov-21
C19IMPACT_2644	Trinidad and Tobago	27-Sept-21	MoH	N	N	N	N	N	Y	14.00		30-Sept-21	05-Oct-21	08-Oct-21		11-Nov-21
C19IMPACT_2645	Trinidad and Tobago	27-Sept-21	MoH	N	N	N	N	N	Y		8.00	01-Oct-21	05-Oct-21	08-Oct-21		11-Nov-21
C19IMPACT_2646	Trinidad and Tobago	27-Sept-21	MoH	N	N	N	N	N	N	16.00		01-Oct-21	05-Oct-21	08-Oct-21		11-Nov-21
C19IMPACT_2647	Trinidad and Tobago	27-Sept-21	MoH	N	N	N	N	N	N	14.00		01-Oct-21	05-Oct-21	08-Oct-21		
C19IMPACT_2648	Trinidad and Tobago	27-Sept-21	MoH	N	N	N	N	N	N	20.00		01-Oct-21	05-Oct-21	08-Oct-21		
C19IMPACT_2649	Trinidad and Tobago	28-Sept-21	MoH	N	N	N	N	N	N	19.00		01-Oct-21	05-Oct-21	08-Oct-21		11-Nov-21
C19IMPACT_2650	Trinidad and Tobago	28-Sept-21	MoH	N	N	N	N	N	N	19.00		01-Oct-21	05-Oct-21	08-Oct-21		11-Nov-21
C19IMPACT_2651	Trinidad and Tobago	28-Sept-21	MoH	N	N	N	N	N	N	18.00		01-Oct-21	05-Oct-21	08-Oct-21		11-Nov-21
C19IMPACT_2652	Trinidad and Tobago	28-Sept-21	MoH	N	N	N	N	N	N	17.00		01-Oct-21	05-Oct-21	08-Oct-21		11-Nov-21
C19IMPACT_2653	Trinidad and Tobago	28-Sept-21	MoH	N	N	N	N	N	N	19.00		01-Oct-21	05-Oct-21	08-Oct-21		11-Nov-21
C19IMPACT_2654	British Virgin Islands	16-Sept-21	CARPHA	N	N	N	N	N	N	20.19		07-Oct-21	12-Oct-21		14-Oct-21	11-Nov-21
C19IMPACT_2655	British Virgin Islands	19-Sept-21	CARPHA	N	N	N	N	N	N	16.97		07-Oct-21	12-Oct-21		14-Oct-21	11-Nov-21
C19IMPACT_2656	British Virgin Islands	19-Sept-21	CARPHA	N	N	N	N	N	N	19.75		07-Oct-21	12-Oct-21		14-Oct-21	11-Nov-21
C19IMPACT_2657	British Virgin Islands	22-Sept-21	CARPHA	N	N	N	N	N	N	16.16		07-Oct-21	12-Oct-21		14-Oct-21	11-Nov-21
C19IMPACT_2658	British Virgin Islands	22-Sept-21	CARPHA	N	N	N	N	N	N	15.43		07-Oct-21	12-Oct-21		14-Oct-21	11-Nov-21
C19IMPACT_2659	British Virgin Islands	22-Sept-21	CARPHA	N	N	N	N	N	N	14.62		07-Oct-21	12-Oct-21		14-Oct-21	11-Nov-21
C19IMPACT_2660	British Virgin Islands	22-Sept-21	CARPHA	N	N	N	N	N	N	19.67		07-Oct-21	12-Oct-21		14-Oct-21	11-Nov-21
C19IMPACT_2661	British Virgin Islands	22-Sept-21	CARPHA	N	N	N	N	N	N	19.37		07-Oct-21	12-Oct-21		14-Oct-21	11-Nov-21
C19IMPACT_2662	British Virgin Islands	22-Sept-21	CARPHA	N	N	N	N	N	Y	16.52		07-Oct-21	12-Oct-21		21-Oct-21	11-Nov-21
C19IMPACT_2663	British Virgin Islands	24-Sept-21	CARPHA	N	N	N	N	N	N	16.25		07-Oct-21	12-Oct-21		14-Oct-21	11-Nov-21
C19IMPACT_2664	British Virgin Islands	24-Sept-21	CARPHA	N	N	N	N	N	N	16.72		07-Oct-21	12-Oct-21		14-Oct-21	11-Nov-21
C19IMPACT_2665	British Virgin Islands	24-Sept-21	CARPHA	N	N	N	N	N	N	20.17		07-Oct-21	12-Oct-21		14-Oct-21	11-Nov-21
C19IMPACT_2666	British Virgin Islands	24-Sept-21	CARPHA	N	N	N	N	N	N	19.89		07-Oct-21	12-Oct-21		14-Oct-21	11-Nov-21
C19IMPACT_2667	Saint Vincent and the Grenadines	26-Sept-21	CARPHA	N	N	N	N	N	N	16.44		05-Oct-21	12-Oct-21		14-Oct-21	11-Nov-21
C19IMPACT_2668	British Virgin Islands	26-Sept-21	CARPHA	N	N	N	N	N	N	15.18		07-Oct-21	12-Oct-21		14-Oct-21	
C19IMPACT_2669	Trinidad and Tobago	26-Sept-21	MoH	N	N	N	N	N	Y		14.75	01-Oct-21	12-Oct-21	16-Oct-21		11-Nov-21
C19IMPACT_2670	Saint Vincent and the Grenadines	27-Sept-21	CARPHA	N	N	N	N	N	N	11.90		05-Oct-21	12-Oct-21		14-Oct-21	
C19IMPACT_2671	Saint Vincent and the Grenadines	27-Sept-21	CARPHA	N	N	N	N	N	N	17.07		05-Oct-21	12-Oct-21		14-Oct-21	11-Nov-21
C19IMPACT_2672	Saint Vincent and the Grenadines	27-Sept-21	CARPHA	N	N	N	N	N	N	13.17		05-Oct-21	12-Oct-21		14-Oct-21	11-Nov-21
C19IMPACT_2673	Saint Vincent and the Grenadines	27-Sept-21	CARPHA	N	N	N	N	N	N	16.97		05-Oct-21	12-Oct-21		14-Oct-21	11-Nov-21
C19IMPACT_2674	Trinidad and Tobago	27-Sept-21	MoH	N	N	N	N	N	Y		12.08	05-Oct-21	12-Oct-21	16-Oct-21		
C19IMPACT_2675	Trinidad and Tobago	27-Sept-21	MoH	N	N	N	N	N	Y		5.02	05-Oct-21	12-Oct-21	16-Oct-21		11-Nov-21
C19IMPACT_2676	Saint Vincent and the Grenadines	27-Sept-21	CARPHA	N	N	N	N	N	N	15.97		07-Oct-21	12-Oct-21		14-Oct-21	11-Nov-21
C19IMPACT_2677	Saint Vincent and the Grenadines	27-Sept-21	CARPHA	N	N	N	N	N	N	16.17		07-Oct-21	12-Oct-21		14-Oct-21	11-Nov-21
C19IMPACT_2678	Trinidad and Tobago	27-Sept-21	MoH	N	N	N	N	N	N	15.00		07-Oct-21	12-Oct-21	16-Oct-21		11-Nov-21
C19IMPACT_2679	Trinidad and Tobago	27-Sept-21	MoH	N	N	N	N	N	N	20.00		07-Oct-21	12-Oct-21	16-Oct-21		11-Nov-21
C19IMPACT_2680	Trinidad and Tobago	27-Sept-21	MoH	N	N	N	N	N	N	18.00		07-Oct-21	12-Oct-21	16-Oct-21		11-Nov-21
C19IMPACT_2681	Trinidad and Tobago	27-Sept-21	MoH	N	N	N	N	N	N	17.00		01-Oct-21	12-Oct-21	16-Oct-21		
C19IMPACT_2682	Trinidad and Tobago	27-Sept-21	MoH	N	N	N	N	N	N	19.00		01-Oct-21	12-Oct-21	16-Oct-21		11-Nov-21
C19IMPACT_2683	Trinidad and Tobago	28-Sept-21	MoH	N	N	N	N	N	Y		8.99	05-Oct-21	12-Oct-21	16-Oct-21		11-Nov-21
C19IMPACT_2684	Trinidad and Tobago	28-Sept-21	MoH	N	N	N	N	N	Y		9.93	05-Oct-21	12-Oct-21	16-Oct-21		11-Nov-21

C19IMPACT_2685	Trinidad and Tobago	28-Sept-21	MoH	N	N	N	N	N	Y		4.67	05-Oct-21	12-Oct-21	16-Oct-21		11-Nov-21
C19IMPACT_2686	Trinidad and Tobago	28-Sept-21	MoH	N	N	N	N	N	N		11.00	05-Oct-21	12-Oct-21	16-Oct-21		
C19IMPACT_2687	Trinidad and Tobago	28-Sept-21	MoH	N	N	N	N	N	N		8.00	05-Oct-21	12-Oct-21	16-Oct-21		
C19IMPACT_2688	Trinidad and Tobago	28-Sept-21	MoH	N	N	N	N	N	N	20.00		07-Oct-21	12-Oct-21	16-Oct-21		11-Nov-21
C19IMPACT_2689	Trinidad and Tobago	28-Sept-21	MoH	N	N	N	N	N	N	18.00		08-Oct-21	12-Oct-21	16-Oct-21		11-Nov-21
C19IMPACT_2690	Trinidad and Tobago	28-Sept-21	MoH	N	N	N	N	N	N	17.00		08-Oct-21	12-Oct-21	16-Oct-21		
C19IMPACT_2691	Trinidad and Tobago	29-Sept-21	MoH	N	N	N	N	N	Y	17.00		05-Oct-21	12-Oct-21	16-Oct-21		11-Nov-21
C19IMPACT_2692	Trinidad and Tobago	29-Sept-21	MoH	N	N	N	N	N	Y		13.18	05-Oct-21	12-Oct-21	16-Oct-21		
C19IMPACT_2693	Trinidad and Tobago	29-Sept-21	MoH	N	N	N	N	N	Y		5.56	05-Oct-21	12-Oct-21	16-Oct-21		11-Nov-21
C19IMPACT_2694	Trinidad and Tobago	29-Sept-21	MoH	N	N	N	N	N	N	20.00		07-Oct-21	12-Oct-21	16-Oct-21		11-Nov-21
C19IMPACT_2695	Trinidad and Tobago	29-Sept-21	MoH	N	N	N	N	N	N	20.00		07-Oct-21	12-Oct-21	16-Oct-21		11-Nov-21
C19IMPACT_2696	Trinidad and Tobago	30-Sept-21	CARPHA	N	N	Y	N	N	N	10.74		05-Oct-21	12-Oct-21	16-Oct-21	16-Oct-21	11-Nov-21
C19IMPACT_2697	Trinidad and Tobago	30-Sept-21	MoH	N	N	N	N	N	N	16.00		05-Oct-21	12-Oct-21	16-Oct-21		11-Nov-21
C19IMPACT_2698	Trinidad and Tobago	30-Sept-21	MoH	N	N	N	N	N	N	15.00		08-Oct-21	12-Oct-21	16-Oct-21		11-Nov-21
C19IMPACT_2699	Trinidad and Tobago	30-Sept-21	MoH	N	N	N	N	N	N	20.00		08-Oct-21	12-Oct-21	16-Oct-21		11-Nov-21
C19IMPACT_2700	Trinidad and Tobago	01-Oct-21	CARPHA	N	N	Y	N	N	N	16.43		07-Oct-21	12-Oct-21	16-Oct-21	14-Oct-21	11-Nov-21
C19IMPACT_2701	Trinidad and Tobago	01-Oct-21	CARPHA	N	N	Y	N	N	N	13.80		07-Oct-21	12-Oct-21	16-Oct-21	16-Oct-21	11-Nov-21
C19IMPACT_2702	Trinidad and Tobago	02-Oct-21	MoH	N	N	N	N	N	Y	12.00		05-Oct-21	12-Oct-21	22-Oct-21		11-Nov-21
C19IMPACT_2703	Trinidad and Tobago	02-Oct-21	MoH	N	N	N	N	N	Y	18.00		05-Oct-21	12-Oct-21	16-Oct-21		11-Nov-21
C19IMPACT_2704	Trinidad and Tobago	02-Oct-21	CARPHA	N	N	Y	N	N	N	21.45		07-Oct-21	12-Oct-21	16-Oct-21	16-Oct-21	11-Nov-21
C19IMPACT_2705	Trinidad and Tobago	02-Oct-21	CARPHA	N	N	N	N	N	N	24.69		07-Oct-21	12-Oct-21	16-Oct-21	14-Oct-21	
C19IMPACT_2706	Trinidad and Tobago	02-Oct-21	MoH	N	N	N	N	N	N	19.00		07-Oct-21	12-Oct-21	16-Oct-21		11-Nov-21
C19IMPACT_2707	Trinidad and Tobago	02-Oct-21	MoH	N	N	N	N	N	N	19.00		07-Oct-21	12-Oct-21	16-Oct-21		
C19IMPACT_2708	Trinidad and Tobago	02-Oct-21	MoH	N	N	N	N	N	N	19.00		07-Oct-21	12-Oct-21	16-Oct-21		
C19IMPACT_2709	Trinidad and Tobago	03-Oct-21	CARPHA	N	N	N	N	N	N	24.32		07-Oct-21	12-Oct-21	16-Oct-21	14-Oct-21	
C19IMPACT_2710	Trinidad and Tobago	03-Oct-21	MoH	N	N	N	N	N	Y		9.03	08-Oct-21	12-Oct-21	16-Oct-21		11-Nov-21
C19IMPACT_2711	Trinidad and Tobago	03-Oct-21	MoH	N	N	N	N	N	N	16.00		08-Oct-21	12-Oct-21	16-Oct-21		11-Nov-21
C19IMPACT_2712	Trinidad and Tobago	04-Oct-21	CARPHA	N	N	Y	N	N	N	21.54		07-Oct-21	12-Oct-21	16-Oct-21	16-Oct-21	11-Nov-21
C19IMPACT_2713	Trinidad and Tobago	04-Oct-21	CARPHA	N	N	Y	N	Y	N	14.47		07-Oct-21	12-Oct-21	16-Oct-21	16-Oct-21	11-Nov-21
C19IMPACT_2714	Trinidad and Tobago	04-Oct-21	MoH	N	N	N	N	N	N	20.00		07-Oct-21	12-Oct-21	16-Oct-21		
C19IMPACT_2715	Trinidad and Tobago	04-Oct-21	MoH	N	N	N	N	N	N	20.00		07-Oct-21	12-Oct-21	16-Oct-21		11-Nov-21
C19IMPACT_2716	Trinidad and Tobago	04-Oct-21	MoH	N	N	N	N	N	Y		16.62	08-Oct-21	12-Oct-21	16-Oct-21		
C19IMPACT_2717	Trinidad and Tobago	04-Oct-21	MoH	N	N	N	N	N	Y		9.05	08-Oct-21	12-Oct-21	16-Oct-21		11-Nov-21
C19IMPACT_2718	Trinidad and Tobago	04-Oct-21	MoH	N	N	N	N	N	Y		4.04	08-Oct-21	12-Oct-21	16-Oct-21		11-Nov-21
C19IMPACT_2719	Trinidad and Tobago	04-Oct-21	MoH	N	N	N	N	N	Y		13.07	08-Oct-21	12-Oct-21	16-Oct-21		11-Nov-21
C19IMPACT_2720	Trinidad and Tobago	04-Oct-21	CARPHA	N	N	Y	N	N	N	13.94		11-Oct-21	12-Oct-21	16-Oct-21	16-Oct-21	11-Nov-21
C19IMPACT_2721	Trinidad and Tobago	05-Oct-21	MoH	N	N	N	N	Y	N	16.00		07-Oct-21	12-Oct-21	16-Oct-21		11-Nov-21
C19IMPACT_2722	Trinidad and Tobago	05-Oct-21	CARPHA	N	N	Y	N	N	N	16.22		07-Oct-21	12-Oct-21	16-Oct-21	16-Oct-21	11-Nov-21
C19IMPACT_2723	Trinidad and Tobago	05-Oct-21	MoH	N	N	N	N	Y	N	18.00		07-Oct-21	12-Oct-21	16-Oct-21		
C19IMPACT_2724	Trinidad and Tobago	05-Oct-21	CARPHA	N	N	Y	N	N	N	14.57		11-Oct-21	12-Oct-21	16-Oct-21	16-Oct-21	11-Nov-21
C19IMPACT_2725	Trinidad and Tobago	05-Oct-21	CARPHA	N	N	Y	N	Y	N	16.86		11-Oct-21	12-Oct-21	16-Oct-21	14-Oct-21	11-Nov-21
C19IMPACT_2726	Trinidad and Tobago	05-Oct-21	CARPHA	N	N	Y	N	Y	N	20.05		11-Oct-21	12-Oct-21	16-Oct-21	16-Oct-21	11-Nov-21
C19IMPACT_2727	Trinidad and Tobago	06-Oct-21	MoH	N	N	N	N	Y	N	19.10		08-Oct-21	12-Oct-21	16-Oct-21		11-Nov-21
C19IMPACT_2728	Trinidad and Tobago	06-Oct-21	MoH	N	N	N	N	N	N	20.00		07-Oct-21	12-Oct-21	16-Oct-21		11-Nov-21
C19IMPACT_2729	Trinidad and Tobago	06-Oct-21	MoH	N	N	N	N	N	N	16.73		07-Oct-21	12-Oct-21	16-Oct-21		11-Nov-21
C19IMPACT_2730	Trinidad and Tobago	06-Oct-21	MoH	N	N	N	N	N	N	14.71		07-Oct-21	12-Oct-21	16-Oct-21		
C19IMPACT_2731	Trinidad and Tobago	06-Oct-21	MoH	N	N	N	N	N	N	17.41		07-Oct-21	12-Oct-21	16-Oct-21		
C19IMPACT_2732	Trinidad and Tobago	06-Oct-21	MoH	N	N	N	N	N	N		14.00	08-Oct-21	12-Oct-21	16-Oct-21		11-Nov-21
C19IMPACT_2733	Trinidad and Tobago	06-Oct-21	MoH	N	N	N	N	N	N		13.00	08-Oct-21	12-Oct-21	16-Oct-21		11-Nov-21
C19IMPACT_2734	Trinidad and Tobago	06-Oct-21	MoH	N	N	N	N	N	N	16.20		08-Oct-21	12-Oct-21	16-Oct-21		
C19IMPACT_2735	Trinidad and Tobago	06-Oct-21	MoH	N	N	N	N	N	N	15.80		08-Oct-21	12-Oct-21	16-Oct-21		11-Nov-21
C19IMPACT_2736	Trinidad and Tobago	06-Oct-21	CARPHA	N	N	Y	N	N	N	14.15		11-Oct-21	12-Oct-21	16-Oct-21	16-Oct-21	11-Nov-21
C19IMPACT_2737	Trinidad and Tobago	06-Oct-21	MoH	N	N	N	N	N	N	15.00		11-Oct-21	12-Oct-21	16-Oct-21		11-Nov-21
C19IMPACT_2738	Trinidad and Tobago	06-Oct-21	MoH	N	N	N	N	N	N		25.00	08-Oct-21	12-Oct-21	16-Oct-21		
C19IMPACT_2739	British Virgin Islands	30-Sept-21	CARPHA	N	N	N	N	N	N	20.35		12-Oct-21	15-Oct-21		21-Oct-21	11-Nov-21
C19IMPACT_2740	British Virgin Islands	02-Oct-21	CARPHA	N	N	N	N	N	N	15.51		12-Oct-21	15-Oct-21		21-Oct-21	11-Nov-21
C19IMPACT_2741	British Virgin Islands	02-Oct-21	CARPHA	N	N	N	N	N	N	15.01		12-Oct-21	15-Oct-21		21-Oct-21	11-Nov-21
C19IMPACT_2742	Trinidad and Tobago	06-Oct-21	MoH	N	N	N	N	N	N		6.00	11-Oct-21	15-Oct-21	22-Oct-21		11-Nov-21
C19IMPACT_2743	Trinidad and Tobago	08-Oct-21	MoH	N	N	N	N	N	N	19.00		11-Oct-21	15-Oct-21	22-Oct-21		11-Nov-21
C19IMPACT_2744	Trinidad and Tobago	08-Oct-21	MoH	N	N	N	N	N	N	16.00		11-Oct-21	15-Oct-21	22-Oct-21		11-Nov-21
C19IMPACT_2745	Trinidad and Tobago	08-Oct-21	MoH	N	N	N	N	N	N	15.00		11-Oct-21	15-Oct-21	22-Oct-21		11-Nov-21
C19IMPACT_2746	Trinidad and Tobago	08-Oct-21	MoH	N	N	N	N	N	N	16.00		11-Oct-21	15-Oct-21	22-Oct-21		
C19IMPACT_2747	Trinidad and Tobago	08-Oct-21	MoH	N	N	N	N	N	N	18.00		11-Oct-21	15-Oct-21	22-Oct-21		11-Nov-21
C19IMPACT_2748	Trinidad and Tobago	08-Oct-21	MoH	N	N	N	N	N	N	16.00		11-Oct-21	15-Oct-21	22-Oct-21		11-Nov-21

C19IMPACT_2749	Trinidad and Tobago	08-Oct-21	MoH	N	N	N	N	N	N	16.00		12-Oct-21	15-Oct-21	22-Oct-21		
C19IMPACT_2750	Trinidad and Tobago	08-Oct-21	MoH	N	N	N	N	N	N		4.00	12-Oct-21	15-Oct-21	22-Oct-21		11-Nov-21
C19IMPACT_2751	Trinidad and Tobago	08-Oct-21	MoH	N	N	N	N	N	N		9.00	12-Oct-21	15-Oct-21	22-Oct-21		11-Nov-21
C19IMPACT_2752	Trinidad and Tobago	08-Oct-21	MoH	N	N	N	N	N	N		6.00	12-Oct-21	15-Oct-21	22-Oct-21		11-Nov-21
C19IMPACT_2753	Trinidad and Tobago	08-Oct-21	MoH	N	N	N	N	N	N	16.00		12-Oct-21	15-Oct-21	22-Oct-21		11-Nov-21
C19IMPACT_2754	Trinidad and Tobago	10-Oct-21	MoH	N	N	N	N	N	N	14.00		12-Oct-21	15-Oct-21	22-Oct-21		11-Nov-21
C19IMPACT_2755	Trinidad and Tobago	10-Oct-21	MoH	N	N	N	N	N	N	15.00		12-Oct-21	15-Oct-21	22-Oct-21		11-Nov-21
C19IMPACT_2756	Trinidad and Tobago	10-Oct-21	MoH	N	N	N	N	N	N	17.00		12-Oct-21	15-Oct-21	22-Oct-21		11-Nov-21
C19IMPACT_2757	Trinidad and Tobago	10-Oct-21	MoH	N	N	N	N	N	N	18.00		12-Oct-21	15-Oct-21	22-Oct-21		11-Nov-21
C19IMPACT_2758	Trinidad and Tobago	11-Oct-21	MoH	N	N	N	N	N	N	18.00		12-Oct-21	15-Oct-21	22-Oct-21		
C19IMPACT_2759	Trinidad and Tobago	11-Oct-21	MoH	N	N	N	N	N	N	17.00		12-Oct-21	15-Oct-21	22-Oct-21		
C19IMPACT_2760	Trinidad and Tobago	11-Oct-21	MoH	N	N	N	N	N	N	16.00		12-Oct-21	15-Oct-21	22-Oct-21		
C19IMPACT_2761	Trinidad and Tobago	05-Oct-21	MoH	N	N	N	N	N	Y		8.58	15-Oct-21	20-Oct-21	22-Oct-21		11-Nov-21
C19IMPACT_2762	Trinidad and Tobago	05-Oct-21	MoH	N	N	N	N	N	Y		8.15	15-Oct-21	20-Oct-21	22-Oct-21		11-Nov-21
C19IMPACT_2763	Trinidad and Tobago	05-Oct-21	MoH	N	N	N	N	N	Y		14.82	15-Oct-21	20-Oct-21	22-Oct-21		
C19IMPACT_2764	Trinidad and Tobago	05-Oct-21	MoH	N	N	N	N	N	Y		5.07	15-Oct-21	20-Oct-21	22-Oct-21		11-Nov-21
C19IMPACT_2765	Trinidad and Tobago	06-Oct-21	MoH	N	N	N	N	N	Y		9.34	15-Oct-21	20-Oct-21	22-Oct-21		11-Nov-21
C19IMPACT_2766	Trinidad and Tobago	07-Oct-21	MoH	N	N	N	N	N	Y		4.06	15-Oct-21	20-Oct-21	22-Oct-21		11-Nov-21
C19IMPACT_2767	Trinidad and Tobago	07-Oct-21	MoH	N	N	N	N	N	Y		19.04	15-Oct-21	20-Oct-21	22-Oct-21		
C19IMPACT_2768	Trinidad and Tobago	08-Oct-21	MoH	N	N	N	N	N	N	18.00		12-Oct-21	20-Oct-21	22-Oct-21		11-Nov-21
C19IMPACT_2769	Trinidad and Tobago	08-Oct-21	MoH	N	N	N	N	N	Y		13.97	15-Oct-21	20-Oct-21	22-Oct-21		11-Nov-21
C19IMPACT_2770	Trinidad and Tobago	08-Oct-21	MoH	N	N	N	N	N	Y		8.25	15-Oct-21	20-Oct-21	22-Oct-21		11-Nov-21
C19IMPACT_2771	Trinidad and Tobago	09-Oct-21	MoH	N	N	N	N	N	N	14.00		12-Oct-21	20-Oct-21	22-Oct-21		11-Nov-21
C19IMPACT_2772	Trinidad and Tobago	09-Oct-21	MoH	N	N	N	N	N	Y		9.43	15-Oct-21	20-Oct-21	22-Oct-21		11-Nov-21
C19IMPACT_2773	Trinidad and Tobago	09-Oct-21	MoH	N	N	N	N	N	Y		8.72	15-Oct-21	20-Oct-21	22-Oct-21		
C19IMPACT_2774	Trinidad and Tobago	09-Oct-21	MoH	N	N	N	N	N	Y		11.72	15-Oct-21	20-Oct-21	22-Oct-21		11-Nov-21
C19IMPACT_2775	Trinidad and Tobago	10-Oct-21	MoH	N	N	N	N	N	N	20.00		12-Oct-21	20-Oct-21	22-Oct-21		11-Nov-21
C19IMPACT_2776	Trinidad and Tobago	10-Oct-21	MoH	N	N	N	N	N	N	19.00		12-Oct-21	20-Oct-21	22-Oct-21		11-Nov-21
C19IMPACT_2777	Trinidad and Tobago	10-Oct-21	MoH	N	N	N	N	N	Y		5.98	15-Oct-21	20-Oct-21	22-Oct-21		
C19IMPACT_2778	Trinidad and Tobago	10-Oct-21	MoH	N	N	N	N	N	Y		7.68	15-Oct-21	20-Oct-21	22-Oct-21		11-Nov-21
C19IMPACT_2779	Trinidad and Tobago	11-Oct-21	MoH	N	N	N	N	N	Y		4.94	15-Oct-21	20-Oct-21	22-Oct-21		11-Nov-21
C19IMPACT_2780	Trinidad and Tobago	11-Oct-21	MoH	N	N	N	N	N	Y		7.83	15-Oct-21	20-Oct-21	22-Oct-21		11-Nov-21
C19IMPACT_2781	Trinidad and Tobago	12-Oct-21	MoH	N	N	N	N	N	N		11.00	14-Oct-21	20-Oct-21	22-Oct-21		
C19IMPACT_2782	Trinidad and Tobago	12-Oct-21	MoH	N	N	N	N	N	N	21.00		14-Oct-21	20-Oct-21	22-Oct-21		
C19IMPACT_2783	Trinidad and Tobago	12-Oct-21	MoH	N	N	N	N	N	N	21.07		14-Oct-21	20-Oct-21	22-Oct-21		11-Nov-21
C19IMPACT_2784	Trinidad and Tobago	12-Oct-21	MoH	N	N	N	N	N	N		15.50	14-Oct-21	20-Oct-21	22-Oct-21		
C19IMPACT_2785	Trinidad and Tobago	12-Oct-21	MoH	N	N	N	N	N	N	15.79		14-Oct-21	20-Oct-21	22-Oct-21		11-Nov-21
C19IMPACT_2786	Trinidad and Tobago	12-Oct-21	MoH	N	N	N	N	N	N	18.90		14-Oct-21	20-Oct-21	22-Oct-21		11-Nov-21
C19IMPACT_2787	Trinidad and Tobago	12-Oct-21	MoH	N	N	N	N	N	N	14.70		14-Oct-21	20-Oct-21	22-Oct-21		11-Nov-21
C19IMPACT_2788	Trinidad and Tobago	12-Oct-21	MoH	N	N	N	N	N	N	16.40		14-Oct-21	20-Oct-21	22-Oct-21		11-Nov-21
C19IMPACT_2789	Trinidad and Tobago	12-Oct-21	MoH	N	N	N	N	N	N	13.70		14-Oct-21	20-Oct-21	22-Oct-21		11-Nov-21
C19IMPACT_2790	Trinidad and Tobago	12-Oct-21	MoH	N	N	N	N	N	N	19.70		14-Oct-21	20-Oct-21	22-Oct-21		
C19IMPACT_2791	Trinidad and Tobago	12-Oct-21	MoH	N	N	N	N	N	N	21.40		14-Oct-21	20-Oct-21	22-Oct-21		
C19IMPACT_2792	Trinidad and Tobago	12-Oct-21	MoH	N	N	N	N	N	N	16.50		14-Oct-21	20-Oct-21	22-Oct-21		
C19IMPACT_2793	Trinidad and Tobago	12-Oct-21	MoH	N	N	N	N	N	N	15.20		14-Oct-21	20-Oct-21	22-Oct-21		
C19IMPACT_2794	Trinidad and Tobago	12-Oct-21	MoH	N	N	N	N	N	N	16.20		14-Oct-21	20-Oct-21	22-Oct-21		11-Nov-21
C19IMPACT_2795	Trinidad and Tobago	12-Oct-21	MoH	N	N	N	N	N	N	16.10		14-Oct-21	20-Oct-21	22-Oct-21		
C19IMPACT_2796	Trinidad and Tobago	12-Oct-21	MoH	N	N	N	N	N	N	12.15		14-Oct-21	20-Oct-21	22-Oct-21		11-Nov-21
C19IMPACT_2797	Trinidad and Tobago	12-Oct-21	MoH	N	N	N	N	N	Y		4.72	15-Oct-21	20-Oct-21	22-Oct-21		11-Nov-21
C19IMPACT_2798	Trinidad and Tobago	13-Oct-21	MoH	N	N	N	N	N	N	16.17		15-Oct-21	20-Oct-21	22-Oct-21		11-Nov-21
C19IMPACT_2799	Trinidad and Tobago	13-Oct-21	MoH	N	N	N	N	N	N	10.50		15-Oct-21	20-Oct-21	22-Oct-21		11-Nov-21
C19IMPACT_2800	Trinidad and Tobago	13-Oct-21	MoH	N	N	N	N	N	N	20.00		15-Oct-21	20-Oct-21	22-Oct-21		11-Nov-21
C19IMPACT_2801	Trinidad and Tobago	13-Oct-21	MoH	N	N	N	N	N	N	14.30		15-Oct-21	20-Oct-21	22-Oct-21		11-Nov-21
C19IMPACT_2802	Trinidad and Tobago	13-Oct-21	MoH	N	N	N	N	N	N	19.55		15-Oct-21	20-Oct-21	22-Oct-21		11-Nov-21
C19IMPACT_2803	Trinidad and Tobago	13-Oct-21	MoH	N	N	N	N	N	N	19.00		15-Oct-21	20-Oct-21	22-Oct-21		11-Nov-21
C19IMPACT_2804	Trinidad and Tobago	13-Oct-21	MoH	N	N	N	N	N	N	20.00		15-Oct-21	20-Oct-21	22-Oct-21		11-Nov-21
C19IMPACT_2805	Montserrat	01-Oct-21	CARPHA	N	N	N	N	N	N	14.59		19-Oct-21	23-Oct-21		27-Oct-21	11-Nov-21
C19IMPACT_2806	Montserrat	01-Oct-21	CARPHA	N	N	N	N	N	N	16.92		19-Oct-21	23-Oct-21		27-Oct-21	11-Nov-21
C19IMPACT_2807	Montserrat	01-Oct-21	CARPHA	N	N	N	N	N	N	23.19		19-Oct-21	23-Oct-21		27-Oct-21	11-Nov-21
C19IMPACT_2808	Trinidad and Tobago	03-Oct-21	MoH	N	N	N	N	N	N	19.00		19-Oct-21	23-Oct-21	29-Oct-21		11-Nov-21
C19IMPACT_2809	Montserrat	04-Oct-21	CARPHA	N	N	N	N	N	N	18.64		19-Oct-21	23-Oct-21		27-Oct-21	11-Nov-21
C19IMPACT_2810	British Virgin Islands	05-Oct-21	CARPHA	N	N	N	N	N	N	18.58		21-Oct-21	23-Oct-21		27-Oct-21	11-Nov-21
C19IMPACT_2811	Trinidad and Tobago	09-Oct-21	MoH	N	N	N	N	N	N	16.00		19-Oct-21	23-Oct-21	29-Oct-21		11-Nov-21
C19IMPACT_2812	Trinidad and Tobago	09-Oct-21	MoH	N	N	N	N	N	N	16.00		19-Oct-21	23-Oct-21	29-Oct-21		

C19IMPACT_2813	Trinidad and Tobago	11-Oct-21	CARPHA	N	N	Y	N	N	Y	14.25		19-Oct-21	23-Oct-21	29-Oct-21	27-Oct-21	11-Nov-21
C19IMPACT_2814	Trinidad and Tobago	11-Oct-21	MoH	N	N	N	N	N	N		15.00	19-Oct-21	23-Oct-21	29-Oct-21		
C19IMPACT_2815	Trinidad and Tobago	11-Oct-21	MoH	N	N	N	N	N	N		2.95	19-Oct-21	23-Oct-21	29-Oct-21		11-Nov-21
C19IMPACT_2816	Trinidad and Tobago	11-Oct-21	MoH	N	N	N	N	N	N	19.00		19-Oct-21	23-Oct-21	29-Oct-21		11-Nov-21
C19IMPACT_2817	Trinidad and Tobago	12-Oct-21	CARPHA	N	N	N	N	N	N	16.68		19-Oct-21	23-Oct-21	29-Oct-21	27-Oct-21	11-Nov-21
C19IMPACT_2818	Trinidad and Tobago	12-Oct-21	CARPHA	N	N	N	N	N	N	17.95		19-Oct-21	23-Oct-21	29-Oct-21	27-Oct-21	11-Nov-21
C19IMPACT_2819	Trinidad and Tobago	13-Oct-21	MoH	N	N	N	N	N	N	18.00		15-Oct-21	23-Oct-21	29-Oct-21		11-Nov-21
C19IMPACT_2820	Trinidad and Tobago	13-Oct-21	MoH	N	N	N	N	N	N	19.00		15-Oct-21	23-Oct-21	29-Oct-21		
C19IMPACT_2821	Trinidad and Tobago	13-Oct-21	CARPHA	N	N	Y	N	N	N	11.96		19-Oct-21	23-Oct-21	29-Oct-21	27-Oct-21	11-Nov-21
C19IMPACT_2822	Trinidad and Tobago	13-Oct-21	CARPHA	N	N	Y	N	N	N	17.16		19-Oct-21	23-Oct-21	29-Oct-21	27-Oct-21	11-Nov-21
C19IMPACT_2823	Trinidad and Tobago	13-Oct-21	CARPHA	N	N	Y	N	N	N	11.47		19-Oct-21	23-Oct-21	29-Oct-21	27-Oct-21	11-Nov-21
C19IMPACT_2824	Trinidad and Tobago	13-Oct-21	CARPHA	N	N	N	N	N	N	18.48		19-Oct-21	23-Oct-21	29-Oct-21	27-Oct-21	
C19IMPACT_2825	Trinidad and Tobago	13-Oct-21	CARPHA	N	N	Y	N	N	N	17.97		19-Oct-21	23-Oct-21	29-Oct-21	27-Oct-21	11-Nov-21
C19IMPACT_2826	Trinidad and Tobago	13-Oct-21	MoH	N	N	N	N	N	N		6.01	19-Oct-21	23-Oct-21	29-Oct-21		11-Nov-21
C19IMPACT_2827	Trinidad and Tobago	13-Oct-21	CARPHA	N	N	Y	N	N	N	15.17		21-Oct-21	23-Oct-21	29-Oct-21	27-Oct-21	11-Nov-21
C19IMPACT_2828	Trinidad and Tobago	14-Oct-21	MoH	N	N	N	N	N	N	12.20		19-Oct-21	23-Oct-21	29-Oct-21		11-Nov-21
C19IMPACT_2829	Trinidad and Tobago	14-Oct-21	MoH	N	N	N	N	N	N	18.47		19-Oct-21	23-Oct-21	29-Oct-21		
C19IMPACT_2830	Trinidad and Tobago	14-Oct-21	MoH	N	N	N	N	N	N	13.20		19-Oct-21	23-Oct-21	29-Oct-21		11-Nov-21
C19IMPACT_2831	Trinidad and Tobago	14-Oct-21	MoH	N	N	N	N	N	N	16.50		19-Oct-21	23-Oct-21	29-Oct-21		11-Nov-21
C19IMPACT_2832	Trinidad and Tobago	14-Oct-21	MoH	N	N	N	N	N	N	13.20		19-Oct-21	23-Oct-21	29-Oct-21		11-Nov-21
C19IMPACT_2833	Trinidad and Tobago	14-Oct-21	MoH	N	N	N	N	N	N	15.30		19-Oct-21	23-Oct-21	29-Oct-21		11-Nov-21
C19IMPACT_2834	Trinidad and Tobago	14-Oct-21	MoH	N	N	N	N	N	N	17.60		19-Oct-21	23-Oct-21	29-Oct-21		
C19IMPACT_2835	Trinidad and Tobago	14-Oct-21	MoH	N	N	N	N	N	N	18.11		19-Oct-21	23-Oct-21	29-Oct-21		
C19IMPACT_2836	Trinidad and Tobago	14-Oct-21	MoH	N	N	N	N	N	N	18.00		19-Oct-21	23-Oct-21	29-Oct-21		11-Nov-21
C19IMPACT_2837	Trinidad and Tobago	14-Oct-21	CARPHA	N	N	Y	N	N	N	16.67		21-Oct-21	23-Oct-21	29-Oct-21	27-Oct-21	11-Nov-21
C19IMPACT_2838	Trinidad and Tobago	14-Oct-21	CARPHA	N	N	Y	N	N	N	14.19		21-Oct-21	23-Oct-21	29-Oct-21	27-Oct-21	11-Nov-21
C19IMPACT_2839	Trinidad and Tobago	15-Oct-21	MoH	N	N	N	N	N	N		7.00	19-Oct-21	23-Oct-21	29-Oct-21		
C19IMPACT_2840	Trinidad and Tobago	15-Oct-21	MoH	N	N	N	N	N	N	14.00		19-Oct-21	23-Oct-21	29-Oct-21		11-Nov-21
C19IMPACT_2841	Trinidad and Tobago	15-Oct-21	MoH	N	N	N	N	N	N	19.00		19-Oct-21	23-Oct-21	29-Oct-21		11-Nov-21
C19IMPACT_2842	Trinidad and Tobago	15-Oct-21	MoH	N	N	N	N	N	N	19.00		19-Oct-21	23-Oct-21	29-Oct-21		11-Nov-21
C19IMPACT_2843	Trinidad and Tobago	15-Oct-21	MoH	N	N	N	N	N	N	18.00		19-Oct-21	23-Oct-21	29-Oct-21		11-Nov-21
C19IMPACT_2844	Trinidad and Tobago	15-Oct-21	MoH	N	N	N	N	N	N	18.00		19-Oct-21	23-Oct-21	29-Oct-21		11-Nov-21
C19IMPACT_2845	Trinidad and Tobago	15-Oct-21	MoH	N	N	N	N	N	N	18.00		19-Oct-21	23-Oct-21	29-Oct-21		11-Nov-21
C19IMPACT_2846	Trinidad and Tobago	15-Oct-21	MoH	N	N	N	N	N	N	19.00		19-Oct-21	23-Oct-21	29-Oct-21		11-Nov-21
C19IMPACT_2847	Trinidad and Tobago	15-Oct-21	CARPHA	N	N	Y	N	N	N	13.94		21-Oct-21	23-Oct-21	29-Oct-21	27-Oct-21	11-Nov-21
C19IMPACT_2848	Trinidad and Tobago	15-Oct-21	MoH	N	N	N	N	N	N		5.00	21-Oct-21	23-Oct-21	29-Oct-21		11-Nov-21
C19IMPACT_2849	Trinidad and Tobago	16-Oct-21	CARPHA	N	N	Y	N	N	N	14.39		21-Oct-21	23-Oct-21	29-Oct-21	27-Oct-21	11-Nov-21
C19IMPACT_2850	Trinidad and Tobago	17-Oct-21	MoH	N	N	N	N	N	N	19.00		19-Oct-21	23-Oct-21	29-Oct-21		11-Nov-21
C19IMPACT_2851	Trinidad and Tobago	17-Oct-21	MoH	N	N	N	N	N	N	19.00		21-Oct-21	23-Oct-21	29-Oct-21		11-Nov-21
C19IMPACT_2852	Trinidad and Tobago	18-Oct-21	MoH	N	N	N	N	N	N	18.00		21-Oct-21	23-Oct-21	29-Oct-21		11-Nov-21
C19IMPACT_2853	Trinidad and Tobago	18-Oct-21	CARPHA	N	N	Y	N	N	N	13.70		21-Oct-21	23-Oct-21	29-Oct-21	27-Oct-21	11-Nov-21
C19IMPACT_2854	Trinidad and Tobago	18-Oct-21	CARPHA	N	N	Y	N	N	N	14.62		21-Oct-21	23-Oct-21	29-Oct-21	27-Oct-21	11-Nov-21
C19IMPACT_2855	Trinidad and Tobago	18-Oct-21	CARPHA	N	N	Y	N	N	N	16.35		21-Oct-21	23-Oct-21	29-Oct-21	27-Oct-21	11-Nov-21
C19IMPACT_2856	Trinidad and Tobago	18-Oct-21	MoH	N	N	N	N	N	N		11.00	21-Oct-21	23-Oct-21	29-Oct-21		11-Nov-21
C19IMPACT_2857	Trinidad and Tobago	19-Oct-21	MoH	N	N	N	N	N	N		5.00	21-Oct-21	23-Oct-21	29-Oct-21		11-Nov-21
C19IMPACT_2858	Trinidad and Tobago	19-Oct-21	MoH	N	N	N	N	N	N		8.00	21-Oct-21	23-Oct-21	29-Oct-21		
C19IMPACT_2859	Trinidad and Tobago	19-Oct-21	MoH	N	N	N	N	N	N	19.65		21-Oct-21	23-Oct-21	29-Oct-21		
C19IMPACT_2860	Trinidad and Tobago	19-Oct-21	MoH	N	N	N	N	N	N		6.00	21-Oct-21	23-Oct-21	29-Oct-21		
C19IMPACT_2861	Trinidad and Tobago	19-Oct-21	MoH	N	N	N	N	N	N	14.66		21-Oct-21	23-Oct-21	29-Oct-21		11-Nov-21
C19IMPACT_2862	Trinidad and Tobago	19-Oct-21	MoH	N	N	N	N	N	N	17.57		21-Oct-21	23-Oct-21	29-Oct-21		11-Nov-21
C19IMPACT_2863	Trinidad and Tobago	19-Oct-21	MoH	N	N	N	N	N	Y	20.13		21-Oct-21	23-Oct-21	29-Oct-21		
C19IMPACT_2864	Trinidad and Tobago	19-Oct-21	MoH	N	N	N	N	N	N	18.79		21-Oct-21	23-Oct-21	29-Oct-21		
C19IMPACT_2865	Trinidad and Tobago	20-Oct-21	MoH	N	N	N	N	N	N	18.00		21-Oct-21	23-Oct-21	29-Oct-21		
C19IMPACT_2866	Trinidad and Tobago	20-Oct-21	MoH	N	N	N	N	N	N	20.00		21-Oct-21	23-Oct-21	29-Oct-21		
C19IMPACT_2867	Trinidad and Tobago	20-Oct-21	MoH	N	N	N	N	N	N	18.00		21-Oct-21	23-Oct-21	29-Oct-21		11-Nov-21
C19IMPACT_2868	Trinidad and Tobago	20-Oct-21	MoH	N	N	N	N	N	N	17.00		21-Oct-21	23-Oct-21	29-Oct-21		11-Nov-21
C19IMPACT_2869	Trinidad and Tobago	20-Oct-21	MoH	N	N	N	N	N	N	19.00		21-Oct-21	23-Oct-21	29-Oct-21		
C19IMPACT_2870	Antigua and Barbuda	18-Sept-21	CARPHA	N	N	N	N	N	N	15.99		22-Oct-21	28-Oct-21		29-Oct-21	11-Nov-21
C19IMPACT_2871	Saint Lucia	20-Sept-21	CARPHA	N	N	N	N	N	N	15.16		22-Oct-21	28-Oct-21		29-Oct-21	11-Nov-21
C19IMPACT_2872	Saint Lucia	22-Sept-21	CARPHA	N	N	N	N	N	N	13.78		22-Oct-21	28-Oct-21		29-Oct-21	11-Nov-21
C19IMPACT_2873	Saint Lucia	23-Sept-21	CARPHA	N	N	N	N	N	N	24.06		22-Oct-21	28-Oct-21		29-Oct-21	
C19IMPACT_2874	Saint Lucia	23-Sept-21	CARPHA	N	N	N	N	N	N	18.06		22-Oct-21	28-Oct-21		29-Oct-21	11-Nov-21
C19IMPACT_2875	Saint Lucia	24-Sept-21	CARPHA	N	N	N	N	N	N	17.40		22-Oct-21	28-Oct-21		29-Oct-21	11-Nov-21
C19IMPACT_2876	Saint Lucia	24-Sept-21	CARPHA	N	N	N	N	N	N	13.77		22-Oct-21	28-Oct-21		29-Oct-21	11-Nov-21

C19IMPACT_2877	Saint Lucia	25-Sept-21	CARPHA	N	N	N	N	N	N	12.82		22-Oct-21	28-Oct-21		29-Oct-21	11-Nov-21
C19IMPACT_2878	Trinidad and Tobago	01-Oct-21	MoH	N	N	N	N	N	Y		9.00	22-Oct-21	28-Oct-21	29-Oct-21		11-Nov-21
C19IMPACT_2879	Trinidad and Tobago	01-Oct-21	MoH	N	N	N	N	N	N		7.00	22-Oct-21	28-Oct-21	29-Oct-21		
C19IMPACT_2880	Saint Lucia	04-Oct-21	CARPHA	N	N	N	N	N	N	12.64		22-Oct-21	28-Oct-21		29-Oct-21	11-Nov-21
C19IMPACT_2881	Saint Lucia	05-Oct-21	CARPHA	N	N	N	N	N	N	14.28		22-Oct-21	28-Oct-21		29-Oct-21	11-Nov-21
C19IMPACT_2882	Antigua and Barbuda	06-Oct-21	CARPHA	N	N	N	N	N	N	16.92		22-Oct-21	28-Oct-21		29-Oct-21	11-Nov-21
C19IMPACT_2883	Saint Lucia	06-Oct-21	CARPHA	N	N	N	N	N	N	16.51		22-Oct-21	28-Oct-21		29-Oct-21	11-Nov-21
C19IMPACT_2884	Antigua and Barbuda	07-Oct-21	CARPHA	N	N	N	N	N	N	17.70		22-Oct-21	28-Oct-21		29-Oct-21	11-Nov-21
C19IMPACT_2885	Antigua and Barbuda	07-Oct-21	CARPHA	N	N	N	N	N	N	24.63		22-Oct-21	28-Oct-21		29-Oct-21	
C19IMPACT_2886	Antigua and Barbuda	09-Oct-21	CARPHA	N	N	N	N	N	N	13.62		22-Oct-21	28-Oct-21		29-Oct-21	11-Nov-21
C19IMPACT_2887	Antigua and Barbuda	10-Oct-21	CARPHA	N	N	N	N	N	N	16.97		22-Oct-21	28-Oct-21		29-Oct-21	11-Nov-21
C19IMPACT_2888	Antigua and Barbuda	11-Oct-21	CARPHA	N	N	N	N	N	N	17.38		22-Oct-21	28-Oct-21		29-Oct-21	11-Nov-21
C19IMPACT_2889	Antigua and Barbuda	11-Oct-21	CARPHA	N	N	N	N	N	N	14.95		22-Oct-21	28-Oct-21		29-Oct-21	11-Nov-21
C19IMPACT_2890	Antigua and Barbuda	11-Oct-21	CARPHA	N	N	N	N	N	N	21.42		22-Oct-21	28-Oct-21		29-Oct-21	11-Nov-21
C19IMPACT_2891	Antigua and Barbuda	11-Oct-21	CARPHA	N	N	N	N	N	N	21.18		22-Oct-21	28-Oct-21		29-Oct-21	
C19IMPACT_2892	Antigua and Barbuda	11-Oct-21	CARPHA	N	N	N	N	N	N	16.09		22-Oct-21	28-Oct-21		29-Oct-21	11-Nov-21
C19IMPACT_2893	Antigua and Barbuda	11-Oct-21	CARPHA	N	N	N	N	N	N	12.79		22-Oct-21	28-Oct-21		29-Oct-21	11-Nov-21
C19IMPACT_2894	Antigua and Barbuda	11-Oct-21	CARPHA	N	N	N	N	N	N	12.77		22-Oct-21	28-Oct-21		29-Oct-21	11-Nov-21
C19IMPACT_2895	Trinidad and Tobago	11-Oct-21	MoH	N	N	N	N	N	Y		3.40	22-Oct-21	28-Oct-21	29-Oct-21		11-Nov-21
C19IMPACT_2896	Antigua and Barbuda	12-Oct-21	CARPHA	N	N	N	N	N	N	13.45		22-Oct-21	28-Oct-21		29-Oct-21	11-Nov-21
C19IMPACT_2897	Trinidad and Tobago	12-Oct-21	MoH	N	N	N	N	N	Y		4.40	22-Oct-21	28-Oct-21	29-Oct-21		11-Nov-21
C19IMPACT_2898	Antigua and Barbuda	13-Oct-21	CARPHA	N	N	N	N	N	N	17.12		22-Oct-21	28-Oct-21		29-Oct-21	11-Nov-21
C19IMPACT_2899	Trinidad and Tobago	13-Oct-21	MoH	N	N	N	N	N	Y		10.35	22-Oct-21	28-Oct-21	29-Oct-21		
C19IMPACT_2900	Antigua and Barbuda	14-Oct-21	CARPHA	N	N	N	N	N	N	16.81		22-Oct-21	28-Oct-21		29-Oct-21	11-Nov-21
C19IMPACT_2901	Antigua and Barbuda	14-Oct-21	CARPHA	N	N	N	N	N	N	16.20		22-Oct-21	28-Oct-21		29-Oct-21	11-Nov-21
C19IMPACT_2902	Antigua and Barbuda	14-Oct-21	CARPHA	N	N	N	N	N	N	14.34		22-Oct-21	28-Oct-21		29-Oct-21	11-Nov-21
C19IMPACT_2903	Trinidad and Tobago	14-Oct-21	MoH	N	N	N	N	N	Y		5.18	22-Oct-21	28-Oct-21	29-Oct-21		11-Nov-21
C19IMPACT_2904	Antigua and Barbuda	15-Oct-21	CARPHA	N	N	N	N	N	N	14.47		22-Oct-21	28-Oct-21		29-Oct-21	11-Nov-21
C19IMPACT_2905	Antigua and Barbuda	15-Oct-21	CARPHA	N	N	N	N	N	N	16.76		22-Oct-21	28-Oct-21		29-Oct-21	11-Nov-21
C19IMPACT_2906	Trinidad and Tobago	17-Oct-21	MoH	N	N	N	N	N	Y		14.17	22-Oct-21	28-Oct-21	29-Oct-21		
C19IMPACT_2907	Trinidad and Tobago	18-Oct-21	CARPHA	N	N	Y	N	N	N	19.53		22-Oct-21	28-Oct-21	29-Oct-21	06-Nov-21	11-Nov-21
C19IMPACT_2908	Trinidad and Tobago	18-Oct-21	CARPHA	N	N	Y	N	N	N	17.22		22-Oct-21	28-Oct-21	29-Oct-21	29-Oct-21	11-Nov-21
C19IMPACT_2909	Trinidad and Tobago	18-Oct-21	CARPHA	N	N	Y	N	N	N	13.95		22-Oct-21	28-Oct-21	29-Oct-21	06-Nov-21	11-Nov-21
C19IMPACT_2910	Trinidad and Tobago	18-Oct-21	CARPHA	N	N	Y	N	Y	N	15.25		22-Oct-21	28-Oct-21	29-Oct-21	06-Nov-21	11-Nov-21
C19IMPACT_2911	Trinidad and Tobago	18-Oct-21	CARPHA	N	N	Y	N	Y	N	19.98		22-Oct-21	28-Oct-21	29-Oct-21	06-Nov-21	11-Nov-21
C19IMPACT_2912	Trinidad and Tobago	18-Oct-21	CARPHA	N	N	Y	N	Y	N	13.37		22-Oct-21	28-Oct-21	29-Oct-21	06-Nov-21	11-Nov-21
C19IMPACT_2913	Trinidad and Tobago	18-Oct-21	CARPHA	N	N	Y	N	Y	N	14.22		22-Oct-21	28-Oct-21	29-Oct-21	06-Nov-21	11-Nov-21
C19IMPACT_2914	Bermuda	05-Oct-21	CARPHA	N	N	N	N	Y	N	20.70		02-Nov-21	06-Nov-21		09-Nov-21	28-Nov-21
C19IMPACT_2915	Bermuda	05-Oct-21	CARPHA	N	N	N	N	N	N	21.91		02-Nov-21	06-Nov-21		09-Nov-21	28-Nov-21
C19IMPACT_2916	Bermuda	05-Oct-21	CARPHA	N	N	N	N	N	N	17.43		02-Nov-21	06-Nov-21		09-Nov-21	28-Nov-21
C19IMPACT_2917	Bermuda	05-Oct-21	CARPHA	N	N	N	N	N	N	26.53		02-Nov-21	06-Nov-21		09-Nov-21	28-Nov-21
C19IMPACT_2918	Bermuda	05-Oct-21	CARPHA	N	N	N	N	N	N	26.19		02-Nov-21	06-Nov-21		09-Nov-21	28-Nov-21
C19IMPACT_2919	Bermuda	05-Oct-21	CARPHA	N	N	N	N	N	N	22.50		02-Nov-21	06-Nov-21		09-Nov-21	28-Nov-21
C19IMPACT_2920	Bermuda	05-Oct-21	CARPHA	N	N	N	N	N	N	24.65		02-Nov-21	06-Nov-21		09-Nov-21	28-Nov-21
C19IMPACT_2921	Antigua and Barbuda	09-Oct-21	CARPHA	N	N	N	N	N	N	16.15		27-Oct-21	06-Nov-21		09-Nov-21	28-Nov-21
C19IMPACT_2922	Antigua and Barbuda	11-Oct-21	CARPHA	N	N	N	N	N	N	17.34		27-Oct-21	06-Nov-21		09-Nov-21	28-Nov-21
C19IMPACT_2923	Antigua and Barbuda	11-Oct-21	CARPHA	N	N	N	N	N	N	18.95		27-Oct-21	06-Nov-21		09-Nov-21	28-Nov-21
C19IMPACT_2924	Antigua and Barbuda	11-Oct-21	CARPHA	N	N	N	N	N	N	17.17		27-Oct-21	06-Nov-21		09-Nov-21	28-Nov-21
C19IMPACT_2925	Antigua and Barbuda	11-Oct-21	CARPHA	N	N	N	N	N	N	16.54		27-Oct-21	06-Nov-21		09-Nov-21	
C19IMPACT_2926	Antigua and Barbuda	11-Oct-21	CARPHA	N	N	N	N	N	N	19.34		27-Oct-21	06-Nov-21		09-Nov-21	28-Nov-21
C19IMPACT_2927	Antigua and Barbuda	11-Oct-21	CARPHA	N	N	N	N	N	N	18.81		27-Oct-21	06-Nov-21		09-Nov-21	
C19IMPACT_2928	Antigua and Barbuda	12-Oct-21	CARPHA	N	N	N	N	N	N	18.34		27-Oct-21	06-Nov-21		09-Nov-21	
C19IMPACT_2929	Antigua and Barbuda	12-Oct-21	CARPHA	N	N	N	N	N	N	15.36		27-Oct-21	06-Nov-21		09-Nov-21	
C19IMPACT_2930	Bermuda	12-Oct-21	CARPHA	N	N	N	N	N	N	18.42		02-Nov-21	06-Nov-21		09-Nov-21	
C19IMPACT_2931	Trinidad and Tobago	14-Oct-21	CARPHA	N	N	Y	N	N	N	18.52		27-Oct-21	06-Nov-21	13-Nov-21	09-Nov-21	28-Nov-21
C19IMPACT_2932	Trinidad and Tobago	14-Oct-21	CARPHA	N	N	Y	N	N	N	13.41		27-Oct-21	06-Nov-21	13-Nov-21	11-Nov-21	28-Nov-21
C19IMPACT_2933	Trinidad and Tobago	15-Oct-21	MoH	N	N	N	N	N	N		8.00	22-Oct-21	06-Nov-21	13-Nov-21		28-Nov-21
C19IMPACT_2934	Trinidad and Tobago	15-Oct-21	CARPHA	N	N	Y	N	N	N	15.67		27-Oct-21	06-Nov-21	13-Nov-21	09-Nov-21	28-Nov-21
C19IMPACT_2935	Trinidad and Tobago	15-Oct-21	CARPHA	N	N	Y	N	N	N	17.75		27-Oct-21	06-Nov-21	13-Nov-21	11-Nov-21	28-Nov-21
C19IMPACT_2936	Trinidad and Tobago	15-Oct-21	CARPHA	N	N	Y	N	N	N	16.86		27-Oct-21	06-Nov-21	13-Nov-21	11-Nov-21	28-Nov-21
C19IMPACT_2937	Trinidad and Tobago	15-Oct-21	CARPHA	N	N	Y	N	N	N	14.45		27-Oct-21	06-Nov-21	13-Nov-21	11-Nov-21	28-Nov-21
C19IMPACT_2938	Trinidad and Tobago	15-Oct-21	CARPHA	N	N	Y	N	N	N	15.37		27-Oct-21	06-Nov-21	13-Nov-21	11-Nov-21	28-Nov-21
C19IMPACT_2939	Trinidad and Tobago	15-Oct-21	CARPHA	N	N	N	N	N	N	13.58		27-Oct-21	06-Nov-21	13-Nov-21	11-Nov-21	
C19IMPACT_2940	Trinidad and Tobago	16-Oct-21	CARPHA	N	N	Y	N	N	N	17.70		27-Oct-21	06-Nov-21	13-Nov-21	11-Nov-21	28-Nov-21

C19IMPACT_2941	Trinidad and Tobago	16-Oct-21	CARPHA	N	N	Y	N	N	N	13.10		27-Oct-21	06-Nov-21	13-Nov-21	11-Nov-21	28-Nov-21
C19IMPACT_2942	Trinidad and Tobago	17-Oct-21	MoH	N	N	N	N	N	Y		4.60	22-Oct-21	06-Nov-21	13-Nov-21	11-Nov-21	28-Nov-21
C19IMPACT_2943	Trinidad and Tobago	17-Oct-21	CARPHA	N	N	N	N	N	N	19.74		27-Oct-21	06-Nov-21	13-Nov-21	09-Nov-21	
C19IMPACT_2944	Trinidad and Tobago	17-Oct-21	CARPHA	N	N	Y	N	N	N	17.56		27-Oct-21	06-Nov-21	13-Nov-21	11-Nov-21	28-Nov-21
C19IMPACT_2945	Trinidad and Tobago	17-Oct-21	CARPHA	N	N	N	N	Y	N	19.67		27-Oct-21	06-Nov-21	13-Nov-21	11-Nov-21	
C19IMPACT_2946	Trinidad and Tobago	18-Oct-21	MoH	N	N	N	N	N	Y		7.88	22-Oct-21	06-Nov-21	13-Nov-21	11-Nov-21	28-Nov-21
C19IMPACT_2947	Saint Vincent and the Grenadines	18-Oct-21	CARPHA	N	N	N	N	N	N	21.52		25-Oct-21	06-Nov-21		09-Nov-21	28-Nov-21
C19IMPACT_2948	Saint Vincent and the Grenadines	18-Oct-21	CARPHA	N	N	N	N	N	N	17.76		25-Oct-21	06-Nov-21		09-Nov-21	28-Nov-21
C19IMPACT_2949	Saint Vincent and the Grenadines	18-Oct-21	CARPHA	N	N	N	N	N	N	16.60		25-Oct-21	06-Nov-21		09-Nov-21	28-Nov-21
C19IMPACT_2950	Saint Vincent and the Grenadines	18-Oct-21	CARPHA	N	N	N	N	N	N	20.77		25-Oct-21	06-Nov-21		09-Nov-21	28-Nov-21
C19IMPACT_2951	Trinidad and Tobago	19-Oct-21	MoH	N	N	N	N	N	Y		3.00	22-Oct-21	06-Nov-21	13-Nov-21		28-Nov-21
C19IMPACT_2952	Saint Vincent and the Grenadines	19-Oct-21	CARPHA	N	N	N	N	N	N	20.92		25-Oct-21	06-Nov-21		09-Nov-21	28-Nov-21
C19IMPACT_2953	Saint Vincent and the Grenadines	19-Oct-21	CARPHA	N	N	N	N	N	N	20.17		25-Oct-21	06-Nov-21		09-Nov-21	28-Nov-21
C19IMPACT_2954	Saint Vincent and the Grenadines	19-Oct-21	CARPHA	N	N	N	N	N	N	21.75		25-Oct-21	06-Nov-21		09-Nov-21	28-Nov-21
C19IMPACT_2955	Saint Vincent and the Grenadines	19-Oct-21	CARPHA	N	N	N	N	N	N	17.71		25-Oct-21	06-Nov-21		09-Nov-21	28-Nov-21
C19IMPACT_2956	Saint Vincent and the Grenadines	19-Oct-21	CARPHA	N	N	N	N	N	N	19.56		25-Oct-21	06-Nov-21		09-Nov-21	28-Nov-21
C19IMPACT_2957	Trinidad and Tobago	19-Oct-21	MoH	N	N	N	N	N	N		13.00	25-Oct-21	06-Nov-21	13-Nov-21		28-Nov-21
C19IMPACT_2958	Trinidad and Tobago	19-Oct-21	MoH	N	N	N	N	N	N		7.00	25-Oct-21	06-Nov-21	13-Nov-21		28-Nov-21
C19IMPACT_2959	Trinidad and Tobago	20-Oct-21	MoH	N	N	N	N	N	N	16.96		22-Oct-21	06-Nov-21	13-Nov-21		28-Nov-21
C19IMPACT_2960	Trinidad and Tobago	20-Oct-21	MoH	N	N	N	N	N	N	17.86		22-Oct-21	06-Nov-21	13-Nov-21		28-Nov-21
C19IMPACT_2961	Trinidad and Tobago	20-Oct-21	MoH	N	N	N	N	N	N	16.96		22-Oct-21	06-Nov-21	13-Nov-21		28-Nov-21
C19IMPACT_2962	Trinidad and Tobago	20-Oct-21	MoH	N	N	N	N	N	N	23.73		22-Oct-21	06-Nov-21	13-Nov-21		
C19IMPACT_2963	Trinidad and Tobago	20-Oct-21	MoH	N	N	N	N	N	N	18.09		22-Oct-21	06-Nov-21	13-Nov-21		
C19IMPACT_2964	Trinidad and Tobago	20-Oct-21	MoH	N	N	N	N	N	N	15.00		22-Oct-21	06-Nov-21	13-Nov-21		28-Nov-21
C19IMPACT_2965	Trinidad and Tobago	20-Oct-21	MoH	N	N	N	N	N	N	15.00		22-Oct-21	06-Nov-21	13-Nov-21		28-Nov-21
C19IMPACT_2966	Trinidad and Tobago	20-Oct-21	MoH	N	N	N	N	N	N	18.00		25-Oct-21	06-Nov-21	13-Nov-21		
C19IMPACT_2967	Trinidad and Tobago	20-Oct-21	MoH	N	N	N	N	N	N	19.00		25-Oct-21	06-Nov-21	13-Nov-21		28-Nov-21
C19IMPACT_2968	Trinidad and Tobago	20-Oct-21	MoH	N	N	N	N	N	N	19.00		25-Oct-21	06-Nov-21	13-Nov-21		28-Nov-21
C19IMPACT_2969	Trinidad and Tobago	20-Oct-21	MoH	N	N	N	N	N	N	19.00		25-Oct-21	06-Nov-21	13-Nov-21		
C19IMPACT_2970	Trinidad and Tobago	20-Oct-21	MoH	N	N	N	N	N	N	18.00		25-Oct-21	06-Nov-21	13-Nov-21		28-Nov-21
C19IMPACT_2971	Trinidad and Tobago	20-Oct-21	MoH	N	N	N	N	N	N		9.00	25-Oct-21	06-Nov-21	13-Nov-21		
C19IMPACT_2972	Trinidad and Tobago	20-Oct-21	MoH	N	N	N	N	N	N	18.00		25-Oct-21	06-Nov-21	13-Nov-21		28-Nov-21
C19IMPACT_2973	Trinidad and Tobago	20-Oct-21	MoH	N	N	N	N	N	N		6.00	25-Oct-21	06-Nov-21	13-Nov-21		28-Nov-21
C19IMPACT_2974	Trinidad and Tobago	21-Oct-21	MoH	N	N	N	N	N	N	18.00		25-Oct-21	06-Nov-21	13-Nov-21		
C19IMPACT_2975	Trinidad and Tobago	21-Oct-21	MoH	N	N	N	N	N	N	19.10		22-Oct-21	06-Nov-21	13-Nov-21		28-Nov-21
C19IMPACT_2976	Trinidad and Tobago	21-Oct-21	MoH	N	N	N	N	N	N	16.00		22-Oct-21	06-Nov-21	13-Nov-21		28-Nov-21
C19IMPACT_2977	Trinidad and Tobago	21-Oct-21	MoH	N	N	N	N	N	N	20.00		25-Oct-21	06-Nov-21	13-Nov-21		
C19IMPACT_2978	Trinidad and Tobago	21-Oct-21	MoH	N	N	N	N	N	N		9.00	25-Oct-21	06-Nov-21	13-Nov-21		
C19IMPACT_2979	Trinidad and Tobago	22-Oct-21	MoH	N	N	N	N	N	N	19.00		25-Oct-21	06-Nov-21	13-Nov-21		28-Nov-21
C19IMPACT_2980	Trinidad and Tobago	22-Oct-21	MoH	N	N	N	N	N	N	16.00		25-Oct-21	06-Nov-21	13-Nov-21		28-Nov-21
C19IMPACT_2981	Trinidad and Tobago	22-Oct-21	MoH	N	N	N	N	N	N	17.79		25-Oct-21	06-Nov-21	13-Nov-21		28-Nov-21
C19IMPACT_2982	Trinidad and Tobago	22-Oct-21	MoH	N	N	N	N	N	N	12.90		27-Oct-21	06-Nov-21	13-Nov-21		28-Nov-21
C19IMPACT_2983	Trinidad and Tobago	22-Oct-21	MoH	N	N	N	N	N	N	11.60		27-Oct-21	06-Nov-21	13-Nov-21		28-Nov-21
C19IMPACT_2984	Trinidad and Tobago	22-Oct-21	MoH	N	N	N	N	N	N	19.50		27-Oct-21	06-Nov-21	13-Nov-21		28-Nov-21
C19IMPACT_2985	Trinidad and Tobago	22-Oct-21	MoH	N	N	N	N	N	N	11.70		27-Oct-21	06-Nov-21	13-Nov-21		28-Nov-21
C19IMPACT_2986	Trinidad and Tobago	22-Oct-21	MoH	N	N	N	N	N	N	14.60		27-Oct-21	06-Nov-21	13-Nov-21		28-Nov-21
C19IMPACT_2987	Trinidad and Tobago	22-Oct-21	MoH	N	N	N	N	N	N		4.00	27-Oct-21	06-Nov-21	13-Nov-21		28-Nov-21
C19IMPACT_2988	Trinidad and Tobago	22-Oct-21	MoH	N	N	N	N	N	N		14.00	27-Oct-21	06-Nov-21	13-Nov-21		28-Nov-21
C19IMPACT_2989	Trinidad and Tobago	23-Oct-21	MoH	N	N	N	N	N	N	16.00		25-Oct-21	06-Nov-21	13-Nov-21		28-Nov-21
C19IMPACT_2990	Trinidad and Tobago	23-Oct-21	MoH	N	N	N	N	N	N	16.00		27-Oct-21	06-Nov-21	13-Nov-21		28-Nov-21
C19IMPACT_2991	Trinidad and Tobago	23-Oct-21	MoH	N	N	N	N	N	N	17.00		27-Oct-21	06-Nov-21	13-Nov-21		28-Nov-21
C19IMPACT_2992	Trinidad and Tobago	23-Oct-21	MoH	N	N	N	N	N	N	17.00		27-Oct-21	06-Nov-21	13-Nov-21		28-Nov-21
C19IMPACT_2993	Trinidad and Tobago	23-Oct-21	CARPHA	N	N	Y	N	N	N	12.70		02-Nov-21	06-Nov-21	13-Nov-21	11-Nov-21	28-Nov-21
C19IMPACT_2994	Trinidad and Tobago	24-Oct-21	MoH	N	N	N	N	N	N		12.00	27-Oct-21	06-Nov-21	13-Nov-21		
C19IMPACT_2995	Trinidad and Tobago	25-Oct-21	MoH	N	N	N	N	N	N	13.00		27-Oct-21	06-Nov-21	13-Nov-21		28-Nov-21
C19IMPACT_2996	Trinidad and Tobago	25-Oct-21	CARPHA	N	N	Y	N	N	N	12.12		02-Nov-21	06-Nov-21	13-Nov-21	11-Nov-21	28-Nov-21
C19IMPACT_2997	Trinidad and Tobago	25-Oct-21	CARPHA	N	N	Y	N	Y	N	10.00		02-Nov-21	06-Nov-21	13-Nov-21	11-Nov-21	28-Nov-21
C19IMPACT_2998	Trinidad and Tobago	25-Oct-21	CARPHA	N	N	Y	N	Y	N	11.56		02-Nov-21	06-Nov-21	13-Nov-21	11-Nov-21	28-Nov-21
C19IMPACT_2999	Trinidad and Tobago	25-Oct-21	CARPHA	N	N	Y	N	Y	N	21.86		02-Nov-21	06-Nov-21	13-Nov-21	11-Nov-21	28-Nov-21
C19IMPACT_3000	Trinidad and Tobago	26-Oct-21	CARPHA	N	N	Y	N	Y	N	14.53		02-Nov-21	06-Nov-21	13-Nov-21	11-Nov-21	28-Nov-21
C19IMPACT_3001	Trinidad and Tobago	27-Oct-21	CARPHA	N	N	N	N	Y	N	23.83		02-Nov-21	06-Nov-21	13-Nov-21	11-Nov-21	28-Nov-21
C19IMPACT_3002	Trinidad and Tobago	02-Oct-21	MoH	N	N	N	N	N	Y		4.00	22-Oct-21	12-Nov-21	29-Oct-21		28-Nov-21
C19IMPACT_3003	Trinidad and Tobago	05-Oct-21	MoH	N	N	N	N	N	Y		4.00	22-Oct-21	12-Nov-21	29-Oct-21		28-Nov-21
C19IMPACT_3004	Trinidad and Tobago	06-Oct-21	MoH	N	N	N	N	N	Y		4.00	22-Oct-21	12-Nov-21	29-Oct-21		28-Nov-21

C19IMPACT_3005	Trinidad and Tobago	06-Oct-21	MoH	N	N	N	N	N	Y		6.00	22-Oct-21	12-Nov-21	29-Oct-21		28-Nov-21
C19IMPACT_3006	Trinidad and Tobago	07-Oct-21	MoH	N	N	N	N	N	Y		7.00	22-Oct-21	12-Nov-21	29-Oct-21		28-Nov-21
C19IMPACT_3007	Trinidad and Tobago	07-Oct-21	MoH	N	N	N	N	N	Y		5.00	22-Oct-21	12-Nov-21	29-Oct-21		28-Nov-21
C19IMPACT_3008	Trinidad and Tobago	11-Oct-21	MoH	N	N	N	N	N	Y		8.00	22-Oct-21	12-Nov-21	29-Oct-21		28-Nov-21
C19IMPACT_3009	Trinidad and Tobago	13-Oct-21	MoH	N	N	N	N	N	Y		6.00	22-Oct-21	12-Nov-21	29-Oct-21		28-Nov-21
C19IMPACT_3010	Trinidad and Tobago	13-Oct-21	MoH	N	N	N	N	N	Y		4.00	22-Oct-21	12-Nov-21	29-Oct-21		28-Nov-21
C19IMPACT_3011	Trinidad and Tobago	13-Oct-21	MoH	N	N	N	N	N	Y		5.00	22-Oct-21	12-Nov-21	29-Oct-21		28-Nov-21
C19IMPACT_3012	Trinidad and Tobago	17-Oct-21	MoH	N	N	N	N	N	Y		4.00	22-Oct-21	12-Nov-21	29-Oct-21		28-Nov-21
C19IMPACT_3013	Saint Vincent and the Grenadines	18-Oct-21	CARPHA	N	N	N	N	N	Y	18.95		25-Oct-21	12-Nov-21		29-Oct-21	28-Nov-21
C19IMPACT_3014	Saint Vincent and the Grenadines	18-Oct-21	CARPHA	N	N	N	N	N	Y	16.18		25-Oct-21	12-Nov-21		29-Oct-21	28-Nov-21
C19IMPACT_3015	Saint Vincent and the Grenadines	18-Oct-21	CARPHA	N	N	N	N	N	Y	19.39		25-Oct-21	12-Nov-21		29-Oct-21	28-Nov-21
C19IMPACT_3016	Saint Vincent and the Grenadines	18-Oct-21	CARPHA	N	N	N	N	N	Y	15.95		25-Oct-21	12-Nov-21		29-Oct-21	28-Nov-21
C19IMPACT_3017	Saint Kitts and Nevis	18-Oct-21	CARPHA	N	N	N	N	N	N	17.39		09-Nov-21	12-Nov-21		16-Nov-21	28-Nov-21
C19IMPACT_3018	Trinidad and Tobago	19-Oct-21	CARPHA	N	N	Y	N	N	Y	14.58		25-Oct-21	12-Nov-21	29-Oct-21	06-Nov-21	28-Nov-21
C19IMPACT_3019	Trinidad and Tobago	19-Oct-21	CARPHA	N	N	Y	N	Y	Y	21.15		25-Oct-21	12-Nov-21	29-Oct-21	29-Oct-21	28-Nov-21
C19IMPACT_3020	Trinidad and Tobago	19-Oct-21	CARPHA	N	N	Y	N	N	Y	20.38		25-Oct-21	12-Nov-21	29-Oct-21	06-Nov-21	28-Nov-21
C19IMPACT_3021	Saint Kitts and Nevis	19-Oct-21	CARPHA	N	N	N	N	N	N	25.48		09-Nov-21	12-Nov-21		16-Nov-21	
C19IMPACT_3022	Trinidad and Tobago	20-Oct-21	CARPHA	N	N	Y	N	N	Y	17.32		25-Oct-21	12-Nov-21	29-Oct-21	06-Nov-21	28-Nov-21
C19IMPACT_3023	Trinidad and Tobago	20-Oct-21	CARPHA	N	N	N	N	N	Y	25.63		25-Oct-21	12-Nov-21	29-Oct-21	29-Oct-21	
C19IMPACT_3024	Trinidad and Tobago	20-Oct-21	CARPHA	N	N	Y	N	N	Y	15.31		25-Oct-21	12-Nov-21	29-Oct-21	06-Nov-21	28-Nov-21
C19IMPACT_3025	Trinidad and Tobago	21-Oct-21	CARPHA	N	N	Y	N	N	Y	14.28		25-Oct-21	12-Nov-21	29-Oct-21	06-Nov-21	28-Nov-21
C19IMPACT_3026	Saint Kitts and Nevis	22-Oct-21	CARPHA	N	N	N	N	Y	N	20.30		09-Nov-21	12-Nov-21		16-Nov-21	28-Nov-21
C19IMPACT_3027	Saint Kitts and Nevis	23-Oct-21	CARPHA	N	N	N	N	N	N	12.30		09-Nov-21	12-Nov-21		16-Nov-21	28-Nov-21
C19IMPACT_3028	Saint Kitts and Nevis	24-Oct-21	CARPHA	N	N	N	N	N	N	14.69		09-Nov-21	12-Nov-21		16-Nov-21	28-Nov-21
C19IMPACT_3029	Saint Kitts and Nevis	25-Oct-21	CARPHA	N	N	N	N	N	N	21.88		09-Nov-21	12-Nov-21		16-Nov-21	28-Nov-21
C19IMPACT_3030	Saint Kitts and Nevis	25-Oct-21	CARPHA	N	N	N	N	N	N	16.47		09-Nov-21	12-Nov-21		16-Nov-21	28-Nov-21
C19IMPACT_3031	Saint Kitts and Nevis	25-Oct-21	CARPHA	N	N	N	N	N	N	15.91		09-Nov-21	12-Nov-21		16-Nov-21	28-Nov-21
C19IMPACT_3032	Trinidad and Tobago	26-Oct-21	CARPHA	N	N	Y	N	N	N	22.11		02-Nov-21	12-Nov-21	19-Nov-21	20-Nov-21	28-Nov-21
C19IMPACT_3033	Trinidad and Tobago	26-Oct-21	CARPHA	N	N	Y	N	Y	N	19.60		02-Nov-21	12-Nov-21	19-Nov-21	20-Nov-21	28-Nov-21
C19IMPACT_3034	Trinidad and Tobago	26-Oct-21	CARPHA	N	N	Y	N	Y	N	10.36		02-Nov-21	12-Nov-21	19-Nov-21	20-Nov-21	28-Nov-21
C19IMPACT_3035	Saint Kitts and Nevis	26-Oct-21	CARPHA	N	N	N	N	Y	N	19.11		09-Nov-21	12-Nov-21		16-Nov-21	28-Nov-21
C19IMPACT_3036	Saint Kitts and Nevis	26-Oct-21	CARPHA	N	N	N	N	N	N	19.34		09-Nov-21	12-Nov-21		16-Nov-21	28-Nov-21
C19IMPACT_3037	Saint Kitts and Nevis	27-Oct-21	CARPHA	N	N	N	N	N	N	23.75		09-Nov-21	12-Nov-21		16-Nov-21	
C19IMPACT_3038	Saint Kitts and Nevis	27-Oct-21	CARPHA	N	N	N	N	N	Y	21.10		09-Nov-21	12-Nov-21		16-Nov-21	28-Nov-21
C19IMPACT_3039	Trinidad and Tobago	30-Oct-21	MoH	N	N	N	N	N	N	16.00		09-Nov-21	12-Nov-21	19-Nov-21		28-Nov-21
C19IMPACT_3040	Trinidad and Tobago	30-Oct-21	MoH	N	N	N	N	N	N		20.00	09-Nov-21	12-Nov-21	19-Nov-21		
C19IMPACT_3041	Trinidad and Tobago	31-Oct-21	MoH	N	N	N	N	N	N	17.00		09-Nov-21	12-Nov-21	19-Nov-21		28-Nov-21
C19IMPACT_3042	Trinidad and Tobago	31-Oct-21	MoH	N	N	N	N	N	N		17.00	09-Nov-21	12-Nov-21	19-Nov-21		
C19IMPACT_3043	Trinidad and Tobago	01-Nov-21	MoH	N	N	N	N	N	N		12.00	09-Nov-21	12-Nov-21	19-Nov-21		
C19IMPACT_3044	Saint Kitts and Nevis	01-Nov-21	CARPHA	N	N	N	N	N	N	19.26		09-Nov-21	12-Nov-21		16-Nov-21	28-Nov-21
C19IMPACT_3045	Saint Kitts and Nevis	01-Nov-21	CARPHA	N	N	N	N	N	N	13.99		09-Nov-21	12-Nov-21		16-Nov-21	28-Nov-21
C19IMPACT_3046	Saint Kitts and Nevis	01-Nov-21	CARPHA	N	N	N	N	N	N	24.17		09-Nov-21	12-Nov-21		16-Nov-21	
C19IMPACT_3047	Saint Kitts and Nevis	01-Nov-21	CARPHA	N	N	N	N	N	N	17.57		09-Nov-21	12-Nov-21		16-Nov-21	28-Nov-21
C19IMPACT_3048	Trinidad and Tobago	01-Nov-21	MoH	N	N	N	N	N	N			09-Nov-21	12-Nov-21	19-Nov-21		28-Nov-21
C19IMPACT_3049	Trinidad and Tobago	01-Nov-21	MoH	N	N	N	N	N	N	15.10		09-Nov-21	12-Nov-21	19-Nov-21		
C19IMPACT_3050	Trinidad and Tobago	01-Nov-21	MoH	N	N	N	N	N	N	17.00		09-Nov-21	12-Nov-21	19-Nov-21		
C19IMPACT_3051	Trinidad and Tobago	01-Nov-21	MoH	N	N	N	N	N	N	18.00		09-Nov-21	12-Nov-21	19-Nov-21		28-Nov-21
C19IMPACT_3052	Trinidad and Tobago	01-Nov-21	MoH	N	N	N	N	N	N	17.00		09-Nov-21	12-Nov-21	19-Nov-21		28-Nov-21
C19IMPACT_3053	Trinidad and Tobago	01-Nov-21	MoH	N	N	N	N	N	N	17.00		09-Nov-21	12-Nov-21	19-Nov-21		28-Nov-21
C19IMPACT_3054	Trinidad and Tobago	01-Nov-21	MoH	N	N	N	N	N	N	14.40		09-Nov-21	12-Nov-21	19-Nov-21		28-Nov-21
C19IMPACT_3055	Trinidad and Tobago	01-Nov-21	MoH	N	N	N	N	N	N	10.00		09-Nov-21	12-Nov-21	19-Nov-21		28-Nov-21
C19IMPACT_3056	Trinidad and Tobago	01-Nov-21	MoH	N	N	N	N	N	N	13.00		09-Nov-21	12-Nov-21	19-Nov-21		28-Nov-21
C19IMPACT_3057	Saint Kitts and Nevis	02-Nov-21	CARPHA	N	N	N	N	N	N	15.58		09-Nov-21	12-Nov-21		16-Nov-21	28-Nov-21
C19IMPACT_3058	Saint Kitts and Nevis	02-Nov-21	CARPHA	N	N	N	N	N	N	20.70		09-Nov-21	12-Nov-21		16-Nov-21	28-Nov-21
C19IMPACT_3059	Saint Kitts and Nevis	02-Nov-21	CARPHA	N	N	N	N	N	N	24.48		09-Nov-21	12-Nov-21		16-Nov-21	28-Nov-21
C19IMPACT_3060	Trinidad and Tobago	02-Nov-21	MoH	N	N	N	N	N	N	14.00		09-Nov-21	12-Nov-21	19-Nov-21		28-Nov-21
C19IMPACT_3061	Trinidad and Tobago	02-Nov-21	MoH	N	N	N	N	N	N	17.00		09-Nov-21	12-Nov-21	19-Nov-21		28-Nov-21
C19IMPACT_3062	Trinidad and Tobago	02-Nov-21	MoH	N	N	N	N	N	N		24.00	09-Nov-21	12-Nov-21	19-Nov-21		
C19IMPACT_3063	Trinidad and Tobago	02-Nov-21	MoH	N	N	N	N	N	N		14.00	09-Nov-21	12-Nov-21	19-Nov-21		
C19IMPACT_3064	Trinidad and Tobago	03-Nov-21	MoH	N	N	N	N	N	N		8.00	09-Nov-21	12-Nov-21	19-Nov-21		28-Nov-21
C19IMPACT_3065	Trinidad and Tobago	03-Nov-21	MoH	N	N	N	N	N	N		3.00	09-Nov-21	12-Nov-21	19-Nov-21		28-Nov-21
C19IMPACT_3066	Trinidad and Tobago	03-Nov-21	MoH	N	N	N	N	N	N		5.00	09-Nov-21	12-Nov-21	19-Nov-21		28-Nov-21
C19IMPACT_3067	Trinidad and Tobago	03-Nov-21	MoH	N	N	N	N	N	N		3.00	09-Nov-21	12-Nov-21	19-Nov-21		28-Nov-21
C19IMPACT_3068	Trinidad and Tobago	20-Oct-21	MoH	N	N	N	N	N	Y		10.43	09-Nov-21	18-Nov-21	26-Nov-21		28-Nov-21

C19IMPACT_3069	Trinidad and Tobago	20-Oct-21	MoH	N	N	N	N	N	Y		9.34	09-Nov-21	18-Nov-21	26-Nov-21		28-Nov-21
C19IMPACT_3070	Trinidad and Tobago	21-Oct-21	MoH	N	N	N	N	N	N	17.00		09-Nov-21	18-Nov-21	26-Nov-21		28-Nov-21
C19IMPACT_3071	Trinidad and Tobago	21-Oct-21	MoH	N	N	N	N	N	Y		6.23	09-Nov-21	18-Nov-21	26-Nov-21		28-Nov-21
C19IMPACT_3072	Trinidad and Tobago	21-Oct-21	MoH	N	N	N	N	N	Y		11.59	09-Nov-21	18-Nov-21	26-Nov-21		28-Nov-21
C19IMPACT_3073	Trinidad and Tobago	22-Oct-21	MoH	N	N	N	N	N	Y		4.08	09-Nov-21	18-Nov-21	26-Nov-21		28-Nov-21
C19IMPACT_3074	Trinidad and Tobago	23-Oct-21	MoH	N	N	N	N	N	Y		3.59	09-Nov-21	18-Nov-21	26-Nov-21		28-Nov-21
C19IMPACT_3075	Trinidad and Tobago	23-Oct-21	MoH	N	N	N	N	N	Y		8.47	09-Nov-21	18-Nov-21	26-Nov-21		28-Nov-21
C19IMPACT_3076	Trinidad and Tobago	24-Oct-21	MoH	N	N	N	N	N	N			09-Nov-21	18-Nov-21	26-Nov-21		28-Nov-21
C19IMPACT_3077	Trinidad and Tobago	24-Oct-21	MoH	N	N	N	N	N	Y		3.71	09-Nov-21	18-Nov-21	26-Nov-21		28-Nov-21
C19IMPACT_3078	Trinidad and Tobago	24-Oct-21	MoH	N	N	N	N	N	Y		16.85	09-Nov-21	18-Nov-21	26-Nov-21		
C19IMPACT_3079	Trinidad and Tobago	25-Oct-21	MoH	N	N	N	N	N	N		31.00	09-Nov-21	18-Nov-21	26-Nov-21		
C19IMPACT_3080	Trinidad and Tobago	25-Oct-21	MoH	N	N	N	N	N	N		30.00	09-Nov-21	18-Nov-21	26-Nov-21		
C19IMPACT_3081	Trinidad and Tobago	25-Oct-21	MoH	N	N	N	N	N	Y		13.53	09-Nov-21	18-Nov-21	26-Nov-21		
C19IMPACT_3082	Trinidad and Tobago	25-Oct-21	MoH	N	N	N	N	N	N	14.70		09-Nov-21	18-Nov-21	26-Nov-21		
C19IMPACT_3083	Trinidad and Tobago	25-Oct-21	MoH	N	N	N	N	N	N	15.90		09-Nov-21	18-Nov-21	26-Nov-21		
C19IMPACT_3084	Trinidad and Tobago	25-Oct-21	MoH	N	N	N	N	N	N	19.56		09-Nov-21	18-Nov-21	26-Nov-21		28-Nov-21
C19IMPACT_3085	Trinidad and Tobago	25-Oct-21	MoH	N	N	N	N	N	N	11.30		09-Nov-21	18-Nov-21	26-Nov-21		
C19IMPACT_3086	Trinidad and Tobago	25-Oct-21	MoH	N	N	N	N	N	N	16.30		09-Nov-21	18-Nov-21	26-Nov-21		
C19IMPACT_3087	Trinidad and Tobago	25-Oct-21	MoH	N	N	N	N	N	N	12.70		09-Nov-21	18-Nov-21	26-Nov-21		28-Nov-21
C19IMPACT_3088	Trinidad and Tobago	26-Oct-21	MoH	N	N	N	N	N	N		18.00	09-Nov-21	18-Nov-21	26-Nov-21		
C19IMPACT_3089	Trinidad and Tobago	26-Oct-21	MoH	N	N	N	N	N	N	18.72		09-Nov-21	18-Nov-21	26-Nov-21		28-Nov-21
C19IMPACT_3090	Trinidad and Tobago	26-Oct-21	MoH	N	N	N	N	N	N	18.87		09-Nov-21	18-Nov-21	26-Nov-21		28-Nov-21
C19IMPACT_3091	Trinidad and Tobago	26-Oct-21	MoH	N	N	N	N	N	N	18.87		09-Nov-21	18-Nov-21	26-Nov-21		28-Nov-21
C19IMPACT_3092	Trinidad and Tobago	26-Oct-21	MoH	N	N	N	N	N	N	13.50		09-Nov-21	18-Nov-21	26-Nov-21		28-Nov-21
C19IMPACT_3093	Trinidad and Tobago	26-Oct-21	MoH	N	N	N	N	N	N	13.90		09-Nov-21	18-Nov-21	26-Nov-21		28-Nov-21
C19IMPACT_3094	Trinidad and Tobago	26-Oct-21	MoH	N	N	N	N	N	N	19.89		09-Nov-21	18-Nov-21	26-Nov-21		28-Nov-21
C19IMPACT_3095	Trinidad and Tobago	26-Oct-21	MoH	N	N	N	N	N	N	15.62		09-Nov-21	18-Nov-21	26-Nov-21		28-Nov-21
C19IMPACT_3096	Trinidad and Tobago	26-Oct-21	MoH	N	N	N	N	N	N	14.90		09-Nov-21	18-Nov-21	26-Nov-21		28-Nov-21
C19IMPACT_3097	Trinidad and Tobago	26-Oct-21	MoH	N	N	N	N	N	N	14.03		09-Nov-21	18-Nov-21	26-Nov-21		28-Nov-21
C19IMPACT_3098	Trinidad and Tobago	26-Oct-21	MoH	N	N	N	N	N	N		8.00	09-Nov-21	18-Nov-21	26-Nov-21		28-Nov-21
C19IMPACT_3099	Trinidad and Tobago	26-Oct-21	MoH	N	N	N	N	N	N		9.00	09-Nov-21	18-Nov-21	26-Nov-21		
C19IMPACT_3100	Trinidad and Tobago	26-Oct-21	MoH	N	N	N	N	N	N		13.00	09-Nov-21	18-Nov-21	26-Nov-21		
C19IMPACT_3101	Trinidad and Tobago	27-Oct-21	MoH	N	N	N	N	N	N			09-Nov-21	18-Nov-21	26-Nov-21		28-Nov-21
C19IMPACT_3102	Trinidad and Tobago	27-Oct-21	MoH	N	N	N	N	N	N		9.00	09-Nov-21	18-Nov-21	26-Nov-21		
C19IMPACT_3103	Trinidad and Tobago	27-Oct-21	MoH	N	N	N	N	N	N		17.00	09-Nov-21	18-Nov-21	26-Nov-21		
C19IMPACT_3104	Trinidad and Tobago	27-Oct-21	MoH	N	N	N	N	N	N		19.00	09-Nov-21	18-Nov-21	26-Nov-21		
C19IMPACT_3105	Trinidad and Tobago	28-Oct-21	CARPHA	N	N	N	N	N	Y	20.51		09-Nov-21	18-Nov-21	26-Nov-21	26-Nov-21	28-Nov-21
C19IMPACT_3106	Trinidad and Tobago	28-Oct-21	CARPHA	N	N	N	N	N	Y	22.02		09-Nov-21	18-Nov-21	26-Nov-21	26-Nov-21	28-Nov-21
C19IMPACT_3107	Trinidad and Tobago	28-Oct-21	MoH	N	N	N	N	N	N	14.60		09-Nov-21	18-Nov-21	26-Nov-21		28-Nov-21
C19IMPACT_3108	Trinidad and Tobago	29-Oct-21	MoH	N	N	N	N	N	N	15.00		09-Nov-21	18-Nov-21	26-Nov-21		28-Nov-21
C19IMPACT_3109	Trinidad and Tobago	29-Oct-21	MoH	N	N	N	N	N	N	17.00		09-Nov-21	18-Nov-21	26-Nov-21		28-Nov-21
C19IMPACT_3110	Trinidad and Tobago	29-Oct-21	MoH	N	N	N	N	N	N	16.00		09-Nov-21	18-Nov-21	26-Nov-21		28-Nov-21
C19IMPACT_3111	Trinidad and Tobago	29-Oct-21	MoH	N	N	N	N	N	N	19.00		09-Nov-21	18-Nov-21	26-Nov-21		28-Nov-21
C19IMPACT_3112	Trinidad and Tobago	29-Oct-21	MoH	N	N	N	N	N	N	15.10		09-Nov-21	18-Nov-21	26-Nov-21		28-Nov-21
C19IMPACT_3113	Trinidad and Tobago	29-Oct-21	MoH	N	N	N	N	N	N	13.50		09-Nov-21	18-Nov-21	26-Nov-21		28-Nov-21
C19IMPACT_3114	Trinidad and Tobago	29-Oct-21	MoH	N	N	N	N	N	N	16.90		09-Nov-21	18-Nov-21	26-Nov-21		28-Nov-21
C19IMPACT_3115	Trinidad and Tobago	29-Oct-21	MoH	N	N	N	N	N	N	17.90		09-Nov-21	18-Nov-21	26-Nov-21		28-Nov-21
C19IMPACT_3116	Trinidad and Tobago	29-Oct-21	MoH	N	N	N	N	N	N	19.30		09-Nov-21	18-Nov-21	26-Nov-21		28-Nov-21
C19IMPACT_3117	Trinidad and Tobago	29-Oct-21	MoH	N	N	N	N	N	N		5.00	09-Nov-21	18-Nov-21	26-Nov-21		28-Nov-21
C19IMPACT_3118	Trinidad and Tobago	30-Oct-21	MoH	N	N	N	N	N	N	13.60		09-Nov-21	18-Nov-21	26-Nov-21		28-Nov-21
C19IMPACT_3119	Trinidad and Tobago	30-Oct-21	MoH	N	N	N	N	N	N	19.30		09-Nov-21	18-Nov-21	26-Nov-21		
C19IMPACT_3120	Trinidad and Tobago	30-Oct-21	MoH	N	N	N	N	N	N	13.20		09-Nov-21	18-Nov-21	26-Nov-21		28-Nov-21
C19IMPACT_3121	Trinidad and Tobago	30-Oct-21	MoH	N	N	N	N	N	N	14.70		09-Nov-21	18-Nov-21	26-Nov-21		28-Nov-21
C19IMPACT_3122	Trinidad and Tobago	30-Oct-21	MoH	N	N	N	N	N	N	19.80		09-Nov-21	18-Nov-21	26-Nov-21		28-Nov-21
C19IMPACT_3123	Trinidad and Tobago	31-Oct-21	MoH	N	N	N	N	N	N		16.00	09-Nov-21	18-Nov-21	26-Nov-21		
C19IMPACT_3124	Trinidad and Tobago	01-Nov-21	CARPHA	N	N	N	N	N	Y	17.51		09-Nov-21	18-Nov-21	26-Nov-21	26-Nov-21	28-Nov-21
C19IMPACT_3125	Trinidad and Tobago	01-Nov-21	CARPHA	N	N	N	N	N	Y	17.30		09-Nov-21	18-Nov-21	26-Nov-21	26-Nov-21	28-Nov-21
C19IMPACT_3126	Trinidad and Tobago	01-Nov-21	CARPHA	N	N	N	N	N	Y	22.27		09-Nov-21	18-Nov-21	26-Nov-21	26-Nov-21	28-Nov-21
C19IMPACT_3127	Trinidad and Tobago	01-Nov-21	MoH	N	N	N	N	N	N	16.00		09-Nov-21	18-Nov-21	26-Nov-21		28-Nov-21
C19IMPACT_3128	Trinidad and Tobago	01-Nov-21	MoH	N	N	N	N	N	N	18.00		09-Nov-21	18-Nov-21	26-Nov-21		28-Nov-21
C19IMPACT_3129	Trinidad and Tobago	01-Nov-21	MoH	N	N	N	N	N	N	16.00		09-Nov-21	18-Nov-21	26-Nov-21		28-Nov-21
C19IMPACT_3130	Trinidad and Tobago	01-Nov-21	MoH	N	N	N	N	N	N		11.00	09-Nov-21	18-Nov-21	26-Nov-21		
C19IMPACT_3131	Trinidad and Tobago	01-Nov-21	MoH	N	N	N	N	N	N		7.00	09-Nov-21	18-Nov-21	26-Nov-21		
C19IMPACT_3132	Trinidad and Tobago	02-Nov-21	CARPHA	N	N	N	N	N	N	14.72		09-Nov-21	18-Nov-21	26-Nov-21	26-Nov-21	28-Nov-21

C19IMPACT_3133	Trinidad and Tobago	02-Nov-21	CARPHA	N	N	N	N	N	Y	11.76		09-Nov-21	18-Nov-21	26-Nov-21	26-Nov-21	28-Nov-21
C19IMPACT_3134	Trinidad and Tobago	02-Nov-21	MoH	N	N	N	N	N	N	14.00		09-Nov-21	18-Nov-21	26-Nov-21		28-Nov-21
C19IMPACT_3135	Trinidad and Tobago	02-Nov-21	MoH	N	N	N	N	N	N	16.00		09-Nov-21	18-Nov-21	26-Nov-21		28-Nov-21
C19IMPACT_3136	Trinidad and Tobago	02-Nov-21	MoH	N	N	N	N	N	N	22.00		09-Nov-21	18-Nov-21	26-Nov-21		28-Nov-21
C19IMPACT_3137	Trinidad and Tobago	03-Nov-21	MoH	N	N	N	N	N	N	14.00		09-Nov-21	18-Nov-21	26-Nov-21		28-Nov-21
C19IMPACT_3138	Trinidad and Tobago	03-Nov-21	MoH	N	N	N	N	N	N	10.00		09-Nov-21	18-Nov-21	26-Nov-21		28-Nov-21
C19IMPACT_3139	Trinidad and Tobago	03-Nov-21	MoH	N	N	N	N	N	N	13.20		09-Nov-21	18-Nov-21	26-Nov-21		28-Nov-21
C19IMPACT_3140	Trinidad and Tobago	03-Nov-21	MoH	N	N	N	N	N	N	16.20		09-Nov-21	18-Nov-21	26-Nov-21		28-Nov-21
C19IMPACT_3141	Trinidad and Tobago	03-Nov-21	MoH	N	N	N	N	N	N	14.40		09-Nov-21	18-Nov-21	26-Nov-21		28-Nov-21
C19IMPACT_3142	Trinidad and Tobago	03-Nov-21	MoH	N	N	N	N	N	N	14.30		09-Nov-21	18-Nov-21	26-Nov-21		28-Nov-21
C19IMPACT_3143	Trinidad and Tobago	03-Nov-21	MoH	N	N	N	N	N	N	15.40		09-Nov-21	18-Nov-21	26-Nov-21		28-Nov-21
C19IMPACT_3144	Trinidad and Tobago	03-Nov-21	MoH	N	N	N	N	N	N	16.00		09-Nov-21	18-Nov-21	26-Nov-21		28-Nov-21
C19IMPACT_3145	Trinidad and Tobago	03-Nov-21	MoH	N	N	N	N	N	N	14.00		09-Nov-21	18-Nov-21	26-Nov-21		28-Nov-21
C19IMPACT_3146	Trinidad and Tobago	03-Nov-21	MoH	N	N	N	N	N	N	14.00		09-Nov-21	18-Nov-21	26-Nov-21		28-Nov-21
C19IMPACT_3147	Trinidad and Tobago	03-Nov-21	MoH	N	N	N	N	N	N	15.50		09-Nov-21	18-Nov-21	26-Nov-21		28-Nov-21
C19IMPACT_3148	Trinidad and Tobago	03-Nov-21	MoH	N	N	N	N	N	N	13.00		09-Nov-21	18-Nov-21	26-Nov-21		28-Nov-21
C19IMPACT_3149	Trinidad and Tobago	03-Nov-21	MoH	N	N	N	N	N	N	19.00		09-Nov-21	18-Nov-21	26-Nov-21		28-Nov-21
C19IMPACT_3150	Trinidad and Tobago	03-Nov-21	MoH	N	N	N	N	N	N	15.80		09-Nov-21	18-Nov-21	03-Dec-21		28-Nov-21
C19IMPACT_3151	Trinidad and Tobago	05-Nov-21	MoH	N	N	N	N	N	N		16.00	09-Nov-21	18-Nov-21	26-Nov-21		
C19IMPACT_3152	Trinidad and Tobago	05-Nov-21	MoH	N	N	N	N	N	N		5.00	09-Nov-21	18-Nov-21	26-Nov-21		28-Nov-21
C19IMPACT_3153	Trinidad and Tobago	05-Nov-21	MoH	N	N	N	N	N	N		6.00	09-Nov-21	18-Nov-21	26-Nov-21		28-Nov-21
C19IMPACT_3154	Trinidad and Tobago	05-Nov-21	MoH	N	N	N	N	N	N		7.00	09-Nov-21	18-Nov-21	26-Nov-21		28-Nov-21
C19IMPACT_3155	Trinidad and Tobago	06-Nov-21	MoH	N	N	N	N	N	N		16.00	09-Nov-21	18-Nov-21	26-Nov-21		
C19IMPACT_3156	Trinidad and Tobago	26-Oct-21	MoH	N	N	N	N	N	N	17.00		09-Nov-21	27-Nov-21	03-Dec-21		06-Dec-21
C19IMPACT_3157	Trinidad and Tobago	27-Oct-21	MoH	N	N	N	N	N	N	19.00		09-Nov-21	27-Nov-21	03-Dec-21		06-Dec-21
C19IMPACT_3158	Trinidad and Tobago	27-Oct-21	MoH	N	N	N	N	N	N	18.00		09-Nov-21	27-Nov-21	03-Dec-21		06-Dec-21
C19IMPACT_3159	Trinidad and Tobago	28-Oct-21	MoH	N	N	N	N	N	N	13.00		09-Nov-21	27-Nov-21	03-Dec-21		
C19IMPACT_3160	Trinidad and Tobago	28-Oct-21	MoH	N	N	N	N	N	N	11.30		09-Nov-21	27-Nov-21	03-Dec-21		06-Dec-21
C19IMPACT_3161	Trinidad and Tobago	28-Oct-21	MoH	N	N	N	N	N	N	16.80		09-Nov-21	27-Nov-21	03-Dec-21		06-Dec-21
C19IMPACT_3162	Trinidad and Tobago	28-Oct-21	MoH	N	N	N	N	N	N	12.50		09-Nov-21	27-Nov-21	03-Dec-21		
C19IMPACT_3163	Trinidad and Tobago	28-Oct-21	MoH	N	N	N	N	N	N	16.00		09-Nov-21	27-Nov-21	03-Dec-21		
C19IMPACT_3164	Trinidad and Tobago	28-Oct-21	MoH	N	N	N	N	N	N	16.00		09-Nov-21	27-Nov-21	03-Dec-21		06-Dec-21
C19IMPACT_3165	Trinidad and Tobago	28-Oct-21	MoH	N	N	N	N	N	N	16.00		09-Nov-21	27-Nov-21	03-Dec-21		06-Dec-21
C19IMPACT_3166	Trinidad and Tobago	28-Oct-21	MoH	N	N	N	N	N	N	17.00		09-Nov-21	27-Nov-21	03-Dec-21		06-Dec-21
C19IMPACT_3167	Anguilla	28-Oct-21	CARPHA	N	N	N	N	N	N	14.50		24-Nov-21	27-Nov-21		30-Nov-21	06-Dec-21
C19IMPACT_3168	Anguilla	28-Oct-21	CARPHA	N	N	N	N	N	N	10.99		24-Nov-21	27-Nov-21		30-Nov-21	06-Dec-21
C19IMPACT_3169	Anguilla	28-Oct-21	CARPHA	N	N	N	N	N	N	12.76		24-Nov-21	27-Nov-21		30-Nov-21	06-Dec-21
C19IMPACT_3170	Trinidad and Tobago	29-Oct-21	MoH	N	N	N	N	N	N	19.00		09-Nov-21	27-Nov-21	03-Dec-21		06-Dec-21
C19IMPACT_3171	Trinidad and Tobago	29-Oct-21	MoH	N	N	N	N	N	N	17.00		09-Nov-21	27-Nov-21	03-Dec-21		06-Dec-21
C19IMPACT_3172	Anguilla	29-Oct-21	CARPHA	N	N	N	N	N	N	12.29		24-Nov-21	27-Nov-21		30-Nov-21	06-Dec-21
C19IMPACT_3173	Anguilla	29-Oct-21	CARPHA	N	N	N	N	N	N	13.09		24-Nov-21	27-Nov-21		30-Nov-21	
C19IMPACT_3174	Anguilla	29-Oct-21	CARPHA	N	N	N	N	N	N	11.96		24-Nov-21	27-Nov-21		30-Nov-21	06-Dec-21
C19IMPACT_3175	Trinidad and Tobago	30-Oct-21	MoH	N	N	N	N	N	N	13.50		09-Nov-21	27-Nov-21	03-Dec-21		06-Dec-21
C19IMPACT_3176	Anguilla	30-Oct-21	CARPHA	N	N	N	N	N	N	14.14		24-Nov-21	27-Nov-21		30-Nov-21	06-Dec-21
C19IMPACT_3177	Anguilla	30-Oct-21	CARPHA	N	N	N	N	N	N	17.04		24-Nov-21	27-Nov-21		30-Nov-21	06-Dec-21
C19IMPACT_3178	Anguilla	31-Oct-21	CARPHA	N	N	N	N	N	N	13.64		24-Nov-21	27-Nov-21		30-Nov-21	06-Dec-21
C19IMPACT_3179	Anguilla	31-Oct-21	CARPHA	N	N	N	N	N	N	12.63		24-Nov-21	27-Nov-21		30-Nov-21	06-Dec-21
C19IMPACT_3180	Trinidad and Tobago	01-Nov-21	MoH	N	N	N	N	N	N	18.00		09-Nov-21	27-Nov-21	03-Dec-21		06-Dec-21
C19IMPACT_3181	Trinidad and Tobago	05-Nov-21	CARPHA	N	N	N	N	N	N	12.73		16-Nov-21	27-Nov-21	03-Dec-21	30-Nov-21	06-Dec-21
C19IMPACT_3182	Trinidad and Tobago	08-Nov-21	CARPHA	N	N	N	N	N	N	20.56		16-Nov-21	27-Nov-21	03-Dec-21	30-Nov-21	06-Dec-21
C19IMPACT_3183	Trinidad and Tobago	08-Nov-21	CARPHA	N	N	N	N	N	N	10.34		16-Nov-21	27-Nov-21	03-Dec-21	30-Nov-21	06-Dec-21
C19IMPACT_3184	Trinidad and Tobago	08-Nov-21	CARPHA	N	N	N	N	N	N	13.55		16-Nov-21	27-Nov-21	03-Dec-21	30-Nov-21	06-Dec-21
C19IMPACT_3185	Trinidad and Tobago	08-Nov-21	CARPHA	N	N	N	N	N	N	18.15		16-Nov-21	27-Nov-21	03-Dec-21	30-Nov-21	06-Dec-21
C19IMPACT_3186	Trinidad and Tobago	08-Nov-21	CARPHA	N	N	N	N	N	N	16.66		16-Nov-21	27-Nov-21	03-Dec-21	30-Nov-21	06-Dec-21
C19IMPACT_3187	Trinidad and Tobago	08-Nov-21	CARPHA	N	N	N	N	N	N	12.19		16-Nov-21	27-Nov-21	03-Dec-21	30-Nov-21	06-Dec-21
C19IMPACT_3188	Trinidad and Tobago	08-Nov-21	CARPHA	N	N	N	N	N	N	21.82		16-Nov-21	27-Nov-21	03-Dec-21	30-Nov-21	06-Dec-21
C19IMPACT_3189	Trinidad and Tobago	08-Nov-21	CARPHA	N	N	N	N	N	N	14.49		16-Nov-21	27-Nov-21	03-Dec-21	30-Nov-21	06-Dec-21
C19IMPACT_3190	Trinidad and Tobago	08-Nov-21	CARPHA	N	N	N	N	N	N	14.51		16-Nov-21	27-Nov-21	03-Dec-21	30-Nov-21	06-Dec-21
C19IMPACT_3191	Trinidad and Tobago	08-Nov-21	CARPHA	N	N	N	N	N	N	12.72		16-Nov-21	27-Nov-21	03-Dec-21	30-Nov-21	06-Dec-21
C19IMPACT_3192	Trinidad and Tobago	08-Nov-21	CARPHA	N	N	N	N	N	N	16.71		16-Nov-21	27-Nov-21	03-Dec-21	30-Nov-21	
C19IMPACT_3193	Trinidad and Tobago	08-Nov-21	CARPHA	N	N	N	N	N	N	18.92		16-Nov-21	27-Nov-21	03-Dec-21	30-Nov-21	06-Dec-21
C19IMPACT_3194	Trinidad and Tobago	08-Nov-21	CARPHA	N	N	N	N	N	N	18.22		16-Nov-21	27-Nov-21	03-Dec-21	30-Nov-21	06-Dec-21
C19IMPACT_3195	Trinidad and Tobago	09-Nov-21	CARPHA	N	N	N	N	N	N	20.76		16-Nov-21	27-Nov-21	03-Dec-21	30-Nov-21	06-Dec-21
C19IMPACT_3196	Trinidad and Tobago	09-Nov-21	CARPHA	N	N	N	N	N	N	20.56		16-Nov-21	27-Nov-21	03-Dec-21	30-Nov-21	06-Dec-21

C19IMPACT_3197	Trinidad and Tobago	09-Nov-21	CARPHA	N	N	N	N	N	N	13.54		16-Nov-21	27-Nov-21	03-Dec-21	30-Nov-21	06-Dec-21
C19IMPACT_3198	Trinidad and Tobago	09-Nov-21	CARPHA	N	N	N	N	N	N	20.38		16-Nov-21	27-Nov-21	03-Dec-21	30-Nov-21	06-Dec-21
C19IMPACT_3199	Trinidad and Tobago	09-Nov-21	CARPHA	N	N	N	N	N	N	22.92		16-Nov-21	27-Nov-21	03-Dec-21	30-Nov-21	
C19IMPACT_3200	Trinidad and Tobago	09-Nov-21	CARPHA	N	N	N	N	N	N	12.28		16-Nov-21	27-Nov-21	03-Dec-21	30-Nov-21	06-Dec-21
C19IMPACT_3201	Trinidad and Tobago	09-Nov-21	CARPHA	N	N	N	N	N	N	18.84		16-Nov-21	27-Nov-21	03-Dec-21	30-Nov-21	06-Dec-21
C19IMPACT_3202	Trinidad and Tobago	09-Nov-21	CARPHA	N	N	N	N	N	N	15.29		16-Nov-21	27-Nov-21	03-Dec-21	30-Nov-21	06-Dec-21
C19IMPACT_3203	Trinidad and Tobago	09-Nov-21	CARPHA	N	N	N	N	N	N	14.63		16-Nov-21	27-Nov-21	03-Dec-21	30-Nov-21	06-Dec-21
C19IMPACT_3204	Trinidad and Tobago	09-Nov-21	CARPHA	N	N	N	N	N	N	12.90		16-Nov-21	27-Nov-21	03-Dec-21	30-Nov-21	06-Dec-21
C19IMPACT_3205	Trinidad and Tobago	09-Nov-21	CARPHA	N	N	N	N	N	N	14.63		16-Nov-21	27-Nov-21	03-Dec-21	30-Nov-21	06-Dec-21
C19IMPACT_3206	Trinidad and Tobago	09-Nov-21	CARPHA	N	N	N	N	N	N	13.62		16-Nov-21	27-Nov-21	03-Dec-21	30-Nov-21	
C19IMPACT_3207	Trinidad and Tobago	09-Nov-21	CARPHA	N	N	N	N	N	N	15.82		16-Nov-21	27-Nov-21	03-Dec-21	30-Nov-21	06-Dec-21
C19IMPACT_3208	Trinidad and Tobago	09-Nov-21	CARPHA	N	N	N	N	N	N	19.42		16-Nov-21	27-Nov-21	03-Dec-21	30-Nov-21	06-Dec-21
C19IMPACT_3209	Trinidad and Tobago	09-Nov-21	CARPHA	N	N	N	N	N	N	17.32		16-Nov-21	27-Nov-21	03-Dec-21	30-Nov-21	06-Dec-21
C19IMPACT_3210	Trinidad and Tobago	10-Nov-21	CARPHA	N	N	N	N	N	N	18.18		16-Nov-21	27-Nov-21	03-Dec-21	30-Nov-21	06-Dec-21
C19IMPACT_3211	Trinidad and Tobago	10-Nov-21	CARPHA	N	N	N	N	N	N	14.51		16-Nov-21	27-Nov-21	03-Dec-21	30-Nov-21	06-Dec-21
C19IMPACT_3212	Trinidad and Tobago	10-Nov-21	CARPHA	N	N	N	N	N	N	15.41		16-Nov-21	27-Nov-21	03-Dec-21	30-Nov-21	06-Dec-21
C19IMPACT_3213	Trinidad and Tobago	10-Nov-21	CARPHA	N	N	N	N	N	N	19.38		16-Nov-21	27-Nov-21	03-Dec-21	30-Nov-21	06-Dec-21
C19IMPACT_3214	Trinidad and Tobago	10-Nov-21	CARPHA	N	N	N	N	N	Y	29.74		24-Nov-21	27-Nov-21	03-Dec-21	30-Nov-21	
C19IMPACT_3215	Trinidad and Tobago	11-Nov-21	CARPHA	N	N	N	N	N	N	12.59		24-Nov-21	27-Nov-21	03-Dec-21	30-Nov-21	06-Dec-21
C19IMPACT_3216	Trinidad and Tobago	11-Nov-21	CARPHA	N	N	N	N	N	Y	13.46		24-Nov-21	27-Nov-21	03-Dec-21	30-Nov-21	06-Dec-21
C19IMPACT_3217	Trinidad and Tobago	11-Nov-21	CARPHA	N	N	N	N	N	Y	13.89		24-Nov-21	27-Nov-21	03-Dec-21	30-Nov-21	06-Dec-21
C19IMPACT_3218	Trinidad and Tobago	11-Nov-21	CARPHA	N	N	N	N	N	Y	19.07		24-Nov-21	27-Nov-21	03-Dec-21	30-Nov-21	06-Dec-21
C19IMPACT_3219	Trinidad and Tobago	18-Nov-21	CARPHA	N	N	N	N	N	Y	15.85		24-Nov-21	27-Nov-21	03-Dec-21	30-Nov-21	06-Dec-21
C19IMPACT_3220	Trinidad and Tobago	19-Nov-21	CARPHA	N	N	N	N	N	N	15.47		24-Nov-21	27-Nov-21	03-Dec-21	30-Nov-21	06-Dec-21
C19IMPACT_3221	Trinidad and Tobago	20-Nov-21	CARPHA	N	N	N	N	N	N	15.38		24-Nov-21	27-Nov-21	03-Dec-21	30-Nov-21	06-Dec-21
C19IMPACT_3222	Trinidad and Tobago	01-Nov-21	MoH	N	N	N	N	N	Y		16.11	24-Nov-21	02-Dec-21	03-Dec-21		13-Dec-21
C19IMPACT_3223	Trinidad and Tobago	01-Nov-21	MoH	N	N	N	N	N	Y		14.85	24-Nov-21	02-Dec-21	03-Dec-21		13-Dec-21
C19IMPACT_3224	Trinidad and Tobago	03-Nov-21	MoH	N	N	N	N	N	Y		9.17	24-Nov-21	02-Dec-21	03-Dec-21		13-Dec-21
C19IMPACT_3225	Trinidad and Tobago	03-Nov-21	MoH	N	N	N	N	N	Y		11.24	24-Nov-21	02-Dec-21	03-Dec-21		13-Dec-21
C19IMPACT_3226	Trinidad and Tobago	03-Nov-21	MoH	N	N	N	N	N	Y		13.53	24-Nov-21	02-Dec-21	03-Dec-21		13-Dec-21
C19IMPACT_3227	Trinidad and Tobago	03-Nov-21	MoH	N	N	N	N	N	Y		22.10	24-Nov-21	02-Dec-21	03-Dec-21		
C19IMPACT_3228	Trinidad and Tobago	03-Nov-21	MoH	N	N	N	N	N	Y		2.95	24-Nov-21	02-Dec-21	03-Dec-21		13-Dec-21
C19IMPACT_3229	Trinidad and Tobago	03-Nov-21	MoH	N	N	N	N	N	Y		7.02	24-Nov-21	02-Dec-21	03-Dec-21		
C19IMPACT_3230	Trinidad and Tobago	03-Nov-21	MoH	N	N	N	N	N	Y		9.54	24-Nov-21	02-Dec-21	03-Dec-21		13-Dec-21
C19IMPACT_3231	Trinidad and Tobago	04-Nov-21	MoH	N	N	N	N	N	Y		10.41	24-Nov-21	02-Dec-21	03-Dec-21		13-Dec-21
C19IMPACT_3232	Trinidad and Tobago	04-Nov-21	MoH	N	N	N	N	N	Y		9.65	24-Nov-21	02-Dec-21	03-Dec-21		13-Dec-21
C19IMPACT_3233	Trinidad and Tobago	04-Nov-21	MoH	N	N	N	N	N	Y		3.66	24-Nov-21	02-Dec-21	03-Dec-21		13-Dec-21
C19IMPACT_3234	Trinidad and Tobago	05-Nov-21	MoH	N	N	N	N	N	Y		14.00	24-Nov-21	02-Dec-21	03-Dec-21		13-Dec-21
C19IMPACT_3235	Trinidad and Tobago	05-Nov-21	MoH	N	N	N	N	N	Y		5.47	24-Nov-21	02-Dec-21	03-Dec-21		13-Dec-21
C19IMPACT_3236	Trinidad and Tobago	06-Nov-21	MoH	N	N	N	N	N	Y		5.29	24-Nov-21	02-Dec-21	03-Dec-21		13-Dec-21
C19IMPACT_3237	Trinidad and Tobago	06-Nov-21	MoH	N	N	N	N	N	Y		4.71	24-Nov-21	02-Dec-21	03-Dec-21		13-Dec-21
C19IMPACT_3238	Trinidad and Tobago	07-Nov-21	MoH	N	N	N	N	N	Y		6.00	18-Nov-21	02-Dec-21	03-Dec-21		13-Dec-21
C19IMPACT_3239	Trinidad and Tobago	07-Nov-21	MoH	N	N	N	N	N	Y		11.84	24-Nov-21	02-Dec-21	03-Dec-21		13-Dec-21
C19IMPACT_3240	Trinidad and Tobago	07-Nov-21	MoH	N	N	N	N	N	Y		2.95	24-Nov-21	02-Dec-21	03-Dec-21		13-Dec-21
C19IMPACT_3241	Trinidad and Tobago	08-Nov-21	MoH	N	N	N	N	N	Y		11.00	18-Nov-21	02-Dec-21	03-Dec-21		13-Dec-21
C19IMPACT_3242	Trinidad and Tobago	08-Nov-21	MoH	N	N	N	N	N	Y		13.00	18-Nov-21	02-Dec-21	03-Dec-21		13-Dec-21
C19IMPACT_3243	Trinidad and Tobago	08-Nov-21	MoH	N	N	N	N	N	Y	15.10		18-Nov-21	02-Dec-21	03-Dec-21		13-Dec-21
C19IMPACT_3244	Trinidad and Tobago	08-Nov-21	MoH	N	N	N	N	N	Y	11.80		18-Nov-21	02-Dec-21	03-Dec-21		13-Dec-21
C19IMPACT_3245	Trinidad and Tobago	08-Nov-21	MoH	N	N	N	N	N	Y		8.00	18-Nov-21	02-Dec-21	03-Dec-21		13-Dec-21
C19IMPACT_3246	Trinidad and Tobago	08-Nov-21	MoH	N	N	N	N	N	Y		16.00	24-Nov-21	02-Dec-21	03-Dec-21		13-Dec-21
C19IMPACT_3247	Trinidad and Tobago	08-Nov-21	MoH	N	N	N	N	N	Y		7.13	24-Nov-21	02-Dec-21	03-Dec-21		13-Dec-21
C19IMPACT_3248	Trinidad and Tobago	08-Nov-21	MoH	N	N	N	N	N	Y		10.49	24-Nov-21	02-Dec-21	03-Dec-21		13-Dec-21
C19IMPACT_3249	Trinidad and Tobago	08-Nov-21	MoH	N	N	N	N	N	Y		6.39	24-Nov-21	02-Dec-21	03-Dec-21		13-Dec-21
C19IMPACT_3250	Trinidad and Tobago	08-Nov-21	MoH	N	N	N	N	N	Y		13.47	24-Nov-21	02-Dec-21	03-Dec-21		13-Dec-21
C19IMPACT_3251	Trinidad and Tobago	08-Nov-21	MoH	N	N	N	N	N	Y		12.92	24-Nov-21	02-Dec-21	03-Dec-21		13-Dec-21
C19IMPACT_3252	Trinidad and Tobago	09-Nov-21	MoH	N	N	N	N	N	Y	12.80		18-Nov-21	02-Dec-21	03-Dec-21		13-Dec-21
C19IMPACT_3253	Trinidad and Tobago	09-Nov-21	MoH	N	N	N	N	N	Y	11.40		18-Nov-21	02-Dec-21	03-Dec-21		13-Dec-21
C19IMPACT_3254	Trinidad and Tobago	09-Nov-21	MoH	N	N	N	N	N	Y		12.00	18-Nov-21	02-Dec-21	03-Dec-21		13-Dec-21
C19IMPACT_3255	Trinidad and Tobago	09-Nov-21	MoH	N	N	N	N	N	Y		11.00	18-Nov-21	02-Dec-21	03-Dec-21		13-Dec-21
C19IMPACT_3256	Trinidad and Tobago	09-Nov-21	MoH	N	N	N	N	N	Y		6.50	24-Nov-21	02-Dec-21	03-Dec-21		13-Dec-21
C19IMPACT_3257	Trinidad and Tobago	09-Nov-21	MoH	N	N	N	N	N	Y	16.00		24-Nov-21	02-Dec-21	03-Dec-21		13-Dec-21
C19IMPACT_3258	Trinidad and Tobago	10-Nov-21	MoH	N	N	N	N	N	Y	15.29		18-Nov-21	02-Dec-21	03-Dec-21		13-Dec-21
C19IMPACT_3259	Trinidad and Tobago	10-Nov-21	MoH	N	N	N	N	N	Y	15.00		18-Nov-21	02-Dec-21	03-Dec-21		13-Dec-21
C19IMPACT_3260	Trinidad and Tobago	10-Nov-21	MoH	N	N	N	N	N	Y		3.00	18-Nov-21	02-Dec-21	03-Dec-21		13-Dec-21

C19IMPACT_3261	Trinidad and Tobago	10-Nov-21	MoH	N	N	N	N	N	Y		4.00	18-Nov-21	02-Dec-21	03-Dec-21		13-Dec-21
C19IMPACT_3262	Trinidad and Tobago	10-Nov-21	MoH	N	N	N	N	N	Y		6.00	18-Nov-21	02-Dec-21	03-Dec-21		13-Dec-21
C19IMPACT_3263	Trinidad and Tobago	10-Nov-21	MoH	N	N	N	N	N	Y	14.00		24-Nov-21	02-Dec-21	03-Dec-21		13-Dec-21
C19IMPACT_3264	Trinidad and Tobago	10-Nov-21	MoH	N	N	N	N	N	Y	16.00		24-Nov-21	02-Dec-21	03-Dec-21		13-Dec-21
C19IMPACT_3265	Trinidad and Tobago	11-Nov-21	MoH	N	N	N	N	N	Y	12.80		18-Nov-21	02-Dec-21	03-Dec-21		13-Dec-21
C19IMPACT_3266	Trinidad and Tobago	11-Nov-21	MoH	N	N	N	N	N	Y	12.20		18-Nov-21	02-Dec-21	03-Dec-21		13-Dec-21
C19IMPACT_3267	Trinidad and Tobago	11-Nov-21	MoH	N	N	N	N	N	Y	11.90		18-Nov-21	02-Dec-21	03-Dec-21		13-Dec-21
C19IMPACT_3268	Trinidad and Tobago	11-Nov-21	MoH	N	N	N	N	N	Y	12.40		18-Nov-21	02-Dec-21	03-Dec-21		13-Dec-21
C19IMPACT_3269	Trinidad and Tobago	11-Nov-21	MoH	N	N	N	N	N	Y		4.00	18-Nov-21	02-Dec-21	03-Dec-21		13-Dec-21
C19IMPACT_3270	Trinidad and Tobago	11-Nov-21	MoH	N	N	N	N	N	Y	17.00		24-Nov-21	02-Dec-21	03-Dec-21		13-Dec-21
C19IMPACT_3271	Trinidad and Tobago	12-Nov-21	MoH	N	N	N	N	N	Y	17.00		24-Nov-21	02-Dec-21	03-Dec-21		13-Dec-21
C19IMPACT_3272	Trinidad and Tobago	12-Nov-21	MoH	N	N	N	N	N	Y	17.00		24-Nov-21	02-Dec-21	03-Dec-21		13-Dec-21
C19IMPACT_3273	Trinidad and Tobago	12-Nov-21	MoH	N	N	N	N	N	Y	18.00		24-Nov-21	02-Dec-21	03-Dec-21		13-Dec-21
C19IMPACT_3274	Trinidad and Tobago	12-Nov-21	MoH	N	N	N	N	N	Y	19.00		24-Nov-21	02-Dec-21	03-Dec-21		13-Dec-21
C19IMPACT_3275	Trinidad and Tobago	12-Nov-21	MoH	N	N	N	N	N	Y	13.00		24-Nov-21	02-Dec-21	03-Dec-21		13-Dec-21
C19IMPACT_3276	Trinidad and Tobago	12-Nov-21	MoH	N	N	N	N	N	Y	15.00		24-Nov-21	02-Dec-21	03-Dec-21		13-Dec-21
C19IMPACT_3277	Trinidad and Tobago	12-Nov-21	MoH	N	N	N	N	N	Y		3.00	24-Nov-21	02-Dec-21	03-Dec-21		13-Dec-21
C19IMPACT_3278	Trinidad and Tobago	12-Nov-21	MoH	N	N	N	N	N	Y		9.00	24-Nov-21	02-Dec-21	03-Dec-21		13-Dec-21
C19IMPACT_3279	Trinidad and Tobago	14-Nov-21	MoH	N	N	N	N	N	Y	14.00		24-Nov-21	02-Dec-21	03-Dec-21		13-Dec-21
C19IMPACT_3280	Trinidad and Tobago	14-Nov-21	MoH	N	N	N	N	N	Y	13.00		24-Nov-21	02-Dec-21	03-Dec-21		13-Dec-21
C19IMPACT_3281	Trinidad and Tobago	15-Nov-21	MoH	N	N	N	N	N	Y		7.00	24-Nov-21	02-Dec-21	03-Dec-21		13-Dec-21
C19IMPACT_3282	Trinidad and Tobago	18-Nov-21	CARPHA	N	N	N	N	N	N	15.27		24-Nov-21	02-Dec-21	03-Dec-21	03-Dec-21	13-Dec-21
C19IMPACT_3283	Trinidad and Tobago	19-Nov-21	CARPHA	N	N	N	N	N	Y	22.87		24-Nov-21	02-Dec-21	03-Dec-21	03-Dec-21	13-Dec-21
C19IMPACT_3284	Trinidad and Tobago	19-Nov-21	CARPHA	N	N	N	N	N	Y	20.32		24-Nov-21	02-Dec-21	03-Dec-21	03-Dec-21	13-Dec-21
C19IMPACT_3285	Trinidad and Tobago	20-Nov-21	CARPHA	N	N	N	N	N	N	21.44		24-Nov-21	02-Dec-21	03-Dec-21	03-Dec-21	13-Dec-21
C19IMPACT_3286	Trinidad and Tobago	10-Nov-21	MoH	N	N	N	N	N	Y		14.00	02-Dec-21	04-Dec-21	10-Dec-21		07-Dec-21
C19IMPACT_3287	Trinidad and Tobago	10-Nov-21	MoH	N	N	N	N	N	Y		26.07	02-Dec-21	04-Dec-21	10-Dec-21		
C19IMPACT_3288	Trinidad and Tobago	10-Nov-21	MoH	N	N	N	N	N	Y		13.86	02-Dec-21	04-Dec-21	10-Dec-21		
C19IMPACT_3289	Trinidad and Tobago	11-Nov-21	MoH	N	N	N	N	N	Y		6.00	02-Dec-21	04-Dec-21	10-Dec-21		07-Dec-21
C19IMPACT_3290	Trinidad and Tobago	11-Nov-21	MoH	N	N	N	N	N	Y		9.00	02-Dec-21	04-Dec-21	10-Dec-21		07-Dec-21
C19IMPACT_3291	Trinidad and Tobago	11-Nov-21	MoH	N	N	N	N	N	Y		8.00	02-Dec-21	04-Dec-21	10-Dec-21		07-Dec-21
C19IMPACT_3292	Trinidad and Tobago	11-Nov-21	MoH	N	N	N	N	N	Y		5.00	02-Dec-21	04-Dec-21	10-Dec-21		07-Dec-21
C19IMPACT_3293	Trinidad and Tobago	11-Nov-21	MoH	N	N	N	N	N	Y		3.87	02-Dec-21	04-Dec-21	10-Dec-21		07-Dec-21
C19IMPACT_3294	Trinidad and Tobago	11-Nov-21	MoH	N	N	N	N	N	Y		10.13	02-Dec-21	04-Dec-21	10-Dec-21		07-Dec-21
C19IMPACT_3295	Trinidad and Tobago	11-Nov-21	MoH	N	N	N	N	N	Y		21.54	02-Dec-21	04-Dec-21	10-Dec-21		
C19IMPACT_3296	Trinidad and Tobago	11-Nov-21	MoH	N	N	N	N	N	Y		5.40	02-Dec-21	04-Dec-21	10-Dec-21		07-Dec-21
C19IMPACT_3297	Trinidad and Tobago	11-Nov-21	MoH	N	N	N	N	N	Y		9.76	02-Dec-21	04-Dec-21	10-Dec-21		07-Dec-21
C19IMPACT_3298	Trinidad and Tobago	11-Nov-21	MoH	N	N	N	N	N	Y		4.03	02-Dec-21	04-Dec-21	10-Dec-21		07-Dec-21
C19IMPACT_3299	Trinidad and Tobago	12-Nov-21	MoH	N	N	N	N	N	Y		3.00	02-Dec-21	04-Dec-21	10-Dec-21		07-Dec-21
C19IMPACT_3300	Trinidad and Tobago	12-Nov-21	MoH	N	N	N	N	N	Y		5.00	02-Dec-21	04-Dec-21	10-Dec-21		07-Dec-21
C19IMPACT_3301	Trinidad and Tobago	12-Nov-21	MoH	N	N	N	N	N	Y		5.00	02-Dec-21	04-Dec-21	10-Dec-21		
C19IMPACT_3302	Trinidad and Tobago	12-Nov-21	MoH	N	N	N	N	N	Y	12.10		02-Dec-21	04-Dec-21	10-Dec-21		07-Dec-21
C19IMPACT_3303	Trinidad and Tobago	12-Nov-21	MoH	N	N	N	N	N	Y	14.40		02-Dec-21	04-Dec-21	10-Dec-21		07-Dec-21
C19IMPACT_3304	Trinidad and Tobago	12-Nov-21	MoH	N	N	N	N	N	Y		11.20	02-Dec-21	04-Dec-21	10-Dec-21		07-Dec-21
C19IMPACT_3305	Trinidad and Tobago	12-Nov-21	MoH	N	N	N	N	N	Y		20.37	02-Dec-21	04-Dec-21	10-Dec-21		07-Dec-21
C19IMPACT_3306	Trinidad and Tobago	12-Nov-21	MoH	N	N	N	N	N	Y		26.34	02-Dec-21	04-Dec-21	10-Dec-21		
C19IMPACT_3307	Trinidad and Tobago	12-Nov-21	MoH	N	N	N	N	N	Y		3.40	02-Dec-21	04-Dec-21	10-Dec-21		07-Dec-21
C19IMPACT_3308	Trinidad and Tobago	12-Nov-21	MoH	N	N	N	N	N	Y		3.77	02-Dec-21	04-Dec-21	10-Dec-21		07-Dec-21
C19IMPACT_3309	Trinidad and Tobago	12-Nov-21	MoH	N	N	N	N	N	Y		8.45	02-Dec-21	04-Dec-21	10-Dec-21		07-Dec-21
C19IMPACT_3310	Trinidad and Tobago	12-Nov-21	MoH	N	N	N	N	N	Y		7.58	02-Dec-21	04-Dec-21	10-Dec-21		07-Dec-21
C19IMPACT_3311	Trinidad and Tobago	13-Nov-21	MoH	N	N	N	N	N	Y	16.00		02-Dec-21	04-Dec-21	10-Dec-21		07-Dec-21
C19IMPACT_3312	Trinidad and Tobago	13-Nov-21	MoH	N	N	N	N	N	Y		6.00	02-Dec-21	04-Dec-21	10-Dec-21		07-Dec-21
C19IMPACT_3313	Trinidad and Tobago	14-Nov-21	MoH	N	N	N	N	N	Y		14.00	02-Dec-21	04-Dec-21	10-Dec-21		07-Dec-21
C19IMPACT_3314	Trinidad and Tobago	14-Nov-21	MoH	N	N	N	N	N	Y	10.70		02-Dec-21	04-Dec-21	10-Dec-21		07-Dec-21
C19IMPACT_3315	Trinidad and Tobago	14-Nov-21	MoH	N	N	N	N	N	Y	12.70		02-Dec-21	04-Dec-21	10-Dec-21		07-Dec-21
C19IMPACT_3316	Trinidad and Tobago	14-Nov-21	MoH	N	N	N	N	N	Y		8.71	02-Dec-21	04-Dec-21	10-Dec-21		
C19IMPACT_3317	Trinidad and Tobago	14-Nov-21	MoH	N	N	N	N	N	Y		10.30	02-Dec-21	04-Dec-21	10-Dec-21		07-Dec-21
C19IMPACT_3318	Trinidad and Tobago	14-Nov-21	MoH	N	N	N	N	N	Y		10.76	02-Dec-21	04-Dec-21	10-Dec-21		07-Dec-21
C19IMPACT_3319	Trinidad and Tobago	14-Nov-21	MoH	N	N	N	N	N	Y		6.88	02-Dec-21	04-Dec-21	10-Dec-21		07-Dec-21
C19IMPACT_3320	Trinidad and Tobago	15-Nov-21	MoH	N	N	N	N	N	Y		4.00	02-Dec-21	04-Dec-21	10-Dec-21		07-Dec-21
C19IMPACT_3321	Trinidad and Tobago	15-Nov-21	MoH	N	N	N	N	N	Y		5.00	02-Dec-21	04-Dec-21	10-Dec-21		07-Dec-21
C19IMPACT_3322	Trinidad and Tobago	15-Nov-21	MoH	N	N	N	N	N	Y		10.38	02-Dec-21	04-Dec-21	10-Dec-21		07-Dec-21
C19IMPACT_3323	Trinidad and Tobago	15-Nov-21	MoH	N	N	N	N	N	Y			02-Dec-21	04-Dec-21	10-Dec-21		07-Dec-21
C19IMPACT_3324	Trinidad and Tobago	16-Nov-21	MoH	N	N	N	N	N	Y		8.02	02-Dec-21	04-Dec-21	10-Dec-21		07-Dec-21

C19IMPACT_3325	Trinidad and Tobago	16-Nov-21	MoH	N	N	N	N	N	Y		8.76	02-Dec-21	04-Dec-21	10-Dec-21	07-Dec-21
C19IMPACT_3326	Trinidad and Tobago	16-Nov-21	MoH	N	N	N	N	N	Y		5.66	02-Dec-21	04-Dec-21	10-Dec-21	07-Dec-21
C19IMPACT_3327	Trinidad and Tobago	16-Nov-21	MoH	N	N	N	N	N	Y		7.56	02-Dec-21	04-Dec-21	10-Dec-21	07-Dec-21
C19IMPACT_3328	Trinidad and Tobago	16-Nov-21	MoH	N	N	N	N	N	Y		23.94	02-Dec-21	04-Dec-21	10-Dec-21	
C19IMPACT_3329	Trinidad and Tobago	17-Nov-21	MoH	N	N	N	N	N	Y		4.45	02-Dec-21	04-Dec-21	10-Dec-21	07-Dec-21
C19IMPACT_3330	Trinidad and Tobago	12-Nov-21	MoH	N	N	N	N	N	N	19.00		06-Dec-21	07-Dec-21	10-Dec-21	13-Dec-21
C19IMPACT_3331	Trinidad and Tobago	14-Nov-21	MoH	N	N	N	N	N	N	17.00		06-Dec-21	07-Dec-21	10-Dec-21	
C19IMPACT_3332	Trinidad and Tobago	15-Nov-21	MoH	N	N	N	N	N	Y	17.00		02-Dec-21	07-Dec-21	10-Dec-21	13-Dec-21
C19IMPACT_3333	Trinidad and Tobago	15-Nov-21	MoH	N	N	N	N	N	N	18.00		06-Dec-21	07-Dec-21	10-Dec-21	13-Dec-21
C19IMPACT_3334	Trinidad and Tobago	15-Nov-21	MoH	N	N	N	N	N	N	16.00		06-Dec-21	07-Dec-21	10-Dec-21	13-Dec-21
C19IMPACT_3335	Trinidad and Tobago	16-Nov-21	MoH	N	N	N	N	N	Y		14.00	02-Dec-21	07-Dec-21	10-Dec-21	
C19IMPACT_3336	Trinidad and Tobago	16-Nov-21	MoH	N	N	N	N	N	Y		7.00	02-Dec-21	07-Dec-21	10-Dec-21	13-Dec-21
C19IMPACT_3337	Trinidad and Tobago	16-Nov-21	MoH	N	N	N	N	N	Y		9.00	02-Dec-21	07-Dec-21	10-Dec-21	13-Dec-21
C19IMPACT_3338	Trinidad and Tobago	16-Nov-21	MoH	N	N	N	N	N	Y	12.80		02-Dec-21	07-Dec-21	10-Dec-21	13-Dec-21
C19IMPACT_3339	Trinidad and Tobago	16-Nov-21	MoH	N	N	N	N	N	Y	13.20		02-Dec-21	07-Dec-21	10-Dec-21	13-Dec-21
C19IMPACT_3340	Trinidad and Tobago	16-Nov-21	MoH	N	N	N	N	N	N	18.00		06-Dec-21	07-Dec-21	10-Dec-21	13-Dec-21
C19IMPACT_3341	Trinidad and Tobago	16-Nov-21	MoH	N	N	N	N	N	N	17.00		06-Dec-21	07-Dec-21	10-Dec-21	13-Dec-21
C19IMPACT_3342	Trinidad and Tobago	16-Nov-21	MoH	N	N	N	N	N	N	15.00		06-Dec-21	07-Dec-21	10-Dec-21	
C19IMPACT_3343	Trinidad and Tobago	17-Nov-21	MoH	N	N	N	N	N	Y		23.49	02-Dec-21	07-Dec-21	10-Dec-21	
C19IMPACT_3344	Trinidad and Tobago	17-Nov-21	MoH	N	N	N	N	N	Y		3.24	02-Dec-21	07-Dec-21	10-Dec-21	13-Dec-21
C19IMPACT_3345	Trinidad and Tobago	17-Nov-21	MoH	N	N	N	N	N	Y	12.10		02-Dec-21	07-Dec-21	10-Dec-21	13-Dec-21
C19IMPACT_3346	Trinidad and Tobago	17-Nov-21	MoH	N	N	N	N	N	Y	13.90		02-Dec-21	07-Dec-21	10-Dec-21	13-Dec-21
C19IMPACT_3347	Trinidad and Tobago	17-Nov-21	MoH	N	N	N	N	N	Y	13.70		02-Dec-21	07-Dec-21	10-Dec-21	13-Dec-21
C19IMPACT_3348	Trinidad and Tobago	17-Nov-21	MoH	N	N	N	N	N	N	17.00		06-Dec-21	07-Dec-21	10-Dec-21	13-Dec-21
C19IMPACT_3349	Trinidad and Tobago	17-Nov-21	MoH	N	N	N	N	N	N	15.00		06-Dec-21	07-Dec-21	10-Dec-21	13-Dec-21
C19IMPACT_3350	Trinidad and Tobago	18-Nov-21	MoH	N	N	N	N	N	N	16.00		06-Dec-21	07-Dec-21	10-Dec-21	13-Dec-21
C19IMPACT_3351	Trinidad and Tobago	18-Nov-21	MoH	N	N	N	N	N	N	16.00		06-Dec-21	07-Dec-21	10-Dec-21	13-Dec-21
C19IMPACT_3352	Trinidad and Tobago	18-Nov-21	MoH	N	N	N	N	N	N	16.00		06-Dec-21	07-Dec-21	10-Dec-21	13-Dec-21
C19IMPACT_3353	Trinidad and Tobago	18-Nov-21	MoH	N	N	N	N	N	N	16.00		06-Dec-21	07-Dec-21	10-Dec-21	13-Dec-21
C19IMPACT_3354	Trinidad and Tobago	18-Nov-21	MoH	N	N	N	N	N	N	16.00		06-Dec-21	07-Dec-21	10-Dec-21	
C19IMPACT_3355	Trinidad and Tobago	18-Nov-21	MoH	N	N	N	N	N	N	15.00		06-Dec-21	07-Dec-21	10-Dec-21	13-Dec-21
C19IMPACT_3356	Trinidad and Tobago	19-Nov-21	MoH	N	N	N	N	N	Y	18.80		02-Dec-21	07-Dec-21	10-Dec-21	13-Dec-21
C19IMPACT_3357	Trinidad and Tobago	19-Nov-21	MoH	N	N	N	N	N	Y	15.00		02-Dec-21	07-Dec-21	10-Dec-21	13-Dec-21
C19IMPACT_3358	Trinidad and Tobago	19-Nov-21	MoH	N	N	N	N	N	Y	18.70		02-Dec-21	07-Dec-21	10-Dec-21	13-Dec-21
C19IMPACT_3359	Trinidad and Tobago	19-Nov-21	MoH	N	N	N	N	N	N	19.00		06-Dec-21	07-Dec-21	10-Dec-21	13-Dec-21
C19IMPACT_3360	Trinidad and Tobago	20-Nov-21	MoH	N	N	N	N	N	N	15.00		06-Dec-21	07-Dec-21	10-Dec-21	13-Dec-21
C19IMPACT_3361	Trinidad and Tobago	20-Nov-21	MoH	N	N	N	N	N	N	18.00		06-Dec-21	07-Dec-21	10-Dec-21	13-Dec-21
C19IMPACT_3362	Trinidad and Tobago	23-Nov-21	MoH	N	N	N	N	N	Y	19.00		02-Dec-21	07-Dec-21	10-Dec-21	13-Dec-21
C19IMPACT_3363	Trinidad and Tobago	24-Nov-21	MoH	N	N	N	N	N	N	14.00		06-Dec-21	07-Dec-21	10-Dec-21	13-Dec-21
C19IMPACT_3364	Trinidad and Tobago	29-Nov-21	MoH	N	N	N	N	N	N		8.00	06-Dec-21	07-Dec-21	10-Dec-21	13-Dec-21
C19IMPACT_3365	Trinidad and Tobago	29-Nov-21	MoH	N	N	N	N	N	N	16.00		06-Dec-21	07-Dec-21	10-Dec-21	13-Dec-21
C19IMPACT_3366	Trinidad and Tobago	29-Nov-21	MoH	N	N	N	N	N	N	15.00		06-Dec-21	07-Dec-21	10-Dec-21	13-Dec-21
C19IMPACT_3367	Trinidad and Tobago	29-Nov-21	MoH	N	N	N	N	N	N		3.00	06-Dec-21	07-Dec-21	10-Dec-21	13-Dec-21
C19IMPACT_3368	Trinidad and Tobago	30-Nov-21	MoH	N	N	N	N	N	N	15.00		06-Dec-21	07-Dec-21	10-Dec-21	13-Dec-21
C19IMPACT_3369	Trinidad and Tobago	30-Nov-21	MoH	N	N	N	N	N	N	14.00		06-Dec-21	07-Dec-21	10-Dec-21	13-Dec-21
C19IMPACT_3370	Trinidad and Tobago	01-Dec-21	MoH	N	N	N	N	N	N	11.00		06-Dec-21	07-Dec-21	10-Dec-21	13-Dec-21
C19IMPACT_3371	Trinidad and Tobago	01-Dec-21	MoH	N	N	N	N	N	N	12.00		06-Dec-21	07-Dec-21	10-Dec-21	13-Dec-21
C19IMPACT_3372	Trinidad and Tobago	01-Dec-21	MoH	N	N	N	N	N	N	17.00		06-Dec-21	07-Dec-21	10-Dec-21	13-Dec-21
C19IMPACT_3373	Trinidad and Tobago	01-Dec-21	MoH	N	N	N	N	N	N	16.00		06-Dec-21	07-Dec-21	10-Dec-21	13-Dec-21
C19IMPACT_3374	Trinidad and Tobago	22-Nov-21	MoH	N	N	N	N	N	N	16.00		06-Dec-21	09-Dec-21	10-Dec-21	13-Dec-21
C19IMPACT_3375	Trinidad and Tobago	23-Nov-21	MoH	N	N	N	N	N	N	17.00		06-Dec-21	09-Dec-21	10-Dec-21	13-Dec-21
C19IMPACT_3376	Trinidad and Tobago	23-Nov-21	MoH	N	N	N	N	N	N	11.00		06-Dec-21	09-Dec-21	10-Dec-21	13-Dec-21
C19IMPACT_3377	Trinidad and Tobago	23-Nov-21	MoH	N	N	N	N	N	N	14.00		06-Dec-21	09-Dec-21	10-Dec-21	13-Dec-21
C19IMPACT_3378	Trinidad and Tobago	23-Nov-21	MoH	N	N	N	N	N	N	14.00		06-Dec-21	09-Dec-21	10-Dec-21	13-Dec-21
C19IMPACT_3379	Trinidad and Tobago	24-Nov-21	MoH	N	N	N	N	N	N	15.00		06-Dec-21	09-Dec-21	10-Dec-21	13-Dec-21
C19IMPACT_3380	Trinidad and Tobago	24-Nov-21	MoH	N	N	N	N	N	N	14.00		06-Dec-21	09-Dec-21	10-Dec-21	13-Dec-21
C19IMPACT_3381	Trinidad and Tobago	24-Nov-21	MoH	N	N	N	N	N	N	13.00		06-Dec-21	09-Dec-21	10-Dec-21	13-Dec-21
C19IMPACT_3382	Trinidad and Tobago	24-Nov-21	MoH	N	N	N	N	N	N	14.00		06-Dec-21	09-Dec-21	10-Dec-21	13-Dec-21
C19IMPACT_3383	Trinidad and Tobago	24-Nov-21	MoH	N	N	N	N	N	N	15.00		06-Dec-21	09-Dec-21	10-Dec-21	13-Dec-21
C19IMPACT_3384	Trinidad and Tobago	25-Nov-21	MoH	N	N	N	N	N	N	16.00		06-Dec-21	09-Dec-21	10-Dec-21	13-Dec-21
C19IMPACT_3385	Trinidad and Tobago	25-Nov-21	MoH	N	N	N	N	N	N	19.00		06-Dec-21	09-Dec-21	10-Dec-21	13-Dec-21
C19IMPACT_3386	Trinidad and Tobago	26-Nov-21	MoH	N	N	N	N	N	N	18.00		06-Dec-21	09-Dec-21	10-Dec-21	13-Dec-21
C19IMPACT_3387	Trinidad and Tobago	26-Nov-21	MoH	N	N	N	N	N	N	14.00		06-Dec-21	09-Dec-21	10-Dec-21	13-Dec-21
C19IMPACT_3388	Trinidad and Tobago	26-Nov-21	MoH	N	N	N	N	N	N	13.00		06-Dec-21	09-Dec-21	10-Dec-21	13-Dec-21

C19IMPACT_3389	Trinidad and Tobago	26-Nov-21	MoH	N	N	N	N	N	N	12.00		06-Dec-21	09-Dec-21	10-Dec-21		13-Dec-21
C19IMPACT_3390	Trinidad and Tobago	26-Nov-21	MoH	N	N	N	N	N	Y	14.00		06-Dec-21	09-Dec-21	10-Dec-21		13-Dec-21
C19IMPACT_3391	Trinidad and Tobago	27-Nov-21	MoH	N	N	N	N	N	N	16.00		06-Dec-21	09-Dec-21	10-Dec-21		13-Dec-21
C19IMPACT_3392	Trinidad and Tobago	27-Nov-21	MoH	N	N	N	N	N	N	14.00		06-Dec-21	09-Dec-21	10-Dec-21		13-Dec-21
C19IMPACT_3393	Trinidad and Tobago	28-Nov-21	MoH	N	N	N	N	N	N	17.00		06-Dec-21	09-Dec-21	10-Dec-21		13-Dec-21
C19IMPACT_3394	Trinidad and Tobago	29-Nov-21	MoH	N	N	N	N	N	N	15.00		06-Dec-21	09-Dec-21	10-Dec-21		13-Dec-21
C19IMPACT_3395	Trinidad and Tobago	29-Nov-21	MoH	N	N	N	N	N	N	15.00		06-Dec-21	09-Dec-21	10-Dec-21		13-Dec-21
C19IMPACT_3396	Trinidad and Tobago	10-Dec-21	MoH	N	N	N	N	N	Y	22.05		11-Dec-21	12-Dec-21	17-Dec-21		13-Dec-21
C19IMPACT_3397	Trinidad and Tobago	10-Dec-21	MoH	N	N	N	N	N	N	23.00		11-Dec-21	12-Dec-21	17-Dec-21		13-Dec-21
C19IMPACT_3398	Trinidad and Tobago	30-Nov-21	MoH	N	N	N	N	N	N	15.00		13-Dec-21	15-Dec-21	17-Dec-21		17-Dec-21
C19IMPACT_3399	Trinidad and Tobago	30-Nov-21	MoH	N	N	N	N	N	N	15.00		13-Dec-21	15-Dec-21	17-Dec-21		17-Dec-21
C19IMPACT_3400	Trinidad and Tobago	30-Nov-21	MoH	N	N	N	N	N	N	15.00		13-Dec-21	15-Dec-21	17-Dec-21		17-Dec-21
C19IMPACT_3401	Trinidad and Tobago	30-Nov-21	MoH	N	N	N	N	N	N	16.00		13-Dec-21	15-Dec-21	17-Dec-21		17-Dec-21
C19IMPACT_3402	Trinidad and Tobago	01-Dec-21	MoH	N	N	N	N	N	N	15.00		13-Dec-21	15-Dec-21	17-Dec-21		17-Dec-21
C19IMPACT_3403	Trinidad and Tobago	01-Dec-21	MoH	N	N	N	N	N	N	14.00		13-Dec-21	15-Dec-21	17-Dec-21		17-Dec-21
C19IMPACT_3404	Trinidad and Tobago	01-Dec-21	MoH	N	N	N	N	N	N	16.00		13-Dec-21	15-Dec-21	17-Dec-21		17-Dec-21
C19IMPACT_3405	Trinidad and Tobago	02-Dec-21	MoH	N	N	N	N	N	N	16.00		13-Dec-21	15-Dec-21	17-Dec-21		17-Dec-21
C19IMPACT_3406	Trinidad and Tobago	02-Dec-21	MoH	N	N	N	N	N	N	15.00		13-Dec-21	15-Dec-21	17-Dec-21		17-Dec-21
C19IMPACT_3407	Trinidad and Tobago	02-Dec-21	MoH	N	N	N	N	N	N	14.00		13-Dec-21	15-Dec-21	17-Dec-21		17-Dec-21
C19IMPACT_3408	Trinidad and Tobago	02-Dec-21	MoH	N	N	N	N	N	N	15.00		13-Dec-21	15-Dec-21	17-Dec-21		17-Dec-21
C19IMPACT_3409	Trinidad and Tobago	03-Dec-21	MoH	N	N	N	N	N	N	15.00		13-Dec-21	15-Dec-21	17-Dec-21		17-Dec-21
C19IMPACT_3410	Trinidad and Tobago	03-Dec-21	MoH	N	N	N	N	N	N	18.00		13-Dec-21	15-Dec-21	17-Dec-21		17-Dec-21
C19IMPACT_3411	Trinidad and Tobago	05-Dec-21	MoH	N	N	N	N	N	N	15.00		13-Dec-21	15-Dec-21	17-Dec-21		17-Dec-21
C19IMPACT_3412	Trinidad and Tobago	05-Dec-21	MoH	N	N	N	N	N	N	16.00		13-Dec-21	15-Dec-21	17-Dec-21		17-Dec-21
C19IMPACT_3413	Trinidad and Tobago	05-Dec-21	MoH	N	N	N	N	N	N	16.00		13-Dec-21	15-Dec-21	17-Dec-21		17-Dec-21
C19IMPACT_3414	Trinidad and Tobago	05-Dec-21	MoH	N	N	N	N	N	N	17.00		13-Dec-21	15-Dec-21	17-Dec-21		17-Dec-21
C19IMPACT_3415	Trinidad and Tobago	05-Dec-21	MoH	N	N	N	N	N	N	18.00		13-Dec-21	15-Dec-21	17-Dec-21		17-Dec-21
C19IMPACT_3416	Trinidad and Tobago	05-Dec-21	MoH	N	N	N	N	N	N	15.00		13-Dec-21	15-Dec-21	17-Dec-21		17-Dec-21
C19IMPACT_3417	Trinidad and Tobago	05-Dec-21	MoH	N	N	N	N	N	N	17.00		13-Dec-21	15-Dec-21	17-Dec-21		17-Dec-21
C19IMPACT_3418	Trinidad and Tobago	06-Dec-21	MoH	N	N	N	N	N	N	18.00		13-Dec-21	15-Dec-21	17-Dec-21		17-Dec-21
C19IMPACT_3419	Trinidad and Tobago	06-Dec-21	MoH	N	N	N	N	N	N	15.00		13-Dec-21	15-Dec-21	17-Dec-21		17-Dec-21
C19IMPACT_3420	Trinidad and Tobago	26-Nov-21	MoH	N	N	N	N	N	N	16.00		06-Dec-21	17-Dec-21	24-Dec-21		17-Dec-21
C19IMPACT_3421	Trinidad and Tobago	26-Nov-21	MoH	N	N	N	N	N	N	15.00		06-Dec-21	17-Dec-21	24-Dec-21		17-Dec-21
C19IMPACT_3422	Trinidad and Tobago	26-Nov-21	MoH	N	N	N	N	N	N	18.00		06-Dec-21	17-Dec-21	24-Dec-21		17-Dec-21
C19IMPACT_3423	Trinidad and Tobago	28-Nov-21	MoH	N	N	N	N	N	N	16.00		06-Dec-21	17-Dec-21	24-Dec-21		17-Dec-21
C19IMPACT_3424	Trinidad and Tobago	28-Nov-21	MoH	N	N	N	N	N	N	17.00		06-Dec-21	17-Dec-21	24-Dec-21		17-Dec-21
C19IMPACT_3425	Trinidad and Tobago	29-Nov-21	MoH	N	N	N	N	N	N	16.00		06-Dec-21	17-Dec-21	24-Dec-21		17-Dec-21
C19IMPACT_3426	Trinidad and Tobago	29-Nov-21	MoH	N	N	N	N	N	N	18.00		06-Dec-21	17-Dec-21	24-Dec-21		17-Dec-21
C19IMPACT_3427	Trinidad and Tobago	29-Nov-21	MoH	N	N	N	N	N	N	13.00		06-Dec-21	17-Dec-21	24-Dec-21		
C19IMPACT_3428	Trinidad and Tobago	01-Dec-21	MoH	N	N	N	N	N	N	16.00		06-Dec-21	17-Dec-21	24-Dec-21		17-Dec-21
C19IMPACT_3429	Trinidad and Tobago	02-Dec-21	MoH	N	N	N	N	N	N	18.00		13-Dec-21	17-Dec-21	24-Dec-21		17-Dec-21
C19IMPACT_3430	Trinidad and Tobago	03-Dec-21	MoH	N	N	N	N	N	N	17.00		13-Dec-21	17-Dec-21	24-Dec-21		17-Dec-21
C19IMPACT_3431	Trinidad and Tobago	03-Dec-21	MoH	N	N	N	N	N	N	20.00		13-Dec-21	17-Dec-21	24-Dec-21		17-Dec-21
C19IMPACT_3432	Trinidad and Tobago	04-Dec-21	MoH	N	N	N	N	N	N	16.00		13-Dec-21	17-Dec-21	24-Dec-21		17-Dec-21
C19IMPACT_3433	Trinidad and Tobago	06-Dec-21	MoH	N	N	N	N	N	N	16.00		13-Dec-21	17-Dec-21	24-Dec-21		17-Dec-21
C19IMPACT_3434	Trinidad and Tobago	06-Dec-21	MoH	N	N	N	N	N	N	16.00		13-Dec-21	17-Dec-21	24-Dec-21		17-Dec-21
C19IMPACT_3435	Trinidad and Tobago	06-Dec-21	MoH	N	N	N	N	N	N	16.00		13-Dec-21	17-Dec-21	24-Dec-21		
C19IMPACT_3436	Trinidad and Tobago	11-Dec-21	MoH	N	N	N	N	N	Y	18.00		16-Dec-21	17-Dec-21	24-Dec-21		17-Dec-21
C19IMPACT_3437	Trinidad and Tobago	13-Dec-21	MoH	N	N	N	N	N	Y	17.00		16-Dec-21	17-Dec-21	24-Dec-21		17-Dec-21
C19IMPACT_3438	Trinidad and Tobago	13-Dec-21	MoH	N	N	N	N	N	Y	21.00		16-Dec-21	17-Dec-21	24-Dec-21		17-Dec-21
C19IMPACT_3439	Trinidad and Tobago	15-Dec-21	MoH	N	N	N	N	N	Y	20.00		16-Dec-21	17-Dec-21	24-Dec-21		17-Dec-21
C19IMPACT_3440	Trinidad and Tobago	16-Dec-21	MoH	N	N	N	N	N	Y	18.00		16-Dec-21	17-Dec-21	24-Dec-21		17-Dec-21
C19IMPACT_3441	Trinidad and Tobago	16-Dec-21	MoH	N	N	N	N	N	Y	16.00		16-Dec-21	17-Dec-21	24-Dec-21		17-Dec-21
C19IMPACT_3442	Trinidad and Tobago	26-Nov-21	MoH	N	N	N	N	N	N	16.00		06-Dec-21	18-Dec-21	24-Dec-21		31-Dec-21
C19IMPACT_3443	Trinidad and Tobago	29-Nov-21	MoH	N	N	N	N	N	N	15.00		13-Dec-21	18-Dec-21	24-Dec-21		31-Dec-21
C19IMPACT_3444	Trinidad and Tobago	30-Nov-21	MoH	N	N	N	N	N	N		11.00	06-Dec-21	18-Dec-21	24-Dec-21		31-Dec-21
C19IMPACT_3445	Trinidad and Tobago	30-Nov-21	CARPHA	N	N	N	N	N	Y	18.75		07-Dec-21	18-Dec-21	24-Dec-21	21-Dec-21	31-Dec-21
C19IMPACT_3446	Trinidad and Tobago	30-Nov-21	MoH	N	N	N	N	N	N	15.00		13-Dec-21	18-Dec-21	24-Dec-21		31-Dec-21
C19IMPACT_3447	Trinidad and Tobago	30-Nov-21	MoH	N	N	N	N	N	N	15.00		13-Dec-21	18-Dec-21	24-Dec-21		31-Dec-21
C19IMPACT_3448	Trinidad and Tobago	30-Nov-21	MoH	N	N	N	N	N	N	14.00		13-Dec-21	18-Dec-21	24-Dec-21		31-Dec-21
C19IMPACT_3449	Trinidad and Tobago	30-Nov-21	MoH	N	N	N	N	N	N	16.00		13-Dec-21	18-Dec-21	24-Dec-21		31-Dec-21
C19IMPACT_3450	Trinidad and Tobago	01-Dec-21	MoH	N	N	N	N	N	N	15.00		13-Dec-21	18-Dec-21	24-Dec-21		
C19IMPACT_3451	Trinidad and Tobago	01-Dec-21	MoH	N	N	N	N	N	N	15.00		13-Dec-21	18-Dec-21	24-Dec-21		31-Dec-21
C19IMPACT_3452	Trinidad and Tobago	03-Dec-21	MoH	N	N	N	N	N	N	16.00		13-Dec-21	18-Dec-21	24-Dec-21		31-Dec-21

C19IMPACT_3453	Grenada	03-Dec-21	CARPHA	N	N	N	N	N	N	28.60		17-Dec-21	18-Dec-21		21-Dec-21	
C19IMPACT_3454	Trinidad and Tobago	04-Dec-21	CARPHA	N	N	N	N	N	Y	16.62		07-Dec-21	18-Dec-21	24-Dec-21	21-Dec-21	31-Dec-21
C19IMPACT_3455	Trinidad and Tobago	05-Dec-21	MoH	N	N	N	N	N	N	16.00		13-Dec-21	18-Dec-21	24-Dec-21		31-Dec-21
C19IMPACT_3456	Trinidad and Tobago	06-Dec-21	CARPHA	N	N	N	N	N	N	19.55		17-Dec-21	18-Dec-21	24-Dec-21	21-Dec-21	31-Dec-21
C19IMPACT_3457	Trinidad and Tobago	06-Dec-21	CARPHA	N	N	N	N	N	N	17.40		17-Dec-21	18-Dec-21	24-Dec-21	21-Dec-21	31-Dec-21
C19IMPACT_3458	Trinidad and Tobago	07-Dec-21	CARPHA	N	N	N	N	N	Y	14.94		17-Dec-21	18-Dec-21	24-Dec-21	21-Dec-21	31-Dec-21
C19IMPACT_3459	Trinidad and Tobago	08-Dec-21	CARPHA	N	N	N	N	N	Y	23.18		17-Dec-21	18-Dec-21	24-Dec-21	21-Dec-21	31-Dec-21
C19IMPACT_3460	Trinidad and Tobago	16-Dec-21	MoH	N	N	N	N	N	Y	16.94		18-Dec-21	18-Dec-21	24-Dec-21		31-Dec-21
C19IMPACT_3461	Trinidad and Tobago		MoH	N	N	N	N	N	Y			17-Dec-21	18-Dec-21	24-Dec-21		
C19IMPACT_3462	Trinidad and Tobago		CARPHA	N	N	N	N	N	Y	14.07		17-Dec-21	18-Dec-21	24-Dec-21	21-Dec-21	
C19IMPACT_3463	Trinidad and Tobago	16-Nov-21	MoH	N	N	N	N	N	Y		17.00	21-Dec-21	23-Dec-21	24-Dec-21		
C19IMPACT_3464	Trinidad and Tobago	16-Nov-21	MoH	N	N	N	N	N	Y		5.00	21-Dec-21	23-Dec-21	24-Dec-21		31-Dec-21
C19IMPACT_3465	Trinidad and Tobago	17-Nov-21	MoH	N	N	N	N	N	Y		5.00	21-Dec-21	23-Dec-21	24-Dec-21		31-Dec-21
C19IMPACT_3466	Trinidad and Tobago	17-Nov-21	MoH	N	N	N	N	N	Y		6.00	21-Dec-21	23-Dec-21	24-Dec-21		31-Dec-21
C19IMPACT_3467	Trinidad and Tobago	17-Nov-21	MoH	N	N	N	N	N	Y		3.00	21-Dec-21	23-Dec-21	24-Dec-21		31-Dec-21
C19IMPACT_3468	Trinidad and Tobago	17-Nov-21	MoH	N	N	N	N	N	Y		5.00	21-Dec-21	23-Dec-21	24-Dec-21		
C19IMPACT_3469	Trinidad and Tobago	18-Nov-21	MoH	N	N	N	N	N	Y		6.00	21-Dec-21	23-Dec-21	24-Dec-21		
C19IMPACT_3470	Trinidad and Tobago	18-Nov-21	MoH	N	N	N	N	N	Y		8.00	21-Dec-21	23-Dec-21	24-Dec-21		
C19IMPACT_3471	Trinidad and Tobago	18-Nov-21	MoH	N	N	N	N	N	Y		4.00	21-Dec-21	23-Dec-21	24-Dec-21		
C19IMPACT_3472	Trinidad and Tobago	19-Nov-21	MoH	N	N	N	N	N	Y		11.00	21-Dec-21	23-Dec-21	24-Dec-21		
C19IMPACT_3473	Trinidad and Tobago	19-Nov-21	MoH	N	N	N	N	N	Y		6.00	21-Dec-21	23-Dec-21	24-Dec-21		
C19IMPACT_3474	Trinidad and Tobago	20-Nov-21	MoH	N	N	N	N	N	Y		9.00	21-Dec-21	23-Dec-21	24-Dec-21		
C19IMPACT_3475	Trinidad and Tobago	20-Nov-21	MoH	N	N	N	N	N	Y		6.00	21-Dec-21	23-Dec-21	24-Dec-21		31-Dec-21
C19IMPACT_3476	Trinidad and Tobago	20-Nov-21	MoH	N	N	N	N	N	Y		8.00	21-Dec-21	23-Dec-21	24-Dec-21		31-Dec-21
C19IMPACT_3477	Trinidad and Tobago	20-Nov-21	MoH	N	N	N	N	N	Y		16.00	21-Dec-21	23-Dec-21	24-Dec-21		
C19IMPACT_3478	Trinidad and Tobago	21-Nov-21	MoH	N	N	N	N	N	Y		14.00	21-Dec-21	23-Dec-21	24-Dec-21		31-Dec-21
C19IMPACT_3479	Trinidad and Tobago	22-Nov-21	MoH	N	N	N	N	N	Y		11.00	21-Dec-21	23-Dec-21	24-Dec-21		
C19IMPACT_3480	Trinidad and Tobago	22-Nov-21	MoH	N	N	N	N	N	Y		5.00	21-Dec-21	23-Dec-21	24-Dec-21		
C19IMPACT_3481	Trinidad and Tobago	22-Nov-21	MoH	N	N	N	N	N	Y		14.00	21-Dec-21	23-Dec-21	24-Dec-21		31-Dec-21
C19IMPACT_3482	Trinidad and Tobago	22-Nov-21	MoH	N	N	N	N	N	Y		7.00	21-Dec-21	23-Dec-21	24-Dec-21		31-Dec-21
C19IMPACT_3483	Trinidad and Tobago	22-Nov-21	MoH	N	N	N	N	N	Y		17.00	21-Dec-21	23-Dec-21	24-Dec-21		31-Dec-21
C19IMPACT_3484	Trinidad and Tobago	22-Nov-21	MoH	N	N	N	N	N	Y		9.00	21-Dec-21	23-Dec-21	24-Dec-21		31-Dec-21
C19IMPACT_3485	Trinidad and Tobago	23-Nov-21	MoH	N	N	N	N	N	Y		8.00	21-Dec-21	23-Dec-21	24-Dec-21		31-Dec-21
C19IMPACT_3486	Trinidad and Tobago	23-Nov-21	MoH	N	N	N	N	N	Y		5.00	21-Dec-21	23-Dec-21	24-Dec-21		31-Dec-21
C19IMPACT_3487	Trinidad and Tobago	23-Nov-21	MoH	N	N	N	N	N	Y		9.00	21-Dec-21	23-Dec-21	24-Dec-21		31-Dec-21
C19IMPACT_3488	Trinidad and Tobago	23-Nov-21	MoH	N	N	N	N	N	Y		7.00	21-Dec-21	23-Dec-21	24-Dec-21		31-Dec-21
C19IMPACT_3489	Trinidad and Tobago	23-Nov-21	MoH	N	N	N	N	N	Y		3.00	21-Dec-21	23-Dec-21	24-Dec-21		31-Dec-21
C19IMPACT_3490	Trinidad and Tobago	23-Nov-21	MoH	N	N	N	N	N	Y		4.00	21-Dec-21	23-Dec-21	24-Dec-21		
C19IMPACT_3491	Trinidad and Tobago	29-Nov-21	MoH	N	N	N	N	N	Y		12.00	21-Dec-21	23-Dec-21	24-Dec-21		31-Dec-21
C19IMPACT_3492	Trinidad and Tobago	30-Nov-21	MoH	N	N	N	N	N	Y		3.00	21-Dec-21	23-Dec-21	24-Dec-21		
C19IMPACT_3493	Trinidad and Tobago	30-Nov-21	MoH	N	N	N	N	N	Y		4.00	21-Dec-21	23-Dec-21	24-Dec-21		31-Dec-21
C19IMPACT_3494	Trinidad and Tobago	30-Nov-21	MoH	N	N	N	N	N	Y		12.00	21-Dec-21	23-Dec-21	24-Dec-21		31-Dec-21
C19IMPACT_3495	Trinidad and Tobago	30-Nov-21	MoH	N	N	N	N	N	Y		4.00	21-Dec-21	23-Dec-21	24-Dec-21		31-Dec-21
C19IMPACT_3496	Trinidad and Tobago	30-Nov-21	MoH	N	N	N	N	N	Y		8.00	21-Dec-21	23-Dec-21	24-Dec-21		31-Dec-21
C19IMPACT_3497	Trinidad and Tobago	30-Nov-21	MoH	N	N	N	N	N	Y		9.00	21-Dec-21	23-Dec-21	24-Dec-21		31-Dec-21
C19IMPACT_3498	Trinidad and Tobago	01-Dec-21	MoH	N	N	N	N	N	Y		5.00	21-Dec-21	23-Dec-21	24-Dec-21		31-Dec-21
C19IMPACT_3499	Trinidad and Tobago	01-Dec-21	MoH	N	N	N	N	N	Y		10.00	21-Dec-21	23-Dec-21	24-Dec-21		31-Dec-21
C19IMPACT_3500	Trinidad and Tobago	03-Dec-21	MoH	N	N	N	N	N	Y	16.00		21-Dec-21	23-Dec-21	24-Dec-21		31-Dec-21
C19IMPACT_3501	Trinidad and Tobago	04-Dec-21	MoH	N	N	N	N	N	Y	14.00		21-Dec-21	23-Dec-21	24-Dec-21		31-Dec-21
C19IMPACT_3502	Trinidad and Tobago	04-Dec-21	MoH	N	N	N	N	N	Y	16.00		21-Dec-21	23-Dec-21	24-Dec-21		31-Dec-21
C19IMPACT_3503	Trinidad and Tobago	04-Dec-21	MoH	N	N	N	N	N	Y	18.00		21-Dec-21	23-Dec-21	24-Dec-21		31-Dec-21
C19IMPACT_3504	Trinidad and Tobago	04-Dec-21	MoH	N	N	N	N	N	Y	19.00		21-Dec-21	23-Dec-21	24-Dec-21		31-Dec-21
C19IMPACT_3505	Trinidad and Tobago	06-Dec-21	MoH	N	N	N	N	N	Y	11.80		21-Dec-21	23-Dec-21	24-Dec-21		31-Dec-21
C19IMPACT_3506	Trinidad and Tobago	06-Dec-21	MoH	N	N	N	N	N	Y	10.20		21-Dec-21	23-Dec-21	24-Dec-21		31-Dec-21
C19IMPACT_3507	Trinidad and Tobago	06-Dec-21	MoH	N	N	N	N	N	Y	13.10		21-Dec-21	23-Dec-21	24-Dec-21		31-Dec-21
C19IMPACT_3508	Trinidad and Tobago	06-Dec-21	MoH	N	N	N	N	N	Y	7.80		21-Dec-21	23-Dec-21	24-Dec-21		31-Dec-21
C19IMPACT_3509	Trinidad and Tobago	06-Dec-21	MoH	N	N	N	N	N	Y	16.00		21-Dec-21	23-Dec-21	24-Dec-21		31-Dec-21
C19IMPACT_3510	Trinidad and Tobago	06-Dec-21	MoH	N	N	N	N	N	Y	16.00		21-Dec-21	23-Dec-21	24-Dec-21		31-Dec-21
C19IMPACT_3511	Trinidad and Tobago	06-Dec-21	MoH	N	N	N	N	N	Y	16.00		21-Dec-21	23-Dec-21	24-Dec-21		31-Dec-21
C19IMPACT_3512	Trinidad and Tobago	06-Dec-21	MoH	N	N	N	N	N	Y	15.00		21-Dec-21	23-Dec-21	24-Dec-21		31-Dec-21
C19IMPACT_3513	Trinidad and Tobago	06-Dec-21	MoH	N	N	N	N	N	Y	18.00		21-Dec-21	23-Dec-21	24-Dec-21		31-Dec-21
C19IMPACT_3514	Trinidad and Tobago	06-Dec-21	MoH	N	N	N	N	N	Y	20.00		21-Dec-21	23-Dec-21	24-Dec-21		
C19IMPACT_3515	Trinidad and Tobago	06-Dec-21	MoH	N	N	N	N	N	Y	15.00		21-Dec-21	23-Dec-21	24-Dec-21		31-Dec-21
C19IMPACT_3516	Trinidad and Tobago	06-Dec-21	MoH	N	N	N	N	N	Y	14.00		21-Dec-21	23-Dec-21	24-Dec-21		31-Dec-21

C19IMPACT_3517	Trinidad and Tobago	06-Dec-21	MoH	N	N	N	N	N	Y	18.00		21-Dec-21	23-Dec-21	24-Dec-21		31-Dec-21
C19IMPACT_3518	Trinidad and Tobago	06-Dec-21	MoH	N	N	N	N	N	Y	18.00		21-Dec-21	23-Dec-21	24-Dec-21		31-Dec-21
C19IMPACT_3519	Trinidad and Tobago	06-Dec-21	MoH	N	N	N	N	N	Y	15.00		21-Dec-21	23-Dec-21	24-Dec-21		31-Dec-21
C19IMPACT_3520	Trinidad and Tobago	06-Dec-21	MoH	N	N	N	N	N	Y	15.00		21-Dec-21	23-Dec-21	24-Dec-21		31-Dec-21
C19IMPACT_3521	Trinidad and Tobago	06-Dec-21	MoH	N	N	N	N	N	Y	15.00		21-Dec-21	23-Dec-21	24-Dec-21		
C19IMPACT_3522	Trinidad and Tobago	07-Dec-21	MoH	N	N	N	N	N	Y	8.50		21-Dec-21	23-Dec-21	24-Dec-21		31-Dec-21
C19IMPACT_3523	Trinidad and Tobago	07-Dec-21	MoH	N	N	N	N	N	Y	11.10		21-Dec-21	23-Dec-21	24-Dec-21		31-Dec-21
C19IMPACT_3524	Trinidad and Tobago	07-Dec-21	MoH	N	N	N	N	N	Y	18.00		21-Dec-21	23-Dec-21	24-Dec-21		31-Dec-21
C19IMPACT_3525	Trinidad and Tobago	07-Dec-21	MoH	N	N	N	N	N	Y	18.00		21-Dec-21	23-Dec-21	24-Dec-21		31-Dec-21
C19IMPACT_3526	Trinidad and Tobago	07-Dec-21	MoH	N	N	N	N	N	Y	18.00		21-Dec-21	23-Dec-21	24-Dec-21		31-Dec-21
C19IMPACT_3527	Trinidad and Tobago	07-Dec-21	MoH	N	N	N	N	N	Y	14.00		21-Dec-21	23-Dec-21	24-Dec-21		31-Dec-21
C19IMPACT_3528	Trinidad and Tobago	07-Dec-21	MoH	N	N	N	N	N	Y	15.00		21-Dec-21	23-Dec-21	24-Dec-21		31-Dec-21
C19IMPACT_3529	Trinidad and Tobago	07-Dec-21	MoH	N	N	N	N	N	Y	15.00		21-Dec-21	23-Dec-21	24-Dec-21		31-Dec-21
C19IMPACT_3530	Trinidad and Tobago	07-Dec-21	MoH	N	N	N	N	N	Y	18.00		21-Dec-21	23-Dec-21	24-Dec-21		31-Dec-21
C19IMPACT_3531	Trinidad and Tobago	07-Dec-21	MoH	N	N	N	N	N	Y	15.00		21-Dec-21	23-Dec-21	24-Dec-21		31-Dec-21
C19IMPACT_3532	Trinidad and Tobago	08-Dec-21	MoH	N	N	N	N	N	Y	15.00		21-Dec-21	23-Dec-21	24-Dec-21		31-Dec-21
C19IMPACT_3533	Trinidad and Tobago	08-Dec-21	MoH	N	N	N	N	N	Y	18.00		21-Dec-21	23-Dec-21	24-Dec-21		31-Dec-21
C19IMPACT_3534	Trinidad and Tobago	08-Dec-21	MoH	N	N	N	N	N	Y	16.00		21-Dec-21	23-Dec-21	24-Dec-21		31-Dec-21
C19IMPACT_3535	Trinidad and Tobago	09-Dec-21	MoH	N	N	N	N	N	Y			21-Dec-21	23-Dec-21	24-Dec-21		31-Dec-21
C19IMPACT_3536	Trinidad and Tobago	09-Dec-21	MoH	N	N	N	N	N	Y	12.30		21-Dec-21	23-Dec-21	24-Dec-21		31-Dec-21
C19IMPACT_3537	Trinidad and Tobago	09-Dec-21	MoH	N	N	N	N	N	Y	11.40		21-Dec-21	23-Dec-21	24-Dec-21		31-Dec-21
C19IMPACT_3538	Trinidad and Tobago	09-Dec-21	MoH	N	N	N	N	N	Y		10.00	21-Dec-21	23-Dec-21	24-Dec-21		31-Dec-21
C19IMPACT_3539	Trinidad and Tobago	09-Dec-21	MoH	N	N	N	N	N	Y		8.00	21-Dec-21	23-Dec-21	24-Dec-21		
C19IMPACT_3540	Trinidad and Tobago	09-Dec-21	MoH	N	N	N	N	N	Y		3.00	21-Dec-21	23-Dec-21	24-Dec-21		31-Dec-21
C19IMPACT_3541	Trinidad and Tobago	10-Dec-21	MoH	N	N	N	N	N	Y	13.00		21-Dec-21	23-Dec-21	24-Dec-21		31-Dec-21
C19IMPACT_3542	Trinidad and Tobago	10-Dec-21	MoH	N	N	N	N	N	Y	17.00		21-Dec-21	23-Dec-21	24-Dec-21		31-Dec-21
C19IMPACT_3543	Trinidad and Tobago	11-Dec-21	MoH	N	N	N	N	N	Y		7.03	21-Dec-21	23-Dec-21	24-Dec-21		31-Dec-21
C19IMPACT_3544	Trinidad and Tobago	14-Dec-21	MoH	N	N	N	N	N	Y		4.09	21-Dec-21	23-Dec-21	24-Dec-21		31-Dec-21
C19IMPACT_3545	Trinidad and Tobago	14-Dec-21	MoH	N	N	N	N	N	Y		6.90	21-Dec-21	23-Dec-21	24-Dec-21		31-Dec-21
C19IMPACT_3546	Trinidad and Tobago	14-Dec-21	MoH	N	N	N	N	N	Y		4.95	21-Dec-21	23-Dec-21	24-Dec-21		31-Dec-21
C19IMPACT_3547	Trinidad and Tobago	15-Dec-21	MoH	N	N	N	N	N	Y		3.66	21-Dec-21	23-Dec-21	24-Dec-21		31-Dec-21
C19IMPACT_3548	Trinidad and Tobago	15-Dec-21	MoH	N	N	N	N	N	Y		6.66	21-Dec-21	23-Dec-21	24-Dec-21		31-Dec-21
C19IMPACT_3549	Trinidad and Tobago	16-Dec-21	MoH	N	N	N	N	N	Y		6.58	21-Dec-21	23-Dec-21	24-Dec-21		31-Dec-21
C19IMPACT_3550	Trinidad and Tobago	20-Dec-21	MoH	N	N	N	N	N	N	25.00		22-Dec-21	23-Dec-21	24-Dec-21		31-Dec-21
C19IMPACT_3551	Trinidad and Tobago	11-Dec-21	MoH	N	N	N	N	N	Y		4.09	21-Dec-21	24-Dec-21	24-Dec-21		31-Dec-21
C19IMPACT_3552	Trinidad and Tobago	13-Dec-21	MoH	N	N	N	N	N	Y	10.80		21-Dec-21	24-Dec-21	24-Dec-21		31-Dec-21
C19IMPACT_3553	Trinidad and Tobago	13-Dec-21	MoH	N	N	N	N	N	Y	14.00		21-Dec-21	24-Dec-21	24-Dec-21		31-Dec-21
C19IMPACT_3554	Trinidad and Tobago	13-Dec-21	MoH	N	N	N	N	N	Y	14.10		21-Dec-21	24-Dec-21	24-Dec-21		31-Dec-21
C19IMPACT_3555	Trinidad and Tobago	13-Dec-21	MoH	N	N	N	N	N	Y		9.00	21-Dec-21	24-Dec-21	24-Dec-21		31-Dec-21
C19IMPACT_3556	Trinidad and Tobago	13-Dec-21	MoH	N	N	N	N	N	Y		7.00	21-Dec-21	24-Dec-21	24-Dec-21		31-Dec-21
C19IMPACT_3557	Trinidad and Tobago	14-Dec-21	MoH	N	N	N	N	N	Y	13.96		21-Dec-21	24-Dec-21	24-Dec-21		31-Dec-21
C19IMPACT_3558	Trinidad and Tobago	15-Dec-21	MoH	N	N	N	N	N	Y		5.98	21-Dec-21	24-Dec-21	24-Dec-21		31-Dec-21
C19IMPACT_3559	Trinidad and Tobago	15-Dec-21	MoH	N	N	N	N	N	Y		5.45	21-Dec-21	24-Dec-21	24-Dec-21		31-Dec-21
C19IMPACT_3560	Trinidad and Tobago	15-Dec-21	MoH	N	N	N	N	N	Y		7.61	21-Dec-21	24-Dec-21	24-Dec-21		31-Dec-21
C19IMPACT_3561	Trinidad and Tobago	15-Dec-21	MoH	N	N	N	N	N	Y		8.95	21-Dec-21	24-Dec-21	24-Dec-21		31-Dec-21
C19IMPACT_3562	Trinidad and Tobago	15-Dec-21	MoH	N	N	N	N	N	Y		3.48	21-Dec-21	24-Dec-21	24-Dec-21		31-Dec-21
C19IMPACT_3563	Trinidad and Tobago	15-Dec-21	MoH	N	N	N	N	N	Y		3.23	21-Dec-21	24-Dec-21	24-Dec-21		31-Dec-21
C19IMPACT_3564	Trinidad and Tobago	15-Dec-21	MoH	N	N	N	N	N	Y	9.70		21-Dec-21	24-Dec-21	24-Dec-21		31-Dec-21
C19IMPACT_3565	Trinidad and Tobago	15-Dec-21	MoH	N	N	N	N	N	Y	8.50		21-Dec-21	24-Dec-21	24-Dec-21		31-Dec-21
C19IMPACT_3566	Trinidad and Tobago	15-Dec-21	CARPHA	N	N	N	N	N	Y	11.30		22-Dec-21	24-Dec-21	24-Dec-21	24-Dec-21	31-Dec-21
C19IMPACT_3567	Trinidad and Tobago	15-Dec-21	CARPHA	N	N	N	N	N	Y	11.57		22-Dec-21	24-Dec-21	24-Dec-21	24-Dec-21	31-Dec-21
C19IMPACT_3568	Saint Vincent and the Grenadines	16-Dec-21	CARPHA	N	N	N	N	N	N	17.62		22-Dec-21	24-Dec-21		24-Dec-21	31-Dec-21
C19IMPACT_3569	Saint Vincent and the Grenadines	17-Dec-21	CARPHA	N	N	N	N	N	N	16.94		22-Dec-21	24-Dec-21		24-Dec-21	31-Dec-21
C19IMPACT_3570	Saint Vincent and the Grenadines	17-Dec-21	CARPHA	N	N	N	N	N	N	18.16		22-Dec-21	24-Dec-21		24-Dec-21	31-Dec-21
C19IMPACT_3571	Saint Vincent and the Grenadines	17-Dec-21	CARPHA	N	N	N	N	N	N	16.26		22-Dec-21	24-Dec-21		24-Dec-21	31-Dec-21
C19IMPACT_3572	Saint Vincent and the Grenadines	17-Dec-21	CARPHA	N	N	N	N	N	N	18.77		22-Dec-21	24-Dec-21		24-Dec-21	31-Dec-21
C19IMPACT_3573	Trinidad and Tobago	21-Dec-21	MoH	N	N	N	N	N	N	19.00		22-Dec-21	26-Dec-21	31-Dec-21		31-Dec-21
C19IMPACT_3574	Trinidad and Tobago	21-Dec-21	MoH	N	N	N	N	N	N	19.00		22-Dec-21	26-Dec-21	31-Dec-21		31-Dec-21
C19IMPACT_3575	Trinidad and Tobago	21-Dec-21	MoH	N	N	N	N	N	N	18.00		22-Dec-21	26-Dec-21	31-Dec-21		31-Dec-21
C19IMPACT_3576	Trinidad and Tobago	21-Dec-21	MoH	N	N	N	N	N	N	15.00		22-Dec-21	26-Dec-21	31-Dec-21		31-Dec-21
C19IMPACT_3577	Trinidad and Tobago	21-Dec-21	MoH	N	N	N	N	N	N	22.00		22-Dec-21	26-Dec-21	31-Dec-21		31-Dec-21
C19IMPACT_3578	Trinidad and Tobago	22-Dec-21	MoH	N	N	N	N	N	Y	27.00		24-Dec-21	26-Dec-21	31-Dec-21		31-Dec-21
C19IMPACT_3579	Saint Kitts and Nevis	19-Dec-21	CARPHA	N	N	N	N	N	N	15.15		24-Dec-21	26-Dec-21		26-Dec-21	
C19IMPACT_3580	Saint Kitts and Nevis	20-Dec-21	CARPHA	N	N	N	N	N	N	17.31		24-Dec-21	26-Dec-21		26-Dec-21	31-Dec-21

C19IMPACT_3581	Saint Kitts and Nevis	19-Dec-21	CARPHA	N	N	N	N	N	N	23.04		24-Dec-21	26-Dec-21		26-Dec-21	31-Dec-21
C19IMPACT_3582	Saint Vincent and the Grenadines	17-Dec-21	CARPHA	N	N	N	N	N	N	17.03		24-Dec-21	26-Dec-21		26-Dec-21	31-Dec-21
C19IMPACT_3583	Saint Vincent and the Grenadines	18-Dec-21	CARPHA	N	N	N	N	N	N	19.50		24-Dec-21	26-Dec-21		26-Dec-21	31-Dec-21
C19IMPACT_3584	Saint Vincent and the Grenadines	20-Dec-21	CARPHA	N	N	N	N	N	N	15.66		24-Dec-21	26-Dec-21		26-Dec-21	31-Dec-21
C19IMPACT_3585	Saint Vincent and the Grenadines	20-Dec-21	CARPHA	N	N	N	N	N	N	16.06		24-Dec-21	26-Dec-21		26-Dec-21	31-Dec-21
C19IMPACT_3586	Grenada	13-Dec-21	CARPHA	N	N	N	N	N	N	18.41		24-Dec-21	26-Dec-21		26-Dec-21	31-Dec-21
C19IMPACT_3587	Grenada	21-Dec-21	CARPHA	N	N	N	N	N	N	26.22		24-Dec-21	26-Dec-21		26-Dec-21	
C19IMPACT_3588	Grenada	17-Dec-21	CARPHA	N	N	N	N	N	N	16.81		24-Dec-21	26-Dec-21		26-Dec-21	
C19IMPACT_3589	Trinidad and Tobago	16-Dec-21	MoH	N	N	N	N	N	N	18.00		20-Dec-21	30-Dec-21	31-Dec-21		21-Jan-22
C19IMPACT_3590	Trinidad and Tobago	16-Dec-21	MoH	N	N	N	N	N	N	14.00		20-Dec-21	30-Dec-21	31-Dec-21		21-Jan-22
C19IMPACT_3591	Trinidad and Tobago	16-Dec-21	MoH	N	N	N	N	N	N	18.00		20-Dec-21	30-Dec-21	31-Dec-21		21-Jan-22
C19IMPACT_3592	Trinidad and Tobago	16-Dec-21	MoH	N	N	N	N	N	N	19.00		20-Dec-21	30-Dec-21	31-Dec-21		21-Jan-22
C19IMPACT_3593	Trinidad and Tobago	18-Dec-21	MoH	N	N	N	N	N	N	14.00		20-Dec-21	30-Dec-21	31-Dec-21		21-Jan-22
C19IMPACT_3594	Trinidad and Tobago	18-Dec-21	MoH	N	N	N	N	N	N	14.00		20-Dec-21	30-Dec-21	31-Dec-21		21-Jan-22
C19IMPACT_3595	Trinidad and Tobago	18-Dec-21	MoH	N	N	N	N	N	N	14.00		20-Dec-21	30-Dec-21	31-Dec-21		21-Jan-22
C19IMPACT_3596	Trinidad and Tobago	21-Dec-21	MoH	N	N	N	N	N	N	18.00		29-Dec-21	30-Dec-21	31-Dec-21		21-Jan-22
C19IMPACT_3597	Trinidad and Tobago	21-Dec-21	MoH	N	N	N	N	N	N	16.00		29-Dec-21	30-Dec-21	31-Dec-21		21-Jan-22
C19IMPACT_3598	Trinidad and Tobago	21-Dec-21	MoH	N	N	N	N	N	N	14.00		29-Dec-21	30-Dec-21	31-Dec-21		21-Jan-22
C19IMPACT_3599	Trinidad and Tobago	21-Dec-21	MoH	N	N	N	N	N	N	15.00		29-Dec-21	30-Dec-21	31-Dec-21		21-Jan-22
C19IMPACT_3600	Trinidad and Tobago	21-Dec-21	MoH	N	N	N	N	N	N	16.00		29-Dec-21	30-Dec-21	31-Dec-21		21-Jan-22
C19IMPACT_3601	Trinidad and Tobago	21-Dec-21	MoH	N	N	N	N	N	N	15.00		29-Dec-21	30-Dec-21	31-Dec-21		21-Jan-22
C19IMPACT_3602	Trinidad and Tobago	21-Dec-21	MoH	N	N	N	N	N	N	14.00		29-Dec-21	30-Dec-21	31-Dec-21		21-Jan-22
C19IMPACT_3603	Trinidad and Tobago	22-Dec-21	MoH	N	N	N	N	N	N	14.00		29-Dec-21	30-Dec-21	31-Dec-21		21-Jan-22
C19IMPACT_3604	Trinidad and Tobago	22-Dec-21	MoH	N	N	N	N	N	N	21.00		29-Dec-21	30-Dec-21	31-Dec-21		21-Jan-22
C19IMPACT_3605	Trinidad and Tobago	22-Dec-21	MoH	N	N	N	N	N	N	12.00		29-Dec-21	30-Dec-21	31-Dec-21		21-Jan-22
C19IMPACT_3606	Trinidad and Tobago	22-Dec-21	MoH	N	N	N	N	N	N	16.00		29-Dec-21	30-Dec-21	31-Dec-21		21-Jan-22
C19IMPACT_3607	Trinidad and Tobago	22-Dec-21	MoH	N	N	N	N	N	N	17.00		29-Dec-21	30-Dec-21	31-Dec-21		21-Jan-22
C19IMPACT_3608	Trinidad and Tobago	22-Dec-21	MoH	N	N	N	N	N	N	17.00		29-Dec-21	30-Dec-21	31-Dec-21		21-Jan-22
C19IMPACT_3609	Trinidad and Tobago	24-Dec-21	MoH	N	N	N	N	N	N	13.00		29-Dec-21	30-Dec-21	31-Dec-21		21-Jan-22
C19IMPACT_3610	Trinidad and Tobago	28-Dec-21	MoH	N	N	N	N	N	N	15.00		29-Dec-21	30-Dec-21	31-Dec-21		21-Jan-22
